# The complete annotated plastome sequences of six genera in the tropical woody Polygonaceae

**DOI:** 10.1186/s12870-024-05144-y

**Published:** 2024-05-17

**Authors:** Janelle M. Burke, Daniel M. Koenemann

**Affiliations:** 1 Dept. of Biology, Howard University, Washington, District of Columbia USA; 2 Biology Department, Claflin University, Orangeburg, South Carolina USA; 3 Catholic Distance University, Charles Town, West Virginia USA

**Keywords:** Illumina, GetOrganelle, GeSeq, CHLOROBOX, tRNAscan-SE, Neotropics

## Abstract

**Background:**

The Polygonaceae is a family well-known for its weeds, and edible plants, *Fagopyrum* (buckwheat) and *Rheum* (rhubarb), which are primarily herbaceous and temperate in distribution. Yet, the family also contains a number of lineages that are principally distributed in the tropics and subtropics. Notably, these lineages are woody, unlike their temperate relatives. To date, full-genome sequencing has focused on the temperate and herbaceous taxa. In an effort to increase breadth of genetic knowledge of the Polygonaceae, we here present six fully assembled and annotated chloroplast genomes from six of the tropical, woody genera: *Coccoloba rugosa* (a narrow and endangered Puerto Rican endemic), *Gymnopodium floribundum*, *Neomillspaughia emarginata*, *Podopterus mexicanus*, *Ruprechtia coriacea*, and *Triplaris cumingiana*.

**Results:**

These assemblies represent the first publicly-available assembled and annotated plastomes for the genera *Podopterus*, *Gymnopodium*, and *Neomillspaughia*, and the first assembled and annotated plastomes for the species *Coccoloba rugosa*, *Ruprechtia coriacea*, and *Triplaris cumingiana*. We found the assembled chloroplast genomes to be above the median size of Polygonaceae plastomes, but otherwise exhibit features typical of the family. The features of greatest sequence variation are found among the *ndh* genes and in the small single copy (SSC) region of the plastome. The inverted repeats show high GC content and little sequence variation across genera. When placed in a phylogenetic context, our sequences were resolved within the Eriogonoideae.

**Conclusions:**

These six plastomes from among the tropical woody Polygonaceae appear typical within the family. The plastome assembly of *Ruprechtia coriacea* presented here calls into question the sequence identity of a previously published plastome assembly of *R. albida*.

**Supplementary Information:**

The online version contains supplementary material available at 10.1186/s12870-024-05144-y.

## Background

The Polygonaceae family of plants is well-known for its weedy taxa, such as docks and sorrels (*Rumex* L.), Japanese knotweed (*Reynoutria* Houtt.), and *Persicaria* (L.) Mill. / *Polygonum* L. (knotweeds) [1, 2]. The family is also well-known for its edible taxa such as *Fagopyrum* Mill. (buckwheat) and *Rheum* L. (rhubarb) [3]. All of these taxa are herbaceous and primarily temperate in distribution [1, 2]. Within the Polygonaceae, there also exist several clades of primarily woody and exclusively tropical taxa [1, 2]. These groups include large genera such as *Coccoloba* P.Browne (ca. 200 species), as well as more moderately-sized genera such as *Triplaris* Loefl. and *Ruprechtia* C.A.Mey. (a few dozen species each) [1, 2].

The temperate and herbaceous taxa of the Polygonaceae are not only more readily called to mind, they have also been the subject of much of the plant science work in the family. This is true particularly of genetic work, much of it motivated by systematics research [4–6]. The six assembled nuclear genomes of the Polygonaceae listed on GenBank [7] as of November 2023, are all temperate in distribution and herbaceous in habit. Similarly, of the 462 assembled chloroplast genomes (“plastomes”) of the Polygonaceae listed on GenBank [7], as of November 2023, 426 (92%) of them are of temperate and herbaceous taxa in the Polygonaceae. As a result of this distribution of sequences, there exists latent diversity not represented in Polygonaceae nuclear genomes, plastomes, and mitogenomes in genera of the tropical, woody Polygonaceae, such as *Coccoloba*, *Gymnopodium* Rolfe, *Neomillspaughia* S.F.Blake, *Podopterus* Bonpl., *Ruprechtia*, and *Triplaris*.

Some genera of the tropical woody Polygonaceae are relatively species-poor: *Neomillspaughia* contains two species of large shrubs, both endemic to Central America. The genus is closely allied with the genus *Podopterus* [1] and with the genus *Coccoloba* [1, 8]. *Podopterus* contains three species of large shrubs, all endemic to Central America. *Gymnopodium* contains three species of large shrubs also all endemic to Central America [1]. *Coccoloba*, *Triplaris*, and *Ruprechtia*, are more species-rich. *Ruprechtia* is a genus containing approximately 20 species of small trees and large shrubs, present mostly in tropical dry forests from Central America to northern Argentina. *Triplaris* is a genus also containing approximately 20 species of medium-sized trees and lianas. In contrast to *Ruprechtia*, species of *Triplaris* are typically present in low elevation rain forests [9].

*Coccoloba* (Polygonaceae) is the largest of these genera, with some 150–200 species [1, 8]. The genus is composed of trees, shrubs, and lianas native throughout the tropics of the New World, but mostly confined to low elevations. Some species of *Coccoloba*, such as *Coccoloba uvifera* (L.) L., are extremely widespread, occurring along the coasts of North, Central, and South America, as well as nearly all of the islands of the Caribbean [8, 10, 11]. Other species, such as *Coccoloba rugosa* Desf., are endemic to a single island in the Caribbean (Puerto Rico; [10]). This species has been recognized as endangered since the 1990s [12].

We here improve the understanding of genetics in the tropical, woody Polygonaceae by providing the assembled and annotated chloroplast genomes of six species (in six genera) in this group of plants: *Coccoloba rugosa*, *Gymnopodium floribundum* Rolfe, *Neomillspaughia emarginata* S.F.Blake, *Podopterus mexicanus* Bonpl., *Ruprechtia coriacea* S.F.Blake, and *Triplaris cumingiana* Fisch. & C.A.Mey. ex C.A.Mey. (Table 1). We also compare the genomes of these six species, highlight areas of genetic divergence, and place them in a phylogenetic context.

**Table 1:** The source materials and results of the chloroplast assemblies. Herbarium acronyms following Index Herbariorum [13]

Species	Sequence Read Archive accession number	Source material (herbarium)	Length of assembly (bp)	Median coverage	GC content
*Coccoloba rugosa*	SRX22117935 (https://www.ncbi.nlm.nih.gov/sra/SRX22117935)	*D.M.Koenemann and K.Wallick 081–19* (Howard University Herbarium [HUDC] Barcode Number: HUDC00010700)	168,901	98X	36.7%
*Gymnopodium floribundum*	SRX22117901 (https://www.ncbi.nlm.nih.gov/sra/SRX22117901)	*J.M.Burke 48* (L.H. Bailey Hortorium Herbarium [BH] Barcode Number: BH000341552)	168,651	74.8X	36.8%
*Neomillspaughia emarginata*	SRX22117878 (https://www.ncbi.nlm.nih.gov/sra/SRX22117878)	*J.M.Burke 66* (L.H. Bailey Hortorium Herbarium [BH] Barcode Number: BH000341551)	169,915	62.5X	36.5%
*Podopterus mexicanus*	SRX22117879 (https://www.ncbi.nlm.nih.gov/sra/SRX22117879)	*J.M.Burke 27* (L.H. Bailey Hortorium Herbarium [BH] Barcode Number: BH000341550)	170,399	133.8X	36.5%
*Ruprechtia coriacea*	SRX22117893 (https://www.ncbi.nlm.nih.gov/sra/SRX22117893)	*J.R.Abbott 24,975* (Fairchild Tropical Botanic Garden Herbarium [FTG] Barcode Number: FTG00145332)	170,640	70.4X	36.4%
*Triplaris cumingiana*	SRX22117894 (https://www.ncbi.nlm.nih.gov/sra/SRX22117894)	*S.Zona 872* (Fairchild Tropical Botanic Garden Herbarium [FTG] Barcode Number: FTG103484)	171,221	86.4X	36.3%

## Methods

The authors and their collaborators collected leaf material from living specimens of each of the six species included in this study. These collections are vouchered through herbarium specimens (Supplement 1). All identifications of the specimens were verified by the authors.

Leaf material destined for DNA extraction was preserved in silica gel and then frozen at -20C. The remaining material was used to generate a voucher specimen (Table 1). Whole genomic DNA was extracted using protocols outlined by Koenemann and Burke [8]. The DNA sample was cleaned with the Clean and Concentrator kit (Zymo Research, Irvine, CA). Whole genomic libraries were prepared using the NEBNext Ultra II DNA PCR-free Library Prep kit (New England BioLabs, Ipswich, MA). Whole genomic shotgun sequencing was conducted on an Illumina NovaSeq 6000, using a 500 bp insert size and 150 bp paired-end reads (University of South Carolina Functional Genomics Core Facility, Columbia, SC). Sequencing was scaled to generate 15 million reads per sample. These reads have been uploaded to the Sequence Read Archive [14] (Table 1).

We checked the reads for anomalies with FastQC v.0.11.8 [15] and did not find any. We then used the reads to generate a primary assembly for the chloroplast genome using GetOrganelle v.1.6.2d [16]. We did not clean the reads, as requested by GetOrganelle, so as not to interfere with the internal read cleaning of GetOrganelle. We did not provide a seed plastome to GetOrganelle as there did not exist an assembled plastome from a closely related taxon at the time we were making our assemblies (GetOrganelle uses an internal database as it's default when no seed is provided). We provided the following additional flags to GetOrganelle: -R 15 -k 21,45,65,85,105 -F embplant_pt. We annotated the assembled genome using GeSeq in the CHLOROBOX web platform [17], utilizing the added functionality of tRNAscan-SE v2.0.7 [18], but otherwise accepting the default settings.

Using the GeSeq annotation, we extracted the sequences of each feature for each species. The GeSeq annotation was returned in GFF3 format. We converted this to BED format using a custom script (see Supplement 2 for code). We then used BEDTools [19] to extract the sequence of each feature in FASTA format. We then aligned the sequences of each feature for all species using MAFFT v7.505 [20]. For each aligned feature, we then calculated the average, pairwise, per-site nucleotide diversity (π) as a measure of sequence divergence across the six plastomes. We used the pegas v.1.1 [21] package in R [22] to calculate the π values.

During the course of this study, we became aware of a possible misidentification of an existing GenBank accession. As part of our efforts to investigate this misidentification, we reconstructed a phylogeny of the Polygonaceae. The sampling for this phylogeny generally followed that of Zhang et al. [5] with the addition of the six plastomes assembled by us in this paper. We aligned all the plastomes using MAFFT with the additional flag “—adjustdirectionaccurately”. We examined the alignment using the NCBI Multiple Alignment Viewer v.1.25.0 [23] and Geneious Prime v2023.0.1 (https://www.geneious.com, [24]). We did not discover any anomalies. We removed one of the two inverted repeats from the aligned plastomes prior to phylogenetic analysis in order not to bias the contribution of the sequences in these regions.

Following alignment, we assessed the likely nucleotide substitution model using IQ-TREE v.2.1.3 [25]. The model selected by IQ-TREE was GTR + F + R5. We subsequently conducted a (maximum likelihood) phylogenetic analysis in IQ-TREE using the GTR + F + R5 model. The analysis utilized 1000 search replicates to assess topology and 1000 rapid bootstraps to assess support (Code: iqtree -s InFile.phy –alrt 1000 -B 1000 -lmap 2000) (See Supplement 3 and Supplement 4).

## Results

We were able to successfully assemble a complete, circular chloroplast genome (“plastome”) for each of the six species (Supplement 5). The sizes of the six plastomes ranged from 168,651 bp – 171,221 bp, with the GC content varying between 36.3 –36.8% (Table 1). These plastome sizes are larger than has been reported for other genera of the Polygonaceae. For example, chloroplast genome size in *Persicaria* has been reported at 160,585 bp [26], in *Rumex* at 159,087 bp [6], and in *Rhuem* at 161,563 bp [27].

We were able to successfully annotate all six of the assembled plastomes (Supplement 6). For all six species the annotation identified 164 features: 37 tRNAs, 10 rRNAs, 103 exons, and 14 introns. This is similar to what has been documented elsewhere (e.g. [26]). These features are located in a large single copy region (LSC) (94 features), a small single copy region (SSC) (14 features), and two inverted repeat regions (IR) (28 features each).

The overall mean value of π across all features was 0.004262, and the overall median value was 0.002875. The upper quartile of π values was 0.006333. Among the different types of features, the most variable were the introns (mean: 0.0084) and the least variable the rRNAs (mean: 0.000555) (Table 2).

**Table 2:** The π values for different types of features identified in the annotation

Feature	Mean	Median	Min	Max
tRNA	0.001117	0.000000	0.000000	0.021005
Exon	0.005183	0.004712	0.000000	0.017647
Intron	0.008442	0.007464	0.000502	0.029104
rRNA	0.000555	0.000403	0.000000	0.001303

**Fig. 1:**
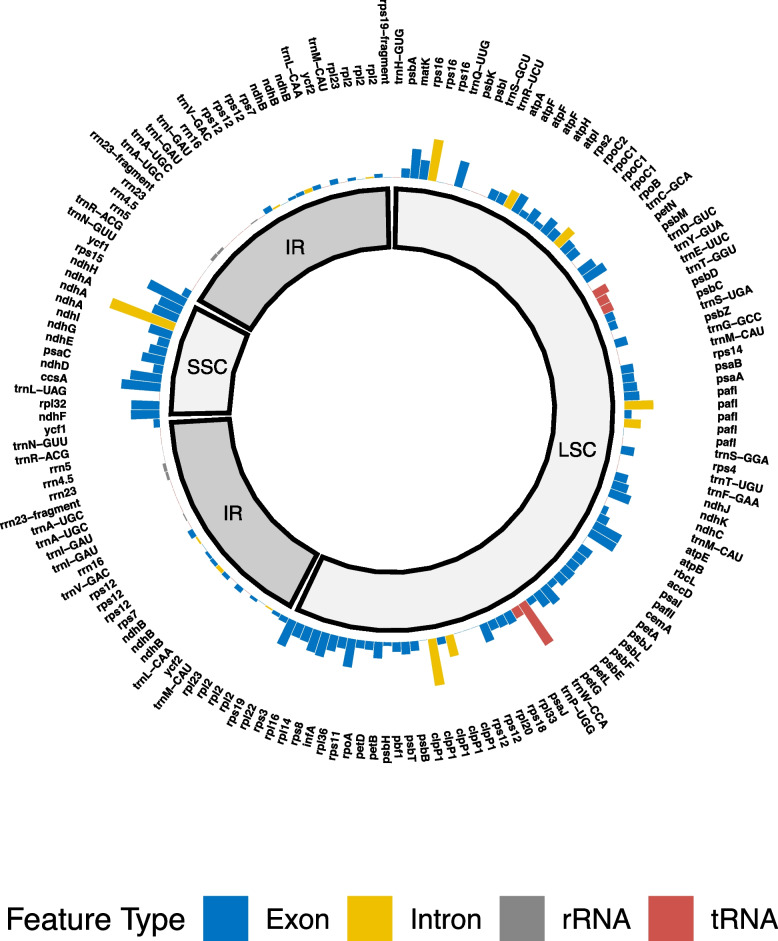
The values of π for each annotated feature, as calculated across the six assembled plastomes. The features are placed within their proper chloroplast region and in syntenic order according to the annotation. The colors represent the feature type (exon, intron, tRNA, rRNA). The variation is to scale (values of π range from 0.0 to 0.029). Absolute π values for each annotated feature are available in Supplement 7

The features in the upper quartile, in order from lowest to highest π value, are: *trnY-GUA*, *pafI*, *infA*, *ndhK*, *petL*, *petB*, *pafI*, *rpoC2*, *petN*, *ndhG*, *atpF*, *rpl20*, *rps16*, *atpF*, *psbE*, *clpP1*, *rpoC1*, *ndhC*, *rpl14*, *ndhE*, *ndhA* (exon), *psbM*, *rps8*, *ndhH*, *psbK*, *ndhA* (exon), *rpl22*, *rpl32*, *rps11*, *ndhF*, *pafI*, *accD*, *matK*, *rbcL*, *ndhD*, *ccsA*, *rps15*, *rps16*, *clpP1*, *trnW-CCA*, *ndhA* (intron) (Fig. 1, Supplement 7).

Of the features that have π values in the top quartile, 11 are in the SSC (of 14 total features in the SSC, 79%), and the other 30 are in the LSC (of 94 total features in the LSC, 32%). None of the features in the upper quartile of π values were found in either of the IRs. In fact, only a single feature in the IRs (*ycf1*, 0.00307) has a π value above the median π value. With respect to feature type, 2 of the features in the upper quartile of π values are tRNA (of 37 total tRNAs, 5%), 8 are introns (of 14 total introns, 57%), and 31 are exons (of 103 total exons, 30%).

The GC content among the six species and genera is nearly identical, varying only half a percent. GC content is notably highest in the IRs and adjacent regions of all six species, rising above 50%. This is the only location in the plastome where this is the case.

**Fig. 2:**
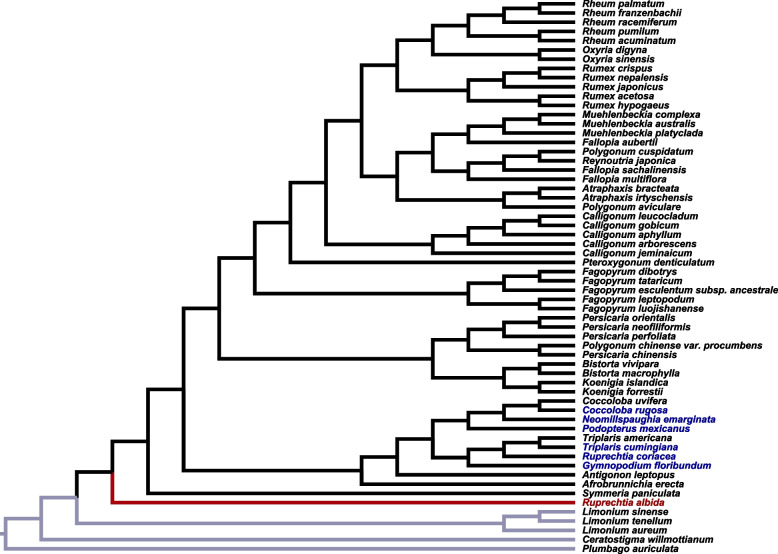
A plastome phylogeny of the Polygonaceae reconstructed using previously assembled whole plastomes and the plastomes assembled in this study. The maximum likelihood phylogeny was generated using IQ-TREE. Branch lengths are not to scale. All nodes have bootstrap support above 70. Outgroups are highlighted with gray branches. Taxa represented by sequences generated in this study are highlighted in blue. The *Ruprechtia albida* specimen from Zhang et al. [5]. is highlighted in red

The phylogeny was fully resolved, with all nodes representing lineage bifurcations (Fig. 2). Moreover bootstrap support was above 70 for all nodes. The topology of our phylogeny is broadly reflective of those found in other phylogenetic studies in the Polygonaceae. In particular, our results are largely congruent with the phylogeny of Zhang et al. [5]. Differences include some of the relationships among species in *Rumex*, and a different placement of *Afrobrunnichia* (sister to Persicarieae in Zhang et al. [5] but sister to Eriogonoideae in ours). Importantly, the phylogeny of Zhang et al. [5] resolved *Ruprechtia* as sister to the entire Polygonaceae whereas ours resolved *Ruprechtia* as sister to *Triplaris* and within the Eriogonoideae.

## Discussion

### Comparisons with existing assemblies

We here present six successfully assembled and annotated chloroplast genomes from six genera of the tropical woody Polygonaceae: *Coccoloba rugosa*, *Gymnopodium floribundum*, *Neomillspaughia emarginata*, *Podopterus mexicanus*, *Ruprechtia coriacea*, and *Triplaris cumingiana*. To our knowledge, these represent the first assembled and annotated plastomes for the genera *Podopterus*, *Gymnopodium*, and *Neomillspaughia*. Additionally, to our knowledge, these represent the first assembled and annotated plastomes for the species *Coccoloba rugosa*, *Ruprechtia coriacea*, and *Triplaris cumingiana*.

The sizes of the plastomes assembled here are above the median value of those reported for genera of the Polygonaceae. Our plastomes ranged in size from 168,651 bp to 171,221 bp. Of the assembled plastomes of the Polygonaceae available on GenBank [7], as of November 2023, the sizes range from 179,064 bp to 128,371 bp, with a mean size of 160,633 bp and a median size of 161,093 bp. As a result, all six of the plastomes presented here are above the average size of plastomes in the family.

For three of the genera, *Coccoloba*, *Ruprechtia*, and *Triplaris*, there exist recent assemblies to which we can compare our own. A previously assembled *C. uvifera* plastome (GenBank: NC_068873.1) reports a size of 169,369 bp, similar to the one we recovered here for *C. rugosa* (168,901 bp). Likewise, an existing assembly of the *T. americana* L. plastome (GenBank: NC_068874.1) is listed as 171,340 bp, similar in size to the one we report here for *T. cumingiana* (171,221 bp). We do see major differences between the existing assembly of *R. albida* Pendry (GenBank: NC_068875.1) and the one we present here for *R. coriacea*. *Ruprechtia albida* is reported to have a plastome size of 157,255 bp and we here report the *R. coriacea* plastome to have a size of 170,640 bp. Additionally, aligning the sequences of *R. albida* and *R. coriacea* shows very poor sequence identity (76.1%).

One possible explanation for this sequence divergence is a difference in assembler. The GenBank record and associated publication [5] indicate that the *Ruprechtia albida* sequence was assembled using NOVOPlasty and Geneious (NC_068875.1). We assembled our plastomes using GetOrganelle. Yet, in our opinion, this explanation seems unlikely. Others [28] have conducted studies comparing plastome assemblers, using both simulated and real data. What was found is that some plastid assemblers work better than others. GetOrganelle generally performed the best but both GetOrganelle and NOVOPlasty were recommended as reliable assemblers. Differences between the assemblies were slight and both had strengths and weaknesses in different situations. Moreover, the amount of divergence between the sequences, in our experience, is consistent with a generic or familial separation in taxa, not a specific separation [6].

Another possible explanation for the sequence divergence is that one of the assemblies has been generated from a misidentified voucher specimen or is the result of contamination [29]. An NCBI BLAST [30] search of the *Ruprechtia albida* assembly using its *rbcL* sequence (the land plant barcoding gene, [31]) reveals a high sequence affinity with *Hydrangea* L. (Hydrangeaceae) and *Philadelphus* L. (Hydrangeaceae) (Table 3). Using BLAST for the same feature from our (*R. coriacea*) assembly reveals affinities to *Triplaris* (Polygonaceae), *Afrobrunnichia* Hutch. & Dalziel (Polygonaceae), *Antigonon* Endl. (Polygonaceae), and *Coccoloba* (Polygonaceae) sequences.

**Table 3:** Top results of BLAST nucleotide search of select plastid genes of GenBank accession NC_068875.1, currently identified as *Ruprechtia albida*. Search conducted on Feb. 28, 2024

Gene	Hit (taxonomic)	Percent identity	Percent coverage
*rbcL*	*Philadelphus calvescens* (Hydrangeaceae)	99.93%	100%
*ndhA*	*Hydrangea arguta* (Hydrangeaceae)	95.02%	100%
*psbA*	*Philadelphus calvescens* (Hydrangeaceae)	99.91%	100%

We have not been able to inspect the voucher listed on GenBank for the *Ruprechtia albida* specimen. It is listed simply as “voucher 19693518” with no institutional affiliation indicated. Poor voucher metadata in GenBank has been written about by others [32]. Moreover, while rare, there have been documented cases of GenBank sequences having been assigned an incorrect taxonomy [33]. And while we have been unable to verify the voucher provided by Zhang et al. [5], we are confident in our own voucher and determination, which are derived from a living specimen accessioned at the Fairchild Tropical Botanic Garden and vouchered in their herbarium (Table 1, Supplement 1).

The *Ruprechtia albida* plastome was published as part of a study examining phylogenomics in the Polygonaceae. Another avenue for examining the identity of the sequence was to add our sequences to their phylogeny and examine the placement of taxa. Zhang et al. [5] reconstructed *Ruprechtia albida* as sister to the Polygonaceae as a whole. This placement is unexpected given the previous literature placing *Ruprechtia* as sister to *Triplaris* and within the Eriogonoideae [8, 34–37]. The phylogeny we reconstructed here, including the plastomes we assembled for this study, verifies the position of their *Ruprechtia albida* sequence, but places our *Ruprechtia coriacea* plastome sequence as sister to *Triplaris* and within the Eriogonoideae. The placement of our *Ruprechtia coriacea* sequence is consistent with the placement of *Ruprechtia* species in previous studies.

Multiple lines of evidence (voucher identification, sequence affinity in BLAST search, and phylogenetic placement) all suggest that the sequence we assembled for this study is correctly connected to the taxon *Ruprechtia coriacea*, but that the sequence presented in Zhang et al. [5] and currently accessioned on GenBank is likely not correctly connected to the taxon *Ruprechtia albida*.

### Comparisons among assemblies

Among the plastomes of the six species presented in this paper, the most variable regions tended to fall within the SSC. Furthermore, among the features in the SSC, the *ndh* series of genes were the most variable. Moreover, the *ndh* genes located outside of the SSC were also within the top quartile of π values. The *ndh* genes, both those located within the SSC and those outside of it, code for protein elements of the NADH dehydrogenase-like complex. This complex is a membrane-embedded electron transport protein, very similar in structure to, and proposed to be homologous with respiratory complex I in the mitochondria [38]. Though its function was initially somewhat mysterious, it is now thought to be involved in the photosynthetic process, primarily in an optimizing role by helping to reduce the oxidative stress produced by processes such as photolysis [38, 39].

As the sequencing of chloroplast features and genomes has increased, variability in the *ndh* genes has become a known phenomenon among the land plants [39]. Additionally, certain groups of plants, notably epiphytes and parasitic plants, may lack some or all of the *ndh* genes [39]. As a result, our finding of variability in the SSC and *ndh* genes among six genera of the tropical woody Polygonaceae is unsurprising. Other studies using similar metrics (π) to quantify sequence divergence in the Polygonaceae have also found high variation in the SSC and among the *ndh* genes in both *Rumex* [6] and *Rheum* [40]. The list of genes in the top quartile of π values in these studies is nearly identical to the list of genes in the top quartile of π values in this study.

Two other patterns of variability that we noticed in our sequences were a strikingly low sequence variation in the IRs (only a single feature above the median π value), and a high GC content (above 50%) in these same regions. Further investigation revealed that this pattern was also observed in *Rumex* [6]. While this is not enough evidence to say that these patterns are common, it is at least consistent with the otherwise ordinary characterization of the plastomes assembled in this paper.

## Conclusion

These six plastomes from among the tropical woody Polygonaceae appear more or less typical within the family (462 assembled Polygonaceae plastomes on GenBank as of November 2023). They are above the median size of Polygonaceae plastomes but otherwise exhibit characteristics common in the family: the features of greatest sequence variation are found among the *ndh* genes and in the SSC, and the IRs show little sequence variation and high GC content. The plastome assembly of *Ruprechtia coriacea* presented here calls into question the sequence identity of a previously published plastome assembly of *R. albida*.

### Supplementary Information

Supplementary Material 1.

Supplementary Material 2.

Supplementary Material 3.

Supplementary Material 4.

Supplementary Material 5.

Supplementary Material 6.

Supplementary Material 7.

## Data Availability

Data generated or analyzed during this study are included in this published article [and its supplementary information files]. The raw Illumina reads used to generate the chloroplast genome assemblies are available on the Sequence Read Archive (BioProject: PRJNA1109728 [https://www.ncbi.nlm.nih.gov/bioproject/PRJNA1109728]; Samples: SRX22117935 [https://www.ncbi.nlm.nih.gov/sra/SRX22117935], SRX22117901 [https://www.ncbi.nlm.nih.gov/sra/SRX22117901], SRX22117878 [https://www.ncbi.nlm.nih.gov/sra/SRX22117878], SRX22117879 [https://www.ncbi.nlm.nih.gov/sra/SRX22117879], SRX22117893 [https://www.ncbi.nlm.nih.gov/sra/SRX22117893], SRX22117894 [https://www.ncbi.nlm.nih.gov/sra/SRX22117894]) (Table 1).

## Abbreviations

SSC: Small single copy region of the typical green plant chloroplast genome
IR: Inverted repeat region of the typical green plant chloroplast genome
LSC: Large single copy region of the typical green plant chloroplast genome
Plastome: The complete, circular genome of the chloroplast
Mitogenome: The complete genome of the mitochondria
