# Supplementary material for: The complete annotated plastome sequences of six genera in the tropical woody Polygonaceae
Source: BMC Plant Biol. 2024 May 17;24:417. doi: 10.1186/s12870-024-05144-y (PMC11100190; doi:10.1186/s12870-024-05144-y)

Supplement 1- Plant Specimen Vouchers for genomic data generated for this study

Coccoloba rugosa Desf. Voucher, *Koenemann 081-19*. Image courtesy of Howard University Herbarium.

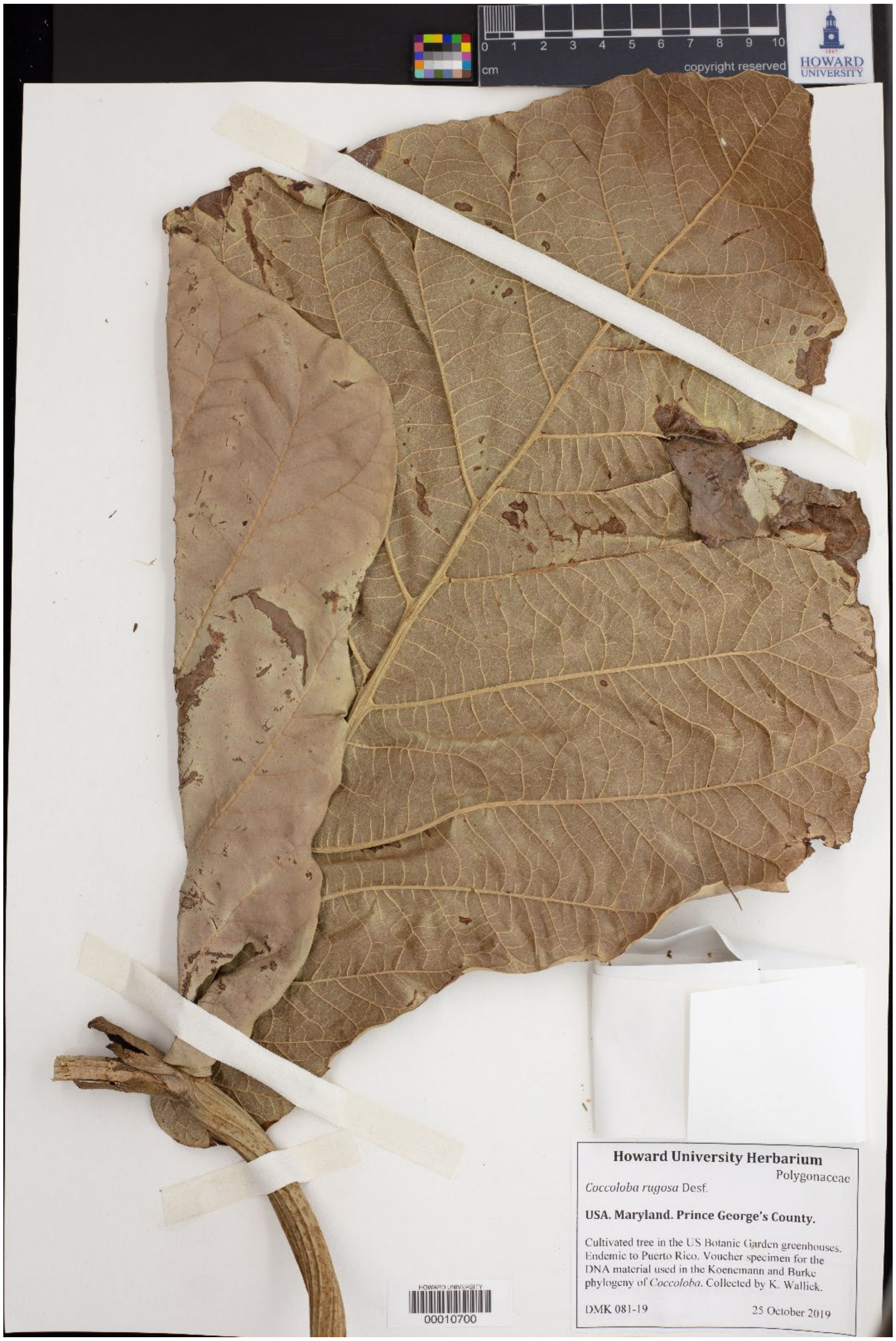

Gymnopodium floribundum Rolfe, *Burke* 48. Image provided courtesy of L.H. Bailey Hortorium Herbarium (BH).

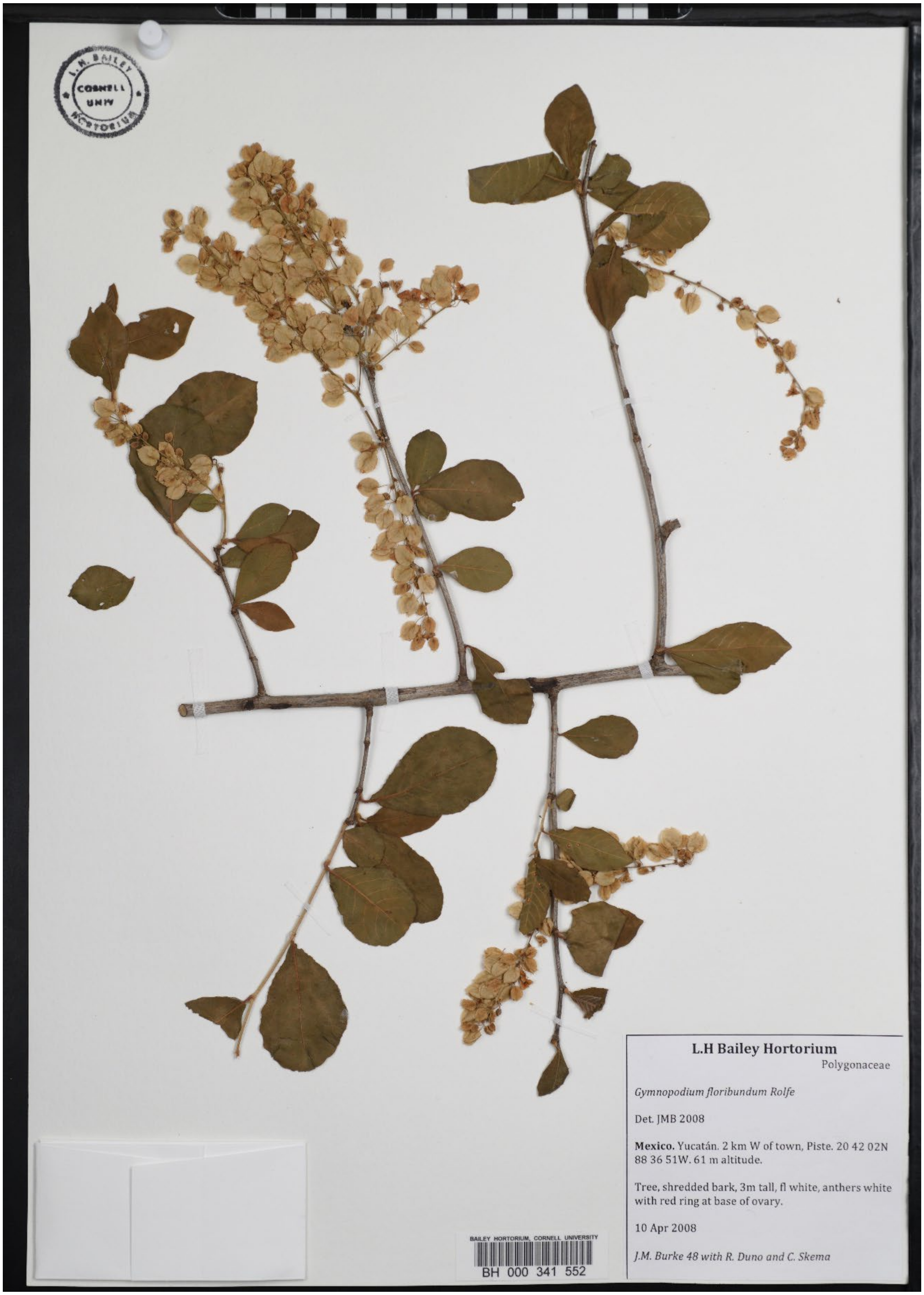

Neomillspaughia emarginata S.F. Blake*, Burke* 66. Image provided courtesy of L.H. Bailey Hortorium Herbarium (BH).

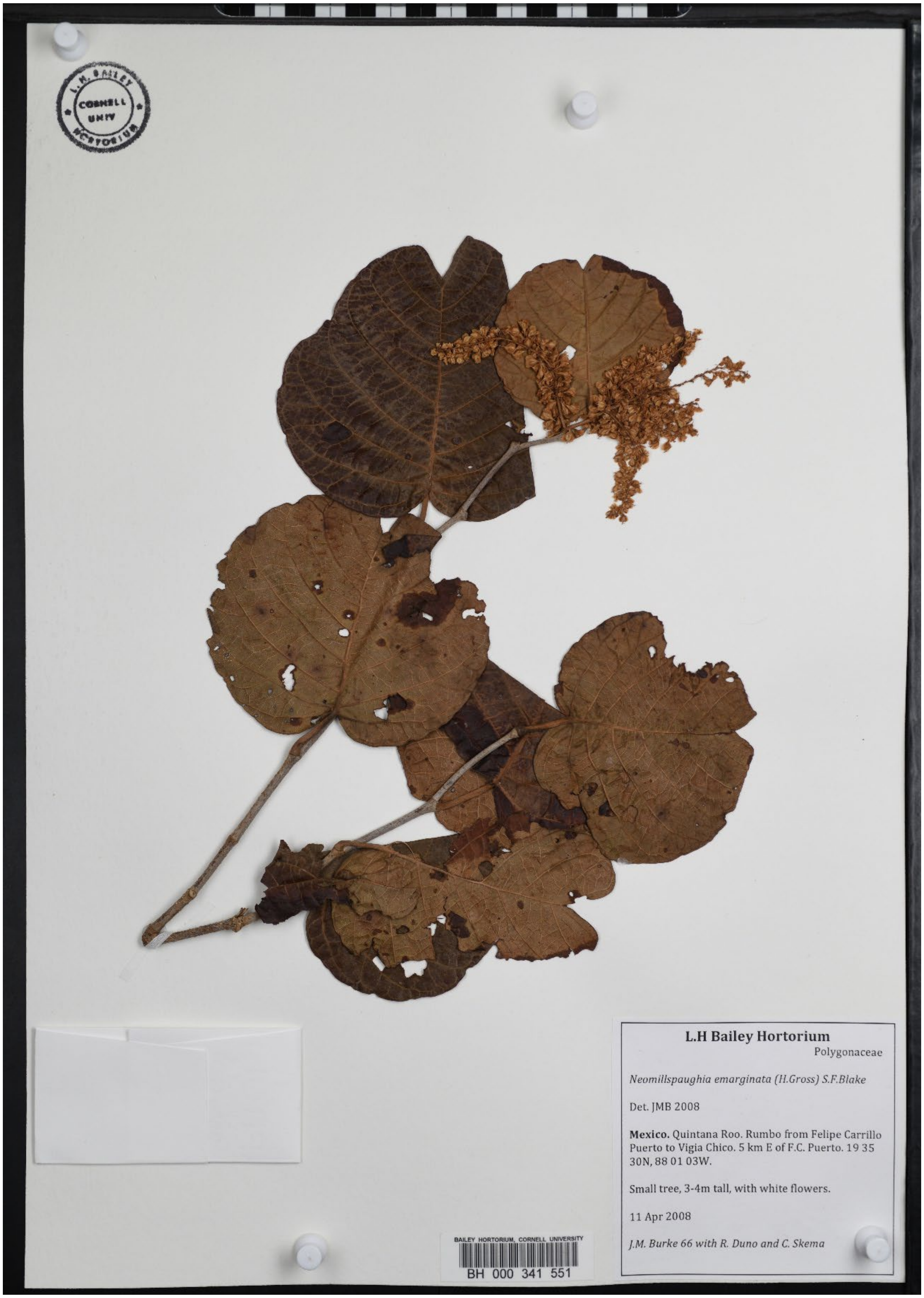

Podopterus mexicanus Bonpl.*, Burke* 37. Image provided courtesy of L.H. Bailey Hortorium Herbarium (BH).

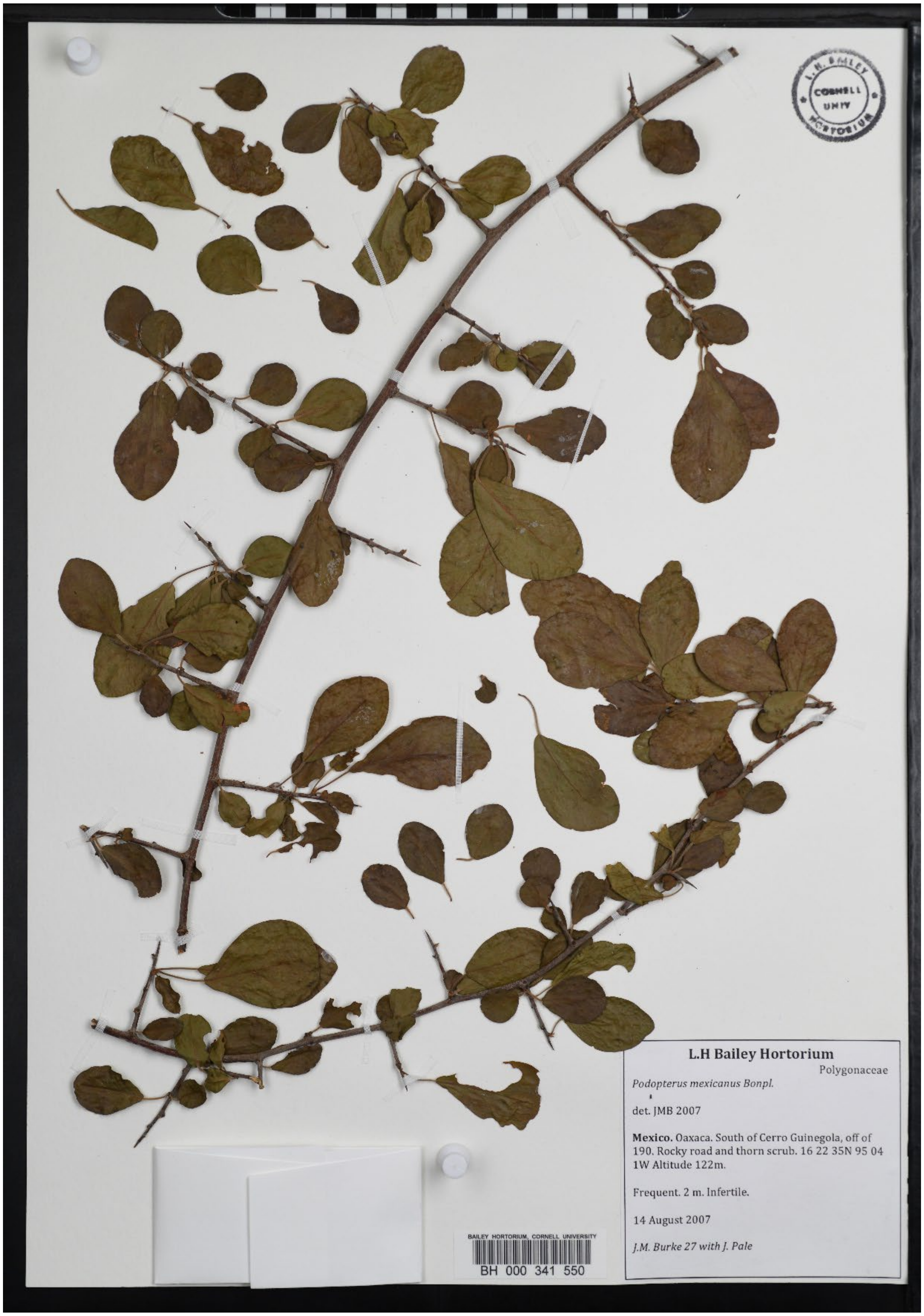

Ruprechtia coriacea (H. Karst) S.F. Blake, *Abbott #24975*. Image provided courtesy of Fairchild Tropical Botanic Garden (FTG).

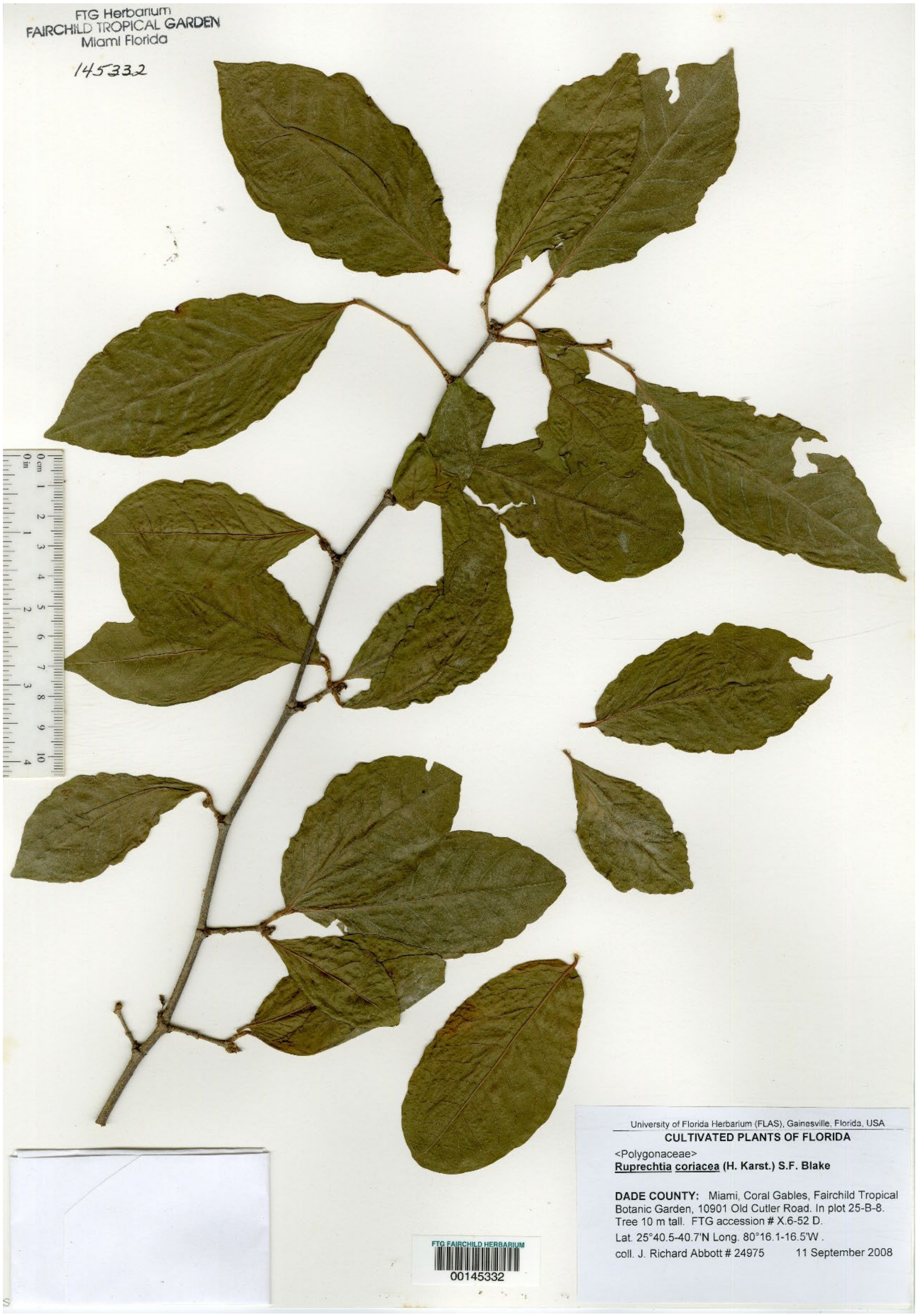

Triplaris cumingiana Fisch & C.A. Mey., *Zona 872*. Image provided courtesy of Fairchild Tropical Botanic Garden (FTG).

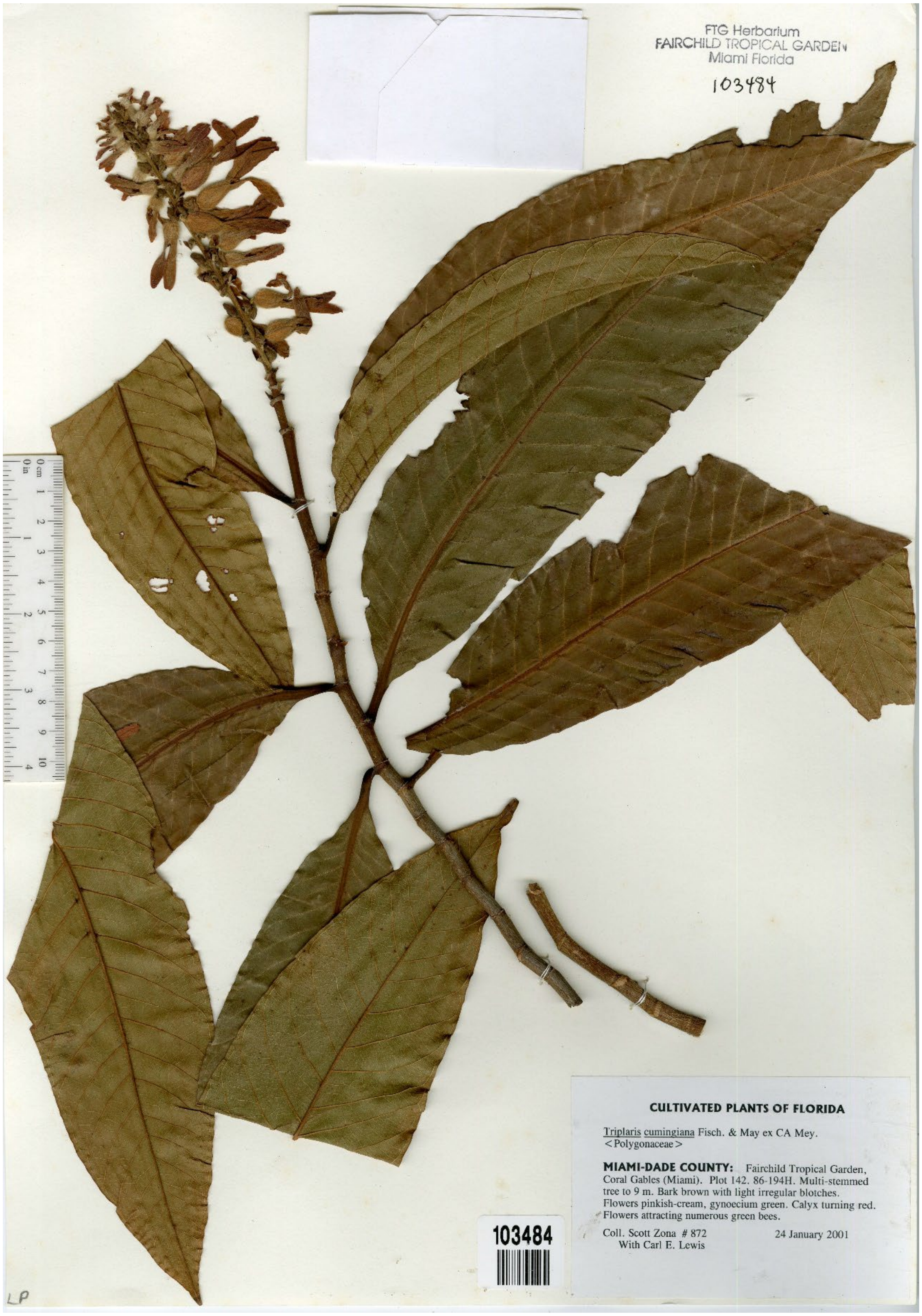

Supplement: Supplementary file 1 — Supplementary Material 1. [file 12870_2024_5144_MOESM1_ESM.pdf]
