# Supplementary material for: The complete annotated plastome sequences of six genera in the tropical woody Polygonaceae
Source: BMC Plant Biol. 2024 May 17;24:417. doi: 10.1186/s12870-024-05144-y (PMC11100190; doi:10.1186/s12870-024-05144-y)
Supplement: Supplementary file 2 — Supplementary Material 2. [file 12870_2024_5144_MOESM2_ESM.docx]

Supplement2: This file includes the code used to manipulate the annotation files provided by GeSeq. The file also includes the code necessary to calculate Pi values. The file also include the code necessary to extract the FASTA sequences from the assembled plastome. The file also includes the code necessary to make figure 1. The file is in RMD (R Markdown) format.

```{r Libraries}

################################################################################

#Supplement 2 - This file includes the code used to manipulate the annotation###

#files provided by GeSeq. The file also includes the code necessary to #########

#calculate Pi values. The file also include the code necessary to extract the###

#FASTA sequences from the assembled plastome.The file also includes the code####

#the code necessary to make figure 1############################################

################################################################################

#Libraries Required for Subsequent Code#########################################

################################################################################

library(ape)

library(bedr)

library(dplyr)

library(ggprism)

library(ggsci)

library(magrittr)

library(pegas)

library(rcartocolor)

library(RColorBrewer)

library(readr)

library(stringr)

library(tidyr)

library(tidyverse)

library(viridis)

library(wesanderson)

```

```{r GFF3toBED}

################################################################################

#Converting Gff3 Annotation Output to BED Format################################

################################################################################

fileNames <- Sys.glob("*.gff3")

for (fileName in fileNames) {

InitialFile <- read_delim(file = fileName,

delim = "\t",

skip = 4,

col_names = FALSE)

justname <- str_remove(fileName,

"_GFF3.gff3")

InitialFile %>%

separate(X9,

sep = "_",

into = c("Useless1",

"Feature",

"Useless2")) %>%

mutate(X1 = justname) %>%

select(-X6,

-X7,

-X8) %>%

filter(X3 != "gene") %>%

filter(X3 != "exon") %>%

filter(X2 != "Chloe") %>%

select(-Useless1,

-Useless2) %>%

mutate(X3 = ifelse(X3 == "CDS",

paste("Exon"),

X3)) %>%

mutate(X3 = ifelse(X3 == "intron",

paste("Intron"),

X3)) %>%

arrange(X4) %>%

select(-X2) %>%

mutate(X6 = X1) %>%

unite(Feature,

X3,

X6,

col = "Feature",

sep = "_") %>%

select(X1,

X4,

X5,

Feature) %>%

write_delim(file = paste(justname,

".bed",

sep = ""),

col_names = FALSE,

delim = "\t")

}

```

```{r CompleteBED}

################################################################################

#Modify Naming Conventions in BEDs##############################################

################################################################################

bedlist <- Sys.glob("*.bed")

for (bedfile in bedlist) {

InitialBED <- read_delim(file = bedfile,

delim = "\t",

col_names = FALSE) %>%

rename("Species" = X1,

"StartPos" = X2,

"EndPos" = X3,

"GeneName" = X4) %>%

separate(col = GeneName,

sep = "_",

into = c("FeatureName",

"FeatureType",

"SpeciesName")) %>%

unite(FeatureName,

FeatureType,

SpeciesName,

col = GeneName,

sep = "_") %>%

arrange(StartPos)

justname <- str_remove(bedfile,

".bed")

InitialBED %>%

rownames_to_column(var = "RowNM") %>%

unite(RowNM,

GeneName,

col = "GeneName",

sep = "_") %>%

select(Species,

StartPos,

EndPos,

GeneName) %>%

write_delim(file = paste(justname,

"_Complete.bed",

sep = ""),

col_names = FALSE,

delim = "\t")

}

```

```{r SortBEDs}

################################################################################

#Sort BEDs by Feature Rather than by Taxon######################################

################################################################################

featurelist <- read_delim(file = "CoccolobaRugosaT248_Complete.bed",

delim = "\t",

col_names = FALSE) %>%

select(X4) %>%

separate(X4,

into = c("Feature",

"FeatureType",

"Species"),

sep = "_") %>%

select(Feature,

FeatureType) %>%

unite(Feature,

FeatureType,

col = "Features",

sep = "_")

paste(featurelist)

featurelist <- c("1_trnH-GUG", "2_psbA", "3_matK", "4_rps16", "5_rps16", "6_rps16", "7_trnQ-UUG", "8_psbK", "9_psbI", "10_trnS-GCU", "11_trnR-UCU", "12_atpA", "13_atpF", "14_atpF", "15_atpF", "16_atpH", "17_atpI", "18_rps2", "19_rpoC2", "20_rpoC1", "21_rpoC1", "22_rpoC1", "23_rpoB", "24_trnC-GCA", "25_petN", "26_psbM", "27_trnD-GUC", "28_trnY-GUA", "29_trnE-UUC", "30_trnT-GGU", "31_psbD", "32_psbC", "33_trnS-UGA", "34_psbZ", "35_trnG-GCC", "36_trnM-CAU", "37_rps14", "38_psaB", "39_psaA", "40_pafI", "41_pafI", "42_pafI", "43_pafI", "44_pafI", "45_trnS-GGA", "46_rps4", "47_trnT-UGU", "48_trnF-GAA", "49_ndhJ", "50_ndhK", "51_ndhC", "52_trnM-CAU", "53_atpE", "54_atpB", "55_rbcL", "56_accD", "57_psaI", "58_pafII", "59_cemA", "60_petA", "61_psbJ", "62_psbL", "63_psbF", "64_psbE", "65_petL", "66_petG", "67_trnW-CCA", "68_trnP-UGG", "69_psaJ", "70_rpl33", "71_rps18", "72_rpl20", "73_rps12", "74_rps12", "75_clpP1", "76_clpP1", "77_clpP1", "78_clpP1", "79_clpP1", "80_psbB", "81_psbT", "82_pbf1", "83_psbH", "84_petB", "85_petD", "86_rpoA", "87_rps11", "88_rpl36", "89_infA", "90_rps8", "91_rpl14", "92_rpl16", "93_rps3", "94_rpl22", "95_rps19", "96_rpl2", "97_rpl2", "98_rpl2", "99_rpl23", "100_trnM-CAU", "101_ycf2", "102_trnL-CAA", "103_ndhB", "104_ndhB", "105_ndhB", "106_rps7", "107_rps12", "108_rps12", "109_rps12", "110_trnV-GAC", "111_rrn16", "112_trnI-GAU", "113_trnI-GAU", "114_trnA-UGC", "115_trnA-UGC", "116_rrn23-fragment", "117_rrn23", "118_rrn4.5", "119_rrn5", "120_trnR-ACG", "121_trnN-GUU", "122_ycf1", "123_ndhF", "124_rpl32", "125_trnL-UAG", "126_ccsA", "127_ndhD", "128_psaC", "129_ndhE", "130_ndhG", "131_ndhI", "132_ndhA", "133_ndhA", "134_ndhA", "135_ndhH", "136_rps15", "137_ycf1", "138_trnN-GUU", "139_trnR-ACG", "140_rrn5", "141_rrn4.5", "142_rrn23", "143_rrn23-fragment", "144_trnA-UGC", "145_trnA-UGC", "146_trnI-GAU", "147_trnI-GAU", "148_rrn16", "149_trnV-GAC", "150_rps12", "151_rps12", "152_rps12", "153_rps7", "154_ndhB", "155_ndhB", "156_ndhB", "157_trnL-CAA", "158_ycf2", "159_trnM-CAU", "160_rpl23", "161_rpl2", "162_rpl2", "163_rpl2", "164_rps19-fragment")

for (somefeature in featurelist) {

read_delim(file = "AllBEDs.bed",

delim = "\t",

col_names = FALSE) %>%

separate(X4,

into = c("Feature",

"FeatureType",

"Species2"),

sep = "_") %>%

unite(Feature,

FeatureType,

col = "Features",

sep = "_") %>%

rename("Species" = "X1",

"StartPos" = "X2",

"EndPos" = "X3") %>%

filter(Features == somefeature) %>%

unite(Features,

Species2,

col = "Features",

sep = "_") %>%

select("Species",

"StartPos",

"EndPos",

"Features")%>%

write_delim(file = paste(somefeature,

".feature.bed",

sep = ""),

col_names = FALSE,

delim = "\t")

}

```

```{r BEDtoFASTA}

################################################################################

#Use BEDs to Extract Sequences from Assembled Genomes###########################

################################################################################

bedlist <- Sys.glob("*.bed")

for (bedfile in bedlist) {

justname <- str_remove(bedfile,

"\\.feature.bed")

outfilename <- paste(justname,

"_feature.fasta",

sep = "")

fullcommand <- paste("bedtools getfasta -fi AllFASTAs.fasta",

" -bed ",

bedfile,

" -fo ",

outfilename,

" -name ",

sep = "")

system(paste(fullcommand))

}

```

```{r PiCalculation}

################################################################################

#Calculating Pi Values for All Features#########################################

################################################################################

ALNlist <- Sys.glob("*.fasta.ALN.fasta")

FinalResult <- tibble()

for (SomeALN in ALNlist) {

FastaMatrix <- read.dna(SomeALN,

format = "fasta",

as.matrix = TRUE)

PiDiversity <- nuc.div(FastaMatrix)

justname <- str_remove(SomeALN,

"_feature.fasta.ALN.fasta")

Vals <- c(paste(justname),

"Tropical Woody Polygonaceae",

PiDiversity)

IntermediateResult <- matrix(data = Vals,

nrow = 1,

ncol = 3) %>%

as_tibble() %>%

separate(V1,

into = c("Feature",

"FeatureCategory"),

sep = "_") %>%

rename("Dock_Sorrel" = "V2",

"Pi" = "V3")

print(Vals)

FinalResult <- bind_rows(FinalResult, IntermediateResult)

}

FinalResult %>%

unique() %>%

write_csv("WoodyPolygonaceaePi.csv",

col_names = TRUE)

```

```{r PiStats}

################################################################################

#Calculating Basic Pi Statistics################################################

################################################################################

Infile <- read_csv(file = "WoodyPolygonaceaePi.csv",

col_names = TRUE) %>%

mutate(FeatureCategory = as_factor(FeatureCategory),

Feature = as_factor(Feature),

CpRegion = as_factor(CpRegion))

summary(Infile)

str(Infile)

read_csv(file = "WoodyPolygonaceaePi.csv",

col_names = TRUE) %>%

mutate(FeatureCategory = as_factor(FeatureCategory),

Feature = as_factor(Feature)) %>%

group_by(FeatureCategory) %>%

summarize(Mean = mean(Pi),

Median = median(Pi),

min = min(Pi),

max = max(Pi))

read_csv(file = "WoodyPolygonaceaePi.csv",

col_names = TRUE) %>%

# filter(FeatureCategory == "Spacer") %>%

mutate(FeatureCategory = as_factor(FeatureCategory),

Dock_Sorrel = as_factor(Dock_Sorrel),

Feature = as_factor(Feature)) %>%

group_by(Dock_Sorrel) %>%

summarize(Mean = mean(Pi),

Median = median(Pi),

min = min(Pi),

max = max(Pi))

```

```{r PiPlot}

################################################################################

#Generating the Pi Plot#########################################################

################################################################################

Data <- read_csv(file = "WoodyPolygonaceaePi.csv",

col_names = TRUE)

# Calculating the label positions

base_data <- Data %>%

group_by(CpRegion) %>%

summarize(start = min(Order),

end = max(Order)) %>%

rowwise() %>%

mutate(title = mean(c(start,

end)))

# Calculating the angle of each label

label_data <- Data

number_of_bar <- nrow(label_data)

angle <- 90 - 360 * ((label_data$Order - 0.5) / number_of_bar)

label_data$hjust <- ifelse(angle < -90,

1,

0)

label_data$angle <- ifelse(angle < -90,

angle + 180,

angle)

# Dashed line prep

LineSegments <- label_data %>%

na.omit()

# The Plot

Data %>%

ggplot() +

geom_bar(aes(x = as_factor(Data$Order),

y = Data$Pi,

fill = Data$FeatureCategory),

stat = "identity") +

ylim(-0.1,

0.25) +

# LSC Rectangle

geom_rect(xmin = 1,

xmax = 94,

ymin = -0.03,

ymax = -0.005,

fill = "gray95",

color = "black",

alpha = 1,

size = 0.75) +

# IR Rectangle

geom_rect(xmin = 95,

xmax = 122,

ymin = -0.03,

ymax = -0.005,

fill = "gray80",

color = "black",

alpha = 1,

size = 0.75) +

#SSC Rectangle

geom_rect(xmin = 123,

xmax = 136,

ymin = -0.03,

ymax = -0.005,

fill = "grey95",

color = "black",

alpha = 1,

size = 0.75) +

#IR Rectangle

geom_rect(xmin = 137,

xmax = 164,

ymin = -0.03,

ymax = -0.005,

fill = "grey80",

color = "black",

alpha = 1,

size = 0.75) +

#Feature Labels

geom_text(aes(x = Data$Order,

y = 0.04,

label = Data$Feature,

hjust = label_data$hjust),

fontface = "bold",

alpha = 1,

size = 1.4,

angle = label_data$angle,

inherit.aes = FALSE ) +

geom_text(x = 129.5,

y = -0.0185,

label = "SSC",

size = 2.5,

color = "black") +

geom_text(x = 108.5,

y = -0.0185,

label = "IR",

size = 2.5,

color = "black") +

geom_text(x = 150.5,

y = -0.0185,

label = "IR",

size = 2.5,

color = "black") +

geom_text(x = 47,

y = -0.0185,

label = "LSC",

size = 2.5,

color = "black") +

theme_void() +

coord_polar(start = 0) +

scale_fill_jco() +

labs(color = "Chloroplast Region",

fill = "Feature Type") +

theme(legend.direction = "horizontal",

legend.position = c(0.5, 0.25),

legend.box = "vertical",

legend.text = element_text(size = 10)) +

theme(strip.text.x = element_text(size = 15,

face = "bold"))

ggsave(filename = "PiPlot_Names.pdf",

plot = last_plot(),

device = "pdf",

width = 10,

height = 10,

units = "in",

dpi = 700,

bg = "white")

```
