# Supplementary material for: The complete annotated plastome sequences of six genera in the tropical woody Polygonaceae
Source: BMC Plant Biol. 2024 May 17;24:417. doi: 10.1186/s12870-024-05144-y (PMC11100190; doi:10.1186/s12870-024-05144-y)
Supplement: Supplementary file 3 — Supplementary Material 3. [file 12870_2024_5144_MOESM3_ESM.docx]

Supplement3: This is the infile alignment for the IQ-TREE analysis. The alignment is in PHYLIP format.

60 200044

RhePal -------------------------------------------------------------------------------------------------------------------------------------------------------------------------------------------------------------------------------------------------------------------------------------------------------------------------------------------------------------------------------------------------------------------------------------------------------------------------------------------------------------------------------------------------------------------------------------------------------------------------------------------------------------------------------------CGGGCGAACGACGGGAATTGAACCCGCGCATGGTGGATTCACAATCCACTGCCTTGATCCACTTGGCTACATCCGCCC-----CTAC-----GTT---------CCTTACT----TGAAACTTG---AAAATGAT-----TCAAAT--------TACACTCACTAACTCACTAT-TCATCA---TTTTTT--------T-----ACTTAATTTTTTATATTCA-----------------------------------------------------------------------------------------------------------------------------------------------------------------------------------------------------------------------------------------------------------------------------------------------------------------------------------------------------------------------------------------TTTTTTTCTTTTACAA-----------------------AAATTT--------------------------------------GAAATCTTTCTCTCTATGAATATAAATGACTATGAAAC-AAAAAATTTCCCCAA----AAACCAA-AACAACAAAGAAAGCGTATAGGGTAGAAATTAAAAAAATGCGAATGCGTCCATAAA------TATA-AAAAAATACAACATTCAATCATCACTCATC--------ACTTA-----------------------------AAACATTTTTTTC-------------TTTTTTTT---------A------------ATGGGGAAGAA-------AACTTATG--ACTCATAACATAAAATATATATATAATAAAA----------------------------------ATTTTAATTAAAGGAGCAATACCAACCCGCTCG-ATAGAACAAGAAATTGGGTATTGCTCCTTT------------AATTAAA-------AAAACTCGACTACACTAAG-----ACCAAAATCTTATCCATTGATAGATGGAGCTTGGATAGCAGCTAAGTCTAGAGGGAAGTTATGAGCATTACGTTCATGCATAACTTCCATACCAAGGTTAGCGCGGTTAATAATATCAGCCCAAGTATTAATTACACGACCTTGACTATCAACTACAGATTGGTTGAAATTGAAGCCGTTCAGATTAAACGCCATAGTACTAATACCTAAAGCAGTAAACCAGATACCTACTACAGGCCAAGCAGCTAAGAAGAAATGTAAAGAACGAGAATTGTTGAAACTAGCATATTGGAAGATTAATCGGCCAAAATAACCATGAGCGGCTACGATATTATAAGTTTCTTCCTCTTGACCAAATCTGTAACCTTCATTAGCAGATTCATTTTCTGTGGTTTCCCTGATCAAACTAGAGGTTACCAAAGAACCATGCATAGCACTGAATAGGGAGCCGCCAAATACACCAGCTACGCCTAACATGTGAAATGGGTGCATAAGGATGTTGTGCTCGGCCTGAAATACAATCATGAAGTTGAAAGTACCAGAGATTCCTAGAGGCATACCATCAGAAAAGCTTCCTTGACCAATTGGGTAGATCAAGAAAACAGCAGTAGCAGCCGCAACAGGAGCTGAATACGCAACAGCAATCCAAGGGCGCATACCCAGACGGAAACTAAGTTCCCACTCACGACCCATGTAACAAGCTACACCAAGTAAGAAGTGTAGAACAATTAGCTCATAAGGACCACCGTTGTATAACCATTCATCAACAGATGCAGCTTCCCATATTGGATAAAAGTGCAAACCGATAGCTGCAGAAGTAGGAATAATGGCACCAGAGATAATATTGTTTCCGTAAAGAAGAGATCCAGAAACAGGCTCACGAATACCATCAATATCTACTGGAGGAGCAGCAATGAAGGCTATAATAAATACAGAAGTTGCGGTCAATAAGGTAGGGATCATCAAAACGCCAAACCATCCAATGTAAAGACGGTTTTCAGTACTGGTTATCCAGTTACAAAAACGACCCCATAGGCTT------TCGCTTTCGCGTCTCTCTAAAATTGCAGTCATGGTCAAA-AATCTTGGTCTATTTCATTTAATCATCAGGGACTCCCAAGCGCACAAATTCTATA-------TAATCTATACTCGA-TTAAAAACTCGATTAAAAATAGATAATTATAATTGAAGG---CTTGTTATTCGACAGTATAACACGCCTTATATGCCCGTGTCAACC-----------AATATAAATCGAGGTCCATCAGAA--ATTTTTGTG---AATAAATGAATT------------------------------------------AAATTAATAAAAAAGTGTG-----------ATACACATATGACTT-------------CGATATTATAATT----AATAT-----GGGTTGCCCGGGGCTCGAACCCGGAACTAGTCGGATGGAGTAGATATTTTCTTTAT---------------------AAAAAAAAAATAAGATAAAAATCCCTCCCCAAGCCGTGCTTGCAGTTTTCATTGCACACAGCTTTCCCTATGTACACAT-----CTAAAACTCCGTTACTTTCCTAGATGG-AAAATTGAATACTCAGTTGATTCAAACCCTA-------CTACATGAACATTTCAGAATGGACATTGATGCA-TTTTTTTATTT------ATC-----------ATA----TTTTTTTACTAGAATTTCGATAATGAT-AATTCATTTAG-----ATTTAGT-TAGATAATTTCATAA--------------T----------AAATATCAATAATT----ATGACTGGCCAAATCATAAATACAAAAAATATCCAAATACCAAATGCGTACTCTATATAACCTTCGTGAAATAGAAGAATCTCTTGATAAGATCAAAGAAAGAACTTTTTCTTCCTTGATAAAAAATTCTTCCAAAAATTCTGAACCTAATCGTTTCAAAAAAGCACGTACGGTGCTTTTATGTTTACGAGCCAACGTTCTAGCACAAGAAAGTCGAAGTATATACTTTATCCGATACAAACTCCCCTTTTTTGAAGATCCGCTGTAATAATGAGAAAGATTTCTACATATACGCCCAAATCGGCGAATAATATCAGAATCGGATAAATCAGCCCAGGCTGGCTTACTAATGGGATGTCCTAATACGTTACAAAATTTCGCTTTAGCCAATAATCCAATTAGAGGCATAATTGGAACTAGGGTCTCAAACTCCTTAATACCATTATCTATTAAGAATGCATTTTCTAACGTTTGACTCCGTACCACGGACGGGTTTAGTCGTACACTTAAAAGAAAACCCATAAACTCCATGGGCCGATTTGATGATTGATTGATATAGATTC------------TTTGCAACCACAGGGAAAAATTATATTGCCAGAAATTGACAAAGTAATATTTCAATTTAGTCATCAGAAAAAATGCCCC------CCTTGAAGCCAGAATCCATTTTCCCTGATATCGAACATAATGCAGAAAAGGGTCCTTGAAGAACCACAGGATAACCCCAGAATGCTTAGTAAATACT------TTTACAAAATGTTCTAACTTTAGGTAGAAATAAACTCGTGCAAGAAAGGCTCCGTAAGATGTTGATGGTAAATGAGAGGATTGATTGCGGAGAAAAACGAAGATGGATTCGCATTCACACACGTAGGAATTATATAGGAACAATAAGAATCTTTGA---TTTTTTTTTTTTGAAAAATCGGAAACAGATTTCTTT------AGAGTAATAACACTATTA------CAATACTCATAAAGAAAGAATCGTAATAAATGCAAACAAGAGGTATCTTTTACCCAGTAACGAATAGTTTGAACCAAGATTTCCAGATGGACAGGGTGAGGTATCAATATATCTAACACATAATTTAAACGTAAAAATTTGTCCTCTAAAAAAGGAAATATTGAATGAATTGATCGTAAATTTTGATATTTTTTT---AGTCCTTTTCCGTCTAGGGAAGATATTAATCGCATAGAAAATGGAATTTCCGTAATAGTTGCAAATCCCTCTGAGATCTGTTGAGAATACAAATTTTTCTTGGGCACAAGAAATTC---GGTTTTGTTAGAATCATTAACAGAAAGAATAAAAAAATTCTGTTGATACATTCGAATAACTAAACGTTTTATAACTAGTAAACTGTATTTTGTGTCAGAACTTTTT---------TTTTTATTTGACAAC---AAAATGGATCTGTTTAAACCTAAATCCTGATCATGTACAAGTGCATAAATATATTCCTGAAAGATAAGGGGGTATAAAAAATCATCGTGCCAAGATCTAGCTAATTCTAAATATCCTTGGAATTCCTCCAT--------------------------CGACCTGAAACGAAAAGAAAAGTAGAGGGTTTCTTGGGTTAT-------AAAATG----ATAC-ATAGTGCGATACAGTCAAAACAAGGTATTCTAG------TACAAAAGAATAG----------------ATCCCTCGGAAACAGGTAAA-CTCATCAAC--GGACTCTCTATCTT---TTTTCCAT-------CTAATTGGTTTC-------GTTC---------------CTATATAAT--TATAGGAT-------------------AAGAAGATGGTTAGAAATCCTTTATTTTTTCAACTCAATCGCTCTTTTGATTTTGG--AA---AAAAAGTATCCTTATCAATATACTGTTTCTTCTACACATTCATCTCCA-TTTTCCA------T--TAGAAAA----------GAAAA----TGGCT----A---ATAGTTAGGATTCACT---AAAAAATCGGTAATCCACTCCTGGAAAAG--CCGCTCCGCATCAGGCACTAATCTATTTTTAACGTTTAA-----TTA--GGGCGGGTAATCATTCCAATTAAGAACATAAGCTCGTTTCTTTTTCTTTCCCTACAATTAG----AGCCA-TAAGGCTCGATCCATGTATTCAAT-CGACCCAACTTTGAA-----TTCAT-TTCGTTCTAAGAATTCAACCAA-AGGTTTTGTACCG------------------------ATCTAATAAGAACGAAATTGTTTCATAATTCTCCATTGATACGACATGCTCTTTTTTCCATTCATTCC-TTTCAGGATCAGTCGTGGTCTTACAAACTCTACCGATAGTGTGGACGAATCCCTTGCTTCATCCAAATGTGTAAAAGAACCTAGCCGCACTTAAAAGCCGAGTACTCTACCGTTGAGTTAGCAACCC----AAAG-----AGCATATA----GTATATAGATACAATCGAGATC-----A----------------------------AAATAAAGAAATTAGACA--------AGACAACCAAAAATTTGAATTAG-AA-------------------------------------------------------------------------------------------------------------------------A----AAAAA------------------------C-TAGA------------CCACTTATTAGATATTTTCATCCACTAC---------------------------------------------------------------------------------A-TTATATATA-------------------------------------TTTTCTATTTAATTTCTCTAT-----CTTTCTATCTCT---ATATTTTTTAAG--------AGATTC--TTTTT----------------------------------------------------TCAAT-----------------TATC-------------------------------------TATCCATTTCC-----TTAT-AAAAATTTGGGTTTTGTATCACAACAAATTCAACGAATC------TTT------------------GAATAAAGTAAAAACCAAAACCTG---------TTTTTTGTATTTAGGAA---------------------------AAAAAAATAG--AAAAAAATGGATCCATTTACAC------------------------------------------------------------------------------------------------------------TTGAATTATATTTGTTCACTACACTC----------------------TTG------TCAATA-TGTATGTT-A----------------------------------------------------AAAAAAAAAA-------------------------------------------------------------------------------------------------------------ACAC----------------------------------------------------------------------------------------------------------------------------------------------------------------------------------------------------------------------------------------------------------------------------------------------------------------------------------------------------------------------------------------------------------------------------------------------------------------------------------------------------------------------------------------------------------------------------------------------------------------------------------------------------------------------------------------------------------------------------------------------------------------------------------------------------AAAAATAAAT-----------AAAGAACTTGTGTTGGATTGGCAC--------TATCTAAATAAG----------------------GTA-CATGATTAGAAAGGAAAGAATGTAGAGCGAAAT---------ACAAAAGAAGTAGACAAAAT--------------CTCAATAA---TTATACGTC-----AAATAATT--------------------------CATTC-------------------TTTTTTGAGTATAGTTCCAAAGAAAA-CCATCCAA----TTGAAAGGAATGTCAGAATTTTCGA---------TTGCTAGATCCAACTCCTAC------TGTTAGTGAGTTGG------TC-----AATAT----AAAACTTTCTTCGTTTGTTCACTTGAT-CTTTTTTCTAGAC-------------ATCT------------------T------------AAAATTTTT-------ATTTTGATCATTCATTAGAGGAGACACAGCCTCC-----------------------------------TACTGGAACAATACCAAATAGG-----------------------TCTGACTAGAGCAAA-AGAAGGTGTA-------AATAATAAAGA---------------------------AAGGGTTA----------------TATAAAAC----TATATACA-TATGAATCGCTCA------------------------------------------------------------------------------------------------------------------AGTCCCTTTGTTGTTGTACTTGTTGC-------------------AGTTAATTAAGTGAA----TTTTG------TT-----------TCATTAGGGCGAAGTTCTTTAAAAACCTCTGCTTTCTTTAAAATATTATAAACAGTTCCAGTAGGTTGAGCACCCCTTTCAAGAAAATATAGAATAGCGGGAACATTTAAATAAGTTTTATTCTTTATTGGATCATAAAAACCAACTTTCCGAAGATCTCTTCCTTCTCTTCGGGACCGAACATCAATTGCAACAATTCGATAGACAGCTCATTGGGATAGAT----TATATGAACAATA---CC-CCCCCTAGAAACGTATAAGAAGTTTTCTCCTCGTACGGCTCAAGAAAAATGATTTCTTC---------------------------------------------------------------------TTTTTTT-----------------------------TTCAAGTTATG---------------AATATATAAACTTC---------------TAATGGCAAATAG--------------------------------------------ATCC-------ATAAAACCATCAAATTAATTAGACTAAGAATTAAGC------CCCATTTTGCTCT------TATTCTTCTTT---------CCGGAAAAACCCATTTGCACCCATAACTCAAGTTGAATAACT-----CTCAAATAACTCAAAAGAAAAATTTA------ATTTAT-----ATTTATTGAGTGGTCTCTAAC--CCCCC-----CTTTGTCTGGC-----TTATTTAACCTC-------TATTGGAATTCTTCATTCT------AAT-TTAGTTGTTGATACAAT-----TGAGAATGAAAAGGGCCTTTCCTTGTTTCGGAATC-----TCTTTGCTTTAAATCATTAGGTTTATACATTACTTCGTTGATCTTTAATCCTTTCAAAATGGCAGCAACATACCCCTTTTGTGATTGTTTATC-AAAGAAAAGAATCATACAAACACTTGCGCTTGATTCTTTCACAATATACT------TTGTTAT------CGAAAAG------GATTTATCAAT----------TCCAACAAATTTTCCT-----TTTGGATTGGAAACTTGGT----GGGGTTGGATCCTTTCGATTTATCTGTCACA-----AATATACTTACGAAGTTGTTCTAATTTATTGATTCACACTAACCCTAGATTCTTGCTCTTAAGAAATGAATCAA-------TACTTTCTACTCGAGCTCCATCATA-------TACTAG-TTT-----AAATTAACTATAA------CACA--AA-AAAAAGTGTAGGTACTAGTCTAACAGAACAGGGGATGTCGAGCCAAGAGCACCTTTTTCCATAT----------AAAATGGTGGATAT--AAAAATCCACATCAGATCATGTCCTTCAAGTCGCGCGTTGCTTTCTACCACATCGTTTCAAACGAAGTTTTACCATAA----CATTCCTCAAATTTTGAACCGGTATGCAATTGATTCAATTATGGAATCATGAATAGTCATTGGTTTAGG-CGGTACGTACATAGAA-ATCTATACTT------------------------------------------TATTC---------------------------TCTATTAAATAGA-----------------------------------TATTCTAGAT----------------------------------------------TCAATATATAATATTCTAT----------------------------------------------------------------------------TATTGGC---------------------------------------------ATTAAAGAAT--------------AT---ACGGATATTCACCCTTTTCTTATCTTATATTTAATATTTTTTAATATAAAATTATATCGA-----------ATTTTAT---------TTTACTAGATTTTCGATATCT-----------------------------------------ATTTTATATTGAT---------------------TGATAGTAGTA-----------------------------------------------------------------------------------------------------------------------TTATGGGATTCGAAAGATA-----------AATTTGA----------------AATAG---AAAGTTATATATAA-----------------------------------ATATAAGATAAACTAAG-TAAATGAATAAGTGT------AAAAAAATTCC----AAAAA---TGATGTTGAATTA------GGAA----------------------------------------------------------------TATT----------------TTT---------TACATTTAC-------------AAG-------------------AAAAATAAAAAAGAAATACA------------------------------GCTTTTTC-TAGAACTCAG-TATT-------AATG------ATTT------------AAATAAACCC----GA-TAGGTCTA---TCGAA----------------------ATGATAACTTGTAAAAAC------------AAATCTGA----TTTTTTTCACAA---------------AAACAAAAATCGTAAACCCTT-------CTT----------------------------------------------------------ACTGAGATCAAAG----------------------------GTTAT--------------ATAAA---TAAAGTAAGAATCT-------------------TTC---CTCATTTTATACGTT--------ACGTATTTCTTTT-------------GGGGTTCAATAAATTTTC--------------------------AATTAAGGTGAATAAAATGGCTAA--------ATGAATCGCCTTTCCTTTCAAC-------CATTAC-----ATGGATTTC---------TACTTATTC--GTTCGTTATGT-TCGT--------TATAAAATAAAGAACAAAA-----------------------------------------------TACGAATTATCAAAACA-----TTAAGTTT------------------------------------------------------------------------------------CAAACTA----------------------CATATGTACCTTA--------------ATGTTTTGATAATTC----GATTCGAA----------ATGAGATTCGGGTAGATATTTAAATTGACACC-----T---------TTTCTTG-TTGATTAACTCTTATCTACCCCCTCGCCCAA-TTATCTATAGGTACCGGGGGTCATAACCCCC----CC----AAAAAAAAGCACCCTTTTTTATTTAC----------AAA-----------ATCATAAACT------GTCTAGGTACAAAACGAAACAAAACAGACC--------------------------------------------------------------------------------------------------------------------------------------------------------------------------------------------------------------T------ATACACAATACGGCA----------AATTTCTAATTTCGCCGCCTCCTTTA------------------------AGGGATAGAGAATATAGA---ATTGCTTTCGCA-------TTTTTAGTATG------AATACATC--------------TTTGATAATTCT-----------------ATTAAT--------ATTAACATAAC---------------TATTATTTTTTATATTTAT---------ATTTTTTTTAT--------ATAGAA--------------ATAGACACACGGCATAAGTAACTAAAT----TCCTTCCATATGGA--------------------------------------TTTTC-------ATTGAAATTTAAAGAATCA----ATCTATTATCCTATCTTGCCTGTATTAGATCACA---------ATTGGATTCAGTTAGATC--TGTGTGTGTC---------AATTCC-----------GTTTGTAAAAAAGATA-----GAATTCAGAAACGAATCTTT-----------------------------------------------------------------AAAT-AAA-----AAAAGCGAAAGAAGAA-AAA---------ATTATCTAGA-TACTCTATCTATAATACTCTAT---------------------ATAATATAAGACTACAC-TTTTTT------------TACAAAAAGCCCCTTGGTC------AAAAAA-------TTTTTGGATAGAATCCATATGCTCTGGGACGGAAGGATTCGAACCTCCGAATAGCGGGACCAAAACCCGTTGCCTTACCACTTGGCCACGCCCCACTTAGATTT----------------CTACTCTACACTAA----------TCTTGTTATTGATTGTTCGTCAATTCCAGCCAAAATC-TCTATAGAATCCAGTCGATTGTTATTTTGATTTTAACAC--AT-ATA---------GGTATAGAATTAAACAAACTGAATTTCTTGATCATTACATAT--------------------AATTTAATTAAGATAATGTATGAAAGTATGATTTCTTCTATTCTCT-TTTTATT------TGAGAATGGAAGAGTTTTTGATTGAGTAAGTTC--AAA--AAAAAAA-AGAAACGAAAGGATTTTTTATCGACTTTACTTTTTTTAATTTTCGCTTATCTTATATCAATAACTCAATCAAAATGCAATA------ATCTTC--AAT-AAAAAAGATGTCTGCTATGCTTAATATCTTTAGTTTGATCTGTATTTGTCTTAATTCTACCCTTTCTTCGAGTAGTTTTTTATTCGCCAAATTGCCCGAAGCCTATGCATTTTTGAGTCCAATCGTCGATTTTATGCCAGTCATACCTCTATTCTTTTTTCTACTAGCCTTTGTTTGGCAAGCTGCTGTAAGTTTTCGATGAGAT------TTAAAATATCGTCCT----------AGAA-----AACGATTTATTCGAG-----AAAAATTTCAA---------ATA-----TG--------------------GTTATATT----------AATAAATGAAAAGATCAGATAC--G-TT-----TTATAGTATGAACTCT-CAATTC-------AAATTTCAAATAGA-AAAAGTCTTGGATAGAATAGCCTAGAAAAATGGGAATCACTTCCTTT-ATCGTTCTAACAAAAAT--TTCCGTGAAAGGCCC-------TGTGAGGTCTTCCACAA----AAATTGTGGGT----CGGAAAGCGATTGGTTATT---------------ATGGAGGCT-----CCTAACACTTAACAAATAAA------------------------AT---TTTTCTTTT------------------------------------------------------------------------------ATAG----AATATAT-----GGGGGAATCTATTCTCTTTTTT----------------ACACAAAAAGATCTTGGAGATTGTGTAATGCTTACTCTCAAACTCTTCGTTTACACAGTAGTGATATTCTTTGTTTCTCTCTTCATTTTCGGATTTCTATCTAATGACCCAGGACGTAATCCTGGACGTGAAGAATA----AAAGAGAAGTTTCCTTACTTT----------TTTTTAGTGTCTTA-------TT-----TT----------TTTT---------AT-------AAAAAAATAAAAAGAATTC-----AAGAAATTGGAAAGAG-------------------AAAAAATAGTAAATCATCAACAGAAACGGAAAGAGAGGGATTCGAACCCTCGGTACGAATGACTCGTACAGCGGATTAGCAATCCGACGCTTTCGTCCACTCAGCCATCTCTC----CCTAT-------------------------TGAAAAAAGTAA---------------TTACAAAAAAGAATTACTA--------ATTACTATAGTTAG-------ATTACACATAACGT--------------GCCATTTTAAAAATAT--------------------------------------TTTTTTTCTAGGTT-------------TTCAATATAAAAAA-----------------------------AATATA-ATA---------------------------TATAAAATTTTAATTCGAAA-TTCTTTAAGATTCCAGACTCT--------------------------TCTAAATTCTAGAGAAT---------------------------------------------------------------------------------AATTAAACT------GAAATA-TAAGTCAAATTTT---------TAAAGTTATTTA--TATTA----------------------------------------------------------------------------------------------------------------------------------------------------------------------TTTATTATTATTTT-------TTAT--------------------------------------------------------------------------------------------------------------------------------------------TTATTCTGTT-------------------------------------------------------------------------------------------------TATA-----------------ATCTAAAT----------------------------AGAAATTTAT------------------------------------------ATATATAATAT-----------------------------AT-ATATAATAAG-TCTGAT----------ATATATATAAACAT--------------AATATAGAAAAAA------------------TATGTATACAAACATA--------TTATG--------------------------------------TATACAAAG------------------------------------------------AGATATAGC--------------ATTTATAAT---------ATATATATAATATAT-----------------------------------------------------------------------------------------AAATACATATAG---------------------------------------------------------------------------------AAATAACTAGAATAAATA--------------------------------TAAGTTAAACAAAA------------------AAATATAAATAGATACAAGATAGACT---------ACAAGGTTTATCCGAAAGTCCAACT------------------------------------------------------CAACTAAGGATTCAAAAAAAAA----AGTTCAATACAT---------------------ATAAATAAAACCAGAAAGA--------CTTCTT-------------GTGAAAGGC---CTTTTAGTCC----CACGGCCTGGCCTGGTCACTACCTCGCCGGGCC----TTTTAT---------------TAATTCAACGGATCATAGCATAGATAGAAAAATTTTT--AGTT----CA---TTTTTTTATAACTGT-TTTTTTA---------------TTTTATAACTCCAT-----------------TAATACTTT------------AATACT-------------------------------------------------------------------------------------------------------------------------------------------------------------------------------------------------------------------------------------------------------------------------------------------------------------------------------------------ATAG-AAAAAATGCTT----------------------------GTTAAAGCAAGAAC----AAAAAAAGGCATGTTTCTA----T----------------------TCGATATAGAATAAAAATGCC---------CTATTGATTATCCTTT-----------------------------ATTTTT----------------TTTAAGAAA-----------------ACCTATAGA----TTCTTGCACAACTCG----TCTAT---TATAACTTTAGCTATTTTGTTAAAAC---------------------------------------------------------------------------------------------------GTATCCGTCAAAA-CT-CTCTGCC----------CAAAAATAGAACTTCGTGCTTAGGT-ATTTAAATCCCGT------TTCTGAATCTCCTCC--------GA--AAATAGTTCAATACTCTCA------------------TTTTTCATGATTATTTTATCA----------TCTTTTCTTGATTACGTTAAATTTCGTTATTCGACAAAAGTTCCATTTCGATACAATAATCGCATTGTAGCGGGTATAGTTTAGTGGTAAAAGTGCGATTCGTTCT----------TTAATAGTTAAGGGATCCTTCGATTT-----GATTCCTAATCCGAT-AAAAAACTCTATTTC----ATAA-AAGGAATTAACCCTTTCCCTCTCAATGACAAATTTGAGG-AGAATGTTAAATTATCGTAATTTG------TATC-TAAG-GG-----------------------------------TAA--ATTATT--------AATTG------AAATTTTGGATTATGAAATTACGAAACATAATT-GATTTTGAATTGGATCAATACTGCCAATTGAATAAGTATGAGTAAAGAATCCATGGGTGAAGATAGAAATATAAATTTTTAATCGTAACTAAATCTTCAA---------TTTTTTCTTTATA-GAAAGGACATTGAAGC----AAAAAAATAGCTAAAAAACGACGACTTTAGTTTACTAAAGACATCGATAGGCA-----TATTCTTTTAGCTCGGTAGAAACAAAATGTT-TCTCCTCAAGATTCTCTAAAATAGAAATAGAGAACGAAGTAACTAGAA----TGTTAGAATACTCATCTTCTAG--------AGGGATCATCTAGAAAGCAAGTACTT--------------TTGAATGC-----C-TTCAGGC-AAAAGCTGACATAGATGTTATGGGTTAA--TTT--------TGTTGTTACCACTCTGGATTTCGATCTGGCAATTTC-----------TCCATCTTCCATAAAGGAGCCGAATGAAATCAAAGTTTCATGTTCGGTTTTGAATTAGAGACGTT--AAA-AAAAA-ATAGACGTCGACTATAACCCCTAGCCTTCCAAGCTAACGATGCGGGTTCGATTCCCGCTACCCG--CTCC-----ATTTTTT-----TTATTTAATGC---------ATTCAAAAATGCATTAATTA---------------------------------ATTAATTTAAGT----------------------------------------------------A----------AAGAAAAGTCGTAATGA--------GAAGCGTCCATTGTCTAATGGATAGGACAGAGGTCTTCTAAACCTTTGGTATAGGTTCAAATCCTATTGGACGCAATT-TATTTCCATA-----TG-TTTTTT---------TCGATATCTATGGGA----------TTTTTTTCTATTCT----TT------TATATTC------TAAAAATAAGG----------AATTTGAATCAGAAATTTTTATTTTAGTTTCTTTTTTTA-----ATTTTT--TTTATT--------------------------------------------------------------------------------------------------------------------------------------------------------------------------------------------------------------------------------------------------------------------------------------------------------------------------------------------------------------------------------------------------------------------------------------------------------------TA----------------------------------------------------------------------AATAAA------------------------------------------------------------AAATATAATAA----------------------------------------------------------------------------------------------------------------------------------------------------------------------------------------------TAAATATAATTC------------TTTGATTATT------------------------------------------------------AAGCTAAGA------AGGCTCAATTTC------------------------TTTATGCCTATTCCTGAAGTAGAAAACGTTCCATCTGTTCTTGAATACCTTCTTTTAAAAGGGCTTCCGCTTCCTCAGTGAATGTCTTGGTAGAAGATATAATTTCTTGGAACTGTGGTTTATTCGTTTTGAAGTACTTACGTAAGTCATCAAGAAATGGACTTACCTGTCCAATTTCTAATGAATCAAGATAACCATTCGTTCCAGTATAAATAGTCATTATCTGCTCTTCTACCGCCAGGGGGGAAGCTTGGGATTGTTTGAGCAATTCGCGTAATCGTTGGCCTCTTGCCAATTGATTCTGAGTAACTTTATCGAGATCAGAAGCAAATTGTGCAAAGGCTTCTAATTCTGTGAATTGCGCCAGTTCCAATTTTAATTTGCCGGCCACCTGTTTCATGGCTTTAATTTGAGCTGCAGATCCCACTCTGGAAACAGAAATACCCACATTAATGGCGGGTCTGATTCCAGAATTGAATAAATCAGCGGATAAGAATATTTGTCCATCTGTAATAGAAATTACATTAGTAGGAATATAAGCCGAAACGTCTCCTGATTGGGTCTCAACTATCGGTAAAGCGGTCATACTTCCTTCGCCTAAACGTGAACTTGATTTAGCGGCTCTTTCCAAAAGGCGTGAATGCAAATAAAAAACATCTCCTGGATAAGCTTCCCGACCCGGCGGTCTTCGTAATAGAAGAGACATTTGTCGATAAGCCTGTGCTTGTTTGGAAAGATCATCATAAATTATTAAAGTGTGCCGCTCACGGTACATAAAATATTCGGCCAGTGCCGCTCCTGTATAAGGGGCAAGATATTGTAATGCAGCTGGAGAATCTGCAGTTTCAGCTACCACAATAGTATATTCCATCACTCCCCTTTCTTGGAAAGTAGTGACTACCTGCGCTACCGAAGATGCTTTTTGCCCAATAGCTACATAAACACATATTACATTTTGTCCTTTTTGATTAAGAATAGTATCCGTAGCTACTGCTGTTTTACCAGTCTGTCTGTCTCCAATAATTAATTCTCGCTGACCGCGACCTATAGGGATCATCGAATCAATAGCAATAAGCCCCGTTTGAAGAGGCTCATATACGGAACGTCTCGAAATAATACCAGGAGCGGGAGATTCAATTAAGCGAAATTCCGAAGCTGAAATTTCGCCTCTACCATCAATAGGTTTAGCCAGGGCATTTATAACACGACCCAAATAAGCCTCACTGACAGGTATCTGAGCAATTCTTCCTGTTGCTTTTACAGAACTTCCTTCTTGTATCATTAAACCGTCACCCATTAATACAACACCGACATTATTTGATTCCAAATTCAGAGCAATGCCTATTGTACCCTCTTCAAATTTGACTAATTCGCCTGCCATTACTTCATCAAGACCGTGAATACGAGCAATGCCATCGCCCACTTGAAGTACGGTACCCGTATTTACAATCTTGACTTCTCTATTATATTGTTCAATACGTTCGCGAATAATATTATAAATTTCATCAGCTC---GAATGGTTGTCATGAGTCTTTCTTAAATTAA----ATGAATTATTTTTTGGAAA-C--AAAA-AAAATAATA-------CCTTAACCACAGTAGAAGGCCTAATCGGTTATTTCTTTCATTGCGCCAAACATGCCAATATTAGCATTGATGGTACGTAAATGTAACTCGTTGCTCAAACAACTATTCAGAGTTCCTAGAGCTCCTTGTAGGGCTTGTTGAAAAACCCGCTGGCGTACTTGATTAATCGCCCTTTGTTGTTCAAAATGAATGGTTTCGTTTTTGTAATTTTCTAATTGTTCTAAAGTTTTATAAGTTGAATTAATCAAATTCAATTTATCTCGTTCTATTTCAGAATATCCATTCGCTCGAAACTGATCTGCTTCCATTTCCACTTTCCGTAAGCGAGCCCGGGCTTTTTCTAGCTGTTCAACGGCCCTTTCGCGCAGTTCTTCTGAATTTCGAATAGTATTCACGATTCGCAGTTTTCGATTATCTAATAAATCACTTAATGAAAGTAGATTATCTTTCCATTCATTTCAAAACTTTCATGATCCCTTCCCGAACCAAACTTGAATCTTTCGATTCATTTGGCTCTCACGCTCAATTACTTCCA---------TT---TTTTATGGCCAATTTCCA-----TATCTTT-----TTTTA--------ATGTAATGAACCTATCCTCTACTCT----TTTTGTTCAT-----------ATTCGAACAAAATTG--GAAATGAATCAATAATCCAAGGCCGGAATA-----TTTGGAGGACTCTTCTGACCAAAC--AAAAAATATGTAATTGTCAGCAAAGTTG------TTTTTTTTTCAAATC----CAAAATTTCTTATTTT--ATATTTTTTT--------------AAAAAATAGGTCATCAACTCAGCATTTGGT-----ATTTAAACAAAAATGTAAA--AAAAAAAAAAGAAAAAAGGATGAA-----CCCCTTTCCAATACCAATAAAGGTTTCGAATC-TTTTTATCGATATGAGTGTTATATATCGATAAATTTCTAAC-----TATTCCTTGGAAATGGAAAACCATTTC--------------AGTATTAATATAGTGGTAGAAAGAGTACCATGCTGTGGCTGAACTT----CAAACAGTTTAGCTTTAACC-----ATGTTAAT--------AGATCCACATTATTGGTTGCTAGAGAATCAAAGTATATTTACCAACGAATCACGAAATGCTATGGTTCTTAC-------ATATGAT---------------TATATGA----TTTCTTAA-------TTTATTCAGAAGTAATTCGCGAGATCATGCACCTTT-----CTTTACTAGTTATACCGA-------------AAAGGGGCGCAGCTGG----TTGAATCCAGTCTATTCTTGAAATAAACAACTCGCACACACTCCCTTTCCAAAAAAGATCAATACACCAATCACTACACTGAGATTTATTGGATTTGTTGCTAAAATATCGGTATTAAATCCGAAACTCCCGGCAGATGGCCAATGACCCGGGGAAACGAAAGAATCGGTTACATTTTTCATATGATCTCC--------TCTTA------------------------------------TAGATA---GACT-CAAAAATCGAACATCATTTTTTG-----------------------------------------------------TTGTATTACTTGACCTATTTCC------------------TATTTAGAA---------ATTGAAAATAGATTCA-AAAT----------------------CGATTCCATT----TCACAATGTA-----------T--TTTCTTTTTC--AATTGAGAT-----T----------TCCAATAAGAATAAGACTTATTCGAA--TAGGATTAGGGACCGGG----CGGGTTTT----------------------CGTGTAAATTGCGAAATACCTCGTTT------GTTGCACT----ATTTCCTAA-----AC---AGCTTTCGTTGG-----ACTAAGAA-GGGGAAGGAAGAAAGCGAATCGGTAACACTAATT-CCTCATCCTCAAATCAGCCCT----T-------CCCCCC---GGGTTTTCTCA-----------AAG-----TAATTGTAGGAG-------CGAAATCTTGGTATAATGCGAAAAGGCAAGCAGGCAAGCGTCAAGTCCAAAG----A-AAAAAATACGTA--------TTTTTTTTTATTAGAATTAAACTTA-----AACAAAAGGATTCGCAAATAAAAGAGCTAACGCTACAACCAATCCATAAATTGTTAAAGCTTCCATAAAAGCCAAACTAAGCAATAAAGTACCTCGTATTTTACCTTCTGCTTCGGGCTGTCTCGCAATACCTTCTACAGCTTGGCCTGCAGCAGTACCTTGACCAACTCCTGGTCCAATAGAAGCAAGCCCTACAGCCAACCCAGCAGCAATAACGGAAGCGGCAGAAATAAGTGGATTCATGA-----TAAGTTCCTCACAC--AAAAAAAAGAAATGGTTAATGATACAATCAACGAAAAAATTATTAC--TT-AATTATTCCATCGACTAAGATTCAGCCAGTCGAAGTCAGTAAGAACTCCAAA-------TTAAAATAAAAA--------------------TATTCCATCAGATCATCAGAA-----T-----TCTCCTTGTT--------------AGTTCCTATTTGTTGAGTCTTTCCTGAATC-TATACAACTTGAGTTTCTCCTTTC-C--------TTC-----TTTCTAACCATT-CGTTGAATTCTTCGACCCTTTCTTGC--------------TTTATTTTATTT----GTTTATTCATTCAATT---CATAATC--------ATGAATAAATG--AAAAAACAT---------------AAAAAAAAGACTTCTCTTAATATCCCC-------------------AT----------------------------CTAAATGAAA----------------------------------------------------------------------------GTAGGGC----TTAATATTAG--------------TTCAGATATAACGA---------------GTCA------ATATCTAATATCCAATATACATGTCTTTCTTCCATAACGTAAACCCGGCATTCTACCTTAA-----ATTCAATTGGATTCTAGAATCTCT------------CTTTGAATTGAAATATCTACAGGAGTTGACTTATAACCATTCGATTCTATATGCCTAGTTAGA--------------------------------------------------------------------TCTTTCTATACTA-----------------------------------------------------------------------------------------------------------------------------------------------ACCAA-----------------------------CCCTCT----------------------------------------------------------------------------------------------------------------------------------------------------------------------------------------------------------------CTCTAGTATCTC-------------TTTTCTATTACTAT---------AGAA-CATACTT------------GTATGTTCCC-----TAAGTAGATTCTTTTGAAA----CATATAGT--------------------GACTT-----------------AAAAAAGACTCTTTCGAA--------AATGAATTAATGATGACCCTCCATGGATTCGCCTATATAAGCTGCGGCTAAAGTTGCAAAAATAAGAGCCTGAATACCGCTTGTAAATAATCCAAGAAACATGACAGGTATAGGAACTACTAAAGGTACTAAAGAAACAAGAACAACAACGACTAATTCATCGGCTAATATATTTCCGAAAAGTCGAAAACTAAGGGATAGAGGTTTTGTGAAATCTTCTAAGATGTTAATGGGTAACAGAATTGGAGTTGGTTGAATGTATTTACTAAAATAACCCAACCCTTTTTTTGTAAGACCCGCATAGAAATATGCCACTGACGTGAGTAAAGCTAAAGCTACAGTCGTATTTATATCATTCGTAGGTGCGGCTAATTCCCCATGAGGTAACTGTAAAATTTTCCAAGGTAAAAGAGCCCCTGACCAATTAGAAACAAAAATAAATAGAAACATAGTCCCAATAAAGGGAACCCAAGGACGATATTCTTCTCCAATCTGAGTTTTGCTCACGTCTCGAATGAATTCAAGGACATATTCAAAGAAATTCTGACCGTCAGTCGGAATTGTTTGTGGATTACGAGCAGCTATGGCGGCTGAACTTAATAAGATAGCAATTACAACCCAAGAAGTAATAAGTACTTGGCCGTGGACTTGGAAACCACCTATTTGCCAATAGAAATGTTGGCCGACTTCCACACCGGATATATCATATAATCCCTTTAGTGTATTGATTGAAC---ATGATAGAACATTCATATTGTCCTCTGACAGAAATATAACC-TT--CAA-AAAAATATTATTTTGATTCAAC-------CATTGCTTTCTCGCCTTG-----TCTACTTC-AATCGT-----ATATAATACCAACTAATCA-ATCATATCCCCAGTTATTTTT--ATATC-TTTTTTATATT-CAG-----------GAA---TCCTAACCGATT----------CTACCCTAT------TAATTCGAG-----AAA-------TCA----------ATTATAGAGTTCAC-TAAAGTCA-----TTTT-------------TTTATTATGAAT---------CAAGGATTTCTT---------ATATAGCTAGAACGACCTTCACAAATTGCGAATACTAATTTGGTGAGAATTAGTCGGATTGAGGCTATAGCGTCATCGTTTGCCGGAATCGAAATATCTGCAAGATCGGGGTCACAATTTGTATCGATTAAACAAATCGTTGGAATTCCCAAAGTAATACATTCTCGAAGGGCTGTATATTCTTCTTGCTGATCAACGATGATTACAATATCCGGCAACCCTGTCATATATTTAATCCCACCCAGATATGTTTGCAAGTGAGATAATTGTCTCTTCAACATGGCTGCATCTCTCTTCGGAAGACAGGCCAGTCTTCCCGCCTTTTGTTCCATTCTCAAGTCTCTGAACTTATGAAGTCTCGTTTCTGTGGTGGACCAATTCGTTAACATACCCCCAAGCCATTTTTTATTAACATAATGACACCGAGCCCTTATTGCAGCCCGTGCTACTGAATCAGCTGCTTTATTTTTTGTCCCAACAATTAAAAATTGTTTTCCTTTACTTGCTGCATCAAAAACTAAATCACAAGCTTCTGATAAAAAACGAGCAGTTCTAGTAAGATTTGTAATATGAATACCTTTACGCTTTGCAGAGATATAAGGTGACATTCTAGGATTCCATTTCCTAGTACCATGGCCAAAATGAACTCCCGCTTCCATCATCTCTTCCAAATTGATGTTCCAATATCTTCTTGTCATTTCTCCTCACATTTTCTC----------TTTTTT---------TAAGAGATGAGGTATCTCGAA-----ATAAATAATTGTTCCGAAGGAACCTTCTCTTCGGCAGCTAATTGGCCATTG----------------------------------------------------------------------------------------------------------------------------------------------------------------------------------------------------------------------------------------------------------------------------------------------------------------------------------------------------------------------------------------------------------------------------------------------------ATACACAATCCAAACCATTAATTCCTTTCTATTCCTTATTATCTTT------TATCTTT----------------------------AAAAAA-----------------------------------------AAAAGAAAATGCCCGTAAGAAATACAG------AACAGAG-----AAATAGGAGGAA-------TCCGTTCTTAAATT-------------------AACTAAA-CTAGGGTTT----TGATGTATGAT----------------TTTT-------TTTTAAACCCAAGA-------ATTAAAAAATTCTCTGTGGTAAAACAAAATATCGTTCATTTCCCCCTCGAATAGATTC---TTCT------TTTTGTTTTTCAAAGGAATGCTCTTATATTGGCTTGAACGATGTACTAATCCTTTGAATCCGGTACCGACGGGTATCATTCCGCCCAGAACCACGTTTTCTTTTAGACCTTTCAACCAATCGATACGGCCCCGGAGAGCAGCTTTTGCTAAAACTCGAGCAGTTTCTTGAAAACTCGCTTCGGATATAAAACTTTGAGTATTCAAAGAAGCTCTCGTTATTCCCAATAAGACGGCTCGGTAAGAGACCGCTTCTTCCAAAGCACGCCCTGTTCGTTCCGCTCGCAACAAGCCGATTAGCTCTCCTGGTAAAAAAACATTAGACATTCCATCTTCTGAAACCAAGACTTTTGATGTTATTTGACGTACAATAATTTCTATATGCCTATTATGTATCTGTACCCCTTGGGATCGATAAACCTTTTGGATCTTATTAACCAAAGAGATACGACTTTGCACTATAGTTAGCTCGGCGCCAATCAAGAATCCCCAAGGAAGTCCAAGAATTCCTGCTATACGTTCATTCCAAGCATCAATCCTCCTTTCTAGATTCATCGATATTGACTCAAGCGAACGAACTTCTAAGACTTGTTCCACCTTTGGAAGGCCTTGCGTTATATCACCAGACCTCGATTTTTCATATATAAATGTAACTAATGTATCTCCTTCATAAATGATTTCCCCATAATGGCCATGAACAGTTGCTCCTGGGGTGGCTAAGTAGGGCTTGGCTGCTCTTATTACTACAGAGTCAACTTGAACAATTAGAACTTGACCCGCCTTTAAGTGCGGCCCGTTTTTGGCTATACATAAATTTTCACAAAGAAATTGTCCAAGACTTATTTTTGTGGAAGTCTCTTCATCTTCACAATAATTGTGATGAAGACAATACC------AATTCAATTTGAACGGATTCAAAATAATGTTACTTACCGGATCGGGATTATAAATCTTCCTATTTTCATCGATTAAATAATATTTCATTACTCGAAAAGTTTGTTTTAAAT------TGTCAAGTTGCAAATAGTTAGTTACCAAGATCTGATTATGAGTTATTAAACGGTAAAATGAATAAAAATTCGCAATTTGAAGGACTGTTCCAAAAGGGCCCGACAAAGTCCTAATTGGAATTCGCGGGTCGAG------------------TTCTTTGTAAT------ATTTTAGACCCTTGAATGGACCCATTCGAAAACAATTGGCTGATGACAAAATGAGAAAAGATTGGCATTCCTTCGTTCTATTCAACAACGTACGAACAGTTTCATGATTTTGGCTAAAAGATTGTTGAAGTC---------TTGCTTTTGAATAAATG------GAATAAAACGGATTCATA------CGGTCTGATCCATTATCAGAAAGCAATCCTGAACCTGATGGATCGTTCCTTTTTCCGGCATACGAAATTTTGGATTTCACTAAATCGATTCTTAGAAAATTTCGAATGATACCATTTGTCTTTACTTCAACAAAGGCAGCACGCGCCTCTTCAATAGACGAACTTTTTTTGTCTTGGTCCCAATTCAATACTAAACAAGTCCGAACTAATTGAATACTTGTGTCAGAAATTCCCCGAATAGGCTTGC---CATTTCCATAAAGGATATAATTGACAACTTGAAGTTGCACCTTATTCCTTTCCTGCAACAGATCCTGAGGGAAAAGTGTTGATAAACTTATACCGTCCGTTATTTCATATGTGACTACGGGTCGAACCAAAACAAAATACCTTTTCTTAGTAGGTGTGATTCGTTGGACATAGATCCA------ATTTTTCAAATGTTTCGAT---------TTTGTTTG------------------TCCCCTTCCCGGCGGTATCAAAATGCCCCTGTGTCGGGATATTTTATCCGTTTTTCCGGGAAAATGGATATTTCCCGAAAAGATTTTTATTTCAATCTTTTTTTTTTTCTTCTCTATTCGGACCAACCCGCCTACCCGGCTTCTTGTATTTAAAGTTATTTCTGTATCTACTCCAATGATACTATTGTTCCGTAC------------------------------------------------------------------------------------------------------------------CATTATGGAAGAAGATCGCGGTAAGATATGTACTTCCTCGGGAATGAAAAAAAACCGATCTACTTGCATTTGGTATTTTGCTTTAAATTCTTTGACTCCTCGATATTC------AATCAAACCCTCTTTTTTTATGATTGAATGCTCCTCTAT------AGTCCCATATTTAGTAATTCCCGAACTGTCTCTTCGATATCTAGGATCATCAAAATAAGCAAGAATACTATTTCTACGGAAAATACCATTTATGGGTATTTCAA---------TCGAGATAC---CTGAAGGGGGCATTAATTCTTTCTCTCGTTCTTGAATCGATTGATGAAATGGAATGGTGAATCTATTTCTTCGTCTCTTTGCCAATAAATCAGAGTTTT---------TGTCAGGATATATGAGATTAT---------AATGCCCAGTTCTTACGATTCGATTTAGTTTTGAATAATCAGGAATCCTACCCCCCCCTTTTTTTCTAGAA---ATAGAAAGATCCGAACTCAAAAATTTACGTCTCGTGTGAACATTGGTCACTGAAGAGTTAGAAATATATCTTTGTTCGACAGAAATAAAATGAGCGCTCGTTTGATCTTGATCCTTGTGGAGCGAACGCGGGGCCACAATGGATTTGTGTGGCCTTCCTGCTAATATCCATAAATGACTTGTTTTTGGTAAGAGATGAACATTACTATATGTAAATTCGGGTGCATGGTCCACGTCGGTACTCCAGTGCATTTCTCCCTCTGAGTCAGAATAAATATGTTTTCGAACCTTTTCTTTAAAATTAAAAGTGGATGTTCCCGCGCGAATTTCGGCAATCACTTGTTCTGAGTCTACATATTGATCATTTTGAACTAAAAGAAAACTTTTTGGCGGAATATTCACATTATGTATAATATCTTCACTCTCAATAGTGACAGCCAAGTCTATATAACAGAGAAAGGCAGGATGGCCGTGACGTGTACGTGTGGGGTGAACCAAATTCTCGTTGAATTTTATTTTTCCATTAGAAGGGGCTCGTACATGTTCTGCAGTACCCCCTGTGAATACTCCACCAGTATGAAAAGTTCTTAATGTGAGTTGAGTACCCGGCTCTCCAATGGATTGTCCCGCAATAATACCTACAGCTTCTCCCAATTCGACCAGGTCACCGTGAATAGGACTCCGGCCATAGCATAATCGACAGATCCAAGATGTACTTCTACAGGTAAAGGGAGTTCGAATAGATATTGGTTGGACTTGAAAGGTTATCAATCGATTGACAAGTCCAACCCCAATATCCTGATTTCTAGCGGCAATGCACCGCGGGCCCATATATATATCGTCTGCTAATACACGACCGATTAGTGTTTGGATAAAAATTATTTCCGGTA---TCGTCCCGTTTTGGGGACTCACAGAAATACCTCGGATGGTGCCACAATCTCTTCTACGTACAACAATATGTTGAACTACTTCAACAAGTCTTCGCGTGAGATATCCAGCGTCTGACGTTCGGACAGCAGTATCCACAACTCCTTTACGGGCCCCGTAGCAAGAAATTATATATTCTGTTAACGAGAGTCCTTCGCGTAAATTGCTTTGAATAGGTAAATCAATCATTTGTCCTTGTGGATCCGACATTAATCCTCTCATGCCTACTAATTGGTGTACCTGAGAGGCATTTCCTCTAGCTCCTGAAAAAGACATCATATGAACTGGATTATAAGGGTCAGTCATCCTAAAATTAGGATTCATTTCTTGTCGCAAATGTTCACTTGTAGCATACCATATCTCAATGGATTGACGTAATTTTTCTACCGCGTGGATATTCCCATAATGATGGTGTTTTTCCAAAATTAAACTTTGTTGCTCAGCATCTTGGACTAGCCATCCCTTAGAAGGTATTGTTAAAAGATCATCAATTCCTAATGAAATAGATGTAGCAGTGGCTTGTTGGAAACCGAGAGTCTTTACTTGATCCAGGATGTGTGATGTATATGCCATTCCAAAATGATCTATTAATCTGCTAATAAGTCGTTTCATGGCAATTCCATCTATTACTTTATTGTGAAAGACCAGATTGGCCCCTTCTGCCATAAGTACCTCCATATTCCGCT-----GAGTGGGATTCGACAATGAATGGGTTTAAGTTAGTGAGTGGAAAACTTCCTTTTCTAGATCT---TAATTCGCGTATAAATTCACGAACTATG-----ATCCTAGTTGAACTCGAGCGGGCCGAATTCCAT-------CGGT------ATCATAGAGT-------TACTTAGCT-A-----GATATAATTAAGTACCGTATGAGCAGGCTCGAGAAAATCCTTGTATAGCTTCTTGAATTTCTCGATAAAACGAAATATGACCAACGGTTGTTCGAATGTATATAAAAAGAATTTCCTTTTTTACACTTCTTACTATTAAATAGTGCCCATAAATCTCATGATAGGTACCCAAAGATTCATAGTGAACTTCGATGGGAGCTTCTC------TTGAAACCACAACACGTTGATCTAGTTGCCACCGGAGCCATAAAGGACTATCTAAATGGATTCTTTTTTGACGATAAGCCCCAATTGCATCATAGGAATTACAAAAAAAGGG------TTCTTTCA------T------ATAATTATAATCGTTCAATTTTTCATTTT---TATAGTTTCGTCGACTCCACGGATTATATCTATTTGCACAAATACCTCGACGATTCCCACCCGTTAATACATAGAGCCCAATAAGCATATCTTGAGTTGGTACAGAAATGGGATCCCCAATAGCCGGAGACAAAAGATTCATATGAGAAAACATAAGTAAACGGGCCTCCGCTTGGGCCTCCAAAGATAAAGGTACATGAACAGCCATTTGATCCCCATCAAAGTCTGCATTGAATCCTTTACAAACCAATGGATGTAAACAAATAGCACGCCCTTCCACTAAAATGGGTTGGAATGCCTGTATGCCCAATCTATGCAGAGTAGGCGCTCTATTTAATAATACAGGATGCCCCCGCATAACTTCCTGAAGTATTTCCCATACAACAGGTTCTTTTTCCCGAATTTTACTCTTAGCAACTCCTATATTCGAAGCAAGATCTTGTCTAATTAGACCGCGAATTACAAATGTCTGGAAAAGTTCTATTGCAATTTCACGAGGCAATCCACATCGATGTAATGAAAGTGAAGGGCCTACGACAATGACGGAACGCCCTGAATAATCGACCCGTTTGCCAAGAAGAGTTTCCCGAAACCTTCCCTCTTTACCTTCAATTACATCCGAAAATGATTTGTAAACCTTATTATGACCATCCCTCATTGGTTGTCCGCGTATTCCATTATCCAGAAGTGTATCTACGGCTTCTTGTACCAATTTCTCCTGGCACATTACTAATTCTCCGGGCGTAGATCTACTTGTTGTTAATAGATCAGTAAGAGTATTGTTCCGATAGATAACTCTTCTATAGAGTTCATTAATATCCGAGCTCATTAATTTACCTCCATCGATCTGAATGATCGGCCTCAATTCGGGAGGAAGAACTGGTAATAGACACAAAACCATCCATTCTGGTTCTATATTTGTTTGAATGAAATGCTTAACTAATTCCATGCGTCTAACCAAAAAATCCCTTCTTCTTCCAACTTTTCGATCTTCCCATTCATTTCCAGTAGGCCCTTCTTCCCCTAACTCTTTCCATTCTGTCAACGAAGAATCTAGAATAGTTCGCAAATCCAAATCGGCTAATTGTTCTCGGATAGCACTTGCTCCAGTAGATATTTCTCGATTTCGAAAAGTATCGAAGCCTTGGGTAGTAAAAAAAAGTGGGATACTATATTTCCAAGATTGGAT---TTCATATTCGAATAAACCCCGTAATCGTAAAAAAGTAGGTTTTTTAGCTATGGGCCTAGCAAAAGAAAAATTGGGATAGGATCCTATACTATAAGATCT-CCCCCCTTCAAAGCCGGACGTGAAAGTTTCCTCTCATCCGGCTCAAGTAGTTACAGC--------------AAAAAAAGGAGTTCTCGCTTTCAA-----ATTCTA-GAAATTATGTA--------AAATCCTCAAAGCAAAACAAAAG------ATCTACTCCTTACTCAAGTTCCCGTGGAAGACTAAGCAACATTTCATTAATCCAGTCTTC----CGTTT------AGTTAG---ATTTTTTGAATTC-------------------CAAATTCAAGTCAAAATGAAATGTGAAATTCTTGAGTAGTCTACCTCCCCTCGAACGATGAATCCCCTT-----------------AAAGAAAGAAAAAGGGCAGGTCCTTGGAATTCATAAGATATAAGATAAAAGATTTACTTGTCTATGGATCGTTTCATTCGATCTTTTAGGTCACGACTTCACCTCGACGGTTATACCAGGATGCCCTT-------AAGCCTATATGCGATGGATAGACTCTTGTAACCATG--ACA-------TATTTGCTATTTGCTTGCGTG-AACATAATTTATT---------TCTAAACGAC----GGAGAGTGGTTAATTCCACAAACA-----AAAGAAGTC-------TTT-TTTTACGAGGTACAACTATAAAT-----TAAAATTTACTTGTTACGAAATCAACCATGGATCAATTCC--CTTTTTATT-----TGGGAGTATTGAATACACCCATAATTCTGAGCTTCATGTTACTCTTACCAAGAGAC-----ATGTCAGAGCCGGGGCATCCCAATTCGATGGAATGGGATAACAGTTTCTCATTTCGAATCTGTAAAATCAAAATTTCGATCAAATCACACATCGCAGTAGACTAGGCCCTCTAATTCTTTAAGAGGTTTATCTAAAAAATTCGCAATATAACTAGGAAGACGCTTCAAATACCACACGTGAGTTACTGGGCACGCCAATTTTATGTAGCCCATTTGATACCTTCGTATTCGAGAATCAACAAATTCGACTCCGCATTGTTCACAAAATTTCGGTTCTTCTTTTTCATCTGCGATTACTCGAGAATTTCCACAAGCACAAATTCCGCTTTTTATAGGCCCAAAAATTCTTTCACAAAATAATCCATCTTTTTCCGGTTTATTGGTTTTGTAATGAAAAGTATAGGGTTTTGTGACCTCTCCAACTATCTCTCCGTTCGGTAGGATTTTCGTGGCCCAAGCACTTATTTGTTGGGGAGAAACTAATCCAATTCGGAGTTGTTGATGTTTATACTGATCTATCATAGAATAAAATTTTGAATTCATTCCGATTAAGCTTCCACCCTATTAATCTGCAAATTCTTCTCAGATACAAGGAAATGATTCAGTTCCAGAGCCAAAGATCGTAGTTCTCGAACGAGCAATCGAAAAGATTCTGGAGCATCCTCGGGGTTAGGTATTGTTCCTCCAACGATCGTAGTACCAAGTACTTCCTGGCGAGCTCTAATATGATCCGATTTATAAGTAAGCATCTCTTGTAAAATATGAGCAACACCAAATCCTTCGAGAGCCCAAACCTCCATTTCTCCTACCCGCTGCCCCCCCTGCTTGGCCCTTCCTCTAAGGGGTTGTTGTGTAACAAGTGCATAATGCCCACTAGAACGTCCGTGGATTTTATCATCAACTTGATGAATTAATTTCAAGATATAAGGATTTCCGATTATAACAGGTTGTTCAAAAGGATCTCCTGTTCTTCCGTCAAATATTCTGCTCTTTCCCGGATACTCGGGTTCAAATACCCATGGGTTGGCTGTTTGCTTACTGGCCTCATATAATTCAGAAAACACTAGTTTTCGCGAAGCCTCTTGTTCATATCTCTCATCAAAAGGTGCTATTCGATAATGTCTGCCTAGCAAACCTCCCGCTAATCCGAGTGAACATTCAAAAATTTGCCCTACATTCATTCGTGAAGGTACTCCTAAGGGATTAAAGACCATATCAACAGGTCTTCCATCTTGCAAATAGGGCATATCTTGTCGAGGTAAAATTTTTGAAATTATACCTTTATTTCCATGTCTTCCGGCTACTTTATCACCTACTTTTATTTCTCGTTTCTGTGAAATATATACACGAATCGTTTCCGGATTAGAACTAGAACCACCCCTTTTCCGGATCCATCTCACATCAACAACTCGACCCCTACCACCTATAGGTAGTTTTAGACAAGTTTCCTTTGAAGTGGATACCTGAATGCCAAGTATGGCTCGTAATAATCTATCTTCCGGAGCATAGGATGATTCTTTTGCCATCTGAGGCGTTAATTTACCTACCAAAACATCGCCTGTCTCTACCCATGCTCCCAGCATCACAATTCCATTTTTGTCTAAATTGCGGAGTAAATGGGCTTCTAAATGTGGTATTTCATTAGTTAACCTTTCGGGACCTTGACTTGTCACATGAGTCTGAATTTCAACTTTTCGTATGTGAAAAGAAGTATAAACATCTCCATATACAAGACGCTCACTAATGAGTACAGCATCTTCAAAATTGTAACCTTCCCATGGCATATAAGCTACTAATACGTTTTTTCCCAAAGCGAGTTCGCCCCCAACCGTGGCGGCACCGTCCGCTAAAATTTGTCCCTTTTTAATGCATTTACCCTGCTGAATCCGGACTTTTTGATGCATACAAGTATTTTTGTTGGAACGTTGGTACATAACTAATGGAATGCTTAAAGTATACCCA---T------TACCCGACAAAACGATCTTGTCAGTATCGGTAGAAATGATCTTTCCTTCATGTTCGGCTATAGCAAGAACCCCCGAATCGAGAGCCGCTTGGCGTTCCAACCCCGTTCCAACAATGCACTTCTCGGAATGCGAAAGCGGAACGGCTTGACGTTGCATATTCGAACTCATTAAAGCCCGATTCGCATCATTATGCTCAAAAAAAGGAATGAGGGAAGCTCCAATAGAAAAATATTGGAAGGGAAAAATACTTCGAAAATGAACCTCTTCCCATGCACTAGTCAGGAATTCTTGACGGTATCGGGCTGGAACAACCCCCTCTTCCTGAACACCTCGATTCAAGGCTAAAGAATTTCCTGCCGCTACCATATAGTATTCGTCTCTACTTGGTGATAAATAAAGCATCTGTACCCTTTTTGATTTCTCATAAATTTCATAAAACGGGCTTTCTAGAGACCCCCAAGACCCAATCCTAGCATGAATTGCTAAGGATCCAATAAGTCCAACATTGATTCCTTCAGACGTGTCAATTGGGCAAATACGCCCATAGTGACTAGGATGGATATCTCGTATACGAAAACTAGCAGTTCGCCCTGTCAATCCTCCAGGGCCCAAATAACTCAATTTTCTTCCATGAACTATTTGTGTCAATGGATTAGTTTGATCCAAAACTTGAGATAGTGGATGTAACCCAAAAAAAGATTCATAAGTGGTTGTTAATGGAGTTGAAGTTACTAAATTCTGAGGAGTCGGTATTAATTTATGCCTAATTGCTCCACAGATAGTTCCTCGAACCACATTTTCTAAACGAACCAGAGCCAATCCAAATTGATCTTGTAATAGATCCGCCAAAGAACGAATCCGTTTATTTTTCAAATGATTCATATCGTCAAGTGTACCCATTCCAAATTTCATTCCAATCAAATGGTCTGCAGCTGCCAATATATCTCGCGGTAACAAAAATGTATTGTTTTGAGGTATATCAAGGTTCAGTCTCTGATTCATATTTCGTCGACCAATTCTTCCTAATTCGCATCTTTGTTGAAAGAATTTTTTTTTTAATTCCTTACATAAGGATTCAGAAAATACCGGGTCCCCGCCTACACAAGCAAATTGTTGATAAAACTCCAAAATCGCATTTTCTTTTGATCCAATTTTTTTTTTCTCCTTATCATTCAGGAAAGACAAGAAAATTTCCGGGTAGCAAACGTTCTCTAGAATTTCTCTTAGACTCGAACCCATAGCTGATGATAGAACTAGAATAGATATTTTCTGTTTCCTACTCACACGAGCCCATATCCTTGCTTTTCTATCAATCTCTAATTCTGATCTTCCTCCCCAATCTGATATTATGGTGCCGGTATAGACCGCAACTCCGTTATGGTCCAATTCTGACCGATAATAAATACCGGGGCTTTGCAGTATTTGATTAATCACAATTCTGTAGATTCCGTTTACTATAAAAGTTCCCAGGGAATTCATTAAAGGAATGTTTCCAATAAAAATGGTTTGCTCTTGCATATCCCTACGGGTTCTCCAAATTAACCCCGCGGATACGTATAATTCAGAAGAATATGTAAGCGATTCATACACAGCATCTCTTTCTTTTAGCAGCGGTTCCACCAATTGATATGTTTCCACAAATAATTTAAATTCAATTTCTTGATCTGTATCTTCAATCTTGGGAACCTTATAAAGTTCTTCCGTCAAGCCCTGATCAATGAACCTACAAAATCCTTCAAATTGTATCTGATTAAGCCCAGGTATTGTCGATATTCCCTCATTTCCATCCCGGAACATTTGAAACGAATTTCCCA-------TTTATAGAAAAA--TCCCATT------ATTATCGCATTCTTCATCG-----AATCATATAGATTTGACCCAACGCCGATGGAATTTCTATTCTGT-------TTACTGAATCACATAAAAT-----TTTA-CCCAATTCCATACCATATATGTCCATATATGTCCATATATGGACAGTATGAAATATGTAT---------GGGGGGGTAGAGAGAATTTTCTACTCAAG---TAAAATTTCGGAAT-------TTAATT-----TGTAAGA-GAAAAGATGAAA-GGAATTGATAAAAAATTCTTGGAACCAGAATTCTGCTGCTTAGATT--------TATGGGCTTT-------------AGTATA-------------------------TAGTAT------ATCAG-----------------AACAAAAGTGATTCAATTACTAC-------------TATTATAATGAT-----------ATTATATATTCCAAT-CGATTGGATA-CCGG----AAAAAGAAAAGGATTCAGAATTTGATCTGTTTGCC-----GAAA-----TAAATATAGAATAATCAGAAACAGTACAACGTTGATT--TTT--TCTTACT----TAACCCCTT--------------------------------------------------------------------------------------------------------------------------------------------------------TTGGGATTTCATTGT-AAAAAAAA-T-------------TTGCCGAG----AAAAGAAAGATAAACTTTGACCGATTCTCT----------------------TATTATTACTTAG---------TACTAGAATTTGT-----AAAATACGC-----TTGGGAAGC----GGGT-------------TATATAT---ATATTTAGTT-----GTATTTATT------AAACATATGTAGCTATC------------TTCTATATATCCCTATAT--------------------ATCCGTCTCCCCTTTTATTCTTTTAGTGCAGTTCTATTCGGGGCAG----CGCGGGCGGTGGTCTATCCAAA-------TTTATAC-TTTTT----TCAATCGATTC----AATGAAAAATTTAAGTACGAT-ATTTTCTGAGAATT--CCCTACCGGCATCA-----------TATATTATATTTGAATAT-CCGATTATATATCTGGG--------------TAAGTTCTGTTCTGGTTCCGGGGTTTCCTTTACATATAT-----------------------------------------------------------------------------------------------------------------------------------------------------------------------------------------CAATATTT-----------------TTTTTATTATAATTAT-------------------------------------------------------------------------------------------------------------------TTATATTTATTATCGAAATTG---AGAAAAACGGAAATTAAGAAAGA----TTTTTTGATTGA---------AAAGAATCAAT---CAA---------------------AA----TTAGTTA-----------TTATTTTGACTTT---------CTTATGTCATTAGGGAAACAAA----ATTTTCGACCCAAATCTCAA-----AATCA-------TTCACGAATTCACATTCAAGTCAC------AAGTCAATAGTTAAT--------GGTTCAAATTCTTTATGAA--------TTT-------TTTTGTGAAGGAAAATTT-AAATTTTCCCTTTCAATAGAAA------GGATAGGGGAAGTTTTTAGGTATTGTGTATTTTGCGATACT--AT-AGAATC--AATCGAAGGGGTGGATCTAAT--------------------------------------------------------------------------------------------------------------------AA-AAAAGGGAGTAATATTGTCATCTAT----------------------------------------------------------------------------TTT------TTTTGGCGACATGGCCGAGCGGTAAGGCGGAGGACTGCAAATCCTTTTTTCCCCGGTTCAAATCTGGGTGTCGCCTGGTCAACAAAAGACCCGAAATCC--------------CT-------------------------------------------------------------------------------------------TGAAGATTCTCG-AAAGATCTGTAACTTTTTGTGTCTAGAATTCAAAAAAG-ATT---TTCGTACTCTGAAGGGA--------GTCGAGAAGTCTTGATAGCCCTTCCA-----CTAC---------TAATGGAATG-------------------------------GAATAT--TTACTGACTGGGCCTTGAATTA-------GATTGGATAACCAAG-GGGGAGTCTTACTAAT------------A-------------------------------------------------------------------------------------------------------------------------------------------------------------------------------------------------------------------------------------------------------------------------------------------------------------------------------------------------------------------------------------------------------------------------------------------------------------------------------------------------------------------------------------------------------------------------------------------------------------------------------------------------------------------TAGCATGAACTA-ACAGTTAGACTCCCCT----GTCCATG-GGGGTCGTTGCATATTTTGCTTGTACTTAATCTTTCCCA-----ATTTTACTAG--------------AAATCATAATGATAAGAAAATTTGTATTAAAAATT----------------------------------------AATTATAAATGCATTTATTGAATTGGTTCATTACATAAAGAATTGGGGGTAAGAATCCCCTTTTGACTATACACCCTGGATTTCACTATTATTACTAAAATTATTAGTAAACAAGAATGGAA----TAATTCCTTCAGA-----TTCATAGAGATAGGGGACATAATTCACATGGATATAGTAAGTCTCGCTTGGGCTGCTTTAATGGTAGTCTTTACATTTTCCCTTTCACTCGTCGTATGGGGGAGAAGTGGACTCTAGAAGTAC------TATT----------AATGTAATTGCGGT-------AAGGAATCAAAC-TTTATAAATTGGTTTATAGATCA---------------------------------------------------------------------------------------------------------------------------------------------------------------------------------------------------------------------------------------------TTCTA-----------------------CAAAGCGTTTTGTTTGAACTTTAACTATAAAAAAA--------------------AATAATAATGT---------------------CAATCAAAC------AGATATTTCAATGATTCCCATGTTTGTATTTC-----GGAAGGGGATAC------GTATGGTAAGAAATTTTC----------ATTTTCTTAAATT-----CTCTATTTCGCCGAAGGGCTCTTATCT---------ATCAGACTTTCTTATCAGACTT------------CTTA-TATAGGGACAATGGGGAAC-----AACAAGCCACTTCTTATTATTTTT----TATTTAT-------TTGCCTAT----------------------------------TTAAAGAGAAAAAGGCGTCCTGTTATTAAT----------------------------ATTAGGGGGATGCGT-----AC-TTTCTAGGGTAAAGA------ACATCTACATAGTGGTT-----------------------CTTCAACAAGATACTACGCAT-----AATAAAATCTTGCCCCCGGCGAGTCACA----TAT--TGTGTACTCGCCGC-TTTCTTTATT------GTTGTAGAAATTTTATTTAGGC-TTTATCGACATCGACTCATTTAATAGCGCGGTTCAAGTGTTAC-----AAATTGGTAGGA-----TTTACTACCCCTTTT---------CTAAATTGGAAGAAGTTCCCATAC-------------TCCTTTCTGTTAGCGATTCAATCAAATACTT-T-----TTTGGAATGCT-AAA---AAAAGATTACTGGC-T--------TTTTTTTATTTTTTTTTATGGGAA----CCCTGCCCGTAGACT-----TT-TTACTTAAAAATTTTTT-CTTTTT---TCGATAGATACTGGGATCTCGATTT-----GAAATAATAAGAAATCTCGGAATTCAAGCCGTAAAAGTAAGAGTTA------------CCCCTCCCTTTTTTTC----------ATTATTTAG------GATTG-----------AGCGG---AAT-------CAATACA--AATCAATAAAA------TAGA----TGTAGC-AAAGAAATATAACTGGG------------GCCCTAT-GTATATTC-----------------TATAGAT-----------AGAATTCTATCG-------------ATATGAAGATTGCT--------------------CTACTCTAT--------------ATT-AA----------GCCTGTATCTTTAT---ACAGTACAACTT------TATATACTACAAAACCGCG---------------CATAGATAATATGGTAGAAAG-------------ATTTATATATT----TCTTTCTACCATA----TTATA-----AAATCTCGTAGAATACTGTTGATTCTAGCCTACGTATTA---AA-TTGAAGATT----TAAGAAACGAAATTGGAATCC-------------------------TTTATTTATTT--------------------------CTTACAAAATTCAGATTGATAAAGAC-----ATAAGAAGTCAAGTTTCATTCATATT----AATCGTTTTGGCTGACCGTTTTTACATATATGATAAGTAAAAAAGCAGTAGGAACTAGAATGAAGAGTGCAGTAGCAATAAATGCGAGAATATTTACTTCCATAATCTCCGTG-GTTTTTACTTCGCAATAACTCGGGATTTAATCCCATAGAGATAATCAAAATTTCGCCTGTCAATTCAATGGGATGGGATGA-----ATTACATCTCGATGA-----TATTGAATCGCATC-----AATATTATG---------AATAACAATATCTGGACTATCAAATTACTTCGTCGTCGCG------AATTA------------AATAGTATAACATAGGAAGATCCTTTATCCATAC------T-CAAT------AGAA------------AATGGAATTCGTAATCGAATCAAGAAATCTT----TTTCTTTATT----ATTCTTTTACA-----CT-----CTTTCTACAACCTAC----CATCTTCCTTGGACAATCATCGGATGAAGTAGCATCTGACCG---------CTTTCCAC-TTACATTGCATTGATAACAACCCCCC-----CAAAAAATAACAACAC-TCGAAGTAAAATGAAA---------GGGGCG------GG-----GGTTAAACTCGAAACTCCTA-----------------TTTTTTTATGATATAATTTTC-TTTTCTTGT-------------------AAGAAAA--------AGAAAAGTGTGAAAAAGCCAAATTCCGGTCT-ATCTAAA------------------------------------------------------------------------------------------------------------------------------------------------------------------------------------------------------------------------------------------------------------------------------------------------------------------------------------------------------------------------------------------------------------------------------------------------------------------------------CAAAATCGGAACTAGAAAGGGAGAGAGTTTT-------AATCTGAAACG------TC-TTTTCGGGGTAACTCAAAATTTA----TTTTGTAGTGTACAAGA-------------AATGAATTCTAG-----------TTGTGTATGTGC--------TCCCGAGAAACATATGATACTCTATTCCAT----------------------------------------------TAGATAGAG-----GCAATAAATTG-------AAAGACTAGACCCAGTACGCTATCCCTTGGA---------------ATCCTGAATA-TGATGTTCCCAATAATTGGACTAATCCAATTATATCTCTCTCCC-------ACCA---------ATAGGTA-------------------CTAGTTGAAGTAATGAAAATT------TAT------ATATT----------TTTGTTTG---ATGAGAAATAACTAAAAAAG--------------------------AAAAAAATCCCTA------TTGA-------GAAGGACCTAAATTCTA-------------TTCATTT---------CATTA-------TGCTTC-TTTTACCAAAAAATGAAAATGAAGAGGCGAGTTGATGTGTTTATTGGATCCGTCGGGACTGACGGGGCTCGAACCCGCAGCTTCCGCCTTGACAGGGCGGTGCTCTGACCAATTGAACTACAATCCCAGGGAAA----TAAGGTATACCGCATCAAGATTT-------TTAGGATTTAATTCCAA--T--------------TTTTCGTGTTGTAACAGAGACACGAGTGATATAGTGATATCTA----CATGACTATGCACTTTCT------------TTTTAGTTTT-------AGTTAGAGTG-ACACGAATTACAT----TATTAGTGATTCTTTCC----TTATTTCCAAATC----------------------------TCGATTGATCATCA---------ATTTTTC-AAT---------AAAAAAGATTTAT---------TTTCTCGTTTCCT----TAGACTTTCTTTTTTA----------------GACTTATAA----------------ATACT--GATATGAATCTAGATCATTATATTATCTAG--ATCGGA------ATTTTTTGGAAC-AAATAAATCGAGCAAATAAAACAAG------------GAAGGTA------TGTATATAGAAAAAAATTTAATTCTTTTATTCCCACGTATCGCGCG-----CTTTGGAAAGACAAAAATTTGCAT-----CTCACGGTCTAT-GAGCGAATTCTT--GGGCCGAGCTGGATTTGAACCAGCGTAGACATATTGCCAACGAATTTACAGTCCGTCCCCATTAACCCCTCGGGCATCGACCCAGG----AAAAAGAAATTAC------------------TTTTTCAATTTAAGGCTTATTGATAATGCACGCTCAACTTCCTTTTGTAGTAACCTACCCCCAGGGGAAGTCGAATCCCCGCTGCCTCCTTGAAAGAGAGATGTCCTGAACCACTAGACGATGGGGGCATATATGTCCGACCGCC----ATGATACTAT-GCTCATAGTATCAACAGTTTTTCGA-AATT---------GTCAATAGAGTCAATAGAATGTAAGAATATGATGCGATCC------AAGGTA-------------------------TCCTTAGAGTTTTTTTATTCATCATTCC------TATTTATGAATCCTTAATTCTAATCG-----CCATTCGATTCAATATAT-----AAAGCCA------GTAT-C-----ATTATCCA---TAT---TATT-TAATTTTTTTTATGAGAATTT--------------------------------------------------------------------------------------------------------------------------------------------------------------------------------------------------------TT--------TGATTCGAATATAATTCGAA----------------------------------------------------------------------------------------------------------------------------------------TTTCATTTCAAAAC-----------TTAAACAA---------------------AATTTAAAC--TTCGAATATCTA----------------------------------ATAGAATTCGA--------------------------TCTAAAATTCTATCTAAATTTAAATTAAT-------------TAAATAT--------------------------TAAATAAAAAAAGTCTCGATT-----------------AACTATAT------AGAAAATAAAAA------------GATAGAAAATAAAAAGCATTTCTT-------------------------ACA---------AAGAGAAAA-----AAATACGA-GGGAAAGGAT------------------CCCTTT-------------GTGGAATGGTTTATA-----------CACC----CCAAA---------AAAATAAAAATA---------AAATTTTCAACTC------CATTTCTTCGA--------CTTTC------TTGATTCATTCA----TTTTTTTAAG---------ACGAAATATGTCTTC-----------------------------GTAG--------------------------------TAAACCAGAAA------ATTTATAAAGGAGAAATTCGGGATGATAA-------------AGGGGATCAATAAAATTTCATAAATTG-------TTTTTTATGGATTAAG---------GGGGAAGGGAACAAATCGAACTTCTCCATCACA-TAATTTAAT--------------------GAAATATCTTGG-------ATCTGC---GTCGAATTGGTAGG----TATATGTCTCAAGTGATTTTTTTTT----------TATGATGGGGAT-------CAATTTAATAAAAGAAA---------------------------AAAAAATGAGGGTCGACTTGGTTCATTGAAGTGATAGTTTAAAAAGATCAAAAAATCGTTTGATCCGAGAC-------TCGACAG------------TAAATCAACTTTA------TCATTCCGGAAAGAGCCACCCGCCGCTATGAATTTACTT------------TATT---------------------------ATA---------TAGTAAG-----TATAGTCTATAA-----TT------------ATATA--TATT-----------ATGTAGTTG--------------------------------------------------------------------------------------------TAATATAGGGAGAGAGAGATAT----TTTATCCACATAGTGACTC-ATTCAGGAATT-CAGTTA-GTTAGTTAGTTTAAAGGGCCCCTTTAACTCAGTGGTAGAGTAACGCCATGGTAAGGCGTAAGTCATCGGTTCAAATCCGATAAGGGGCTTTTTAC--------TTTTTTTCATA-----AACCCGAGCCGTAGTATTCATAT------TTGAACGTAGAA--------TCGATTTGTTTCTTGCT----------------------------------TTTTTTAAAGGAAAAAGG----------AAACAACTGTAT-----AAT--------------------------------ATAAGGATAATACGTCCTA-G-----TGGTTA----TAAA-GTTAAACATCTTTTC-----AT-----TAT--------------------------------AATGATA------------AGAGAAGTTGTTTATT------GAATCA----CCAAATATTTA-------TATAT--TTTTTTT-------TTCAATTATTCCAAACTAA------ATCCATTGGAAAGATTTAAAATCAGC--------AAATAAAAAG-GGAAAAGTAAGTGGACCTGGCCTATTGAATCAGGACTATATCCGCTATTCTGATAATAAAATTAGATAGAGATGAAATTAGAACGGTTGA-CCCCTT-----TTTTATTT----AATTTCTTTGGACTGCGCACCAATTTGTCGATATTTCCGGTTAAATTTTGGTATTCCTA---GATATTCCGTAAG--TAAGAATAATAAGAATA---------ATTTAGC--TTTTCCCTTCCTT-CATAGAGACG-GAAGGGAAAAAGTTTCTTCTAAATCACAAGAT-AAAAAAACTTTTTTCGCTATCTTTC------TTTGATTCCA------GAGGAGG--------------ATTAATATCTA-TTTATCGAATAA-------------------GATTTAAATATATC-------TTATTCGATCGTAATTTCATGTACCAACTCTTTTTAGATCGATGCATCTCATA-TTCTTCTTTCGCCACTGGGGTGG------AAAATACATGCG---AGAAAAAAG-TGTATTTCGGGTATCCTAGTCTT-----TTTT------------------TATTGAAT-----AT----TGTATGGAA------TTTTTTTAAGTTAGGA---------AAAAACGGTAATTCTCTCT-----TTTCCTGACAAATAAAAGT---AAAAAAAAAAAATAAATAGAAAATTTTGCA--------TTCTTTCCTCGACCCATG----AAAATGAAAAGAGAGAC-----TCTGAAG-TTTTTGATTCATCT----------------AAAGGAA---------------------AGGGAAAACGGGCAGAAAATACTAAAATAATG----ATATTTAT---------------------------------ATTATATATAAATATA--------------------------------------------------------------ATATA---------------TTATAT----------------------------------------------------------ATAATTAT----------------CTATATAAG-AGAT------------------------------------TCTATAGAAGACTT--------ATTCT----------ATATAAGACTGTAGTAG------CATACATATCAATTCGAAA-------------AAACT-------CTCAAGATATTTCA-----TTTTT------------------------------------------------------------------------------------------------------------------------------------------------------------------------------------------------------------------------------------------------------------TCAGG------------TGCTTATTCAGA------CTCGTTAA---------AGAATAATCGAATTGAGGTTAGGGATTTACTTTCAAT-----TAAAGGTAAAGT----------CAATTTATGAACCA-----ATAAAG--------TTTATCTTCGAAACCTGTTGT---------AAGAGTCAATGTACGAG--A-AAAATCATACATAAATGATCGAATTCTCGGACTCC------GCGCTATGAGGTGTTCGAAAATGGTTGAAATAGTTGAATAGGAGGATCACTATGACTATAGCCCTTGGTAAATTTACCAAAGACGAAAATGATTTATTTGATATTATGGATGACTGGTTACGGAGGGACCGTTTCGTTTTTGTAGGTTGGTCCGGTCTATTGCTCTTTCCTTGTGCGTATTTCGCTGTAGGAGGTTGGTTTACAGGTACAACCTTTGTAACTTCATGGTATACTCATGGTTTGGCCAGTTCCTATTTGGAAGGCTGCAATTTCTTAACCGCAGCGGTTTCTACTCCTGCTAATAGTTTAGCACACTCTTTGTTGTTACTATGGGGCCCTGAAGCACAAGGAGATTTTACTCGTTGGTGTCAATTAGGTGGCCTGTGGACTTTTGTTGCTCTCCATGGCGCTTTCGGACTGATAGGTTTTATGTTACGTCAATTCGAACTTGCCCGATCTGTTCAATTGCGACCTTATAATGCAATCGCATTCTCTGGCCCAATTGCTGTTTTTGTTTCTGTGTTTCTAATTTATCCACTAGGCCAGTCTGGTTGGTTCTTTGCGCCGAGTTTTGGTGTAGCAGCTATATTTCGCTTCATCCTCTTTTTTCAAGGGTTTCATAATTGGACATTGAATCCATTTCATATGATGGGAGTGGCCGGTGTATTGGGCGCCGCTCTGCTATGCGCTATTCATGGTGCTACCGTAGAAAATACTTTATTTGAAGACGGTGATGGTGCAAATACATTCCGGGCTTTTAACCCAACCCAAGCGGAAGAAACTTATTCAATGGTCACTGCTAACCGCTTTTGGTCCCAAATTTTTGGGGTTGCTTTTTCCAATAAACGTTGGTTACATTTCTTTATGTTATTTGTACCAGTAACCGGTTTATGGATGAGTGCTCTTGGGGTAGTCGGTCTGGCTTTGAACCTACGCGCCTATGACTTCGTTTCCCAGGAAATCCGTGCAGCAGAAGATCCTGAATTTGAGACTTTCTACACAAAAAATATTCTATTAAATGAGGGTATTCGCGCTTGGATGGCGGCTCAAGATCAGCCTCATGAAAACCTTATATTCCCTGAGGAGGTTCTACCACGTGGAAACGCTCTTTAATGGAACTTTAGCTTTAACCAGTCGTGACCAAGAAACCACCGGTTTTGCTTGGTGGGCCGGGAATGCCCGACTTATCAATTTATCTGGTAAACTACTCGGAGCTCATGTAGCCCATGCCGGATTAATCGTATTCTGGGCCGGAGCAATGAACCTATTTGAAGTGGCTCATTTCGTACCAGAAAAGCCGATGTATGAACAAGGATTAATTTTACTTCCCCACCTCGCTACTCTAGGTTGGGGGGTGGGCCCCGGTGGGGAAGTTATAGACACTTTTCCATACTTTGTATCGGGAGTACTTCACTTAATTTCCTCGGCAGTATTGGGCTTTGGTGGGATTTATCATGCACTTCTGGGCCCTGAGACTCTTGAGGAATCTTTTCCATTCTTCGGTTATGTATGGAAAGATAGAAATAAAATGACCACAATTTTGGGTATTCACTTAATCTTGTTAGGTCTAGGTGCTTTTCTTCTAGTATTCAAAGCTATTTTTTGGGGGGGCATATATGATACCTGGGCTCCAGGAGGGGGAGATGTAAGAAAAATTACCAACTTGACCCTTAGCCCAAGTATTATATTTGGTTATTTACTAAAATCGCCTTTTGGGGGAGAAGGATGGATTGTTAGCGTGGACGATTTGGAAGATATAATCGGAGGACATGTATGGTTAGGTTCTATTTGTATACTTGGCGGAATCTGGCATATATTAACCAAACCTTTTGCATGGGCTCGCCGCGCACTTGTATGGTCTGGAGAAGCTTATTTGTCTTATAGTTTAGGTGCTTTATCTGTCTTCGGTTTCATTGCTTGTTGCTTTGTCTGGTTCAATAATACCGCTTATCCGAGTGAGTTTTACGGTCCCACTGGACCCGAAGCTTCTCAAGCTCAAGCATTTACTTTTCTAGTTCGAGACCAACGTCTTGGGGCTAACGTGGGATCCGCTCAAGGACCTACTGGTTTAGGTAAGTATCTAATGCGTTCCCCAACCGGAGAAGTCATTTTTGGAGGAGAAACTATGCGTTTTTGGGATTTGCGTGCTCCTTGGTTAGAACCTCTAAGGGGTCCAAACGGTTTGGACTTGAGTCGGCTGAAAAAAGACATACAACCGTGGCAAGAACGCCGTTCCGCGGAATATATGACTCATGCTCCTTTAGGGTCCTTAAATTCTGTGGGTGGTGTAGCTACCGAGATCAATGCAGTCAATTACGTCTCTCCTCGAAGTTGGTTAGCTACTTCTCATTTTGTTCTAGGCTTCTTCCTATTCGTAGGTCATTTGTGGCACGCGGGAAGGGCTCGTGCAGCTGCAGCGGGATTTGAAAAAGGAATTGATCGTGATTTTGAACCTGTTCTTTCGATGACCCCTCTTAATTGAGA------------CAAGAGATCCACTGC------TTAAAG----TCGGAACCGTTTTGATTCCATCATACGTATTTG-----GGTCGGG-------TCATACTT----AAAGATGAGTCCCTTTTT-TCTT--------------------------------------------------------TAT-TTA---AAGTTTTTT----------AACTCATA--------------------TATCTCATATAT----------GATAATAA------TATATATCAT------ATAAAATTTT-----------------ACGATATAACT--AA-----------------------------------------AAAATTGTCGTTCTTTTCTGGCTCGGCTAGCTCACCCTAGCCGAGCCATTCCCTTGATT-TTAGTGTACCGGGACGCGCAAAACAAAAAAGG---------------------AAAAAATCT---ATTCAATG----AGCAAAAGGAGAGAGAGGGATTCGAACCCTCGATAGTT-----TTTAGAACTATACCGGTTTTCAAGACCGGGGCTATAAACCACTCAGCCATCTCTC--------CAACCGACAATTTATATTTTATTTTGATTCCTCCGAATAGAACATGGTCATAGGGGTTGATAC------CGTTACTA-----------------TCTATAGATAAATATCAGGTGTGAAATCTATAGGCCG-----ATCTATTTATCTTTAAATACCTCTAT-------ATA-------------------TAGAGGTATGATCT----------------------AGCCTGCCCATTTAGTGACGTAAAAA--AA-TTCCCCG------------CAACTCGAATAAAATGGTA------AAAAA--------------GGTGGTAATAAGTCATAGTATATAGAAAAA--------ATTCATAGT--------AAAAAAAATCCCTATAT-------------AATGCATTTTTTTA-----------CTGATAGAGGGATCAAATGGTATAGT-TCATTTGTTCGTAGT-----TTGGAGGATTATAAGTATGACTATAGCTTTCCAATTGGCTGTTTTTGCATTAATTGCTACTTCATCAATCTTACTTATTAGTGTACCTGTCGTATTTGCGTCTCCTGACGGGTGGTCAAGTAATAAAAATTTTGTATTTTCCGGTACATCATTATGGATTGGATTAGTCTTTCTGGTAGGTATTCTTAATTCTCTAATCTCTTGAACCTATTCGTCCCAGATC-CAAAAATGA-----CCCCT------CCCCCGAATTCGCTATTTTCC--------------GGGTTGTGAGACAGAGT---AAAATT----------------------------------------------CAAT------CAATA-TAAGTCCC-------------CAAAAAG-----------------------------------------AAAAAAATGAGAGGGAGGGGTCAAACTTAAACTTCTTGAATG-AATTCGAT--------GAAATAAAAA-AGATTTA-----AAATTTAATTGGAAGAACCTTGATTGAAAACAGTATCCGGCCCAGCTCTGC--ACAAATGTGGTCAAGACATATATACTATA-----------------TATGTG----------ATATGTGGACATATTCCTTCTCAAGGAGGACAA--------AATGCGGATATAGTCGAATGGTAAAATTTCTCTTTGCCAAGGAGAAGACGCGGG--------------------------------TTCGATTCCCGCTATCCGCTCAAGGTTCAA-GATGAAGTTATTA--------------A-------TATGAT---------TTAAGAGATTTGATATAGTTGACC-AGGATAGTGTAG-----TGATTCTATCTTCTTCCTTCG-----TTTTT-TTTCGCCCACCT-------ACCCCAAAACACAAAAAAGAAA-------------------------AACAA-AAAATTCTTA---ATAAAAAAATCGTGCGGAGACAGGATTTGAACCCGTGACCTCAAGGTTATGAGCCTTGCGAGCT-ACCAAGCTGCTCTACTCCGCGCTGAAGAGAAGAATTGGGA-------ACTGATGTA--ATGGACAAACAA-GAATGTA-----CCCCCTACCATCTCTGTA-------------------------------------------------------------------------------------------------------CAAATAGTATAACCCATTTATACAGAATGGTCAAGGGG-TCCTCTATGACTTATGATCATAG-----AAACGAGCGGATATTTGAATCCTTACCAACTTGATCTTGTTGCCCCTGGCAACAAGCATGTATGAACCATTTCACGAAGGATGTGTCCGGATAACCCAAAGTCTCGATAGTTAGCTCTCGGTCTTCCGGTCAAAAAACAACGCCGATGAAGACGTGTAGGGGCACTATTACGCGGTGGAGATTGTAACTTTCCATGAATTTCCCATTTCTCATTCAAGGACGTAACTTTACTTATTTCTTTCTTTGAGGATCGACGAATCAAATGATATTTCTGTTCCAATTTTTGCCTCTTCTTTTCCCTCTGAATCAAACTTTTACTTGCCAT--AAAGGTTCAGTTCCT----ATTAACATCAATGATACAAGTCGGATCCTAGATGTAAAAATAAA---AAAAAAGGGGGGATACCCTTC-TAC-------ATCGAAAGAAAT-GAGATTATCGAGTATACAACACATTA-----AAAAATTAACCAAATTTTCCTGATGTAGAGGCAATCAAGAAAGCCGCATAAGTGAATATATAACCTACGGAGAAGTGGGCTAATCCAACCAATCTTGCTTGCACAATGGAAAGAGCTACAGGTTTATCTCGCCATCGAATCAAATTGGCCAAAGGTGTGCGCTCATGAGCCCATGCTAACGTTTCGATCAATTCCTGCCAATACCCACGCCAGGAAATTAAGAACATAAACCCCGTAGCCCAAACAAGATGTCCAAATAAGAACATCCACGCCCAAACCGATAAACTATTCATACCAAAAGGGTTATATCCATTGATAAGTTGTGAAGAGTTTAACCATAGATAATCTCTTAACCATCCCATCAAATAGGTGGAAGATTCATTAAATTGTGAAACATTACCCTGCCATAATGTGATGTGTTTCCAATGCCAATAAAAGGTAACCCATCCAACGGTATTTAACATCCAGAAAACTGCCAAATAAAATGCGTCCCAAGCCGAAATATCACAAGTACCACCTCGTCCCGGACCATCACAAGGAAAACTATAACCGAAATCCTTTTTATCTGGCATTAACTTGGAACCGCGTGCATCTAAAGCACCTTTTACTAAGATCAATGTAGTTGTATGTAAACCTAGAGCAATAGCATGATGAACCAAAAAGTCTCCAGGACCTATTGTTAAGAATAGTGAGTTACTATTCTCATTAACAGCATTTAACCAGCCGGGCAACCAGATACTTCGACCCGCATTAAACGCCGGGCCATTTGTTGAAGATAAAAGGACGTCGAAGCCGTATGACGTTTTACCATGAGCAGATTGTATCCATTGAGCAAATATGGGTTCGATCAAGATTTGTTTTTCCGGAGTACCAAAAGCAAGCATGACATCATTATGAACATAAAGACCCAAAGTATGGAACCCTAAAAAGAGGCTGGCCCAGCTTAAATGGGATATGATAGCTTCCTTATGGTCTAACATTCTTGCCAATACATTATCCTCATTCTGCTCCGGATTGTAATCTCGAATGAAAAAGATAGCTCCATGCGCAAAAGCCCCTGTCATGATGAATCCTGCGATGTATTGGTGATGTGTATATAATGCAGCTTGAGTAGTAAAGTCTTGCGCTATGAACGCATAAGCAGGTAAAGAGTACATGTGTTGAGCAACCAAGGAGGTAATAACCCCTAACGAGGCGAGAGCAAGCCCTAATTGAAAATGAATCGAATTATTGATTGTGTCATAAAGACCCTTATGTCCACGTCCCAATCGACCTCCCGGAGGAATATGCGCTTCTAAAAGATCTTTCATGCTGTGCCCAATCCCGAAGTTAGTTCTATACATATGACCAGCAACGAGAAAAATAAATGCAATAGCTAAATGATGATGAGCCATGTCGGTTAGCCATAAACTTTGCGTTTGTGGATGGAATCCCCCAAGAAGTGTTAGAATGGCAGTTCCCGCCCCTTGGGAGGTACCAAATAAATGACTGCTTGAGTCCGGGTTTTGAGCATAAAGATTCCACTGACCTGTAAAAAGCGGGCCTAACCCCTGAGGATGCGGTAATACATCTAAGAAATTATTCCACCGAACGTACTCCCCCCTGGATCCGGGAATAGCGACATGAACTAAATGTCCTGTCCAAGCCAAGGAACTTACTCCGAAGAGTCCTGACAAATGATGATTGAGACGGGATTCCGCATTTTTGAACCAAGAAACGCTCGGCTTCCATTTCGGTTGTAGATGTAACCAACCCGCTATTAAAGATATGACAGAAAGAAATAATAGAAAAAGAGATCCAGTATAAAGATCTTCATTAGTGCGTAAACCGATTGTATACCACCACTGATAAACACCAGAATAAGCGATATTCACTGGACCGGGAGCACCACCTCGAGTAAAAGCTTCCACGGCCGGTTGACCAAAATGAGGATCCCAAATTGCATGGGCAATAGGTCTTACATGTAAAGGGTCCTGTACCCATGACTCAAAATTTCCTTGCCAAGCTACATGAAACAGATTTCCGGAAGTCCACAGAAAAATTATCGCTAATTGCCCAAAATGAGAAGCAAAAATATTCTGATAAAGACGTTCTTCGGTAATATCATCATGACTCTCGAAATCATGTGCGGTAGCAATGCCAAACCAAATACGACGAGTAGTGGGGTCCTGAGCTAAGCCTTGGCTAAACCTTGGAAATCTTAATGCCATAATGCCTTTCAAATCCTCCTAGCCATTATCCTACTGCAATAATTCTTGCTAAGAAGAATGCCCATGTTGTGGCAATTCCACCCAGAAGGTAATGAGTTACTCCTACAGCACGCCCTTGTACAATGCTCAAGGCTCTAGGCTGAGTAGCAGGAGCAACCTTTAATTTATTATGAGCCCAAACGATGGATTCAATAAGTTCTTGCCAATAACCACGCCCGCTGAATAGAAACATTAAACTAAAAGCCCATACAAAATGAGCCCCTAGGAAAAAAAGGCCATATGCAGATAATGAAGAACCATAAGACTGAATTACCTGAGATGCTTGTGCCCATAAGAAATCACGGAGCCAGCCATTAATAGTAATGGAACTCTGCGCAAAGTTTCCACCCGTGATATGAGTTACCACCCCTTGATCACTTATATTACCCCAAACATCGGACTGCATTTTCCAACTGAAATGGAATATGACTACTGAAATTGCATTGTACATCCAAAACAGCCCCAAGAATACATGATCCCAAGCAGATACTTGGCATGTCCCCCCTCGTCCCGGACCGTCACAAGGAAAACGAAAACCAAGATTTGCCTTATCCGGTATCAAACGAGAGCTACGAGCAAATAGAACACCCTTCAGAAGTATCAGTACCGTCACATGAATTGTAAATGCATGAATGTGGTGGACCAAAAAATCCGCGGTCCCTAACGGAATAGGTAACAAAGCGACCTTGCCACCCACTGCTA------CTAAATCACCACCCCCCCAAGTCAAACTGGTGCTTGTTGTTGCACCAGGAGCCGTTGCACTAGGCGCTAAAGCATGGGTGTTTTGTATCCATTGAGCAAAGATGGGTTGTAATTGTATAGCGGTATCTGAAAACATATCTTGAGGACGTCCTAAAGCGCTCATGGTATCATTATGAATATACAAACCAAAACTGTGAAAGCCTAGAAATATACATGCCCAATTGAGATGTGATATGATTGCATCGCGATGTCTAAGGATACGATCTAATAGATCATTGTATCGAGTAGTTGGATCATAGTCTCTTACCATAAAAATGGCTGCGTGCGCAGCAGCACCAACTATGAGAAATCCACCAATCCACATGTGATGTGTAAACAATGACAGTTGAGTACCATAGTCAGTAGCTAGGTATGGATAAGGGGGCATGGAATACATATGGTGAGCTACAACAATGGTTAAAGACCCTAACATAGCTAGGTTAAGGGCTAGTTGAGCATGCCATGACGTTGTTAGGATCTCATATAGGCCCTTATGCCCCTGGCCTGTAAATGGACCTTTATGAGCCTCTAAAATATCTTTAAGGCCATGACCGATACCCCAATTGGTCCTATACATGTGACCCGCTATCAGGAAAAGAATTGCAATAGCTAAATGATGGTGTGCAATATCGCTCAGCCATAAACCCCCAGTTACTGGGTCTAATCCTCCACGAAAAGTAAGAAATTCTGCGTATTTTGACCAATTAAGGGTAAAAAACGGGGTTGCTCCCTCGGCAAAACTGGGATAAAGTTGAGCCAAAAGATCCCGATTCAAGATAAATTCATGAGGAAGTGGTATCTCTTTAGGATCGACTCCAGCATTTAGAAATTGGTTAATCGGCAAAGATACGTGTACTTGATGCCCCGCCCAAGAAAGAGACCCAAGTCCTAGTAGCCCTGCTAAATGGTGATTCAACATAGATTCCACATCTTGAAACCAAGCCAGTTTTGGAGCTGCTTTGTGATAATGGAACCAACCGGCAAAAAGCATTAACGCCGCAAAGACCAATGCACCAATTGCGGTACAATAGAGTTGTAATTCACTAGTTATTCCAGAAGCTCGCCAAATATGAAAAAATCCAGAGGTTATTTGGATTCCTCGGAAACCTCCGCCTACATCACCATTCAATATTTCTTGGCCCACTATTGGCCAAACCACTTGGGCACTAGGTCCAATGTGAGTAGGATCGCTTAGCCACGCTTCATAGTTGGAAAAACGAGCACCATGGAAATACATGCCACTCAGCCAAAGAAAGATGATGGAAAGTTGACCGAAATGGGCACTAAATATTTTTCGAGAGATCTCCTCCAAATCACTGGTATGGCTATCGAAATCGTGAGCATCAGCATGTAGATTCCAGATCCAAGTGGTAGTTTCAGGGCCCTTAGCTATTGTCCTTGAAAAATGACCTGGTCTGGCCCATTCCTCAAATGAGGTTTTTACGGGGTCCCTATCTACCAAAATTTTGACTTCTGGTTCCGGCGAACGAATAATCATTGAGTCCTCCTCTTTCCGGACACGACATACAAAGAGACCTGCCAAGCGTCAAGTAAT---TAATGAACCTCTGAGAGATATTTC--AAATTAGTTTC-------TTTATCTTCTAT------TTCCCATCTCTCTAGTT--TCTTTAGTTATTCACTAGAACAAATATGATCGGGAAGTCGATC-TAAGGCAAGTGTTCGGATCTATTATGACATAGCCGGTAGGCGCTCAACGGACCC--TTTTTC-TTATTATAAACCCTTTCCGAGCTTTGGATTGATGTAAAAAAC--GA-TTTTTT----------------TATTCCTATCT-----AAATTATAAGGTCTTAGACAGAACTAT-----TTAA----------TGTTT-----TACAGAGATTCTAGTAATTCATATTACTTT-----ATTCCAA-ATC--------ATGCGAGCAGTCATTAGTGATTACTAATGAAACATCCCAGTA-----TCTTTAGGTATTTGA----AGGTA-TTTTAAA-----TATTATTATTATTA-----------------------------------------------------------------------------------TATAA-ATTTATATAT-------------------------------------------------------------------------------------------------------------------------------------------------------------------------------------------------------------------------------------------------------------------------------------------TTTATTTT-----------------------TATTATATA-TATATAA-TTAAAAATAAATATAAAAAACTAAAACTTCAAGTTT------------------------------TTCAAG---------------TTAAAGTA--TTCATTAT------------------TATTATTATTATTAT-----------------------------------------------------------------------------------------------------------------------TTTT----------TTAATAGCT----------------AAAAA------------------AGAGGTGAGGTATTAATTGCTCATAATCGA-----AAAGAAAGAAATAGATT-------CTGTCCTCTATCTGTTTATTC--TTT-AATCACTTTAACCAT----ATGAAATACCCGACAAAATAGAACGATC-----TGAGAA---GGGATATAATGAAATTCTTTGATTGGTTCTTCCCGTAGGAATGATTCT-A-------------TTTTATTTGACTC------ATAGGT--AG----------------AACATCAATTAATTCTAACAATAACAA---------A-----TAGAATAT-AAAAA-AATTA-ATAAT-------------------TTTTTATTCGAAACGCCTCGTGATCTTCAACCAATTATGTGCTTCAATATAATTACCAGGAGTAAGCGCTATAGCTTGTTTCCAATACTCAGCGGCTTGATCGAACCAAGCCTCCGCAATTTCAGAATCTCCCTGTCGAATGGCCTGCTCTCCCCGG------------------TCGGAATAGG-----GGACTCCTTCCCTTAGAACCGTACTTGAGAGTTTCCTAACTCATACGGCTCAACAGTC-----AATTCTTTTGGTATCCG-------TTTTACCTACCGAACGGAATGAGATTTCGCATAGATCTATCTCGCTTTTCGGTTTCG-GGGGTTTAACCAAAAGAAGTTAATCGCATGAGTTTCAAACTTGAATTTTAATTTTG----------------------AATTCGTT-----------------------TTTGTTTTATCTTTTA-TCCCACCTTCAAACGAAT----AAAG--------------------GATGGACATTTCCTCTTTTCGTTAACATTTTCTGCAAGGTAACTATCTCGGTTTCATATCCAA--------------------------ATT----TATATAGAATTCTTGAAAAAGGCTTTCTTT----------CCTGCATAAGAAAGAAAAA-------CTTACTATCTTTGGGATCTGATCCTACACCGCTGCTCAATACCTTAGTGGATCGCCTCTATTACATAAGCAGATTACTCAC---TTTTATCTATCTTCTATTATGT----------------------------------AGG-GCATAAGTAAGCAGTTCT-----------TAATGTATT-GGC-----GCAAACCTC--------------------GTTAATTGATCTTTACGGTGCT--TCTTCT-CTATCAATTCGA-TTTTTTTATCCATAGAATAAAGTATCTAG-GCATATCTT-ATTTCTTCATATTTTCGA--------------------------------------------CTCATATGAAGTTT-------CGTTCCTTGCTACAGCTC-ATAAAAATCGTTGTTTT-TGACGATG-CATATGTAGAGAGCCTTTTTT-----------ATTTTTTTTTTTACTAGAAGA-TTTTTTTGGTCT----TTCCTTTTT---------TCTTTCTATAGTGGAGATAGTCGCACGTAATGACAGATCACGGCCATATTATTAAACGCTTGTGGTAAGAATGGGTTTCGTTCTAGTGCCCTAAAATAATATTCTAAAGCTTTCGTATGATCCCCATTACTTGTGTGAATAAGGCCTATGTTATAGAGTATATAACTTCGATCGTAGGGATCAATTTCTAGTCGCATA----GCTTCATAATAATTCTGTAA-------------AGCTTCTGCATAATTTCCTTCGGATTGAGCTGACATCCGTTACGGTCGTCATTCGATTAAAA-------GAATCTCCGTTCCAGAACCGTACGTGAGATTTTCATCTCATA----------------CGGCTCCTCCTTT-----------------------------ATATGCATAATGATAATATTTCAATCATT---------------------------TTTGATTCCATGTATCGAT----------------------------TATTTTCATTATGAATTGAGCGGGGCTAGT--------GTTTTTGCACGAAA-----TTTCTAGCCAACCTTCC-TGCGCGG--------GAGCTTT--------------------------------------TGTTAACATCA-AACGTGTTGGTACTAGATAGAAATGGCAACTC-------CAACAATTTCTTTGTCCT------CAACGCCCCCTCATTTCCAGGAATTAGTCACTTCAACAGTCTTTGATGGTTATATGGGTATCCAAGGTACGAACGAGATGGATATTTGTTGTCCCAACCATTCTT-----TTAAGTCCCAATCCAGATAAGG---A-A-GGGGGTAAGTAATTTTT--AACAAAGCTTTCGTC-----TTGTTGATTTCTAGATGTAATGCTT-TTCCCCTATGCTGC-----CTAT-TAGGACTAGTAGAGTGGGATTGAC--------CTGTAATACAGAACCG-----ATAGGTGTAACCTTTCGCTCAATGCTAAAATGGA--------------------TAATGGAAGCATA---------TGAGGCTGCATTAATCGGGGAT--ACACGACAGAAGGAATTGTTCTAT---------TTCTAAACTTCACCTTCAACAAGCGTAGATTTT--------TTTCAATAATCG-----ATCAAAATAAT----A---ATAATC-TTTT-TTCTTTCTATCCCGAATTTTTTGTC-----TT---------TCTCAT------AAGACTGGGGG---------------AAAAAAAGAATCAAATCACACCATCTCTGTAATAGGTA----AATGCCTCCTTTTCGCCTGAAGTTGTTGGAATTATTCG---TAATAAAATATTGGCCACAACCGAAAAGGTCTTATCAATAAAAT----------TTCCATTT----ATCCGTGATCTAGGCATAGGTAACCAATCCATTCTAAA--ATTCTTTTCATTCTCCCTA--GGGGGAAAATAATCCTAC-AAAGAAAGGAATTGTACAATACGAAATAGC-----ATAAAAAA----GATTCATTAAAAAAAAAAGAAAT--------------------------------------TA----AATAT-------------------------------TTATC-----TCAAA--------------------------TAAAATGTAAAGATA-----CGAACC------------TTCT---------------ACTC-----C-TACT----CAAATTCAAAGACTCAAATTGC---------TCCTTTTTT-----TT---------------TAGGAATCAAAAATAAATAGTTGAATACGATTCC-----AA----------------------------------------------------------------------------------------------------------------------------------------------------------------------------------------------------------------------------------------------------------------------------------------------------------------------------------------------------------------------------------------------------------------------------------------------------------------------------------------------------------------------------------------------------------------------------------------------------------------------------------------------------------------------------------------------------------------------------------------------------------------------------------------------------------------------------------------------------------------------------------------------------TTTGCA-----AATACTAAAAAAAT----------ATCTTTTTAGTTTAGCCTTT-CACAA------TAAAA-AC-TTTTTTATTTGAATT-----CCCGAGCCTTGAATTTT----------------TAATTGAAGTAAAGATCCTT-----ATCAAAAATGGG--ATA-TTT---------------------TTTTAGTT----------------CGAATTGGTTGGTTGG-------A-CCTT---------------------ACCTA-GCCAA--------------TCC------------AAACAAA---------AATA---TGAATAACTCGCTATTCATTCTGTTA--------CTGGGTCATAA---------TTGTTGTGTAGGA------------GAGGTGGCCGAGTGGTTCAAGGCGTAGCATTGGAACTGCTATGTAG-------ACTTTTGTTTACCGAGGG--------TTCGAATCCCTCTCTTTCCGTACCTTCACCCAA------------------------------------------------------CTCACCAACATCGCTAACCGT-----------AACAAATCAACCAA-GAGGTAGATCCTTCTTTCT------------ATATTTATATATATATTCAT----------------------------------------------------------------------TATA------------T-----ATATTCATATAGATCTATAT------TCTAG----ATA---T----------------------AGATAGATTTTC---------TATTTCTAATTT-AATTG---CTGCGAT-------ATGTA-------------------AAATT----------------ATGGAAT----------AAGGTCGAAAAAAAAGAAAAA-GATCTCTGTCGATCTATGATACATGAAG------------------------------GGGAAAAATCCGGATCAAACCCTTT-C-----CCTTAAT---------TTTA---------AATCAA-TTTTTTTGGCGAA-------AGGGG----GCTCA------------TTTTTTCGAAA-CTTTTTT------------------------CTTTAAG-GTAGGCTTAAGTCTGACGGGAATAAT----------------ATTCTACGACTAGCAATTCGTTTATTTTCAAACCGACCCACTTATTATCTATTATTTGATTGACTACTCCTTTATATGGGAATGGGTGAAGAGTCAAATGTTTTGGCAATTCCTCGCTGTGGGATGAATCTAGATAATTTTGAACGAGAGCTTTAGATTTTTGCTTATCCCTCGCCATAACAATATCGTTGGGTTTGCAAC---GATAACTTGGTATATCCACTATACGACCATTAACTAAAATATGTCTATGATTAACTAATTGGCGGGCTCCCGGAATA----GTCGGAGACATACCCAACCGAAAAAGGATGTTGTCCAAACGCATTTCAAGTAATTGGAGTAAAACCTGACCTGTTGACCCTTTGGCTTTTCTAGCGATACGAAAGTATTTAAGTAATTGTCGTTCTGTAATA--CCATAATGAAAACGTAATTTTTGTTTTTCTTCTAGACGAATACGATATTGAGATCTTTTCCCGGAACGTGATGGGTTTCTAAGAT-----------CTCTAGGCTTTTTATTAGTTAGTCCTGGTAAAACCCCCAGACGTCGTATTTTTTTGAAACGAGGCCCTCGGTAACGCGACATAAAGACTCCTTA-TTTATTGTT-----ATTGATATTTCA-TTTTA-----------------------------------------------TTTAAATTAAA----GAAATTAAAACTGAACT--------AA-----ATGGTAAATGAAGCGAAATCCCCGGAAGTATTCTATT-----------AA-----TTGTACTAGAACGAATAATGGCACTAGAAGGAATAGTGAG------ATGAAAGATGTACGGATCCGAAGTTCCTCC------TTC----------------------TTTTTGTATTAA-------TATTCATTC-------------TTTTTTTATTTGAAA--------TGAAATTTCGTTTAGTATT------------------------------------------------------------------------TTTATTT------------------TTATAATTA-TT--GATTGG-----------------------AATTTTATATTG--------------------------------------------------------------------------------------------TATTTAGTA------------------TATTAAT-AATTATTAA-------TT------GAA-----------T-----------TAT----TGTATATGTAT--------------AAATTCTTGTATATAT-----------------------------------TTATTTTTAGTTTAGTTAAT---------------------------------ATTTCGAATTTTTGT------TGTATATTTATTTAT-----------------------------------------------------------------------------------------------------------------------------------------------------------------------------------------------------------------------------------------------------------------------------ATTT-----ATATATTTAGATTATATAG--------------------------------------------------------------------------------------------------------------AATCTAAA--AAAAAA---------------------------------------------------------------------------------------------------------------------------------------------------------------------------------------------------------------------------------------------------------------------------------------------------------------------------------TCGAAAATAAAGAATA-------------------------------------------------------------------------------------------------------------------------GAAAAAGGAAAGGT--------------------------GTCTTTTTTCTTTAATTTCAAAGAAACGTTCT-------CAATCATA--------------------TAAAAAA---TAGAAC----ATAG---------AAA-AAAGCCGGCTATCGGAGTCGAACCGATGACCATCGCATTACAAATGCGATGCTCTAACCTCTGAGCTAAGCGGGCTC-----------ACATAAAATAAACT------------TTACACGCATA--ATACTT-CCATCAATTAT----ATCTTAGCTATTAACCAT-------------------TCATAAATAATAAAAAA--------------TATAGAATATAAATAGATTTCAAATC------------------------------------------------------------------------------------------------------------------------------TATATTGC------------AGTAAATTAA------------TTTTACTATTTTAATA------TAGAGTATGTA------TATAAATAAGA-----------TAAAGATTACTA-----------TACCGATCTAT-----AATT------------------TATATTTA-----------TTA------CATTCCAATT------------------CTAACAATTAGAATAATATC-TTCTATTTCTCCTTTTTTATC--------------AAAAATTTTTAATTAGAT----------TTTTAG------------ATTTAGATAGAATTTTAGATCAAATTATATAATTTAT----------------------------------------------------------------------------------------------------------------------------------------------------------------------------------------------------------------------------------------------------------------------------------------------------------------------------------------------------------------------------------------------------------------------------------------------------------------------------------------------------------------------------------------------------------------------------------------------------TAAGAACTTAATC--------------TTAAGTTGAAGATTTTATA-----------------TTTTAGATATCTTA-----T-------ATTTTAGATATATTA-TAAAGGTCTACCCCTAAAAAAAGCA-------------TAAAAAAATGGAATAGGCCTATA---TATAATAATAGAAATAAATATAATAATAGAAATAAAATA-------TAAA------------TATATAA-------------------AAGATAAAGAAAGATAAAGATG----CAATTCAGATCAGAATAAGATA---------------TTCCTATGCTTT-------------------------AATTTGAAAACTAAG-----------AAAAAAATCGATCCTTTGAGTATTCCAACTTTCATGGG-AA-AATGAAAAGAAAGGTTCA--TATATAG-AGTGATAGATATCTGTCTATATTGAATTGAAG--ATAAAAAAATG-ATAG------AATTATTTCTGATTGGCCTATAGC-AATATGAGCTCCT----------ACTAGAAATGAAAGAAAATAGA---AAAAAAAT------AGGATGAAATGCCTTTCGGTATATT-A---------ATTTT----------TAT---------------------------------------ATAAT---------AATGAATTCAACGGTTC---C-----AAACGAAAGG------------------------------------ATAA-------------------------------------------------------AA-GGGGGGATATGGCGAAATCGGTAGACGCTACGGACTTAATTGGTTTGAGCCTTAGTATGGAAACCTACT-AAGTGAGAACTTTCAAATTCAGAGAAACCCTGGAATAAA-AAAAATGGGCAATCCTGAGCCAACTCCTTC------TTTAA-------------------AAAAGGAAGAAT-AAAAAAGGATAGGTGCAGAGACTCAATGGAAGCTGTTCTAACAA----------ATGGAGTTGACGATCTTGCGTTA--------TTATAAAAATTCTTCCATCGAAACTCCAAAAAAGATGAAGAATAAACCT------ATATGCAGACGTACTTAA---------ATAT-AAATAATATAT---ATAAT-------------AACTATATATAATAAC--TAGTTATATATATAATAACTATATAT---------------AATAACTAT----------------------------------------------------------------------ATATAATATA---TTATATAAAAAAATTAAATTAAAAAATCTT-------------------------------------------------GATGACGACCCGCATTT------TTTTT--ATGACT------ATG-AAAAAATGGAAGAATTGTTGTGAAGCGA-TTCCAAGTTGAAGAAAGAGTCGAATATGCG------------TTTAATA------AATTAAAGCATTTATTCCCC--GGTCTGAGAGATCTTT---TGAAGAACCGAT--------CCATCGGATGAGAATGAAGATAGAGTCCCATTCTACGTGTCAATCCTGACAACAATGAAATTTATAGTAAGAGGAAAATCCGTCGACTTTATAAATCGTGAGGGTTCAAGTCCCTCTA--------TCCCCAAAA-------------------AATATAAAAAGATCATTTGATTCTCTAACTAATTATCCTC--------TTTGTTTGTTAACGGTTCAAATAAAAATTGGGTCTCTTACTCATTTACTCTTCTTTCAC-AAAGGCATCCGAGCAGAAAAGTTTTTC----------------TATTATCACAAGTC--------TTGTGATGTA-----------TGATGTAAATGATACA--------TGAGCAGCTTTGAGCAAG----GAGTACTCTTACTCATT------TGAATGATTCCCAATACATATCATTACTCTTAC-TAAGA----------ACATACAAAGTCTT-C------CTTTCAAGATACAGGAAATTCCGGGGCCTAGATAAGAC---TTTGTCATACCCTTT-CGC-C----TTTTTAATTGACATAGACCCCTG-TTTTCTAG-----TAAAATGAGTAGATGATGCGTAGGGAA-----------TGGTCGGGATAGCTCAGTTGGTAGAGCAGAGGACTGAAAATCCTCGTGTCACCAGTTCAAATCTGGTTCC-----TGGCACATGATTCATTTGGT------TGAGTATCTATTTTACAAATT--------AATTGAAAATTAATAAATATGGATCCAT--------ATTCATTAAT-----------CGTATAG--CCCATG-CATAGACGTAA---T-------------TTTTTTCT-----AGGGTCTAGAGATATACG--------------------CCACC------------------TAT-AAAATAGATGGGTAAAG-------AGTATATAAAAAAG--------ATCTAA----------------------------------------------------------------------------------------------------------TCATTGAAAAAGA---ATATTCCTTCTTC---------GTGTGGATTCGAAAAA----------------------GGATTAAATTTAG------------TTAAATTAGTTGCAAG--------ACGAAAAAGGGGGG---------AGTTAAAGTAAAGGATGAGAATAGACA------------AAATGTATCTCAGA-TACAATAC----AAGTAGAATCCCAC-CTCCTTTCAT------TTC-T-TTTTTTCTATTTC-----------------------TTCATTT-----------------------------------------------------------------------------------------------------------CCCCCCTA-------CATTCCGTGACTTTCTACAGACCAGCTAAGTGATGCTCG-----ATGTTCGCGGTACAAAGTTCATGATAT-GAAAATTTTT-------TTCTATT-----GGC-TCGGCTAATAAAAA-ATCAAAGTATCTTCCCAACTTTAAAATTTCC------AATGAATTCGTAATTCGATTT-------TTTT--------------------------------------------------------------------------------------------TTAACCAACCATACTCCG----AAAAAAA-TGTCAATTACTCCGATTGTTT---------GTTATAGAACAGAGTGTACAAATATT----------------------------CCTTATACTTA-------TAATATA------------------------------------------GATTTGTTTG-ATTTGAAATTGTTGAAGTGAAGATAAGTTTCTTATAATTCAAAAAGCATCTTGTATTTCATAAAAATTGGGGGCAATATAATCTTTACGTAAAGGCCAT----------CCTATCCAACTTTCGGGCATTAAAATACGTTTCGGGCGTGGATGATTATCATAAAAGATTCCCAACATATCATAGGATTCCCGTTCTTGAAAATCCACACTTT----TCCAAACCCAGAAAACAGACGGAATTCGAGGGTTCCTCCTTGGGGCAAATACTTTTATGCATACCTCTTCTGGCTGATCCACACCGGATTCTATTCT--------CGTAAGATGAT-----------------ACACGCTAGCTAACAGCCCGCCCGGTTCGACATCA----TAAGCACATTGGGAACGTAGATAATTGTAACCATAGACATATAAAATGACAGCAATGGAATGCCAATCTTCGGGCTTTATTTGTAAAGTCTCTATTCCTTGGTAATCGAAGCCTAAAGATCTATGAACTAGC-CCGCGTTTGA---CTAGCCAAGCAGACAAACGACCCTGCATCTTGTTTATTTCTCCCACATTTTTATTTG-------TAT-------------------------------AAATATTTAT-------------TTTT----ATAAGTATTT---------CCTGAAATTTGTAAACACTGGA--------CCCCC--------------TTTATTC---TGT-------AAAAAAAAGGAATCCCGCTT--------------------AATTCACTAATTCGTGGGAA------------------------------------------------GATACTGAATTTTTGTA---------TTTGAAAAAT------TTTTCAGGAGGGATCTCGGAAATAGATGGAGATTTAGAAAGTAATCCTTGATTATAATTTCCAGTATGAATACGGCGTCCAACACGAAACTTGTGTTTGATAGTAAAACATCGATTCTTCCGTTGCGACTTAATTCTATCTTCATAGATTTCCCAA------------GATATTTTCTTACGAAGCTTTGTTATAGCATCTATAACTGCTTCCGGTTTAGGTGGACAGCCTGGCAAATAGATATCCACAGGAATTAGTTTATCGACCCCCCGAACAGTACTATAAGAATCGGTACTGAACATCCCCCCTGTAATTGTACAGGCTCCCATAGCAATAACATACTTTGGTTCAGGCATTTGCTCATATAATCTCACTAAAGAAGGGGCCATTTTCATTGTTACTGTACCAGCTGTTAAAATGAGGTCTGCTTGTCTAGGGCTCGATCTTGGTACCAGTCCATAA------------CGATCAAAGTCGAATCGCGAGCCTATTAATGAAGC------------------------------AAATTCAATGAAGCAACAAC-----------------------TGGTACCATAAAGAAGCGGCCATAAACTAGAGAGTCTTG----ACCAATTTGAAAGATCATTTGATGTAGTTGAAATAACTGAGTTTGGGGTTATTCGATCAAGTAGAGTA------AACTCAACGGAATTCATAACTC---------------TTTCA---------ATTTCA---ATCTTATTG------TTTTTATTGTTAGAATATTCAGAAACTAAGAC-CATTCCAATGCT-------------------CCTTTTCGCCACGCATAAACTAAACCAACAATTAGAATAAGCACGAAAATGAAAGCTTCTATAAAAACAGATACACCTAATACATCGAAACTCATTGCCCATGGATAAAGAAAAACCGTTTCAACATCAAAAACAACAAAAACTAAAGCAAACATATAATAACGGATTCGAAATTGTAACCAAGCATCACCAATGGGTTCTATACCTGATTCATAACTAGAAAGTTTCTCTGGTTCTTTTCTAATGGGAGCTAAAATTCCGGAAAGTAGAAATGCCAAAATCGGAATAACACTTGATATTATTAGAAATGCCCAGAAAATATCATATTCATAAAGCAGAAACATAAACGCACTCCTATGAATGTGGAAAATATAACGAATTAGG-CGATTCGACTTGGAATTGCCAAGTC---------ATCCATAACTGT-----------------------------------------------------------TTAGTCAAAACAACAATTCAT------T---TTGCCCGAACTA-------CCCAGTTTCGTT------TGTTTACCGCGAGACATGTATCCTTTCCTTTCACGAGTCATTGGTTGGAATCTTATTTCC-GTTTTCATTACACCTAACTTACTT-----------------------AGT---------------------------------------TATATA---------------TATTTCAACTAAAAT-----------------------------------AA--CA------AA-TAAGTAA------------AAGAAT-----------------AATATGCTCT-----TATCTTATC----TTATATTAT-------------------------------------------ACATGTACATG---------------TAT-----AAA-----------------------ATACATAT--------------AA-----------------------AAATA-AAACGTTCTCGCTTTCACCTAACTTCCT------------ATATTTCTCTAAAT--------------------------------------------------------------------------AACAAGGG------------------------GGAAAT-----------------------------------------------------------------------------------------------------------------------------------------------------------------------------------------------------------------------------------------------------------------------------------------------------------------------------------------------------------------------------------------------------------------------------------------------------------------------------------------------------------------------------------------------------------------------------------------------------------------------------------------------------------------------------------------------------------------------------------------------------------------------------------------------------------------------------------------------------------------------------------------------------------------------------------------------------------------------------------------------------------------------------------------------------------------------------------------------------------------------------------------------------------------------------------------------------------------------------------------------------------------------------------------------------------------------------------------------------------------------------------------------------------------------------------------------------------------------------------------------------------------------------------------------------------------------------------------------------------------------------------------------------------------------------------------------------------------------------------------------------------------------------------------------------------------------------------------------------------------------------------------TACAATTTTC-----------------------------------ATCTA------GAAA------------ATGATAGGGCTATACGGACTCGAACC-------------------GTAGACCTGCTCGGTAAAACAGCTCAAACT---------------------------------TTATTATTATTATCAAACTGATTCGAACTGTTTCAAAGACCCAACATGCA----------------TTTTTTTTTT---------GCATTGGGCTCTTTCATTAA-----CTGA---TATATATATATTACC----------------TGATATAA-ATATCAGTTAGTCTACCATATTTTTTCTTG-ACAGA-----AAGCGGATGGTTCCATGTGCTCGGATTCATTATTTGGACTCTGATCCAGGAGCACTACCAAAGTGTTTCAAAGAA--------GGGTTATCTTGACGTAGGTCTGCCTTTGGCCTAGATTTTT-----------------------------------------------------TTAAT-GAAATGGAGTCTCTA-------------TCGCTCTGTTTAAAGAA-------------------------------TCAAATATGAAACTTCATACACCTTAAAGTTCATAGGACGAAAAGAGA-TTTTT-GAGGTCCTTATACTTC-----------ATTCTGCC--TAGCATTGAATAGGCTGGTTATTCACCCTATCACTTACTATC-----TCAAATCAAAAAGGCGTTC------------------------------------TTAAATGAACACCCGAATTGGGTTGAACTAAT-----------------------------------TGTCAGGCTATTGTTCTCTTGTTGCCGCAAAGT------------------CATAGAGTAAGACATCGATTTGTCA-----ACCAAG-TCTTTTGATTGCATGGTGAACTCCCCTGAAAAACATTGGCGCACGTGTAAACGAGGTGCTCTACC-AACTGAGCTATAGCCC-TTGCTTTTGTGTTAC-----AT-------------------------------------------ATTTTAGCACTTAG-----------------AAAATTTTTTGTCAA------GACGAGGATTCCATGATCCAATATCGTAGCTTTTTGGTCTGTTTGAT-----TCATA-TTGCTTAGAAGTAAGATTACATTTATA-------------ATCTATGTGATGGGGTTTCTATTTGTTTCTCTT----------TGTGA-TGATAAACGACCTACTTAACTCAGCGGTT-------------AGAGTATTGCTTTCATACGG------------CAGGAGTCATTGGTTCAAATCCAATAGTAGGTAGA-------GCTTATTAGATACCA---------TCGACTCTGGTATCTAATAAGTTTTTCTACT-CACCCCTATCT-----TTA----TCTTATAATCTTAT--------------------------------------------------------------------------------------------------------------------------------------------------------------------------------------------------------------------------------------------------------------------------------------------------------------------------------------------------------------------------------------------------------------------------AAAAAAAGAATGAATATGAA----------------------------TTTTTATCT----TTTTT-------------------------------TTTATTTCATTTCAATCTCATTTT----------TTCCTTACATTAG-ATTTCATTATGCTTAATTGTAGTTTTATTCAATCAGC----------------------AAAAAAAGAT----------------AAAAAAAATAGGATTCT-------ATTTCTAAACAAAACAT-CCTTTTT-------TTAGTATGC---------TTATGCGGACATACCGACTCGTTACGAAATC-----ATATTGATAGCCTCGACTCGTGTCCTAGCTCGTCTGAGAGCTAGA---------------TTTGCCTCAATTGTTTGTCTCTTTC-----------CTTCTGCTTTCCTCAAATTAGCTTCTGCTATTTCAAGAGTTTGCTGAG--------CTTCTTGTGGATCAATGTCACTACCCCTCTCAGCATCGTTTACTAAAACTGTG---ATCTCATTAT----------TGCCTATTCGAGCAAAACCGCCCATCAGAGCCATCGTTAACCATTGGTCGTTAA---GTCGTATTCTTAATATACCTATATCTACAGCAGTGG--------CAATCGGAGCGTGGTTTGGTAATACGCCGATTTGTCCACTATTA-GTAGATAAAATGATTTCTTTTACTTCGGAATCCCAAACAATTCGATTAGGAGTCAGTA---------------CACAAAGATTTAAGGTCATTTC---TTCAATTTGTTCTCCATTTCAAAGTTCATAGC--------------TTTCGCGGTAGCTTCGTCGATGTTACCTACCAAATAAAAGGCCTGCTCGGGAAGCCCGTCTAATTCTCCGGAAAGGATCAATTGAAACCCTCTAATTGTTTCTGCTAGACCGACATATTTTCCTGGCGAACCCGTAAAGACTTCGGCAACGAAAAAAGGTTGTGATAAGAAACGCTCAATTTTTCGTGCTCTTGCTACGGTTAAACGATCTTCTTCGGATAATTCGTCTAACCCAAGGATAGCTATAATGTCCTGAAGTTCTTTGTAACGTTGTAAAGT-----TTGCTTAACTCTTTGCGCAATTTTATAATGTGCCTCGCCAACGATTCGAGGTTGGAGCATAGTTGACGTTGAATCTAAAGGATCTACTGCCGGATAGATACCTTTGGCGGCTAATGCTCTTGATAGTACAGTAGTTGCATCTAAATGTGCAAATGTTGTGGCAGGAGCGGGGTCAGTCAAATCGTCTGCAGGTACATAAACTGCTTGAATCGAAGTTATGGACCCTTGTTTGGTAGAAGTAATTCTTTCTTGTAAAGAGCCCATTTCGGTACTAAGAGTAGGTTGATAACCCACAGCTGACGGCATTCTACCCAATAAGGCAGATACTTCAGATCCTGCTTGGACGAAACGGAAGATATTGTCAATAAAGAGAAGTACGTCTTGTTTATTAACATCTCGAAAATATTCCGCCATAGTTAGGGCTGTCAAGCC----AACTCTCATACGAGCGCCGGGCGGTTCATTCATTTGCCCGTAGACTAGAGCCACTTTTGATTCTGGAATATTTTCTTCGTTAATAACTCCGGATTCTTTCATTTCCATGTAAAGATCATTTCCTTCACGAGTACGTTCACCTACTCCGCCAAATACAGATACACCTCCATGAGCTTTTGCAATATTGTTGATTAATTCCATAATGA--------------GTACGGTTTTACCCACTCCAGCCCCTCCAAATAGTCCTATTTTTCCTCCACGGCGATAGGGGGCTAAAAGGTCTACCACTTTAATTCCTGTTTCAAAAATAGATAATTTTGTGTCTAACTGTATAAAGGCAGGTGCGGATCTAT-GAATAGGAGATGTTGTTCGAGTATCTACAGGACCTAAATTATCAACGGGCTCCCCAAGTACATTAAAAATTCGTCCGA------------------GAGTTGCTCCGCCCACCGGAACACTTAAAGGAGCCCCTGTATCAATCACTTCCATTCCTCGCGTTAGGCCGTCTGTAGCACTCATCGCTACAGCCCTAACGCGATTATTTCCTAATAATTGTTGTACCTCACAAGTTACATTAATCGGGTGACCGGAAGTATCTCGGCCCTTAACGACCAGAGCGTTGTAAATATTAGGCATCTTGCCT-GGTGGAAAAGCTACATCCAACACTGGACCAATGATTTGAGCGACACGCCCCAGGTTATTTTTTTCAAGTGTGGAAACCCCAGGACTAGAAGTAGTAGGATTGATTCTCATA------A---------TTATAAA-----------GTAATAAAGT----------------------AAAAAA--AAAATAATATTAAATAAAAT------------------------------------------------------------------GAAATAAATATTATATTT--------------------------------------------------------------------------------------------------------------------------ATATATA-TAATAT-----------------------------------------------------------------------------------------------TATTTT-TTTTGCG-------------AA--AATG----AA------AAAAAAAGAAATGTCCGATAACAAGTTGATCGGTTAATTC-------------------------------------------------------ACTACGA----AATGGGAGTTAGCA----------------CTCTATTTTGTTGGGATCATA---------------------------------------------------CAACCGAATCCAATTCAATTGTTTACTT-A-----TTCCTTCC-----------TTTTTCTATTTCTTT---------GATTCAATTTCAATGAGTGAGTTCAACCA----------------A--TCTATTTTC----AAAAT---AGAAAGTCAATGAACAAAAAT-----------------TTGTAGAAAGTCTTTT---------ATTTGTCTATCATTCTAGAAAATCCC--ATATATATTAGCGGCGGAATTCGAACCTGAACTC-------------------------------TTAATTTATGGTTCATTCTT----TCATTTCTATCGCATCGGC---------------------------TT------------------TTTTTTAGCATATTGATTTAC----GTATACCCTATTC------T-TTTT-------TTT--------TTTT-----TTTATAC-------CCTTTCGTCTACGAATTCTGCATATT---TACACATCTAAG--------ATTTACATATAC-----------------AACATATATTGCTG-------TCAAGAGTGCA-----TTTTTTTATTAATTATT------------------------------TAGTCAATTC-------AAAAAA-----GGTTAAGGC-----ATTAGAAACTTGAAAAAG---------------AAAGGTTGGGTTGCGCCACATATAGGAACGAGTATACAATAATGATGTATTTGACGAATCAAATACTATG-----------------------GTCTAATAACTAACCTTTT-TAATTAG--------------ATTAGTTGATAATTTTATGACA------------------------------------------------GATTCCTGTAAA-------AAAAAGGTTTAATTAACGCC----------TAATTCATGTCGAGTAGACCTTGTTGTTGTGAGAA-----------------------------------------------TTTAGTAATTGATGAGTTGTAGGGAGG---------------------------------GACTTATGTCACCACAAACAGAGACTAAAGCAGGTGTTGGATTCAAAGCTGGTGTTAAAGAATACAAATTGACTTATTATACTCCTGACTATGAACCCCATGACCA-TGATATCTTGGCAGCATTTCGCGTAACTCCTCAACCTGGAGTTCCACCAGAAGAAGCAGGGGCCGCGGTAGCTGCCGAATCTTCTACTGGTACATGGACAACTGTG----------------------TGGACCGATGGACTTACCAGCCTTGATCGTTACAAAGGACG------ATGCTACCACATCGAGCCTGTTC---------CTGGAGA---------------AGAAAATCAATTTATTGCTTATGTAGCTTACCCATT--------AGACCTTTTTGAAGAAGGT-----TCTGTTACTA-ACATGTTTACTTCCATTGTGGGTAATGTATTTGGGTTCAAAGCCCTGCGTGCTCTACGTTTGGAGGATTTGCGAATCCCTCC-----------------TGCTTATACGAAAACTTTCCAAGGCCCGCCTCATGGTATCCAAGTTGAGAGAGATAAATTGAACAAATATGGACGTCCCCTATTGGGATGTACTATTAAACCGAAATTGGGGTTGT-----CCGCTAAGAACTACGGCCGAGCAGTTTATGAATGTCTTCG--------TGGCGGACTTGATTTTACCAAAGATGATGAAAACGTG-------------AACTCC-----------------------CAACCATTTATGCGTTGGAGAGACCGTTTCTTATTCTGTGCCGAAGCTCTTTTTAAAGCACAGTCTGAAACAGGTGAAATCAAAGGACATTACTTGAATGCTACTGCGGGTACATGCGAAGAAATGATAAAAAGGGCTGT-----------------------------------ATTTGCCAGAGAATTGGGAGTTCCTATCGTAATGCATGACTACTTAACAGGGGGATT----------CACTGCAAATACTAGTTTAGCTCATTATTGCAGAGAT---------------AATGGCCTACTTCTTCACATCCACCGTGCAATGCATGCAGTTATTGATAGACAGAAAAATCACGGTATACACTTCCGCGTACTAGCTAAAGCCTTACGTCTGTCTGGTGGAGATCATATTCATTCCGGTACTGTAGTAGGTAAACTTGAAGGGGAAAGGGACATCACTTT----------AGGTTTTGTTGATTTACTACGTGATGACTTTGTTGAAAAAGACCGAAGTCGCGGTATTTATTTCAC----------------------------TCAACCTTGGGTTTCCCTACCTGGTGTTATTCCTGTGGCTTCCGGGGG-------------------------------------------TATTCACGTTTGGCATATGCCCGCTCTAACCGA----------------------GATCTTTGGGGA--------TGATTCTGTACTACAGTTCGGTGGCGGAACTTTAGGCCACCCGTGGGGAAATGCGCCGGGTGCTGTAGCGAATCGAGTAGCTCTAGAAGCCTGTGTACAAGCTCGTAATGAGGGGCGTGATCTTGCTC--GCGAGGGTAACGATATTATTCGTAAAGCTGCCAAATGGAGTCCTGAACTAGCAGCCGCTT--------------GTGAAGTGTGGAAGGAAATCAA---------------------------------------------------------ATTTGAATTCCAAGCAATGGATACTTTGTAATCC-----------------------------------AGTAATTACTGGTCGGTCCCT------------------------------------------TAAATTGAAT--------------------------------------TGTAATTAAACTTGTAT------------TCGGCTCAATCCTTTTACTAATAC-----------------TAAAAGTAAAAAGAT-------TGAGCCGAATACAA-----GTTTTGCATAGATCTT----------------AGATCTACAAGCAAAATCC--------------------------------------TAAATAAAAAATCGAAAACTAAAAAACTCAAAAGTTTCTTTG---------------------------GTTGTGCT-GGATCCACAATTAATCCTATGGATCTCTA-------GGATT-----GGTGTATTCT----------------------------------------------------------------------------------------------------------------------------------------------------------------------------------TATAT----ATATCCCGTAGCTTAGGACCGCGGATAACGAGTCAAGTATAAG------------AGCCCCTT-----CTACCCATCCTGTATATTGTCCTTTTCTTCCA--------------------------------------------------------------------------------------------------------------------------------------------------------------------------------------------------------------------------------------------------------------------------------------------------------------------------------------------------------------------------------------------------------TTTGTTTATATTACAA-TT----------GAAAAAAAAG-TTCTATCATA-------------------------------------------------------------------TATTTATCACCCATT-----TTTTTTTTATAATAAT-------------AATCCGAGTGC----------------TTTTTTTTAGTAAATGAAAATTTC----AAATGACTTT-TCATCGA---------------------------ATGACTATTCATC-----TTTTTTTTCAT-------------------------------GCAAATAGGGGGTAAGAAAACTATATGGAAAAACGGTGGTTTAATTCGATGTTGTCTAAGGAGGAGTTCCAACATAGGTGTGGACTAAGTAAATCAATGGGCAGTCTTGATCCTATTGAAAATAACAGTATCAGTGAAAATACGAGTCTAGATTATACAGAAAAAAACATTCGTAGTTGGAGTAATGGTTCTAGTTACAGTAATTTTGATCTTTTATTCGGTATCAGGGACATTCGAAATTTTATCTCTGATGATACTTTTTTAGTTAGGGATATTAAGGGGGAAACTTATTCCATTTATTTTGAT---------------------ATTGAAAATGATCATTCTTTTTGTAGTTCACTCCAAAAAAAATTTTCGAATTATTGGAATTCTAGTTA---TGGGAATGGATCTAAAAGTGACGATCCCCATTATGACCTTTA---------------------CATGTACGATACTAAATCTAGTTTGAATAATCACATTAATAGTTGTATTGACAGTTAT------------------CTTCATTCTGAAATGC------------------------GTATTGAT------AATTCTG------------------------------------------------------------------------------------------------------------------------------------------------------------------------------------------------------------------------------------------------TTTTAAGTGATAGTGACAATTACATCAATAATTACATTTT------------------------------TGATGAAAGTCAGACT---ACC---------GC------------TAAGACAAATGGTAGGGATAAGAAT---------------------------------CTTGAGGTCACTAAAAAATACAGCAATTTATGGATTCAATGTGAAAATTGTTATGAATTAAATTATAAGAAATTGTTGAAGTCAAAAACGAACATTTGTGATGAATGCGGATATCATTTGAAAATGAGTAGTTCAGAGAGAATCGAACTCTCGATTGATCCAGGTACTTGGGATCCTATGGATGAAGACATGGTCTCAACGGATCCCATTGA---------------------------------------------------------------------------------------------------------------------------------------------------------------------------------------------------------------------------------------------------------------------------------------------------------------------------------------------------------------------------------------------------------------------------------------------------------------------------------------------------------------------------------------------------------------------------------------------------------------------------------------------------------------------------------------------------------------------------------------------------------------------------ATTTCATTCGGA-------------------------------------------------------------------------------------------------------------------------------------------------------------------------------------------------------------------------------------------------------------------------------------------------------------------------------GGAGGAACCCTATAAAGA-------------------TCGTATTCATTCTTATCAAAAAGAGACGGGGTTAACCGAAGCCGTTCAAACAGGTATAGGTCAACTAAATGGCATTCCTGTAGCAATAGGTGTT--ATGGAT-------------------------------------------------------------------------------------------------------------------------------------------------------------------------------------------------------------------------------------------------------------------------------------------------------------------------------------------------------------------------------------------------------------------------------------------------------------------------------------------------------------------------------------------------------------------------------------------------------------------------------------------------------------------------------------------------------------------------------------------------------------------------------------------------------------------------------------------------------------------------------------------------------------------------------------------------------------------------------------------------------------------------------------------------------------------------------------------------------------------------------------------------------------------------------------------------------------------------------------------------------------------------------------------------------------------------------------------------------------------------------------------------------------------------------------------------------------------------------------------------------------------------------------------------------------------------------------------------------------------------------------------------------------------------------------------------------------------------------------------------------TTTAAGTTTATGGGAGGTAGTATGGGATCTGTAGTAGGAGAGAAAATCACTCGTTTGATTGAGTATGCTACTAATAAAAATCTACCCCTTATTATAATATGCGCTTCCGGCGGGGCACGCATGCAAGAAGGAAGTTTGAGCTTGATGCAAATGGCTAAAATTTCGTCGGCTTTATTTGATTATCAATCAAATAAAAAGTTATTTTATGTATCAATTCTTACATCCCCTACTACTGGGGGGGTGACAGCGAGTTTTGGTATGTTGGGAGATATCATTATTTCCGAACCCAACGCCTACATTGCATTTGCGGGTAAAAGAGTAATTGAAGAAACATTGAATACAAAAGTACCTGAGGGTTCACAAGAAGCTGAATATTTATTCGATAAGGGTTTATTTGATCCAATTGTACCACGTAATCCTTTAAAAGGCGTTCTAAGTGAGTTATTTCAGTTGCACGCTTTCTTTCCT-----TTGAATAAAAATTCGATC-------GAACAGTAAGGTCAATTA-----TTTTT-TTTGT-----------------CACAAAAAAGTAGTTAGTTGTCGTAATCAACCAAAGTAAAA-----AT-------------------GATAAAG-------------AAT-----------------------------------------------------------------------------------------CC----AT------TAT----------AATAGAATACT--GGGGATGGGGTTTTCGTGGTTGCAATAC-----------TAATTCTAT--------------------------------------------------------------------------------AACTATA------TAGAATATAAAT------AATCA-AAAGTTGCGGATAAATTG-TTT-----TTT-TTATTTGACTTCATATTCCATTCCTGATTAGTAATCAGAGAGAACCTCTATTCTATA----CAAC------------ATTCTTACTTCTGCTTCTG----TAAATTGAAAATTTGGCAAATAAAACTAATTTTATC-----TTTCTTA------CATATGG-----AAAATTCT--------AAAAAAAAGCC-----TTTTGCATCTTAATATTTTT-CTTG------------TCGGAGAGTCTCCGTTTTGA-------CTAACAAGAGAATAT----------------CTCTTG--------TCGTTAGAATCTTTCGGGACTTTGTAAGCAACTCTTTCTTTA------TTTATATTGAAT--------TAAAAGACAAGAGCAAAAGAAAAATAA-----AAGGTGAAGAATAAAAAAGTTGATTATCAAACATATA------TTTTTTATGGCGACGGCTAATTAAG---------------TTAGTTCTACATTTCTTGAACTTAGTATATACTATACTCACTTAGATAT-----------------AATTAG------TATAATTATATAGAA--------TTAGAATTCTA-A-TTAAGTTAAGATAATTT-----TAGAACAATATAACA--AACAGG-----TACAA-ATAGTAAATCGAGGTA-CCCATTCTATGACAGATATAAACCTTCCCTCTATTTTTGTGCCTTTCGTAGGCCTAGTATTTCCGGCAATTGCAATGGCTTCTTTATTTCTTCATGTTCAAAAAAACAAGATTGTTTAGGCTAGATGGTG---AGGCCAAATCCCA-------TTTTTTCACGACTTAGACTTTATTGAATCATAACACGGATATCTATCTA-TTTA--------------TTTATTGG------AAAGTGGAATATGGTAT------AA------TACATGATTT-------CTTTCG-----AACATAAGTGCAAGA---------CATGCGAAA----CC-------TGATA-TAGGAGTA-AATTCATTTTCAC-TATTTTCAAATC----------------------AATAG------ATCAGGA-CGGGTCAA-GCCATGT-----TTG---------AAATAGAAAGTCAATGCTTGTAG----------ATATCTAAGGCGGGG----------TCATATGAAGGGGACGGTCTT------ATTTTCGATCGAACGGTTTTTATG-----------AATTACCCCGACAGGTTCACATTCGAATAGTTCTAGTTGATGAGAGTTACTTCAGAAACAAAATAGC------------------GTTAA------------GTAAAGTAAAAGTATAAGTGAAATTCATTTTGGTTATTCT-----ATCAATTCAATTAAGT-------------CAAATTAAATGCAACTAGATT-AGTATGAATTGGCGATCAGAACGTATACGGATAGAACTTATAAGGGGGTCTCGAAAAACAAGTAATTTCTGCTGGGCCTTTATCCTTTTTTTAGGTTCATTAGGATTTTTATTAGTTGGAACTTCCAGCTATCTTGGTAGGAATTTGATATATTTCTTTCCCTCTCAACAAATAATTTTTTTCCCACAGGGGATCGTGATGTCGTTCTATGGGATCGCAGGTCTCTTTATTAGCTCCTATTTGTGGTGCACAATTTCGTGGAATGTAGGTAGTGGTTATGATCTATTCGATAAAAAAGAAGGAATAGTGTATATTTTTCGTTGGGGATTTCCGGGAAAAAATCGTCGCATCTCACTCCGATTCCTTATAAATGATATTCAGTCTATCAGAATAGAACTTAAAGAGGGTATTTATCCTCGTCGTGTCCTTTATCTAGAAATCAGAGGCCAGGGGGCCGTTCCTTTGACTCGTACTGATGAGAATTTGACTCCACGAGAAATGGAGCAAAAAGCTGCTGAATTGGCCTATTTCTTGCGCGTACCGATTGAAGTATTTT--GAAATGAACC------------------------GAACAATGAAT------GTTTTCTGATTTTCTGCTGGGGGTAGAAAA----ACTCTACAACCCCCCCTTTTTAATAA--------CTTTTCTGCGGTATAATGTAAC----------TAAAATT-CGTCAAAACGTCCTTT-----CGAG-------TCAAAGCAAACGT-----------ATATT--------------------------ATATGGAACATAAAAAAA------------------------------------------------------------------------------------------------------------------------------------------------------------------------------------------------------------------------------------------------------------------------------------------------------------------------------------------------------------------------------------------------------------------------------------------------------------------------------GGGGGGCTG----TTTTTGTCTGCAAAAAATAT-----ATTTTATGCGTATAGAAATCCACTCG-----ACGCAATTCATTAGC-----------AACAAATAG----AAATAAGGATAGATTCATTCCAAAAC---------------ATTTTGAAATAAATAAATTTTTTTTATTTTCG---------------------TTTCAATATTT-GATTTTGAATTGATAAGTTAGAGACAAATAGCTC-----ATGTAAATTTATTATCTTTCTT------GATGTTTCGTTTTCTCCCCTCTTTCATTTTCTTCT-----CTTTAATGAATGACTCAACATCTTGCTA------------------------------CCCCCCCC---TTT--------------TTTTGACCATCACA------TTCAGAAATTTATCTCAATTCTTTT------------TAT-GGTAGTCAGCGAA---TTTTTTTGCAATAT---------------TTGTAGAGT-TTTTT-GTCTC-GAAATCAGA-------ATTCCTT------------ACCT---------T-AGGTTTTCGGGGATTCATCTAATTCATCTAAAGG-------AAGGAATTGCTTCG-----AATTTGACC-------AAT----TGAAATGGCTGGAAACT----TTTTTTCGCATTTTCACATTCGAA-------GTGGACTCTTATTC----------GATTTCTGTATTCTTGTAAAATTCTTCAAA------ATT-----ATCAAGGACTAA-------TTACCGAATCACAAATAAAAA-----ATAGAGAAT---------GATTCGAT-ACCTTGGAATCGAACTCATTTTGGTGAAAAAT--------------------AAAATATTCGATCGCG----------TAGAGTCGACGAATGAGGCCACTTTAGTA--------------TTAGTAAATT-AACAA-----T-TTC-------------TAAAAAATATGGCAAAAAAGAAAGCATTTATTCCTTTCCTATTTCTTGTATCTACAGTCTTTTTACCCTGGTGGAGCTTTCTAACATTTAAAAAAAGTCTAGAATCTTGGGTTACGAATTGGTGGAATACCAAGCAAGCTGAAACTCTTTTGAATGATATTCCAGAAAAGAGTCTTATAGAAAAATTCCTAGAATTAGAGGAACTCCTGCTGTTGGACGAGCTGATAAAGGAATACCCGGAGACAC------ATCTAAAAAAGCTTCGTATA------GGAATCCATAAAGAAACGATTCAATTGATCAAGCTGCACAATGAAGATTGTATCCATACAATTTTGTCCTTCTCGACCAATATAATCTGTTTTGTTATTCTAAGTGGTTATTCTATTATAGGTAATAAAGAACTTATTACTCTTAACTCTTGGGTTCAGGAATTTCTATATAACCTAAGCGACACAATAAAAGCATTTTTTATTCTTTTATTAACCGATTTATGTATCGGATTCCATTCACCCCATGGTTGGGAACTAATGATTGGCTCTATCTACCAAGATTTTGGATTTTCTTATAACGATCAAATTATATCTGGCCTTGTTTCCACTTTTCCAGTCATTCTAGATACAATTTTGAAATATTTGATCTTCCGTTATTTAAATCGTGTATCCCCATCACTTGTAGTGATTTATCATTCAATGAATGAC----TGAAAA-----------AAGGTCT----------ACTGA-------TAT-------------------------TAATTCAA-TTAAAATTTTTGATACTTTG-----------------------------------------------------------------------------CTCT------TTCTACCCATCCAAGGCAGAAAGGCCCTCCA----ATATTCCAGTAAGATTATTCC-----AGTAAATAGCAGAATCGTGGATAGGGAACTATACTAGCAACCTACCCAATTTATTGTAGAAATTCTCGGGATCAAAAATTGGAATATGCAAACTATAACTACCCTTTCTTGGATAAAAGAACAGATTATTCGATCCATTTCCGTATCACTCATTATATATATAATAACTCAGTCATCCATTTCAAATGCATATCCCATTTTTGCACAGCAGGGTTATGAAAATCCCCGAGAAGCGACCGGTCGTATTGTATGCGCCAATTGTCATTTAGCTAATAAACCCGTGGATATTGAGGTTCCACAAGCGGTTCTTCCTGATACTGTATTTGAAGCAGTTGTTCGAATTCCTTATGATATGCAACTAAAACAAGTTCTTGCTAATGGCAAGAAGGGGGGTTTGAATGTAGGAGCTGTTCTTATTTTACCCGAGGGGTTTGAGTTGGCCCCAACCGATCGTATTTCTCCCGAGATGAAAGAAAAGATAGGCAATCTTTCTTTTCAGAGCTACCGACCGAATAAAAAAAATATTCTTGTGATAGGCCCTGTTCCGGGGCAAAAATATAGTGAAATCACCTTTCCTATTCTTTCACCGGACCCTGTTACTAAGAAAGATGTTCACTTTTTAAAATATCCTATATATGTAGGCGGTAACAGGGGAAGGGGTCAGATTTATCCCGACGGAAGCAAGAGTAACAATACTGTTTATAATGCTACAGCAGCGGGTATAGTAAAGAAAATAATACGAAAAGAAAAGGGCGGATACGAAATAACCATAGGGGATGCCTCGGATGGGCGTCAAGAAGTCGTTGATATTATTCCTCCAGGGCCAGAACTTCTTGTTTCAGAGGGTGAATCTATCAAACTCGATCAACCATTAACGAGCAATCCTAATGTGGGTGGATTTGGTCAGGGAGATACAGAAATAGTACTTCAAGACCCATTACGTGTCCAAGGCCTTTTGTTCTTCTTGGCATCGGTTATTTTAGCACAAATTTTTTTGGTTCTTAAAAAGAAACAGTTCGAGAAGGTTCAATTGTCCGAAATGAATTTCTAG----------------------ATCAAGTTCATAACAAGAACCAAA------TTCTTATTTGTTTAT----------------AGTTGATCACGAAAT--------AAAAAAAGTACAAAAG-CCT-CTTCTT---------------------------------------------------------------------------------------------------------------------TGTTTATA----CTTT--------------------TTT-CAATCCAAA-TTGTAATGATGTGACTA--------TGTAA-CTCTT----------------------------------------------------------------------------------------------------------------------------AGCATAGGAT------ATAACACATAGAAAAA-TGA----GGGGAATAA------ATGAGTCTAGGGGGTATTCTTTTCCTT------ACTAATCTTCGACACAGG--AAAAGGATGTATAAAATTCCCTTTCTTGTGTCAA-TGTAAAATAGTAACG----ATTCGTGATAGCC----------------------------------------------TTCGCT-------GACGCGTCATTTTCAATTT-------TATAGAT----A-----------TGGGGAAGGTTCAGGAGATTTAACCGAAC-TTTTGTTGTCT-----TA-CTATTAC----GC----ATCTAATAGATAGAAC------AAAGTAGTGGACAAAC--AAAAAAGAGAGAGAAT---------TTTATTGAACAACAACTAGAACTTCTTC----AATGAACTTAT-CAAAAGAA-TTAAACTTTG-ATTTG--ATGAAT---------------ACAGATAGACTACTCGAATTT------TTTCTAGTAG-TA-----TCGAAGGGTTTT-CGTGCT------------A-TTTGATCTACCTA----------------------------------------TTTT---------------------------------------------------TGT-----------------ACTTTATT-----------------------------------------------------------------TTATGCGAG-----------------------------AAAATAGTC-----------------------------------------------TAATTATTACGAATT----TAAC-GGG---TACCTCCCCCTTCTTTGTTTCTCTA--ATTCGAGGGGGAAGGAGGGTCCCGT--------------------TGAGTTCTTACGTTTTCCTA-----TCTATACCGCAGTTCATCCGAT----------------TACTAC-------AGGGATGAACCCAATCCGGAATATGAACCATAAAAGAAAATGCCTATTAAACCGATCACAAGAATGCCGGCTACAGTACCTATTATCCAAAGCGGAATCCTTCCAGTAGTATCGGCCATTTACCCCACTTCCCTCCACATTTCATCAAGTGGTCATGCTAGAGACATAAACAGTCATGGGTAATTTTGAGATGAGATCCTTCCGAATGGGATAATAAAATTCCTAC-------TC------------------TATAT---------------------------------ATATAATTCTCTCTCGCTCTTGTTC-------TTTTTAATTGAAAAAATAATTTGAAAATAAAACAGCAAGTACAAAAATGAGTAATAACCCCCAGTAGAGACTGGTACGATTGAATTCAACATTTTGTTCGTTCGGGTTTGATTGTGTCGTAGCTCTATAATTCGGATTAGGTTTTGTTTATCGTTGGATGAATTGCATTGCGGATATTGACCCCAAAAAAGAAACGGTAGGTACAGCTAGTCCGTGAACAGCCAACCATCGCACTGTAAAAATGGGATAGGTTCGATCTATGGTCATT-GGGCCTCCTAAAAGGATCTACTAAATTCATCG-----------------------------------------------------------------------------------------------------------------------------------------------------------------------AGTTGTTCCAAAGAATCAAAACGGCCAGTTATTAATGGAATTCCTTGTCGGCTCTCTGTAAAATACTCGTTTGGCCGAGGGCTTCCAAACACATCGTAAGCTAAACCTGTGCTGACGAATAACCAACCCGCAATGAATAGGGAAGGTATAGTAATGCTATGAATGACCCAGTATCGAATACTGGTAATAATATCCGCAAAAGAACGTTCTCCTGTGCTTCCAGACATGCTCAGCTCCACATATTCTTGTACAGGCAAA-TGTAAATCGATTCCGTAAAAGATGAGATCTGT-AAATGGAAATTTACTGAAAT------TCTTTGTG-GGATCGGCAATATTGTACCAAGGGTGTCTTTAGAGTATACCAAATCAGTATAGCCGTCCTTCTTCTGACACAGCAAGGCAATTTCAATTAATATGGAAAC---------------TAAGTATACGTATTAGAC-----AATTAAATTTCTATTAGACAATTT--ATTTTTTCTTG---------CTTG-------------------TAA-TATAGAACTATGCGCCTTTTAATA-------GAAAAAT--------------ATAGTCTAAA----------------AAT-------ATAGTCTAAAGGTTCTCTTCATTA------TTGGCTTCGGACTAGAGACGG-----------AAATCGGG-TAAGGGATCAATCTGATACGTCGGTTTCTAAGTCTAAAA--------ATTCATCAAA---GATCAAAGAGAG----------------TATTATATTCCCAC------AATTCAATTAGACACGAAATCTCGAACTCTCTTTT-TTGTGGTTTG-----TT-------TATAGAAAGAAAG-GCT-----------------TTTTTTT---CATT--------------TGAAGAAATCGATTAGTTGT-------------CTAGTACCAACTATA------TAGT----------ATTAGGTAGGAGATAGTATTCGAT---G-AAAAAAAAAAAAGAATACAAGAATTG-AATTGTAAGAAATTAATTAATATTCGTGATACATGCATTCCTGTATT------AGATCCAAGGGTTCTTTATTGC-------TCTAACTA-----CAGGGATGAGACTT-------GAT--AAAAAATACGGAAAGAAAAAATGTAGAACCTAA--------------------AATAAAGATAATCAGAAT----GACTCATCTTAAAATC------GATTTG--AACTCAAATCTCAAATAAAAAT-----T---TTA-----TTTTTTTTTGCGTGATCGC-----GTTAATATCC-TTTTTCTTATCAT-----------------------------------------------------------------------------------------------------------------------------------------------------------TAAAAAAAAATAAATGC------TAGAAATGCTTTTCCTGTTT---------------TCTTCTTCGATAGTGAGAT-----------TCTTTTTTAACAAAG---------TTACATGA-----CATAGTTCTTATTTCT---------------------------------------------------------------------------------------------------------------------------------------------------TTTTTTT-TTAGTTTACT-CCAAGAGTTGCTCAAAAAA--TCTGTTGATTAG---------------------------------------------------------------------------------------------------------------------------------------------------------------------------------------------------------------------------------------------------------------------------------------------------------------------------------------------------------------------------AAATCACGGAATTTTTAGATGTAACAGACAATGAGTCGATTTCTTTTTCTACTTCTCTTCTTTAT---ATC----------------------TTTCTT-AGATAT--------------------------------------------ATTTATAAAT------------ATAATGTAATAA------------------------------------------------------------------------------------------------------------------ATAATGTAATAAATATAATGATTGAGGAA----------TAAATTTAACTTATTCT-----TTC-----AATTGGTATTTTTTCTTA-----TTTGCGTTCCTAT---------CTTTCAA--------AAAAAATGTAAACTTAGGTAAGTGCTTTATAAATAT-ATGT-A--A-AAAAAA-------CATATTTGATTTAGCTCCTTCATGCCTACTCTAACTAGTTATTTCGGTTTTCTACTAGCAGCTTTAACTATAACCTCCGCTCTATTTATTGGTCTGAGCAAGATACGGCTTATTTA-------AAAAAAAAATTGAATCAACAATTC-----ATAATCAT------AAAAGAATCTTTCTG------------------------------------ATAGTTCTTTAT-----------------------TTTATCGCGAAAATTTTCAAT----TTTAGG-------TTATTGAGATTCATGGACAAT-----TAGGATTAAAATTTAG----AGATAGATATTACCTCC-----C--CTT------TTTC-----CTTTCAAAAAAATTGAAATGATTGAAGTACTTCTATTTGGAATCGTCTTAGGTTTGATTCCTATTACTTTGGCTGGATTATTCGTAACTGCGTATTTACAATATAGGCGTGGTGATCAGTTGGACCTTTGATTAGTTAACAA-----C-TTTTTTTTATTGA---CCTCCTCTG-----GCTTAAATAAAG------GAGGTCAATT--------TTAGATTCTTC-----TTCAA-----------------------TTATTTCAGTCTAATTAGAACTAACAAGAATGGAATCACGCTCTGTAGGATTTGAACCTACGACATCGGGTTTTGGAGACCCACGTTCTACCGAACTGAACTAAGAGCGCTTTCTT-------ATCACAGACAGTAAAGAA--------------AAAAAGATTCCTTTTGTAACCG--------A-----ATACTACATCTTGCATGCATAT-----CACATA---------TATAT-AAGTATCGAATAT------AGAGTTTG------------------AATTGTATATGTGCTATATGTAT-----ATATTGTATATGTGTTATATGTATA-----TATAGTATTCTATACTACTAGAATA-------------------------------------TGATATATAT--TAATAGTATTCTATAGAATACTATTCTATAGT--------------ATCTATAGTATTC--------------------------------------------------------------------------------------------------------------------------------------------------------------------------------------------------------------------------------------------------------------------TAATATTCGAATACAGTTCTAAAAA-------------TGACAGATTATATATGTCCAATTTGAATCTATTTCGTTGATCT----CAATTAATTCCTCTTTACTTCTCAGAGG-------AAAAGC----AATAGGTAGGGATGACAGGATTTGAACCCG-TGACATTTTGTACCCAAAACAAACGCGCTACCAAGCTGCGCTACATCCC------TTTT------AATAGGTTTACAGTGTTATTGTAAAGAATCCTTTTCTTTTTTTCC----ACACCATTATTT-CTCAGATTTAGATACACA---ATAGA-----TCTTGCCATTTTTT---C--------TTTTTTTTTCATAT-------------------------------------------------AT------------------------------------------GATATAGATAGAATC-----------------TAAA------------------AGTCTTTGGATACATATACGCT------------GTAAACT------------------------------------------------AAAAAGGGCTTTTAGGG------CAGTAGGAAAGA---------------------------------------------------------------------TGTATCTTTTTA----CTTTTT-------TAAACT------------------TAAAGGTAGAAAATCTTATCT----------ACCGG-ATTGTTGTACATTTTTATTTGCTTTAGGAATTTCATGTAC----AAATACAAGTGTTTTTTCCTT------------------------TGCATCTGATCA---------TTTATTA-------------TATATGTA---TTAT--TCTTA--------TGT-------------------------------GTTACAATATATAAATAA-----------------------------------------------AG-AAAAAAAGAAGGAGGATTTTCAATGCGAGATCTAAAAACATATCTCTCCGTGGCACCGGTACTAAGTACTTTATGGTTCGGATCTTTAGCAGGTCTATTGATCGAAATCAATCGTTTTTTCCCGGATGCGTTAACATTCCCCTTTTTTTCATT------CTAGTTAT-----TGACATGGTA----AGGGGGT----AACGAAGATTAGAGATAGGATCCACTATCTGTGA-----CTAATCCC-----CCGCCCTTTCTCCCTTTAAC-----------CTTATATT------------------------------CGA---------A---------------------------------------------------------------------------------------------------------------------------------------------------------------------AAGAGTGAGAACGGAAATTTAAAAGTGGGTCTAGGGCAAAAGT--------CTC----ATACACGAGACT---------TAAACGAAATACTCTACTGTATACTCTACTGTGA----------TTAGAAA---TATAGTTTGAGGCAAAATTGATTTCGAACTCTA-------AA--------GATAAATATTAGTACTAA-AAAAAA----ATATAATAGTT-----------AGAAATCATTGGATTACGCCTTCTTTATTATA-CTGA----------ATTATACTGA-----T-ACTGAA--------------------------------------TTCTATTTCCTATTAATAT-TTACTATTCTTTTTTTTAT-----TA--TTTACTATTCTTC--------------TATTT-AATTTGCTGCAACGAAATTTTT----TGAA-----GTGTATTTCTAGTTTGCAATTTCTA--------------TTTTTTCTTTCTTTACGCTTTGGGTCGAAAATAAAAAAGTTGC-----TTGAATAAAAAATTCACACACAAAAG---GG----GGGTTCATGGCCAAGGGTAAAGAT------GTCCGAGTAAGCGTTATTTTGGAATGTACTAGTTGTGTTCGAAAGAATGTTAATAAGGAATCAAGGGGTATTTCCCGATATATTACTAAAAAGAATCGACGCAACACGCCCAGCCGGTTGGAATTGAAAAAATTCTGTCCCTATTGTTACAAGCATACAATTCATGGAGAGATAAAGAAATAGATCGAACCGAG--CGTCTGTG----TA-TCGTCTT----------TTGAAACAAGAGTACAAAAGGACATATATTAT--ACATA----------------------------------------------TATTTCCTTATAT------------------------GCCTAAAAAA-------------TGATAAGAAAATGGAATCAA----------AA-------------------------AAATAT-TAT--------TCA----------------------------------------------ATAGATAATAATTCTAA---------------------------TATATAGATA---------TAGAAT-------------------TCTATTC----TATTTCT-------------------ATT-AGAAT----------------------------AAAAAAAAGAATATTAG------AAAATATAGAATAGATAAAAAAAGGGA-------------------------------------------------------------------------------------------------------------------------------------TTCCAGACTAGAA----------------------------------------------GCTA-TATAGA----------------ATATAA---------ATGT----ATAATAATATAA-----GCGATAA--------GTATATAAAAAA-------------------AATATAAATA---------------------------------------------------------------------TATAAATAAGGATAACATATAT--------AAAATAAACAAATTCAAA------TTAA-----AGAAAAGAAT-AAAAAAACCAAATCCTAT-TTCT------CATTTCTT--T-TTTTTAGAT----CCGACCGAAATAGGATTTTCGGTAGAA-TATTTT---------T-----------TATATT--------ATAACG----AATAAATAA--ACTAAACAAACCATGGATAAATCCAAGCGATCTTTACTTAAACCTAAGCGGCCTTTTCGTAGGCGCTTACCCCCGCTCCAATCGGGGGACCGAATTGATTATAGAAACATGAGTTTAATTAGTCGATTTATTAGTGAACAGGGAAAAATATTATCTAGGCGCGTGAATAGATTGACTCTGAAACAACAACGATTAATTACTATTGCTATAAAACAAGCTCGTATTCTATCTTTATTACCCTTTCTTAATAACGA---------------------------GAAACAATTTGAAAGAGCCAAGTCGGCCGTTAGAACTACGGGTTTTCGAGGTTTTCGAACAAAAA---AAC---AATAGGTTT-AC---------TTTTTTT--------------ATTCATTCAAGTGATGTTTT-------------GC----------------------------TCT-AAAAAA--TCCGATAATCCGGA-TTT-----TTTGTTGTCTCGGAAT------------------------AAAAATCGAGGAAGAAGAAAATTTTTTTTTATTGATATTGAATGCCTTTGTTCAT-------TTTGACTAC--TTT---------------------------------------------ATTATAATTATAAATTATTTGTTGTA-----------TTTTTTCTT------TTTTCGGACTA-----ATTTATATTCTATCTTCCCTTCCCGGAGTTCCTTCTCCGGGGAACTTTG-TTTAAAAAAATTCGGGTGCTTTTTTCCAATCTTCTTTTTTTATGATCTCATTGGAAATAC-----TATAAAGACAATTCCTATTTAAAATAGCTATTTGTGCAAGTATTTTACGATTAAGAATCAACTGCCGCTTGTACAGATCATGTATAAATTTACTATAGTTATAGTATACGCCCTTTTCCGTTTCGCGAATTGCTGCGTTTATCCGAGTAATCCACAACCGACGAAAATCTCTCTTTTTCTTATCTCTATCTCGATGAGCCGAAAACAAAGCTCTTATTTTCTGTTGAGCAATAATACGAATAGGTTTTGAATGGGCCCCTCGAAAGCTTGATACAAATAAACGCATTTTTTTTCTACGTCTCCGAGCTATATATCCCCGTCTAACTCTGGTCATTGAATAAATAAAACTTT------------GCTAAATAACTAATTGA-----TTTTATTTCTCGTTGAGTTA--TTTTTTTTCTTTCCTCACTGG------TAATAACAAAACGGATTTTTCCAATGTATAAAAA--GAATTCCAATGGCTTTTGCTACTATAACCTTCCCGACCACGAT----TT---------------TTT-CTTTTTTTTTTCGAGGCATTTTGCCTCAACTCAAAATTAAATAAGAAAATG----------AAATAATAAATTGGATTGATACTAGGTATCAAAAATAAAAACGTAGTA---------AATCTAGATGAATAAAGAAAT------AGTGGGTTCCTTCGTTTCTATGGTTCCTTCTTAAACGGTGAGGTCCTCTCTATACACCGGAG-CCCTT-TACTTCGTTTCGTCAACGTTATTGGTAACTTGTACAATTCAAAATCTTTGGCTCTACCCATGAATTATCCAGTAATAGGTCTTTCACAACGAGATCCGCCTATCCAGTAACGGTATTTAG-----TTTTGAAGGTTAGCTGGATAGC-TGACCCTGTTAGTCCGTT-TTGCA-----AAAATGGGAGCATAA----TC--TTTTTTCTTT------AAAAAC--CGTACTTTCCCGCGTAATGTATAG-CATTTGCTACCAA-TGGGAACTTGCTTCTCA--TCTTAAATTGAGCTAATTGGGGTTACACCAAGGGAAACCATAAATTTAATACGCAAT-----------AGATGGATATG------GTAGATC---TTT-----TTTTCGATAGTGACCAGAGTTCTTCCAT----TTTATCCTA-----TTCACTGGTAATGCTCATTGATGCTGGAAA-TTT-------------GCCCAGTTCATAATTTGAACGAGTCGCACATACACCCTAGTACATGTTCCTCGGCGTTGAGGACATCCCCGAAGAGCGGGGGA-----------------------------------------------------------------------------------------------TTTCGTGACGTTTCGAATTGGCTGTCTTGTGTTTCTAATAAGCTGTTTAATAGTTGGCATGCTGA---------ATCAT-----TTACA-TAAGGGACTGGTTTAGATGAATCCTAACCTGAT-----GAGTATGAATTCTTTCT----------------------------------------------------------------------------------------------------------------------------------------------------------------------------------------------------------------------------------------------------------------------------------------------------------------------------------AGT--------T-----------------------------------------------------------------------------------------------------------------------------------------------------------------------ATATAGAA--------------------------------------------------------------------------------------------------------------------------------------TA-TT---------------------------------------------------------------------------------------------------------------------------------------------------------------------------------------------------------------------------------------------------------------------------------------------------------------------------------------------------------------------------------------------------------------------------------------------------------------------------------ACCCAGG-AA-------AGTCA----AAA------------------------------------------GAG--------AAAATAGAAATTTGC-----------GCATTTGACTACTTCGGATCTTT----------------------------------------CCATTGGTCCTTCTTTACTATTTCGACTCCTTCAT---------------------------------------------------------------------------------------------------------------------------------------------------------------------------------------------TCGCTATAAGATCAATAATTCCGTGAGCTTGGGCTTCTCTTGCTGACATAAAAACATCCCTTTCCAGGTCTTCGGTTAGAACCCAAAAAGGTTG-----GCCTGTTCTTTGTGCATAAACCTTTGTGAGTGTTTCTCGCAAAGTCAATAATTCTTCTGCTTCCATCA------TACATTCTCCCGTTGCTCCCTCATGAAAAGAACTAGCAGGTTGATGGATCATTACCCTGATGATATAAA---AAAAAGAAGGTTCCTCCATCTCG-------CGTGATGAGGCGAAGAC---------------AAAAGATAGGGAATAACA----------ATAAACTTGAACAACCGTACGTG------CATCTTTTGCTCATTGCATACGGCTCGACAGTGGAATTTACTTTTTTCTTCCATCGAAGAAAAATAGAATCGAT---------CAGATCCAGATCACTAAATCATCCAATTACCACCCTTCTTTTC-----------GTGAGTTC-AAAATACTATGATGGCTCCGTTGCT-----TTCTATA------------------TAAATTTCGTCTGTAATTCAGCAATCCCAAAGTTTCTTTTTGATCCTAAATAAG------AATA-----AGGAATTTTTT--------TTATTTTCGTACTCTTTCAT---------AAA-----TATTGTTAGGTTA-----AAGAGTCTTTTGGTATAA------AAAAGGTTTGTGACGCTGAAACGGACTCCGGATAAATAAAAAA---TCGGGAATA-TCCTTTA-TCTCATACTCCTCTCTCGATACATAATTTAATGTTTTG------------AAAAAAA----------------------------------------------------------------------------------------------------------------------------------------------------------------------------------------------------------------------------------------------------------------------------------------------------------------------------------------------------------------------------------------------------------------------------------------------------------------------------------------------------------------------------------------------------------------------------------------------------------------------------------------------------------------------------------------------------------------------------------------------------------------------------------------------------------------------------------------------------------------------------------------------------------------------------------------------------------------------------------------------------------------------------------------------------------------------------------------------------------------------------------------------------------------------------------------------------------------------------------------------------------------------------------------------------------------------------------------------------------------------------------------------------------------------------------------------------------------------------------ACTAACCAAATTTTGCATATCGAATTCAAAGTGCCATG---------CTATTTTTACTTAACATTAC---TCATA-----ATATATAATATAG-------------CTTGATATTTCTT-----GTAGTGAAGGCATAGTC-----TTTTTTTCT------------------CAAATAAAAAA-CTCATTGGCG----CCAAAGCCAAGCGTGAGGGAATGCGAAACGTTTGGTAATTTCTCCTCCGACCAGGATAAAAGATCCCATTGAAGCGGCTATTCCCATGCATATTGTATATACATCTGGTAGCACAAGTTGCATAGCATCAAAAATAGCTATTCCGGGTAATACCCATCCGCCAGGACAATTTATAAACAAATACAAATCCTGGGTCTGATCCTCTATACTGAGATATATGATAAGACCAACAAGATAATTCGAGATCTCGGTCGTAATTTCTTGGGTTAAAAAAAGTAATCTT----------------------------------------------------------------------------------------------------------------------------------------------------------------------------------------------------------------------------------------------------------------------------------------------------------------------------------------------------GCTCGATAAAGTCGGTTGATTAGGGTAAAATTTTATCCCTTAG--GAACCGTACATGCA-CCTTTTGATGCATACGGCTC-AAAAAA-TGTGAAAAAGAAAAAAATCAATGTCTAGATTACTTCCCTCT----------TTTTTTTTTTATAGTAGTTC--------TTTCTAACTTCTAATGA--AGGGAGGTTGTCTTCTAT-----TTTTCAA---------------TAAATATGAGTTTTTCTTC--------------------TACTATTAAAA-------------------------------------------------------------------------------AAATTAATTATCTATATATATTT----------TAAATATATA----------------------------------------ATAAAGAAATAAA-AAAAGAGAATATAATGATAATGAATATAATATGATAATTAATATAG------TAAATCAAAGTAAAAAGATAATATAGAAGACTCC---------------------ATATCTAGA---TACAA--------AATAGAAT-----AAAGGCATCAT---------------------------AAACATAAACAGAAT-----------------------------------------------------------------------AAAAAAAATTACAT------------------------------ACATAT-ATATAA-----------------------------------------------------------CAAAC--------AATATGCAAAT-AAAAAA--------------------------------------------------------------------------------------AAAAAAAAAGAATAGTATGTATAAAGAAAGAGTCTTTCTATAGGTATA-------------TAAAA----------GGTAAATTTTTCGAACGAACTGC-----TCGTTGATTTCTTGTTTCATCGAGATCGACTGTAAACCACGATGTCATTTTCTTGTTCTTGAAGGGGC-----CTCTTTAATTC-----TTTTAGGTTTATGCTCTACTCCGGGTAAAACGATG---------------------------------------------------------------------------------------------------------------------------------------------------------CTCGATTTCGATTTGCACA-TATAGGTCAAATGCATCTAATACCGCTT-------TTTTTCTCAGA------------TTTTTTTCATTTAGTGCATGCCTTTGC--CAAAAAATTGGATATTGGACATATTGAGTTCA-----TCTAGTTTATTCGAGTG------AT----TAAGGTTCAGATGAGTCCTCTACCTA---------------------TTCTAAT------------------TTGGATAA----------ATAGGA-------------TTTCATACAAG------CAGTAATTATCGATATATT-----AACAATAGGG-----ATTTGTTTAAACGGAGCCTGGATACTTCATGTCATTTTATTGGTTGAAC-----CAAGCCAACCATAAAGTATTCTAATTGATACTATTAGTC--T-GAATCCCCCTACAAATGGATCTAGTTGGACTTCGCGCTCCAAATTTTTGATGAT----TCAATCAATCTTGCTTGGGCGAAGAGAAGGATATC-TCGATCGGGGAAGAGAACGGGGAAAGCCCATATGACCCAATATATCTGACAAGTCGCACTATACGTCAACCCAAGTTGCATCTTCATCT---------------CCCGGAATTCGAAAGGGTACTTTTGGAACACCAATAGGCATTAACT-AAAAGAAAAAAG-AATTAAGTACTATATTTCACTTTGGTATGGAA------------------ACGTAAT------------------AATCGGGCTATTCTCTTCA------TAATATGAACCTTTG----------TCATATATGTTGATA---TTC-----TTTATCATATATTCTGTCTAG-------------------TTAGAAAAG---------------------------GTCATAAAAGCCGAT-----------AGAATAAC---------TAAAGGAA---------AATTCTTACGACTAGAGCCGTCCAATGATGAACAAAT---ATGGCGGCATTTGCTCATAG-AAAAAGGTATCAACCCCCATTGCGTATTGGTACTTATTGGGTATAA-----------------------AATAGATCTGTTTCTCTTTGTTCCTACAAACCTAA-TTGTTCCATTAT-------------------------------------------------------------------------------------------------TAAC-----------------------------------------------ATATAGAATAG----AACAAATATTAACCCTT-GCGCCGAGATAATCT-----AC--------TGCGC-----AGGGGTCCGTT-----------------GATAGTCATAGT-----CTTTTCCAATGCAATAAAGTTACATAGTGTGTATTTTT-TTTTATA--AAGGGGTATTTCCATGGGTTTGCCTTGGTATCGTGTTCATACCGTTGTATTGAATGATCCTGGTCGGTTGATTTCTGTTCATATAATGCATACGGCCTTGGTTGCTGGTTGGGCCGGTTCGATGGCTCTATATGAATTAGCAGTTTTTGATCCCTCTGATCCCGTTCTTGATCCAATGTGGAGACAGGGTATGTTCGTTATACCCTTCATGACTCGTTTAGGAATAACCAATTCCTGGGGCGGTTGGAGTATTACAGGGGGGACGGTAACGGATCCCGGTATTTGGAGTTATGAAGGGGTGGCCGGGGCACATATTGTGTTTTCTGGCTTGTGCTTTTTGGCCGCTATTTGGCATTGGGTCTATTGGGATCTAGAAATTTTTTGTGATGAACGTACAGGAAAACCTTCATTAGATTTGCCTAAGATTTTTGGAATTCATTTATTTCTTTCCGGGGTGGCTTGTTTTGGTTTTGGCGCATTTCATGTAACAGGCTTGTATGGTCCTGGAATATGGGTGTCTGATCCTTATGGACTAACAGGAAAAGTTCAGTCAGTAAATCCTGCATGGGGCGTAGAGGGTTTTGATCCTTTTGTTCCAGGGGGAATAGCCTCTCATCATATTGCAGCAGGGACATTGGGCATATTAGCGGGATTATTCCATCTTAGTGTCCGCCCGCCCCAACGTCTATACAAAGGATTACGTATGGGGAATATTGAAACCGTACTTTCCAGCAGTATCGCTGCTGTCTTTTTTGCAGCTTTTGTAGTTGCCGGAACTATGTGGTATGGTTCAGCAACTACTCCGATCGAATTATTTGGTCCTACTCGTTATCAATGGGATCAGGGATACTTTCAGCAAGAAATATATCGAAGAGTTAGTGCTGGGCTGGCCGAAAATAAAAGTTTATCAGAAGCTTGGTCGAAAATTCCTGATAAATTAGCCTTTTATGATTACATTGGCAATAATCCGGCAAAGGGGGGGTTATTCAGGGCAGGTTCAATGGACAATGGGGATGGAATAGCTGTTGGATGGTTAGGACACCCAATATTTAGAGATAAAGAAGGACGTGAGCTATTTGTACGTCGTATGCCTACCTTTTTTGAAACATTTCCAGTCGTTTTGATAGACGGAGATGGAATTGTTAGAGCCGATGTTCCTTTTAGAAGAGCCGAATCAAAATATAGCGTCGAACAAGTAGGTGTAACTGTTGAGTTCTATGGTGGCGAACTCAATGGAGTCAGTTATAGCGATCCTGCTACTGTGAAAAAATATGCTAGACGTGCTCAATTGGGTGAAATTTTTGAATTAGATCGTGCTACTTTGAAATCGGATGGTGTTTTTCGTAGTAGTCCAAGGGGTTGGTTTACTTTTGGACATGCTTCCTTTGCCCTGCTCTTTTTCTTCGGACATATTTGGCATGGGGCTAGAACTTTGTTTAGAGATGTCTTTGCTGGTATTGACCCAGATTTAGATGCTCAAGTAGAATTTGGGGCATTCCAAAAACTAGGAGATCCGACTACAAGAAGACAAGCAGTCTGATACAAAA------TTGCTTTCGTCATTTTCGTCTCTCTTTTTGTGATTTGA-----CATTAGGGATCAGAGAAATCTTGATTTAATCAT--------------TTGACTCTTT------ATTTATCAG--------GGAAATAATCCCCAAT-AAACAGGTATGGAAGCTA-----------TAATCGTAAACCACAATCGAATCTATGGAAGCACTGGTTTATACATTTCTATTAGTCTCGACTCTAGGGATAATTTTTTTCGCTATCTTTTTTCGAGAACCGCCTAAAGTTCCAACTAAGAA--------ATGATTTTTCATTATCTCTGTTGAAGTAATGAGCCTCC-------CAAT-----AT---------TGAATGCATATCGG-----------------------------------------------------------------------------------------------------------------------------GAGGCTCATTAC-TTC------------------------------------------------------------------AACTAGTCCCCGTGTTCCTCGAAGGGATCTCTTAGTTGTTGAGAGGGTTGCCCAAAAGCGGTATATAAGGCATACCCGGTAAAACTTACAAGTAAACCAGATATAAAGATGGCGACTAGGGTTGCTGTTTCCATTCTTAT----------------------------------------------------------------------------------------------------------------------------------------------------------------------------------------------------------------------------------ATGAATTAAAGACCGCAAAGTAT----CTCTGATAAGATCCTTTATTTAC--AGGGGAATGGTATACAAAGTCAACAGATCTCAATAAATACAATCGGATTTATGGCTACACAAACCGCTGATAGTAGTTCTAGATCTCGTCCAAGACAAACTACGGTAGGGGCTTTATTGAAACCGTTGAATTCGGAATATGGTAAAGTAGCTCCCGGGTGGGGAACTACTCCTTTGATGGGTATCGCAATGGCTTTATTTGCAGTATTCCTATCCATTATTTTGGAGATTTATAATTCTTCCGTTTTACTGGATGGAATTTCAA-----TGAATTGAATACACAAGAAC----TATTAAGTTCGAGTT---------TT----TCA-----ATAC-AAAGTTAATT-----CTCGGGTTTCTTGTTTATAACCCTGTTGCTGTTGGTAGTTCGATCGCGAAA----TTTCTTTCTGTATTTCCGG-AATATGAGTGTGTGACTTGTTATAATTGATCCTATTGATAATACAGAGAATGAGTCTGTCATCTTATCTTTAT-AAAGACGGTTCTACCTCGTCGGATACTCATCCTAGTATCTGGAGCACAG-----AATATTATGAAATAGATCCAGAA----TTGAACTATGATTCATACTT----A-----------ATAATCAGACCTTGTGACCGG-------AT----TCTAAC-TACAAAATTTTCAACGAATT-----------------------TGATT-ATAAATT------GAA--------AGATTTTTCT---------TTCTTTTTATGCTTATTTT-------------GACTAA-AAGGTAAATTAGGTAAATTTTTTCGTATTTTGAGTCATTA-----TACCT-------------------ATGAA-----TAAGTGATGATCCGATAGTTCTTACTCAGGGAATCTTTG-----GGCTTGGGGTTTTT-----ATTGAATCATCATGGTTCTAGTATGAATCTGGGGTTTCA-------------------------ATTAA----TTTATAGGGTCTTAACAAGAGAAATTCCTATCAATGA-----GAAAAAAC---------AATAGTAAAAGCCGGATTACACACAACT----AACAAAA---AAAAATAGGGAAAGAGAAGATTCAAGAGGCC----TGTAGTAATAATAA-----AGCGAAAAAGGAAGAGCCGACTTGATATTTTGGCATTATTACC-----ACAAAGAATAACTTTCGTA---TTTTTAATGCTTCGTATCTGCACTTCCGCGAAGATTAAATC-------------AGAAGAT-----------ATATTCTA-TATGCGTCGGGAGCAGTATTTGTGTGTTTCTGCTTGAGCTGTACGAGATAAAATTCTCATATACGGTTCTCAGAGGGGGAGTCCCCC-------CGGTTTACCTATCTCAATAAAGTCTACGATTGGTTCGAAGAACGTCTCGAGATTCAGGCGATTGCAGATGATATAACTAGTAAATATGTTCCTCCTCATGTCAACATATTTTATTGTCTAGGCGGAATTACCCTTACTTGTTTTTTAGTACAAGTAGCTACGGGGTTTGCTATGACTTTTTATTATCGTCCAACTGTTACTGAGGCGTTTGCTTCTGTTCAATACATAATGACTGAAGCAAATTTTGGTTGGTTAATCCGATCAGTTCATCGGTGGTCGGCAAGCATGATGGTTCTAATGATGATACTGCACGTATTTCGTGTGTATCTCACCGGTGGGTTTAAAAAACCTCGAGAATTGACTTGGGTTACAGGTGTCGTTCTGGCTGTATTGACCGCGTCTTTTGGTGTAACTGGTTATTCCTTACCTTGGGACCAAATTGGTTATTGGGCGGTCAAAATTGTAACAGGCGTCCCTGACGCTATTCCTATAGTAGGATCGCCTTTGGTAGAGTTATTACGCGGAAGTGCTAGTGTGGGACAATCTACTTTGACTCGTTTTTATAGTTTACACACTTTTGTATTGCCTCTTCTTACTGCTGTATTTATGTTAATGCACTTCCTAATGATACGTAAACAGGGTATTTCGGGTCCCTTATAGAGAAGC-------------------TTATAGAGAAGATAGATCATA---------GATCTTTGTAATCAACCGCTTATCACTCACTTGGGGAAGGAACTATAGTATTTCATTGCTACAAATGTGTCT------TATTAATA-------TG-------AATAAGACATGTTTTTGGACATTCT--CTTTCCTTCAACCC-------CACAAT-----ATT---GTAA-------------TATTGTA----------TTATGTTA----TTTAACATAAC----ACGACTAGTTGAAGGGAATTCTCCGAAAGGA-AAATGGATTATGGGAGTGTGTGACTTGAACTATTGATTAGG-CCGTGCGGAT-----ATATGCC----CTTTTCTGCCACATTGAAAT---------A----ATGTGTCTTTGTTCCAACCACCGTA------TAAGCTAT-----------------------AT----ACAGACGATAGGCTGGTTCGCTTGAATAGAATTCTTTCTATGATCAGCCCCGAATCATGTCATGCATGACCAGGCTCCGTAAGATCCAGTCGAATCAATGAT-----TTGGCAGAATCCAGATTCCATTTTATTT-----A-----TTTC----GTAATTTTATTAA------TGTTTTGT----TTTA-ATAGTATGGAAATGCATTCATTTCCTCTGCATCGACGCGAC--------CTATGATACTATCGGAGTGAAACAAGGCATCTAAAGAAGACTC---GAGGCT---ATATGTTAGTTAGTAACAAGTAAACCTTTTGC-TTTGTATGT------AAAAAGTCTCACA--ATTTGTTGAGAGAAACACCAATCGC-----AAAGTCTAAGACGACCCAGAAAGCATTTGAGCACGATCAAC----TTTGTAAGCCTACTTGGGGGTTGA----------GCATTTATCTGGAAAAACGGAATTCCTT----GTAATGGGTAATTGCAACTTTGGAAAGGGGAATCTAGTCAAACTTTTCATACTTCAT-ACAGAGAACCATTCAT-------ATATG-----------TATGGATATCAACAACATAAA-ATGTA-------TATATATCTTAAC-----------CTAT--------------ATATGT---------TG---------------ATCCTTTTCATTCTTGCTCGAGCCGGATGATGAAAAATTCTCATGTCCGGTTCCTTCGGGGGATGAGTCTATAATAATTCACCTATCCTAATAACAAAAAAACCTGACTTGAATGATCCTGTATTAAGAGCTAAATTGGCTAAAGGAATGGGTCATAATTATTACGGGGAGCCTGCATGGCCAAACGATCTTTTATATATTTTTCCAGTCGTCATTCTAGGGACTATTGCGTGTAACGTAGGCTTAGCCGTTCTCGAACCGTCAATGATTGGCGAGCCAGCGGATCCATTTGCAACTCCTTTGGAAATATTACCTGAATGGTATTTTTTTCCCGTATTTCAAATACTTCGTACAGTACCCAATAAATTGTTGGGTGTTCTTTTAATGGTTTCAGTACCTGCGGGATTATTAACAGTACCTTTTTTGGAGAATGTTAATAAATTCCAAAATCCATTTCGTCGCCCAGTCGCCACAACCGTTTTTTTGGTTGGCACCGTAGTGGCCCTTTGGTTAGGTATTGGAGCAACATTACCGATTGATAAATCTCTAACTTTAGGTCTTTTT----------------TAACTTG----ATTCAATTGTA-----------------------------------------------------AAATAATCTGACGTGTGTATCTAGGGAATAGTTGCTTCAAAGTTAATTTTCCCTAGATACATCGATTTCG--------TTTTA--------------ATTGCATGCA---------------------------------------GGAGCTATTCCTAATAATACGGATTGCGCT-AAAAA--------------------------------------ATGAAT---TGAT---TATTG-----------------------------------------------GTTTTGATTAAATTAT----AAAAGAAA-AAATCCAA-------------------------------------------CGAATTTAAACC-------TTCTTTTTAGGTAAATT------AAT----------------------------TACGAAATGCTTTTCGAGAGTGTCCAAAATTTGTTTTACATCTTCTATATGAAAATGCTCGATTTTCATAAGATCTTCTTG------ACTGTTATTCAATA---GGTCCAACAATATATGTATAT------TGGCATTTTTTAGGCAATTATAGATCCTGGGAGGCAATTCTAATTGATCAATAAAAATCGATTTCAATGCTATTTTTTTTTTGTTTTTTCTTAGTTCAGTCAATCTATCATGAAAGAAAAAAAGGGGTAAAGTAACCCTGTCTTGATTATCCTCTAAATAGAAGTTTTCTTTTTCCGCATATAGAAAGGGAATAAATAAATCAATTAAATTCCGAGAGGCTTCATACAGTGCTTCTTTAGGAGTTAAACTGCCATTTGTCCATATTTCGAAAAAAAGTATCTCTTGTTTTTCATTACCATTACTATATGAATGAATACTATGATTCACATTTCGAACAGGCATGAATACAGCATCTATAGGATAACTTCCGTCTTGAAAGTTATTTGGCGTTTTTATACGATATCCGCGATTCTTCTCGAGTTGTAATCCAATACGCAAATCTATCGGTTCTGTCAAGTTAGCTATATGTTGTGTAGTATCAACGATTTCCACATAAGGTGGTAAGATGATATCTTGAGCAGTTACACATCCAGGTCCCCTAATACAAATAGACGCGTCACAGGTTCCATATACATTGCTTCTCAATACAATTTCTTTCAAATTCATTAAAATTTCATGTACTGATTCTTGAATACCTACTATAGTAGAATATTCGTGTGGTATTTTCTCAGATTTTGCGCGTGTAATACATGTTCCTTCTATTTCTCCAAGCAGAGCTCTGCGCATCGCAATTCCTATTGTGTCGGCTTGACCTTTCATAAGTGGAGATAGAATAAAGCGTCCATAATAAAGACGTTTACTATCTGTTCTTGATTCAACACACTTCCACTGCAGTGTCCGAGTA------GATACTCTTATTTT------CTCTCGAACCATAGTAATATTATAGATAATAGATCAGATCGTTGAGTCATTTATTTCTCTTGAAATCCC-----TTCAATGCTTA-------G-TTTTTTTTTACACACGTCTTTTTTTAGGAGGTCGACAGCCGTTATGCGGCATGGGGGTTACATCTCGTACGAAACTTAACAGTATGCCGCTTCTACGAATAGCTCGTAATGCTGCATCCCTTCCGAGACCAGGACCCTTTATCATGACTTCTGCTCGTTGCATACCTTGATCTACTACTTTACGAATAGCGTTTCCTGCTGCGGTTTGAGCAGCAAATGGTGTCCC---TCTTTTTGCCCCTTTGAACCCGCAAGTGCCAGCGGAGGCCCAAGAAACCACTCGACCTCGTACATCTGTAACAGTCACAATGGTATTGTTAAAACCGGCTTGAACATAA------ATAACCCCCTTGGGTATTTTACGTGCACTCTTA---CGTGAATTAATACGCCTATTTCTATGTGAACCAATTCTTGGGATGGGTTTTGCCATATTTTATTGTCTCATAAATATGAGTC------AGAGATATATGGAGATATCCATTTCATGTCAAAACAAAACAAATCTTTTCG-TTTTTTTTTTTTTTTATTGGTACATCATATTTGTACATCGAGTCCGTTAGAAAGTCTCTTT---------TA---TTTAGTAGACTT------ATTATCCTTGTCGTTGTTTATGTTTCGGGTTGGAACAAATTACTATAATCCGTCCCCGCCTACGAATTAGTCGACATTTTTCACAAATTTTACGAACGGAGGCTCTTATTTTCATATTTTTAATTCC----TTAC----TTTAATTCCGAATCCATTTCTTGGAAGAAAATAAGTTTCTTGAAATTTTTAATCTC-----GTATTCC--------------GGAATGTAGAAGTG----GAAAAACAACTTAATCGG-------------TTGAATCCTTGTTACGGAGTCTATAAATTATACGTCCTCTGGTTGAATCATAACGGCTTACTTCAATTTTAACTCTATCCCCCGTCAGGATTCGTATAAAACTACGACGTATCCTTCCCGAAACATAACCTAGAATCAGATCCTCATTATCTAAACGAACCCGGAACATGCCGTTAGGAAGTGATTCAACAATTAAACCTTCATGAATCGATTTTTCTTCTTTCATTCCAGGTAAAACCCCT-----TTAAAGTATCAACTAATGGAG-------GAGGAGTGATATTAGACAACCCGTCCTTTCTC-------------TTTTTTTCCAAAATAGGAAATTTCGGATCCAATTCGTATAT----AAGAGGGATTACCATATATAACATAAAATTTCTCCACCAATTCTTTCTAGTCGAGCCTCTCGGTCTGTCATTATACCTCGAGAAGTAGAAAGAATTAGAATCCCTATTCCACCTAAAATTCTAGGAAGTTTTTGATAGTTCGAATAGATTCGTAGACCGGGGCGACTGACCTGTTTTAAATTTAAAAATCTTGTATATGGCCCTTTCCTATTCCTTCTATGTCGTAGAGTTGAAACCAAAAAATATTTGTTTTTTTCCTGATGTTTTCTCACGTTTTCGATAAAACCTTCTCGTAAAAGTATTTTAACAAAGGTTTCCGTGATTTTAGTCGATGTTATTCGAACCGTTCCCTTTCTATTCATGTCAGCATTTCGGATCGAGGTTATTATATCGGCAATGGTGTCCCTACTCATAATGCCCTAAAATTTGTGGTGC-TCCGAATTTTTATATAATCAACATG----TTTTTCTTAG----TTTATTC------------TTTTTCTTTT------AT-----------------------------GTAAA-AAGGAAAGGTATATACGTGATACACAATCTAC--------TGC-----------TCTATTTCAT---TCAAATAGCCCACT---------------------------AT-TCTCGTGGTTTATAATACCTCGGGAGCTAATGAAACTATTTTTGTAAAGTTTAATTGTCGCAATTCTCGGGCGATTGCACCAAAAACTCGAGTTCCTTTTGGATTTCCTTTTTGATCAATAATAACCGCAGCATTGTCATCATATCGTATTATCATACCGTTGTCGCGTTTGAGTTCTTTGCGGGTACGCACAATTACAGCTCGGATCACTTCGGACCTTTCTAAGGGCA------TATTTGGTATTGCTTCTTTTATCACAGCAACAATAATGTCGCCAATATAAGCATATCGACGATTGCTAGCTCCTATGATTCGAATACACATCAATTCTCGAGCCCCGCTGTTGTCTGCTACATTCAAATGGGTCTGAGGTTGAATCATATCA----TTTTTTAATC-----TTTCAATGCAAA-----GGCGAAA-------------------------------------------------------------------------------------------------------------------------------------------------------------------------------------------------------------------------------------------------------------------------------------------------------------------------------------------------------------------------------------------------------------------------------------------------------------------------------------------------------------------------------------------------------------------------------------------------------------------------------------------------------------------------------------------------------------------------------------------------------------------------------------------------------------------------------------------------------------------------------------------------------------------------------------------------------------------------------------------------------------------------------------------------------------------------------------------------------------------------------AAAGAAAAAGAAATATTATTTGTC-----------------CAAAAC-AAAA-------G----------------------------------------GTG------TTTT-TTTATCCCAA-----CGTTTGTTT--------------CTACA-TTCCTATT------CTGAAATAAGAAATTGAGTTCGTATAGGCATTTTGGATGCTGCTATTGAGATAGCTTTTCTGGCGATTTTTTCTGTTACTCCGCTTATTTCATAAAGTATTCGACCTGGTTTGACAACAGCTACCCAATATTCGGGGGATCCTTT------CCCCGAACCCATACGTGTTTCCGCAGGTCTTACTGTAACTGGTTTGTCTGGAAATATACGTACCCATAGTTTTCCACCACGACGCGCATTTCGTGTCATTGCCCGTCGTCCTGCTTCTATTTGTCTAGATGTAATCCAGGCGGGTTCAAGTGCCTGAAGAGCATATTTGCCGAAACAAATACGATTACCTCGATAAGATATTCCCTTCATTCTTCCTCTATGTTGTTTACGGAATCGAGTTCTTTTTGGGGTTATAGTGGATGGTTCTTTTTCAATTCCATCCCTATTACAGAACCGGACATGATAATTTCTTCTCATCCGGCTCCTCGCG-----AATGAAATGATCCAAAA------ATAGTAT-----------------------------------------------------------------------------------------------------------------------------------------ATAT------------------ATTTTAGAAAATTTATA---------------------------------------------------------------ATATTATAATA----------GAAGT--------------------------------------------------------------------------------------------------------------------------------------------------------------------------TATATAAAAAAATAAA--------ATA--------------------------------------------------------------------TAGTAAATATAT----------------------------------------------------------------------------------------------------------------------------------------------------------------------------------------------------------------------------------------------------------TTATTTCGATT----------------------------------------------------------------------------------------------------------------------------TTTATCAGCAT--AAAATAAA---------------------------------------------------------------------------------------------------ATATAACAAAAATTGAAATAT---------------------------------------TAAAAACTTTCT----------------------------------------ATTGA-TTTTTTTATT-----------TAATA--------------AATATAGTTATAAAGTATAGTTATAACAAATCTT----------------------------------TGTTTTCTTTTCGCTTTTCTCGTATC----AGATCGCC--------TATATTTTAGCAATTAAATAAGA---------------------AAAAAATGTCGCGGGCGAATATTTACTCTTTCAATATCTATTTCTGTT------GTAGGGTTAGTTCATGAC-TTCTCAGAAT-------AGATGAATTGG--TCTTTGGTTTATTCCGCCATCCCGCCCG-----CTGAATCATGTGTATTCATTTTCAATTGAATCTTCTGTATTCAC-AGGTTCCATCGTTCCCAGCGCTTCTTGATTAATGGTTAGGCCTGAATTTGACAACGGAGCTTTTACT----------TTATTTTG--AGTCAACGTTCTTAGTCTTTATTGGCTCGAGGCTC-CTTATTTTT--GTGCTACGAAGAGATTC--------GTATAATGCTAGATG--AATCTG------TATTGATGCTTTATTACATTGCCCTT------TTTGAGAGGATTCATAGACCTTACATAT-----TGGAATTATATATCATTGATA-GATTTTTATATCTTTCTCTCA-CCTTCCGTTTATCCACACCCTTTCGT------------TTACAACTCCTAATCGGATTGCTTTTTC---TTTT-GTTTATGCCA----AAGCGAATTCAGTTGCTGCAATGATAAGACCAACA--------T----ATCATATCTTGACTGCTTCCTTGGATCCAGAT-AA----TTTGAAGTGATGAGTTAGTTAT-------------------------------------------TAGT-----TC-------ATATTA------TGGTTTGTTAA---TA---TTTTATCTTAATCCTAAC-AAAAACCAACGAGTCACACACTAAGCATAGCAA----TTTG-----GTCAAAA-----GGGGGTCAATTGAA---TTTTTATTCAACCTTATAGAAT-----T----------------------------------------------------------AGAATTTATCATTTTTC---------------------------------------------------------------------------------------------------TTTTTTGTTCCGTCTT---------GG----ATATAAGGAAAAGACAAGTAAAGGCTTATTAT----------TCTATTCCTCGTCTATAAATATCCAAATTTTGATACCTAAAACCCCATATATAGTTCGAACTGTATAGGCACAATAATCAATTTTGGCGCTAATGGTTTGTCGGGGAACTCTACCTTCTCTGATCCATTCGACACGTGCAATTTCTTTTCCGTCGATACGTCCTGCAATTTGTATTTGAATTCCTTTTGTATCAGCCTGTTCAGTTAGTTCAATAGCTTTTTTCATCGCTTTTCGAAAAGAGACCCTATTTTTTAATTGTCCGGCTATAAATTCCGCAAGAATATTAGGGTGTCTGTAAGGTTTTGCAATTCTTGTAATAGCAATGTTGAGTTTTCGGTTCACAGAATTCAATTCTTTTTGTACATTCATCTGTAGTTCTTCGATGCCCCGTGGTCTATTTTCTATTAATAACTTGGGGAATCCCATATAGATTATGACCTGGATCAGATCAATTCTTTTTTTAATTTCTATCCGTGCAATTCCCTCAACACCGGAGGATGTTCTCATATTTTGTTGAGCATAATTCTTGATACAATCCCGTATTTTTTGATCTTCTTGTAAACCCTCAGAATAATTTTTCGGTTGTGCAAACCAAATGGAAT------------------AATGACTTTGGCTTGTACCAAGTCTGAAACCAAGTGGATTTATTTTTTGTCCCATATTGCCCCACT------------AGATTTTAAACGGAAATTGCTAGGTAAATACTTTTC-------------TCGATTATCT-AATGTTTC---------------------------------------ATATGTTTTTTTA--------TAGTACGATATAT------CTTTCAATACAATAGTTATATGACAAGTGGGTCTTTTTATCAGATAACTACGCCCTCGAGCTCGAGGTTTTAATTTTTTCCTGGTAGTTCCTTTGTTGACTTCCGCTTTCCAAACGATTAAATCTTCTTTTTCGAACCCTCTATTGTGTTTAGCGTTTGCTGCTGCGGAATAAATCAATTTAAAAATAGGATAACATGCTCGATAAGGCATAAGTTCGAGTATCATAAGCGTTTCTTTATAGGAACGTCCACGGATCTGATCAATTACTCTTCTCGCTTTGTGAACAGACATAGGTATATATTGGCCTA---------------AAGCAGATACTTCAGTCAGCGACTTTTTTTTTTTTCTCATAAGTTTTACCTCTGCA-T--------------------------------TAATGAAGGATAAGCATCTC------TA------------TTTAT-----------TAATT-----------------ATT------ATTAACGACGAGATCTATTATCGTTTTTTGCATGTCCCCGGAAATTTAGAGTAGGTGCAAATTCTCCCAATTTGTGGCCTACCATACGATCTGTTATATAAACAGGCAAGTGTTCTCTTCCGTTATGGATAGCAATAGTATGGCCAATCATTATGGGGATAATGGTAGATGCCCGGGACCAAGTTACTATTATTTCTTTTTCTTCCTTTGTGTTAAGCTTATCAATTTTGCTTAATAAATGATTCGCTACAAAAGGATTTTTTTTTAGTGAACGTGTCACGGTTAATTACTCCTA-TTT-----TTTTTTATTTAAAGACGAAGAAACTAATTAAAATTTCTCTCCTAT------TTACTACGTCGACGAATAATAAAATTATCACTATATTTATTCCTTTTT-CTACTTCTTCTTCCAAGTGCAGGAAAGCCCCATTTATTTGTTGGGCTTTTTCTACCAATTGGGGCCCTCCCTTCACCACCCCCATGGGGATGGTCTACAGGGTTCATAACGACTCCTCTTACTACAGGACGTTTACCTAGCCAACGCTTAGATCCGGCTCTACCCAAACTTTTCTGGTTCACTCCAACATTCCCCACTTGCCCGACTGTTGCTGAGCAGTTTTTGGATATCAAACGGACCTCCCCAGAAGGTAATTTTAATGTGGCCGATTTCCCCTCTTTTGCAATAAGTTTCGCTACAGCACCCGCTGCTCTAGCTAATTGTCCACCCTTTCCAAGTGTGATTTCTATGTTATGTATGGCCGTGCCTAAGGGCATATCGGTTGAAGTAGATTCTTCTTTTTGATCAATCAAAACCCCTTCCCAAACTGTACAAGCTTCTTCCAAA--------------------------------------------------------------------------GCATACGGCTTTCTGGATGTAGAT-GATGATATCTAT-ACAGATGGATCTTCTATTTATCATACAATGAAGTACTACATGAGCAGGTATATAGGAATCCCAATCTGCCGAATCACTCATGTTATGATCTTCTACATCCTAGGTCTTTCCGTTCCGTCATCTGGCTTATGTTCTTCATGTAGCATTCAGACCGAATGACTCTATGAAATTACGTCGATACTTCCAC-----ATATACATATTATGGGTAACGTAGGAGACATCTCTATTTTT-CCCCGGGGAATCTTTAGA---------CACTGCTTAGCTTTCAATTCGCCTCTGACCATCAAATGAAATGTGAATAACCCGTCCTCCTC----------TC-------TTTGAAAGAAGGGGC-GCTTCCGGTTC----TGTCGGTGCTTGAAACAATTTTGTCTTCTCCATATTACTATATCTCTAGAGTCAATAATTTTATATGAGGAACTACTGAACTCAATCACTTGCTGCCATTACTCTTCAGTTTTCTGTTGAGGTCTATCCTCTAGAGGTACTCAAATTGAATCCGTGATCGATTTCTAGGTTTCGTCGTAAACCTAATTGGTTACTTCCAATTACGTAAATCCATAGTTCAAACCGCACTCAAAGGTAGGGCATTTCCCATTTTTATAGGAACTTCTGTACCAGAAACAATGGTATCTCCAATTATAGCCCCTCTGGGATGTAAAATATATCTCTTCTCACCATCCCCATAGTGTATGAGACAAATGTATGCATTTCGATTAGGGTCGTATTCTATGGTTACGATTCTACCATATATGTCTTTTTCATTCCGTCGAAAATCGATTTTACGGTATAGACGCTTATGACCTCCCCCTCTATGCCTTGCGGTAATGATTCCTCTGGCATTACGACCTTTACCACAACGACGCTGTCCATAGATCAAATTATTTCGTGGATTGGATTTCACTTGACTGTCTACGGCCCTATTGCCTGTGTTCGGGGTAGAAGTTTTGTATAAATG---TATCGCCATGCTATT--AAGTA-TTTTT-TTTTAAGTTCTTTTCTTTCTAAGAGGTGGAATAGAATAACCCGGTTGAAGCGTAATGATCATACGTCTGTAATGCATTGTATGTCCCATAATAGGTCCCATTCTTCTACCCTTTCCCGGGAGTCGATGACTATTCATAGCTATTACCTTGACACCAAAGAAGAGTTCGACCCAA----TGCTTTATTTCTGTCC------------------------TAGTTGATCCTGATTCG-----ACATTAGAAGTATATTTATTTTTCCC-------------------------CAA----TAAC----C---------TGTCTGTAAATATTGCATATGTGATTCTATCTATTTTCTTCATATTCTGCATATATTGCATATGTGATTCTATCTATT------------------------------------------------TTCTTCCCTATGTATGAGTTCTAGTCTCAATAAGAATGCTAGCTCTTACTGTTCCTATGTTATGAAATGAATAT-----ACCAATTCGTTATGTATGGAGGATGAGATTCCATTGATACAGAGCCAATTCCA--------ATAGACTTATACTTATTGGAGGGTCCCATTGGCGTGCATCCAGTAGGAATTGAACCTACGAATTCGCCAATTATGAGTTGGGCGCTTTAACCATTCAGCCATGGATGCTTAGCGGGGATCCTCGTACATGGTGAATAACCAAATTCCAATTGAAATGAAATCTTTAGGATAAATCAATACAAATACAATTTTCATTTTATACAAATATCATTGAA---------------------------------------------------------ATTTTCATTG----------------------------------------------AAATAGAATATAGAAATGTTAGACATATTTTC---------------ATTTTCAGTGAAATATCATA------ATTGGAGTATCATAATAATAATAATATAT---------------CTATTATAGATATACATACAATTACGTTTTCG--------------TATTGTATTATACTA-------------------------TAGTATAAGAGTA------------------------TAAGT-------------------------ATCAAATTGCA------------------------------------------------------------------------------------------------------------------------------------------------------------TTTTT----T-TTAAC---------------------------------TTTAACAATTGCATATTA-------------AAGTAAATTTATAGTATTTAAAAATTTAA------------TTTTTTTTAACAAATGACAAAAAAA-----------CATTCCAAAAATTTC---GATTTCTGGGTTTTCGAGTTGAAAGAGAT------ATTGAGAGAGATCAAGAAT------------------------------------------TCAGTGGGATCTTTGGTTAAGATTTTTTTCCACCAAGAACGTTTTATAAAACTCTTTGATCCCCGAATTTGGAGTATCCTACTTTCACCCAATTCGCGGGGTTCAATAATTAAGCGATATTTCACTTTCACGATCACG------------------------GGTGTAGTATTCTTTGTAGTAGCGGGCCTTATATATCGTATTAACAATCGAAATATGGTCGAAAAAAAAAATATCTAT---TTGATAGGGCTTCTTCCTATACCTATGAATTCTCTTGGGTCCAGAAATGATAGATTGGAAGAATCCTTTGGGTCTTCCAATATCA------------ATAGGCTGATTGTTTCGCTCCTCTATCTTCCAAAAGGAAAAAAGATCTCTGAGAGCTGTTTCCTGGATCCGAAAGAGAGTACTTGGGTTCCTCCAATAACTAAAAGGTGTA---------AATCTAACGGGGGTTCGCGGTGGTGGAGGAACTGGATCGTAAAAAAGAG---------------------------------------------------------------------------------------------------------------------------------------------------------------------------------------------------------------------------------------------------------------------------------------------------------------------------------------------------------------------------------------------------------------------------------------------------------------------------------------------------------------------------------------------------------------------------------------------------------------------GGATTCTAGCCAATTGATAGGATCCTTT------------------------------------------GATCAATCTAGAGATCGCTTGGATTCCATCAGGAATGCGGATTCGGAATATCACACATCTCTCAATCAAAGGGA--------------------------------------------GATTCAACAGCGAAAAGAAAGATCGATTCCTTGGG-----------------------------ATCCTTCCTTTCTTCAAACGGAAGAAAGAGAGATAGGATCAGGCCGATTCCCGAAATGCCTTTCTGGATATTCCTC---------AATGTCTCGGCTATTCACGGAAGGTGAGAAGCAGATGAATAATCATCGGCTTCCGGAAGAAATCGAAGAACTTCTTGGGAATCCTACAAGATCCATTCGTTCTTTTTTCTCTGACAGATGGTCAGAACTTCATCTGGGTTCAAATCCTACTGAGAGGTCCACTAGAGATCAGAAATTGTTGAAGAAAGAACAAGA-TGTTTCTTTTGTCCCCTGCAGGCGATCGGAAAATAAAGAAATGGTTAATATATTCAAGATAATTACGTATTTACAAAAGACCGTCTCAATTTATCCTATTTCATCAGATCGGAGATGTGATAGGGTTCCGAAGGATGAACCGAATATGGACAGTTCCAAT------AAGATTTCATTCTTGAACAAAAATCCATTTTTTGATTTATTTCATCTATTCCATGACCGGAACAGGGGGGGATACACGTTACACCACGATTTTGAATCCGAAGAGAGATTTCAAGAAATGGCAGATCTATTCACTCTATCAATAACCGAGCCGGATCTGGTGTATCATAAGGGATTTTCCTTTTCTATTGATTCCTACGGATTGGATCAAAAACAATTCTTGAATGAGGTATTCAACTCCAGGGATGAATCGAAAAAGAAATCTTTATTGGTTCTACCTCCTATTTTTTATGAAGAGAATGAATCTTTTTATCGAAGGATCAGAAAAAAAGGGCTTCGGATCTCCTGCGGGAATTATTTGAAAGATACAAAAGAAAAAAGAGTGGTATT---------------------TGCTAGCAACAACATAATGGAGGCAGTCAATCAATATAGATTGATCCGAAATCTGATTCAA----------ATCCAATATAGC-ACTTATGGGTACATAAGAAATG-TATTGAAT------CGA---------------------------------------------------------------------------------------------------------------------------------------TTCTTTT--------------------------TAATAAAT--------AGATCCGATCGCAAC---------------------TTCGAATCTGGAA---------------------------------TTCAAAAGGATCAAATAGGAAACGATACTCTGAATCATAGAACTA------TAATGAAATATACGATCAACCAACATTTATCGAATTTGAAAAAGAATCAGAAGAAACGGTTCGATTCTCTTATTTGGATTTCTCGAAGCGAGAGATCCATGAATCGGGATCCTGATGCATATAGATACAAATGGTTCAACGGGAGCA------AGAATTTCCAT---------GAGCATTTCGTTTCTGAGCAGAAAAGCCGTTTTCAAGTTC-----AAGTAGTCTTCGACCGATTACGTATTAATCAATATTGGATTGATTGGTCTAAAGTTATCAACAAAAAAAATTTTTCTAAGTCA---------------------------------TTGTCAAAGCTGATTCTCTTTTTGTCTAACTCACTTCCTTTTTTCTTTGTGAGTTTAGGGAATATGCCCATTCATAGGTCCGAGATCCACATTTATGAATTGAAAGGTACGAATGATCAACTCTGCAATCCGTTGTTAAAATCACTAGGTCTTCCAATCGTTCATTTGAAAAAATGGAAAGC---------GGATGATCATGATACTTCCAAAAAATCGAAATTATTGATCAATGGAGAAACAATATCACCCTTTTTGTTCAATAAGATACCAAAGTGGAAGTGGATGATTGACTC---CCATACTAGAAAGAATCGCAGGAAATCCTTTGATAACACGGATTCCTATTTCTCAATAATATCCTGCGATCAAGACAATTGGCTGAATCCCGTAAAAGCATTTCATAGAAGTTCATTGATATCTTCTTTTTATAAAGCAAATCGACTTCGATTCTTGAATAATCCACATCACTTCTTCTTCTAT------TGTAACAAAAGATTCCCTTTTTATATGGAAAAGGCCCGTATCAAGAATTATGATTTTACGTATCGACAATTCCTCAATATCTTGTTCATTCGCAACAAAAAATTTTCTTTGTGCGTCGGTAAAAAAAAACATGCTTTTTTGGAGAGAGATACTATTTCACCAATCGAGTCACAGGTATCTAACATATTCATACCTAACGATTTTCCACAAAGGGGTAACGAAAGGTATAACTTGTACAAATCTTTCCATTTTCCAATTCGATCCGATCTATTCGTTCGTAGAACTATTTACTCGATCGCAGACATTTCTGGAACACCTCTAACAGAGGAAGAAATAGTCAATTTGGAAAGAACTTATTGTCAACCTCTT---------TCAGATATGAATCTATCTGATTCAGAAA--------------------------------------------------------------------------------------------------------------------------------------------------------------------------------------------GGAAGAACTT---------GCATCAGTATCTG----------------------------AATTTCAATTCAAACCTGGGTTTGATTCA------------------------CACTCCACGGTCTGAGAAATATTTACC---------------ATCCGAAACGAGTAAAAAATTGCGTCTTTGGCGAAAT---------------------------------------------------------------------------------------------------------------------------TGG-------------CTAAAGAAAGGCGTTGAGAAAGGGCAGATGGGTAGAACCTTTCAACGAG---------ATAGTGCTTTTTCAACTCTCTCAAAA------TGGAATCTATTCCAAACATATATGCCTTGGTTCTTTACTTCGACAGGGTACAAATATCTAAATTTGATATTTTTAGATGCTTTTTCAGACCTA----------------------------------------------------------------------TTGCCGA--TGCTAAGTAGCAGTCACAAATTTGTATCCAATTTTCATTATATTATGCACAGATCAG------CCTGGCGAATTCTTAAGCTAAAATGGCGAGCTCT------------------------------------TAAGCTAAAATTGTGGGGATTGTGGGCACCGATAAGTGAGATTTCAAGTGATATTTCGTGGAAGTGTTTCCGTAGGCTTCTTCGGGTCGAAG------------------------------------AAATGATTCATCGAAATAATGAGTCACCGTTGATATCGACACATCTGAGCTCGCCAAATGTTCGGGAGTTCCTCTATTCAAGCCTTTTACTTCTTCTTCTTGTTGGATGTCTCGTTCAGGTACTTCTTTTCTCTGTTTCCCTAGACTCTAGTGAGTTACAGACAGAGTTCGAGAGGATAAAATCTTTGACGATTCCATCATACAC---GATTGAGGTGTACAAACTTGTGAATGGGTATCCTAAACC---------TGAACCGAATTCTTTCTGGTT---------------------------AAAGAATCTCTTTCTAGTTGCTCGGGAACAATTAGAAGATTTTCTAGCAGAAATACTG---------------------GGTTTTGC---------------GCTATTTGGTGGTGGTCCCGCTTATGGGGTCAAATTTATACAGA--------AGATATTTT----TCAATCTCATCGATCTCAT------------AAGTATCATACCAAA---TCCCATCAATCGAATCACTTTTTCGAGAAATACGAGACATCTAAGTCATACAAGTAAAGAGATCTATTCATGGATAAGAAAAGGACAAA---------GGTTTCCGACTCATGATGAAATAGAATCCTGGATCGAGACCTGTGATTGGTTTTTGGATAAAGAGAGAGTTTACTCGTTTCATTTCTCCACCTTAAGGCCAGAAAAAGGGATTGATCAAATTCTATGGAGTCTGACTCATATTGATCATTTAGTAAAGAGTGACTATGGTTATCAAATGTTTGAACAAGCGGGAGCAATTTACTTACGATACGTAGTTGACATTCATCAAAAGGATCTAATGAATTATGAGTTCAATACATCCAGTTTAGCAGAAAGACGGATATTCCTTGCTCATTATCAGACAATCACTTATTCACAAACCTCGTGTGGGGCTAATAGTTTTCATTTCCCATCTCATGGAAAACCAA---------------------------------AACCCTTTTCGTTCCGCCTAGCCCTATCCCCCTCTAGGGGTATTTTAGTGATAGGTCCTATAGGAACTGGACGATCCTATTTGGTCAAATCCCTAGCGACAAACTCCTATCTTCCTTTCATTACGGTATTTCTGAACA---AGCTGGATTTTAAAATAGTTATTGAT---------GATCTCGATCC---TGAGGACTATATGGAAGCGCTTGATGATGTGGATATTGATCGTATTGATGA------TGATAGCGATCCTGCTAAGGACTATATGGATGCGCTTAAAGATGTG------------GA---------CGATATTGATGGTCGTGACTCTATTTATTCGAACTTG------------------GACTCGGACCCGGAGCTGAGGGAGGAGTATACGGCGGATGATGAGATACTTAGGTATATCATCGAGTTGGAAATAGACCTA---GCTTCTATCAACTTGCAATTCGAATTGGCAAGAACAATGTCTCCTTGCATAGTATGGATTCCAAACATTCATGATCTGTATGTGG------ATGAGTC-GGA------GTCCCTCGGTTTATTATTGAACTA---------------TCTCTCCGGGGATTGT---GAAAGACGGTCCACTAGAGATATTCTTGTTATTGCTTCGACTCATATTCCCCAAAAAGTGGATCCCGCTCTAATAGCTCCGAATAAATTAAATACATGCATTAAGATACGAAGGCTTCTTATTCCACAACAACGAAAGCACGTTTTCACCCTTTCATATACTAGGGGATTTCACTTGGAAAAGAAAATGTTCCGTACTA------ATGGATTCGGGTCCA---TAGCCATGGGTTACAATGCACGAGATCTTGTAGCAATTACCAATGAGGCCCTATCGATTAGTATTACACAGAAGAAATCAATTATAGACACTAATACAATTAGATTCGCTCTTCATAGACAAACTTGGGAGTTGCGAGCCCATGTAAGACCGGTTCCGGATCATGGGATCCTTTTCTATCAGATAGGAAGG------------GCTGTTGCACAAAATGTACTTATAAATAATTGC---------------------TGCCTTATAGATCCTATATCTATCTATATGAAGAAGCAATCATGTTACGAAGGGGATCCTTATTTGTACAAATGGTTCTTCGAACTTGGAACGAACATGAAGAAATTAACGATACTTCTTTATCTTTTGAGTTGTTCTGCCGGATCGATCGCTCAAGATCTTTGGT---CTCTACCC---------GGACCCGATGAAAAAAATTGGATCACT---------------------TCTTATAGACTCGTTGAGACGGATTCTGATCTAGTTGATGGCCTATTAGAAGTAGTAGAAGGCGCTCTGG----------------TGGGATCCTC------------------------------------------------GCTTCTTCGG-----------------CCCGAACCAAGGAATCCCTTAGAGATGATGGAAAATGGATCTCGTTCTATCTTTGATCGTAGATTTCTCTATGAA----------------------TCGGAGTTTAAAGAATGG------GCAGAAGGCACCGACCCGCAACAG-TTAGCGGAGGATGCAGTCGATCACATAGTTTGGGCTCCTAGAATATGGCAATCTTGGGGCTTTCTATTTGATTG---GATCGAAAGGCCCAATGAATTGGAATTTCCCTATTGGGCCAGGTCATTTCGGGGCAAGCCGATCATTTCT---------------------------------GATGAAG---------------------------------------------------------------------------------------------------------------------------------------------------TTAATGATGAATATTTTGATTATGCATTTTATGGTGAAGGGGATGGTGGAT-ATGAT------------------------------------------------------------------------------------------------------------GAAGAGGATGAGCTTCAAGAGAATGATTGGGAGTTCTTGCAGAGTGAAACCATGGAGTACCC-----------------------------------------------------------------GGGACGAG----------------------------------------------------------------------------------------------------------------------ATAGATCTTCCAAAGAACAAGTCTTTTTT------CGAAAAGGCCAATTCATTTGGGACCCTGGAGATCCACTCTTTTACATATT---------------------------CAACGACGAGCTC---TCTGTCTTTCTGTTTTCACATCGAGAATTCTTTG------------------CAGATGAAGAGATGTCAAAG------------------GGGCTTCTTCTGACTTCCCAA---AGGGAGACTC---------TATATAAACGCGGGT---TTAGCAAGAAACCGAAAGAAAAGTACTTCGAATTTTTTATTAATCGCCA------GAGACGGAGACGGCTTCGAACCATTAGT---TCATTATATAATAGAT---------------------CTTTCCGTTCTAATATTCAATCCGCGAGTTATCAGTACTTATCAAACCTGTTCCTATCTAACGGAAGGCTG-----------------------------------------------------------------------------------------------------------------------------------------------------------------------TTGGATCAAATGACAAAGACATTGTTTAGAAAAAGATGGATTTTCCCGGATGAACTGAAA----------------------------ATTGGATTCATGTAACAGGAGAAAGATTTCCCATTCCGTAGTC-----GTAAAGATATGTGGCCATG-----AAAGAGGGATTAAGTGGAACAGAATTGACT----GG--GCGGTAGAGTCGTGGAAATACT--TG-TTTTTTCCA------TATTTCGGACCTTAGCTCCACGGAACAATA---TGCTACTGCTGAAACATGGAAGAATTGAAATCTTAGAT-----CAAAACACTATGTATGGATGGTATGAATGGCCTAAACAAGAATTCTTGAACAGCGAACA--------------------ACCAGAGCCTAT-TACTCACTACATAA--AAAAATTTCCATTAATGAAAGATGTAAATCCATTGTAAAATAAAAAATACGCATGTCTGATGAAAGTTGCTATCTGTTCCAATAACGAATCATTGGTTTGACTGA------ATAACT--AAAAAAAATACCC----TA-----TAGGGATAATACACATTCCAGTTGACC-----------GAATTGTTTTGTTC-------CGAAGCAAGGGTATCCACGGGGTCGTTCGTCCTATTCATTCAGATATTCACGACCAAGAAGTACTGGA-----TTCTCTTTCGGATAGGCCCC-GAAAGGAGAAGGGAGGCTGGAATGCCAACAGGCGTCTATTCTTGAATTCACCCGACCCGATAGTACCCATTTT------GGGGGGGAACGTCCAGTGCCAAAGTCACTGAATGGGTAAGTCGCCAATCCCTAAAACGGAC-----TATGTAATTTCTCTGCTGAGTTA--CGTTACGGGCGGGCATTTTACCAGAGGTTTCTATTGTATCAATCTACCCTTGTGTGATTCCTGTTGAAGCATATACTC-----------------GGGGGGGTTCAGGGCGGACGATTT-----CAAAGCGGACTCCCCCC------------TC-------ATTAGATAGA-----GAAGATCACCAATATTTCGTGATCCGCTGCCGAACTTATTCCAATTCCAAGA------------------------------------------TCTCTTATTGAATTGCTCATTCAATGAGCATTCTC-----TATGCCTTGAAGAGGACTCGAACCTCCACGCTCTTT-AGCACGAGATTTTGAGTCTCGCGTGTCTACCATTTCACCACCAAGGCATCTTGAAAGTGATTCGTATTCCATGAATATGATATCTATCTAGTGTGATGTATGGAATATATGACAAAGGTGGGGTGTTGGAGTATTGCTATTGATCGGTCATGTCATATAGGCC-----TGAGTCGGACATCCAATTGCTTCGATTTTCATTTTCCGGAGGATGCCCTATACTTATATATGCCCTATACTTA---------TA------------TATATCAAAAAGATGGACAATCAAACTTA-----TTTCTCGATTCAATAGAAGCCCAAA-GAGATGAATAGGGTCCCAAATAAAATAACAAGAGATATGTAAAAAGAAGGTCCGAT------TACGCCTATTCCTAATCCGA-----AATGGAATGTAAGTACGT---AGGGATCCATACGGAA----ATCTA-----------------GTCTTTAT-TTAGATAGGC------TCGAATGACCCC---------------TTCTCATAATGAGAATTTATAGAACCCTCTTCCGGCCTAGTCCGGTATGGAATGAACTTATAATC------------ATGGAATCGACTCGATCATCAGATTCTAGATTATAAGTTCATA-----ACCCTAGCCCATTCCCATTTTGGGCGGAACAGATCTACTAATTCTTTGATTCCAGTTAGTAAGAGGGAT----------GAAATAGACTCTAGAAGCTAAAAAAGGGTATCCTGAGCAATTGCAATAATAGGGTTCATTGATATTCCCGGTATAGTAGATGCTATCACACATACAATCATACTCAATTCGACGGAATTCTTTGATCTTAAAGGAGATCTTCTATAATTTCGCACGTGAGGGGTTATTTCTTGGTTTCGTCCAGTCATTAATAACTTGATTATTTTTAGATAATAGTAGATAGAAACAACGCTCGTAAGGAGTCCTATTGAAACCAAGAAATATAGGCCTGCCTGCCATCCACACCAGAATAAATGGAGTTTTCCGAAAAAACCTGCTAGTGGAGGAAGACCTCCTAGGGATAAGAGACATAGTGCTAAAGAGAGAGCCAAAGGGGGATCTTTCGTGTATAATCCTGCATAATCTCGAATGTTATCAGTTCCGGTACGTAGACCAAATGATACAATGCAAGCAAAAGTTCCTAGATTCATGGAGATATAGAACAGCATATAAGTTATCATGCTTGCATATCCATCATTTGAGTCTCCAACAATTATTCCAATAATTACATATCCGATTTGACCTATGGACGAATATGCAAGCATACGTTTCATGCTTGTTTGAGTAATAGCAATGAGATTCCCCACTATCATGCTAAGAATAGCTAGGATTTCCAGAAGAAGATGCCATTCGTTTGATGAGAAATAAAAAGGAATATCGAAAATTCGAGTGGCTGAAGCTGAAGCAGCTACTTTCGAAGTAACAGAAAGAAAAGCAACGACTGGAGTGGGAGAGTCAGAGTCGAAAAGAGGATTCCTCACTTCTTTCTCTCATTCAAAACCGTGCATGAGACTTTCATCTCGCACGGCTCCTA-AGTGAT------AAAAGAAAGAAGAACTCATCTTCTT--------------TCTTTTTTGATTACCTTCCTCGCGTATGTATAAGACCGAATCCATTCGATTTCTAAAAAGGATTACTAATCCTTAACTTTTCGAGGAATCCTTCATCAGTGGTTGCGAATGACTGATTTTTTCAATCTTTTCGACCTTGGTTCCGTAGGAGCAA-GTCAGAAAGATTGAGAAATAGAACCATCTGATTTGATTCGTTCTCAATAGCCATGAGATGATCATCTTAGGGTGATCCTTTTGTCGACGGATGCTC--CTATTACACTCGTAGTCTCTGAAGGATGAGAACCAACTATGTAGCATCTACATCGAGAATTCAAGTATTGTATACGTCATTAGTCCGACCCTTTGTAGGAACTACCCGTAATAACGAACTTGCAAAATGGATCTGTTTATCATAAAGAGATTCGTTGTT------CCTGACCCTGCTTCACCTTAATTGTTATTTGAACAAGTAAAAGTTATGTCTTGGTCCG--AGTGGGGATAGCATTTCTCTTCTGCATGTCCATGGAGTTTTGAAAAATCCAAACATCTCGGAGATAGATAGAGAGCTAGGAATTTTTCGAACGAACCGCACTCCTTCGTATACGTCAGGAGTCCATTGATGAGAAGGGGCTGGGGAAAGCTTGAACCCAATTCCTACAGTGATGAATATAAGCGCAATTGAAATTCCTGGGGAGTTATACATTTGTGTATTGATAAGACCATTCACTATTTCTTGAAGCTCGATCTCTCCCCCGGATGAACCATATAGCCAAGAGAAACCATGAACCAGAATAGAGGAGCTTGCCCCACCCATGAGTAAATATTTCGTAGTAGCCTCATTAGACCGTACATCTTTCTTGGTATATCCAGATAATAGGTAGGAGCATAAACTGAAACATTCTGGAGCTACAAAGATAGTTATTAAATCGTTAGCACCGCATAAAAACATTCCTCCTAGAGTAGCTGTTAATACGAATAACAGAAACTCTGTTATAGCCATTTCTGTACATTCAATGTACTCTACGGATAGAGGAATACATAGAGTTGAACATAGTAAAATAAGAAATTGAAAGATTTCGTTGAAATTGTTCGTTTGGAAATTTCCCGAAAAGCTAATCATAGGTTCTTCTCTCCATCGGAACAACAGGGCCGTTATGCTCATTACTAAACTTGTTGAAGAGATGAAATAGAACCAAGGTATATCTTTTTGATCAGAGGTTAAATCGATCATCAGAAGAAGAATTAGGCCAAAAATTAGGATACATTCTGGGAAAATAAAACTTCCATCGAAGAGAAGCAAATGAAAGGCTTTCATAAAAATTCTCGTAGAATCGAGAATGAAGTTTTCATTCTGTACATGCCAGATCATGAATTAGTAACTGCATCCAATCTCAAAAAAAATCCCAATTGTTTAGAACTTTCTATTTTTGAAATGGAATATTTACGGAATCTCCACGAATAGGATCCAACCCTATTCCATGG--TATTTCCATGAGATTCCTCTTGC-TTATTTTTTCTTAAGCAAGTCCCCGAGAGGGCTTAGTTGATCCATGATTTATCTTTCATCTTTCGTTTCCTTTTCGTTTGTTTC-------GAAATAAATATATCG-----ATCAATTCCGATTCTTTCTTTTTCTATTGATTCTTTTCCGATCGAGATGTATGGATCCATGGATCTAGGTGTCTACTATATAGATAGATCCTGTTCA---------------TGGATTAACGAAAATGTGCAAAAGCTCTATTTGCCTCTGCCATTCTATGAGTCTCTTCCTTTTTGCGTATGGCACCGCCACTCCCTTTGGCAGCATCCACTAATTCGGAACTTAATTTGAAAGCCATATTTCGACCCGGACGTTTTCGGGATTCCCCTAATAACCAACGAATGGCAAGTGCTTTTCCTTGTGTGGATCCTATTTCAATGGGAACTTGATGAGTCGATCCGCTTACACGTCTTGCTTTTACTGCTATATCGGGAGTTACTCCACGTATTGCTTGACGTAAAACAGATAGTGGATTTTTTTCTGTCTCTTGTTGAATCTTTTTCACGGCTCGATAGATAATTTGATAAGCCAATGATTTTTTTCCGTGTTTCAGAATACGGTTAACCAACATGTTAACTAATCGATTACGATAAATTGGATCGGATTTTGCAGTTTTTTCTTCTGCAGTACCTCGACGTGACATGAGCGTGAAAGGGGTTCAAGAATCAGTTTTCTTTTTATAAGGGCTAAAATCACTTATTTTGGCTTTTTGACCCCATATTGTAGGGTGGATCTCGAAAGATATGAAAGATCTCCCCCCAAACCGTACATACGACTTTCATCGAATACGGCTTTCCACAGAATTCTATATGTATCTATGAGATCGAGTATGGAATTCTGT-------------TTACTCAC------TTTAAATTGAGTATCCGTTTCCCTCCTTTTCCTGCTAGGATTGGAAATCCTGTATTTTCCATATCCATACGATTGAGTCCTTGGGTTTCCGAAATAG-----TGT-------AAAAAGAAGTGCTTCGAATCATTGCTATTTGACTCAGACCTGTTCTAAAAAAGTCGAGGTATTTCGAATTGTTTGTTT--A--CA-CGGACAAAGTCAGGGAAAACCTCTGAAATTA---------TGGACCTTGGACATATAATAGTTCCGAATCGAATCTCTTTAG-AAAGAAGATCTTTTGTCTCATGGTAGCCTGCTCCAGTCCCCTTACGAAACTTTCGTTATTGGGTTAG------CCATACACTTCACATGTTTCTAGCGATTCACATGGCATCATCAAATGATACAAGTCTTGGATAAGAATCTACAACGCACTAGAACGCCCTTGTTGACGATCCTTTACTCCGACAGCATCTAGGGTTCCTCGAACAATGTGATATCTCACACCGGGTAAATCTTTAACCCTTCCCCCTCTTACTAAGACTACAGAATGTTCTTGTAAATTATGGCCAATACCGGGTATATAAGCAGTGATTTCAAATCCAGAGGTTAATCGTACTCTGGCAACTTTACGTAAGGCAGAGTTTGGTTTTTTGGGTGTGATAGTGGAAAAGTTGACAGATAAGTCACCCTTACTGCCACTCTACAGAACCGTACATGAGATTTTCACCTCATACGGCTCCTCGTTCAATTCTTTCGAAGTCATTGGATCCCTTCCCTTGTTCGAGAATCTCCTCCCTTCTTCCACTCCGTCCCGAAGAGTAACTAGGACCAATTCAGTCATGTTTTCATGTTTCAATTGAACACGTTC-CACTT------------------TTAATTATTCT-----C---------GAGAAGATTCTTCTTTTT-----------------------ACCAAACATATGCAG-------------------ATCCAATCACGATCTTCTAATAAGAACAAGAGATCTTTCTCGATCAATCCCCTTGCCCCTCATTCTTCGAGAATTAGAAGGATCCTTTTCAAGTTTGAATTTGTTTTCATTTGGAATCTGGGTTCTTCTACTTTTTAC----------------------TTTTTTTCTATTTTTTCCCTCTC-TTTTTTTATTCCC-----TTCCATAAT-----TCCTTAAGTCTCATAGGTTTGATCCTGTAGAATCTGACCCATTTTCTCATTGAGCGAGGGGTACGAAATAAATCAGA-TTGATTTTCATTTTTC-----GATCAAAAGTACTATGTGAAATCTTCGGC-TTTCCTCTTCCTCTATTCCTATCCCATAGGTACAGC-----GTTTAAATCAATAGAGAAC-----TTTCTGTATGAATCGATATTATTACATTCCATCCAATTCCTTCCCGAAACCTCCCAAGGAAAATCCCGAATTGGATCCC------AAATTGACGGGTTAGTGTGAGCTTATCCATGCGGTTATGCACTCTTCGGATAGGAATCCTTTTTTTGAAAGATCCTGGCTTTCGTGCTTTGGTGGGTCTCCGAGATCCTTTCGATGACCTATGTTGTGTTGAAGGGATATCTATGTGATCCGACCGATTGCGTAAAGCCCGCGGTAGCAACAGAACCGGGGAAAGTATACAGAAAAGACAGTGCTTTTCTATTATATTAGTATTTTCTATTAG-ATT-------------AATTAGATTAGTC---------TTGGTTAGTGATCCCGGCTCAGTGAGTCCTTTATTCCGTGATGAACTGTTGGCACCAGTCCTACATTTTGTCTCTGTGGACCGAGGGGAAAGGGGACTCGTCGGGAAGAGGATTGTACC-----AGAAGCAAGGAGGTCAACCTCTTTCAAATATACAACACGGATTCTGGCAATGCAACGTAGT-----TCTCATGTCGATCCGAATGAATCATCCTTTCCACGTAGGTAAATCTTTGCCTGCTAGTCAAGAGGGTAGCAAGTTACAAATTCTATCTCGGTAGGACATGTATTTCT------------------ATTACTATGAAATT-----------CATA-AATTAAATGA------------AGTAGTTAAGTTAATGGTGGGGTTACCATTATCCATTTTGGAGTGACGAATCCTGTATGTGTTCCTAAGAAAAGGAATTTGTCCATTTTTCGGGGTCTCAAAGGGGCGTGTAAACACATAAGAACTCTGGA---------------------------------------------------------------------------------------------------------------------------------------------------------------------------------------------------------TTAGATCGGTAAAAGCGTACGGTTTTATGAAACCATGTGCTATGGTTCGAATCCGCAGTCAATCCGATTTCCGATAGGAGCAGTTGACAATTGAATCCAAATTTTTCCATTCTTTTCATATCCGTAATAGTGCGAAAAGAAGGCCCGGCTCCAAGTTGTTCAAGA-ATA------GTAGAATAGTGGCGTTGAGTTTATCGACCCCTTGCCTTAGGATTAGTCAGTTCTATTTC----TGGGGGCAGGGAAGGGATATAACTCAGCGGTAGAGTGTCACCTTGACGTGGTGGAAGTCATCAGTTCGAGCCTGATTATCCCTAAACCCAATGTGAG-----TTTTTCTGTTTTGGCTTGCCCCCCCGCC--GTGATTGAA-----------AGAGAATGGATAAGAGGCTCGTGGGATTGACGCG---------------------------------------AGGGGCTAGGAATGGCTATATTTCTGGGAGCGAACTCCGTGCGAATAGGAAGCGCATGGATACAAGTTATGCCTTGGAATGAAAGACAATTCCGAATCTGCTTTGTCTACGAACAAGGAAGCTATAAGTAATGCAACTATGAATCTCATGGAGAGTTCGATCCTGGCTCAGGATGAACGCTGGCGGCATGCTTAACACATGCAAGTCGGACGGGAAGTGTTGTTTCCAGTGGCGGACGGGTGAGTAACGCGTAAGAACCTGCCCTTGGGAGGGGAACAACAGCTGGAAACGGCTGCTAATACCCCGTAGGCTGAGGAGCAAAAGGAGGAATCCGCCCGAGGAGGGGCTCGCGTCTGATTAGCTAGTTGGTGGGGCAATAGCTTACCAAGGCGATGATCAGTAGCTGGTCCGAGAGGATGATCAGCCACACTGGGACTGAGACACGGCCCAGACTCCTACGGGAGGCAGCAGTGGGGAATTTTCCGCAATGGGCGAAAGCCTGACGGAGCAATGCCGCGTGGAGGTAGAAGGCCCACGGGTCGTGAACTTCTTTTCCCGGAGAAGAAGCAATGACGGTATCTGGGGAATAAGCATCGGCTAACTCTGTGCCAGCAGCCGCGGTAAGACAGAGGATGCAAGCGTTATCCGGAATGATTGGGCGTAAAGCGTCTGTAGGTGGCTTTTTAAGTCCGCCGTCAAATCCCAGGGCTCAACCCTGGACAGGCGGTGGAAACTACCAAGCTGGAGTACGGTAGGGGCAGAGGGAATTTCCGGTGGAGCGGTGAAATGCGTAGAGATCGGAAAGAACACCAACGGCGAAAGCACTCTGCTGGGCCGACACTGACACTGAGAGACGAAAGCTAGGGGAGCGAATGGGATTAGATACCCCAGTAGTCCTAGCCGTAAACGATGGATACTAGGCGCTGTGCGTATCGACCCGTGCAGTGCTGTAGCTAACGCGTTAAGTATCCCGCCTGGGGAGTACGTTCGCAAGAATGAAACTCAAAGGAATTGACGGGGGCCCGCACAAGCGGTGGAGCATGTGGTTTAATTCGATGCAAAGCGAAGAACCTTACCAGGGCTTGACATGCCGCGAATCCTCTTGAAAGGGAGGGGTGCCTTCGGGAACGCGGACACAGGTGGTGCATGGCTGTCGTCAGCTCGTGCCGTAAGGTGTTGGGTTAAGTCCCGCAACGAGCGCAACCCTCGTGTTTAGTTGCCAACGTTGAGTTTGGAACCCTGAACAGACTGCCGGTGATAAGCCGGAGGAAGGTGAGGATGACGTCAAGTCATCATGCCCTTTATGCCCTGGGCGACACACGTGCTACAATGGCCGGGACAAAGGGTCGCGATCCCGTGAGGGTGAGCTAACCCCAAAAACCCGTCCTCAGTTCGGATTGCAGGCTGCAACTCGCCTGCATGAAGCCGGAATCGCTAGTAATCGCCGGTCAGCCATACGGCGGTGAATTCGTTCCCGGGCCTTGTACACACCGCCCGTCACACTATGGGAGCTGGCCATGCCCGAAGTCGTTACCTTAACCGCAAGGAGGGGGATGCCGAAGGCAGGGCTAGTGACTGGAGTGAAGTCGTAACAAGGTAGCCGTACTGGAAGGTGCGGCTGGATCACCTCCTTTTCAGGGAGAGCTAATGCTTGTTGGGTATTTTGGTTTGACACTGCTTCACACCCAAAAAGAAGCGAGCGACGCCTGGG---TGAAA-----CTTGGAGATGGAAGTCTTCTT---------TCGTTTCTCGACGGTGAAGTAAGACCAAGCTCATGGGCTTATTATCCTAGGTCGGAACAGGTTGATAGGAT--CCCCCCC-TTTTCGCCCCCATGTCGCCAC--------AC--GGGGGGGCATGGGGACG-----TCAA-AAAGAAAGAGAGGGATGGGGTTTCTCTCGCTTTTGGCATAGCGG------GCCTCCCAC-----TGGGGGCCCGCACGACGGGCTATTAGCTCAGCGGTAGAGCGCGCCCCTGATAATTGCGTCGTTGTGCCTGGGCTGTGAGGGCTCTCAACCACATGGATAGTTCAATGTGCCCATCCGCGCCTGACCTTGAGATGTGGATCATCCAAGGCACATTAGCATGGCGTACTCCTCCTGTTCGAACCGGGGTTTGAAACCAAACTTCTCCTCAGGAGGATAGATGGGGCGATTCAGGTGAGATCCAATGTAGATCCAACTTTCGATTCACTCGTGGGATCCGGGCGGTCCGGGGGGGACCACTATGGCTCCTCTCTTCTCGAGAATCCATACACCCCTTATCAGTGTATGGACAGCTATCTCTCGAGCACAGGTTTAGGTTCGGCCTCAATGGGAA---AAAATGGAGCACCTAACAACGCATCTTCACAGACCAAGAACTACGAGATCACCCTCTTCATTCTGGGGTGACGGAAGGATCGTACCATTCGAGCC---TTTTT--TTCATGC-TTTCCCGAAGGTCTGGAGAAAGCTGCAATCAATAGGATTTCCCTAATCCTCCCTTCG-----CGAAAGGC-----AGAACTTTAAA--------------------AGAACTGTAAATTCTTTTCCCTTTCCGCAGGGACCAGGAGATTGGATCTAGCCGTAAGAAGAATGCTTGGTATAAATAACTAACTTCTTGGTCTTTGACCCCCTCAGTCACTACGAACGCCCCCCGATCAGTGCAATGGGATGTGTCTATTTATCTATCTCTTGACTCGAAATGGGAG--CAGGTTTGAAAAAGGATCTTAGAGTGTCTAGGGTTGGGCCAGGAGGGTCTCTTAACGCCTTCTTTTTTCTTCCC----GG----------GTTATTTCACAAAGACTTGCCATGGTAAGAAGTAATAAGGAGCGAACAAGCACACTTGGAGAGCGCAGTACAACGGAGAGTTGTATGCTGCGTTCGGGAAGGATGAATCGCTCCCGAAAAGGAATCTATTGATTCTCTCCAAATTGGTTGGACCGTAGGTGCGATGATTTACTTCACGGGCGAGGTCTCTGGTTCAAGTCCAGGATGGCCCAGCTGCGCCAGGGAAAAGAATAGAAGAAGCATCTGACTCCTTCTCATGCATGCTCCACCTGGCTCGGGGGGATATAGCTCAGTTGGTAGAGCTCCGCTCTTGCAATTGGGTCGTTGCGATTACGGGTTGGATGTCTAATTGTCCAGGCGGTAATGATAGTATCTTGTACCTGAACCGGTGGCTAACTTTTTCTAAGTAATGGGGAAGAGGACCGAAACATGCCACTGAAAGACTCTACTGAGACAAAGATGGGCTGTCAAGAACGTAGAGGAGGTAGGATGGGCAGTTGGTCAGATCTAGTATGGATCGTACATGGACCGTAGTTGGAGTCAGCGGCTCTCCTAGGGTTCCTTAATCTGGGAT-CCTGGGGAA-GAGGATCAAGTTGGCCCTTGCGAACAGCCTGATGCACTATCTCCCTTCAACCCT---TTGAGCGAAATGCGGCAAAAGGAAGGAAAATCCATGGACCGACCCCATCGTCTCCACCCCGTAGGAACTACGAGATCACCCCAAGGACGCCTTCGGCATCCAGGGGTCACGGGCCGACCATAGAACCCTGT-------TCAATAAGTGGAACGCATTAGCTATCCGCT------------------------------------------------------------------------------------------------------------------------------CTCCGGTTGGGCAGTTAAGGGTCGGAGAAGGGCAATCACTCATTCTTAAAACCAGCATTCTTAA----GAC----CAAAGAGTTGGGCGGAAA--GGGGGGTAAAGCTCTCCGTTCCTGGTTCTCCTGTAGCTGGATCCTCCGGAACCACAAGAATCCTTAGTTTGAATTGGATTCCAACTCAGCACCTTTTGA----GATTTTGAGAAGAGTTGCTCTTTGGAGAGCACAGTACGATGAAAGTTGTAAGCTGTGTTCGGGGGGGAGTTATTGTCTATCGTCAGCCTCTATGGTAGAATCAGTC-GGGGGCCTGAGAGGCGGTGGTTTACCCTGTGGCGGATGTCAGCGGTTCGAGTCCGCTTATCTCCAACTCGTGAACTTAGCCGATACAAA-----GCTATATGATAGCACCCAA-TTTTCCGACTCGGCGGTTCGATCTATGATTTATCATTCATGGACGTTGATAAGATCCTTCCATTTAGCTTAGCAGCACCTTAGGATGGCATAGACT-----TCAAGTTAAGGGCGAGGTTCAAACGAGGAAAGGCTTACGGTGGATACCTAGGCACCCAGAGACGAGGAAGGGCGTAGTAAGCGACGAAATGCTTCGGGGAGTTGAAAATAAGCATAGATCCGGAGATTCCCGAATAGGTCAACCTTTCGAACTGCTGCTGAATCCATGGGCAGGCAAGAGACAACCTGGCGAACTGAAACATCTTAGTAGCCAGAGGAAAAGAAAGCAAAAGCGATTCCCGTAGTAGCGGCGAGCGAAATGGGAGCAGCCTAAACCGTGAAAACGGGGTTGTGGGAGAGCAATACAAGCGTCGTGCTGCTAGGCGAAGCGGTGGAGTACTGCACCCTAGATGGCGAGAGTCCAGTAGCCAAAAGCATCACTAGCTTACGCTCTGACCCGAGTAGCATGGGGCACGTGGAATCCCGTGTGAATCAGCAAGGACCACCTTGCAAGGCTAAATACTCCTGGGTGACCGATAGCGAAGTAGTACCGTGAGGGAAGGGTGAAAAGAACCCCCATCGGGGAGTGAAATAGAACATGAAACCGTAAGCTCCCAAGCAGTGGGAGGAGC-ACATAGGGCTCTGACCGCGTGCCTGTTGAAGAATGAGCCGGCGACTCATAGGCAGTGGCTTGGTTAAGGGAACCCACCGGAGCCGTAGCGAAAGCGAGTCTTCATAGGGCAATTGTCACTGCTTATGGACCCGAACCTGGGTGATCTATCCATGACCAGGATGAAGCTTGGGTGAAACTAAGTGGAGGTCCGAACCGACTGATGTTGAAGAATCAGCGGATGAGTTGTGGTTAGGGGTGAAATGCCACTCGAACCCAGAGCTAGCTGGTTCTCCCCGAAATGCGTTGAGGCGCAGCAGTTGACTGGACATCTAGGGGTAAAGCACTGTTTCGGTGCGGGCCGCGAGAGCGGTACCAAATCGAGGCAAACTCTGAATACTAGATATGACCTCCAAAT---------------------AACAGGGGTCAAGGTCGGCCAGTGAGACGATGGGGGATAAGCTTCATCGTCGAGAGGGAAACAGCCCGGATCACCAGCTAAGGCCCCTAAATGACCGCTCAGTGATAAAGGAGGTAGGGGTGCAGAGACAGCCAGGAGGTTTGCCTAGAAGCAGCCACCCTTGAAAGAGTGCGTAATAGCTCACTGATCGAGCGCTCTTGCGCCGAAGATGAACGGGGCTAAGCGATCTGCCGAAGCTGTGGGATGTAAAAATGCATCGGTAGGGGAGCGTTCCGCCTTAGAGGGAAGCAACGGCGCGAGCCGCGGTGGACGAAGCGGAAGCGAGAATGTCGGCTTGAGTAACGAAAACATTGGTGAGAATCCAATGCCCCGAAAACCTAAGGGTTCCTCCGCAAGGTTCGTCCACGGAGGGTGAGTCAGGGCCTAAGATCAGGCCGAAAGGCGTAGTCGATGGACAACAGGTGAATATTCCTGTACTACCCCTTGTTGGTCCCGAGGGACGGAGGAGGCTAGGTTAGCCGAAAGATGGTTATCGGTTCAAGGATGCAAGGTGACCCTGCTTTTTCAGGGTAAGAAGGGGTAGAGAAAATGCCTCGAGCCAATGTTCGAGTACCAGGCGCTACGGCGCTGAAGTAACCCATGCCATACTCCCAGGAAAAGCTCGAACGACCTTCAACAAAGGGGTACCTGTACCCGAAACCGACACAGGTGGGTAGGTAGAGAATACCTAGGGGCGCGAGACAACTCTCTCTAAGGAACTCGGCAAAATAGCCCCGTAACTTCGGGAGAAGGGGTGCCTCCTCACAAAGGGGGTCGAAGTGACCAGGCCCGGGCGACTGTTTACCAAAAACACAGGTCTCCGCAAAGTCGTAAGACCATGTATGGGGGCTGACGCCTGCCCAGTGCCGGAAGGTCAAGGAAGTCGGTGACCTGATGACAGGGGAGCCGGCGACCGAAGCCCCGGTGAACGGCGGCCGTAACTATAACGGTCCTAAGGTAGCGAAATTCCTTGTCGGGTAAGTTCCGACCCGCACGAAAGGCGTAACGATCTGGGCACTGTCTCGGAGAGAGGCTCGGTGAAATAGACATGTCTGTGAAGATGCGGACTACCTGCACCTGGACAGAAAGACCCTATGAAGCTTCACTGTTCCCTGGGATTGGCTTTGGGCCTTTCCTGCGCAGCTTAGGTGGAGGGCGAAGAAGGCCCCCTTCCGGGGGGGCCCGAGCCGTCAGTGAGATACCACTCTGGAAGAGCTAGAATTCTAACCTTGTGTCAGGACCCACGGGCCAAGGGACAGTCTCAGGTAGACAGTTTCTATGGGGCGTAGGCCTCCCAAAAGGTAACGGAGGCGTGCAAAGGTTTCCTCGGGCCGGACGGAGATTGGCCCTCGAGTGCAAAGGCAGAAGGGAGCTTGACTGCAAGACCCACCCGTCAAGCAGGGACGAAAGTCGGCCTTAGTGATCCGACGGCGCCGAGTGGAAGGGCCGTCGCTCAACGGATAAAAGTTACTCTAGGGATAACAGGCTGATCTTCCCCAAGAGATCACATCGACGGGAAGGTTTGGCACCTCGATGTCGGCTCTTCGCCACCTGGAGCTGTAGTATGTTCCAAGGGTTGGGCTGTTCGCCCATTAAAGCGGTACGTGAGCTGGGTTCAGAACGTCGTGAGACAGTTCGGTCCATATCCGGTGTGGGCGTTAGAGCATTGAGAGGACCTTTCCCTAGTACGAGAGGACCGGGAAGGACGCACCTCTGGTGTACCAGTTATCGTGCCCACGGTAAACGCTGGGTAGCCAAGTGCGGAGCGGATAACTGCTGAAAGCATCTAAGTAGTAAGCCCACCTCAAGATGAGTGCTCTCCTATTCCGACTTCCCCAGAGCCTCCGGT-----AGCACAGCCGAGACAGCGACGGGTTCTCTGCCCCTGCGGGGATGGAGCGACAGAAGTTTTGAGAATTCAAGAGAAGGTCACGGCGAGACGAGCCGTTTATCATTACGATAGGTGTCAAGTGGAAGTGCAGTGATGTATGCAGCTGAGGCATCCTAACAGACCGATAGACTTGAACCTTGTTCCTACATGACCCGATCAATTCGATCAGGCACTCGCCATCTATTTTCATTGTTCAACTCTTTGACAATACGAAAAAACCATTATTCAACTCTTTGACAACATGAAAAAACCAAAAGCCCTGCCCT---TCCACCCCTTGGTTAGATATAGAGGGAGGGCAGAGGCCTTTGGTGTCCCCTCCAGTCAAGAATTGGGGCCTCACAATCACTAGCCCA-----TTTTCTCTCATGCCT-------T-----TCTTAGTTCGTGGTTCGATATTCTGGTGTCCTAGGCGTAGAGGAACCACACCAATCCATCCCGAACTTGGTGGTTAAACTCTACTGCGGTGACGATACTGTAGGGGAGGTCCTGCGGAAAAATAGCTCGACGCCAGGATGATAAAAAGCTTAACACCTCTCATTCTTATTAC-------TTTTTCAATAT---GA------------AAGAAAAAAAATGAAAAAAGGAAAAGGTCGTCTTATT------CAAAACCCCAATTATGACATCCCCTCTCTCCC-------------------------------------------------------------------ACTTCACACCTCGGAACGCACCGTTCTTATAGATAGAAAC----------GCGCTTTCACATCTTCTTAACCG---------G---AAA--TGGCTGGGGAGAGGAAAGGTTCCT-------TTTT-TTTTAGGATACTCCCGCTCCCGGGAACAGATCCAGTGGAGGCAGGGTGGGGCCTGTAGCTCAGAGGATTAGAGCACGTGGCTACGAACCACGGTGTCGGGGGTTCGAATCCCTCCTCGCCCACAACCTTCCCC------TTTTGGAAGGACCTATCCCTCCGGGGATAGGAAAATCATGATCGGGATAGCGGACCCAAAGCTATGGAACTTGGGTGTGGGTCTTTTGTCGA-----AATGGAATGGGCCTTACC------TTTTTTTTACGTGTTATCGTGAATGAGGTAATTTTAA-----------AGTATAGCATGCC-------CCGGCCGGCAGCATA--CCT----TTTTGTTTTACGTCCCGTA----ACTCTTCCTCAGCCAGGCTTGGGCAGAATAGCAGAGCAAGTACAAGTATTAGTAGCATAGCAAAAAAGCGTTCCTCGTCAT-----TAATATGTTTTCTGTTTGCTCGCGGTAATT-----GTAGCCTATCGGGAGAGTCGATGACTCCATCTT--------TGATGCACTCCT---AGTACT----AGTACATCCGAGAATTATGAATTGGCTAGTTGTAAATAGCCCCAGGACTATGGAACAAAGGATTATCCCGGACCTACATCGAGGTATTGACGGAGATTCTCAAATATCGCAGAACAGGATGTCATACGATGAGATAGAATACAATAGAAACAAAGACAGGGAACGGG-----------TACTCTTAACGGTCAAAGTGAGCCCTTTCAT-------------TCTCATTCTGAATTCTTTAA-TTCAGAAT--GAATCAAATCTCCCCAAGTAGGATTCGAACCTACGACCAGTCAGTTAACAGCCGACCGCTCTACCACTGAG-CTACTGAGGAACAACGGGGGATTTTATCTCATAGAGTGAAATTCCCGTTCTCAACCCATGACCCATATGAGCTCGAAGCTTCCTTCGTAACTCCCAAAACTTCTTCGTAGCGGCTCCCTTCCATGCC-------------TCATTTCATAGGG-----AACCTCAAAGTGGCTCTATTTCATTATATTCCATCCAT----------------------------ATCCCAATTA---------------------------------CATTTATTTCATATCTCTTTTGTGTCATTGACATAA-----------------------------------GAGATCTCGTTTCTGGTCTATCTCTTTCTATTT----------------------------------CTATCTAGAAT--------------AAAATAAGTTAAACAATTATCTTATTCTTATAATAA----------------------TTATCATATAAGATAAGAAGGTAAAAACTTATCATATA-------TAATAGAATCGTATAGAATATA--------------------ATTAATATA-ATAATAAT-----------------AG-------------------------------AAATAGAATAA-----TAAT------ATAAAA-------------------------AATAGAATTTTATAATTTT-------------ATAAGGTCAAATA------ATAAGGTCAAAGATTATC--------TTATAATA-----AGAAGGTCAAAATT------------------------------------------------TATTATATAAT-----------------------------AATCATAATTCAGAAATTGCA----------------------------------------------------------------------------------------------------------------------------------------------------AAATCACAAAGGGGGTTTGTCATGATTTT------TTTTCTACTAGGTAA---------CTTATGCATGAAGATAGTCAATTCGGTCGTTGTGGTCGGACTCTATTATGGATTTCTGACCACATTCTCCATAGGGCCCTCCTATCTCGTCCTTCTCCGAACTCTGGTTATGGA------AGAAGGAGAAGA---------AGGAACCGAGAAGACGGTAGCAGCAACAACAGGTTTTATTATGGGACAGCTCATGATGTTCATATCGATCTATTATACGCCTCTGCATCTAGCATTGGGTAGACCTCATACAATAACTTTCCTAGCTCTACCTTATCTTTTGTTTAATTTCTTCTGGAGCAATCA---------TTTTGATTATGGATCTACTACCAG---------AAATTCAATGCGTAATCTCAGCATTCAATGTGTATTCCTGAATAATCT---------------------------TATTTTTCAATTATTCAACCATTTCGTTTTACCAAGTTCAATGTTAGCCAGATTAGTCAACATTTATATGTTTCGATGCAACAACAAGATGTTATTTGTAACAAGTAGTTTTGTTGGTTGGTTAATCGGTCACATTTTATTCATGAAATGTGTTGGCTTGGTATTAGTCTGGATAC------------GGCAAAATTTTTATATTCGATCGATTATTCGATCGAATACGTACCTTC---------------------TTAATGTACTTATTCG-AGCTATTAATGTACC------------------------------------------------------------------TATTCGATCGAA------------TAAGTACCTTAATGGAATTATTC---------GATCTAAGAGGTATAAGAAGTA---------------------------------CAAGGACTCTGTGTCAG---------------------AATTGAAGGACCTTGTGTCAGAATTGAAGTACTTTGTGTTAGACTTGATAGATTCTAT---------------------GGATCGAATC------------------------------------------------------------------------------------------------------------------------------------------------------------------------------------------------------------------TTTAGTATTCTCTTATTTATTAGCTGTGTCTACTCTTTAGGCAGAATGCCGTCACCCAT------TTTTAGTAGGAAACTGCAAGAAACC-------------------------------------------------TCAAAAACGAC-------------------------AGAAAGAGATGTAGAAATAGAAACAACTTTCGAAACGAAAGGGACTAAACAGGAACAAGAGGGATTCACCGAAGAAGATCCTT------------------------------------------------------------------------------------------------------------------------------------------CTCTTTCCCTTTTTTCGGAAGAAAGGGAGGATCCGGACAAAATCGATGAAACGGAAAGGATCCGAGTGAATGGAAAGGACAAAACAAAGGATGAATTCCACTTTCACTTAAAAGAAGAAGAT-------AAAGACCTCTTCTGGTTTGAAAAACCCCCTGTGAGTCTTCTTTTCGACTATAAACGATGGAATCGCCCATTGCGATATATAAAAAATTATAGATTCGAACATGCTGTAAGAAATGAAATGTCACAATATTTTTTTTATACATGTCAAAGTGATGGAAAACGAAGAATTTCTTTTACATATCCATCCAGTTTATCAGCTTTTTTTGAAATGCTACGACAAAAAATGTATTTTTATTTTTTTACAACAAAAAAATTTG---------TCTGTGATGAACTGGATAAATTCTTCTATGATGAACTGCATAATTATTATTGTTGGATTTATACCAATGAAAAAAAATGGAGGAGCCTAAGGAACGAGTTTAGAGATCGAATTGAAGCCCTAGACAGAGGATCTCCTTATCTGGATGTACTCGAAAAAAAGACTCGATTATGCAATAATA---------------------------------------------------------------AAACTAAAGAAGAATACTTGCCTAAAATATATGATCCTCTCTTAAACGGATCCTATCGTGGAATAATTAA------AAAATTTTTTTTACCTTCAAT-----------------------------------------------------CCTAAATGAAACTGCAGTCAAAAATTC------------------------------------------------------------------------------------------GATAGAGACAAATTTTATAAATAAAC---------------------------------------TCAATAAAGTTCATAGTATCCTTCTTTTTAAGGGTAA-----GGAACTT--AATGTTCATAA-------------------------TTTTGAAGAATTGTACC-------AGAAATTGGAAGGG------------------------------------------------------------------------AAAATAGCTACATTGGAAGAGAAATT----GGTCCAGAAAT-----------------TGGACC------------AGAAATTGGAAGATAAATTGGGACAGAAAATATATACATTGGATAAAAAATCATTAGCAAGAGAATTGAGTCTTTTAATC-----GATGAATTTGCT------------------GAAGAATCTACATCAAATTTGAA--AGGAATTTCTTTATTTCCGGA--------------------------ACAAAGACGAATTGATTCAGACGAT---CCAGAAAAAGTTTTGAAATTTTTAATCGAAAGAG-------------------------------------------TCATAATTGATCCCA-TCATTCAAACAATATGCGATGCAGCCATAATTCCTCCCATGGAAAAAACAAC------------------------TCGAAAAAAATCGATTGGAATAA---------ATAAAAAAGTCCCCCGATGGTCATACAAATTATTCAGCGAGGTAGAACAACTC----GGAAAAACTGCAACAAC----GGAAGAGGGGGAAGAGTGGATAGTAGATCATCAAATTCGCTCGAGGAAAGCGAAACGTATAGTTCTTTTTACGCAGGGTC---------------------------------CAGAGAATGCCGAC---------------------------------CCTAGTATCAAAGCTATGACGAAGCCTGAT------------------------------GAAATAGAAGAA---------GTGGATATGATAGA---------------------------TTATCCCTATGAAGCGGATTTTCGGCGAGACATAATCACAGGTTCTATGCGTGTTCAAAGACGTAAAA---CCCTTACGGGGAAAATGTTTTTCTTATATCCGTATTCCCCACTTTTTTTCGACAGAGTA--GGGTTTTATT-------GGGATGTTCTTTTCGAACCATTCA------TATTATCAGTAATTGAG---ATTTCCGACCTAATACAAGACATTTTTAGAAA---------------------------GGGTATAAAAGGAAG---------------------------CGTAGCAAAAAGAACAAAGAGGTTGAAAAAAAAAA-----------AAAAAATGTACA-------------TGGAGGAAAACAAAAGCTACGAAGAAATTAA---AAAAGAAGTCAATGAAATGGAAAAGGGGCGGGACGGGCAGACGGAAATGCAACGAATAGAAA-------GGACACGAGAA------------------------------------------------------------------------------------------AAAATATCAGACCTCTATGATATCCTTATTTATGCTCATGGAATAAGAGCTTTTATTTTACTAATTCAGTCGAGGCTTAGACAATCTATTGTATTACCTTCATTGATACTAGCTAAAAATATTGTCCGTTTCTTATTACGCCAAGAGTCCGAGTGGGAGCAGGATATAAGGGAGATGAATAGAGAAGTGTATGTTATATGCACCTATAATGGTATGCCAGTACTAGAACCAGGAAGAAACGGAATTTTTCCTCCAAACTGGGCTACAGAGGGT------ATACAAATAATGATACGATTTCCTTTCCGTCTTAAACCTTGGCACCGATCTAAGATACG---------------------------------------------------------------------------------------------------------------------------------------------------------------------------------------------------------------------------------------------------------------------------------------------------------------------------------------------ACCTTCTCGTAGGGATCCAAATCCAAAGCAGGAAAG---------------------------------------------TCCTGCTGCTTTTTTAACGATTTGGG---GACTGGAAACTGACCGTCCTTTTGGTTCTCCTCTCGTAGGACTTGGTATTTTGTTTTGTTATTATTTTGGACCCCCTTTGAAAAAACTCCAAAAAACAATTATAAAATGGAGTTTTCGAGTTCTAAAAAGTTTCAAAGAAAGAACAAAATTCTTGTTTCTAAAGGTCCAAA---------AAGAACCAAAAAAATTGAGAGAGGATTCGAGTGAAATAAAAAAAGATTCTATAATCAATAA---------------------------TCAGATTATTCATGAATCATCCATTCAAACCCGATCTATGGATTGGACAAATTATTC-------------------------------------------------------------------------------ACTGACAGAAAGAAAAATGAAAGAT-----------------------------CTGACTGATAGAACAAGCACAATCAGAAATCAAATAGAAAGAA------------------------------TTACAAAAGACAAGAAAAATGGATTTCAAACTCTAAAGAGAAATATTAGTCCTAACAAAACAAGTTATGGTGCTCAAAAATTAGCATCACT------AAAAAATATTTTTCAGATATTAAAAAGAAGAAATGATCGATTAATCCGTAAATCACATTATTTTCGAAAATGGATCGTGGAAAGGATATACACGGATATCCTTCTAGAGAGCTTTCCATATATC------------------ATTAAT------------CGTCTTACTAATAGTCTTTATAGGCCCATGATCATTATAAAACTTTTTCGTAAATTAAAAAAAAAATCTTTTTTTGATACAA---AAGAGAAAAAGATAATTGAGCGTATTTCAACTATACAAAAAAAACTTTCACCTTCTCGTATTCGTCAT-------------------AAGATTCAGA------------------CGAAGTCGGAGGTTTCTTTTAAATTATCCCTCGTGTCACAGGCCTATGTATTTTACAAATTATCACAAACCCAGGTTATTAACTTGTATAAGTTAAGATCTGTCTTTCAAT------ATGACGGAGCATCTTTATTTCTTAAGAATGAAATAAAAGATTATTTTCGAAGACAAGGAATAATTTCTTCCGA------------------------------------------------ATTAAAGCATAAGAAACTTCAGAATTCTGGAATGAATCAATGGAAAAATTGGTTAAA---------------------------------------------------------GAGTCATTATCCATACGATTTATCTGAGCTAAAATGGTCTAAATTAGTACCGCAAAAATGGCGAAATAGAGTCAATCAACATTGTAGGGTTGAAAATAAAAATTTAATCAAACG------GGATTC------ATCTGAAAGAGAGGAAAAGGTTTCGTTATTGCTAAATAAAAATGATCATTTAAAAAAAATGTATAGATATGATCTTTTAGCATATCAATCGATTTATTATGAAGATAAGAAGGACTCATATAATTA------------------------------------------------------CAACATACATAAACCGAAATTTGTTGATATGGGGGGGAGTATCCCTATTACTAATTTTATAAGAAAAGATTATT----TTATGTATATAAAAAAT--CCAGATAGA----AAATTTTTTGATACGAAAGGTCTT------TTTTATCTCAAAATTA------------------------------------------------------------------------ATAAAGATAAAGAAATCAACCCATCCAA------T------CAAAAGGGTTTCTTTTCTTTTTTT-----------------------------------------------------------------------------------------------------------------------------------------------------------------------------------------------------------------------------------GATTGGATGGGAATGAATGAAGA------------------AAGACTAAATCGTCCTGTATCGAAGCCGATACCTTGGTTATTCCCACAATTTGAGTTATTTTTTAA---------------------------------------------------------TGTATATAAAATGAAACCCGGGTTTATACCAATTCATTCACTTATTTT---------------TCATTTTAATGAAGATGTTAGTAAAAATAAAAATATCA------------CTAAAAATAAAAAAGGGGATCTTATACTATCAAATGAAAAAGAAAACCAATATTTT------GAATTAGAGAATCAAG------------------------------------------------AAGAAAAAAAAATC----------ATAGGTCAAAGA---------------------------------------------------------------------GATCTCGCATCAGATGCCCAAAACCGAGGGAACCCTGAATCTGTTTTC-------------------------------------------------------TCAAACCAACAAAAATATATGGAAGAACTTTATACGAA-ATCAGATATGAAAAT-----------GGGTAGAACGAAAA--------ATCAACCCAAAAGCATTTGGACTTGGGAAATAGACTTAGATGCGTTCATGAAAG--------------GATCTTTTGC-------TTTGCAATTGAAATGGCTG------------------------------------------------------------------------CTGAGCCCTTTGACTATGGAATTA--------------------------------------------------------------------------------------------TTCGATTATGCGC------------------------------------------------------------TGTCCGTACTTGAAT----------------------------------------------------------------------------------------------------------------------------GGGAAAAGGAAAGCAGAATGACAACAAA----------------------------------------------------------------------------------------------------------------------------------------------GTTTGGTTTCGGCTTTATTAAGAAGGAGGAGTTAACTCTGGATCCAATGCTAATCCGGG---------------ATTTTAATCTTTCAAAAATCCTAAAAGAGGGAATATTTATTATCGAACCAGTTCGTATACC---------TGTAAAACATAATGTAGAATTTATTATGTATCAAACCATAAGGATTTCTTTGGTTCATGAGATTAAACAAAAAAAGAATCAAAAAAGATACAGAGAAAATATGGATAAGAATCATTTTGAGGAATCGATTGCAAGACATCAAATGATGACGAAAAATAGAAACAAAAATCATTATGATTTGCTTGTTCCTGAAAATATTTTCTCGTCTAGACGGCGTAGAGAATTGAGAATTCTAAGTTGTTTCAATTCAAGGAATAGTAATTGTGTGGATAAAAATGCAGTATTTTGCAATGGGAACAAGGTAAAAACCTGTGGTCAATTTGTGGATGAAAGCAAA------GATCTTGATAGAGATAAAATTAAATTAATTAAATTAAAATTATTT------CTTTGGCCCAATTATCGATTAGAAGATTTAGCTTGTATGAATCGCTATTGGTTTGAT---------------------------------------------------ACTAATAATGGTAGTCGTTTCAGTATGTTAAGGATACATATGTATCCACGATTG-CAAATTGATTGATGATACAATTGTCT-----------TCCCCTACG--------ATAT----ATATCGGGTGGATAAATAGCTGCGCACATGCCTTGTCTTACATCCTTT-TTTTATACAT-GAATACTAATTCAATGACG------------------------------TATCAATTAGATCATAAAATGAATCAATAAGTAAATTCGGATTGATTCTTGTGTATACCAGATCAAAATACCTCGCAT---------TATTATTACTGATCAG------TAAAATTCATATTCG------TAAAATAAAAATA-----------------------------------------------------------------------------------------------------GTAAATTAATAAAAAAA-------------------------------------------TTAACGCATA------------------------------------------------------GAATAAAT---------ACAAAAAAAGATAGGAGGAAATGCGCCCCCCGCCTACATACTTGAGACCTTCTCCTAAAAAAAAACTTGCAACACCCAATC--CGTTTGGAGTTCCATCA-----ATTACTCGTCTGTCAAAAAA----ATTA------ACTAGTTCGGACAATCCTCTTACACTTCGAATTATGTATACTGTATAAAAAGCATCTATGTAACCACGATGATGGGCCCAATCATATATT------------ACATTTTGAATTTTGTCAAAA---AAAAAACCA----------------------------TTTGGATGCCTTTTGGCAA-----AAAAATTAATTACGAAAAAATTTTG---------TAAGG------ACGAATAAATGGGTTTATATAAAAAAGACGCAATAACTATTCCAGAATAAGCTATACTAACCGAAAGGGTTG--CAT-------------TTATCACAAATTCAGACCAA------TCAAAAATTTTTTTATTATTAAATT------TTT--------------------GCTGTAAAAGATTTACAGGTGGGGTTAACCACTTGGACACGATATCCAAATCTATGCCT-----------TCTTGATT---------------GAAAGGAATTCCTAGGAATCCAATGAACAAAGTA--------AATAAAATCAATACAAGTAGAGGGAATAACATAGTATTACCCGATTCGTGAGGATATAGCGCAATTTTTTTATTGTCCCAATTATTAATAATAAGAAAG-----GATTGCATGGTTTTTTTTCTATTTTCG---------TGTGGATTCTT---------------AGAAAAACTTTCG--------------------TTATTTTTTATT--------------GGTAAT------------------AAAGGAAAAT---------TTTTGT-----TAATAGGTTTCATTC---CGTCTTGACCCCACAAAGATATTGAA----TA--------------GAAAGAACTACT-TTTTTTTCCATTGTAATTTTGAAAATTAACGTTTAAATGACCCTCAAACGTAAGTA---------AATAGATGCGAAACATATAAAA-----TGCAGTTAATCCTGCTGTAGCCCAGGCTATTATTGCGAAAGTTGGTGAATACAACCAAGTATCATTAAGAATTTCATCTTTGGACCAAAAACAAGCAAGAGGTGGAATACCAGAAAGAGAAAGTGTACCTAATAAAAAAGCAGTTTTTGTAATTGGCACGTGTTTTTTTAAACCCCCCATAAAAACCATATTTTGGCTTTTATCTGGAGAATACCCAACAATAGCTTCCATA---GAATGAATAATAGAGCCAGATCCTAAAAACAACAATGCTTTTGAGTAAGCATGAGTAATCAAATGAAATAAAGCAGCTCGATAAGACCCCATACCTAGAGCTAACATCATATACCCCAGTTGAGACATTGTAGA----------ATAAGCTAAACTTCTCTTAATGTCTTTTTGGGCAAGAGCTAA------AGTAGCTCCTAAAATTACTGTTATTATACCTATAAAAGCGATTAGATGTAGTATGGAGGGGATTACTATCAAAAGAGGAAAAAGTCGCGCGACAAGAAAAATCCCCGCAGCTACCATAGTAGCAGCATGGATAAGAGCCGAAATAGGAGTAGGACCTTCCATA---GCATCAGGTAACCATACATGAAGGGGGAA--------TTGGGCAGATTTGGCAACTGCACCGGCAAA-------TAATAAGAAAGTAC---------ATACAGTTCCAACTAAAAAATTAACTTCATTGTT--------------ATAAATCGAGATATTGAATATTTCGAACAAATCTCGAAATTCAAAACTGCCTGTTAGCCAATAAAGACCTAAAATTCCTAATAATAAACCAAAATCCCCTACACGGTT---AGTCACAAACGCTTTTTGACAAGCATTTGCTGCAACCG----------GTCGTGTAAACCAAAAACCTAT----TAATAGATACGAACACATTCCAACTAATTCCCAAAAAAAATAAATTTG------------TATTAAATTCGAACT---AGTAACTAAACCAAGCATAGAAGTATTGAAAAAACTCAAATAAGCAAAGAATCTCAAATATCCTTGATCATGAGACATATAATTGTCACTAT--------------------AAATAAGAACTAGAATCCCAACAGTAGTGATTAACAGTGACATAATAGAAGTAAGTGGATCAACCAG----------GTAACCGAATTCTAAAGAAAAATCATTATTGATGATCCAAGACCATACAGATTGATAGATAGAACTGCTATTTATTT--------------------------GCTGAATAGACAATTTCATTGAAAAAAGCATAACTATACTTAACAACAAAATACTAGGAAAAGCCCACATACGCC-------GAAGATTTTTTGTTGTTTTTGGAAAAACAAGAAGTCCCGCTCCGATTAACAAAGGAACTGTAAGTGGAATAAAAGGTATGATCCATGCATATTGGTATATATGT---------------------TCCAT----AAAAAATAAA------------------------ATTTGA---------TTTTCGATTCACCGGCTCTTACCTCTTTCGAAAG----------AGATCAAT--AAAAAAA--TTACGAGATGCGA-TA------ATAGAA--------------T----------TTTTTC---------CATTAAAAATCG------AAATTATT----AGAATAAGCATTTTATT-------ACT-------ATTCAACTCAAAAAGTTCT------------------------CGTTGGTCAAATGACCA-----------------------------------------------------------------------------------------------------------------------------------------------------------------------------------------------------------------------------------------------------------------------------------------------------------------------------------------------------------------------------------------------------------------------------------------------------------------------------------------------------------------------------------------------------------------------------------------------A-ATAGTTA-----TTAA------TGAAAGT---AAATAC----------TTAGTTATTAATTAA-ATAAACT--------------------------------------------------------------------------------------------------------------------ATAGATAATATCAAAAATTTATACTGTTC--------------------------AT-----------------------TATTATTTT----------------TATAAAT-----CCA-------TAGATAGTAAA----------------------AT----------GATAACAAATTAGACC-AAACAAAAAAGTTCGTAAGTCTGA-----TACTAGTATGAATCA---CAAGATCTACTAAAATTCATAACCTAATTTAAATTTAAA--T--------------------------AGTTCCTATT------------TAAATTA----------GG---------------------------TTATT----------------CGGAA---------TCAGTTATTAGTTCTCTGT---AAAACTCTTTG--------------------------------------------------------------------------------------------------------------------------------------------------------------------------------------------------------------------------------ATTGATTGTCTTCTA-----GTC----------CAATAAAGCATATTT--TTATTTTTCTATGCTAGCCAAGACTT-------TACTTTTGACTTTACATAA---CATAAAA---------------TT-AAAAAAATTGATAAAAGCATATCA----------------AATTAT--------------GTTTATCTTAAACTAAATAA-----CCAGTCTCAT-----------TTCAAT------AATAAAAATAAT-A-----------------AAA-GAAAAAAATGCGATGTATTT-------GTTAAAAAGAGTCAGGTTTT--------------------CCATTCGTTAAGT-----------------------------------AGG-TA------------AGAGCAGCTTACT-TAACTCTTC----------------TAATTTTTTTATTTTAAACCTTAGATGTGTT----------TAGTCT-------------------------------------------------------------------------------------------------------------------------------------------------------------------------------------------------------------------------------------------------------------------------------------------------------------------------------------------------CTCTATAATCTG----------------------ATATGTAATTTGCAGA------TATTTCTAG-----TTTTTTTAGATATTTTCT--------------------AAAA---AA--AAACTTAGAATTAGAGTAAGAAT-AGAAAA--AG-----GGTCTA---------------TAACTT--------------AAA-AAAAAA------------AAGGACAGAAAGATCCCTTTGCTTTGAATAATAGATGTCTTTCACATCCAACTATAATAA--CGAATAACC----TA--TTCATTTTTGAATGGCAGTTCCAAAAAAGC------------------GTACTTCAATTTCCAAAAAGCGTATTCGTAAAAATATTTGGAAAAGAAAAGGACATTCGGCAGCATTAAAAGCTTTTTCATTAGCTAAATCTCT------------TTATACCGGGAATTCGAAAAGTTTTTTT------------------------------------------------------------------------------------------------ATACGA-------AAAATAAGTAATCAAATGT-----------------TAGAATAATCTGAATCGATCTGACT-AAAAAAAA-------ACTTCTACAAAATTTCCTTTAGCATT------------TATATTC-----ATAAAACAT---------------------AAAAAAATCAA-------TCATTTAAAATTTAAA----TAT-----------------AGAA-----------------------------------TTAATGCTTTGTATTCATTAATGC---------------------------------------------------------------------------------------------------------------------------------------------------------------------------------------------------------TTTTT---------TTTGAAAACTAGAAAATTT-----------CACTACC-------CTTCTTTT----------TTTTTTTTTTAGTATATTT------------------------TATTATTTCTGGGATGGGGAGTCT----------------------TACTTTC--CCCATCAACCGGCTGGTT---------------CCAATAGA--------------AAATTAAGG-TGAGT------TTTATATCT-------------------ATTATAG--------------------------------TTTATAAAAATATCGCCATTGAATTGACTCTTTCAATCTCGACGATTAAAGATAAATAGGCTATTATGATTTAAAACAAGCCGCTATGGTGAAATCGGTAGACACGCTGCTCTTAGGAAGCAGTGCTAGAGCATCTCGGTTCGAGTCCGAGTAGCGGCACAACTTTTTAAAAATTCTAAAAAGGAATCTAATA---------------------GTT-------TT-TTTCAAATGTTAGAA-------TAATCTGAA------------------TCGATCTGAC-----TAAA------AA---------------------------------------------------------AAAACTTCTACAAAATTTCCTTTAGCA-----------TTTATATTCATAA--------------------------AACAT-AAAAAAATC---------AATCATTTAAA---------------------ATTTAAATATAGAATTAATGCT-------------------TTGTATT-------------------------------------------------------------------------------------------------------------------------------------------------------------------CATTAATGC----------TTTTTTTTGAAAACTAGAAAA---------TTTCACTACCCTTCT-----------------------------------------------------------------------------------------------------------------------------------------------------------------------------------------------------------------------------------------------------------------------------------------------------------------------------------------------------------------------------------------TTTTTTTTTTTTTAGTATATT------------------------------------------------------------------------------TTATTATTTCTGGGATGGGGAG-------------------TCTTACTTTCCCCATCAACCGGCTGGTTCCAATAGAAAATTAAGGTGAGTTT-------------------------------------------------TATAT-------CTATT----------ATAGTTT------------------------------------------ATAAAAATATCA-------------------CCATTGAATTGA----CTCTTTCAATCTCG----ACGATTAAAGATAAATAGGC-----------TATTATGATTTAAAAC---AAGCCGCTATGGTGAAATCGGTAGACACGCTGCTCTTAGGAAGCAGTGCTAG------AGCATCTCGGTTCGAGTCCGAGTAGCGGCACAACT---TTTTAAAAATTCTAAA------------------AAGGA-----ATC---TAATAGCCCTA----GACTGAAT------AATA---ATCACAATGAGATGA-----ATTCTAAATTTTTAT-TTCA------------------------TGATTTGTAAT----TGAGGGAT--------CTCTTTTATTT-------------------------------------ATATATATATA-------TATATTCTTA------------------------------------------------------TGATACTTTTCACTTTAG------------------AGCACCTTTTCAATCATATTTCCTTTTCGATCGTTTCAATTGTAATTACAATTCATTTAATCACTTTAGTAGGCAACGAAATAGTAGAACTA--GATGATTCATTAGAAA--AGGGTATGATACTT------ACTTTTTCCTGTATAACAGGATT--ATTAGGTA---------TTCGTTGGATTTATTCGGGGCATTTCCCGTTAAGTGATTTATATGAATCATTAATCTTCCTTTCGTGGAGTTTTTA--TATTATTCATATGATT---------CCTTATTTTAAAAAACTTAAAAAATTTG------TAAGT------GCAATAACAGCGCCCGGTGCTATTTTTACCCAAAGCTTTGCTACTT--------CAGGCTT--TTTAGTT-----CAAATGAAGCAATCCACAATATTAGTACCCGCTCT--------------------CCAATCCCAGTGGTTAATGA----------------------TGCATGTAAGTATG-----ATGATATTGGCC-------TATGCAGCCCTTTT------ATGTGGATCGTTATTATCAGTAGCCCTTCTAGTCATTATAT------------------TTCAAAACAATATAAGTC---TTTTTGGTAAAAAAAAACTTTT-------ATTAAAC---------------------------GAGTCTTTGTTCTTTGGGAAGATCCAACACATGAATAAAGAAAACAAA--------------------------------------------------------------ATTTTCCAAAGCATTTATCTCTTTTCTCTTAGAAACTATTATAGGTCACAGTTAATTGAACAGTTTGATCGTTGGAGTTTCCGTGTTATTAGCCTAGGATTTCTCTTTTTAACCATAGGTATTCTTTCAGGAGCCGTATGGGCTAATGAAGCATGGGGATCATATTGGAATTGGGATCCAAAGGAAACGTGGGCATTTATTACTTGGACCATATTCGCTATTTTTTTACATATTAAAA---CAAATAGAAATTTAAAAAGT----------------GAAAATTCTGCAATTGTGGCTTCTATAGGATTTCTTATA-----------------------------------------------------ATTTGGATATGCTA-----TTTTGGGGTCAATTTATTAGGAATAGGATTACACAGTTA-----------TGGTTCATTTCT-----ATTAAAAAGCAC--------------------------------------CTAAAGT---------TAATT-----GCAGAAAGGACC---TAACCTGACGAATACAACTATAAGAACAGGG--------------GATATCCCATATCC----ATATAAAAAT-AAGCAAGTCTCG--------ATCGAG----AACCATTTCAATCAAGTAGTATAGTGATTGAAATGGT-----------------TC-------------------------------TCGCAAACGTCAAATTATCC-----------GATT----------TAAATTCTAAT--------------------------------------------TCGTTTTTTT----------GCGTTACGTAAAAAAGATAGT------------TTCAAATGGAAACTATCTATAAAAAAAAATTA----------------GATA-GAATAGCTTCTACCTTCTCAACTGATAG-------TGAGAGAACGA----------------AATCCGGGTAA---------ATGCCAATACCTATT--------ACTGGTAGAAAGATAGTGAGTGA------AACAAATAACTCTCGCGGAC-----------------------------CTGAATCAAAAAACGAAG---AG--TTTGCACTATTTAATAACTTGTATCCATAGAACATTTGG-------------CGTAACA-------------TAGACAATAAATAAATAGGAGTTAATATCATTCCAATTGCCATGCCA----------AAAGTAATTAGGACTTTTG---ACATTAA---AAAAACTTTTTGACTGGTAATTATTCCAAAAAATACTAT---------TAATTCGGCAAAAAAACCACTCATACCTGGTAACGCAAGAGAAGCCATCGAAAAAGTACTGAAAAGTGTGAATATTTTTGGCATTGAAATGGCTATT---------CCGCCAATTTCGTCAAGATAAACAAG-ACGTATTCTA---TCATAACTCGTTCCTGCTAGGAAAAAAAGAGCAGCACCGATAAATCCATGAGAGAT----------TATTTGTAAAATGGCC-----------------CCGTTGAATCCCGTATCAGTT-ATAGACCCAATTCCTATAATTATGAAACCCATATGAGAAACAGAGGAATATGCTATTCTTTTTTTTA--------AATTACGTTGCCCCGAAGATGCTGAAGCT-----------GCATAGATTATTTGTATTGTGCCTGCTATCATCAACCAAGGAGAAAATATAGAATGAGCGTGAGGCAATAATTCCATATTGA-TCCGAACCAATCCATACGCTCCCATTTTTAATAATATTCCTGCTAAAAGCAT------------------ACAAGTACTATAA--TGTGCTTCTCCATGGGTATCCGGTAACCATGTATGTAGAGGTATAATCGGCGATTTGACAGCAAAAGCAATAAGAAATCCAATATAGAATATTATTTCTAAT-----------------CCTACAGGATATGATTGATTAGCTAACGTT---TCCAAATTTAATGTTGGTTCATTAGAACCATATAACCCGATACCCAAAACTCCCATTAACATAAAAACGGAACCCCCTGCAGTGTA------------CAAAATAAACTT--TGTAGCTGAATATAGGCGTTTTTTTCCTCCCCATACGGATAAAAGTAAATAAACGGGAATTAATTCTAACTCCCACATTAGAAAAAAAAGTAAAAGGTCGCGAGAAGAAAATAATCCTATTTGACCACTATACATTGCTAACATCAAGAAATGGAATAATCGGGAATCCCGAGTAATTGGCCAAGCCGCTAAAGTAGCTAAAGTCGTGATGAATCCGGTCAGTAAAACGGGTCCTATAGAAAGCCCGTCTATT--CCCAATCTCCAGTGGAAATCAAAAAAGTGAATCCAGTTATAATCTTCAGTCAATTGTATTAATGGATCGTCCGGTTGGAAATGATAACAGAACACATAGATTGT--TAGGA-GGAATTCTAATATACATATACACATAGTA-----------TACCA----------------------CCTAATTACCTTATTACCCCTATGGGGTAG-----------------------AAAAAAAATTAATGAACCCGCAAATATAGGCAAAACTACAATTAAGGTTAACCAAGGA----------AAATAATTCGTGGTAAAGACAAAATACACTTGGA-CT----------------------------------------AA-AAAACCCGTACTCG-AATAAGAACAAAATAAG----ATATATA--------------------------------------TATTTC----------------------ATTTCGAGCGCGGG--------------------------TTTTTGTCGGT------------AAACAAAAATCAAAAGGATTC-------------AAGTGGAGTTTTC-------------TGGAACGTATCAATAAGC-----------TAGACCCATACT----GCGAGTTGTTTCATGCC-----------------------------------------ATAAATAAACTCGAACACTCAAAAAATCGGTTGGACAGGCGGAT--TCGCATCTCTTACAACCAACA-------------------CAGT------------------CCTCTGTTCTTGGGGCGGAAGCTATTTGTTTA-----------GCTTTACACCCGTCCCAAGGTATCATTTCTAATACATCTGTG------------------------------------GGGCAGGCTCGGAC--ACATTGCGTACATCCTATACATGTATCATAAACCTTTACTGAATGTGACATTGG-----------------ATCTATACCTTTTTGTTTTT-----GAATCTC----ATAAA-TTTCGATCTAGTATAAC---------------CC-----------CAT--------------------------------------------------------ATTGTATTTACATTAATTAC-------ATATGC----AAAGACCAGACGAATCGATGATTCACCAGAATTTTTTGAATCAACTTATTT---------CTGGGTCGGT-------TTAT------AAAAGAGGTCCAAAATACT--------TGGAGTTCTT------ACATTTTTGA--------------AG---------------ATTCT----------ACATAC----------------CCAGTAA-----------------------TCTA-----------------------------------------------------------ATTTTAATTGATAACTA-CTCTT--------CAAATTTT-------------------------------------------------T-------A--TAATTAAT------------AATA----------------------TACT-------ACTTATTCAACAAAT---------TTGATTGATT-----AATACGAG---------------------------TTGATTTTCTGTTACGATA-----AATTGCCGAAACAATAGCCGGTCCAATA------------GCTGCTTCAGCGGCTGCAATAGCTAT---------AAC------------AAAAATGGAGAAAATGTTTCCTTTTA-----------GTTGACGACTATCAAAA--AAGTCAGAAAATGTTACGAAGTTAAGATTAACCGCATTCAAGATAAGTTC---AAGACACATAAGAGCTCTAACTAAATTT----------CGGCTTGTGATTAATCCATAGATACCGAT---------AGAAAATAAATAGGCACTCAAAACAAGTACATGTTCGAGCATCATTGAGTAACTCC------TTATCAATC--TTGA------------TTTATTTCAATATGAACAA------------------AAAT---------ATCATTCACTCGAATATAAC--AAACAAATACAGAGCAAAG------AAGTATGTTAGT-------------------------------AATAG---------TATA------------C-ATCAATTCAAATAGAATTGAATGAAAATGTATT-----ATA--------CATCAA---TTCAAATAGAATTGAATGAAAATGGATACGATAA----AAGAGAAATAGAATAAAG----------TTTGAT---------TTGGCGTGACGCTTTTTA-------AGATTTTAATCCTATTGGCGGGCCACGGCAATTGCACCTATCAAAGCAACCAAAAGAATTATTGAAATGAGTTCAAATGGGAGAAAAAAATCTGTTGATAAATGAATTCCAATTTGTTGACTATTA--T---TTAT------CAAATCTTGTTCTATAATCTGGTTGAATT--TTGTAGTCCAAATAATTCCGTACCATGACGTATTTAAAATAGTAGTAATTAATAAAAAAAAAATACTGGTACAAAC--TAACAAAGTAATTCCGTCTCCAAGAGTCCAAAGACGAAAATTTTTGTCATATTCT---------------AACCCGGTGATAAACATTACAGCAAATATGATTAAAACATTTATAGCTCC-----------------------------TACGTAA---ATAAGGAGTTGCGCAGAAGCTACAAACTGAGAGTTTGC-----TAGAATATAGAATAAAGATATACAAACAAGAACCAATCCCAACG-------AAAAGGCAGAAAA---------------------------------AATGGGAT----------------------TGGTAAA----------------------------TAATATCACCCCTAGGC-----------------CTCCTAATATAAGACCTGATCCCAGAAAGACTAA-----------------AAGAA----AATCATGTATTGGTCCAGGTAAATCCATTCGATG---------AAA-----------AAAAGATATA----------AAAAATCAGACTCT----------------TTCATGATCTTATTGAACTGACCAGGATAAAA---------TAAGTTTAAATTTATTTAT------TTAAGACACGTTTC------TAGTTG---AATGCGATTCT------------AATGGGTGCGAATTGATGTAGGTACAGCTAGTGTA-------CAAAC-----CATATTCTTTATCTGAAAGGCTAGCTCGAATTTA-----GTTGAAATCATTACATTA-------AAAAA------------GTCCAAGTAAATCGGCAATTTTCATGAGCCAATC--------------------------------GGATCAATA-------GGTGTTATTTTGAATTTAG-T------TATTCAC----------AAA-GAAAAA-----------ACCTGTTAAATTTATATACAACC-------TTAGTT-----------TAGTAATAAAA-AAGGGTTCTTGAATTT-------ATAAAGTTATTTAT---TTT----TTATTGAATTGAGGCCAATTCAAGATAG-TTCGAGTTGTGT----------------AATCGTCAATTGTGGATAT-----------TGGTAAACGACCTAAAGCAATTTGATTATAATTTAATTCATGACGATCATATGTAGAAAGCT------CATATTCTTC--------------AGTCATTGATAAACAATTTGTTGGACAATACTCAACGCAATTACCGCAAAAT---------------ATACAGA--------------------TTCCGAAATCAATAC--------------TGTAATTAAGCAA--ACGTTTCTTTCTAATATTAGTTTCCAATTTCCAATCTACAACAGGTAGATCTATAGG---ACATACACGAACACATACTTCACAAGCAATGCATTTATCGAATTCAAAGT-GGATTCGACCGCGAAAACGTTCT-GATGTGATCA--ATTTTTCATAAGGGTATTGAAT-----------------------------------------------------------------------------------------AGTTACAGGTAAACGATTCGCATGGGATAA----------------------------------------------------------------------------------------------GGTAATCTGA-------------------------AAACCTTGACCAA--TGTACCTAGCCGCCCGTACTGTTTGTTGACCATAA--------------------------TTCAGGAACCCAGTTAGCATAGGGAACATATCCTAAA--------------------------------------------------------------------------TATCGAT---------------------------------AA---------TTTTTTTTCTTTTTCTT---------GTTTGGGAC---AAAT------TATTAATTTAG----------------TTA-CTAGT--------GAATATAAAA------------------TATTCCATTTT-GC----------------TTA--------------------TGTTATAGTGAAAGAAGT--------------------------------------TGGGAAGAAG---------------------------TTGTTAATAATAAATTACCTAAAGAAATAGGT----------AAAAGAAATTTCCAT------------CCAAGATTTAAT---AATTGGTCCATTCTCAGTCTAGGTAAAGTCCATCTTGTTGTGATAGAAATGAACAATAACAAAAAAGTTTTTACTAGTGTAATGAAAATACCAATTGTTGTTTCAAAGACCCCATCCCTTGCATTTATTCCAAATAGGTCAGGAACGGAGATGTA-------CGGAATAGATAA----ATTCCAGCCTCCCAAGTAAAGAATTGTTACAAATAATGAA-------------------GAAACTAGTAGATTTAGATAGGA----AGCAACATAAAATAAACCAAA----TTTAA---------------------TAC-----------CTGAATATTCT------------------GTTTGATAACCTGCTACTAATTCTTCCTC----------------------------------CGCTTCTGG-----------TAAATCAAAAGGCAATCTT----TCACATTCGGCTAGAGAAGAAAT----TATAAAAACGA--------GAAATCCTATAGGTT--------GACGCCACAAATTCCACCCCCACAAAC-CA---------TATTTAGACTG---TGCTTCAACTATATCAACTGTACTTAAACTGTTAGATAATTCTAGTCGGTGATAAGATCACAATTATCATCGCTATTACAGAACCGTACATGAAATTTTCACCTCATACGGCTCCTCGAGGGTCA--CACATAAATCTAAG-GACTGCTTCGATATTC--------TTTA--ATC-----------TTGAAATTTTTATAGGATAGATAGA---------------GTCAAAAGTAATC-----------GAAAGGTCCC-GAATTAGATCAACGGAATTCTGTC-------TGCTATACTA-----------------AAGGGCGTCTGAATTGATCTCATCCTTTACTTTTTTT---------------------------------------------------------------------------------------------------------------------------------------------------------------------------------------------------------------------------------------------------------------------------------------------------------------------------------------------------------------------------------------------------------------------------------------------------------------------------------------------------------------ATA-------------------------------------------------AAAAATGAAG-----------TAAGTAAAGGA-------------------------TTACTTCGTTCCTGATAGTAATTCA--------------TTTTTCGGTGGATA-----------------GCAGCATACTC-----TGGATCGGAATTCTGGGGAGT---------ACTACTCGATCATTTCTACAAATTTAAAGCCCCAA-TTAGTATTTCTTTTA---TTATGTGA------TGTGGAA-------TTTTTTC--------------------CGATAACGT----------------ATAAAATCTCT----AT-TACTAATCCTTTGTGTACCTTGGTGTTCCTAACCATCCACTCAGTCTTGCTCAATCT----TTTCGAC----AATTCGCATCATGTA--------------------------------------TAGTAA----------TAGATATAAACGATAGC--------ACGAACTCCAAAGAATGGA----TCTGTTTAACCCGCTTCAAGCCATGATAACTAAGCAACGAG-TCT----------------------TGGGGTAA-------------ATAGTTT-TTCTTTACTACTTCTA---------------TTTGCT--------TATATTTACT-TTGGCGTAATTCTTGTA---CATA-GGAAATGAGACT-CAATCTT-----TTTAC-----------TGCGAATTTCGA-----------------------------------------------AGCTG--TTTTCTTTCACTCATATAACTATCTG---------GTTT-----AGTTCATCAACCCGAAGGTTG-AGTAAAAAATAAAGTTATCTA------TTTAA-----TGTAT--TTTAGTTCATTCTTAG-AAAACTCTC----GA---------AAAATTTTGTGGAAATTTCA--TGCCTCAACGAATCGCACGTAGAGATATTGATAAAACGGATAGAGTTAATGGTATTTCATAACTAA-------TAGATTGGGCAGCAGCCCGTAGACCACCTAAAAAGGAATATTT-ATTATTTGATCCATATCCTGACATA--------------------------AGAAGTCCA------ATGGGAGCAATACTTGAAAT-----GGCAATCCATA---AAAAAAC----------------------------ACCATTACTGAGA---------TCGACTAGAATAAGTTGATAGCTAAAAGGAATTACTGAATAACTTAGTATAATTGATAT-GACTGCTATGGAGGGTCCCACACT-----------A--AATAAACTCCTAT------------------------------------CA-----------CCTCTTGATGGAAGAAGGTTCTCTTTG------AAAAGTAATTTTGTTC-CATCTGCTAGAGCTTGAAGAATTCC------TAAGGGGCCGGCATATTCAGGTCCAATACGTTGTTGTATCCCTGCGGATATTTCTCTTTCTAACCACACAATAACTAGTACACCTATTGTGATTCCCAAAATAAGAATCAA-------------------AATAGGTACAAGC-----------ATCCATATAGCCCCATA-----------GATTTCTTTTAAGGATTCCAAAATAGAAA---------AAGAATTGATAG---CGTGTACTCCT------------GTTGTATCAAT--------------TATCATTTCAACGATCAA---------TT-------------TCTCCCATAATGATATCTATGC----TACCTAGTATTG--------TCATAATATCAGCCAATTTCATTCCTTTAA------------------------------CTAACTGAGGAAGAATTTGCAAATTGATAAAACCCGGCGG-------------------GCGGATTTTCCATCTCCACG----------------------------------------------GAAAAGCACTCTGATCTCCTATCAAATA------------AATTCCCAATTC---------GCCCTTTGGTGCTTCGATTCTTACATAAAGTTCTTGTCTCGATAACTCAAATGCGGGGGCCGGCTTTTTACTAATGAATCGATATTCAAAATT---ATTCCACTCAGGATCTTTTATTC---TAT------TAAAGCGTCGGATTTCTAAATTCTCATAGGGGCCCCCC------------------------GGAATTCCTTCTAGAGCTTGCTGAATAATTTTGACAGATTCCACCATTTCGCCAATTCGGATTAAATAACGAGCTAAC-GAATCGCCCTCCTTCTGCCATTGAACTTCCCAATTAAATTCTTCATAACATTCATAATTATCA------------------------------------------ACTTTACGAAGATCCCATTGTATTCCG-------------GACGCCCGTAACATTGGTCCTGACAAC----CCCCAATTTATGGCCTCTTCTCCACCAATAATACCCACCCCTTCAACGCGTTCTAAAAAAATAGGATTTCGCGTAATAAGCTTTTGATATTCA-------GCAATTGCTGTTAAAAAATACTCACAGAAATCTAAACATTTA---TCTACCCAGCCATAAGGTAAATCGGCAGCGACTCCTCCGATACGAAAATAATTATGCATCATTCTCATGC-CAGTGGCAGCTTCGAATAGATCATATATCAATTCTCTTT-CTCTAAAAATGTAG--------AAGAAGGGGGTCTGTGCACCAATATCCGCCATAAAA----GGCCCAAGCCATAATAAATGAGAAGCTATACG---------------------------------------GCTCAATTCCAACATA-ATAACTCGGATATAACTAGCCCTTTGGGGTACTTGAATATTTCCTAATTGTTCTGGTGCATTTATAGTTATTGCTTCTGTAAACATAGTAGCTAAATAATCCCAACGTGTTACATAAGGCAAATATTG
[truncated: 11,803,126 more chars]
