# Supplementary material for: The complete annotated plastome sequences of six genera in the tropical woody Polygonaceae
Source: BMC Plant Biol. 2024 May 17;24:417. doi: 10.1186/s12870-024-05144-y (PMC11100190; doi:10.1186/s12870-024-05144-y)
Supplement: Supplementary file 4 — Supplementary Material 4. [file 12870_2024_5144_MOESM4_ESM.docx]

Supplement4: This is the phylogenetic tree file resulting from the IQ-TREE analysis. The file is in NEWICK format.

("Rheum palmatum":0.0033400100,"Rheum franzenbachii":0.0017450642,("Rheum racemiferum":0.0059170322,(("Rheum pumilum":0.0070775650,"Rheum acuminatum":0.0059882876)99:0.0006699930,(("Oxyria digyna":0.0000188915,"Oxyria sinensis":0.0000500744)100:0.0276045708,(((("Rumex crispus":0.0008786241,"Rumex nepalensis":0.0012618555)100:0.0009755088,"Rumex japonicus":0.0020179472)100:0.0130051925,("Rumex acetosa":0.0273416881,"Rumex hypogaeus":0.0134696842)100:0.0027795674)100:0.0170038617,(((((("Muehlenbeckia complexa":0.0011514557,"Muehlenbeckia australis":0.0579027608)100:0.0025602207,"Muehlenbeckia platyclada":0.0054163033)100:0.0033430254,"Fallopia aubertii":0.0151842674)100:0.0045150576,((("Polygonum cuspidatum":0.0005179780,"Reynoutria japonica":0.0004066810)100:0.0013142564,"Fallopia sachalinensis":0.0012693617)100:0.0088762983,"Fallopia multiflora":0.0087456443)100:0.0011585719)100:0.0033075581,(("Atraphaxis bracteata":0.0017062464,"Atraphaxis irtyschensis":0.0014165187)100:0.0244617284,"Polygonum aviculare":0.0310948496)100:0.0096503073)100:0.0230508144,((((("Calligonum leucocladum":0.0006484212,"Calligonum gobicum":0.0002026720)100:0.0002455098,"Calligonum aphyllum":0.0003120793)100:0.0002770925,"Calligonum arborescens":0.0006994765)100:0.0010062686,"Calligonum jeminaicum":0.0019059979)100:0.0253811771,("Pteroxygonum denticulatum":0.0315294156,(((("Fagopyrum dibotrys":0.0011185587,"Fagopyrum tataricum":0.0019627546)100:0.0071118943,"Fagopyrum esculentum subsp. ancestrale":0.0126350029)100:0.0322707017,("Fagopyrum leptopodum":0.0010926773,"Fagopyrum luojishanense":0.0008228128)100:0.0322976200)100:0.0229716819,((((("Persicaria orientalis":0.0239453484,"Persicaria neofiliformis":0.0086605573)100:0.0013289141,"Persicaria perfoliata":0.0205740880)100:0.0131999396,("Polygonum chinense var. procumbens":0.0002779762,"Persicaria chinensis":0.0003421248)100:0.0317770936)100:0.0194420219,(("Bistorta vivipara":0.0001242161,"Bistorta macrophylla":0.0001119131)100:0.0423069725,("Koenigia islandica":0.0848720526,"Koenigia forrestii":0.0008957492)100:0.0728163017)100:0.0129913359)100:0.0098344115,((((((("Coccoloba uvifera":0.0005341644,"Coccoloba rugosa":0.0007614922)100:0.0032991081,"Neomillspaughia emarginata":0.0632702465)100:0.0047338963,"Podopterus mexicanus":0.0089732963)100:0.0023872862,((("Triplaris americana":0.0002019730,"Triplaris cumingiana":0.0000714846)100:0.0052748615,"Ruprechtia coriacea":0.0045910191)100:0.0082734019,"Gymnopodium floribundum":0.0109885843)100:0.0032430714)100:0.0025402440,"Antigonon leptopus":0.0163632662)100:0.0042575762,"Afrobrunnichia erecta":0.0195046797)100:0.0028918253,("Symmeria paniculata":0.0214449073,("Ruprechtia albida":0.1634258379,(("Limonium aureum":0.0033132763,("Limonium sinense":0.0006283432,"Limonium tenellum":0.0185333774)82:0.0026632878)100:0.1012563730,("Ceratostigma willmottianum":0.1845793729,"Plumbago auriculata":0.0356247117)100:0.0349308742)100:0.0701951646)100:0.0351178929)100:0.0067159345)100:0.0120313997)100:0.0043053284)100:0.0032615926)100:0.0065158158)100:0.0024533133)100:0.0194959919)92:0.0012425979)100:0.0130322253)94:0.0002414056)100:0.0031986626);
