# Supplementary material for: The complete annotated plastome sequences of six genera in the tropical woody Polygonaceae
Source: BMC Plant Biol. 2024 May 17;24:417. doi: 10.1186/s12870-024-05144-y (PMC11100190; doi:10.1186/s12870-024-05144-y)
Supplement: Supplementary file 5 — Supplementary Material 5. [file 12870_2024_5144_MOESM5_ESM.docx]

Supplement5: This file contains the complete chloroplast genome sequences assembled in this study. The sequences are in FASTA format.

>CoccolobaRugosa
CGGGCGAACGACGGGAATTGAACCCGCGCATGGTGGATTCACAATCCACTGCCTTGATCCACTTGGCTACATCCGCCCCTATGCTACGCTACTTACTTGAAAATGATTCAAATTGTACTCACTATTCATCATTCTTTTTTTTTGTAACTTTTTATTTATAATTATATTTTATTATATTTATTTATTTTTATTTATTATATTTATTTATTATATTTATTTTTATTTATTATTTTATTTATTTATTATTTTATTTATAATTATATTTATTTATTATATAATAATAAATAAATATTTTATTTTTTTTAAACAAAAATTTTCAATCTTTCTCTCTTGAATGTAAAATAAAAAAATTTCCCCCAAAACAACAAAGAGGGCATAGAAGATAGAAATTACAAAAGAAAATGCGTCCATAAATATACAGAAAGTACAAGATCTAATCATCACTTAAAAAAACATTTTTAAGTAAAAAATAAAATAAGATAAGGAAAACTTATGTAAGTAAAAAATAAAATTAAAGGAGCAATACCAACCCCCTCGATAGAACAAGAAATTGGTTATTGCTCCTTTACTTTCAAAAACTCGACTACACTAAGACCAAAATCTTATCCATTGATAGATGGAGCTTGGATAGCAGCTAGGTCTAGAGGGAAGTTATGAGCATTACGTTCATGCATAACTTCCATACCAAGGTTAGCGCGGTTAATAATATCAGCCCAAGTATTAATTACACGACCTTGACTATCAACTACAGATTGGTTGAAATTGAAGCCGTTTAGGTTAAATGCCATAGTACTAATACCTAAAGCAGTAAACCAGATACCTATTACAGGCCAAGCAGCTAAGAAGAAATGTAAAGAACGAGAATTGTTGAAACTAGCATATTGGAAGATTAATCGGCCAAAATAACCATGAGCAGCTACGATATTATAAGTTTCTTCCTCTTGACCAAATCTGTAACCTTCATTAGCAGATTCATTTTCTGTGGTTTCCCTGATCAAACTAGAGGTTACCAAAGAACCATGCATAGCACTGAATAGGGAGCCGCCGAATACACCAGCTACGCCTAACATGTGAAATGGGTGCATAAGGATGTTGTGCTCAGCCTGGAATACAATCATGAAGTTGAAAGTACCAGAGATTCCTAGAGGCATACCATCAGAAAAGCTTCCTTGACCAATTGGGTAGATCAAGAAAACAGCAGCAGCAGCCGCAACAGGAGCTGAATACGCAACAGCAATCCAAGGGCGCATACCCAGACGGAAACTAAGTTCCCACTCACGACCCATGTAACAAGCTACACCAAGTAAGAAGTGTAGAACAATTAGCTCATAAGGACCACCATTGTATAACCATTCATCAACAGATGCAGCTTCCCATATCGGATAAAAGTGCAAACCTATAGCTGCAGAAGTAGGAATAATGGCACCAGAGATAATATTGTTTCCGTAAAGAAGAGATCCAGAAACAGGTTCACGAATACCATCAATATCTACTGGAGGAGCAGCAATGAAGGCTATAATAAATACAGAAGTTGCGGTCAATAAGGTAGGGATCATCAAAACGCCAAACCATCCAATGTAAAGACGGTTTTCAGTACTGGTTATCCAGTTACAAAAACGACCCCATAGGCTTTCGCTTTCGCGTCTCTCTAAAATTGCAGTCATGGTAAAAAATCTTGGTCTATTTCATTTAATCATCAGGGACTCCCAAGCGCACAAATTCTCTATAATCTATAATAGAAATAGATAATTGAAGGCTTGTTATTCAACAGTATAACATGACTTATATGCCCGTGTCAACCAATATGAATCTAGGTCCATCAGAATTTTTTGTGAATAAATAAAAAAATAGAAAAGTTTTATACACATATGACTTCGATATGATAATTTATATGGGTTGCCCGGGGCTCGAACCCGGAACTAGTCGGATGGAGTAGATATTTTCCTTATTAAAAAGAATAAGAGAAAAATCCCTCCCCAAGCCGTGCTTGCATTTTTCATTGCACACAGCTTTCCCTATGTACACATCTAAAACTCAGTTACTTTCCTAGATGGAAGTTGAATACTCAGTTGATTCAAACCCTACTGTATGAACATTTCAGAATTGAAATGGATGAATTTTTTTATTTATCATATTTTTTTACTAGAATTTCCATTTCATTTAGTTAGATAGTTTCATTATCAATATAAATCATTATGACTGGCCAAATCATGAATACAAAAAATATCCAAATACCAAATGCGTCCTCTATATAACCTTCGTGAAATATAAGAATCTTTTGATAAGATCAAAGAAAGAACTTTTTCTTCCTTGAAAAAAAATTCTTCCAAAAATTCTGAACCTAACCTTTTCAAAAAAGCGCGTACGGTGCTTTTATGTTTACGAGCCAACGTTCTAGCACAAGAAAGTCGAAGTATATACTTTATCCGATACAAACTCCCTTTTCTTGAAGATCCGCTGTAATAATGAGAAAGATTTCTACACATACGCCCAAATCGGCGAATAATATCAGAATCTGATAAATCAGCCCAGGCTGGCTTACTAATGGGATGTCCTAATACGTTACAAAATTTTGCTTTAGCCAATGATCCAATCAGAGGCATAATTGGAACTAGGGTCTCAAACTTCTTAATACCATTATCTATTAAGAATGAATTTTCTAACATTTGACTCCGTACCACGGAAGGATTTAGTCGCACACTTAAAAGAAAACCCATAAAGTCAATGGGCTGATTTGATGATTGATTGATATAGATTCTTCTTGGTTGCAACCATAGGGAAAAATTACATTGACAGAAATTGACAAGGTAATATTTCAATTTAGTCATCAGAAAAAACGTCCCTTTTGAAGCCAGAATCGATTTTCCTTGATACCTAACATAATGCAGAAAAGGATCCTTGAAGAACCATAGGATAACCTGAAAATGCTTAGTAAATACTTTTACAAGATGTTCTAGCTTTACGTAGAAATAGACTCGTGCAAGAAAGGCTCCGGAGGATGTTGATCGTAAATGAGAGGATTGGTTGCGGAGAAAAACAAAGATGGATTCGCATTCACACACATGGGAATTATATAGGAACATTAATAATCTTTGATTCTTTTTTTTTGAAAAATTTGAAACGGATTTCTTTAGAGTAATAACATTATTACAATACTCATAAAGAAAGAATCGTAATAAATGCAAACAGGAGGTATCTTTTACCCAGTAACGAATAGTTTGAACCAAGATTTCCAGATGGACAGGGTGAGGTATCAATATATCTAACACATAATTTAAACGTGAAAATTTGTCCTCTAAAAAAGGAAATATTGAATGAATTGACCGTAAATTTTGAGATTTTTTTAGTTCTTTTCCTTCTAGGGAAGATATTAATCGCAGAGAAAATGGAATTTCCGCAATAGCTGCAAATCCCTCTGAGATCCGTTGAGAATACAAATTTTTCTTGTGCCCAAGAAATTCATTTTTGTTAGAATCATTAACAGAAATAATCAAATGATTCTGTTGATACATTCGAATAACTAAACGTTTTACAACTAGTAAACTGTATTTTGTGTCATAACCCCTTTTTGTATTTGAAAAAAAAATGGACCTATTTAAACCTAAATCCTGATCATGTACAAGTGCATAAATATATTCCTGAAAGATAAGTGGATATAAAAAATCGTCTTGCCAAGATCTATCTAGTTCTAAATATCCTTGGAATTCCTCCATTTAAAATGCGACCAGAAATGAAAAGTAGAGGGTTTCTTGGGTTATAAAATGATACATAGTGCGATACAGTCAAAACAAGGTATTCTAGTACAAAATAATAGATACCTCGGAGACAGGTAAACTCATCAACGGACTCTCTATCTTTTTTCCATCTAATTGGTTTCTTTCTTTATAGTTGATAATAAGATGGTTAGAAATCCTTTATTTTTTCAACCCAATCGCTCTTTTGATTTTGGAAAAAAAGTATCCTTATCAATATACTGTTTCTTCTACACATTCATCTCCTTTTTTTCTAATGGAAAATGGCTAATAGTTAGGATTCACTAAAAAATAGGTAATCCACTCCTGGGAAAAGCCTTTCCCACATCAGTCACTAATATATTTTTAACGTTTAATTAGGGCGGGTAATCGTTCCAATTAAGAACATAAGCTCGTTGCTTTTTCTTTCCCTACAATTAGAGCCATAGGGCTCGATCCATGTATTCAATCGACCCAACTTTGAATTCATTTCGTTTCGTTCTAAGAATTCAACCAAAGTTTTTGTACCGATCTAATAAGAACGAAATAGTTTCATAATTCTCCATTGATACGACATGCTCTTTTTTCCATTCATTCCTTTCAGGATCAGTCGTGGTCTTACAAACACTACCGATGGTGTGGACGAATCCCTTGCTTCATCCAAATGTGTAAAAGAACCTAGCCGCACTTAAAAGCCGAGTACTCTACCGTTGAGTTAGCAACCCGAAGAGGGTATGTAGATACAATCGAGATCAAAATAAAGAAATTAGACAAGACAATCAAAGCATTGAATTAGCAAAAAATCTAAAAAAAATAAAGTTTTCTAATAAAAAAACAGATCAAATAAATGACAATACAAGAAATTTTCAAATAAAAAAGAATTCTAGACCACTTCTTAGATATTCTTATCCCTTAAATATTCTTATCCATTATATATAGAATATATATTTATATTTTTATATATTTTTATATATTATATATATATTTATATATATATATATATTTTTTTATTTCTCTATCTTTCTACCTCTATATTTTTATTCTTTTTAATTTTATTATCCATTTCCTTTTTTATAAAAAATTAGGTTTTGTATCACAGCAAATTCAACGAATCTTTGAATAAAGTAAAAAACAAAACCTATGTTTTGTATGTAGGACAAAAAAATAGAGAAAGAAATGGATCCATTTACACTTGAATTATATTTGCTCACTACACTCTTGTCAATATGTATGTTAAAAAAAAGAATAAATAGATTTATATACAATATTCTATATAAATATATGGTAGAAATATATATATGATAGAAATCTATATATAAATAAAAGGAAAAATAGAAAAAAGGAAAAATAGAAATGATATCAATTTCATTGAAAATTGAAGATAATAAAATAATCAATTGAACAAAAAAATAGGATATCAAATAACAAATAAAATAATAAAACAAACAAATAAAAATAAAACAATACAATAAATAAAAAATAAATAAATAAAGAACTTGTGTTGGATTGGCACTATCTAAATAAGGTACATGATTAGAAAGGAATGTAGATAGAAAAAAAAAGAAGTAGAAAAAATATCAATAACTATACGTCAAATCAAATAAGGAAGCTCCATTCTTTTTCTTTTTTTAGTATAATTCCAAGGAAAACCATCCAATTGAAAGGAATGTCAGAATTGTCTATTGCTAGATCCAATTACTACCGTTAGTGACTTGATCAATATAACTTTCTTCGTTTGTTCACTTGATCTTTCTTCTAGACATCTTATATCAATAAAATAAATTTTGATCATTCATTAGAGGACACAGTCTCCTATGGAAACAATACAAAATAGGTCTGAATAGAGCAAAAGATGGTGGGAATAATAAAGAAAGGATTCTATCAAACTATATACGAATCACTCAAGTCTCTTTCTTGTTGTATTTAATTAAGTGAATTTCGTTTCATTAGGGCGAAGTTCTTTAAAAACCTCTGCCTTCTTTAAAATATCATAAACAGTTCCAGTAGGTTGAGCGCCCCTTTCAAGAAAATATAGAATAGCAGGAACATTTAAATAAGTTTGATTCTTTATCGGATCATAAAAACCAACTTTCCGAAGATCTCTTCCTTCTCTTCGGGACCGAACATCAATTGCAACAATTCGATAGACGGCTCATTGGGATAGATTATATGAACAATACCCCCCCCCTAGAAACGTATAAGAAGTTTTCTCCTCGTACGGCTCAAGAAAAATGATTTTTTATTTCTTTTTTTATTTTTGAAGTTATGTATATATATAATTAATGGCAAATAGATCCATAAAATCATCAAATTAATTAGACTAAGAATTAAGTCCCCTTTTGCTCTTATTCTTCTTTCCGGAAAAAATCATTTGCACTCATAACTCAAGTTGAATAACTCTCAAATAACCAAAAAGAGAATCCTTTGGCATTTCTTTTATTGAGTGGTCTCTAACCCCCTTTTTGTCTGGCTTATTTAACCTCTATTGGATATTGGAATTCTTCATTATAATCCAGTTGTTGATACAATTGAGAATGAAAAGGGCTTTTCCTTGTTTCGGAATCTCTTTGCTTTGAATCATTAGGTTTAGACATTACTTCGTTGATCTTTAATCCTTTCAAAATGGCAGCAACATACCCTTTTTGTGATTGTTTATCAAAGAAAAGAATCATACAAACGCTTGATTCTCTCACGATATACTTTTTATCGAAAAGGGTTTATAAATTCCAACAAATTTTCCTTGTTGATTGGAAACTTGGTAGGATTGGATCCTTTCGATTTCTCTGTCAAAAATAGACTTACGAAGTTGTTCTAATTTATTGATTCACACTAACCCTAGATTCTTGCTCTTAAGAAATGAATCAATATTTTCTACTCGAGCTCCATCATGTACTATTTTATTTTAAATTAACTACAACCCAATAAAAAGTGTGGGTTCTAGTCTAACAGAACAGGGGATGTCGAGCCAAGAGCACCTTTTTATATAGAAAATGATGGATATAAAAATCCACACCAGATCATGTCCTTCAAGTCGCACGTTGCTTTCTACCACATCGTTTCAAACGAAGTTTTACCATAACATTCCTCAAATTTTGAACCGGTATGCAATTGATTCAATTATGGAATCATGAATAGTCATTGGTTTAGTCGCTACATAGAAATCTATACCTATACTTTATCCTATAGACTTTATATTGACTTTATTCTATATTAAATAAATATTCTATATAAATATTCTATTTATATATTAAATATACATATTATTTACATTAAATTAATTCAATTTAAATTATATTAGATTATATTAGATTATTTATATAATAATATATATATAATAATATATATAATAAATAAATATAATAATTATAATTAATTAATAATTATAATTAATTATTTATATTAAAATTAAATAATATACGGATAGAATTATATTAATTAAATTCTATCTAATTGAATATTAATAGATTTTTCTATATCTATATTTTACTATATTTTAGTATCTTATATTTCTGTTTTCTAATCTTATCTTCTATTATACTATTTTCTTATCTTATATAATACTATATATATTTATAATAATCTTATATAAGATAAATAGAATAAATATAAGTTATATATAAATATAAGATATATAATATAAGATAAGTAAGTGAATAATTGAAAAAAAATTCCAATAATGATGTTGAATTAGGAATTTTTTTGACATTTACAATATTTACAATAAAAGCCAAAAAGAAATCCATTTTTTTCTCGAACTCAGCCATTAGCCTTTTATGATTTAAATAAAATAAAACCGATTAGGTATATCGTCGAAATGATAACTTGGAAAAACAAATCTGATTTTTTTTCACAAAAATCGCAAACCCTTGTTACTGAGATCAAAGGTTATATAGATAAGATAGAAATATTTCCTCATTTTATATGTTACGTATTTCTGGGTTCAAGAAATTTTCCGTTAAGGTGAATAAAATGTCTGAATCACCTTTCCTTTCAACCATTACATAGATTTATACTTATTCATTCGTTATAAAATAAAGAAAAAAATACGAAAGACCTAAAAATTAGATTCGAAATGAGATTCGGGTAAATATGAAAATTGACACCTTTTATTTTATTGTTGATTAACTCCTATCTACTCCCCCAATTCCATAGGTATCGGGAGTCATAACCCCCCAAAGCACCTTTTTTTATTTACTTTCCAAAAGTATAAACTGTCTAGGTACAAAACGAAACAAAACAGACCTAGATTAAATACTTGTTCTTCTTTGTTATTTTACCTCAACATAGTTACATAGGAGCTTTAATCCTATTGATTGAATTCAATAAGTACTAAAATAGACTCTTGTTTCGAATACAGACATATAAAAAATAAGGAGAATTAATAATTGAACTGTCTCATTTAAGGGATAGGGAATATAGAATTGCTTTCGCATTTTGATTATGAATGGGAATGCATCCTTGAGAACTCTATCTTGAGAATTCTATTAATATTAACTATTAATAAATTGAATATAGACACACGACATAAGTAACTAATTTCCTTCCCTATGAAGAGGTATATGTCCGTCTGAACCTTTTTTTATTGAAATTATTAGAATCAATCTATTATCCTATCTTGCCTGTATTAGATCACAACTAGATTCAGTTATATCTGTGTGTGCTTTGAACTCAATTCTGTTTGTGAAAAAGATAGAATTCAGAAACTAATCTTTATTTAAAAAAAGCGAAAAAAGAATCAAATTCTATCTATATAATATTAGACTACACTTTATTTTACAAACAGCCCTTTGGTCTGGTCAAAATTTTTTTATGATAGAATCCAGATGCTCTGGGACGGAAGGATTCGAACCTCCGAATAGCGGGACCAAAACCCGTTGCCTTACCACTTGGCCACGCCCCACTTAGATTTCTAATCTACACTAATATTGTTATTGATTGTTCGTCAATTCCAGTCCAAATATCTATAGAATCCAGTCGATTGTTATGAGGATTTTCACACGTGTAGATATAGAATTAAACTGAATTTCTTGATCATTACATATAATTCAATTAAGATAATGTATGAAAGTATGATTTATTCTATTCTCTTTTGATTTGAGAATGGAAGGGTTTTTGATTGAGTAAGTTCAAAATAAAAAAAAGAAAGGATTTTTGATCTACTTTGCTTTCTTCATTTTTCGCTTATCTTATATCAATAACTCAATCAAAATGCAATAATCTTCAAGAAAAAAATGTCTGCTATGCTTAATATCTTTAGTTTGATCTGTATTTGTCTTAATTCTGCCCTTTCTTCGAGTAGTTTTTTATTCGCCAAATTGCCCGAGGCCTACGCATTTTTGAGTCCAATCGTCGATTTTATGCCAGTCATACCTCTACTCTTTTTTCTATTAGCCTTTGTTTGGCAAGCTGCTGTAAGTTTTCGATGAGATTTAAAATCTTGTCCTAGAAAAATGAATGATTTATTCGATAAAAAAAAATTCTAATACGACTATACTAATAAATGAAAAGATCAGATACGTCTTATAGTATGAACTCTCGATTCAAATAGAAAAAGTCTTGGATAGCCTCGAAAAATTGGAATCACTTCCTTTCCCGGTCTAACAACAATTTCCGTGAAAGACCCTATGAGGTCTTCCACAAAAATTGTGGGTAGGAAAGCGACTTTTGATAAAAGGGAGGCTCCTAACACTTAACAAATGAATTCATTTTATGAAAATTTTTTTCGATTTCCAGAAACTACTTAAATTCTCGGTGTCAAAATAGGATATATGGGAGAATCTATTCTCTTTTTTGCACAAAAAGATCTTGGAGATTGTGTAATGCTTACTCTCAAACTCTTCGTTTACACAGTAGTGATATTCTTTGTTTCTCTCTTCATTTTCGGATTTCTATCTAATGACCCAGGACGTAATCCTGGACGCGAAGAATAAAAGAGGAATTTCCTTACTTTATTTTTTTATGAATAAATCAAAAGAATTCAATAAATTCGAAAGAGAAATAAAATAAAAAAAAATAGTAAATCAGCAACAGAAACGGAAAGAGAGGGATTCGAACCCTCGGTACGAATGACTCGTACAACGGATTAGCAATCCGACGCTTTCGTCCACTCAGCCATCTCTCCCTATTGAAAAAAAAGTAATTACAAAAATGAATTACTAATTACTATAGTTAGATTACACATATCGTGCCGATTGAAAAATCTTATTATTTTATTATTATTATTTTATATTTTTTCTAGGCTTTCAATTCGAAATTTATAAGATTCTAGAATCTTATAAATTTTAGAGAATAATTTATAGAATAATTAAAACGAAATATAAGTAAAATTATTTAAATTATTTTAATTAAATTATTTATTTATTATTTATTTAAAATAGAATTCTAAAGTCTAAAGAATTAGAAAATAATAATTATAAATAATTCTATACTATTATAACTATTCTATTTATTTAGAACTATTCTCTTTTTATATTATTGTATTTTATATTATTATAATATTCTATAATTATTAATAGATATTATTAATTAATAATATATATAATATATATAAATAAATTTATATATTTATATATAAATGATAAATAAATTTATATATAAATGATAAATATTAAAATAAATAAAATATTTAAATATAATAATAATTAAATAAATAATTATTTATATCTTTTAAATAATTAAGATAATTAAATAAAATAAATAATTAAGTAATTAAGTAAATAATAAATATATTATATAATAAATATATAACTATTATAAATAGATACAAAATATACTACAAGGTTTATCCTAAATTTCAACTAAGGTAGTAAAGATTCAATAAGTAAAGATTCAATAACAAGATAAATCAATATAAATAAAACCAGAAATACTTCTCCCGAAAGGCCTTTTATTCCCATGGCCTGGCCTGGTCAATACCTCGCCGGGCCTTTTTTTAGTTCAACGGATCATAGATATAAAATTTATTGCATTTTATAAGTATATAAATATAAACAAAAATGCTTGTTATTTATTATTTTTATTATTTATTGAATTGAAGCAAGAACAAAAAAAGGAATGTTTCTAGTCTTTCGATATAGAATCAAAATGCCATTATCTTTTCTTTAAAAAAAAAAAAGAAAACTATAGCTTCTTGCACAATTCGTCTGTTATGACTTTAGCAATTTTGTTGAAACGTATCCGTCAAAACTCTCCATCAAAAAATAGAACTTCGTGCTTAGTTATTTAAATCTCTTTTCTGAATCTCCTCCTACGAAAAATCTCAATACTCTCATTTTTCATGATTATTTTATAATCCTATCTTGATTACGTTAAATTTCGTTGTTCGACAAAAGTTCCATTTCGATACAATAATCGCATTGTAGCGGGTATAGTTTAGTGGTAAAAGTGTGATTCGTTCTTTAAGAGTTAAGGGATCCTTCGATTTGATTCCTAATCCGATAAAAAACTCTATTTCTTAAAAGGAATTAATCCTTTACCTCTCAATGACAAATTTGAGGATAATTTGAAATTCTCGTAATTTATATCCAAGGATCAATTATTAATTGAATAATTTCAACTTTGGATTATGAAATTACGAAACATAATTGTTTTTGAATTGGATCAATACTTCCAATTGAATGAGTATGAGTAAAGAATCCATGGATGAAGATAGAAAGATGAATTTCTAATCGTAACTAAATCTTCAATTTTATATTTGTAGAGAGGACATTGAAGCAAAATAAATGGCTAAAAAACGATGACTTTAGTTTACTAGAGGCATCGATCGACATATTCTTTCTTTTAGCTCGGTAGAAACAAAATGTTTTTCCTCAAGATTCTCTAAAATAGAAATAGAGAACGAAGTAACTAGAAAGATTTTTAGAATACCCATCTTCTAGAGGGATCATCTAGAAAGCGAGTACTTTTGAATGCCTTCAGGCAAAAGCTGACATAGATGTTATGGGTTAATTTTTTTTTGTTCCCATTCTAGATCTCGATCTGGCAATTTCTCCATCTTCCATAAAGGAGCCGAATGAAACCAAAGTTTCATGTTCGGTTTTGAATTAGAGACGTTAAAAATGAAAAATAGACGTCGACTATAACCCCTAGCCTTCCAAGCTAACGATGCGGGTTCGATTCCCGCTACCCGCTCTATATTCTAATTTTTAATGCATTAAAAAATGCATTAATTCCTGAAATTCTTTCATCTCACATCACATACAATCTGATTCGAACAAGAAATAGGAAAGTAAAAAAGGAAAAATCGTAATGAGAAGCGTCCATTGTCTAATGGATAGGACAGAGGTCTTCTAAACCTTTGGTATAGGTTCAAATCCTATTGGACGCAATTTATTTCCATATGTTTTTTTCGATGTCTATAGGATTTTTTTTGATATTCTTTTATATTCTAAAGATAAAGAATTTGAATCAGAAATGTTTTTTAGATTCTTTTTTTTATTATTATTAATTTATTATTGAATTATTAATAAAATTAATAAATAAAAATAAATTATAAATTAAATATTAAATAATAATATTATTCTAATTTAATATTATTCTAATTTAATATTATTCTAATTACTAATTTACTAATTACTAATTTAATATTATTCTAATTACTAATTATATAAATATACTAATTATATAAATATAATAAAAAAATTTAATATAATAAATATATATAAGTATTTCTTTGATTATTGATTATTAATATGATTGATTATTAATATGAAATTAAGTTATTAAATTAAGCTACGAAGGCTCAATTTCTTTATGCTTGTTCCTGAAGTAGAAAACGTTCCATCTGTTCTTGAATACCTTCTTTTAAAAGGGCTTCCGCTTCCTCAGTGAATGTCTTGGTAGAAGATATAATTTCTTGGAACTGTGGTTTATTCGTTTTGAAGTACTTACGTAAGTCATCAAGAAATGGACTTACCTGTCCAATTTCTAATGAATCAAGATAACCATTCGTTCCAGTATAAATAGTCATTATCTGTTCTTCCACCGCCAGGGGGGAAGCTTGGGATTGTTTGAGCAATTCGCGTAATCGTTGACCTCTTGCCAATTGATTCTGAGTAACTTTATCGAGATCAGAAGCAAATTGTGCAAAGGCTTCTAATTCTGTGAATTGCGCCAGTTCCAATTTTAATTTGCCGGCCACTTGTTTCATGGCTTTAATTTGAGCTGCAGATCCCACTCTGGAAACAGAAATACCCACATTAATAGCAGGTCTGATTCCAGCATTGAATAGATCGGCGGATAAGAATATTTGTCCATCTGTAATAGAAATTACATTAGTAGGAATATAAGCCGAAACATCTCCCGATTGGGTCTCAACTATCGGTAAAGCGGTCATACTTCCTTCGCCTAAACGCGAACTTGATTTAGCGGCTCTTTCCAAAAGGCGTGAATGCAAATAAAAAACATCTCCTGGATAAGCTTCACGACCCGGTGGTCTTCGTAATAGAAGAGACATTTGTCGATAAGCCTGTGCTTGCTTGGAAAGATCATCATAAATGATTAAAGTGTGTCGTTCACGGTACATAAAATATTCAGCCAGTGCCGCTCCTGTATAAGGGGCGAGATATTGTAATGCAGCGGGAGAATCCGCAGTTTCGGCTACCACAATAGTATATTCCATCGCTCCCCTTTCTTGGAAAGTAGTGACTACCTGCGCCACCGAAGATGCTTTTTGACCAATAGCTACATAAACACATATTACATTTTGTCCTTTTTGATTGAGAATAGTATCTGTGGCTACTGCTGTTTTACCAGTCTGTCTGTCCCCAATAATTAATTCTCGCTGACCGCGGCCTATAGGGATCATCGAATCAATAGCAATAAGCCCCGTTTGAAGAGGTTCATATACGGAACGTCTCGAAATAATACCAGGAGCGGGAGATTCAATTAAGCGAAATTCCGAAGCTGAAATTTCACCTCTACCATCAATAGGTTTAGCCAGGGCATTTATAACACGACCCAAATAAGCCTCACTGACTGGTATCTGAGCAATTTTTCCTGTTGCTTTTACAGAACTTCCTTCTTGTATCATCAAACCGTCACCCATTAATACAACACCAACATTATTTGATTCCAAATTCAGAGCAATGCCTATTGTACCCTCTTCAAATTTTACTAATTCGCCTGCCATTACTTCATCAAGACCGTGAATACGAGCAATGCCGTCGCCCACTTGAAGTACGGTACCTGTATTTACAATCTTGACTTCTCTATTATATTGTTCAATACGTTCGCGAATAATATTATAAATTTCATCAGCTCGAATGGTTGTCATGAGTCTTTCTTAAATTAAATGAATTCGTTTTTGGAAACAAAAAAAAATAATACCTTACCCACAGTAGAAGGACTAATCAGTTATTTCTTTCATTGCACCAAACATGCCAATATTGGCGTTGATGGTACGTAAATGTAACTCGTTGCTCAAACAACTATTCAGGGTTCCTAGAGCTCCTTGTAAGGCTTGTTGGAAAACCCGCTGGCGGACTTGATTAATCGCTCTTTGTTGTTCAAAATGAATAGTTTCGTTTTTGTAATTTTCTAATTGTTCTAAAGTTTTATAAGTTGAATTAATCAAATTCAATTTGTCTCGTTCTATTTCAGAATATCCATTCACTCGAAACTGATCTGCTTCCATTTCTACTTTCCGTAAGCGAGCCCGGGCTTTTTCTAGCTGTTCAACGGCCCTTCCGCGTAGTTCTTCTGAATTTCGAATAGTATTCACGATTCGCAGTTTTCGATTATCTAATAAATCACTTAATGAAAGTAGATTATCTTTCCATTCATTTCAAAACTTTCATGATCCCTTCCCGAACCAAACTTGAATCTTTCGATTCATTTGGCTCTCACGCTCAATTACTTCAATTTTTTTTATGGTAAATTTCCATATCTTTTTTTGAATGTAATGAACCTATCCTCTACTCTTTGTTCATATTCAAACAAAATTGGAAATGAATCAATAATCCAAGGCCAGAATATTTGGAGGACTCTTCTGACCAAAAAAAATATGTAATTGTCAGCAAAGTTGTTTTTTTTTTCAAATCCAAAAAAGATTTCTTATTTTATATTTATACATAGGTCATCGACTCAGCATTTGGCATTTAAACAAAAATGGAAAAAAAGGATGAACGTTTTTGCAATACCAATAAAAGTTTCAAATCTTTTTATCGATATGAGTGTTATATATCGATAAATTTCTAACTATTCCTTGGAAATGGAAAACCATTTCAGTATTAACATAGTGGTAGAAAGAGTACCATGCTGTGGCTGAACTTCAAACGGTTTAGCTTTAACCATGTTAATGGTTCCACATTATTGGTTGCTAGAGAATCAAAGTATATTTACCAACGAATCACGAAATGCTATGGTTCTTACATATGATTATATGATTTCTTAATTTATTCAGAAGTAATTCGCGAGATCATGCACCTTTCTTTACTAGTTATACCGAAAAGGGGTGCAGCTGGTTGATTGAATCCAGTCTATTCTTGAAATAAACAACTCGCACACACTCCCTTTCCAAAAAAAATCAATACACCAATCACTACACTTAGATTTATTGGATTTGTTGCTAAAATATCGGTATTCAACCCGAAACTCCCGGCAGATGGCCAGTGACCCATGGAAACGAAAGAATCGGTTACATTTTTCATATGATCTCCTCTTATAGATAGACTAAAAATAGATAGACTAAAAAATCGAACATAATTTTTTGTTGTATTACTTGACCTATTTCCTATTTAGAAATAGAAAATATAGAAAATAGATTCAAAATCTATTCCATTTCCCAATGTATTTTCTTTTTCAATTGTCCAATAAGAATAAGACTTATTCGAATAGAATTAGGTACCAGGTTTTCGTGTAAATTGTGAAATACCTCGTTTGTTGCACCATTTCCTAAAGAGCTTTCGTTGGACTAAACTAAGAAGGGGAAGGAAGAAAGCGAGTCGGTAACACTAATTCCTCATCCTCAAATCCGCCCTTCCCCCCGGTTTTTCTCAACGAATAAGTAATTGTAGGAGCGAAATCTTGGTATAATGCGAAAAGGCAAGCAGGCAAGCGTCAAGTCCAAAGAAATAAAAAAATACGTATTTTTTTTTTCGGATTAGGATTAAACAAAAGGATTCGCAAATAAAAGCGCTAATGCTACAACCAATCCATAAATTGTTAAAGCTTCCATAAAAGCCAAACTAAGCAATAAAGTACCTCGTATTTTACCCTCTGCTTCGGGCTGTCTCGCAATACCTTCTACAGCTTGGCCTGCAGCAGTACCTTGACCAACTCCAGGTCCAATAGAAGCAAGCCCTACAGCCAATCCAGCAGCAATAACGGAAGCGGCAGAAATAAGTGGATTCATGATAAGTTCCTCGCGCAAAAAAAAGAAATGGTTAATGATACAATCAACGAATAAATTATGACTTAATTATTCCATCAACTAAGATTCAGCCAGTCGAAGTCAGTAAGAACTCCGAATTGAAATAATAATATTCCATCAGATCATCAGAAAGACTTTCTCTTTTTTAGTTCCTATTTGTTGAGTCTTTTCTGAATCTATACAACTTGAGTTTCTCATTTCCTTCTTTCTAACCATTCTTTGAATTCTTCGACCCTTTCTTGATTTTATTCTTGATTTTATTTGTTTATTCATTCAATTCATAGTCATAAATAAATGAAAAAAAAAACATAAAAAAAGACTTCTATTGATATCCCCATCTAAATTAAAGTAGGGCTTAATATTAGTTCAGATATAACTAGTCAATATCTAATATCCAATATACATGTCTTTCTTCCATAACGTAAACCCAGTATTCTACCTTAAATTCAATTGGATTCTAGAATCATTCTTTGAATTGAAACGTCTACAAGAGTTGACTTATAGCCATTCGATTCCATATACCTAGTTCGGCCTTTCTATACTAACCAATCCCCCCTCTAATATCCCCTTTCTATGACTATAGAACATACTTGTAGGTTCCCTAAGTAGATTATCTTGAAACACACATATATTGACTTGATCTAAGAAAAAAGACTCTTTCGAAAATGAATTAATGATGACCCTCCATGGATTCGCCTATATAAGCTGCGGCTAAAGTTGCAAAAATAAGAGCCTGAATACCACTTGTAAATAATCCAAGAAACATGACAGGGATAGGAACTACTAAAGGTACTAAAGAAACAAGAACAACAACGACTAATTCATCGGCTAATATATTTCCGAAAAGTCGAAAACTAAGGGATAGAGGTTTTGTGAAATCTTCTAAGATGTTAATGGGTAAAAGAATTGGAGTTGGTTGAATGTATTTACTAAAATAACCCAATCCTTTTTTTGTAAGACCCGCATAGAAATATGCCACTGACGTGAGTAAAGCTAAAGCTACAGTCGTATTTATATCATTCGTGGGTGCGGCTAACTCCCCATGAGGTAACTGTATGATTTTCCAAGGTAAAAGAGCCCCTGACCAATTAGAAACAAAAATAAATAGAAACATAGTCCCAATAAAGGGAACCCAAGGACGATATTCTTCTCCAATCTGAGTTTTGCTCACGTCTCGAATGAATTCAAGGACATATTCAAAGAAATTCTGACCGTCAGTTGGAATGGTTTGTGGATTCCGAACAGCTATGGTGGCTGAGCTTAATAAGATAGCAATTACAACCCAAGAAGTAATAAGTACTTGGCCGTGGACTTGGAAACCACCTATTTGCCAATAGAAATGTTGGCCGACTTCCACACCGGATATATCATATAATCCCTTTAGTGTATTGATTGAACATGATAGAACATTCATATTGTCCTCTGACAGAAATATACCCTTAAAAAAAAATATTATTTTGATTCAACCATTTCTTTCTCGACTTGTCTACTTCAATCGTATATAATACCAACTAATCACATCATATCCCCAGTTATTTTTATATCTTTTTTGATATTCAGGAATCCTAACCGATTCTACTCTATTAATTCGAATTCAATTATAGAGTTCACTAAAGTCATTTTTTTTTTATTATGAATCAAGGATTTCTTATATAGCTAGAACGACCTTCACAAATGGCGAATACTAATTTGGTGAGAATTAATCGGATTGAAGCTATAGCGTCATCATTCGCCGGAATCGAAATATCTGCAAGATCGGGGTCACAATTTGTATCGATTAAACAAATCGTTGGAATTCCCAAAGTAATACATTCTCGAAGGGCTGTATATTCTTCTTGCTGATCAACGATGATTACAATATCCGGTAACCCTGTCATATATTTAATCCCACCCAGATATGTTTGCAAGTGAGATAATTGTCTCTTCAACATGGCTGCATCTCTCTTCGGAAGACAGGCCAGTCTTCCCGCCTTTTGTTCCATTCTCAAGTCTCTGAACTTATGAAGTCTCGTTTCTGTGGTGGACCAATTCGTTAACATACCCCCAAGCCATTTTTTATTAACATAATGACACCGAGCCCTTATTGCAGCCCATGCTACTGAATCAGCTGCTTTATTTTTTGTCCCAACAATTAAAAATTGTTTTCCTTTACTTGCTGCATCAAAAACTAAATCACAAGCTTCTGATAAAAAACGAGCAGTTCTTGTAAGATTTGTAATATGAATACCTTTACGCTTTGCAGAGATATAAGGTGACATTCTAGGATTCCATTTCCTAGTACCATGGCCAAAATGAACTCCCGCTTCCATCATCTCTTCCAAATTGATGTTCCAATATCTTCTTGTCATTTATCCTCACACTTTCTCTTTTTTTTTTAAGAGATGAGGTATCCCGAAATAAATAATTGTTCCGACGGAACCTTCTCTTCGACGGCGAATTGGCCATTGATACACAATCCAAACCATTAATTCTTTTCTATTCCTTATTATTTTAATAAAAAAAAAAATGCCCGTAAGAAAGACAGCACAGATAAATAGGAGGAATCCGTTCTTAAAAATGATTAAATACCCTAAACTAGGGTTTTGATGTATCATTGAAATTTTTTGAAACACAAGAATTAAATAATTCTTTGTGGTAAAACAAAATATCGTTCATTTCCCCCTCGAATAGATTCTTCTTTTTGTTTTTCAAAGGAATGCTCTTATGTTGCCTTGAACGGTGTACTAATCCTTTGAATCCGGTACCAACGGGTATCATTCCTCCCAGAACCACGTTTTCTTTTAGGCCTTTCAACCAATCGATACGGCCCCGGAGAGCAGCTTTTGCTAAAACTCGAGCAGTTTCTTGAAAACTCGCTTCGGATATAAAACTTTGAGTATTCAAAGAAGCTCTCGTTATTCCCAATAAGACGGCTCGGTAAGAGATCGCTTCTTCCAAAGCACGCCCTGTTCGTTCCGCTCGCAACAATCCAATTAGCTCTCCTGGTAAAAAAACATTAGACATTCCATCTTCTGAAACCAAGACTTTTGATGTTATTTGACGTACAATAATTTCTATATGCCTATTATGGATCTGTACCCCTTGGGATCGATAAACCTTTTGGATCTTATTAACCAAAGAGATACGACTTTGCACTATAGTTAGCTCAGCGCCAATCAAGAATCCCCAAGGAAGTCCAAGAATTCCTGTTATACGTTCGTTCCAAGCGTCAATCCTCCTTTCTAGATTTATTGATATTGACTCAAGCGAACGAACTTCTAAGACTTGTTCCACCTTTGGAAGGCCTTGCGTTATATCACCAGACCTCGATTTTTCATATATAAACGTGACTAATGTATCTCCTTCATAAATGATTTCCCCATAATGGCCATGAACAGTTGCTCCTGGGGTGGCCAAATAGGGCTTAGCTGCTCTTATTACTACAGAGTCAACTTGAACAATTAGAACTTGACCGGCCTTTAAGTGTGGCCCGTTTTTGGCTATACATACATTTTCACAAAGAAATTGTCCAAGACTTATTTTTGTGGAGGTCTCTTCACAATAATTGTGATAAAGAAAATACCAATTCAATTTGAACGGATTCAAAATAATGTTACTTCCTGGATCGGGATTATAAATCTTCCTATTTTCATCGATTAAATAATATTTCATTACTCGAAAAGTTTGTTTTAAATTGTCAAGTTGCAAATAGTTAGTTACCAAGATCTGATTATGAGTTATTAAACGGTAAAATGAATAAAAATTCGCAATTTGAAGGGCTGTTCCAAAAGGGCCCGACGAATTCCTAATTGGAATTAGAGGATCGGATTCTTTGTAATATTTTAGACCCTTGAATGGACCCATTCGAAAACAATTGGTTGATGACAAAATGATAAAAGATTGGCATTCCTTCGTTCTATTCAACAACGTACGAACAGTTTCGTGATTTTGGCTAAATGATTGTTGAAGTCTTGCTTTTGAATAAATGGAATAAAATGGATTCATACGATCTGATCCATTATCAGAAAGCAATCCTGAACCTGATGGATCATTCCTTTTTCCGGCATACGAAATAGTGGATTTCACTAAATCGATTCTTAGGAAATTTCGAATCATACCATTTGTCTTTACTTCAACAAAGGAGGCACGTGCCTCTTCGATAGAAGAACTTTTTTTGTCTTGGTCCCAATTCAATACTAAACAAGTCCGAACTAATTGAATACTTGTATCAGAAATTCCCCGAATAGGCTTGCCATTTCCATAAAGGATATAATTGACAACTCGAAGTTGCACCTTATCCCTTTCCTGCAACAGATCCTGAGGGAAAAGTGTTGATAAACTTATACCGTCCGTTATTTCATATGTGACTACGGGTCGAACCAAAACAAAATACCTTTTCTTAGTAGGTGTGATTCGTTGGACATAGATCCAATTTTTAAAATTTTTCGATTCCTTGGAATTTGTTTGTCCCCTTCCCGGCGGTATCAAAATGCCCCTGTGGCGGGATATCTTATCTGTTTCTCCAGGAAAATGGATATCTCCCGAAAAGATTTTAAGTTCAATCTTTTTTTTTTTCTTCTCTACTCGGACCAATCCGCCTACCCGGCTTCTTGTATTTAAAGTGATTTGTGTATCTACTCCAATGATACTATTGTTCCGTACCATTATGGAAGAAGATCGTGGTAAGATATGTACTTCCTCGGGAATGAAAAAAAACCGATCTACTTGCATTTTGTATTTTGCTCTAAATTCTTTGACTCCTCGATATTCAATCAAACCCTCTTTTTTTATGATTGAATGCGCCTCTATAGTCCCATATTTAGTAATTCCTGAACTATCTCTTCGGTATCGAGGATCATCAAAATAAGCAAGAATACTATTTCTACGGAAACTACCATTTATGGGTATTTCAATCGAGATACCTGAAGGGGGCATTAATTCTTTCTCTCGTTCTTGAATCGATTGAAATGGAATGGTGAATCTATTTCTTCTCCTCTTTGCCAATAAATCGGAATTTTTGGCAGGATATATGAGATTATAATGCCCAGTTCTTACGATTCGATTAAGTTCTGAATAATCAGGAATCCTATCCCCTTTATTACTAGAAAGATCCGAACTCAAAAATTTGTGTCTCATGTGAGCATTGGTCACTGAAGAGTTATAAAGATATCTTTGTTCGACAGAAAGAAAATGAGCGTTCGTTTGATCTTGATCCTTGTGGAGCGAACGCGGGGCCACAATGGATTTGTGCGGCCTTCCTGCTAATATCCATAAATGACTTGTTTTTGGTAAGAGATGAACATTACCATATGTAAATTCGGGTGCATGGTCCACGTCGGTACTCCAGTGCATTTCTCCCTCTGAGTCAGAATAAATATGTTTTCGAACCTTTTCTTTAAAATTCAAAGTGGATGTTCCCGCGCGAATTTCAGCAATCACTTGTTCTGATTCTACATATTGATCATTTTGAACTAAAAGAAAACTTTTTGGTGGAATATTCACATTATGTATAATATCTTCACTCTCAATAGTGACAGCCAAGTCTATATAACATAGAAAGGCAGGATGGCCGTGACGTGTACGTGTGGGATGAACCAAATCTTCATTGAATTTTATTTTTCCATTAGAAGGGGCTCGTACATGTTCTGCAGTACCCCCTGTGAATACTCCGCCAGTATGAAAAGTTCTTAATGTTAGTTGAGTACCCGGTTCTCCAATGGATTGTCCCGCAATAATACCTACAGCTTCTCCCAATTCGACCAGGTCACCGTGAGTAGGACTCCGGCCATAGCATAATCGACAGATCCAAGATGTACTTCTACAGGTAAAGGGAGTTCGAATAGATATTGGTTGTACTCGAAAGGTTATCAATCGATTGACAAGTCCAACCCCAATATCCTGATTTCTAGCGGCAATGCACCGCGGGCCCATATATATATCGTCTGCTAATACACGACCAATTAGTGTTTGGATAAAAATTCTTTCCGGTATCGTCCCGTTTTGGGGACTCACAGAAATACCTCGGATGGTGCCACAATCTCTTCTACGTACAACAATATGTTGAACTACTTCAACAAGTCTTCGCGTGAGATATCCAGCATCTGATGTTCGTACAGCAGTATCCACAACTCCTTTACGGGCCCCGTAGCAAGAAATTATATATTCTGTTAACGAGAGTCCTTCGCGTAAATTGCTTTGAATAGGTAAATCAATCATTTGTCCTTGTGGATCCGACATTAATCCTCTCATGCCTACTAATTGGTGTACCTGAGAGGCATTTCCTCTAGCTCCTGAAAAAGACATCATATGAACTGGATTATAAGGATCAGTCATCCTAAAATTAGGATTCATTTCTTGTCGCAAATATTCACTTGTAGCATACCATATCTCAATGGATTGACGTAATTTTTCTACCGCGTGGACATTCCCATAATGATGGTGTTTTTCCAAAATCAAACTTTGTTGCTCAGCATCTTGGACTAGCCATCCCTTAGAAGGTATTGTTAAAAGATCATCAATTCCTAATGAAATAGATGTAGCAGTGGCTTGCTGGAAACCCAGAGTCTTTACTTGATCCAGGATGTGTGATGTATATGCCATTCCAAAATGATCTATTAATCTACTAATAAGTCGTTTCATGGCAATTCCATCTATTACTTTATTGTGAAAGACCAGATTGGCCCCTTCTGCCATAAGTACCTCCATATTCCGCTGAGTGGGATTCGACAATGAATGGGTTTAAGTTAGTGATTGGAAAACTTCCTTTTCTCGATCTTAATTCGCGTAGAAATTCACGAACTATGATCCTAGTTGAACTCGGGCGAGCCGAATTCCACTGGTATCATAGAGTTACTTAGCTAGGTACGATATAATTAAGTACCATATGAGCAGGCTCGATAAAATCCTTGTATAGCTTCTTCAATTTCTCGATAAAACGAAATATGACCGACGGTTGTTCGAATGTATATAAAAAGAATTTCTTTTTTTACACTTCTTACTATTAGATAGTGCCCATAAATCTCATGATAGGTACCCAAAGATTCATAGTGAACTTCGATGGGAGCTTCTCTTGAAGCAACAACACGTTGATCTAGTTGCCACCGGAGCCATAAAGGACTATCTAAATTGATTCTTTTTTGACGATAAGCCCCAATTGCATCATAGGAATTACAAAAAAAGGGTTCTTTCATATACTTATAATTATGATCGTTCAATTTTTCATTTTGATAGTTTCTTCGACTCCATGGATTATATCTATTTGCACAAATACCTCGACGATTCCCACTCGTTAATACATAGAGCCCAATAAGCATATCTTGAGTTGGTACGGAAATGGGATCCCCAATAGCCGGAGACAAAAGATTCATATGAGAAAACATAAGTAAACGGGCCTCCGCTTGGGCCTCCAAAGATAAAGGTACATGAACAGCCATTTGATCCCCATCAAAGTCTGCATTGAATCCTTTACAAACCAATGGATGTAAACAAATAGCACGCCCTTCCACTAAAATGGGTTGGAATGCCTGTATGCCTAATCTATGCAGAGTAGGCGCTCTGTTTAACAATACAGGATGCCCCCGCATAACTTCCTGAAGTATTTCCCATACAATGGGTTCTTTTTCCCGAATTTTACTCTTAGCAACTCCTATGTTCGAAGCAAGATCTTGTCTAATTAGACCGCGAATTACAAATGTCTGGAAAAGTTCTATTGCAATTTCACGAGGCAATCCACATCGATGTAATGAAAGTGAAGGACCTACAACAATGACGGAACGCCCTGAATAATCGACCCGTTTGCCAAGAAGAGTCTCACGAAACCTTCCCTCTTTACCTTCAATTACATCAGAAAATGATTTGTAAACCTTATTATGACCATCCCTCATTGGTTGTCCGCGTATTCCATTATCCAGAAGTGTATCTACGGCTTCTTGTACCAATTTCTCCTGACACATTACTAATTCTCCGGGCGTAGATCTACTTGTTGTTAATAGATCAGTAAGAGTATTGTTCCGATAGATAACTCTTCTATAGAGTTCATTAATATCCGAGCTCATTAATTTACCTCCATCGATCTGAATGATCGGTCTCAATTCGGGAGGAAGAACAGGTAATAGACACAAAACCATCCATTCTGGTTCTATATTTGTTCGAATGAAATGCTTAGCTAATTCAATGCGTCTAACCAAAAAATCCCTTCTTCTTCCAACTTTTCGATCTTCCCATTCATTTCCTGTAGGCCCTTCTTCCCCTAACTCTTTCCATTCTGCCAACGAAGAATCGAGAATAGTTCGCAAATCCAAATCGGCTAATTGTTCTCGGATAGCACTTGCTCCAGTAGATATTTCTCGATTTCGAAAGGTATCGAAGCCTTGGGTAGTAAAAAAAAGCGGGATGCTGTATTTCCAAGATTGGATTTCATATTCGAATAAACCTCGTAATCGTAAAAAAGTAGGTTTTTTAGCTATGGGCCTAGCAAAAGAAAAATTGGGATAGGATCCTATACTATAAGATCTCCCCCCTTCAAAGCCGGACGTGAAAGTTTCCTCTCATCCGGCTCAAGTAGTTACAGCAAAGAAAGAAAGGAGTTCTCGCTTTCAAATTCTAGAAATTCTGGAAAATCCTCAAAGCAAAACAAAAGATCTACTCCTTACTCAAGTTCCCGTGGAAGACCAAGCAACATTTCATTAATTCATTCTTCTTTTTATTTATATTTTTTTAATTCTTTATTCAAGTCAAAATGAAATGTGAAATTCTTGAGTAGTCTACCTCCCTTCGAACGATGAATCCCCTTAAAGGAAAAAAGGCAGTGCCTTGGAATTCATAAGATATAAGATAAAGGATTTACTTGTCTATGTATCGTTCCATTCGATCTTTTAGGTCACGACTTCACCTCGACGGTTATACTACGATGCCCTTAAAGCCTATATGCGATGGATAGACTCTTGTAACCATGACATATTTGCTATTTGCTTGCGTGAACATAATTTATTTCTAAACGACGGAGAGTGGTTAATTCCACAAACAAAAGAAGTCTTTTTTTACGAGGTACAACTAGAAATTCAAAATTACTTGTTACGAAATCGACCATGGATCAATTCCCTTTTTATTTGGGAGTATTGAATACACCCATAATTCTGAGCTTCATGTTACTCCTAACAAGAGACATGTCAGAGCCAGGGCATCCCAATTAGATGGAATGGGATAACAGTTTCTCATTTCGAATCTGTAAAATCAAAATTTCGATCAAATCACACATCGCAGTATACTAGACCCTCTAATTCTTTAAGAGGTTTATCTAAAAAATTCGCAATATAACTAGGAAGACGTTTCAAATACCACACATGAGTTACTGGGCACGCCAATTTTATGTAGCCCATTTGATACCTTCGTATTCGAGAATCAACAAATTCGACCCCGCATTGTTCACAAAATTTCGGTTCTTCTTTTTCATCTCCTATTACTCGATAATTTCCACAAGCACAAATTCCGCTTTTTATAGGCCCAAAAATTCTTTCACAAAATAATCCATCTTTTTCCGGTTTATTGGTTTTGTAATGAAAAGTATAGGGTTTTGTGACCTCTCCAACTATCTCTCCGTTCGGTAGGATTTTCGTGGCCCACGCACTTATTTGTTGGGGAGAAACTAATCCAATTCGGAGTTGTTGATGTTTATACTGATCTATCATAGAAGAAAAATTTGAATGAATTCCGATTAAGCTTCCATCCTATTAATCTGCAAGTTCTTCTCAGATACAAGGAAATGATTCAGTTCCAGAGCCAAAGATCGTAGTTCTCGAACGAGCAATCGAAAAGATTCTGGAGCATCTTCAGGGTTAGGTATTGTTCCTCCAATGATCGTAGTACCAAGTACTTCCTGGCGAGCTCTAATATGATCAGATTTATAAGTAAGCATCTCTTGTAAAATATGAGCAACACCAAATCCTTCGAGAGCCCAAACCTCCATTTCTCCTACCCGCTGTCCCCCTTGCTTGGCCCTTCCTCTAAGGGGTTGTTGTGTAACAAGTGCATAATGTCCACTAGAACGTCCGTGGATTTTATCATCAACTTGATGGATTAATTTCAAGATATAAGGATTTCCGATTATAACAGGTTGTTCAAAAGGATCTCCTGTTCTTCCATCAAATATTCTGCTCTTTCCCGGATACTCGGGTTCAAATACCCATGGATTGGCTGTTTGCTTACTGGCCTCATATAATTCAGAAAACACTAGTTTTCGCGAAGCCTCTTGTTCATATCTCTCATCAAAAGGTGCTATTCGATAATGTCTGCCTAGCAAACCTCCCGCTAATCCGAGTGAACATTCAAAAATTTGCCCTACATTCATTCGTGAAGGTACTCCTAAGGGGTTAAAGACCATATCAACAGGTCTTCCATCTTGCAAATAAGGCATATCTTGTCTAGGTAAAATTTTTGAAATGATACCTTTATTTCCATGTCTTCCAGCTACTTTATCACCTACTTTTATTTCTCGTTTCTGTGAAATATATACACGAATCGTTTCCGGATTAGAACTCGAACCCCCCTTTTTCTGGATCCATCTCACATCAATAACTCGACCCCTACCACCTATAGGTAGTTTTAGACAAGTTTCCTTTGAAGTGGATACCTGAATGCCAAGTATGGCGCGTAATAATCTATCTTCCGGGGCATAGGATGATTCTTTTGCCATCTGAGGCGTTAATTTACCTACCAAAATATCGCCTGTCTCTACCCATGATCCCAGCATCACAATTCCATTTTTGTCTAAATTGCGGAGTAAATGGGCTTCTAAATGTGGTATTTCGTTAGTTAACCTTTCGGGACCTTGACTTGTCACATGAGTCTGAATTTCATATTTTCGTATGTGAAAAGAAGTATAAATATCTCCATATACAAGACGCTCACTAATGAGTACAGCATCTTCAAAATTGTAACCTTCCCATGGCATATAAGCTACTAATACGTTTTTTCCCAAAGCGAGTTCGCCCCCAACCGTGGCGGCACCGTCCGCTAAAATTTGTCCCTTTTTAATGCATTTACCCTGCTGAATCTGGGCTTTTTGATGCATACAAGTATTTTTGTTGGAACGTTGATACATAACTAATGGAATGCTTAAAGTATACCCATTACCTGATAAAATGATCTTGTCAGTATCGGTATAAATGATCTTTCCCTCATGTTCGGCTATAGCGAGAACCCCTGAATCTAGAGCCGCTTGGCGTTCCAACCCAGTTCCAACAATGCACTTCTCGGAATGAGAAAGCGGAACGGCTTGACGTTGCATATTCGAACTCATTAAAGCCCGATTCGCATCATTATGCTCGATAAAAGGAATGAGGGAAGCTCCAATAGAAAAATATTGGAAGGGAAAAATACTTCGAAAATGAACCTCTTCCCATGCACTAGTCAGGAATTCTTGACGGTATCGAGCTGGAACAACCCCCTCTTCCTGAATACCTCGATTCAAGGCCAAAGAATTTCCTGCCGCTACCATATAGTATTCATCTCTACTTGGTGATAAATAAAGCATCTGTACCCTTTTTGATTTCTCAGAAATTTCATAAAAAGGGCTTTCTAGAGACCCCCAAGAACCAATCCTAGCATGAATTGCTAAGGATCCAATAAGTCCAACATTGATTCCTTCAGACGTGTCAATTGGGCAAATACGCCCATAGTGACTAGGATGAATATCTCGTATACGAAAACTAGCAGTTCGCCCCGTCAATCCTCCAGGGCCCAAATAACTCAATTTTCTCCCATGAACTATTTGTGTCAATGGATTAGTTCGATCCAAAACTTGAGATAATGGATGTAACCCGAAAAAAGATTCATAAGTGGTTGTTAATGGAGTTGAAGTTACCAAATTCTGAGGAGTCGGTATTAATTTATGCCTAATTGCTCCACATATAGTTCCTCGAACCACATTTTCTAAACGAACCAGAGCCAATCCAAATTGATCTTGTAAGAGATCCGCCACAGAACGAATACGTTTATTTTTCAAATGATTCATATCGTCAAGTGTACCCATTCCAAATTTCATTCCAATCAAATGGTCTGCAGCTGCCAATATATCTCGCGGTAACAAAAATGTATTGTTTTGAGGTATATCAAGGTTCAGTCTCTGATTCATATTTCGTCGACCAATCCTTCCTAATTCGCATCTTTGTTGAAAGAATTTTTTTTGTAATTCCTTACATAAGGATTCAGAAAATACCGGGTCCCCACCTACACAAGCAAATTGTTGATAAAACTCCAAAATGGCATTTTCTTTTGATCCAAAATTTTTTTTCTCCTTATCATTCAGGAAAGACAAGAAAATTTCAGGGTAGCAAACATTCTCTAGAATTTCTCTTAGATTCGAACCCATAGCTGATGATAGAACTAGAATAGATATTTTCTGTTTCCTACTCACACGAGCCCATATCCTTGCTTTTCTATCAATCTCTAATTCTAATCTTCCTCCCCAATCTGATATTATGGTGCCGGTATAGACCGAAATTCCGTTATGGTCCAATTCTGACCGATAATAAATACCGGGGCTTTGCAGTATTTGATTGATCACAATTCTGTATATTCCGTTTACTATAAAAGTTCCCAGGGAATTCATTAGAGGAATGTTTCCAATAAAAATTGTTTGCTCTTGCATATCCCTACTGGTTTTCCAAATTAACCCCGCGGATACATATAATTCAGAAGAATATGTAAGTGATTCATACACAGCATCTCTTTCTTTTAGCAACGGTTCCACCAATTGATATGTTTCCACAAATAATTGAAATTCAATTTCTTGATCTGTATCTTCAATCTTGGGAAACTTATAAAGTTCTTCCGTCAAGCCCTGATCAATGAACCTACAAAATCCTTCAAACTGTATCTGATTAAACCCAGGTATTGTCGACATTCCCTCATTTCCATCCCGGAACATTTGAAATGAATTTCCCATTTATAGAAAAATCCCATTATTAGATTAGCGCATTCTTCGTCGAATCATATAGATCGACCCAACGCCGATGGAATCTATATTCTGTTTACTGAATCACATAAAATTTGACCCCAATTCCATACCAGATATGTCCATATATGGAATGTATGAAATACGTATGAACGGGGGAATAGAGAGAATTTTCTACTCAAGTAAAATTTTGGAATTGAATTTGTATTGTAAGAGAAGAGATGAAAGGAATTGATGAAACATTCTTGGAACCAGAATTCTGCTGCTTAGATTTATGTTATGGGCTTGACTATAGAATATCAGAACAAAAGTGATTCAATTACTACTATTATAATGATATTACATATTCCAATCCGATTGGATACCGGAAAAATAAACGGATTCGGGATTTTATCTGTTCGCCGAGATAAATACAGAATAATGAGAAACAGTACAACGTTGATTTTTTTTCTCACTTAACCCCTTGGGGGATTCCATTGTTAAAAAAAAATTGCCGAGAAAAGAAAGAGAAACTTTGACCTATTCTCTTATTATATTATTACTAGACTAGAATTCGTAAAATACGCTTGGGAAGCGGGTTGTATTTAGTTGTATTTATTAAACATGTGTAGCTATCTTCTATATATCCCTATATATTCATCTCCCCTTTTATTGCAGTTTTATTCGGGGCAGCACGGGCGGTGTTCTATCCAAATTTATACTTTTTTTCGTCAATCGATTCAATGAAAAATTGAAGTACGATATTTTCTGATAATTCCCTACGGGCATAATATATTATATTTGAATACCAGATTATATATCTGTGTAATTTCTGTTCTGGTTCCGAGGTTTCCTTTACATATATAAAAATATATTTATATAATCGAAATTGAGAAAAATGGAAGTTAAGAAAGATTTTTGATTGAAAAGAATCAATAACAATAATAAAAAATTAGTTATTATTTGGACTTTCTTATGTCATTAGGGAAACATAATTTGAGATCCAAATCTAAGAATCATTCATGAATTCGCAGTCAAGTCACAAAATAGTTAATGGTTCAAATTTCTCATGAATTTTTTTTGTGACTGAAAGTCCACATTTTCCGTTTCAATAGAAAGGATAAGGGAAGTTTTTAGGTATTGCGTATTTTGCGATACTATACAATCAATCGAGGGGGTGGATCTAATCAAAAAGGGGAATGGTTTCTTTTGGTTAAGGCAAAGGGGATTCAAGATGCAAGTACAAAAAAAAAAGAAGTTCAGTAATCCACCCCAAACCAAAAAAGGGGTAATATTGTCATCTATTTTGTATTTTTGGCGACATGGCCGAGCGGTAAGGCGGAGGACTGCAAATCCTTTTTTCCCCGGTTCAAATCTGGGTGTCGCCTGATCAACAAAAGAACGGAAATCACTTTTTTATTGATTTATCGATATAACTCACCGAAATATTCCCCAGCAGAGGGGGAGGAGGGAGCTATTGATACGCGCTTGATTCGAAGCATATGGAGGTTCTCGAAAGATCTGTAAATTTTTGTGTCTAGGATTAAAAAAAAGATTTTCGTACTATGAAGGGAGTCGATAAGTCTTGATAGCCCTTACACTACTAATGGAATTTTGACTGACTGGGCCTTGAATTAGATTGGATAACCAAAGGGGAGTCTAACTAATTAGTAGAATTATGGAAAAGGAAGAAGCTACTTTGGTCTACAAACTCACGAATGTCTTTGAGTACTCAGATACTCAGATATTCGATCAATATTTGATTGTATAATTCAGTAATTCAGTTTTTATTTTATATAAAATAAAAAAGTGCAAAAAGAACTTCTTTTCAGTAACCCCTAGTCAAGAATATAATCGACTGTCTCCCCATTTTTTCACAGAATTTTTTCACAGAGACGAAATAGATCAAATGGGAAAGGAAATAAATAAAATAGAGGTATGTGATAGATAATGATCTATCTTGGTTATATAGTTTTTATTCCGTTTTTTTTTATGAATGAATTATGAAGGTTAACAAAAGAATAGGTCTTTTTATTCCTAAATGCTATATTAGTAAGACTCCCTTGTCCACGGGAGTCGTTGCATATTTTGCTTGTGCTTAATCTTTCCCAATTATACTAGAAATCATAATGATAAGAAAATTTGTATAAGAATTTATTAGAATTTATTATATTATAAGTGATAAGTGCATTTATTGGTTTATTGGATTGGTTCATTAAATAAAGAGTTTGGGGTAAGAATTCCCTTTTGACTATGCACCCTGGATTTCACTATTATTAGTGAACAAGAATGGAATAATTCCTTCATATTCATAGAGATAGGGGACATAATTCACATGGATATAGTAAGTCTCGCTTGGGCTGCTTTAATGGTAGTCTTTACATTTTCCCTTTCACTCGTAGTATGGGGAAGAAGTGGACTCTAGAAGTACAATTAATGTACTTTCGGTAAGGAATCAAACTTTATCAATTGTTTTATAGATCATTCTACAAAGCGTTCTGTTTTAAATGTCAATCAAACAGATATTTCAATGATTCCCATGTTTGTATTTCGGAAGGGGATATGTATGGTAAGAAATTTTCATTTTCCTAAATTCTCTATTTCGCCGAACGGGCTCTTATCAGACTTTCTTATCAGACTTATATAGGGACAATGAGGAACAACAGACCACTTCTTATTATTTGTTATTTGTTTCCCTCTTTATCCAAGAAAAAGGCGTTATGTTATTAAGCGGATGCGTACTTTCTAGGGTAAAGAACATATACATAGTGGTTGTTCAACAAGATACTACACATAATAAAATCTTGCCCCGGTCGAGTCACATATTGTGTACTCACCGCTTGCTTTATTGTTGTAGAAATTGGATTTATGCTTTATCGACATCGACTCATTTCATATCATGGTTCAAGTGTTACAAATTGGTAGGGTTTACTACTCCTTTTCGAAATTCGAAGAAGTTCCCATACTCCTTTCTGTTAGCGGTTCAATCAAGTACTTCTTTGGAATGCTAAAAAAAGATTACTGACTTTTATTAATAGGCGTCCTGCCCATAGACTTTTGACTTCTTTGATTTTCTTTTTTTGATGGATACTGGGATTTCGATTTGAAATAATCAGAAATCTCGGAATTCAAGCCGTAAAAGTAAGAGTTACCTTTTGATTATTTCGGATTGATCGGAATCAATACAAATCAATAAAATAAATGTAGCAAAGAAATAGAACTGGGGCCCTATGTATATTCTATAGATATAATTCAGATAGAATTCTATCGATATGAAGATAGATATTGTAGATTGATCTATATTAAGCCCGTATCTTTATACAGTACAACTTTATACACTACAAAACCGCTAATCTAATAGATAGTATGGTAGAAAGATTGATATATTCCTTTCTACCATATTATCAAATCTCATAGAATACTGTTGATTCTAGCCTGCGTATTTAATTGAAGATTTAAGAAACGAAATTGGAATCCTTTGTTTCTTTCTTAAATCATTATCATTGATAAAGACCTAAGAAGTCAAGTTTCATTCAGATTAATCATTTTGGCTGACCGTTTTTACATATATGATAAGTAAAAAAGCAGTAGGAACTAGAATGAAGAGTGCAGTAGCAATAAGTGCGAGAATATTTACTTCCATAATCTAATTGGTTTTTACTTCGCAATAACTCGGGATTTAATCCCATAGAGATAATAAAAATTTCGCCTGTAAATTCAATGGGATGAATTACATCTCGATGGTATTGAATCGCATCAATATTATGAATAACAATATCTGGGCTATCAAATCACTTCCTCGTCGCGAATTGAATAGTATAACATAGGAAGATCTTTTATCCATACTCAATAGAAAATGGAATTCGTAATCGAATCAAGAAATCTTTTTTTTTATTATTCTTTTACATTCTTTCCTTTCTACAACCTACCGTCTTCCTTGGACAATCGTCGGATGAAGTATCATCTGACCGTTTTCCACTTACATTGCATTCATAACAAACCCCACAAAAAAAATAACAATAGAAGTAAAATGAAAAAAGGGGGGGGAGAGGACTTAAGCTCGAAACTCCTATTTTTTTTTATGATATAATTTTGATATAATTTTCTTTTTCTTGTAAGAAAAAGAAAAGTGTGAAAAAGCCAAATTCCGGTATAAAAGATATAATCTTCTATCAAATTCTTTCATTTTAGGCTTTGTTCTTTTTTCGATAGCACCTTTACTTTAACTTTCTAATAAGAAGGAAAAAGATTATTCATTACCTCATTAATATAATAATAAATAAGGCTAAAGGGGGTGGTTGTTTGATCTTTCTTTTATTAATATTCTCTTTTCTTGGATTAGTACTAGCATATATTAAAAGTTCGGTGTAAAATTTGAATGAGATAGATTTTAAAAAAGGAGTTAGTTTGAGTCATTGAGACTACACAAAATCGGAGCTAGAAAGGGAGAGAGTTTGAATCTGAAAAGTTTTTTTCGGGGTAACTCGAATTCCATTTTGTAGTGTACAAGAAATGAATTCTAGTTGTGTATGTGCTCCCGAGAAACATATGATACTCTATTCCATTAGGCAATAAATTTTGAATTTAAAGACCAGACCCAGTACGCTATCTCTTGGAATCCTGAATATGATGTTCCCAATAATTGGACTAATCCAATTATATCTCTCTCCCACCAATCGGTACTAGTTGAAGTAATGAAAATTTCTATATTTGTTTGTGGAAATAAAGAAAAAAGAAATAAAAATTCCTATTGAAAATGACTGAAATTCTATTCATTGTGTCTCTTTTGCCAGAAAATGAAAATGGAGAGATGAGTTGATGTGTTTATTGGATCCGTCGGGACTGACGGGGCTCGAACCCGCAGCTTCCGCCTTGACAGGGCGGTGCTCTGACCAATTGAACTACAATCCCAGGGAAATAAGGTATACCGCATTAATATTTTTAGGATTTCATTCAAACCCATTTTTATTTATCATTTTCGTGTTGTAAGAGAGACACGAGTGATATAGTGATATCTACATGGCTATGCACTTTCTTTTTAGTGATAGCGACACGAATTACATTACTAGTGATCCTTTCCTTATTTCCAAATCGATTGATAATCAATCTTTCAATAAAAAAAAATTAATTTCTCGTTTCCTTAGACTTTCTTTATACTTACAGATACTGATATGAATCTAGATCATTATATTATCTAGATCCGAATTTCTTGGAACAAATAAATCGAGCAAACAAATAGAGGTAGGTATATAGAAAAAAATGGAATTCTTTTATTCCCACGTATCATGCGCTTCGGGAAGACAAATATTTGAATCTCACGGTCTATGAGCGAATTCTTGGGCCGAGCTGGATTTGAACCAGCGTAGACATATTGCCAACGAATTTACAGTCCGTCCCCATTAACCGCTCGGGCATCGACCCAGGAAAAAGAAATTCCATTTTTAATTTTAGGCTTATTGATAATGCACGATCAACTTCCTTTTGTAGTACCCTACCCCCAGGGGAAGTCGAATCCCCGCTGCCTCCTTGAAAGAGAGATGTCCTGAACCACTAGACGATGGGGGCATACGTGTCCGACCGCCATCATACTATGATCATAGTATGAACAGTTTTTCCAAATTGTCAATAGAGTCAATAGAATGTAAGAATATGATTCGATCCAAGGGATCCTTCCGCCCTTCACGATTCCATAGAGTTTTTTGATTCGTCATTCCTATTTATGAATCCTTAATTCTAACCGCCATTCCATTCGATTCGATATATAAAGTCATTATCATTATCCATTATATTATTCATATTTTTTTTATTTAATTTATTAGTTATATTTATTATATTTAAAAAATTTTATTTAATTATTTAATATTTAAAAATATTTATATTTATTATTTTATAATATTATTTATAATATTATATAAAATATAATATAAAATATAAATATTATAAAAACTTAAAAACGAATACTAAATCTAAAAATCGAATATCTAATATAATTCGATCTAAAATTCTATCTAAATCTAAATCGAAATTAAATAAAAATAAAATATGAAATAAATAAAATGAAAATAAAAATAGTAAAAATTCGATTAAATATCTATTTTTTAAAGCATTTCTTATAAATAGAACTAAATACGAAGGAAAGGATCCCTTTGTGGAATGGTTTATACACCCCAAAATCAAAATACAACTTTCAACTCCATTTCTTCCACTTTCATTGATTCATTCATTTTTTTTTTAAGATGAAATGTGCCTATCTCTATTTCATAGTAAACCAGAAAATTAATAAAGGATAAATCCGAGATGATAAAGGGGATCAATAAAATCTCGGAAATTGTTTTTTATTGATTAAGGGGACAAATCGAACTTCTCCATCACATGATTTAATGAAATATCTTGGATCTATGTCGAATTGACATGTATGAAGTGATTTTTGTTCTGATGGGGATCGATTTAATAAAAAAAGAAAAAAATTAGGGTCGGCTTGGTTCATTGAAGTGATAGTTTAAAAAGATCAATAAATCATTTGATCCTAGACTCGATAGTAAATCAACTTTATCATTCCGGAAAGAGCCACCACCGCTATGAATTTACTTTATTATATAGTATTATATAGTATAGTCTATAATAACTTATTATGTAATATATGGAGATAGATATATCTTATCCGCATAGTGACGAATTCAGTAAAAGGGCCCCTTTAACTCAGTGGTAGAGTAACGCCATGGTAAGGCGTAAGTCATCGGTTCAAATCCGATAAGGGGCTTTTTACTTTTTTTTCATAAAACCCGAGTCGTAGTATTCATATTTGAACGTAGAATAGATTTGCTTCTTGCTATTTTTAAAGGAAAAAGTAAACAACTGTATAATAAGTTCTAGTAGTTATAAAGTTGAACATTTATTCATTATACTGAATAATGATAAATGATAAGAGAAGTCGTCTATTGAATCACCAAATATTCCTATTTTTTTTCAATTATTCCAACCTAATCCATTGGAAAGATTAGAAATCAACAAATGAAAAGGGAAAAGTAAGTGGACCTGACCTATTGAATCAGGACTATATCCGCTATTCTGATAATCAAATTAGATAGAGATGAAATTGGAACGGTTGACCCCTTGTTTCTTTCATTTCTTTGGACTGCGCACGAATTTGTCGATATTTCCGATTATATTTTTGTATTCCTAGATATTCCATAAGAACCATAAGAATGAGAATAAATTGGCTATTCCCTTACTTCATAGAGAAGGAAGGGAAAAAGTTTCTTCTAAATCACAACATAAAAAAACCCTTTTTCGCTATCTTTCTTTGATTCCAGAGAAGGATTAATATCTATTTATAGAATAAGATTTAGATATATCTATTAGATCGTAACTTCATGTACCAACTATTTTTAGATCGATGCATCCCATATTTTTGTTTCGACAATGTGATGGAAAATACATGCGGGAAAAAACTTTCATTTCGGGTCTCCTAGTATTTTTTTATTATTGAATATTGTATGGAATATTGAATATTGTATGGAATTTCATTAAGTTAGGGAAAAAACAGTAATTCTCTCTTTTTCTTTTCTGACAGATAAAAAGTAAATAGAAAATTTTTCATTCTTTCCTCGACCCATGAAAATTAAAAAAGAGACTCTGAAGTTTTCGATTCATCTAAAGGAAAGGGAAAGCGGGCAGAAAAGAAATATATATAAGACTGTAGTAGCATACATATAAATTCGAAAAATCTCTCAAGATATTTCATTTTTTTATTAATTATTAATCTCACGAAAAAGAAAAAGATAAGTTAGTTGATGGAAAGCAGGGGTCGGTCTCGGAATCAACTGGTCACAAGAACGAATATTGCTTGTTCCTTGAACAGTTCTTTCAAAAATTTTATATATTTGATTGATGAGACATAAGACAATTCATGGGTCAGGTGCTTATTCAGACTAGGAAAGAATAATCGAATTGAGGTTAGGGATTTACTTTAACTTTAGGTAAAGTAATTTTATGAACCAATAAAGTTTATCTTCGAAACCCATTGTAAGAGTCAATGTACGAGAAATAAAATCATACATAAATGATCGAATCCTCGGACTCCGTGCTATGAGGTGTTCGGAAATGGTTGAAGTAGTTGAATAGGAGGATCACTATGACTATAGCCCTTGGTAAATTTACCAAAGACGAAAATGATTTATTTGATATTATGGATGACTGGTTACGGAGGGACCGTTTCGTTTTTGTAGGTTGGTCCGGTCTATTGCTCTTTCCTTGTGCGTATTTCGCTTTAGGAGGTTGGTTTACAGGTACAACCTTTGTAACTTCATGGTATACTCATGGTTTGGCCAGTTCCTATTTGGAAGGCTGCAATTTCTTAACCGCAGCAGTTTCTACTCCTGCTAATAGTTTAGCACACTCTTTGTTGTTACTATGGGGCCCTGAAGCACAAGGAGATTTTACTCGTTGGTGTCAATTAGGTGGCCTGTGGACTTTTGTTGCTCTCCATGGCGCTTTCGGACTAATAGGTTTTATGTTACGTCAATTCGAACTTGCTCGATCTGTTCAATTGCGACCTTATAATGCAATCGCATTCTCTGGCCCAATTGCCGTTTTTGTTTCTGTGTTTCTAATTTATCCACTAGGTCAGTCTGGTTGGTTCTTTGCGCCTAGTTTTGGTGTAGCAGCTATATTTCGATTCATCCTCTTTTTTCAAGGGTTTCATAATTGGACGTTGAATCCATTTCATATGATGGGAGTTGCCGGTGTATTGGGCGCCGCTCTGCTATGCGCTATTCATGGTGCTACCGTAGAAAATACTTTATTTGAAGACGGTGATGGTGCAAATACATTCCGGGCTTTTAACCCAACCCAAGCTGAAGAAACTTATTCAATGGTCACTGCTAACCGCTTTTGGTCCCAAATCTTTGGGGTTGCTTTTTCCAATAAACGTTGGTTACATTTCTTTATGTTATTTGTACCAGTAACCGGTTTATGGATGAGTGCTCTTGGGGTAGTCGGTCTGGCTTTGAACCTACGCGCCTATGACTTCGTTTCCCAGGAAATCCGTGCAGCGGAAGATCCTGAATTTGAGACTTTCTACACAAAAAATATTCTCTTAAACGAGGGTATTCGCGCTTGGATGGCGGCTCAAGATCAGCCTCATGAAAACCTTATATTCCCTGAGGAGGTTCTACCACGTGGAAACGCTCTTTAATGGAACTTTAGCTTTAGCCAGTCGTGACCAAGAAACCACCGGTTTCGCTTGGTGGGCCGGGAATGCCCGACTTATCAATTTATCCGGTAAACTACTCGGAGCTCATGTAGCCCATGCCGGATTAATCGTATTCTGGGCCGGAGCAATGAACCTATTTGAAGTGGCTCATTTCGTACCAGAAAAGCCGATGTATGAACAAGGATTAATTTTACTTCCCCACCTCGCTACTCTAGGTTGGGGGGTAGGCCCTGGTGGGGAAGTTATAGACACTTTTCCATACTTTGTATCGGGAGTACTTCACTTAATTTCCTCGGCAGTATTGGGCTTTGGCGGGATTTATCATGCACTTCTGGGCCCTGAGACTCTTGAAGAATCTTTTCCATTCTTCGGTTATGTATGGAAAGATAGAAATAAAATGACCACAATTTTGGGCATTCACTTAATCTTGTTAGGTCTAGGTGCTTTTCTTCTAGTATTCAAGGCTGTTTTTTTTGGGGGCGTATATGATACCTGGGCTCCAGGAGGGGGAGATGTAAGAAAAATTACTAACTTGACCCTTAGCCCAAGTATTATATTTGGTTATTTACTAAAATCGCCTTTTGGGGGAGAAGGATGGATTGTTAGTGTGGACGATTTGGAAGATATAATCGGAGGACATGTATGGTTAGGTTCCATTTGTATACTTGGCGGAATCTGGCATATATTAACCAAACCTTTTGCATGGGCTCGCCGCGCACTTGTATGGTCTGGAGAGGCTTATTTGTCTTATAGTTTAGGTGCTTTATCTGTCTTCGGTTTCATTGCTTGTTGCTTTGTCTGGTTCAATAATACCGCTTATCCTAGTGAGTTTTATGGTCCCACTGGACCAGAAGCTTCTCAAGCTCAAGCATTTACTTTTCTAGTTCGAGACCAACGGCTTGGGGCTAACGTGGGATCCGCTCAAGGACCTACTGGTTTAGGTAAGTATCTAATGCGTTCCCCAACTGGAGAAGTCATTTTTGGAGGAGAAACTATGCGTTTTTGGGATTTGCGTGCTCCTTGGTTAGAACCTCTAAGGGGTCCAAATGGTTTGGACTTGAGTCGGCTGAAAAAAGACATACAACCTTGGCAAGAACGGCGTTCCGCGGAATATATGACTCATGCTCCTTTAGGGTCCTTAAATTCTGTGGGTGGCGTAGCTACCGAGATCAATGCAGTCAATTATGTCTCTCCTAGAAGTTGGTTAGCTACTTCTCATTTTGTTCTAGGCTTCTTCCTATTCGTAGGTCATTTGTGGCACGCGGGAAGGGCTCGTGCAGCTGCAGCAGGATTTGAAAAAGGAATTGATCGTGATTTTGAACCTGTTCTTTCGATGACCCCTCTTAACTGAGACAAGAGATCCAATGCTTAAAGTAGGAATCATTTTGATTCCACCATACATATTGGGATCGGGTCATACTTAAAGATTAGTCCCTTTTTTCTTTATTTTATTTATTTTTTTTGTTTTTCAACTCATATCATATAATAATTCACTATATATATAAATATAAATAAAATTGTCGTTCTTTTCTGGCTCGGCTAGGGTGAGCTAGCCGAGCCATTCCCTTTATTTTAGTTTACCGGGACGGGCAAAACCACTAAAATAAGGAAATCAATCTATTCAACGAGCAAAAGGAGAGAGAGGGATTCGAACCCTCGATAGTTTTTAGAACTATACCGGTTTTCAAGACCGGAGCTATCAACCACTCAGCCATCTCTCCAAAAGACCATTTCTATTTTATTTTGATTCCCCCGAATAGAACATGGTCATACGGGTTGATACCATTATTATCTATAGATAAATATCAGGTGTGAAATCGATAGGTCGATCTATTTATCTGTAGATACCTCTATATATAGAGGTATGATCTAGCCTGCCCATTTAGTGAAGTAAAAAAATTCCCCGCGACTCGAATAAAGTGGTAAAAGGTGGTAATAAGTCATAGTATATAGAATCAATATATTCATATTAAATTTTTAAAATCCCTATATATATAATATATAATGCATTTTTTACTGATAGAGGGATCAAATGGTCTAGTTCATTTGTTGGTAGCGTGGAGGATTATAAGTATGACTATAGCTTTCCAATTGGCTGTTTTTGCATTAATTGCTACTTCATCAATCTTACTGATTGGTGTACCCGTCGTATTTGCGTCTCCCGATGGTTGGTCAAGTAACAAAAATTTTGTATTTTCCGGTACATCATTATGGATTGGATTAGTCTTTCTGGTAGGTATTCTTAATTCTCTCATCTCTTGAACCTATTCGTCCCAGATCCAAAAATGACCCCTCCCCCGAATTCGAAATTTTTCGGGTTGTGAGACAGAGTAAAATTCAATCAATATAAGTCCCCAAAATGCAAACAAATACAAATAAAAATAAAGAAAACACAAAAAAGGGAGGGGTCAAACTTAAACTTCTTGAATGAATTCAATGAAATAAAAAAGATTGCAAATTTCATTGGAATCACCTTGATTGAAGAAAGTATCTGGCCCAGCTCTGCACAAATATGGTCAAGACATATATACTATATATGCGGACATATTCCTTCTCAAGAAGGACAAAATGCGGATATAGTCGAATGGTAAAATTTCTCTTTGCCAAGGAGAAGACGCGGGTTCGATTCCCGCTATCCGCTCAAGGTTCAAGATGAAGTTATTAATATGATATGATTAAGGGATTGGGTATAGTTGACCGGGATAGTGTAGTGATTCTATCTTCCTCCTTCGTTTTTTTCTACCACCCACCCAAAAACAAACAAAAGAAAAATAAAGTAATAAATTAAGAATAAGAATTTTTTTTACAAAAAAATCGTGCGGAGACAGGATTTGAACCCGTGACCTCAAGGTTATGAGCCTTGCGAGCTACCAAGCTGCTCTACTCCGCGCTGAAGAGAAGAATTGGGAACTAATGACTAATGTAATGGACAAACAAGAATGTACCCCCTACCATCTCTGTACAAATAGTATAACCCATTTATACAGAATGGTCAAGGGGCCCTCTATGATTTATGATCATAGAAATGAAAGGATATTTTAATCCTTACCAACTTGATCTTGTTGCCCCTGGCAACAAGCATGTATGAACCATTTCACGAAGTATGTGTCCGGATAATCCAAAGTCTCGATAATTAGCTCTCGGTCTTCCGGTCAAAAAACAACGTCGATGAAGACGTGTAGGTGCACTATTACGCGGTGGAGATTGTAACTTTCCATGAATTTCCCATTTCTCATTCAAGGACGTAACTTTGCTTATTTCTTTCTTTAAGGATCGACGAATCAAATGATATTTCTGTTCCAATTTTTGCCTCTTCTTCTCCCTCTGAATCAAACTTTTACTTGCCATAAAGGTTCAGTTCCTATTAGTATCAATGATACAAGTCGGATCCTAGATGTAAAAATATAAAAAAGGGGACACCCTTCTACATCGAAAGAAATGAGATTATCGCGGATACAACACATTCAAAAAATTAACCAAATTTTCCTGATGTAGAGGCAATCAAGAAAGCTGCATAAGTGAATATATAACCTACGGAGAAGTGGGCTAATCCAACCAATCTTGCTTGCACAATGGAAAGAGCTACAGGTTTATCTCTCCATCGAATCAAATTGGCCAAAGGTGTGCGTTCATGAGCCCATGCTAAAGTTTCGATCAATTCCTGCCAATACCCACGCCAGGAAATTAAGAACATAAATCCCGTAGCCCAAACAAGATGTCCAAATAAGAACATCCACGCCCAAACCGATAAACTATTCATACCAAAAGGGTTATATCCATTGATAAGTTGTGAAGAGTTTAACCATAGATAATCTCTTAACCATCCCATCAAATAGGTGGAAGATTCATTAAATTGTGAAATATTACCCTGCCATAATGTGATGTGTTTCCAATGCCAATAAAAGGTAACCCATCCAATGGTATTTAACATCCAGAAAACTGCCAAGTAAAATGCGTCCCAAGCCGAAATATCACAAGTACCGCCTCGTCCCGGACCATCGCAAGGAAAACTATAACCGAAATCCTTTTTATCTGGCATTAACTTGGAACCACGTGCATCTAAAGCACCTTTTACTAAGATCAATGTAGTTGTATGTAAACCTAGAGCAATAGCATGATGAACCAAAAAGTCTCCAGGACCTATTGTTAAGAATAGTGAATTACTATTCTCATTAACAGCATTTAACCAGCCGGGCAACCAGATACTTCGACCCGCATTAAATGCCGGGCCGCTTGTTGAAGATAAAAGTACATCGAAGCCATATGAAGTTTTACCATGAGCAGATTGTATCCATTGAGCAAATATGGGTTCGATCAAGATTTGTTTTTCCGGAGTACCAAAAGCAAGCATGACATCATTATGAACATAAAGACCCAAAGTATGGAACCCCAAAAAGAGGCTGGCCCAGCTTAAATGGGATATGATAGCTTCCTTATGGTCTAACATTCTTGCCAATACATTATCCTCATTCTGCTCCGGATTGTAATCTCGAATGAAAAAGATAGCTCCATGCGCAAAAGCTCCTGTCATGATGAATCCTGCGATGTATTGGTGATGTGTATATAATGCAGCTTGAGTAGTAAAGTCTTGGGCTATGAAGGCATAAGCAGGTAAAGAGTACATGTGTTGAGCGACCAAGGAGGTAATAACCCCTAAAGAGGCTAGAGCAAGCCCTAATTGAAAATGAATCGAATTATTGATTGTGTCATAAAGACCCTTATGTCCACGTCCCAATCGACCTCCCGGAGGAATATGTGCTTCTAAAAGATCTTTCATACTGTGCCCAATCCCGAAGTTAGTTCTATACATATGACCAGCAACGAGAAAAATAAATGCAATAGCTAAATGATGATGAGCAATATCGGTTAGCCATAAACTTTGCGTTTGTGGATGGAATCCCCCAAGAAGTGTTAGAATGGCAGTTCCCGCTCCTTGGGAGGTACCAAATAAATGACTACTTGAATCGGGGTTTTGAGCATAAAGATTCCACTGACCTGTAAAAAGTGGGCCTAACCCTTGAGGATGCGGTAATACGTCTAAGAAATTATTCCATCGAACGTACTCCCCCCTGGATCCGGGAATAGCGACATGAACTAAATGTCCTGTCCAAGCCAAGGAACTTACTCCGAAGAGTCCTGACAAATGATGATTGAGGCGGGATTCTGCATTTTTGAACCACGAAACGCTCGGTTTCCATTTCGGTTGTAGGTGTAACCAACCCGCTATTAAAGATATGACAGAAAGAAATAATAGAAAAAGAGATCCAGTATAAAGATCTTCATTAGTGCGTAAACCGATTGTATACCACCACTGATAAACACCAGAATAAGCGATATTCACTGGGCCAGGAGCACCACCTCGAGTAAAAGCTTCCACGGCCGGTTGACCAAAATGAGGATCCCAAATTGCATGGGCAATAGGTCTTACATGTAAAGGGTCCTGTACCCATGACTCAAAATTTCCTTGCCAAGCTACATGAAACAGATTTCCGGAAGTCCACAGAAAAATTATTGCTAATTGCCCAAAATGAGAAGCAAAAATATTCTGATAAAGACGTTCTTCGGTAATATCATCATGACTCTCGAAATCATGTGCGGTAGCAATGCCAAACCAAATACGACGAGTAGTGGGGTCCTGAGCTAAGCCTTGGCTAAACCTTGGAAATCTTAATGCCATAATGCCTTTCAAATCCTCCTAGCCATTATCCTACTGCAATAATTCTTGCTAAGAAGAATGCCCATGTTGTGGCAATTCCACCCAGAAGGTAATGAGTTACTCCTACAGCACGTCCTTGTACAATGCTCAAGGCTCTAGGCTGAGTAGCAGGAGCAACTTTTAATTTATTATGAGCCCAAACGATGGATTCAATAAGTTCTTGCCAATAACCACGCCCGCTGAATAGAAACATTAAACTAAAAGCCCATACAAAATGAGCACCTAGGAAAAAAAGGCCATATGCAGATAATGAAGAACCATAAGACTGAATTACCTGAGATGCTTGTGCCCATAAGAAATCACGGAGCCAGCCATTAATAGTAATGGAACTCTGCGCAAAGTTTCCTCCCGTGATATGAGTTACCACCCCTTGATCACTTATACTACCCCAAACATCGGACTGCATTTTCCAACTGAAATGGAATATGACTACCGAAATTGCATTGTACATCCAAAATAGCCCCAAGAATACATGATCCCAAGCAGATACTTGGCATGTCCCCCCTCTTCCAGGCCCATCACAAGGAAAACGAAAACCGAGATTTGCCTTATCCGGTATCAAACGAGAGCTACGAGCAAATAGAACACCTTTCAGAAGTATCAGTACCGTCACATGAATTGTAAATGCATGAATGTGGTGGACCAAAAAATCTGCGGTTCCTAATGGAATAGGTAACAAAGCGACCTTGCCACCCACCGCTACTAAATCACCACCCCCCCAAGTCAAACTCGTGCTTGTTGTTGCACCAGGAGCCGTTGCACTAGGCGCTAAAGCATGGGTGTTTTGTATCCATTGAGCAAAGATGGGTTGTAATTGTATAGCGGTATCTGAAAACATATCTTGAGGACGTCCTAAAGCGCTCATGGTATCATTATGAATATACAAACCAAAACTGTGAAAGCCTAGAAATATACATGCCCAGTTGAGATGTGATATGATTGCATCGCGATGTCTAAGGACACGATCTAATAGATCATTGTATCGAGTAGTTGGATCATAGTCTCTTACCATAAAAATGGCTGCATGCGCAGCAGCACCAACTATGAGAAATCCACCAATCCACATGTGATGTGTAAACAATGACAGTTGAGTACCATAGTCAGTAGCTAGATATGGATAAGGGGGCATGGAATACATATGGTGAGCTACAACAATGGTTAAAGACCCTAACATAGCTAGGTTAAGAGCTAATTGAGCATGCCATGACGTTGTTAGGATCTCATATAGGCCCTTATGTCCCTGGCCTGTAAATGGACCTTTATGAGCCTCTAAAATATCTTTTAGGCCGTGACCAATACCCCAGTTGGTCCTATACATGTGACCTGCTATCAGGAAAAGAATTGCAATAGCTAAATGATGGTGTGCAATATCGCTCAGCCATAGACCCCCAGTTACTGGGTCTAATCCTCCACGAAAAGTAAGAAATTCTGCATATTTTGACCAATTCAGGGTGAAAAACGGGGTTGCTCCCTCGGCAAAACTGGGATAAAGTTGAGCCAAAAGATCCCGATTCAATATAAATTCATGAGGAAGTGGTATCTCTTTAGGATCGACTCCAGCATTTAGAAATTGGTTAATCGGCAACGATACATGTACTTGATGCCCCGCCCAAGAAAGAGACCCAAGTCCTAGTAGTCCCGCTAAATGGTGATTCAACATAGATTCCACATCTTGGAACCAAGCCAGTTTTGGAGCTGCTTTGTGATAATGGAACCAACCAGCAAAAAGCATCAACGCCGCAAAGACCAATGCACCAATTGCGGTACAATAGAGTTGTAATTCACTAGTTATTCCAGAAGCTCGCCAAATATGAAAAAACCCAGAGGTTATTTGTATTCCTCGGAAACCTCCGCCTACATCACCATTCAATATTTCTTGGCCCACTATTGGCCAAACCACTTGGGCACTAGGTCCAATGTGAGTAGGATCGCTTAGCCATGCTTCATAATTGGAAAAACGAGCACCGTGGAAATACATGCCACTCAGCCAAAGAAAGATGATGGAGAGTTGACCGAAATGGGCACTAAATATTTTTCGAGAGATCTCCTCCAAATCACTGGTATGGCTATCAAAATCGTGAGCATCAGCATGTAGATTCCAGATCCAAGTGGTAGTTTCAGGGCCCTTAGCTATTGTCCTTGAAAAATGACCTGGTCTGGCCCATTCCTCAAATGAGGTTTTTATGGGGTCCCTATCTACCAAAATTTTGACTTCTGGTTCCGGCGAACGAATAATCATTGAGTCCTCCTCTTTCCGGACACGACATACAAAGAGACCTGCCAAGCGTCAAGTAATTAATGAACCTCTGAGAGATATTTTAAATTTTTTTCTTTATCTTCTATTTCCCATCTCTCTAGTTTCTTTAGTTATTCACTAGAACAAGTATGATCGGGAAGTCGATCTAAGGCAAGTGTTCGGATCTATTATGACATAGCCGTGAGGCGCTCAACGGACCTTTTTTTATATTATAAACTCTTTCTGGGCTTTGGATTGATGTAAAAAACGATTTTTTTGTTATTCCTATCTAAACTAAATTATAAGGTCTTAGACGGAACGACTTAATGTTTTACAGAGATTCTAGTAATTCGTATTACTCTATTCCAAATCACGCGAGCAGTCATTAGTCATTACTAATGAAACATCCCAGTATCTATATTTATTTTATTTTTTTTATTATTTTAATTTAAATTTAAAAATAACTAAAAATTAAAATTCAAGTTTCAAGTTAAAGTATTATTAAATTCGTATTAATAGCTAAAATCTAAAATAAGGGGTATTAATTGCTAATAAAAGAAAGAAATCTATTCTGTACTCTATCTGTTTATTCTTTAACCATACGAAATACCCGACAAAATAGAACGATCTGAAAAGGGATATAATGAAATTCTTTGATTGGTTCTTCCCGTAGGAATGATCCTATTTTATTTGACTCATAGGTACAACATTAATTCTAACAAATATAATATCAAAAAGTCGAAATTTTTTATTCGAAACGCCTCGTGATCTTCAACCAATTATGTGCTTCAATATAATTACCAGGAGTAAGCGCTATAGCTTGTTTCCAATACTCAGCGGCTTGATCGAACCAAGCCTCCGCAATTTCAGAATCTCCCTGTCGAATGGCCTGCTCTCCCCGGTCGGAATAGAGTGTTCCTTCCCTTAGAACCGTACTTGAGAGTTTCCTAACTCATACGGCTCAGCAGTCAATTCTTTTGGTATCCGTTTTACCTATCAAACGGAATGAGATTTCTCGTAGATCTATCTCGCTTTTCGGTTTCGGGTTAACCAAAAGAGGTTAATCGCATGAGTTTCAAACTTTAATTTTGATTTCTAATTAGTTTTTGTTTTATCTTTTATCCCACCTTCAGACGAATAAAGGATGGGCATTTCCTCTTTTCGTTAACATTTTCTGCAAGGTAACTATCTCGGTTTCATATCAAAATTTATATAGAATCCTTGAAAAAGGCTTTCTTTCCTGCATAAGAAAGAAAAGCTTACTATCTTTGGGATCTGATCCTACACCGCTGCTCAATACCTTAGTGGATCGCCTCTATTACATAAGCAGATTACTAACTTTTCTCTATCTTAGATTATGGCATAAGTAAGCAGTTCTTAATGTATTGGCCCAAACCTCGTTAATTGATCTTTACGGTGCTTCTTCTCTATCAATTAGATTTTTTTATCCATAGAATAAAGTATCTAGGCATATCTTATTTCTTCATATTTTCGACTCATATGAAGTTTCGTTCCTTGCTACAGCTCATAAAAATCGTTGTTTTTGACGATGCATATGTAGAGAGCCTATTTTCTTTTTTTTTTCGTATTTACTAGAACATTTTTTTGGTCTTTCTTTCCTTTTATCTTTCTATAGTGGAGATAGTCGCACGTAATGACAGATCACGGCCATATTATTAAACGCTTGTGGTAAGAATGGGTTTCGTTCTAGTGCCCTAAAATAATATTCTAAAGCTTTCGTATGATCCCCATTACTTGTGTGAATAAGGCCTATGTTATAGAGTATATAACTTCGATCGTAGGGATCAATTTCTAGTCGCATAGCTTCATAATAATTCTGTAAAGCTTCTGCATAATTTCCTTCGGATTGGGCTGACATCCGTTACGGTCGTCATTCGATTCAAAGAATCTCCGTTCCAGAACCGTACGTGAGATTTTCATCTCATACGGCTCCTCCCTTATATGCATAATGATAATATTTCAATCGTTTTTGATTCCATGTATCGATTATTCTCATTATGAATTGAGCGGGGCTAGTGTTTTTGCACGAAATTTCTAGCCAACCTTCCTGCGCGAGAGCTTTTGTTAACATCAAACGTGTTGGTACTAGATAGAAATGGTAACTCCAACAATTTCTTTGTCCTCAACGCCCCCTAATTTCCAGGAATTAGTCACTTCAACAGTCTTTGATGGTTATATGGGTATCCAAGGTACGAACGAGATGGATGTTTGTTGTCCCAACCATTCTTTTAAGTCCCAATCCAGATAAGGAAAGGGGGTAAGTAATTTTTAACAAAGTTTTCGTCTTGTTGATTTCTAGGTGTAGTGCTTTTCCCCTATGCTGCCTATTAGCACTAGTAGAGTGGGATTGACCTGTAATACAGAACCGATAGGTGTAACCTTTCGCTCAATACTAAAATGGATAATGGAAGCATATGAGGCTGCATTAATCGGGGATACACGACAGAAGGAATTGCTCTATTTCTAAACTTCACCTTCAACAAGCGTAGATTTATTTCAATAATCTATCAAAATAATAATATTTTTTCGTTCTATCCCGAATTTTTTGTCTTTCTCATAAGACTGGGAAAAAGAATCAAATCACACCATCTCTGTAATAGGTAAATGCCTCCCTTTCGCCTGAAGTTGTTGGAATTATTCGTAATAAAATATTGGCCACAACTGAAAAGGTCTTATCAATAAAATTTCCATTTATCCGTGATCTAGACATAGGTACCAATCCATTCTAAAATTCTTTTCATTCCCCCTCGTGGGAAAATGATCCTACAAACAAAGGAATTGTACAATACGAAATAGCATAAAAAAGATTCATTACAAAAGAAAAGAAATTAAAATATCAATATTAAATAAAATGTAAAGATACGAACCCTCTACTACTACTCATATTCAAAGACTCAAATTGCTCCTTTTTGCAGGAATCAATAATAAATATTTGAATACGATTCGAATTCATCCAATCCAAATGTAGTATACCAATGGGCGAACTAGTCCTATTTCATCGAAGCTGAAGAATCAAGGAATTTTTACTATAGATTCGGGCGAAAGACTTTGATTGAATTATCATCCAAAGAGGGAGGGAAAAAGAATTGAATAATTATTCTATGATGTAAATAGAATAACCGTCTATTTTGTTTGTGTTATGTGCATAGTGAGTAGACACTATACAATCAAATAGCCCCAGGATGAGTCATGAATTTGTAAGAGATCTATGAAATAATCGATTTTTTTGGTGAAAACTTTAAAACTATAGAACTATAGAAAGGAATTTTCTAATTTTTATGGAATTGTCGTTTAAATGTAATCATGATCGAAAGGTATCCATTAATCATAGTCTAAAAAACATAAACCCATCAATCCGTTGATTCCTTCCAATTCATTGATTTAATCCCGTATAAATATCATATCAGAAAAGGGCGGGAACATCATCTTCGCAAATATGAAAAATATATCTTTTTACTTTAACCTTTCACAAGCACAAGAAAAAACTTTTTTATTTTAATCTCCAAGCCTTGAATTGAAGTAAAGATCTTTCTCAAAAATTTGATATTTTTTTAGTTAGAATTGGTTGGTTGTACCTTACCTAGCCAATCCAAACAAAAAGAAAATATGAATAACTCGCTATTCATTCGGTTCCTGGGTCATAATCGTTGTGTAGGAGAGGTGGCCGAGTGGTTCAAGGCGTAGCATTGGAACTGCTATGTAGACTTTTGTTTACCGAGGGTTCGAATCCCTCTCTTTCCGTACCTTCACCCAACTCACCAACATCGCTGACCGTAACAAATCAACCAAGAGGTAGATCCTTCTTTCTATCTTTATATATATCTTATTTATATATTTATATAGATATATATTTTCTAGATATATATTTTCTATTTCTAATTTAATTGCTGCGATATGTAAAATAATGGAATAAATGGAATAAGGTCGACAAGGAATAAAAATATCTCTGTCGATCTATGATACATGAATGGGAAAACCCGGATCAAACCCTTTACTTTTAATTTAAGTCAATTTACTTTGGCGAAAGGGGGCTTATTTTTCCGAAACCTTTTCTAAACCTTTTTCTTTAAGTGTAGGCTTAAGTCTGACGGGAATAATATTCTACGACTAGCAATTCATTTATTTTCAAACCGACCCACTTATTATCTATTATTTGATTGACTACTCCTTTATATGGGAATGGGTGAAGAGTCAAATGTTTTGGCAATTCCTCACTGTGGGATGAATCCAGATAATTTTGAACGAGAGCTTTGGATTTTTGCTTATCCCTCGCCATAATAATATCTTTTGGTTTGCAACGATAACTTGGTATATCTACTATACGACCATTAACTAAAATATGTCTATGGTTAACTAATTGGCGGGCTCCAGGAATAGTCGGAGCCATACCCAATCGAAAAAGGATGTTATCCAAACGCATTTCAAGTAATTGGAGTAAAACCTGACCTGTTGACCCTTTGGCTTTTCTAGCGATACGAAAGTATTTAAGTAATTGTCGTTCTGTAATACCATAATGAAAACGTAATTTTTGTTTTTCTTCTAGACGAATACGATATTGAGATCTTTTCCCGGAACGTGATTGGTTTCTAAGATCTCTAGGCTTTTTATTAGTTAGTCCTGGTAAAACCCCCAGACGTCGTATTTTTTTGAAACGAGGCCCTCGGTAACGTGACATAAAGACTCCTTATTTATTGTTATTGATATTTCATTTTATTTAAAGAAATTAAAACTGAACTAAATGATAAAATGATAAAAGAAGCGAAATCCCCTGAAGTATTCTATTAATTGTACTAGAACGAATAATGGGACTAGAAGGAATAGTGAGATGAAAGATGTACGTATCCGAAGTTCCTCCTTGTTTTTGTATTAATATGAATTATTCATTATTATTTTATTTGAAATGGAATTTCATTTATTATTAATTTATATTAATTTATTTTTATTATATTATTTTTATTAAATTTTTTATTAATATTAAATATATTAATTTAATTAAATATATAAATATAATTCTTACCTTATAATATATAATTCTTATCTTATAATTATCTTATAATTATAATATAATAATAAATAAATATCTTATAATATAATTATCTTATAATTTATAATATAATAATAAATAAATAATAAATAAAAATCGAAAACGAAAAACTAAAATAAAAAACATAGAAAATAAAAATAAGAAAAAAGATCCTTTCCTGAGTTGAGTTGTTCTGCCGAGATTTCACTTTTTCATTGACCTTTTATTCGTAGTTGGAAGTTTCTATGACATAAGAAATCGTCGACCTTTTGAAAAAGGAAAGGTGTCTTTATTCTTTGATTTCAAAGAAACATTCTCAATCATAAAAAAATAGAACAAATAGAGAAAAGCCGGCTATCGGAGTCGAACCGATGACCATCGCATTACAAATGCGATGCTCTAACCTCTGAGCTAAGCGGGCTCACATAAGATAAACTTTACATGCATAATAATTACATAAACTATATCTTAGCTATTAACTATTCATAAATCATAAAAAATATAGAATATAAATTTATTTCAAATCCATATTGCATTATTTATAGTATATAAATAAGATAAAGATTACTATACCGATCTATAATTTATATTTATTACAATTCGAATTATAACAATTAGAATAATACATTTATCCTTTTTTATCAATCAAAAAAATTTGAATTTATAATTCGATTATAATTTTAGTTTTAGAATTCGATTTAGATAGAATTTTAGATCGAATTATATTAGATATTCGACTTTTAGATTTAGTATTCGTTTTTAAGTTTTTATAATATTTTTAATTTTAATATATACATTTCTTTATAAATCTTTATAAAAAATTTTAATATATACATTTCTTTATAAATCTTTATAAAAAATTTAATATATTCTTATAATTTATTCTATTCAATTCAAATTCAATTATGAGCTCTAGCTATAACAATATATATTAAATAAAAAAAATTAAAATATCTTATTCTTATTAAGTTAAGATTAAGGTAAGGAATTTGTATTTGATTTATCTTTTTCTATATTTTAGATTTTAGTTATATTATAGAGGTCTACCCTAAGAAAAGAATAAAAAAAAATGGAATATGCCTATAAGATATAGAAAAAGATAAATCAAATATATAAAAGAGAAAGATGCAATTCAGATCAGAATAAGACATTCCCATGCTTTGATTTGGAGACTAAGACAAAAAAAAGAATCGACCCTTTGAGTATTCCAACTTTCATGGGAAAATGAAAAGAAAGGTTCATATATCTAGTGATAGATATCCGTCTATATTGAATTGAAGATAAAAAAACGATAGAATTATTTCTGATTGGCCCATATCAAATACGGGTTCCCACTAGAGATGAAAGAAAATAGGCAAAATCAAATAGGATGAAATGCCTTTCGATATATGAATTTATATAGAATAATGAATTCAAAGGTTCCGGCATAAATGAAAGGATAAAAAAGGGAAAGTAGATCACACTGAGATCTTAAGCATAAAAGGGGGGATATGGCGAAATTGGTAGACGCTACGGACTTAATTGGTTTGAGCCTTAGTATGGAAACCTACTAAGTGAGAACTTTCAAATTCAGAGAAACCCTGGAATTAATAAAAATGGGCAATCCTGAGCCAACTCCTGCTTTCCAAAAGGGAGAATAAATAAAGGATAGGTGCAGAGACTCAATGGAAGCTGTTCTAACAAATGGAGTTGACTGTCTTACGTTGTTATAAGAATTCTTCCATCGAAACTCCAAAAAGGATGAAGAATAAACCTATATGCATACGTACTTAAATTTAAATACATATTAAATAAAAATAAAAAAATAATAATTAATGACGACCCGAATCTGTATTTTATGAATATGAAAAAATGGAAGAATTGTCGTGAATCGATTCCAAGTTGAAGAAAGAATCGAATATTCGTTTATTAAATTAAAGCATTTACTCCACAGTCTGATAGATCTTTTGAAGAACCGATCAATCGGATGAGAATGAAGATAGAGTCCCATTCTACGTGTCAATACCGACAACAATGAAATTTATAGTAAGAGGAAAATCCGTCGACTTTATAAATCGTGAGGGTTCAAGTCCCTCTATCCCCAAAAAAGCTCGTTTGATTCCCTAACTAGTTATCCTCTTTTTTTGTTAACGGTTCAAATCAAAATTGGGTCTCTTCCTCATTTACTCTTCTTTCACAAAGGTACCGAGCGGAAAAGTTTTTCTCTTATCACAAGTTAAATGATACATGAACAGCTTTGACCAAGGAGTACTCTACTCATTTGAATGATTCCCAATACATATCATTACTCGTACTAAGACTTACATACAAAGTCTTCTTTTGAAGATCCACGAAATTCCGGGGCCTAGATAAGACTTTGTAATACCCTTTCGCCTTTTTAATTGACATAGACCCCAGTTTTCTAGTAAAATGAGTAGATGATGCATAGGGAATGGTCGGGATAGCTCAGTTGGTAGAGCAGAGGACTGAAAATCCTCGTGTCACCAGTTCAAATCTGGTTCCTGGCACATGATTCATTTGGATGAGTATCTATTTTACAAATTAATTGATATGGATCGATATTCATTAATCGTATAGATCATGCACATACGTAACTTCTCTAGGTGTCTAGAGATATACCCCACCTATAAAATAGATGGGTAAAGAGTATATAAAAAAGATGTAAATGTAAAAGAGTTATGTTTTATTTTCTTTCGTTTTTTATTTGTTATTTATACTGTGTCTCCTCTCATTGAAAAAGAATATTACTCCTTCGTACGGATTCGAAAAGGTTTAATTTAATTAGTTAAGTTAGTTGCAAGACGAAAAAGTCTAGGGGAGTTAAAGTAAAGGATGAGAATAGACAAGATGTATCTCAGATACAGTACAAATAGAATCCGACCTCCTCTCATTTCTTTTTTTCTATTTCTTCATTTCCCCCTACATTACGTGACTTTCTACAGACCATCTAAGTGATGTTCGATGTTCGCGGTACAAAGTTCATGATACAAAATTTTTTTGGTTCATTCTATTGGCTCGGCTCATCCAAAATAAAAGTATCTTCCCAACTTTCGAATCTTACTGAATTCCTAATTCGATTTTCTTTCTTTTTTTACCCAACCATAATCCGAATAAAATGTCAATTACTCCGATTGTTTGATCTAGAACAGAGTGTACAAATATTCCTTAATATATATATATTTGTATTTGATTTGGAATTGTTGAAGTGAAGATGAGTTTCTTATCATTCAATAAGCATCTTGTATTTCATAAAAATTGGGGGCAATATAATCTTTACGTAAAGGCCATCCTATCCAACTTTCGGGCATTAAAATACGTTTCAGGCGTGGATGATTATCATAAAAGATTCCCAACATATCATAGGATTCCCGTTCTTGAAAATCCACACTTTTCCAAACCCAGAAAACAGACGGAATTCGAGGGTTCTTCCTTGGGGCAAATACTTTTATGCATACCTCTTCTGGTTGATCCACACCCGATTCTATTCTCGTAAGATGATACACACTAGCTAACAGCCCGCCTGGTTCGACATCATAGGCACATTGGGAACGTAGATAATTGTAACCATATACATATAAAATGACAGCAATGGAATGCCAATCTTCGGGCTTTATTTGTAAAGTCTCTATTCCTTGGTAATCGAAGCCCAAAGATCTATGAACTAGCCCATGTTTGACTAGCCAAGCAGACAAACGACCCTGCATCTTTTTTATTTCTCCCACATTTTGATTTGTATAAATATTTATTTGTATAAGTATTTCCTGAAATTTGTAAACGCGGGACCCCCATTATTCTGTACAAAAAAAAAGAATCCTGCTTAATTCACTAATTCGTGGGAAGATACTGAACTTTTGTATTTGAAAAATGTTTCAGGAGGGATCTCGGAAATAGATGGAGATTTAGAAAGTAATCCTTGATTATAATTTCCAGTATGAATACGGCGTCCAACACGAAACTTGTGTTTGATAGTAAAACATCGATTCTTCCGTTGAGACTGAATTCTATCTTCATAGATTTCCCAAGATATTTTCTTACGAAGCTTTGTTATAGCATCTATAACTGCTTCCGGTTTAGGTGGACAGCCTGGCAAATAGACATCCACAGGAATTAGTTTATCGACCCCCCGAACAGTACTATAAGAATCGGTACTGAACATCCCCCCTGTAATTGTACAGGCTCCCATAGCAATGACATATTTTGGTTCAGGCATTTGCTCATATAATCTCACTAAAGAAGGGGCCATTTTCATTGTTACTGTACCAGCTGTTAAAATTAGGTCTGCTTGTCTAGGGCTCGATCTTGGTACCAGTCCATAACGATCAAAGTCGAATCGCGAGCCTATTAATGAAGCAAATTCAATGAAGCAACAACTGGTACCATAGAGAAGGGGCCATAAACTAGAGAGTCTTGACCAATTTGAAAGATCATTTGATGTAGTTGAAATAACTGAATTTGGGGTTATTCGATCAAGTAGGGGAAACTCAACGGAATTCATAACTCTTTCAATCTTATTGTTTTTTCTTTTTTTATTTTGATTGTTAGAATATTCAGAAACTAAGACCATTCCAATGCTCCTTTTCGCCATGCATAAACTAAACCAACAATTAGGATAAGCACGAAAATGAAAGCTTCTATAAATACAGATACACCCAATACATCGAAACTCATTGCCCATGGATAAAGAAAAACCGTTTCAACATCAAAAACAACAAAAACTAAAGCAAACATATAATAACGAATTCGAAATTGTAACCAAGCATCACCAATTGGTTCTATACCTGATTCATAACTAGAGAGTTTCTCTGGTTCTTTTCTAATCGGAGCTAAAATTCCGGAAAGTAGAAATGCCAAAATCGGAATAACACTTGATATTATTAGAAATGCCCAAAAAATATCATATTCATAAAGCAAAAACATAGACGCACTCCTATGAATGTGGAAAATATAACGAATTAGGCGATTCGACTTGGAATTGTCAAGTCATCCATATCCATAACTGTTTAGTAAAAACAACAATTCATTTTGATCGAACCACCTAGTTTCGTTTGTTTACCGCGGGACATGTATCCTTTCAAGATTCATTGACTGGAATCTTATTTCCGTTTTTCATTACACCTAACTTACTTATCTTATATATTTATTAACTTACTTATCTTATATATAACTTATCTTATATATATTTAAAATCAAATAAATCAAATACAAATAAAATATAAGTAAAATAAAAATAGATATTGCTCTTATCTTATATTATACATATACAAAATACATATATACAAAATACATATACAAATAAAACGTTCTCGCTTTCACCTCATTTCCTATTTTCTCTAAAGAAAAGGAATGTAAAATCGAATTCTCATTTTTTTTTTCGCATTTCTTTCTAATTTTTTTTATTAAAAAAAAATTAAATTCGAAATTGAAAATCTTAATTCTTAAAATAAGAAAAATAAGAAAGATAATAATATCTTAAAAAAAATATTAATAATAAAATTATAATTCTATTTATATTTTTTATTTAGATTTCTTTTATGAATAGATTCTCTTATGCATAAATAGAATCAAGAAAACTGTCTTATTTTTTTCCTCTTTTTTTCGTAGTGATTTAGAATAAAGCAGTCAGACCTAAGAAAATTTCTATTTCAGGATTTCATTTAAAATTATAATAATTAAGGGGTCTTGGTATATTTTATTAGTTATTAGAATCCGGGCGAAGTAATTTATATTGATTGGATATGGAAAGAAAAAACGACTTGTTTTGTTCGTTGCTAGGTAAGGTATACGACGAGAAAAAGTCTATTTCACAACGTTGACAACGTTTCCAATGAAACTTACTAAAGATCTTTTTCAAGCCCTCGCTTGTCCACAAATCAAATAAAACAAATCAAATTAACAAATTAAGTAGTATGTTCCGAGATCAATATGATTTACTATTCGATTGGAGTGAAGTTGAGTTAGGTTGCTCGCTCAGATCATGATTTTTACTCCAAAAATTCACTAGGATTACATATAAAAAATAAAACTAGGATTATCATAATCGATTGGGGATAGTAAAGAGAAAAATTCACATGACATTCGCCTACGAAGATTAATGGAGAAAAACGGGTTTGTTTATCCGAAATTCTAACAATGTCGATTGGATCCGTTGAAATGCACTTTCGTTTTCCGTTTTATGCTTCGCTCTGAATGATCCCTGGGAGCAAGCTTCTGGGAAGAATTTTTTTTCGATGAACCGCCCGGCCATAAAAACAAACACTACTGAGGAAATGAAAATTTTAATATAGAAAATTATAGGGCTATACGGACTCGAACCGTAGACCTTCTCGGTAAAACAGCTCAAACTTGATTATTATTATCAAACTGATTCGAACTGTTTCAAAGACCCAACATGCATTTTTTTTTGCATTGGGCTCTTTCATTAACTGATATAAATATAAGTTAGTCCACCATATTTTTTCTTGACAGAAAGCAGATGGTTCCAGGTGCTCGGATTCGTTATTTGGACTCTGATCCAGGAGCACTACCAAAGTGTTTCAAAGAAGGGTTATCTTGACGTAGGTCTGCCTTTGGCCTAGATCTATTTTAAAATGAAATGGAGTCTCTATCGCTCTGCTTAAAGAATCAAATATGAAACTTCATACACCTTAAAGTTCATAGGACGAAAAGAGATTTTTGAGGTCCTTATACTTCAGTATGCCTAGCATTGAATAGGCTGGTTATTCACCCTATCACTATCTCAAATCAATAATGCGTTCTATTTGGCGACTAAATGGACACCCGAAATGGACTGAACTAATTGTCAGGCTATTGTTCTCTTGTTTCCGCAAAGTCATAGAGTAAGACATCGATTTATCAATAAGATCAATTCTTTTGATTGCATGATGAACTCCCCTGAAAAACATTGGCGCACGTGTAAACGAGGTGCTCTACCAACTGAGCTATAGCCCTTGTGTTTGTGCTACGCTACATATTTTAGCACGTAGAAAATTTCTTGTCAAGATGAGGATTCCATGATCCAACATCGTAGCTCTTTGATCCGTTTGATTCATATCATATTGCTTAGAAGTCATATTACATTTATAATCTATGTGATGGGGTTTCTATTTGTTTCCCTTTGTGATGATAAATGACCTACTTAACTCAGCGGTTAGAGTATTGCTTTCATACGGCAGGAGTCATTGGTTCAAATCCAATAGTAGGTAGAGCTTATTAGATACCAGAGTCGATGGTATCTAATAAGTTTTTCTACTCACCCCTATACCTTATCTTATAATCTTATAAAATTATAAAAAAATCTTATTCTTATATTAAAATTCTTATCTTATAACTTATATATTATAATATTATAATATAAGTATATATATAATTATATATAATAATTAATATAATAATAATTAATATATAATATAAAAATATATAATATAAATTATAAAAAAAAAAAAGAATGAATATGAATTTGTATCTTTTCTATTTTATTATTTATTTTATTTCAACCTCATTTTTTTTCCTTGCATCAGAATTTGATTCTGCTTGATTGTAGTTTTATTCAATCAGCAAAAAAAGATCAAATACCAAATAAAAAAAAATAGGATTCTATTTCTAAACAGAACTTCCTTTTTATTATTATTATTATGCTTATGTTATGCGGACATACCAACTCGTTACGAAATCATATTGATAGCCTCTACTCGTGTCCTAGCTCGTCTGAGAGCTAGATTTGCCTCAATTGTTTGTCTCTTTCCTTCCGCTTTCCTCAAATTAGCTTCTGCTATTTCAAGAGTTTGCTGAGCTTCTTGTGGATCAATGTCACTACCCTTCTCAGCATCATTTACTAAAACTGTGATCTCATTATTGCCTATTCGAGCAAAACCGCCCATCAGAGCCATCGTTAACCATTGGTCGTTAAGTCGTATTCTCAATATACCTATATCTACAGCAGTGGCAATAGGAGCGTGGTTTGGTAATACGCCGATTTGTCCACTATTAGTAGATAAAACGATTTCTTTTACTTCGGAATCCCAAACAATTCGATTAGGAGTCAGTACACAAAGATTTAAGGTCATTTCTTCAATTTGTTCTCCATTTCTAAGTTCATAGCTTTCGCGGTAGCTTCATCGATATTACCTACCAAATAAAAGGCCTGCTCGGGAAGCCCGTCTAATTCTCCGGAAAGGATCAATTGAAACCCTCTAATTGTTTCTGCTAGACCAACATATTTTCCTGGCGAACCTGTAAATACTTCGGCTACGAAAAAAGGTTGTGATAAGAAACGCTCAATTTTTCGTGCTCTTGCTACGGTTAAACGATCTTCTTCGGATAATTCGTCTAACCCAAGGATAGCTATAATGTCCTGAAGTTCTTTGTAACGTTGTAAAGTTTGCTTAACTCTTTGCGCAGTTTTATAATGTTCCTCGCCAACGATTCGAGGTTGGAGCATAGTTGACGTTGAATCTAAAGGATCTACTGCCGGATAGATACCTTTGGCGGCTAATGCTCTTGATAGTACAGTAGTTGCATCTAAATGTGCAAATGTCGTGGCAGGAGCGGGGTCAGTCAAATCGTCTGCAGGTACATAAACTGCTTGAATCGAAGTTATGGACCCTTGTTTGGTAGAAGTAATTCTTTCTTGTAAAGAGCCCATTTCGGTACTAAGAGTAGGTTGATAACCCACTGCGGAAGGCATTCTACCCAATAAGGCAGATACTTCAGATCCTGCTTGGACGAAACGGAAGATATTGTCAATAAATAGAAGTACGTCTTGCTTATTAACATCTCGGAAATATTCCGCCATAGTTAGGGCAGTCAAACCAACTCTCATACGAGCTCCGGGCGGTTCATTCATTTGCCCGTAGACTAGAGCTACTTTTGATTCTGCAATATTTTGTTCATTAATAACTCCGGATTCTTTCATTTCCATGTAAAGATCATTTCCTTCACGAGTACGTTCACCTACGCCGCCAAATACAGATACACCTCCATGAGCTTTTGCAATATTGTTGATCAATTCCATAATGAGTACGGTTTTACCCACTCCAGCTCCCCCAAATAGTCCTATTTTTCCTCCACGGCGATAGGGGGCTAAAAGGTCTACCACTTTAATTCCTGTTTCAAAAATAGATAATTTTGTATCTAACTGTATAAAGGCAGGTGCGGATCTATGAATAGGAGATGTTGTGCGAGTATCTACAGGACCTAAATTATCAACGGGCTCCCCAAGTACATTAAAAATTCGTCCGAGAGTTGCTCCGCCGACTGGAACACTTAAAGGAGCCCCTGTATCAATCACTTCCATTCCTCGCGTTAGGCCATCTGTAGCACTCATAGCTACAGCCCTAACTCGATTATTTCCTAATAATTGTTGTACCTCACAGGTTACATTAATTGGTTGACCAGCAGTATCTCGGCCCTTAACGACCAGAGCGTTGTAAATATTAGGCATCTTGCCTGGTGGAAAAGCTACATCCAGTACTGGACCAATGATTTGAGCGACACGCCCCAGGTTATTTTTTTCAAGTGTGGAAACCCCAGGACTAGAAGTAGTAGGATTGATTCTCATAATAATAAAGTAATAAAGTCAAATAAAAATATATCAAAAAATAAAAAAAAAGAAAAGAAATATAAATATATAATAAATATATATATATTTCAATTTCATTTCGTTTCGAATTTTTTGCGAAAATGAAAATAAAATGTCCGATAACAAGTTGATCGGTTAATTCACTAAGAAATGGGAGTTAGCACTCGATTTTGTTGGGACCATCCAACCGAATCCAATTCAATTGTTTACTTATTCCTTCCTTTATTTTCAATTTCTTTGATTCAATTTCAATGAGTGAGTTCAACCAATCTATTTTCAAAATATCAAGTCAATGAACAAAAATTTGTAGAAAGTCTTTTATTTGTCTATCATTATAGACAATCCCATATATATTATCGACGGAATTCGAACCTGAACTCTAAACTCGATTTTCTTTTTGGTTCATTATTTTATTTCTATCGCATTGGCTATCGCATCGGCCCTTTTTTTTTATTTAGCATATTGATTTACGTCTAGCCTATTCTTTTTTTTTCTTATTTATACCCTTTCGTCTATGAATTCCGCATATTTTCACATCTAGGATTTACATATACAACATATATTGCTGTCAAGAGTGAATTTTTATTATTATTTAGTCAATTCAAAAAAGGTTAAGGCATTAGAAACTTGAAAAAGAAGGGTTGGGTTGCGCCACATATAGGAAAGAGTATACAATAATGATGTATTTGGCGAATCAAATACTATGGTCTAATAACGAACCATTTTAATTAGTTGATAATTTTATGAAAGATTCCTGTAAAAGGGTTAATTAACGCCTAATTCATGTTAATTCATGTCGAGTAGACCTTGTTGTTGTGAGAATTAGTAATTGATGAATTGTAGGGAGGGACTTATGTCACCACAAACAGAGACTAAAGCAAGTGTTGGATTCAAAGCTGGTGTTAAAGAATACAAATTGACTTATTATACTCCTGAATATGAACCCCATCCGCATGATATCTTGGCAGCATTTCGAGTAACTCCTCAACCTGGAGTTCCACCCGAAGAAGCAGGGGCCGCGGTAGCTGCCGAATCTTCTACTGGTACATGGACAACTGTGTGGACCGATGGACTTACCAGCCTTGATCGTTACAAAGGACGATGCTACGGCATCGAGCCTGTTCCTGGAGAAGAAAATCAATATATTGCTTATGTAGCTTACCCATTAGACCTTTTTGAAGAAGGTTCTGTTACTAACATGTTTACTTCCATTGTGGGTAATGTATTTGGGTTCAAAGCCCTGCGTGCTCTACGTTTGGAGGATTTGCGAATCCCTTCTGCTTATTCGAAAACTTTCCAAGGCCCGCCTCATGGTATCCAAGTTGAGAGAGATAAATTGAACAAATATGGACGTCCCCTATTGGGATGTACTATTAAGCCGAAATTGGGGTTGTCCGCTAAGAACTACGGTCGAGCAGTTTATGAATGTCTTCGCGGCGGACTTGATTTTACCAAAGATGATGAAAACGTGAACTCCCAACCATTTATGCGTTGGAGAGACCGTTTCTTATTCTGTGCCGAAGCTCTTTTTAAAGCACAGTCTGAAACAGGTGAAATCAAAGGACATTACTTGAATGCTACTGCAGGTACATCCGAAGAAATGATAAAAAGGGCTGTATTTGCCAGAGAATTGGGAGCTCCTATCGTAATGCATGACTACTTAACAGGGGGATTTACTGCAAATACTACTTTAGCTCATTATTGCAGAGATAATGGCCTACTTCTTCACATCCACCGTGCAATGCATGCAGTTATTGATAGACAGAAAAATCACGGTATGCACTTCCGTGTACTAGCTAAAGCTTTACGTCTGTCTGGTGGAGATCATATTCATGCTGGTACTGTAGTAGGTAAACTTGAAGGGGAAAGGGACATCACTTTAGGTTTTGTTGATTTACTACGTGATGACTTTGTTGAAAAAGACCGAAGTCGCGGTATTTATTTCACTCAAGATTGGGTTTCCCTACCGGGTGTTATTCCTGTGGCTTCCGGGGGTATTCACGTTTGGCATATGCCCGCTCTAACCGAGATCTTTGGGGATGATTCTGTACTACAGTTCGGTGGAGGAACTTTAGGCCACCCGTGGGGAAATGCGCCGGGTGCTGTAGCGAATCGAGTAGCTCTAGAAGCCTGTGTACAAGCTCGTAATGAGGGGCGTGATCTTGCTCGCGAAGGTAACGAGATTATTCGTAAAGCTGCCAAATGGAGTCCTGAACTGGCTGCCGCTTGTGAAGTATGGAAGGAAATCAGATTTGAATTCCAAGCAATGGATACTTTGTAATCCAGTAATTACTGGTCGGTCCCTTAAATTGAATTGCAATTAAACTCGGCCCAATCTTTTACTTTTAGTAAAAGGATTGAGCCGAATGCAACTATTGTTTTGCATAGATCTTAGATCTACAAGCAAAATCCTAAATAAAAAATCGAAGACTAAAAAACTCAAAAGTTTCTTTGGTTGTGCTGGGATCCACAATTAATCCTATGGATCTCTAGGATTGGTGTATTCTATTGGTGTATTCTTATATATATATCCCGTAGCTTAGGACCGCGGATAGCGAGTCAAGTATAAGAACCCCTTCTACCCATCCTGTATATTGTCCTTTTCTTCCGTATTGGAATAGAAACTTTAGACGAGATTTTACGAAAAAAATTTATTCATGGAAAAGAATAAATATTTTTTTTCTGTTGATGCGAATTTCACACGACATGGAAAAACCCCTACTTTTTTTATTTATATTTTTATATTCCAATTGAAAAAAAAAGTTCTATCATATAGAGTGAAGTGATACCCGGATTTTTACAAAGGATAATCTTTTATTTCTCACTCGTTTTTAGTTAATAATCCAAGTTAGGATTTGAAAATTTCAAATGACTTTTCATCGAATGACTATTCATCTATTTTTTTATTTCATGCAAATAGGGGGCAAGAAAGCTCTATGGAAAAATGGTGGTTTAATTCGATGTTGTCTAAGGAGGAGTTAGAACATAGGTGTGGGCTAAGTAAATCAATGGGCAGTCTTGATCCTATTGACAATACCAGTAGCAGTGAAAATACGAGTATAAATTATACAGAAAAAAACATTCATAGTTGGATTAATGGTTCTAGTTACAGTAATTTTGATCTTTTATTCGGTATCAGGGACATTCGGAATTTCATCTCTGATGATACTTTTTTAGTTAGGGATAGTAAGGGGGATACTTATTCCATCTATTTTGATATTGAAAATCAGATTGTTGAGATTGAAAATGATCATTCTTTTCGGAGTGAACTACAAAATTCTTTTTCTAATTATTGGAATTCTAGTTATGGGAAGGGATCTAAAAGTGATGATACCCATTATGATCTTTACATGTACGATACTAAATCTAGTTTGAATAATCACATTCATAGTTGTATTGACAGTTATCTTCATTCTCAAATGCGTATTGATAATTCTGTTTTAAGTGGTATTGATAATTCTGTTTTAAGTGATAGTGACAATTACAGTGATAGTTACATTTTTGATGAAAGTCAGACTACCACTAACAAAAAAGGTAGGGATAAGAATCTTGATGTAAATAAAAAATACAGGCATTTATGGATTCAATGCGAAAATTGTTATGAATTAAATTATAAGAAAATTTTTAAGTCAAAAATGAACATTTGTACGTACTGCGGATATCATTTGAAAATGAGTAGTTCAGAGAGAATCGAACTCTCGATTGATCCAGGTACTTGGAATCCTATGGATGAAGACATGGTCTCTCTGGATCCCATTGAATTTCATTCGGCGGAGGAACCTTATAAAGATCGTATTAATTCTTATCAAAAAGAGACAGGGTTAACCGAAGCTGTTCAAACAGGTATAGGTCAACTAAACGGTATTCCTGTAGCAATGGGGGTTATGGATTTTAAGTTTATGGGAGGTAGTATGGGATCCGTAGTAGGAGAGAAAATCACTCGTTTGATTGAGTATGCTACTAATCAAAATCTACCCCTTATTATAGTGTGTGCTTCCGGCGGGGCGCGCATGCAAGAAGGAAGTTTGAGCTTGATGCAAATGGCTAAAATTTCGTCTGCTTTATTTGATTATCAATCAAATAAAAAGTTATTCTATGTATCAATTCTTACATCTCCTACTACTGGAGGGGTGACAGCTAGTTTTGGTATGTTGGGAGATATCATTATTTCCGAACCCAAAGCCTACATTGCATTTGCGGGTAAAAGAGTAATTGAAGAAACATTGAATACGACAGTACCTGAAGGTTCACAAGAAGCTGAATATTTATTCGATAAGGGTTTATTCGATCCAATTGTACCACGTAATCCTTTAAAAAGCGTTCTAAGTGAGTTATTTCAGTTGCACGCTTTCTTTCCTTTTAATCAAAATTCGATCGAGCAGTAAGGTCAATTATTTATTTCTTTTTTATTTGTTTATTTGTGGCAAAAAAAGTAGTTAGTTATCGTAATCAATCAAAGTAAAAATGATAAAGAATCAATCATAATAGAATAACGGGGATGGGGTTTTCGTGGTTGCCATACTAATTATATTCTATACTAATTCTATAACTATAATATAAAGAATCAAAAGTTGCGGATAAATTTTTTTACTTTTTTATTTTACTTCATATTCCATTCCTGATTACTAATCAGAGAACCTCTATTCTATCAACAAACAAGATATTCTTATAAATTGAAAATTTGGCAAATAAAACGAATTTAATCTTCCTTTCCTTTCTTACATCTGGATATAAAAATTCGAATTAAAAAAAAATAGCCTTTTGCATCTTAATATATTTCCTTGTCGGAGACTCTCCATTTTTACTAACAAGAGAATATCTCTTGGATCAGATTCGTTAGAATCTTTCGGGACTTTGTAAGCAACTCTTTCTTTATTGATATTGAATTAAAAGACAAGGGCAAAAGAAGAAGAAAAGATGAAGAATCAAAAAGTTGATTATCAAACATATATTTTTTTTGTGTCGAAGGCTAATTAAGTTCATTTAGTTAGTTCTACATTTCTTGCACTTAGTATATACTCACTTAGATATAATTAGTATAATTATATAGAATTAGAATTCTAATTAAGTTAAGATAATTTTAGAACAATATAACAAACAGGTACAAATAGTAAATCGAGGTACCCATTCTATGACAGATATAAACCTTCCCTCTATTTTTGTGCCTTTGGTAGGCCTAGTATTTCCGGCAATTGCAATGGCTTCTTTATTTCTTCATGTTCAAAAAAACAAGATTGTTTAGGCCGGATGGTGAGGCCAAATCACATTTTTTCAAGACTTAGACTTGATTGAATCATAACACAGATATCTCTTTATTGGAAAGTGGAATATGGTATAATGCGTGATTTCTTTCGAACATAAATGCAAGAACTCTTATGCATGCGAAACCTGATATGATATAGGGGTAATTTAATTTTAACTATTTTCAAATCAATAGATCAGGACCGGTCGGTCCATGTTTGAAATAGAAAGTCAATGTTTGTAGATATCTAGGACGGGGTCATATGAAGGGGACGTTCTTATTTTCGATCGAACGAATTATATGAATTACCCCTACAGGTTCACATTATAATAGTGCTAGTTGATGAGAGTTACTTCAGAAACAAAATAGCGTTAAGTTAAGTATAAGTAAGGTGAAATTCATTTTGGTTATTCTATCAATTCAATCAATTAAAAAAAAATGCAACTAGATTAGTATGAATTGGCGATCAGAACGTATATGGATAGAACTGATAAAGGGGTCTCGAAAAACAAGTAATTTCTGCTGGGCCTTTATCCTTTTTTTAGGTTCATTAGGATTTTTATTAGTTGGAACTTCCAGCTATCTTGGTAGGAATTTGATATCTTTATTTCCCTCTCAACAAATAATTTTTTTTCCACAGGGGATCGTGATGTCTTTCTATGGGATCGCAGGCCTCTTTATTAGCTCCTATTTGTGGTGCACAATTTCGTGGAATGTAGGTAGTGGTTATGATCTATTCGATAAAAAAGAAGGAATAGTGTGTATTTTTCGTTGGGGATTTCCGGGAAAAAATCGTCGCATCTTCCTCCGATTCCTTATAAATGATATTCAGTCTATCAGAATAGAACTTAAAGAGGGTATTTATCCTCGTCGTGTCCTTTATCTAGAAATCAGAGGCCAGGGGGCCGTTCCTTTGACTCGTACTGATGAGAATTTGACTCCACGAGAAATGGAACAAAAAGCTGCGGAATTGGCCTATTTCTTGCGTGTACCGATTGAAGTATTTTGAAATGAACTGAAGAATGAATGCTTTCTCATTCTCGACTGGGGGTAGAAAAAACTCTACAATCCCCTTTTTGTATAACTTTCATTTTTATATGGTATAACTTAACGAAAATTTCGTCAGAACGTCCATTCGAGTCAAAGCAAACGTATATTATATGGAACATCCAAAAAAAGGGGATTGTTTTTGTCTGCAAAAAAGATATTTTATGCGTATGGAAATTCACTCGACGCAATTCATTAGCAAAAAAAGTAACAAATCGAAATAAGGATAGATTCATCCCAAAACATTTCGAAATAAAGAAATTTTTTATTTTAGTTTCAATATTTTGAATTGATAGGTTAGAGACAAATAACTCATGTAATGTAAATTAATTATCTCTCTTTTATCGATGTTTCGTTTTCTCCCTTCTTTCGCTCTCTTCTCTTTAATGAATGACCCAACATTTGGATTTCTTATAACGATAATTATCCAATTTCTGTCTTGTTTTGCTACCCCTTTTTGATCATCACATTCAGAAAATTCTCTCAATTATTTTTGTACTATGGTAGTCAGCGAATTTTTAAATATTTGGAGAGTTTTTCGTCTCGAAATCCGAATTCATTAACTAAAGCGGGTTTTCGGAGATTCATCGAAAGGAAGGATCGAATTTGACCAATTGAAATAGCTGGAAACTTTATTTTTTCTTATTTTCGCATTCGAATTCGAAGTGGACTCTTATTCGATTTCTGTATTCTTGCAAGATTCTTCAAAATTCTCAAGGACTAATTCTAATTACCGAATCACAAATAAAAAACAGAGAATGATTCGATACCTTGGAATAGAACTCATTTTGATGAAAAATAAAAAATTAGATCACATAGAGTCGACGAATGAGGCCGCTTTATTAACAATTTCTAAATGAAAAAAAAATGGCAAAAAAGAAAGCATTTATTCCCCTTCTATTTCTTGTATCTATAGTCTTTTTACCCTGGTGGAGCTTTCTAGCATTTAATAAAAGTCTGGAATCTTGGGTTACTAATTGGTGGAATACCAAGCAATCCGAAACTTTTTTGAATGATATTCAAGAAAAGAGTATTCTAGAAAAATTCATAGAATTAGAGGAGCTCTTACTGTTGGACGAGATGATAAAGGAATACCCGGAGACACATCTAAAAAAGCTTCGTATAGGAATCCATAAAGAAACGATTCAATTGATCAAGCTGCACAATGAAGATTGTATCCATACAATTTTGTCCTTCTCGACCAATATAATCTGTTTCGTTATTCTAAGTGGTTATTCTATTATAGGTAATGAAGAACTTATTATTCTTAACTCTTGGGTTCAGGAATTCCTATATAACCTAAGCGACACAATAAAAGCATTTTCTATTCTTTTATTAACCGATTTATGTATCGGATTCCATTCACCCCATGGTTGGGAACTAATGATTGGCTCTATCTACAAAGATTTTGGATTTTCTCATAACGATCAAATTATATCTGGCCTTGTTTCCACTTTTCCAGTCATTCTAGATACAATTTTGAAATATTGGATCTTCCGTTATTTAAATCGTGTATCCCCATCACTTGTAGTGATTTATCATTCAATGAATGACTGAAAAAAAGGGTCTACTGATATTAATCCAATTAGAATGTTTGGTACTTTGGGCATAAGCATTCCAAATCGTACTGACTCTTTCTACCCATCCAAGGCAGGAAGGTCCTCCTATATTCCAGTAAGATTATTCCAGTAAAGTAAATAACAGAATCGTGGATAGGGAACTATACTAGCGACCTACCCAATTTATTGTAGAAATTTTCGGGATCAATAATTGGACCATGCAAACTAGAAATACCCTTTCTTGGATAAAAGAACAGATTACTCGATCCATTTCCGTATCACTCATTATATATATAATAACTCAGTCATCCATTTCAAATGCATATCCCATTTTTGCACAGCAGGGTTATGAAAATCCCCGAGAAGCGACCGGTCGTATTGTATGTGCCAATTGTCATTTAGCTAATAAGCCCGTGGATATTGAGGTTCCACAAGCGGTGCTTCCTGATACTGTATTTGAAGCAGTTGTTCGAATTCCTTATGATATGCAACTAAAACAAGTTCTTGCTAATGGCAAGAAGGGAGGTTTGAATGTAGGAGCTGTTCTTATTTTACCCGAGGGGTTTGAGTTGGCTCCAACCGATCGTATTTCTCCCGAGATGAAAGAAAAGATAGGCAATCTTTCTTTTCAGAGCTATCGACCAAATAAAAAAAATATTCTTGTGATAGGCCCTGTTCCGGGGCAAAAATATAGTGAAATCACCTTTCCTATTCTTTCGCCGGACCCTGCTACTAAGAAAGATGTTCACTTTTTAAAATATCCCATATATGTAGGCGGTAACAGGGGAAGGGGGCAGATTTATCCCGACGGAAGCAAGAGTAACAATACTGTTTATAATGCTACAGCAGCTGGTATAGTAAAGAAAATAATACGAAAAGAAAAGGGCGGATATGAAATAACCATAGGGGATGCCTCGGATGGGCGTCAAGTCGTTGATATTATTCCTCCAGGGCCAGAACTTCTTGTTTCAGAGGGTGAATCTATCAAACTCGATCAACCATTAACGAGTAATCCTAATGTGGGTGGATTTGGTCAGGGAGATACAGAAATAGTACTTCAAGACCCATTACGCGTCCAAGGCCTTTTGTTCTTCTTGGCATCGGTTATTTTAGCACAAATTTTTTTGGTTTTTAAAAAGAAACAGTTCGAGAAAGTTCAATTGTCCGAAATGAATTTCTAGATCAAGTTCATAACAAGAACCAAATTCTTGTTTGTTTATAGTTATGTATGATTATGTATGATCACGAAATAAAAAAAGTACAAAAGCCCTCTTCTTTGTTTATACTTTTTTAACGAGATGTCGGGAATTAGTTGTACTACATTCTTAGTCATACTATGTATATTGTATATTGTACAGAAGACTATTTGACTTTTTAATTTTACTTTTTTTTTCAATACAAATTGTAATGATGTGACTATGTAACTCTTATCAGATTTAAATGTTATCGAATGCATCAATACAATAGTTTTTTCTATTCGAGAATCAAATTCGATTAGATACTAGACTAAACATAGAATAGAACACACAGATAAATGCGGGGAATAAATGAGTCTAGGAGGGATTCTTTGCCTTCCTAATCTTCGACACAAGAAAGGGATGTGTAAAATTCCCTTTCTTGTGTCGAAATAGTAATGATTCGTGATGGTCTTCGTCAAAGACGCGTAATTTTCGATTTTTTTATAGATTTTTTGGGGGGTTCAGGAGATTTAACCGAACTTTTTTTGTCTTACTATTATGCATATAATAGATAGAACAAAGTAGTGGACAAACAAAAAAAGAGAGAGAATTTTATTGAGCAACAAATAGAACTTCTTCAATGAACTTATAAAAAAAATTTCTTTGATTTGATGAATACAAAGAGGATATACTACTAGAATTTTTTCTAGTAGTATAGAAGGGTTTTCATGCTATTTGATCTACCTATTTTTTGGCTTTATTTTTATGCGATAAAATATTCTAATTATTACGAATTTAACGGGTACCTCCTCCTTCTTTGTTTCTCTAATTCGAGGGGGAAGGAGGGTCCCGTTGAGTTCTTACGCTTTCATATCTATACCGCAGTTCATCCGATTACTACAGGGATGAACCCAATCCGGAATATGAACCATAAAAGAAAATGCCTATTAAACCGATCACAAGAATGCCGGTTACAGTACCTATTATCCAAAGCGGAATCCTTCCAGTAGTATCGGCCATTTACCCCACTTCCCTCCACATTTCATCAAGTGGTCATGCTAGAGACATAAACAGTCATGGGTAATTATGAGATGATCCTTCCGAATGGGATAAGAAAATGCCTACTCTATATATTATTCTCCCTCGTTCTTTTTAATTGAAAAAATAATTTGAAAATAAAACAGCAAGTACAAAAATGAGTAATAACCCCCAGTAGAGACTGGTACGATTCAATTCAACATTTTGTTCGTTCGGGTTTGATTGTGTCGTAGCTCTATAATTCGGATTAGGTTTTGTTTATCGTTGGATGAATTGCATTGCGGATATTGACCCCAAAAAAGAAACGGTAGGTACAGCTAGTCCGTGAACAGCCAACCATCGCACTGTAAAAATGGGATAGGTTCGATCTATGGTCATTGGGCCTCCTAAAAGGATCTACTAAATTCATCGAGTTGTTCCAAAGAATCAAAACGGCCAGTTATTAATGGAATTCCTTGTCGGCTCTCTGTAAAATACTCGTTTGGCCGAGGGCTTCCAAACACATCATAAGCTAAACCTGTGCTGACGAATAACCAACCCGCAATGAATAGGGAAGGTATAGTAATGCTATGAATGACCCAGTATCGAATACTGGTAATAATATCCGCAAAAGAACGTTCTCCTGTGCTTCCAGACATGCTCAGCTCCACATATTCTTGTACAGGCAAATGTAAATCGATTCCGTAAAAGATGAGATCCGTAAATGGAAATTTACTGAAATTCTTTGTGGGATCGTCAATATTGTACCAAGGGTGTCTTTAGAGTATACCAAATCAGTATAGCCGTCCTTCTTCTGACACAGCAAGGCAATTTCAATTAATATTGAAACTAAGTATACGTATTAGACGATTTCTTTTTTCTTGCTTGTCAATATAGAACTATGCGCCATTTAATAGAAAAATCCTCCAATTAAAATAGTATAGAGATTCTCTATTGGCTTCGGACTAGAAACGAAAATCGGGTAGAAATCGGGTAAGGGATCAATCTGACAAGTCGGTTTCTAATAATTCATCAAGGAGAGTATTATATTCCCACAATTCAATTAGACGCGAAATCTAGAACTCTCTTTTTTGTGTTTATAGAAAGAAAAGCTTTTTTGGAATCCTTTTTTTCATTTTAAGGAATTGATTAGTCCTCTAGTACCAAATAAGAGTAGTAGGTAGGAGATAGTATTCGATGAAAGAAAACAAAGAATACAAGAATTTAATTGTAAGAAATTAATCAACATTCGTGATGCATGCATTCCTGTATTAGATCCAAGGGTTCTTTCTTGCTCTAACTATAGGGATCAGACTTGATAAAAAATACGAAAAGAAAAAATGTAGAACCTATAACAAAGATAATCAGAATGAATCATCTTAGAATCGATTTGGACTAAAATCCAAATTTTATTTTTTTTGCGTGATCGTGTTGATAGCCTTTTTCTTATCATTCAAAAAAAAGATTCTGTGAATAAACTCGATTGCTGAATCTAATGAGTGAAAATAAAAATAAGATCGCTTTTCGTCCTAAACTAAAAAAAAAAGAAATAAAATAAATGCTCAAACTAGAATGCTTTTCCTGTTTTCTTCGATAGTGAGAGTTTTTTTTGAACAAAGTTACATGACATAGTTCTTATTTATTTTTATTAGTTTACTCCAAGAGTTGCTCAAAAAATATGTTGATTAGAAATCACGGAATCTGTAGATGTCACAGACAATGAGTCGATTTCTTTTTCTACCTCTCTTCCTTTATCTTATCTTTTCTTAGTTATATCTATAATGTAATAAATGTAATGATTGGGGAATCAATTGAACTTATTCTTTCAATTGGTATTTTTTCTTATTTTCGCTCCTATCTTTCACAAAAAAAAGTAAACTTAGGTAAATGCTTTAGAAATATATGTATAAAAAAAACATATTTGATTTAGCTCCTTCATGCCTACTCTAACTAGTTATTTCGGTTTTCTACTAGCAGCTTTAACTATAACCTCAGCTCTATTTATTGGTCTGAGCAAGATACGACTTATTTGAAATAAATTGAATCAACAATTCATAATCATAAAAAGAAATCTTTCTGTAGGATTTCATGTATTTTTTAAGTTCTTTATAGCTCTTTATCGCGACAATTGAAAATTTTCGGTTATTGAGATTCACGGACAATTAGGATGAATAGTTAGGGATAGATATTACCTCCCTTTTTTCCTTTCAAACAAATTGAAATGATTGAAGTACTTCTATTTGGAATCGTCTTAGGTTTGATTCCTATTACTTTGGCTGGATTATTCGTAACTGCATATTTACAATACAGACGTGGTGATCAGTTGGACCTTTGATTAGTTAACAAATCTTTTTTGATTGACCTCCTTTCTTTACGCCACAGGAGGTCAATTTTAGATTCCTCTTCAAGGTAGTGAAGTTATTTCAGTCTAATTAGAACTAACAAGAATGGAATCACGCTCTGTAGGATTTGAACCTACGACATCGGGTTTTGGAGACCCACGTTCTACCGAACTGAACTAAGAGCGCTTTCTTATCACAGACAGTAAAGAGATTCCTTTTGTAACCCAATACTACATCCTGCATGCATATACTATCGCATATATCGCATATATAGTTAGAATTGTATATGTGTTATATGTATAGATATATTTATCTAATAGTATAGTTTAGATAATAGTATAGTAGTTGTATAGTAGTTATAAAAAAAAAGTCTTATCTTATAAAAATCATAAAAAAATCATAAAAAAAAAATAATATACATAAAATAATATAAATAAAGTAATATATATTATTTTATATTATAATAAATTATATATTATAATTTATTATATATATTAATACTATTCTATTAATACTATTCTAATAATGACAGATTATGTATGTCCAATTTGAATCGATTTCATCGATCTCAATTAATTCCTCTTTACTTCTCAGAGGAAAAGTAATAGGTAGGGATGACAGGATTTGAACCCGTGACATTTTGTACCCAAAACAAACGCGCTACCAAGCTGCGCTACATCCCTTTTAATAGGTTTACAGTGTTATTGTAAAGAATCCTTTTCTTTTTTTCCACATCATTATTTCTCATATTTAGATACACAATAGATCTTGCCATTTTTTCTTTTTTTTTTCATATCATATATTATATAATATAAAAGTCTTTGGCGCTGTAAAGTAAAAAAAGGCATGCTCAGTAGGAAAGATGCATCTTTTTACTTTTTTAAAATTTAACTTAAAATAAAGGTAGAAAATCTTATCTATCGGATTGTTGTACATTTTAATTTGCCTTAGGAATTTCATGTACAAATACAAGCGGTTTTTCTTTTTAAATACATCTACATATGCATCTGATCATCTATCATATATGTATTAGTCTTATGTGTTACAATATAACAATATATAAATAAAGAAATAAAAAAAAGAAGGAGGATTTTCAATGCGAGATCTAAAAACATATCTCTCCGTGGCACCGGTACTAAGTACTTTATGGTTCGGATCTTTAGCAGGTCTATTGATAGAAATCAATCGTTTTTTCCCGGATGCGTTAACATTCCCCTTTTTTTCATTCTAGTTATTGACATGGTAAGGGGTAACGAAGATTAGAGATAGAATCCACTATCTGTGACTAATCCCCCGCCCTTTCTCCCTTTGACCTTATATTCGAAAAGGGAGAAAGAAAAATGGATTCAACCTCAGCAAAGCTTGGGCTCAAGCTCGAATTTCAAATTCAATTTTAAAATAAATAAAATAAGAGGGAGAACGGAAATTTAAATGTGGGTCTAGGGCAAAAGTCTCATACACGAGACTTAAATGAAATACTGTACTGTGATTAGAAATATAGTTAGAAATCATTGGATTACGTATTCTTTATTATACTGAATTAGATTTTCTATTAATATTTCTTTGATTTTCTATTAATATTTAATATTATTCTTATTGTATTGATATTCCTCTATTTACTGCAACGAAATTTTTTTAAGTGTATTTCGAGTTAGCAATTTCGATTATTATTATAATTTTCTTTCTCTCCGCTTCGGGTCGAAAATGAAAGAGTTGCTTGAATAAAAAATTCAAACACAAAAAAAGGGGGTTCATGGCCAAGGGTAAAGATGTCCGAGTAAGCGTTATTTTGGAATGTACTAGTTGTGTCCGAAAGAATGTTAATAAGGAATCAAGGGGTATTTCCCGATATATTACTCAAAAGAATCGACACAACACACCCAGTCGATTGGAATTGAGAAAATTCTGTCCCTATTGTTACAAACATACAATTCATGGAGAGATAAAGAAATAGATCGAACCGAACGTCTGTGTATCACCTTTTGAAGGAAGAGTACAAAAGGACATCTATTATACATATATTTCATTATATGCATTTACCTTTTAAAAAAATTATAAGAAAAATGTAATAAACATACATATTATTATATGCAATAGATAATAATTATTAATATTAAAAAAAATTATATAGAATTTTAATTTTATTTTAAATACTAAATAATCTAAAATCTAAATAATTAAATAATAATAAAATAATATTAATATGATATTAATATTAGAAAATTATATTATATATATAATAATATATAAAATATAAATTATATTATATAATAATATATAAATATATAAAAACCAAACTAGAAACTATATTCTATATAATATAAGCGTATAAATAAATAGAACATACAAATAAATAGAACATATATAATATAAAATAAACAAATTCAAATTAAAGAAAAGAAAATAATAAAAAAACCAAATCCTATTTTTCATTTTTTTTAGATCCGACCGAAATAGGATTTTCGGTAGAATATTATTTATATTATAAGGAATAAACTAAACAAACCATGGATAAATCCAAGCGATCTTTCCTTAAATCTAAGCGGCCTTTTCGTAGGCGCTTGCCCCCGCTCCAATCGGGGGACCGAATTGATTATAGAAACATGAGTTTAATTAGTCGATTTATTAGTGAACAGGGAAAAATATTATCTAGGCGCGTGAATAGATTGACTCTGAAACAACAACGATTAATTACTATTGCTATAAAACAAGCTCGTATTCTATCTTTGTTACCCTTTCTTAATAATGAGAAACAATTTGAAAGAGCCAAGTTGGCCGTTAGAACTACGGGTTTTCGAGGTTTTCGAACAAAAAAACAATAGCTCTTTATTCAATTCAATTGATGTTTTGTCAATTCAATTGATGTTTTGTTCGAAAAAATCCGATAATCCGGATTTGATTGTTGTCTCGGAAGAAAAATCGAGGAAGAAGAAATCTTTTTTTTATTGAATGGCTTTGTTCATTGTTCATTTTGACTACTTTATTGTTATTGTAATTGTATTTTATATTTTATTTTATCGGACTAATTTCATTTCTATTCTATCTTCCCTTCCCGGAGTTCCTTCTCCGGGGAACTTTGTTTAAATCATTTCGGGTGCTTCTTTCCAATCTTCTTTTTTTATGATCTCATTGGAAATACTATAAAGACAATTCCTATTTAATATAGCTATTTGTGCAAGTATTTTACGATTAAGAATCAACTGTCTCTTGTACAGATCGTGTATAAATTTACTATAATTATAGTATACGCCTATTTCTGTTTCGCGAATTGCTGCGTTTATCCGAGTAATCCACAAACGACGAAAATCTCTCTTTTTCCTATCTCTATCTCGATGAGCCGAAAACAAAGCTCTTATTTTCTGTTGAGCAATAGTACGAATAGTTTTTGAATGAGCCCCTCGAAAGCTTGATACAAATAAACGCATTTTTTTTCTACGTCTCCGAGCTATATATCCTCGTCTAACTCTGGTCATTGAATAAATGAAACTTTGCTAAATAATTAATTGATTTTCTTTCTTTGAGTTATTCTTTTTTCCCCGCCGGGTATATTAATAACAAAACGGATTTTTCCAATGTATAAAAAGAATTCTAATGGCTTTTGCTACTATAACCTTCCCGACCACGATTTTTTTCTTTTTTTTTCTAGGCATTTCGCCTCAACTCAAAATGAAATAAAAATAAATAAGAAATTGGATTGATACTAGGTATCAAAAAGAAAAACGTAGTAAATCGAGATGAATAAAGAAATAGTGGGTTCCTTCGTTTCTATGGTTATTTCTTAAACGGTGAGGTCCTCTCTATACACCGGAGCCCTTTACTTCGTTTAGTAAACGTTATTGGTAACTTGTACAATTCAAAATCTTTGGCTCTACCCATGAATTATCCAGTAATAGGTCTTTCACAACGAGATCCGCCTATACAGTAACGGTATTTAATTTGGAAGGTTAGCTGGATAGCTGACCCTGTTAGTCCGTTTTGCAAAAATGGGAGCATAATCTTTTTTCTTTTAAAATAGTACTTTCCCGCTTAATGTATAGCATTTGCTACCAATGGGAACTTGCTTCTCATCTTAAATTGAGCTGATTGGGGTTACAGCAAGGGAAACCATAAATTTCGTACACAATAGACGGATATGATAGATCTTTTTTTCGATAGTGACCAAAGTTCTTCCATTTTATCCTATTCACTGGTAATGATCATTGATACTGGAAAATTTATTTCTTTTGTTGGCCCAGCTCATAATCTAAACGAGTCGCACATACACCCTAGTACATGTTCCTCGGCGCTGAGGACATCCCCGAAGAGCGGGGGATTTCGTGACGTTTCTGATTGGCTGTCTTGTGTTTCTAATAAGCTGTTTAATAGTTGGCATGCTGAATCATTTACATTACATAAGGGACTGGTTTAGATCAATCCTAACCTGATGATTATGAATTTTTTCTAGTTAATATAATAGAATATTAAAAACCTAGTAAAGTAAAAAGATAAAATATCAATTTGCGAATTTGACTACTTCTACTCCTTCCATTTTTCCTTCTTTACTATTTCTACTCCTTCATTCGCTATAAGATCAATAATTCCGTGAGCTTGGGCTTCTGTTGCTGACATAAAAACATCCCTTTCCAGGTCTTCGGTTAGAACCCACAAGGGTTGGCCTGTTCTTTGTGCATAAACCTTTGTGAGGGTTTCTCGCAAAGTCAATAGTTCTTCCGCTTCCATCATACATTCTCCCGTTGATCCCTCATGAAAAGAACTAGCAGGTTGATGAATCATTACCCTGATGATATAACAAAAAGAGGGTTCCTCTATCTCGCATGATGAGGCGAAGACAAAAGATAGAGAATAACAATAAACTTGAACAACCGTACGTGCATCTTTTGCGCATTGCATACGGCTCGACAATGGAATTTACTTTTCTCTTCCATCGAAGAAAGATAGAATCGATCAGATCACGATCAGTAAATCATCCAATTACCACCCTTCTTTTCGTGAGTTCAAAATACTATGATGGCTCCGTTGCTTTCTATATTAATTTCGTTCATTTCGTCTGTAATTCAGCAATCCCAAAGTTTCTTTTTGATCCGAAATAAGGAATTTTTTTTTATTTTCGTACTCTTTCAAACATAAATATTGTTAGGATTAAGAGTCTTTTAATATAAAAATAAAAAAGTTTGTGACGCTGAAATGGACTCCGGATAAATAAAAAAATCGGGAATACCCTTTATCTCATACTCCTCTCTCGATACATAATCTAATATTTTGAAAAAAAAAACTAACAAAATTTTGCATATCGAATTCAAAGTGCCATGCTATTTTTACTATTTTTACTTAACACTACTTAACTTAATATATATTGATATTTCTTATGGTGAAGGCATAGTCTTTTTTTTTTCTTTCAAATAAAAAACTCATTGGCGCCAACCAAAGCCAAGCGTGAGGGAATGCAAAACGTTTGGTAATTTCTCCTCCGACCAGGATAAAAGATCCCATTGAAGCGGCTATTCCCATGCATATTGTATATACATCTGGTAGCACAAGTTGCATAGCATCAAAAATAGCTATTCCGGGTAATACCCATCCGCCAGGACAATTTATAAACAAATACAAATCCTGGGTCTGATCCTCTATACTGAGATATACGATAAGACCAACAAGGTAATTCGAGATCTCGGTCGTAATCTCTTGGGTTAAAAAAAGTAATCTTGCTCGATAAAGTCGGTTGATTAGGGTAAAATTGTATCCCTTAGGAACCGTACATGCACCTTTTGATGCATACGGTTCAAAAAAATTTTAAAAAGAAAAAAATCAATGTGTAGATTACTGCCCTCTTCTTTTTGGATAGCAGTTCGTTCTTTCTAACTTCTAATGATGGGGATTTGTCTTCTATTTTTCAATAAATATGAGTTTTTCATCGTTTCCTTATTTTTACTATAACTATAAATATATAACTATATATTATTTATAGAAATATATATATTATTTTATTTATAGAAATATATATATTATTTATAGAAATATAGAAATTAGAATTCTATATTATATATAATATAGATATAGAAAGAAAAAAAAAGATAATATAATGATAATATAGTAAATTAAAAAGATAATATAGAAGACTCCATATCTAGATACAAATCCATATCTAGATACCAAATAGAACAAAGGAATCATATTAATATGCAAATTAAAAATAAAAAAATCATAAATAGTAAAACAATAAAGAAATAGTATTAGTATAGTTATAGTATAGTAAGAGGTAAACTTTTCGAACTAACTGCTCGTTGATTTATTGTTTCATCGAGATCGAATGCAAACCACGATGTCATTTTCTTGTTCTTGAAGGGGCCTCTTTAATTCTTTTAGGTTTATGCTCTACTCCGGGTAAAAATCTGCTCGATTTTTATTTGCACATATAGGTCAAATGCGTCTAATACCGCTTCTTTTTGTTTTGCTAAGATTTCCTTTTTTCATTCAGTTCATGCCTTTGCCAAGTTGGATATTCGACATATTGCATTCATCTATTCTATGCGAGTGATTAAGGTTCAGATGATTCCTAGATCTATTCCATTTATAATTTGAAAATGAAAATAGGAATAATTTTTGATACAAGCAGTAATTATCGATATATTACCAATTGGGATTTGTTTAAACGGAGCCTGGATACTTCATGTCATTTTATTGGTTCAACCAAGCCAACCATAAATTATTCTAATTGATACTATTAGTCTGAATCCCCCTCCAAATGGATCTAGTTGGACTTCACGCTCCAAATTTTTGATGATTCAATCAATCTTTGTTGGGCGAAGGGAAGGATATCTCGATCGGGGAAGAGAACGGGGAAAGCCCATATGACCCAATATATCTGACAAGTCGCACTATACGTCAACCCAAGTTGCATCTTCGTCTCCCGGAATTCGAAAGGGTACTTTTGGAACACCAATAGGCATTAACTGACAGAAAAAAGAATTAAGTACTATATTTCACTTTGATATGGAAACGTAATAATCGGGCTATTCTCTTCATAATATCAACCTTTATCATATATTATGTCTAGAATACACATTAGAAAAGGTCATAAAAGACGATAGAATGACTAAAGGAAAATTCTTACGAATAGAGCCTTCCAAGGATCAACAAGTATGGCGGCGTTTGCTCATAGAAAAAGGTATCAACCCCCATTGCGTATTGGTACTTATCGGGTATAGAATAGATCTGCTTCTCTTTGTTCCTACAAACATAATTGTTCCATTATTACCAATAGAATAGAACAAATATTAACCCTTGCTTCGAGATAATCCACTGAACAGGGGTCCATTTATAGTCATAGTCTTTTCCAATGCAATAAAGTTACATAGTGTCTATTTTGATTTGATAAAGGGGTATTTCCATGGGTTTGCCTTGGTATCGTGTTCATACCGTCGTATTGAATGATCCTGGTCGGTTGATTTCTGTCCATATAATGCATACGGCTCTGGTTGCTGGTTGGGCCGGTTCGATGGCTCTATATGAATTAGCAGTTTTTGATCCCTCTGATCCCGTTCTTGATCCAATGTGGAGACAGGGTATGTTCGTTATACCCTTCATGACTCGTTTAGGAATAACTAATTCCTGGGGTGGTTGGAGTATTACAGGGGGGACGGTAACGGATCCCGGTATTTGGAGTTATGAAGGTGTGGCCGGGGCACATATTGTGTTTTCTGGCTTGTGCTTTTTGGCAGCTATTTGGCATTGGGTATATTGGGATCTAGAAATTTTTTGTGATGAACGTACAGGAAAACCTTCATTAGATTTGCCTAAGATTTTTGGAATTCATTTATTTCTTTCCGGGGTGGCTTGTTTTGGTTTTGGCGCATTTCATGTAACAGGCTTGTACGGTCCTGGAATATGGGTGTCTGATCCTTATGGACTAACTGGAAAAGTCCAGGCAGTAAATCCCGCATGGGGCGTAGAAGGTTTTGATCCTTTTGTGCCAGGGGGAATAGCCTCGCATCATATTGCAGCAGGGACATTGGGCATATTAGCGGGCTTATTCCATCTTAGTGTTCGCCCGCCCCAACGTCTATACAAAGGATTACGTATGGGTAATATTGAAACCGTCCTTTCCAGCAGTATCGCTGCTGTCTTTTTTGCAGCTTTTGTAGTTGCCGGAACTATGTGGTATGGTTCAGCAACGACTCCGATCGAATTATTTGGTCCCACTCGTTATCAATGGGATCAGGGATACTTTCAGCAAGAAATATATCGAAGAGTTAGTGCTGGGCTGGCCGAAAATCAAAGCTTATCAGAAGCTTGGTCGAAAATTCCTGAGAAATTAGCCTTTTATGATTACATTGGCAATAATCCGGCAAAGGGAGGATTATTCAGGGCAGGTTCAATGGACAATGGGGATGGAATAGCTGTTGGATGGTTAGGACACCCAATATTTAGAGATAAAGAAGGACGTGAGCTCTTTGTACGTCGTATGCCTACTTTTTTTGAAACATTTCCAGTCGTTTTGATAGACGGAGATGGAATTGTTAGAGCCGATGTTCCTTTTAGAAGAGCAGAATCGAAGTATAGCGTCGAACAAGTAGGTGTAACTGTTGAGTTCTATGGTGGCGAACTCAATGGAGTGAGTTATAGTGATCCTGCTACTGTGAAAAAATATGCTAGACGTGCTCAATTGGGTGAAATTTTTGAATTAGATCGTGCTACTTTGAAATCGGATGGTGTTTTTCGTAGTAGTCCAAGGGGTTGGTTTACTTTTGGACATGCTTCCTTTGCCCTGCTCTTCTTCTTCGGACATATTTGGCATGGGGCTAGAACCTTGTTCAGAGATGTTTTTGCTGGTATTGACCCAGATTTAGATGCTCAAGTAGAATTTGGAGCATTCCAAAAACTAGGAGATCCAACTACAAGAAGACAAGCAGTCTGATACAAAATTGCTTTTGTATTTTTCGTCTTTCTTTTTGTGATTTGATTTGACATTGGGGATCAGAGAAATCTTGATTTAATCATTACCCTTTCGTTGACTCTTTCTTTATCAGGGAAATAATCCCCAATAAACAGGTATGGAAGCTATAATTGTAAACCACAATCGAATCTATGGAAGCATTGGTTTATACATTTCTATTAGTCTCGACTCTAGGGATAATTTTTTTCGCTATCTTTTTTCGAGAACCGCCTAAAGTTCCAACTAAGAAATGATTTTTCATTATCTCCGTTGAAGTAATGAGCCTCCCAATATTGAATGAATATTGGGAGGCTCATTACTTCAACTAGTCCCCGTGTTCCTCGAACGGATCTCTTAGTTGTTGAGAGGGTTGCCCAAAAGCGGTATATAAGGCGTACCCGGTAAAACTTACAAGTAAACCAGATATAAAGATGGCGACTAGGGTTGCTGTTTCCATTCTTATATAAATTCAAGACCGCAATGGATCTCTGATAAGATCCTTTATTTACAAGGGAATGGTATACAAAGTCAACAGATCTCAATGAATACAATCGGATTTATGGCTACACAAACCGTTGAGAGTAGTTCTAGGTCTCGTCCAAGACAAACTACGGTAGGGGCTTTATTGAAACCATTGAATTCGGAATATGGTAAAGTAGCTCCTGGATGGGGAACTACTCCTTTGATGGGTGTCGCAATGGCTTTATTTGCAGTATTCCTATCTATTATTTTGGAGATTTATAATTCTTCCGTTTTACTGGATGGAATTTCAATGAATTAAATCCACAAGAACTACTAAGTTCGAGCTTTTCAATACAAAGTAAAGTGAAATTTCAGGTTTCCGATTTATAACCCCGTTGGTAGTTCGATCGCGGAATTTCTTTCTTTCTGTATTTCCGGAATATGAGTGTGTGACTTGTTATAATTGATCCTATTGATAATACAGAGAATGAGTCTGTCATCTTATCTTGATAGAGATGGTTCTACCTCGTCGGATACTCATCCTAGTATCTGGAACACGGGATATTATGAAATAGATCAAGAAATATTTGAACTATGATTCATACTTAATATTCAGACCTTGTGACCGGATTCTAAAAAATTTTCAACGAATTAGAAATAGTTATAAATTGAAAGATTTTTCTTTCTGTTTATGCTTATTTTGACCAAAAAGTAAATTCTTTCGTATTTTGAGTCATTATACCTATTGATTGAATAAGTGATGATCCAATAGTTCTTACTCAGGGAATCTTTGGGCTTGGGGTTTTTATTGAATCATCGTGGTTCTAGTATGAATCTGGGGTTTCAATTTTTTTTTTTTTTTTTTTATTTATAGGGTCTTAACAAGAGAAATTCCTATCAATAAGAAAAAACAATAGTTAAAGCCGGATTACACACAACTAACAAATCAAAGAAAAAATAGGGAAAGAGAAGATTCAAGAGGCCTGTAGTAACAATAAGAAGAGCCGACTTGATATTTTGGCATTATCACCACAAAGAAGAACTTTCGTATTTTTAATGCTTCGTATCTTCACTTCCGAGAAGATTAAATCGAAGGAGTAAGATATTTCTATTACATATGCGTTGGGAGCAGTATTTGTGTGTTTCTGCTTGAGCTGTACGAGATAAAATTCTCATATACGGTTCTCAGAGGGGGAGTCCCCCCGGTTTACCTATCTCAATAAAGTCTATGATTGGTTCGAAGAACGTCTCGAGATTCAGGCGATTGCAGATGATATAACTAGTAAATATGTTCCTCCTCATGTCAACATATTTTATTGTCTAGGCGGAATTACCCTTACTTGTTTTTTAGTGCAAGTAGCTACGGGGTTTGCTATGACTTTTTACTATCGTCCAACTGTTACTGAGGCGTTTGCTTCTGTTCAATACATAATGACTGAAGCTAATTTTGGTTGGTTAATCCGATCAGTTCATCGGTGGTCGGCAAGCATGATGGTTCTAATGATGATACTGCACGTATTTCGTGTGTATCTCACCGGTGGATTTAAAAAACCTCGAGAATTGACTTGGGTTACAGGTGTAGTTCTGGCTGTATTGACCGCGTCTTTTGGTGTAACTGGTTATTCCTTACCTTGGGACCAAATAGGCTATTGGGCGGTCAAAATTGTAACAGGCGTACCTGAAGCTATTCCGATAATAGGATCGCCTTTGGTAGAGTTATTACGCGGAAGTGCTAGTGTGGGACAATCCACTTTGACTCGTTTTTATAGTTTACACACCTTTGTATTGCCTCTTCTTACTGCTGTATTTATGTTAATGCACTTCCTAATGATACGTAAACAGGGTATTTCTGGTCCCTTATAGAGAAGCTTATAGAGAAGATAGATCATAGATCTTTGTAATCAATCATTTATCACTTGGGGAAGGAACAATAGTATTTCATTGCTACAAATATGTCTTATTAAAATGAATAAGACATGTTTTTGGACATTCTCTTTCCTTCAACCCCACAATATTGGAATATTGTATTATGTTATTTAACATAACACGACTAGTTGAAGGGAATTCTCCGAAACGAACATGGATTATGGGAGTGTGTGACTTGAACTATTGATTAGGCCGTGCAGATATATGCCCCTTTCTGCCACATTGAAATTCACAAACCAATGTGTCTTTGTTCCAACCACCGTATAAGCTCTATACAGACGATAGGCTGGTTCGCTTGAATAGAATTCTTTCTATGATCAGCCCCGAATCATGTCATGCATGAAAAGGCTCCGTAAGATCCAGTGGAATCAATGATTTGGCAGAATCCAGATTCCATTTTATCTATTTCATTTATTTAATTTAATGTTTTGTTTTAATAGTATGGAAATGCATTCATTTCCTCTGCATCGACCCGATCTATGATACTATCGGAGTGAAACAAGGCATCTAAAGAAGACTAGAGGCTATAATATGTTAGTTAGTAACAAGTAAACCCTTTGCTTTGTATCTAAAAAAAAATTTTTGGAGATAAACACCAATCGCAAGGTCTAAGACGACCCAGAAAGCATTTGAGCATGATTAACTTTGTAAGCCTACTTGGGGATTGAGCATTTATCTGGAAGAACGGAATTCCTTGTAATGGGTAGTTGCAACCTTGGAAAGGGGAATCTAGTCAAACTTTTCATATTTCATACAGAGAACCATTCATATATGTATGGATATAGACAACATAACATATATATCTTAATATAATATGTTGGTCTTTTTTATGTTGGTTCTTTTAATTCTTGCTCGAGCCGGATGATGAAAAATTCTCATGTCCGGTTCCTTCGGGGGATGAGTCTATAAGAATTCACCTATCCTAATAACAAAAAAACCTGACTTGAATGATCCTGTATTAAGAGCTAAATTGGCTAAAGGGATGGGTCATAATTATTACGGGGAGCCGGCATGGCCAAATGACCTTTTATATATTTTTCCAGTCGTCATTCTAGGGACTATTGCGTGTAACGTAGGCTTAGCCGTTCTCGAACCGTCAATGATTGGCGAGCCGGCAGATCCATTTGCAACTCCTTTGGAAATATTACCTGAATGGTATTTTTTTCCCGTATTTCAAATACTTCGTACAGTACCCAATAAATTATTGGGTGTTCTTTTAATGGTTTCAGTACCTGCGGGATTATTAACAGTACCCTTTCTGGAGAATGTTAATAAATTCCAAAATCCATTTCGTCGTCCAGTAGCCACAACCGTCTTTTTGGTTGGCACCGTAGTGGCCCTTTGGTTAGGTATTGGAGCAACATTACCCATTGATAAATCTCTAACTTTAGGTCTTTTTTAAATTGATTCAATTGTAAAATAATACGATGTGTGTATCTAGGGAATAGTTGCTTCAAAGTGAATTTTCCCTAGATACATCTATTCAATTGAATTCAGGAGCTATTCTGAACAATACGGATTGCACTAAAAAAAAATTGATTATTGGTTTTGAATTTTAAAGAAAAAAGGAAAGAAATCTAATGAATTTAAACCTTCTTTTTAGGTAAATCAATTGCGAAATGCTTTTCTAGAGTGCCCAATATTTGTTTTACATCTTCTATGCGAAAATGCTCAATTTTCATAAGATCTTCTTGACTGTTATTCAATAGGTCCAATAATGTATGTATATTGGAATTTTTGAGGCAATTGTAGATCCTGGGAGGCAATTCTAATTGATCAATAAAAATCGATTTCAATGCTATTTTTTTTTTGTTTTTTCTTAGTTCAGTCAATCTATCATGAAAGAAAAAAAGGGGTAAAGTAACCCTGTCTTGATTATCCTCGAAATGTAAGTTTTCTTCTTCCGCATATAGAAAGGGAATAAATAAATCAATTAAATTCCGGGAGGCTTCATAAAGTGCTTCTTTAGGAGTTAAACTGCCATTTGTCCATATTTCGAGAAAAAGTATCTCTTGTTTTTCATTACCATTACTATACGAATGAATACTATGATTCACATTTCGAACAGGCATGAATACAGCATCTATAGGATAACTTCCGTCTTGAAAGTTATTTGGCGTTTTTATACGAAATCCGCGATTCCTCTCGAGTTGTAATCCAATACGCAAATCAATTGGTTCCGTCAAGTTAGCTATATGCTGTGTAGTATCAACGATTTCCACATAAGGTGGTGAGATGATATCTTGAGCAGTTACACATCCAGGTCCCCTAACACAAATAGACGCGTCACAGGTTCCGTATAGATTACTTCTCAATACAATTTCTTTCAAATTCATTAAAATTTCATGTACTGATTCTTGAATACCTACTATAGTAGAATATTCGTGTGGTATTTTCTCAGATTTTGCACGTGTAATACATGTTCCTTCTATTTCTCCAAGCAGAGCTCTGCGCATCGCAATGCCTATTGTGTCGGCTTGACCTTTCATAAGTGGAGATAGAATAAAGCGTCCATAATAAAGACGTTTACTATCTGTTCTTGATTCAACACACTTCCACTGCAGTGTCCGAGTAGATACTCTTATTTTCTCTCGAACCATAGTAATATTATAGATAATAGATCAGATCATTGAGTCATTTATTTCTCTTGAAATCCCTTCAATGCTTATTTTTACACACGTCTTTTTTTAGGGGGCCGACAGCCATTATGCGGCATGGGGGTTACATCTCGTACGAAACTTAATAGTATGCCACTTCTACGAATAGCTCGTAATGCTGCATCCCTTCCGAGACCAGGACCCTTTATCATGACTTCTGCTCGTTGCATGCCTTGATCTACTACTTTACGAATAGCGTTTCCTGCTGCGGTTTGAGCAGCAAATGGTGTCCCTCTTTTTGCCCCTTTGAACCCGCAAGTACCGGCGGAGGCCCAAGAAACCACTCGACCTCGTACATCTGTAACAGTCACAATGGTATTGTTGAAACCGGCTTGAACATAAATAACTCCTCTTGGTATTTTACGTGCACTCTTACGTGAATTAATACGCCTATTCCTACGTGAACCAATTCTTGGTATGGGTTTTGCCATATTTTATCGTCTCATAAATATGAGTCAGAGATATATGGAGATATCCATTTCATGTCAAAACAAATCCTTTCATTTTTTTTTATTTCTACATCATTTGTACATCGAGTCCGTTAGAAAGTCCCTTTTTTTTATTAGTAGACTGATTATCCTTGTCGTTGTTTATGTTTCGGGTTGGAACAAATTACTATAATTCGTCCCCGCCTACGAATTAGTCGACATTTTTCACAAATTTTACGAACGGAGGCCCTTATTTTCATATTTGTCATTCCTTACTTTAATTCCGAATCTATTTCTTGGAAGAAAATAAGTTTCTTGAAATTTAGAATCTCAAATTGTATTCCGGAATGTAGAAGTTGAAAAACCACTTAATCGGTTGAATCCTTGTTACGGAGTCTATAAATTATACGTCCTCTGGTTGAATCATAACGGCTTACTTCAATTTTCACTCTATCCCCCGGTAGGATTCGTATAAAACTACGGCGTATCCTTCCTGAAACATAACCTAGAATCAGATCCTCATTATCTAAACGAACCCGGAACATACCGTTAGGAAGTGATTCAATAATTAAACCTTCATGAATCGATTTTTCTTCTTTCATTCCAGGCAAAACCCCCTTAAAGTATCAACTAATGGAGGAGGAGTGATATTAGACAACCCGTCCTTTCTCTTTTTTTCCAAAATAGGAAGTTTCGGATCCAATTCGTATATCAGAGGGATTACCATATATAACATAAAATTTCTCCGCCAATTCTTTCTAGTCGAGCCTCTCGGTCTGTCATTATACCTCGAGAAGTCGAAAGAATTAGAATCCCCATTCCACCTAAAATTCTAGGAAGTCGTTGATAGTTAGAATAGATTCGTAGACCAGGGCGACTGACCTGCTTTAAATTTAAAATTTTGGTATACGGCCCTTTCCTATTCCTTCTATGTCGTAGAGTTGAAACCAAAAAATATTTGTTTTTTTCCTGATGTTTCCTCACGTTTTCAATAAAACCTTCTCGTAAAAGTATTTTAACAAGGGTTTCCGTTATTTTAGTAGATGTTATTCGAACCGTTCCCTTTTTATTCATGTCAGCATTTCGTATCGAGGTTATTATATCAGCAATGGTGTCCCTATTCATGATGACCTAAATTTAGTGGTGCTCCGAATTTTGATATAATCAACGTGCTTTTATTAGTTTCTTTTTCTTATTCTTTTTTTTTTTTTATGATTTATATTTATGTAAAAAGGAAAGGTATATACGTGATACACAATCTACTACTAAATCGATTTCATTCAAATAGCCTACTATTCTCGTGGTTTATAATACCTCGGGGATTCTCGTGATTTATAATACCTCGGGAGCTAATGAAACGATTTTAGTAAAGTTTAACTGTCTCAACTCTCGGGCGATTGCACCAAAAACTCGAGTTCCTTTTGGATTTCCTTTTTGATCAATAATAACTGCAGCATTGTCATCATATCGTATTATCATACCGTTGTCACGTTTGAGTTCTTTGCGGGTACGTACAATTACAGCTCGGATCACTTCTGATCTTTCTAAGGGCATATTTGGTATTGCTTCTTTTATCACAGCAACAATAATGTCACCAATATGAGCATATCGACGATTGCTAGCTCCTATGATTCGAATACACATCAATTCTCGAGCCCCGCTGTTGTCTGCTACATTCAAATGGGTCTGAGGTTGAATCATATAATTTTTGAATCTTTCAATGCAAAGGCGAAAAAAGAAAAAGAAATATTATTTGTCCAAAACTTTGTCCAAAACAAAAAACTGGCGGTTGTTTTTTTATCCCAACGTTTGTTTCTACATTCCTATTTCTACATTCCTATCCTGAAATAAGAAATTGAGTTCGTATAGGCATTTTGGATGCCGCTAGTGAGATAGCCTTTCTGGCTATTTTTTCTGTTACTCCGCTTATTTCATAAAGTATTCGACCTGGTTTGACAACAGCTACCCAATATTCGGGGGATCCTTTCCCCGAACCCATACGTGTTTCCGCAGGTCTTACTGTAACGGGTTTGTCTGGAAATATACGTACCCATAGTTTTCCACCACGACGCACATTTCGTGTCATTGCCCGCCGACCTGCTTCTATTTGTCTAGATGTGATCCAAGCGGGTTCAAGTGCCTGAAGAGCATATCTGCCGAAACAAATACGATTACCTCGATAAGATATTCCCTTCATTCTTCCTCTATGTTGTTTACGGAATCGAGTTCTTTTGGGGTTATAGTGGATGGTTCTTTTTCAATTCCATCTCTATTACAGAACCGGACATGAGAATTTCTTCTCATCCGGCTCCTCGCGAATGAAATGATCAAAAAGAAAGTATATATTTTTGAATTAAAATGAAATAAAATAATAAATAAAATATTTTATTAATTTTTAATATAATATTTTATTTTAATTTTTTAATAAGTTTTTTTTAATATAGTTATAACAAATCTTTGTTTTGTTTTCGCTTTTATCGTATCAGATCACCTACATTTTAGCAATTCAATAATAAAAAAATGTCGTGGGCGAATATTTACTCTTTCAATATCTATTTCTGTTGTAGGGTTAGTTCATGACTTCTCAGAATAGATGAATTGGTCTCTGGTTTATTCCGCCATCCCGCCCGCTGAATCATGTGTATTCATTTTCAATTGAATCTTCTGTATTCACAGGTTCCATCGTTCCCACCGCTTCTTGATTAATGGTTAGGCCTGAATTTGACAACGGAGCTTTTACTTAAATTAATTTGTTCTTGAGTCAACGTTCTTAGTCTTTATTGGCTCGAGGCTCTTAATTTTGGTGCTATGAAGAGATTCATATAATGATAGATGAATCCGTATTGATGCTTTATTACACTGCCTTTTATGAGATGATTCATAGACCTTACATATTGGAATTATATATCATTGATAGATTTTTATATCTTTCTCTCACCTTCCATTTATCCACATCCTTTCGCTTACAACTCATAATCGGATTGCTTTTTCTTTTGTTTATGCCAAAACGAGTTCAGTTGCTGCAATGATACGACCAATATATCATATCTTGACTGCTTCCTTGGATCCAGATAATTTGAAGTGATGAGTTAGTTATTAGTTCTATAGTTATTAGTTCATATTATGGGTTGTTAATTTTTTATCTTAATCCTAACAAAAACCAACGAGTCACACACTAAGCATAGCAATTCGGTCAAAAGGGGTTCAATCAAATTTTTATTCAACCTTATAGAATTGATCATTTTTCTTTTTTGTTCTGTCATTGAATAGAAGGGAAAGACAAGTAAAGGCTTATTATTTTATTCCTCGTCTATAAATATCCAAATTTTTATACCTAAAACCCCATATATAGTTCGAACTGTATAGGCGCAATAATCAATTTTGGCGTGAATGGTTTGTAGGGGAACTCTACCTTCTCTGATCCATTCGATACGTGCAATTTCTTTTCCGTCGATACGTCCTGCAATTTGTATTTGAATTCCTTTTGTATCAGCCTGTTCGGTTAGTTCAATAGCTTTTTTCATCGCTTTTCGAAAAGAGACCCTATTTTTTAATTGTCCGGCTATAAATTCTGCAAGAATATTAGGGTGTCTATAAGGTTTTGCAATTCTTGTAATAGCAATGTTGAGTTTTCGGTTCACAGAATTTAATTCTTTTTGTACATTCATCTGTAGTTCTTCGATGCCTCGTGGTCTATTTTCTATTAATAACTTGGGGAATCCCATATAGATTATGACCTGGATCAGATCAATTCTTTTTTGAATTTCTATACGTGCAATTCCCTCGACACCGGAGGATGTTCTCATATTTTGTTGAGCATAATTCTTGATACAATCCCGTATTTTTTGATCTTCTTGTAAACCCTCAGAATAATTTTTTGGTTGTGCAAACCAAATGGAATAATGACTTTGGCTTGTACCAAGTCTGAAACCAAGTGGATTTATTTTTTGTCCCATATTGCCCCGCTAGATTTTAAACGGAAATTGCTAGGTAAATACTTTTCTCGATTATCTAATGTTTCATATACTTCATAGTAGGATATATCTTTCAATACAATAGTTATATGACAAGTGGGTCTTTTTATCAGATAACTACGCCCTCGAGCTCGAGGTTTTAATTTTTTCCTGGTAGTTCCTTTGTTGACTTCCGCTTTCCAAACGATTAAATCTTCTTTTTCGAAACCTTTATTGTGTTTAGCGTTTGCTGCTGCGGAATAAATCAATTTAAAAATGGGATAACATGCTCGATAAGGCATGAGTTCGAGTATCATAAGTGTTTCTTTATAGGAACGCCCGCGGATCTGATCAATTACTCTTCGCGCTTTGTGAACAGACATAGGTATATATTGGCCTAAAGCAGATACTTCAGTCAGCGACTTTCTTTTTTTTCTCATAAGTTTTACCTCTGCATTAATGAAGGATAAGCATCTCTATTTATTAATTATTAACGTATTAACGACGAGATCTATTATCGTTTTTTGCATGTCCCCGGAAATTTAGAGTAGGTGCAAATTCTCCCAATTTGTGGCCTACCATACGATCTGTTATATAAACAGGCAAATGTTCTCTTCCATTATGGATAGCAATAGTATGGCCAATCATTGTGGGGATAATGGTAGATGCCCGGGACCAAGTTACTATTATTTCTTTTTCCTCCTTTGTGTTAAGCTTATCAATTTTTCTTAATAAATGATTCGCTACAAAAGGATTTTTTTTTAGTGAACGTGTCACGGTTAATTACTCCTATTTTTTTTATTTAAAGACGAAGAAACTAATTCAAATTTCTCTCCTATTTACTACGTCGACGAATAATCAAATTATCACTATATTTATTCCTTTTTCTACTTCTTCTTCCAAGTGCAGGAAAGCCCCATTTATTTGTTGGGCTTTTTCTACCAATTGGGGCCCTCCCTTCACCACCCCCATGGGGATGGTCTACAGGGTTCATAACGACTCCTCTTACTACAGGACGTTTACCTAGCCAACGCTTAGATCCGGCTCTACCCAAACTTTTCTGGTTCACTCCAACATTCCCCACTTGTCCGACTGTTGCTGAGCAGTTTTTGGATATCAAACGGACCTCCCCAGAAGGTAATTTTAATGTGGCCGATTTCCCCTCTTTTGCAATCAGTTTCGCTACAGCACCCGCTGCTCTAGCTAATTGTCCACCCTTTCCAAGTGTGATTTCTATGTTATGTATGGCCGTGCCTAAAGGCATATCGGTTGAAGTAGATTCTTCTTTTTGATCAATCAAAACCCCTTCCCAAACTGTACAAGCTTCTTCCAAAGCATACGGCTTTCTGGATGTAGATGATGATATCTATACAGATGGATCTTCTATTTATCATACAATGAAGTACCACATGAGCAGATATATAGGAATCCCAATCTGCCGAATCACTCATGTTATGATCTTCTACATCCTAGGTCTTCCCGTTCCGTCATCTGGCTTATGTTCTTCATGTAGCATTCAGACCGAATGACTCTATGAAATTACGTCGATACTTCCACATATACATATTATGGGTAACGTAGGAGACATCTCTATTTTTCCCCGGGGAATCTTTAGACACTGCTTAGCTTTCAATTCGCCTCTGACCATCAAATGAAATGTGAATAACCCGTCCTCCTCTCTTTGAAAGAAGGGGCGCTTCCGGTTCTGTCGGTGCTTGAAACAATTTTGTCTTCTCCATATTACTATATCTCTAGAGTCAATAATTTTATATGAGGAACTACTGAACTCAATCACTTGCTGCCGTTACTCTTCAGTTTTCTGTTGAGGTCTATCCTCTAGAGGTACTCAAATTGGATCCGTGATCGATTTCTAGGTTTCGTCGTAAACCTAATTGGTTACTTCCAATTACGTAAATCCATAGTTCAAACCGCACTCAAAGGTAGGGCATTTCCCATTTTTATAGGAACTTCTGTACCAGAAACAATGGTATCTCCAATTATAGCCCCTCTGGGATGTAAAATATATCTCTTCTCACCATCCCCATAGTGTATGAGACAAATGTATGCATTTCGATTAGGGTCGTATTCTATGGTTACGATTCTACCATATATGTCTTTTTCATTCCGTCGAAAATCGATTTTACGGTATAGACGCTTATGACCTCCCCCTCTATGCCTTGCGGTAATGATTCCTCTGGCATTACGACCTTTACCACAACGACGCTGTCCATAGATCAAATTATTTCGTGGATTGGATTTCACTTGACTGTCTACGGCCCTATTGCCTGTGTTCGGGGTAGAAGTTTTGTATAAATGTATCGCCATGCTATTAAGTATTTTTATTTAAGTTCTTTTCTTTCTAAGAGGTGGAATAGAATAACCCGGTTGAAGCGTAATGATCATACGTCTGTAATGCATTGTATGTCCCATAATAGGTCCCATTCTTCTACCCTTTCCCGGGAGTCGATGACTATTCATAGCTATTACCTTGACACCAAAGAAGAGTTCGACCCAATGCTTTATTTCTGTCCTAGTTGATCCTGATTCGACATTAGAAGTATATTGATTTTTCCCCAATAACCGAATACTTTTGTCTGTAAATATTGCATATGTGATTCTATCTATTTTCTTCATATTCAAAATGGCATATATTGCATATGTGATTCTATCTATTTTCTTCCCTATGAGTTCGAGTCTCAATAAGAATGCTAGTTCTTACTGTTCCTATGTTATGAAATGAATATACCAATTCGTTATGTATGGAGGATGAGATTCCATTGATACAGAGCCAATTCCAATAGACTTATACTTATTGGAGGGTCCCATTGGCGTGCATCCAGTAGGAATTGAACCTACGAATTCGCCAATTATGAGTTGGGCGCTTTAACCATTCAGCCATGGATGCTTAGCGGGGATCCTCGTACATGGTGAATAACCAAATTCCAATTGAAATGAAATCTTTAGGATAAATCAATACAAATCCAATTTTAATTTTATACAAATATCATTGAAATTTTCATTGTTGACATTTTCATTGAAATAGAATATAATAGAATATAGAAATTTTAGACACATTTTCATTGAAATAGAATATAGAAATGTTAGACATATTTTCATTTTCAGTGATTAAATTTTCAGTGAAATATCATAATTGGAGTATCATAATCATATTATATTATAGATATACAATTACGTTTTCGTATTGTATTATACTATAGTATAAGTATCAAATTGAATTTTTTTTTAACTTTAACAATTGCATATTAAAGTAAATTTATAGTATTTTAAAATTCAATTTTTTTTTTAACAAATGACAAAAAAACATTCCACAAATTTAGATTTCTGGGTTTTCGAGTTGAAAGAGATATTGAGAGAGATCAAGAATTCAGTGGGATCTTTGGTTAAGATTTTTTTCCACCAAGAACGTTTTATAAAACTCTTTGATCCCCGAATTTGGAGTATCCTACTTTCACCCAATTCGCGGGGTTCAATAATTAAGCGATATTTCACTTTCACGATCACGGGTGTAGTATTCTTTGTAATCAAGGGTGTAGTATTCTTTGTAGTAGCGGGCCTTATATATCGTATTAACAATCGAAATATGGTCGAAAAAAAAAATATCTATTTGATAGGGCTTCTTCCTATACCTATGAATTCTCTTGGGTCCAGAAATGATACATTGGAAGAATCCTTTGGGTCTTCCAATATCAATAGGCTGATTGTTTCGCTCCTCTATCTTCCAAAAGGAAAAAAGATCTCTGAGAGCTGTTTCCTGGATCCGAAAGAGAGTACTTGGGTTCCTCCAATAACTAAAAGGTGTAAATCTAACTGGGGTTCGCGGTGGTGGAGGAACTGGATCGTAAAAAAGAGGGATTCTAGCCAATTGAAAGGATCTTTTGATCAATCTAGAGATCGCTTGGATTCCATCAGGAATGCGGATTCGGAATATCACACATCTCTCAATCAAAGGGAGATTCAACAGCGAAAAGAAAGATCGATTCCTTGGGATCCTTCCTTTCTTCAAACGGAAGAAACAGAGATAGGATCAGGCCGATTCCCGAAATGCCTTTCTGGATATTCCTCAATGTCTCGGCTATTCACGGAAGGTGAGAAGCAGATGAATAATCATCTGCTTCCGGAAGAAATCGAAGAACTTCTTGGGAATCCTACAAGATCCATTCGTTCTTTTTTCTCTGACAGATGGTCAGAACTTCATCTGGGTTCAAATCCTACTGAGAGGTCCACTAGAGATCAGAAATTGTTGAAGAAAGAACAAGATGTTTCTTTTGTCCCCTGCAGGCGATCGGAAAATAAAGAAATGGTTAATATATTCAAGATAATTACGTATTTACAAAAGACCGTCTCAATTCATCCTATTTCATCAGATCGGGGATGTGATATGGTTCCGAAGGATGAACCGAATATGGACAGTTCCAATAAGATTTCATTCTTGAACAAAAATCCATTTTTTGATTTATTTCATCTATTCCATGACCGGAACAGGGGGGGATACACGTTACACCACGATTTTGAATCCGAAGAGAGATTTCAAGAAATGGCAGATCTATTCACTCTATCAATAACCGAGCCGGATCTGGTGTATCATAAGGGATTTGCCTTTTCTATTGATTCCTACGGATTGGATCAAAAACAATTCTTGAATGAGGTATTCAACTCCAGGGATGAATCGAAAAAGAAATCTTTATTGGCTCTACCTCCTATTTTTTATGAAGAGAATGAATCTTTTTATCGAAGGATCAGAAAAAAATGGCTTCGGATCTCCTGCGGGAATTATTTGAAAGATACAAAAGAAAAAATAGTGGTATTTGCTAGCAACAACATAATGGAGGCAGTCAATCAATATAGATTGATCCGAAATCTGATTCAAATCCAATATAGCACTTATGGGTACATAAGAAATGTATTGAATCGATTCTTTTTAATAAATAGATCCGATCGCAACTTCGAATATGGAATTCAAAGGGATCAAATAGGAAACGATACTCTGAATCATAGAACTATAATGAAATATACGATCAACCAACATTTATCGAATTTGAAAAAGAATCAGAAGAAATGGTTCGATTCTCTTATTTTGATTTCTCGAAGCGAGAGATCCATGAATCGGGATCCTGATGCATATAGATACAAATGGTTCAACGGGAGCAAGAATTTCCAGGAACATTTCGTTTCTGAGCAGAAAAGCCGTTTTCAAGTTCAAGTAGTCTTCGATCGATTACGTATTAATCAATATTGGATTGATTGGTCTAAGGTTATCAACAAAAAAAAATTTTCTAAGTCATTGTCAAAGTTGATTCTCTTTTTGTCTAACTCACTTCCTTTTTTCTTTGTGAGTTTAGGGAATATGCCCATTCATAGGTCCGAGATCCACATTTATGAATTGAAAGGTCCGAATGATCAACTCTGCAATCCGTTGTTAAAATCACTAGGTCTTCCAATCGTTCATTTGAAAAAATGGAAAGCGGATGATCATGATACTTCCCAAAAATCGAAATTATTGATCAATGGAGGAACAATATCACCCTTTTTGTTCAATAAGATACCAAAGTGGAAGTGGATGATTGACTCCCATACTAGAAAGAATCCCAGGAAATCCTTTGATAACACGGATTCCTATTTCTCAATGATATCCTGCGATCAAGACAATTGGCTGAATCCCGTAAAAGCATTTCATAGAAGTTCATTGATATCTTCTTTTTATAAAGCAAATCGACTTCGATTCTTGAATAATCCACATCACTTCTTCTTCTATTGTAAGAAAAGATTCCCTTTTTATATGGAAAAGGCCCGTATCAAGAATTATGATTTTACGTATAGACAATTCCTCAATATCTTGTTCATTCGCAACAAAAAATTTTCTTTGTGCGTCGGTAAAAAAAAACATGCTTTTTTGGAGAGAGATACTATTTCACCAATCGAGTCACAGGTATCTAACATATTCATACCTAACGATTTTCCACAAAGGGGTAACGAAGGGTATAACTTGTACAAATCTTTCCATTTTCCAATTCGATCCGATCTATTCGTTCGTAGAACTATTTACTCGATCGCAGACATTTCTGGAACACCTCTAACAGAGGAAGAAATAGTCAATTTGGAAAGAACTTATTGTCAACCTCTTTCAGATATGAATCTATCTGATTCAGAAAGGAAGAACTTGCATCAGTATCTCAATTTCAATTCAAACATGGGTTTGATTCACACTCCACGTTCTGAGAAATATTTACCATCCGAAACGAGTCAAAAATTGCGTCTTTGGCGAAATTGGCTAAAGCTAAAGAAAGGCGTTGAGAAAGGGCAGATGGATAGAACCTTTCAACGAGATAGTGCTTTTTCAACTCTCTCAAAATGGAATCTATTCCAAACATATATGCCATGGTTCTTTACTTCGACAGGGTACAAATATCTAAATTTGCTATTTTTAGATGCTTTTTCAGACCTATTGCCGATGCTAAGTAGCAGTCACAAATTTGTATCCATTTTTCATTATATTATGCACAGATCAGCATGGCGAATTCTTAAGCTAAAATGGCGAGCTCTTAAGCTAAAATTGTGGGGATTGTGGGCACCAATAAGTGAGATTTCAAGTGATATTTCGTGGAAGTGTTTCCGTAGGCTTCTTCGGGTCGAAGAAATGATTCATCGAAATAATGAGTCACCATTGATATCGACACATCTGAGCTCGCCAAATGTTCGGGAGTTCCTCTATTCAAGCCTTTTACTTCTTCTTCTTGCTGGATGTCTCGTTCAGGTACTTCTTTTCTCTGTTTCCCTAGACTCTAGTGAGTTACAGACAGAGTTCGAGAGGATAAAATCTTTGACGATTCCATCATACACGATTGAGGTGTACAAACTTGTGGATGGGTATCCTAAACCTGAACCGAATTCTTTCTGGTTAAAGAATCTCTTTCTAGTTGCTCGGGAACAATTAGAAGATTTTCTAGCAGAAATACTGGGTTTTGCGCTATTTGGTGGTGGTCCCGCTTATGGGGTCAAATTTATACAGAAGATATTTTTCAATCTCATCGATCTCATAAGTATCATACCAAATCCCATCAATCGAATCACTTTTTCGAGAAATACGAGACATCTAAGTCATACAAGTAAAGAGATCTATTCATGGATAAGAAAAGGACAAAGGTTTCAGACTCATGATGAAATAGAATCCTGGATCGAGACCTGTGATTGGTTTTTGGATGAAGAGAGAGTTTACTCGTTTCATTTCTCCACCTTAAGGCCAGAAAAAGGGATTGATCAAATTCTATTGAGTCTGACTCATATTGATCATTTATTAAAGAGTGACTATGGTTATCAAATGTTTGAACAAGCGGGAGCAATTTACTTACGATACTTAGTTGACATTCATCAAAAGGATCTAATGAATTATGAGTTCAATACATCCTGTTTAGCAGAAAGACGGATATTCCTTGCTCATTATCAGACAATCACTTATTCACAAACCTCGTGTGGGGCTAATAGTTTTCATTTCCCATCTCATGGAAAACCAAAACCCTTTTCGTTCCGCCTAGCCCTATCCCCCTCTAGGGGTATTTTAGTGATAGGTCCTATAGGAACTGGACGATCCTATTTGGTCAAATCCCTAGCGACAAACTCCTATCTTCCTTTCATTACGGTATTTCTGAACAAGCTGGATTTAAAAATAGTTATTGATGATCTCGATCCTGAGGACTATATGGAAGCGCTTGATGATGTGGATATTGATGGTATTGATGATGATAGCGATCCTGCTAAGGAATATATGGATGCGCTGAAAGATGTGGACGATATTGATGATCGTGACTATATTTATTCGAACTTGGACTCGGACCCGGAGCTGAGGGAGGAGTATACGGTGGATGAGATACTTAGGTATATCATCGAGTTGGAAATAGACCTAGCTTCTATCAACTTGCAATTCGAATTGGCAAGAACAATATCTCCTTGCATAGTATGGATTCCAAACATTCATGATCTGTATGTGGATGAGTCGGAGTCCCTCGGTTTATTATTGAACTATCTCTCCGGGGATTGTGAAAGACGGTCCACTAGAGATATTCTTGTTATTGCTTCGACTCATATTCCCCAAAAAGTGGATCCCGCTCTAATAGCTCCGAATAAATTAAATACATGCATTAAGATACGAAGGCTTCTTATTCCACAACAACGAAAGCACGTTTTCACTCTTTCATATACTAGGGGATTTCACTTGGAAAAGAAAATGTTCCATACTAATGGATTCGGGTCCATAGCCATGGGTTACAATGCACGAGATCTTGTAGCAATTACCAATGAGGCCCTATCGATTAGTATTACACAGAAGAAATCAATTATAGACACTAATACAATTAGATTCGCTCTTCATAGACAAACTTGGGAGTTGCGAGCCCATGTAAGACCGGTTCCGGATCATGGGATCCTTTTCTATCAGATAGGAAGGGCTGTTGCACAAAATGTACTTATAAATAATTGCTGCCTTATAGATCCTATATCTATCTATATGAAGAAGCAATCATGTTACGAAGGGGATCCTTATTTGTACAAATGGTTCTTCGAACTTGGAACGAGCATGAAGAAATTAACGATACTTCTTTATCTTTTGAGTTGTTCTGCCGGATCGGTCGCTCAAGATCTTTGGTCTCTACCCGGACCCGATGAAAAAAATTGGATCACTTCTTATAGACTCGTTGAGACTGATTCTGATCTAGTTGATGGCCTATTAGAAGTAGTAGAAGGCGCTCTGGTGGGATCCTCGCTTCTTCGGCCCGAACCAAGGAATCCCTTAGAGATGATGGAAAATGGATCTCGTTCTATCTTTGATCGTAGATTTCTCTATGAATCGGAGTTTAAAGAATGGGCAGAAGGCACCGACCCGCAACAGTTAGCGGAGGATGTAGTCGATCACATAGTTTGGGCTCCTAGAATATGGCAACCTTGGGGCTTTCTATTTGATTGGATCGAAAGGCCCAATGAATTGGAATTTCCCTATTGGGCCAGGTCATTTCGGGGCAAGCCGATCATTTCTGATGAAGTTAATGATGAATATTTTGATTATGCATTTTATGGTGAAGGGGATGATGGATATGATGAAGAGGATGAGCTTCAAGAGAATGATTGGGAGTTCTTGCAGAGTGAAACCATGGAGTACCCGGGACGAGATAGATCTTCCAAAGAACAAGTCTTTTTTCGAAAAGGCCAATTCATTTGGGACCCTGGAGATCCACTCTTTTACATATTCAACGATGAGCTCTCTGTCTTTCTGTTTTCACATCGAGAATTCTTTGCAGATGAAGAGATGTCAAAGGGGCTTCTTCTGACTTCCCAAAGGGAGACTCTATATAAACGCGGGTTTAGCAAGAAACCGAAAGAAAAGTACTTCGAATTTTTTATTAATCGCCAGAGACGGAGACGGCTTAGAACCATTAGTTCATTATATAATAGATCTTTCCGTTCTAATATTCAATCCGCGAGTTATCAGTACTTATCAAATCTGTTCCTATCTAACGGAAGGCTATTGGATCAAATGACAAAGACATTGTTTAGAAAAAGATGGATTTTCCCGGATGAACTGAAAATTGGATTCATGTAACAGGAGAAAGATTTCCCATTCCTTAGCCGTAAAGATATGTGGCCATGAAAGAGGGATTAAGTGGAACAGAATTGACTGGGCGGTAGAGTCGTGGAAATACTTGTTTTTTCCATATTTCGGACCTTAGCTCCATGGAACAATATGCTACTGCTGAAAGATGGAAGAATTGAAATCTTAGATCAAAACAAAACACTATGTATGGATGGTATGAACGGCCTAAACAAGAATTCTTGAACAGCGAACAACCAGAGCCTATTACTCACTACATAAAAAAATTTCCATTAATGAAAGATGGAAATCCATTGTAAAATAAAAAATACGCATGTCTGATGAAAGTTGCTATCTGTTCCAATAACGAATCATTGGTTTGACTGAATAACTAAATAAAATACCCTATAGGGATAATACACATTCCAGTTGACCGAATTGTTTTGTTCCGAAGCAAAGGTATCCACGGGGTCCTTCGTCCTATTCATTCAGATATTCACGACCAAGAAGTACTGGATTCTCTTTCGGATAGGCCCCGAAAGGAGAAGGGAGGCTGGAATGCCAACAGGCGTCTATTCTTGAATTCACCCGACCCGATAGTACCCATTTTGGGGGGGAACGTCCAGTGCCAAAGTCACTAATGGGTAAGTCGCCAATCCCTAAAACGGACTATGTAATTTCTCTGCTGAGTTACGGGCGGGCATTTTACCAGAGGTTTCTATTGTATCAATCTACCCTTGTGTGATTCCTGTTGAAGCATATACTCGGGGGGGGGTTCAGGGCGGACGATTTCAAAGCGGACTCCCCCCTCATTAGATAGAGAAGATCACCAAGATTTCGTGATCCGCTGCCGAACTTATTCCAATTCCAAGATCTCTTATTGAATTGCTCATTCAATGAGCATTCTCTATGCCTTGAAGAGGACTCGAACCTCCACGCTCTTTAGCACGAGATTTTGAGTCTCGCGTGTCTACCATTTCACCACCAAGGCATCTTGAAAGTGATTCGTATTCCATGAATATGATATCTATCTAGTGTGATGTATGGAATATATGACAAAGGTGGAGTGTTGGAGTATTGCTATTGATCGGTCATGTCATATAGGCCCGAGTCGGACATCCAATTGCTTCGATTTTCATTTTCCGGAGGATGCCTTATACTTATATATATCAAAAAGATGGACAATCAAACCTATTTCTCGATTCAATAGAAGCCCAAAGAGATGAATAGGGTCCCAAATCAAATAACGAGAGATATGTAAAAAGAAGGTCCGATTACGCCTATTCCTAATCCGAAATGGAATGTAAGGACGTAGGGATCCATATGGAAATATAGTATCTATTTAGATAGGCTCGAATGACCCCTTCTCATAATGAGAATTTATATAACCCTCTTCCGGCCTAGTCCGGTATGGAATGAACTTATAATCATGGAATCGACTCGATCATCAGATTATAGATTATAAGTTCATAACCCTAGCCCATTCCCGTTTTGGGCGGAACAGATCTACTAATTCTTTGATTCCAGTTAGTAAGAGGGATGAAATAGACTCTAGAAGCTAAAAAAGGGTATCCTGAGCAATTGCAATAATAGGGTTCATTGATATTCCTGGTATAGTAGATGCTATCACACATACAATCATACTCAATTCGATGGAATTCTTTGATCTTAAAGGAGATCTTCTATAATTTCGCACGTGAGGGGTTATTTCTTGGTTTCGTCCAGTCATTAATAACTTGATTATTTTTAGATAATAGTAGATAGAAACAACGCTCGTAAGGAGTCCTATTGAAACCAAGAAATATAGGCCTGCCTGCCATCCACACCAGAATAAATGGAGTTTTCCGAAAAAACCTGCTAGTGGAGGAAGACCTCCTAGGGATAAGAGACATAGTGCTAAAGAGAGAGCCAAAGGGGGATCTTTCGTGTATAATCCTGCATAATCTCGAATGTTATCAGTTCCGGTACGTAGACCAAATGATACAATGCAAGCAAAAGTTCCTAGATTCATGGAGATATAGAACAGCATATAAGTTATCATGCTTGCATATCCATCATTTGAGTCTCCAACAATTATTCCAATAATTACATATCCGATTTGACCTATGGACGAATATGCAAGCATACGTTTCATGCTTGTTTGAGTAATAGCAATGAGATTCCCCACTATCATGCTAAGAATAGCTAGGATTTCCAGAAGAAGATGCCATTCGTTTGATGAGAAATAAAAAGGAATATCGAAAATTCGAGTGGCTGAAGCTGAAGCAGCTACTTTCGAAGTAACAGAAAGAAAAGCAACGACTGGAGTGGGAGAGTCAGAGTCGAAAAGAGGATTCCTCACTTCTTTCTCTCATTCAAAACCGTGCATGAGACTTTCATCTCGCACGGCTCCTAAGTGATAAAAGAAAGAAGAACTCATCTTCTTTCTTTTTTGATTACCTTCCTCGCGTATGTATAAGACCGAATCCGTTCGATTTATAAAAAGGATTACTAATCCTTAACTTTTCGAGGAATCCTTCATCAGTGGTTGCGAATGACTTATTTTTTCAATCTTTTCGACCTTGGTTCCGTAGGAGCAAGTCAGAAAGATTGAGAAATAGAACCATCTGATTTGATTCGTTCTCAATAGCCATGAGATGATCATCTTAGGGTGATCCTTTTGTCGACGGATGCTCCTATTACACTCGTAGTCTCTGAAGGATGAGAACCAACTATGTAGCATCTACATCGAGAATTCAAGTATTGTATACGTCATTAGTCCGATCCTTTGTAGGAACTACCCGTAATAACGAACTTGCAAAATGGATCTGTTTATCATAAAGAGATTCGTTGTTCCTGACCCTGCTTCACCTTAATTGTTATTTGAACAAGTAAAAGTTATGTCTTGGTCCGAGTGGGGATAGCATTTCTCTTCTGCATGTCCATGGAGTTTTGAAAAATCCAAACATCTCGGAGATAGATAGAGAGCTAGGAATTTTTCGAACGAACCGCACTCCTTCGTATACGTCAGGAGTCCATTGATGAGAAGGGGCTGGGGAAAGCTTGAACCCAATTCCTACAGTGATGAATATAAGCGCAATTGAAATTCCTGGGGAGTTATACATTTGTGTATTGATAAGACCATTCACTATTTCTTGAAGCTCGATCTCTCCCCCGGATGAACCATATAGCCAAGAGAAACCATGAACCAGAATAGAGGAGCTTGCCCCACCCATGAGTAAATATTTCGTAGTAGCCTCATTAGACCGTACATCTTTCTTGGTATATCCAGATAATAGGTAGGAGCATAAACTGAAACATTCTGGAGCTACAAAGATAGTTATTAAATCGTTAGCACCGCATAAAAACATTCCTCCTAGAGTAGCTGTTAATACGAATAACAGAAACTCTGTTATAGCCATTTCTGTACATTCAATGTACTCTACGGATAGAGGAATACATAGAGTTGAACATAGTAAAATAAGAAATTGAAAGATTTCGTTGAAATTGTTCGTTTGGAAATTTCCCGAAAAGCTAATCATAGGTTCTTCTCTCCATCGGAACAACAGGGCCGTTATGCTCATTACTAAACTTGTTGAAGAGATGAAATAGAACCAAGGTATATCTTTTTGATCAGAGGTTGAATCGATCATCAGAAGAAGAATTAGGCCAAAAATTAGGATACATTCTGGGAAAATAAAACTTCCATCGAAGAGAAGCAAATGAAAGGCTTTCATAAAAATTCTCGTAGAATCGAGAATGAAGTTTTCATTCTGTACATGCCAGATCATGAATTAGTAACTGCATCCAATCTCCAAAAAAATCCCAATTGTTTAGAACTTTCTATTTTTGAAATGGAATATTTACGGAATCTCCACGAATAGGATCCAACCCTATTCCATGGTATTTCCATGAGATTCCTCTTGCTTATTTATTCTTAAGCAAGTCCCCGAGAGGGCTTAGTTGATCCATGATTTATCTTTCATCTTTCGTTTCCTTTTCGTTTGTTTCGAAATAAATATATCGATCAATTCCGATTCTTTCTTTTTCTATTGATTCTTTTCCGATCGAGATGTATGGATCCATGGATCTAGGCGTCTACTATATAGATCCTGTTCATGGATTAACGAAAATGTGCAAAAGCTCTATTTGCCTCTGCCATTCTATGAGTCTCTTCCTTTTTGCGTATGGCACCGCCACTCCCCTTGGCAGCATCCACTAATTCGGAACTTAATTTGAAAGCCATATTTCGACCCGGACGTTTTCGGGATTCCCCTAATAACCAACGAATGGCAAGTGCTTTTCCTTGTGTGGATCCTATTTCAATGGGAACTTGATGAGTCGATCCGCTTACACGTCTTGCTTTTACTGCTATATCGGGAGTTACTCCACGTATTGCTTGACGTAAAACAGATAGTGGATTTGTTTCTGTCTCTTGTTGAATCTTTTTCACGGCTCGATAGATAATTTGATAAGCCAATGATTTTTTTCCGTGTTTCAGAATACGGTTAACCAACATGTTAACTAATCGATTACGATAAATTGGATCGGATTTTGCAGTTTTTTCTTCTGCAGTACCTCGACGTGACATGAGCGTGAAAGGGGTTCAAGAATCAGTTTTCTTTTTATAAGGGCTAAAATCACTTATTTTGGCTTTTTGACCCCATATTGTAGGGTGGATCTCGAAAGATATGAAAGATCTCCCCCCAAACCGTACATACGACTTTCATCGAATACGGCTTTCCACAGAATTCTATATGTATCTATGAGATCGAGTATGGAATTCTGTTTACTCACTTTAAATTGAGTATCCGTTTCCCTCCTTTTCCTGCTAGGATTGGAAATCCTGTATTTTCCATATCCATACGATTGAGTCCTTGGGTTTCCGAAATAGTGTAAAAAGAAGTGCTTCGAATCATTGCTATTTGACTCAGACCTGTTCTAAAAAAGTCGAGGTATTTCGAATTGTTTGTTTACACGGACAAAGTCAGGGAAAACCTCTTAAATTATGGACCTTGGACATATAATAGTTCCGAATCGAATCTCTTTAGAAAGAAGATCTTTTGTCTCATGGTAGCCTGCTCCAGTCCCCTTACGAAACTTTCGTTATTGGGTTAGCCATACACTTCACATGTTTCTAGCGATTCACATGGCATCATCAAATGATACAAGTCTTGGATAAGAATCTACAACGCACTAGAACGCCCTTGTTGACGATCCTTTACTCCGACAGCATCTAGGGTTCCTCGAACAATGTGATATCTCACACCGGGTAAATCCTTAACCCTTCCCCCTCTTACTAAGACTACAGAATGTTCTTGTAAATTATGGCCAATACCGGGTATATAAGCAGTGATTTCAAATCCAGAGGTTAATCGTACTCTGGCAACTTTACGTAAGGCAGAGTTTGGTTTTTTGGGTGTGATAGTGGAAAAGTTGACAGATAAGTCACCCTTACTGCCACTCTACAGAACCGTACATGAGATTTTCACCTCATACGGCTCCTCGTTCAATTCTTTCGAAGTCATTGGATCCCTTCCCTCGTTCGAGAATCTCCTCCCTTCTTCCACTCCGTCCCGAAGAGTAACTAGGACCAATTCAGTCATGTTTTCATGTTCCAATTGAACACGTTCCACTTTTGATTATTCTCAAAGGAGAAGATTATTCTTTTTACCAAACATATGCGGATCCAATCACGATCTTCTAACAAGAACAAGAGATCTTTCTCGATCAATCCCCTTGCCCCTCATTCTTCGAGAATTAGAAAGATCCTTTTCAAGTTTGAATTTGTTTTCATTTGGAATCTGGGTTCTTCTACTTTTTACTTTTTTTCTATTTTTTCCCTCTCTTTTTTTATTCCCTTCCATAATTCCTTAAGTCTCATAGGTTTGATCCTGTAGAATCTGACCCATTTTCTCATTGAGCGAGGGATACGAAATAAATCAGATTGATTTTCATTTTTCGATCAAAAGTACTATGTGAAATCTTCGGCTTTTCCTCTTCCTCTATTCCTATCCCATAGGTACAGCGTTTAAATCAATAGAGAACTTTCTGTATGAATCGATATTATTACATTCCATCCAATTCCTTCCCGAAACCTCCCAAGGAAAATCCCGAATTGGATCCCAAATTGACGGGTTAGTGTGAGCTTATCCATGCGGTTATGCACTCTTCGAATAGGAATCCATTTTCTGAAAGATCCTGGCTTTCGTGCTTTGGTGGGTCTCCGAGATCCTTTCGATGACCTATGTTGTGTTGAAGGGATATCTATATGATCCGATCGATTGCGTAAAGCCCGCGGTAGCAACAGAACCGGGGAAAGTATACAGAAAAGACAGTGCTTTTCTATTATATTAGTATTTTCTATTAGATTAGTATTGGTTAGTGATCCCGGCTCAGTGAGTCCTTTCTTCCGTGATGAACTGTTGGCACCAGTCCTACATTTTGTCTCTGTGGACCGAGGGGAAAGGGGGCTCGTCGGGAAGAGGATTGTACGATGAGAGAAGCAAGGAGGTCAACCCCTTTCAAATATACAACATGGATTCTGGCAATGCAACGTAGTTGGACTCTCATGTCGATCCGAATGAATCATCCTTTCCACGTAGGTAAATCTTTGCTTGCTAGTCAAGAGGATAGCAAGTTACAAATTCTGTCTCGGTAGGACATGTATTTCTATTACTATGAAATTCATAAATGAAGTAGTTAATGGTGGGGTTACCATTATCCATTTTGGAGTGACGAATCCTGTATGTGTTCCTAAGAAAAGGAATTTGTCCATTTTTCGGGGTCTCAAAGGGGCGTGTAAACACATAAGAACTCTGGAATGGAAATGGAAAAGAGATGGAACTCCAGTTCCTTTGGAAATGGTAAGATCTTTGGCGCAAGAAGAAGGGGTTGATCCGTATCATCTTGACTTGGTTCTGCTTCCTCTATTTTTTTTTTAATACCGAGTCGCGTTCTTCTCCTACCTATATCGAATAGAACATGCTGAGCCAAATCTTCTTCATGTAAAACCCGCTTGATTTAGATCGGGAAAATCGTACGGTTTTATGAAACCATGTGCTATGGTTCGAATCCGTAGTCAATCCGATTTCCGATAGGAGCAGTTGACAATTGAATCCAAATTTTTCCATTCTTTTCGTATCCGTAATAGTGCGAAAAGAAGGCCCGGCTCCAAGTTGTTCAAGAATAGTGGCGTTGAGTTTCTCGACCCCTTGCCTTAGGATTAGTCAGTTCTATTTCTCGATGGGGGCAGGGAAGGGATATAACTCAGCGGTAGAGTGTCACCTTGACGTGGTGGAAGTCATCAGTTCGAGCCTGATTATCCCTAAACCCAATGTGAGTTTTTCTATTTTGACTTGCTCCCCCGCCGTGATTGAAAGAGAATGGATAAGAGGCTCGTGGGATTGACGCGAGGGGCTAGGGATGGCTATATTTCTGGGAGCGAACTCCATGCGAATAGGAAGCGCATGGATACAAGTTATGCCTTGGAATGAAAGACAATTCCGAATCTGCTTTGTCTACGAACAAGGAAGCTATAAGTAATGCAACTATGAATCTCATGGAGAGTTCGATCCTGGCTCAGGATGAACGCTGGCGGCATGCTTAACACATGCAAGTCGGACGGGAAATGTTGTTTCCAGTGGCGGACGGGTGAGTAACGCGTAAGAACCTGCCCTTGGGAGGGGAACAACAGCTGGAAACGGCTGCTAATACCCCGTAGGCTGAGGAGCAAAAGGAGGAATCCGCCCGAGGAGGGGCTCGCGTCTGATTAGCTAGTTGGTGAGGCAATAGCTTACCAAGGCGATGATCAGTAGCTGGTCCGAGAGGATGATCAGCCACACTGGGACTGAGACACGGCCCAGACTCCTACGGGAGGCAGCAGTGGGGAATTTTCCGCAATGGGCGAAAGCCTGACGGAGCAATGCCGCGTGGAGGTAGAAGGCCCACGGGTCGTGAACTTCTTTTCCCGGAGAAGAAGCAATGACGGTATCTGGGGAATAAGCATCGGCTAACTCTGTGCCAGCAGCCGCGGTAAGACAGAGGATGCAAGCGTTATCCGGAATGATTGGGCGTAAAGCGTCTGTAGGTGGCTTTTTAAGTCCGCCGTCAAATCCCAGGGCTCAACCCTGGACAGGCGGTGGAAACTACCAAGCTGGAGTACGGTAGGGGCAGAGGGAATTTCCGGTGGAGCGGTGAAATGCGTAGAGATCGGAAAGAACACCAACGGCGAAAGCACTCTGCTGGGCCGACACTGACACTGAGAGACGAAAGCTAGGGGAGCGAATGGGATTAGATACCCCAGTAGTCCTAGCCGTAAACGATGGATACTAGGCGCTGTGCGTATCGACCCGTGCAGTGCTGTAGCTAACGCGTTAAGTATCCCGCCTGGGGAGTACGTTCGCAAGAATGAAACTCAAAGGAATTGACGGGGGCCCGCACAAGCGGTGGAGCATGTGGTTTAATTCGATGCAAAGCGAAGAACCTTACCAGGGCTTGACATGCCGCGAATCCTCTTGAAAGGGAGGGGTGCCTTCGGGAACGCGGACACAGGTGGTGCATGGCTGTCGTCAGCTCGTGCCGTAAGGTGTTGGGTTAAGTCCCGCAACGAGCGCAACCCTCGTGTTTAGTTGCCAACGTTGAGTTTGGAACCCTGAACAGACTGCCGGTGATAAGCCGGAGGAAGGTGAGGATGACGTCAAGTCATCATGCCCTTTATGCCCTGGGCGACACACGTGCTACAATGGCCGGGACAAAGGGTCGCGATCCCGCGAGGGTGAGCTAACCCCAAAAACCCGTCCTCAGTTCGGATTGCAGGCTGCAACTCGCCTGCATGAAGCCGGAATCGCTAGTAATCGCCGGTCAGCCATACGGCGGTGAATTCGTTCCCGGGCCTTGTACACACCGCCCGTCACACTATGGGAGCTGGCCATGCCCGAAGTCGTTACCTTAACCGCAAGGAGGGGGATGCCGAAGGCAGGGCTAGTGACTGGAGTGAAGTCGTAACAAGGTAGCCGTACTGGAAGGTGCGGCTGGATCACCTCCTTTTCAGGGAGAGCTAATGCTTGTTGGGTATTTTGGTTTGACACTGCTTCACACCCAAAAAGAAGCGAGCGACGCCTGGGTGAAACTTGGAGATGGAAGTCTTCTTTCGTTTCTCGACGGTGAAGTAAGACCAAGCTCATGGGCTTATTATCCTAGGTCGGAACAAGTTGATAGGATCCCCCCTTTTTCGCCCCCATGTCGCCACACGGGGGACATGGGGACGTAAAAAAGAAAGAGAGGGATGGGGTTTCTCTCGCTTTTGGCATAGCGGGCCTCCCACTGGGGGCCCGCACGACGGGCTATTAGCTCAGCGGTAGAGCGCGCCCCTGATAATTGCGTCGTTGTGCCTGGGCTGTGAGGGCTCTCAACCACATGGATAGTTCAATGTGCCCATCCGCGCCTGACCTTGAGATGTGGATCATCCAAGGCACATTAGCATGGCGTACTCCTCCTGTTCGAACCGGGGTTTGAAACCAAACTTCTCCTCAGGAGGATAGATGGGGCGATTCAGGTGAGATCCAATGTAGATCCAACTTTCGATTCACTCGTGGGATCCGGGCGGTCCGGGGGGGACCACTACGGCTCCTCTCTTCTCGAGAATCCATACATCCCTTATCAGTGTATGGACAGCTATCTCTCGAGCACAGGTTTAGGTTCGGCCTCAATGGGAAAAAATGGAGCACCTAACAACGCATCTTCACAGACCAAGAACTACGAGATCACCCTCTTCATTCTGGGGTGACGGAAGGATCGTACCATTCGAGCCTTTTTTTTTTCATGCTTTCCCGGAGGTCTGGAGAAAGCTGCAATCAATAGGATTTCCCTAATCCTCCCTTCCCGAAAGGAAGAACGTGAAATTCTTTTTCCTTTCCGCAGGGACCAGGAGATTGGATCTAGCCGTAAGAAGAATGCTTGGTATAAATAACTAACTTCTTGGTCTTCGACCCCCTCAGTCACTACGAACGCCCCCCGATCAGTGCAATGGGATGTGTCTATTTATCTATCTCTTGACTCGAAATGGGAGCAGGTTTGAAAAAGGATCTTAGAGTGTCTAGGGTTGGGCCAGGAGGGTCTCTTAACGCCTTCTTTTTTCTTCCCATCGGGGTTATTTCACAAAGACTTGCCATGGTAAGAAGGAAGAAGGAGCGAACAAGCACACTTGGAGAGCGCAGTACAACGGAGAGTTGTATGCTGCGTTCGGGAAGGATGAATCGCTCCCGAAAAGGAATCTATTGATTCTCTCCAAATTGGTTGGACCGTAGGTGCGATGATTTACTTCACGGGCGAGGTCTCTGGTTCAAGTCCAGGATGGCCCAGCTGCGCCAGGGAAAAGAATAGAAGAAGCATCTGACTCCTTCATGCATGCTCCACCTGGCTCGGGGGGATATAGCTCAGTTGGTAGAGCTCCGCTCTTGCAATTGGGTCGTTGCGATTACGGGTTGGATGTCTAATTGTCCAGGCGGTAATGATAGTATCTTGTACCTGAACCGGTGGCTCACTTTTTCTAAGTAATGGGGAAGAGGACCGAAACATGCCACTGAAAGACTCTACTGAGACAAAGATGGGCTGTCAAGAACGTAGAGGAGGTAGGATGGGCAGTTGGTCAGATCTAGTATGGATCGTACATGGACCGTAGTTGGAGTCAGCGGCTCTCCTAGGGTTCCTTAATCTGGGATCCCTGGGGAAGAGGATCAAGTTGGCCCTTGCGAACAGCCTGATGCACTATCTCCCTTCAACCCTTTGAGCGAAATGCGGCAAAAGGAAGGAAAATCCATGGACCGACCCCATCGTCTCCACCCCGTAGGAACTACGAGATCACCCCAAGGACGCCTTCGGCATCCAGGGGTCACGGGCCGACCATAGAACCCTGTTCAATAAGTGGAACGCATTAGCTGTCCGCTCTCCGGTTGGGCAGTAAGGGTCGGAGAAGGGCAATCACTCATTCTTAAAACCAGCATTCTTAAGACCAAAGAGTTGGGCGGAAAAGGGGGGAAAGCTCTCCGTTCCTGGTTCTCCTGTAGCTGGATCCTCCGGAACCACAAGAATCCTTAGTTCGAATTGGATTCCAACTCAGCACCTTTTGAGATTTTGAGAAGAGTTGCTCTTTGGAGAGCACAGTACGATGAAAGTTGTAAGCTGTGTTCGGGGGGGAGTTATTGTCTATCGTCAGCCTCTATGGTAGAATCAGTCGGGGGGCCTGAGAGACGGTGGTTTACCCTGTGGCGGATGTCAGCGGTTCGAGTCCGCTTATCTCCAACTCGTGAACTTAGCCGATACAAAGCTATATGATAGCACCCAAATTTTCCGATTCGGCGGTTCGATCTATGATTTATCATTCATGGACGTTGATAAGATCCTTCCATTTAGCTTAGCAGCACCTTAGGATGGCATAGCCTTAAAGTTAAGGGCGAGGTTCAAACGAGGAAAGGCTTACGGTGGATACCTAGGCACCCAGAGACGAGGAAGGGCGTAGTAAGCGACGAAATGCTTCGGGGAGTTGAAAATAAGCATAGATCCGGAGATTCCCGAATAGGTCAACCTTTCGAACTGCTGCTGAATCCATGGGCAGGCAAGAGACAACCTGGCGAACTGAAACATCTTAGTAGCCAGAGGAAAAGAAAGCAAAAGCGATTCCCGTAGTAGCGGCGAGCGAAATGGGAGCAGCCTAAACCGTGAAAACGGGGTTGTGGGAGAGCAATACAAGCGTCGTGCTGCTAGGCGAAGCGGTGGAGTGCTGCACCCTAGATGGCGAGAGTCCAGTAGCCGAAAGCATCACTAGCTTACGCTCTGACCCGAGTAGCATGGGGCACGTGGAATCCCGTGTGAATCAGCAAGGACCACCTTGCAAGGCTAAATACTCCTGGGTGACCGATAGCGAAGTAGTACCGTGAGGGAAGGGTGAAAAGAACCCCCATCGGGGAGTGAAATAGAACATGAAACCGTAAGCTCCCAAGCAGTGGGAGGAGCACACAGGGCTCTGACCGCGTGCCTGTTGAAGAATGAGCCGGCGACTCATAGGCAGTGGCTTGGTTAAGGGAACCCACCGGAGCCGTAGCGAAAGCGAGTCTTCATAGGGCAATTGTCACTGCTTATGGACCCGAACCTGGGTGATCTATCCATGACCAGGATGAAGCTTGGGTGAAACTAAGTGGAGGTCCGAACCGACTGATGTTGAAGAATCAGCGGATGAGTTGTGGTTAGGGGTGAAATGCCACTCGAACCCAGAGCTAGCTGGTTCTCCCCGAAATGCGTTGAGGCGCAGCAGTTGACTGGACATCTAGGGGTAAAGCACTGTTTCGGTGCGGGCCGCGAGAGCGGTACCAAATCGAGGCAAACTCTGAATACTAGATATGACCTCCAAATAACAGGGGTCAAGGTCGGCCAGTGAGACGATGGGGGATAAGCTTCATCGTCGAGAGGGAAACAGCCCGGATCACCAGCTAAGGCCCCTAAATGACCGCTCAGTGATAAAGGAGGTAGGGGTGCAGAGACAGCCAGGAGGTTTGCCTAGAAGCAGCCACCCTTGAAAGAGTGCGTAATAGCTCACTGATCGAGCGCTCTTGCGCCGAAGATGAACGGGGCTAAGCGATCTGCCGAAGCTGTGGGATGTAAAAATGCATCGGTAGGGGAGCGTTCCGCCTTAGTAGGAAGTACCGGCGCGAGCCGCGGTGGACGAAGCGGAAGCGAGAATGTCGGCTTGAGTAACGAAAACATTGGTGAGAATCCAATGCCCCGAAAACCTAAGGGTTCCTCCGCAAGGTTCGTCCACGGAGGGTGAGTCAGGGCCTAAGATCAGGCCGAAAGGCGTAGTCGATGGACAACAGGTGAATATTCCTGTACTACCCCTTGTTGGTCCCGAGGGACGGAGGAGGCTAGGTTAGCCGAAAGATGGTTATCGGTTCAAGGATGCAAGGTGACCCTGCTTTTTCAGGGTAAGAAGGGGTAGAGAAAATGCCTCGAGCCAATATTCGAGTACCAGGCGCTACGGCGCTGAAGTAACCCATGCCATACTCCCAGGAAAAGCTCGAACGACCTTCAACAAAGGGGTACCTGTACCCGAAACCGACACAGGTGGGTAGGTAGAGAATACCTAGGGGCGCGAGACAACTCTCTCTAAGGAACTCGGCAAAATAGCCCCGTAACTTCGGGAGAAGGGGTGCCTCCTCACAAAGGGGGTCGAAGTGACCAGGCCCGGGCGACTGTTTACCAAAAACACAGGTCTCCGCAAAGTCGTAAGACCATGTATGGGGGCTGACGCCTGCCCAGTGCCGGAAGGTCAAGGAAGTCGGTGACCTGATGACAGGGGAGCCGGCGACCGAAGCCCCGGTGAACGGCGGCCGTAACTATAACGGTCCTAAGGTAGCGAAATTCCTTGTCGGGTAAGTTCCGACCCGCACGAAAGGCGTAACGATCTGGGCACTGTCTCGGAGAGAGGCTCGGTGAAATAGACATGTCTGTGAAGATGCGGACTACCTGCACCTGGACAGAAAGACCCTATGAAGCTTCACTGTTCCCTGGGATTGGCTTTGGGCCTTTCCTGCGCAGCTTAGGTGGAGGGCGAAGAAGGCCCCCTTCCGGGGGGGCCCGAGCCGTCAGTGAGATACCACTCTGGAAGAGCTAGAATTCTAACCTTGTGTCAGGACCCACGGGCCAAGGGACAGTCTCAGGTAGACAGTTTCTATGGGGCGTAGGCCTCCCAAAAGGTAACGGAGGCGTGCAAAGGTTTCCTCGGGCCGGACGGAGATTGGCCCTCGAGTGCAAAGGCAGAAGGGAGCTTGACTGCAAGACCCACCCGTCGAGCAGGGACGAAAGTCGGCCTTAGTGATCCGACGGCGCCGAGTGGAAGGGCCGTCGCTCAACGGATAAAAGTTACTCTAGGGATAACAGGCTGATCTTCCCCAAGAGATCACATCGACGGGAAGGTTTGGCACCTCGATGTCGGCTCTTCGCCACCTGGAGCTGTAGTATGTTCCAAGGGTTGGGCTGTTCGCCCATTAAAGCGGTACGTGAGCTGGGTTCAGAACGTCGTGAGACAGTTCGGTCCATATCCGGTGTGGGCGTTAGAGCATTGAGAGGACCTTTCCCTAGTACGAGAGGACCGGGAAGGACGCACCTCTGGTGTACCAGTTATCGTGCCCACGGTAAACGCTGGGTAGCCAAGTGCGGAGCGGATAACTGCTGAAAGCATCTAAGTAGTAAGCCCACCTCAAGATGAGTGCTCTCCTATTCCGACTTCCCCAGAGCCTCCGGTAGCACAGCCGAGACAGCGACGGGTTCTCTGCCCCTGCGGGGATGGAGCGACAGAAGTTTTGAGAATTCAAGAGAAGGTCACGGCGAGACGAGCCGTTTATCATTACGATAGGTGTCAAGTGGAAGTGCAGTGATGTATGCAGCTGAGGCATCCTAACAGACCGATAGACTTGAACCTTGTTCCTACATGACCCGATCAATTCGATCAGGCACTCGCCATCTATTTTCATTGTTCAACTCTTTGACAATACGAAAAAACCATTGTTCAACTCTTTGACAACATGAAAAACCAAAAGCCCTGCCCTCCCTCTATATCTAACCAAGGGATGGAAGGGCAGAGGCCTTTGGTGTCCCCTCCAGTCAAGAATTGGGGCCTCACAATCACTAGCCAATTTTCTCTCATGCCTTTCTTAGTTCGTGGTTCGATATTCTGGTGTCCTAGGCGTAGAGGAACCACACCAATCCATCCCGAACTTGGTGGTTAAACTCTACTGCGGTGACGATACTGTAGGGGAGGTCCTGCGGAAAAATAGCTCGACGCCAGGATGATAAAAAGCTTAACACCTCTCATTCTTATTACTTTTTCAATATGAAAGAAAAAAAATGAAAAAAGGAAAAAAGAAAGGGTCGTCTTATTCAAAACCCCAATTATGACATCCCCTCTCTCCCACTTCACACCTCGGAACGCACCGTTCTTATAGATAGAAACGCGCTTTCACATCTTCTTAACCCGAAATGGCTGGGGAGAGGAAAGGTTCCTTTTTTTTGAGGATACTCCCGGGAACAGATCCAGTGGAGGCGGGGTGGGGCCTGTAGCTCAGAGGATTAGAGCACGTGGCTACGAACCACGGTGTCGGGGGTTCGAATCCCTCCTCGCCCACAACCTTCCCCTTTGGGAAGGACCTATCCCTCCGGGGATAGGAAAATCATGATCGGGATAGCGGACCCAAAGCTATGGGACTTGGGTGTGGGTCTTTTGTCGAAATGGAATGCACCTTACCTTTTTTTTTTACGTGTTATCGTGAATGAGGTAATTAAAAATTATAGCATACCCCGGCCGCCGGCTGGCAGCATATTTTTTTTTGTTTTACGTCCCGTAACTCTTCCTCAGCCAGGCTTGGGCAGAATAGCAGAGCAAGTACAAGTATTAGTAGCATAGCAAAAAAGCGTTCCTCGTCATTAATATGTTTTATGTTTGCTCGCGGTAATTGTAGCCTCTCGGGAGAATCGATGACTCCATCTTTGATGCACTGCTAGTACTAGTACATCCGAGAATTCTGAATTGGCTAGTTGTAAATAGCCCCAGGACTATGGAACAAAGGATTATCCCGGACCTACATCGAGGTATTGACGGAGATTCTCAAATATCGCAGAACAGAATGTCATACGATGAGATAGAATACAATAGAAACAAAGACAGGGAACGGGTTACCTACTCTTAACGGTCAAAGCGAGCCCTTTCATTCTCATTCTGAATTAAAGAATTCAGAATGAATCAAATCTCCCCAAGTAGGATTCGAACCTACGACCAGTCAGTTAACAGCCGACCGCTCTACCACTGAGCTACTGAGGAACAACGGGGGATTTGATCTCATAGAGTTAAATTCCCGTTCTCAACCCATGACCCATATGAGCTCGAAGCTTCCTTCGTAACTCCCGAAACTTCTTCGTAGTGGCTCCCTTCCATGCCTCATTTCATAGGGAACCTCAAAGTGGCTCTATTTCATTATATTCCATCCATATCCCAATTACATTCATTTAATATCCCTTTGGTGTCATTGACATAAGAGATCTCGTTTCTAGTCTATCTCTTTCTATTTCTATCTAGAATAAAATAAGTTAAACAATTATCTTATAATAATTATCATATAAGATAAGAAGGTAAAAACTTATCATATATAATAGAATCATATAGAATAGAATATAGAATAGAATAATATTAATATAATAATAATATAAATAGAATAATATAAATAATAGAATTTTATAATTTTATAAGGTCAAATAATAAGGTCAAAGATTATCTTATAATAAGAAGGTAAAAACTTATTATATAATAATCATAATTCAGAAATTGCAAAATCACAAAGGGGGTTTGTCATGATTTTTTTTCTACTAGGTAACTTATGCATGAAGATAGTCAATTCGGTCGTTGTGGTCGGACTCTATTATGGATTTCTGACCACATTCTCCATAGGGCCCTCCTATCTCGTCCTTCTCCGAACTCTGGTTATGGAAGAAGGAGAAGAAGGAACCGAGAAGAAGGTAGCAGCAACAACCGGTTTTATTATGGGACAGCTCATGATGTTCATATCGATCTATTATACGCCTCTGCATCTAGCATTGGGTAGACCTCATACAATAACTTTCCTAGCTCTACCCTATCTTTTGTTTAATTTCTTCTGGAGCAATCATTTTGATTATGGATCTACTACCAGAAATTCAATGCGTAATCTCAGCATTCAATGTGTATTCCTGAATAATCTCATTTTTCAATTATTCAACCATTTCGTTTTACCAAGTTCAATGTTAGCCAGATTAGTCAACATTTATATGTTTCGATGCAACAACAAGATGTTATTTGTAACAAGTAGTTTTGTTGGTTGGTTAATTGGTCACATTTTATTCATGAAATGTGTTGGCTTGGTATTAGTCTGGATACGGCAAAATTATTCTATTCGATCGATTATTCGATCTAATACGTACCTTCTTAATGTACTTATTCGAGCTATTAATGTACCTATTCGATCTAATAAGTACCTTCTTAATGTACTTATTCTATCTAATAAGTACCTTAAAGTAATACTTATTCGATCTAATAAGTACCTTAATGGAATTATTCGATCTAAGAGGTATAAGAAGTACAAGTACTCTGTGTCAGAATTTAAGTACCTTGTGTCAGAATTTAAGTACTTTGTGTTAGACTTGATAGATTCTATGGATCGAATCTTTAGTATTCTCTTATTTATTAGCTGTGTCTACTCTTTAGGCAGAATGCCGTCACCCATTTTTAGTAGGAAACTGCAAGAAACCTCAAAAACGACAGAAAGGGGGGAAAGTGAGGAAGAAAGAGATGTAGAAATAGAAACAACTTTCGAAACGAAGGGGACTAAACAGGAACAAGAGGGATTCACCGAAGAAGATCCTTCTCCTTCCCTTTTTTCGGAAGAAAGGGAGGATCCGGACAAAATCGATGAAACGGAAAGGATCCGAGTGAATGGAAAGGACAAAACAAAGGATGAATTCCACTTTCACTTAAAAGAAGATAAAGACCTCTTCTGGTTTGAAAAACCCCCTGTGAGTCTTCTTTTCGACTATAAACGATGGAATCGTCCATTGCGATATATAAAAAATTATCGATTCGAACATGCTGTAAGAAATGAAATGTCACAATATTTTTTTTATACATGTCAAAGTGATGGAAAACGAAGAATTTCTTTTACATATCCATCCAGTTTATCAGCTTTTTTTGAAATGCTACGACAAAAAATGTATTTTTATTTTTTTACAACAAAAAAATTCGTCTGTGATGAACTGGATAAATTCTTCTATGATGAACTGCATAATTATTATTGTTGGATTTATACCAATGAAAAAAAATGGAGGAGCCTAAGGAACGAGTTTACAGATAGAATTGAAGCCCTAGACAGAGGATCTCCTTATCTGGATGTACTCGAAAAAAAGACTCGATTATGCAATAATAAAACTAAAGAAGAATACTTGCCTAAAATATATGATCCTCTCTTAAACGGATCCTATCGTGGAATAATTAAGAAATTTTATTTACCTTCAATCCTAAATGAAACTGCAGTCAAAAATTCGATAGAGACAAATTTTATAAATAAACTCAATAAAGTTCATAGTATCCTTCTTTTTAAGGGTAAGGAACTTAATGTTCATAATTTTGAAGAATTGTACCAGAAATTGGAAGGGAAAATAGCTACATTGGAAGAGAAATTGGTCCAGAAATTGGACCAGAAATTGGAAGATAAATTGGGACAGAAAATATATACATTGGATAAAAAATCATTAGCAAGAGAATTGAGTCTTTTAATCGATGAATTTGCTGAAGAATCAACATCAAATTTGAAAGGAATTTCTTTATTTCCGGAACAAAGACGAATTGATTCAGAAGATCCAGAAAAAGTTTTGAAATTTTTAATCGAAAGAGTCATAATTGATCCCATCATTCAAACAATATGCGATACAGCCATAATTCCTCCCATGGAAAAAACAACTCGAAAAAAATCGATTGGAATAAATAAAAAAGTCCCCCGATGGTCATACAAATTATTCAGCGAGGTAGAACAACTCGGAAAAACTGCAACAACGGAAGAGGGGGAAGAGTGGATAGTAGATCATCAAATTCGCTCGAGGAAAGCGAAACGTATAGTTCTTTTTACGCAGGGTCCGGAGGAAGCAGAGAATGCCGACCCTAGTATAACTATGACGAAGCCTGATGAAATAGAAGAAGTGGATATGATAGATTATCCCTATGAAGCGGATTTTCGTCGAGACATAATCACAGGTTCTATGCGTGTTCAAAGACGTAAAACCCTTACGGGGAAAATGTTTCCCTTATATCCGTATTCCCCACTTTTTTTCGACAGAGTAGGATTTTCTTGGGATGTTCTTTTCGAACCATTCATATTATCAGTAATTGAGATTTCCGACCTAATACAAGACATTTTTAGAAAGGGTATAAAAGGAAGCGTAGCAAAAAGAATAAAGAGGTTGAAAAAACAAAAAAAAATGTACATGGAGGAAAACAAAAGCTACGAAGAAATTCAAAAAGAAGTCAATGAAATGGAAAAGGGGCGGGACGGGCAGACGGAAATGGAACGAATAGAAAGGACACGAGAAAGAATATCAGACCTCTATGATATCCTTATTTATGCTCATGGAATAAGAGCTTTTATTTTACTAATTCAGTCGAGGCTTAGACAATCTATTGTATTACCTTCATTGATACTAGCTAAAAATACTGTCCGTTTCTTATTACGCCAAGAGTCCGAGTGGGAGCAGGATATAAGGGAGATGAATAGAGAAGTGTATGTTATATGCACCTATAATGGTATGCCAGTACTAGAACCAGGAAGAAACGGAATTTTTCCTCAAAACTGGGCCACAGAGGGTATACAAATAATGATACGATTTCCTTTCCGTCTGAAACCTTGGCACCGATCTAAGATACGACCTTCTCGTAGGGATCCAAATCCAAAGCAGGAAAGTCCTGCTGCTTTTTTAACGATTTGGGGACTGGAAACTGACCGTCCTTTTGGTTCTCCTCTCGTAGGACTTGGTATTTTGTTTTGTTATTATTTTGGACCCCCTTTGAAAAAACTCCAAAAAGCAATTATAAAATGGAGTTTTCGAGTTCTAAAAAGTTTCAAAGAAAGAACAAAATTTTTGTTTCTAAAGGTCCAAAAAGAACCAAAAAAATTGAGAGAGGATTCGAGTGAAATAAAAAAAGATTCTATAATCAATAATCAGATTATTCATGAATCATCCATTCAAACCCGATCTATGGATTGGACAAATTATTCACTGACAGAAATAAAAATGAAAGATCTGACTGATAGAACAAGCACAATCAGAAATCAAATAGAAAGAATTACAAAAGACAAGAAAAATGGATTTCGAACTCTAAAGATAAATATTAGTCCTAACAAAACAAGTTATGGTGCTCAAAAATTAGCATCACTAAAAAAGATTTTTCAGATATTAAAAAGAAGAAATGATCGATTAATCCGTAAATCACATTATTTTATAAAATGGATCGTGGAAAGGATATACACGGATATCCTTCTAGAGAGCTTTCCATATATCATTAATCGTCTTACTAATAGTCTTTATAGGCCCATGATCATTATAAAACTTTTTCGTAAATTAAAAAAAAAATATATTTTTGATACAAAAGAGAAAAAGATAATTGAGCGTATTTCAACTATACAAAAAAAACTTTCACCTTCTCGTATTCGTCATAAGATTCAGACGAAGTCGGAGGTTTCTTTTAAATTATCCCTCGTGTCACAGGCCTATGTATTTTACAAATTATCACAAACCCAGGTTATTAACTTGTATAAGTTAAGATCTGTCCTTCAATATGACGGAGCATCTTTATTTCTTAAGAATGAAATAAAAGATTATTTTCGAAGACAAGGAATAATTTCTTCCGAATTAAAGCATAAGAAACTTCAGAATTCTGGAATGAATCAATGGAAAAATTGGTTAAAGAGTCATTATCCATACGATTTATCTGAGCTAAAATGGTCTAAATTAGTACCGCAAAAATGGCGAAATAGAGTCAATCAACATTGTAGGGTTGAAAATAAAAATTTAATCAAACGGGATTCATCTGAAAGAGAGGAAAAGGTTTCGTTATTGCTAAATAAAAACGATCATTTTCAAAAAATGTATAGATATGATCTTTTAGCATATCAATCGATTTATTATGAAGATAAGAAGGACTCATATAATTACAACATACATAAACCGAAATTAGTTGATATGGGGGGGAGTATCCCTATTACTAATTTTATAAGAAAAGATTATTTTATGTATATAAAAAATCCAGATAGAAAATATTTTGATACGAAAGGTCTTTTTTATCTCAAAATTAATAAAGATAAAGAAATCAACCCACCCAATCAAAAGGGTTTCTTTTCTTTTTTTGATTGGATGGGAATGAATGAAGAAAGACTAAATCGTCCTGTATCGAAGCCGATACCTTGGTTATTCCCACAATTTGAGTTATTTTTGAATGTATATAAAATGAAACCCGGGTTTATACCAATTCATTCACTTATTTTTCATTTTAATGAAGATGTTAGTCAAAATCAAAATATCACTAAAAATAAAAAAGGGGATCTTATACTATCAAATGAAAAAGAAAAAAAATCTTTTGAATTAGAGAATCAAGAAGAAAAAAAACCCATAGGTCAAAGAGATCTCGCATCAGATGCCCAAAACCGAGGGAACCCTGAATCTGTTCTCTCAAACAAAAAAAAATATATGGAAGAACTTTATACGAAATCAGATATGAAAATGGGTAGAACGAAAAATAAATCCAAAAGCATTTGGACTTGGGAAATAGACTTAGATGCGTTCATGAAAGGATCTTTTGCTTTGCAATTGAAATGGCTGCTGAGTCCTTTGACTATGGAATTATTCGATTATGCGCTGTCCGTACTTGAATGGGAAAAGGAAAGCAGAATGACAACAAAGTTTGGTTTCGGCTTTATTAAGAAGGAGGAGTTAACTCTGGATCCAATGCTAATCCGGGATTTGAATCTTTCAAAAATCCTAAAAGAGGGAATATTTATTATCGAACCGGTTCGTATACCTGTAAAACATAATGTAGAATTTATTATGTATCAAACCATAAGGATTTCTTTGGTTCATGAGATTAAACAAAAAAATAATCAAAAAAGATACAGAGAAAATATGGGTAAGAATCATTTTGAGGAATCGATTGCAAGACATCAAATGATGACGAAAAATAGAAACAAAAATCATTATGATTTGCTTGTTCCTGAAAATATTTTATCGTCTAGACGGCGTAGAGAATTGAGAATTCTAAGTTGTTTCAATTCAAGGAATAGTAATTGTGTGGATAAAAATGCAGTATTTTGCAATGGGAACAAGGTAAAAACCTGTGGTCAATTTTTGGATGAAAGCAAAGATCTTGATAGAGATAAAATTAAATTAATTAAATTAAAATTCTTTCTTTGGCCCAATTCTCGATTAGAAGATTTAGCTTGTATGAATCGCTATTGGTTTGATACTAATAATGGTAGTCGTTTCAGTATGTTAAGGATACATATGTATCCACGATTGAAAATTGATTGATGATACAATTGTCTTATATATCCCCTACCATACATATCGGGTGGATAAATAGCTGCGCACATGCCTTGTCTTACATCCTTGTTTGATACATGAATACTAATTCAATGACGTATCAATTAGATCATAAAATGAATCAATAAGGAAATTCGGATTGATTATTGTGTATACCAGATCAAAATACCTCGCATTATTATTACTGATCAGTAAAATTCATATTCGTAAAATAAGAATAGTAAATTAATAAAAAAATTGACGCATAGAATATATACAAAAAAAGATAAGAAGAAATGCGCCCCCCACCTACATACTTGATACCTTCTCCTACAAAAAAACTTGTAACACCAAATCCATTTGGAATTCCATCAATTACTCGTCTGTCAAAAAAATGAACTAGTTCGGACAATCCTCTTACACTCCGAATTACGTATGTTGTATAAAAAGCATCTATGTAACCACGATGATGGGCCCAATCATATATTACATTTTGAATTTTGTCCGAAAAAAACCTCTTTGGATGCTTTTTGGCAAAAAAATTAATTAAGTAAAAATTTTGTAACGATGAATAAATGGGTTTATATAAAAAAGACGCTATAACTATTCCAGAATAAGCTATACTAACCGACAACGTTGCATTTGTCACAAATTCAGACCAATCAAAAAAATTTTGATTATTCAATTTTGGATGTAAAAGGTTTATAGATGGAGTTAACCATTTGGACAATATATCCAAATCTATGCCTTCTTGATTCAAAGGAATTCCTATGAATCCAATGAACAAAGTAAAAAAAATCAATACAAGTAGCGGGACTAACATAGTATTCCCCGATTCGTGAGGATATGGCGAAATTTTTTTATTGTCACAATTATTAATAATAAAGGATCGCATCATTTTTTTTATATTTCCGTGTGAATTCTTAGAAACCCTTTCATTATTTTTTATTGTTAACAAAGTTAATAAATGAAAATTTTTGTTAATAGGTTTCATTCCTTCTTGACCCCATAAGGATATTGAATAGAAGGAGCTACTTTTTTTTCCATTGTAATTTTGAAAATTAACGTTTAAATGACCTTCAAACGTAAGTAAATAGACGCGAAACATATAAAATGCAGTTAATCCTGCTGTGGCACAGGATATGATTGCGAAAATTGGTGAATACAACCAAGTATCATTAAGAATTTCATCTTTGGACCAAAAACAAGCAAGAGGTGGAATACCAGAAAGAGAAAGTGTACCTAATAAAAAAGCGGTTTTTGTAATTGGCACGTGTTTTTTTAACCCCCCCATAAAAACCATATTCTGACTTTTATCTGGAGAATATCCAACAATAGCTTCCATAGAATGAATAATGGATCCCGATCCTAAAAACAATAATGCTTTTGAGTAAGCATGAGTAATCAAATGAAATAAAGCAGCTCGATAAGACCCCATACCTAGAGCTAACATCATATACCCCAATTGAGACATTGTAGAATAAGCTAAACTTCTCTTAATGTCTTTTTGAGCAAGAGCTAAAGTAGCTCCTAATAGTACTGTTATTATACCTATTAAAGCGATTAGATTTAGTATGGAGGGGATGACTATCAAAAGAGGAAAAAGTCGAGCGACAAGAAAAATCCCCGCTGCTACCATAGTAGCAGCATGTATAAGAGCCGAAATAGGAGTAGGCCCCTCCATAGCATCAGGTAACCATACATGAAGGGGAAATTGGGCGGATTTGGCAACTGCACCAGCAAATAATAAGAAAGTACATACAGTTCCAACTAAAAGATGGACTTCATTAGTATAAGTCAAAGTATTGAATATTTTGAACAAATCTCGAAATTCGAAACTGCCCGTTAGCCAATAAAGACCTAAAATTCCTAATAATAAACCAAAATCCCCTACACGATTAGTCACAAACGCTTTTTGACAAGCATTTGCTGCAATAGGTCGTGTAAACCAAAAACCTATTAATAGATACGAACACATTCCAACTAATTCCCAAAAAATATAAATTTGTATTAAATTCGAACTAGTAACTAAGCCAAGCATAGAAGTATTGAAAAAACTCAGATAAGCAAAAAATCTCAAATATCCTTGATCATGAGACATATAATTGTCACTATAAATAAGAACTAGAATCCCAACAGTAGTGATTAACATTGACATAATAGAAGTAAGTGGATCAACCAAGTAACCGAACTCTAAAGAAAAATCATTATTGATGGTCCAAGACCATACAAATTGATAGATAGAACTGCTATTTATTTGCTGAATAGACAATTTCATTGAAAAAATCATAACTATACTTAACAACGAAATACTAGGAAAAGCCCACATACGCCGAAGATTTTTTGTTGTCGTCGGAAAAAGAAGAAGTCCCGCTCCGATTAACAAAGGAACTGTAAGTGGAATAAAAGGTATGATCCATGCATATTGGTATATATGTTCCATAAAAAATAAAATTTAATTTTCGATTCGCCGGCTCTTACCTCTTTCGAAAGAGGTCAATAAAAAAATTAAGATATGCAATAATAGAATTTTCAAATTTTCTATTCGAAATTCTTAGAATAAGAATAAGCATTTTATTAATATTCAACTCAAGAAGTTCTAATTGGTCAAATGACCAAGTAGTTATTAGTGAAAGTAAATACTTAGTAGTAGTTATTAGTGAAAGTACATACTTAGTTATTAACTAAACTATTGAAACCTATGAATATAGAGAATATCGAAAATTTATATTTTCCATTATTATTTTGATAAATGCATAGATGGAAAAATGATAAAAAATGAAACCAAAAAGTATTTAAGTCTGATACTGATATGAATCACAAGATCTACTAAAATTCATAACATAATTTGAGCCAAAAACTAGGAACTATTTAAATTAGTTTATTCGGAATCAGTTATTAGTTCTCCGCGAAATTCTTTGATTGATTGTCTTCTATTCCAATCCAATAAAGCATATTTCTCTTTTTCTATGCTAGACAAGACTTTACTTTCGACTTTACATAAGATAAAATAAAAAAAAAATGATGAAAACATATCAAATCATGTCTACTTTACTAAATAACAAGTCTCATTTCAATTTTTAAATAATAATAAAAGAAAAAAATGCCATGTATTTGTTAAAAGGAGTGAAGTTTTTATATTAAGTAATTAAGTAAGAGTAGCTTGACTCTTCTAAAATGAAACCCTTCGGCGTGTTTATTACATAACATGTAAATACAATATTAAATACAATAAAAATCACCTAGCAAAAATAGACATAAATAGAAGTCTAAAGTTTGCTTCTTGATTTTATTTTTTACGGCCTACCGTAAAAAATAAATAGAAATTTTAATAAGAAAAATGGGAGTCTCTCATAATATGTAATTTTAATATATTTCGAGTTTTTGAGATTTTTTATAAAAAAAACGTAGAATAGAAAAACGTCTATAACTAAATAAAAAAAGGACAGAAAGATTCCTTTGAACAATAGATGTCTTTCACATCCAACTATAACAATGAATAACCTATTTTTTTTTTGAATGGCAGTTCCAAAAAAGCGTACTTCAATTTCCAAAAAGCGTATTCGTAAAAATATTTGGAAAAGAAAAGGATATTCGGCAGCATTAAAAGCTTTTTCATTAGCGAAATCTCTTTCTACCGGCAATTCAAAAAGTTTTTTTATACGAAAAATAAGTAATCAAATGTTAGAATAATCTGAATTGATCTGACTCAAAAAAACTTCTACAAAATTTAATTTAGCATGTATATAAGCATTTATATTCATAAAATAAATAAAAAAATTTAAAATAAAAAAAAATCAATCATTTAAATATAGATATAGAATTAATGATTTGTACTCATTAATATTTTATTGGAACTGATCAACATAAAATAGAACTTGCTTCTCTCTTATGATATGTAGACTAAAAATACGTCTGTATACTCTAAAATGGTAGTTTTTTGTTTGAAAACTAGAAGATTTCACTAACCTTCTTTTTTTTAGTATATTTTCTCATTTCTGGGATGGGGATTCTTACTTTCCCCATCAACCGACTGGTCACAATATTAAATTAAAGGTTGTTATATCTATGGAAGAGAAAACTAAAAATATTTAGTCAAAAAAAAACAAGGGATCCTTAAAAGAATGGATTCAATTAAAAATTTTCCCTCTCTGATTCATTATGTCTCTGAATTGTTATATATCTCCTGGTTTTATTAAAAAAAAATCTTATGATTTTGATAATTTCTATTTATTATTTATGCTTTATATTTATTATTTATATGTTACATGTATTTTGTTCGATTTTATTCTATTTTTTTAGTTTCCATTTGAAATTAAATTTTATAAATTCAAATGAAATTTAGGGGATATTATGTAGGAAGAATTTCGCTTGGGCAATTCAAATATATCTTTAGAACTTTATTTACACTAAAGTGCTATTGTATACATTCATATATTCCCTTTCCTTAGAAAAGAAAATTTTTAGGTATGTTTTCGATTTATATCATTTTTTTACTATATGACATGTCTGGTTCAATATTTAATCGGTTTGTTGAAATTATGATCAACAAAAATCCTATTTTTTTTTTTCATTGATAAGATAAATACATTTGGATAAGATAAATACATTTGTTTATTTCATATTTTTTAAATCAGATAAACAGATAAAAAATGAGTTATTACTACATTAAGATAAGAAAAAACTTTGAGTTCCATCTATGGTTTGAAGGCACATTTGTATAGTTACAAGAGTTCAATTTCAAGAAAACAGAAACTATACGAAATCTTATTGACTATAACTGAGACTATAAAGGAAAACCCTAATAAGGTAAGGATTATTATTGAACATTCAAATTACGATATTGAACGTTACGATTTGAACGACTTTTTTTTATTCCTCAAATTAAAAAAAAATTATGTTTTATAAAAATATCGCCATTGAATTGACTCTTTCAATCTCGACGATTAAAGATAAATAGGCTATTATGATTTCAAACAAGCCGCTATGGTGAAATTGGTAGACACGCTGCTCTTAGGAAGCAGTGCTAGAGCATCTCGGTTCGAGTCCGAGTAGCGGCACAGCATCTTAGAAATTCCAAAAAAGAATCTAATAGTCCTAGAATGAATAATAATCACAATGAGATGAATTTCATTCTTGATTTCTATTTCGTAATTTGTAATTGAGGGATCTCTTTTCTTTTTTTTTATATATATATATTCTTATGATATTTTTTACTTTAGAGCATCTTTTCGATCATATTTCCTTTTCGATCGTTTCAATTGTAATTACAATTCATTTACTAACTTTAGTAGTCAACGAAATAGTAGAACTAGATGATTCGTTAGAAAAGGGTATGATACTTACTTTTTTCTGTATAACAGGATTATTAGGCATTCGTTGGATTTATTCGGGGCATTTCCCCTTAAGTGATTTATATGAATCATTAATCTTCCTTTCGTGGAGTTTTTATATTATTCATATGATTCCTTATTTTAAAAAACATAAAAATCATTTAAGTGCAATAACGGCGCCCGGTGCTATTTTTACCCAAGGCTTTGCTACTTCGGGCTTTTTAACTCAAATGCAGCAATCCACAATATTAGTACCCGCTCTCCAATCCCAATGGTTAATGATGCATGTAAGTATGATGATATTGGCCTATGCAGCCCTTTTATGTGGATCATTATTATCAGTAGCCCTTCTAGTCATTACATTTCAAAACAATATAAGTATTTTTGGTAAAAAAAAACTTTTATTAAATGAGTCTTTGTTCTTCGGGAAGATCCAATACATGAACAAAGAAAACAATATTTTACAAAGCACTTATCTCTTTTCTCTTAGAAATTATTATAGGTCCCAGTTAATTGAACAATTAGATCATTGGAGTTCCCGTGTTATTAGTCTAGGATTTATCTTTTTAACCATAGGTATCCTTTCAGGAGCAGTATGGGCTAATGAAGCATGGGGATCATATTGGAATTGGGACCCAAAGGAAACGTGGGCATTTATTACTTGGACCATATTCGCGATTTATTTACATATTAAAACAAATATAAATTTGCAAAGTGAAAATTCTGCAATTGTGGCTTCTATAGGATTTCTTATAATTTGGATATGCTATTTTGGGGTCAATCTATTAGGAATAGGACTACATAGTTATGGTTCATTTATATTAAAAAGCACCTAAATTTAATTCAAGAAAGGACCTAACCTGACGAATACAACCACAGGACAGGGTATATCCCATATATCGAAATAAGCAAGCCTCGCCGAGAACCATTTCAATCAAGTAGTATAGTGATTCAAATGGTTCTCACAAACGTCAAACTATCCGATTATAATTAAAATTCGTTTTTTGCGTTACGTAAAAAAGACTTTTTTGAAATGAAAACTATCTATAAAAAAAATTAGATAGAATAGCTTCTACCTTCTCAACTGATAGTGAGAGAACGAAATCTGGGTAAATGCCAATACCTATTACTGGTAGAAAGATAGCGAGTGAAACAAATAACTCTCGTGGACCAGAATCAAAAAAATAAGAGTTTGCACTATTAAATAACTTGTATCCATAGAACATTTGGCGTAACATGGATAATAAATAAATAGGAGTTAATATCATTCCAATTGCCATACCAAAAGTAATTAGTACTTTTGACATTAAAAAATATTTTTGGCTGGTAATTATTCCAAAAAATACTATTAATTCGGCAAAAAAACCACTCATGCCCGGTAACGCAAGGGAAGCCATCGAAAAAGTACTGAAGAGTGTGAATATTTTTGGCATTGAAATGGCTATTCCGCCAATTTCGTCGAGATAAACAAGACGTATTCTATCATAACTCGTTCCCGCTAGGAAAAAAAGCGCAGCACCAATAAATCCATGAGAGATTATTTGTAAAATGGCTCCGTTGAATCCCGTATCGGTTATAGAACTAATTCCTATAATTATGAAACCCATATGAGACACAGAGGAATATGCTATTCTTTTTTTTAAATTACGTTGCCCCGGAGATGCTGAAGCTGCATAGATTATTTGTATTGTGCCTACTATCATCAACCAAGGAGAAAATATAGAATGAGCGTGAGGTAATAATTCCATATTGATCCGAACCAATCCATATGCTCCCATTTTTAATAGGATTCCAGCTAGAAGCATACAAGTACTGTAATGTGCTTCTCCATGGGTATCCGGTAACCATGTATGTAGGGGTATAATCGGCGATTTGACAGCAAAAGCAATAAGAAATCCAATATAGAATATTATTTCTAATCCCACAGGATACGATTGATTAGCTAACGTTTCAAAATTTAATGTTGGTTCATTGGAACCATATAACCCAATACCCAAAACTCCCAGTAACAGAAAAACGGAACCCCCTGCAGTGTACAAAATAAACTTTGTAGCTGAGTATAGACGTTTCTTTCCTCCCCATATGGATAAAAGTAGATAAACGGGAATTAATTCTAATTCCCACATTATAAAAAAAAGTAAAAGGTCCCGAGAAGAAAATAATCCTATTTGACCACTATACATTGCTAACATCAAGAAATGGAATAATCGGGAATCTCGAGTAACTGGCCACGCCGCTAAAGTAGCTAAAGTCGTGATGAATCCCGTCAGTAAAATAGGTCCTATAGAAAGCCCGTCTATTCCCAATCTCCAGTGGAAATCAAAAAAGTGAATCCATTTATAATCTTCTGCCAGTTGTATTAATGGATCGTCTGGTTGGAAATGATAACAGAATACATAGGTTGTTAGGAGGAATTCTAATATACATATACACATAGTATACCATCGAATTACCTTATTACCCCTATGAGGGAGAAAGAAAATTAATGAACCCGCAAATATAGGCAAAACTACAATTAATGTTAACCAAGGAAAATAATTCGTGGTAAAGACAAAATACACTTGGACTAAAAAACCCGTACTCGAATAAGAACAAAATAAGATATATATATTTCATTTCGAGCGCGGGTTTTTGTCGGTAAACAAAAATCAAATGGATTCAAGTGGAGTTTTCTGGAACGTATCAATAAGCTAGACCCATACTGCGAGTTGTTTCATGCCATAAATAAACTCGAACACTCAAAAAATCGGTTGGACAGGCGGATTCACATCTCTTACAACCAACACAGTCCTCTGTTCTTGGGGCGGAAGCTATTTGCTTAGCTTTACATCCGTCCCAAGGTATCATTTCTAATACATCTGTGGGGCAGGCTCGGACACATTGAGTACATCCTATACATGTATCATAAATCTTTACTGAATGGGACATTGGATCTATACCTTTTTTTGAATCTCATAAAATTTCGATCTAGTATAACCCTATATTGTATGTAAATGAATTACATATTCAAAGACCAGACGAATCGATGATTCACCAGAATTTGTTGAATCAACTTATTTCTGGGTCGGTTTAGAAAAGAGGTCCAAAATACTTTGATTTCTTACATTTTTGAAGATTCTACATACCTAGTAATCTAATTTGAATTGATAACTATTCAAATTTCTATAATAATAATATTAATACTACTTATTCAACAAATTCGATTGATTAATACGAGTTGATTTTCTGTTACGATAAATTGCCGAAACAATAGCCGGTCCAATAGCTGCTTCAGCGGCTGCAATAGCTATAACAAAAATGGAGAAAATGTTTCCTTTTAATTGACGACTATCAAAAAAGTCAGAAAATGTTACGAAATTAAGATTAACCGCATTCAATATAAGTTCAAGACACATAAGAGCTCTAACTAAATTTCGGCTTGTGATTAATCCATAGATACCAATAGAAAATAAATAGGCACTCAAAACAAGTACATGTTCGAGCATCATTGAACAACTCCTTATCAATCTTCATTTATTTCATTTCAATATGAACAATAATTAAAGCGATTTCGTTGACTCGAATATAACAAATACAGAGCAAAGGAGTATGTTAGTAATAGATTGACATTTATATTTTATATTATACATCAATTCAAATGGAATTGAATGGAAATGGATACGATAAAACAGAATAAAGTTTGATTTGGAGTGATGCTTTTAAAGATTTATTATTTTATTGACGGGCCACGGCAATTGCACCTATCAAAGCAACTAAAAGAATTATCGAAATGAGTTCAAATGGGAGAAAAAAATCTGTTGATAAATGAATTCCAATTTGTTGACTATTATTTATCAAATCTTGTTCTATAATCTGGTTTAATCTTGTAGTCCAAATAATTCCGTACCATGACGTATTTAGAATAGTAGTAATTAATAAAACAAAAATACTGGTACAAACTAACAAAGTAATTCCGTCCCCAAGAGTCCAAAGACGAAAATCTTTGTCATATTCTAACCCGTTGATAAACATTACAGCAAATATGATTAAAACATTTATAGCTCCCACGTAAATAAGGAGTTGTGCAGAAGCTACAAACTGAGAGTTTGCTAGAATATAGAATAAAGATATACAAACAAGAACCAATCCCAACGAAAAGGCAGAAAAAATGGGATTGGTAAATAATATCACTCCTAGGCCTCCTAATATAAGACCTGATCCCAGAAAGACTAAAAGAAAATCATGTATTGGTCCAGGTAAATCCATTCGATGAAAAAAAAAGATATAATAAATCGGACTCTTTCATGATCTTATTGAACTGACCAGGAAAAAATAAGTTAAGTTGATCTATTTAGGACACGTTCCTAGTTGAATGCAATTCTAATGGATGCGAATTTATGTAGGTACAGTTAGTGGACTAATCACATTCTTTATCTAAAAGGCTAATTCGAATCTAGTTGAAATCATTACATTAAAAAAAAATTTCCAAGTAAATCTGCAATTTTCATGAACCAATCAGATCAATAGTTGTTATTTTTAGGTTAGTTATTCACAAAAAAAAAGAAACCTGTTAAATTTGGATAACAACCTTAGTAATAAAAAAAATACCGTTCTTGAATCAAGGTATTTCTTTTTTATTGAATTGAGGCCAATTCAAGATAGTTCGAATTGTATAATCGTCAATTATTGATATTGGTAAACGGCCTAAAGCAATTTGATTATAATTTAATTCATGACGATCATACGTAGAAAGCTCATATTCTTCAGTCATTGATAAACAATTTGTTGGGCAATACTCAACGCAATTACCACAAAATATACAGATTCCGAAATCAATACTGTAATTAAGCAATCGTTTCTTTCGAATATCAGTTTCTAATTTCCAATCTACCACAGGTAGATCTATAGGACATACACGAACACATACTTCACAAGCAATGCATTTATCGAATTCAAAGTGGATTCGACCACGAAAACGTTCTGATGTGATCAATTTTTCATAAGGGTATTGAATAGTTACAGGTAAACGATTCGCATGGGATAAGGTAATTAGAAAACCTTGACCAATGTACCTTGCCGCTCGTACTGTTTGTTGACCATAATTCAGGAACCCAGTTACCATAGGGAACATATCCTAAATATCGATAAAAAATATTTTGTTTGTTTCTTTCTCTTGTTTGGGACAAGTTATCAATCTAGTTACTAGTGAATAGAAAATATTCTATTTTGCTTATAGTGAAAGGAGTTGGGAAGAAGTTGTTAATAATAAATTACCTAAAGAAATAGGTAAAAGAAATTTCCATCCAAGATTTAATAATTGGTCCATTCTCAGTCTAGGTAACGTCCATCTTGTTGTGATAGAAATGAACAAGAACAAAAAAGTTTTCGCTAATGTAATGAAAAGACCAATTGTTGTTCCAAAGACTCCATCCCTTGCATTTATTTCAAAAAGGTCAGGAACGAATATGTACGGAATAGATAAATTCCAGCCTCCCAAGTAAAGAACTGTTACAAATAATGAAGAAACTAGTAGATTTAGATAGGAAGCAACATAAAATAAACCAAATTTAATACCTGAATATTCTGTTTGATAACCTGCTACTAATTCTTCCTCTGCTTCTGGTAAATCAAAAGGCAATCTTTCACATTCGGCTAGAGAAGAAATTAGAAAAACGAGAAATCCTATAGGCTGCCGCCACAAATTCCACCCCCAAAAACCGTATTTGGACTGTGCCTCAACTATATCAACTGTACTTGAACTGTTAGATAATTCTAGTCGGTGATAAGATCACAGTTATCATCGCTATTACAGAACCGTACATGAGATTTTCACCTCATACGGCTCCTCGAGGGCCCCATATAAATCTAAGGACTGCTTCGATATTCTTTAATCTTGATATTTTTGTAGGATAGATAGAGTCAAAATCAATCGAAAGGTCCCGAATTAGACCAATGGAATTCTGTCTGCGATACTAGATATAAGGGCTTCTGAATTGATCTCATCCTTTACTTTTTTTTAATTAAATTTAAATTAATATTTTTTTTTTCATTTTATTAATTTTCAGTTTTCTTTATTTAGAATTAATTTAAACCTTCAGAAAAAACTCTTTTTAGAAATATTAATAGATATCTCACCCTATCCTTTTTTAGTACGAAAAGAAATTAACATGAAGCATGACATGAAGTGTAGCTCTCTTAATACAGCTATAGGAAAGCAAGAATACTAAAAAATTTTTGCAATTAAATTCTTTCTATTTTTTTCTTCCTTTTTTTTATAAAAATAAAAATAAAATAGAAGTAAAGTAAAGGATTACTTCGTTCCTGATAGTCATTCACTTAATCGGTGGATAGGAGCATACTCTGGATCGGAATTCTGGGGAGTACTACTTGATCATTTCTACAAATTTAAAGCCCCAATTAGTATTTCGTTTATGTGGAATTCTTTTTCCGATAACTTAGAAAATCTCTATTACTAATCCTTTGTGTACCTTGGTGTTCCTAACCATCCACTCAGTTTTGCTCAATCTCTGCGGTAATTCGTGTCATGTATAGTAATACATACAAACGATAGCACGAACTCCAAAGAGTGGATCTGTTTAACCCGCTTCAAGCCATGATAACTAATCAACCAGTCTTGGGGTAAATTGTTTTTCTTTATAGTTTTTCTTTACTGCTTCTATTTACTTTGGCGTAATTCTTGTACATAGGAAATGAGACTCAATCTTTTTACTGCGAATTTCGAAGCTGTTTTCTTTCACTCATCTAACTATCTGGTTTAGTTCATCAACCCGAAGGTTGAATAAAAAAGAAAGTTTATTTAAGTTCATTCTTAGAAAACTCTAGAAAGAAAAGAATTTTTGGAAAATTTATGCCTCAACGAATCACACGTAGAGATATTGATAACACACATAGAGTTAATGGTATTTCATAACTAATAGATTGGGCAGCAGCCCGTAGACCGCCTAAAAAGGAATATTTATTATTTGATCCATATCCTGACATAAGAAGTCCAATGGGAGCAATACTTGAAATGGCGATCCATAAAAAAACACCATTACTGAGATCGACTAGAATAAGTCGATAGCTAAAAGGAATTACTGAATAACTTAGTAGAATTGATATGACTGCTATGGAGGGTCCAACACTAAATAAACCCCTATCTCCTCTTGATGGAAGAAGGTTCTCTTTGAAAAGTAGTTTTGTTCCATCTGCTAGAGCTTGAAGAATTCCCAAGGGGCCGGCATATTCAGGTCCAATACGTTGTTGTATCCCTGCGGATATTTCTCTTTCTAACCACACAATTACTAGTACACCTATTGTGATTCCCAAAACAAGAATCAAAATAGGTACAAGCATCCATATGGTCCCATAGACTTCTTTTAAGGATTCCAATAGAGAAAAAGAATTGATAGCCTGTACTCCTGTTGTATCAATTATCATTTCAACGATCAATTTCCCCCATAATGATATCTATGCTACCTAGTATTGTCATAATATCAGCCAATTTCATTCTTTTAACTAACTGAGGAAGAATTTGCAAATTGATAAAACCCGGCGGGCGAATTTTCCATCTCCATGGAAAAGCACTCTGATCTCCTATCAAATAAATTCCCAATTCGCCCTTTGGCGCTTCGACTCTTACATAAAGTTCTTGTTTCGATAACTCATACGTGGGAGACGGCTTTTTACTAACGAATCGATATTCAAAATTATTCCACTCAGGATCTCTTACTCTATTAAAGCGTCGGATTTCTAAATTCTCATAGGGGCCCCCCGGAATTCCTTCCAGAGCTTGCTGAATAATTTTTACAGATTCCACCATTTCGCCAATTCGGATTAAATAACGAGCTAATGAATCGCCTTCCTTCTGCCATTGGACTTCCCAATCAAATTCTTCATAACATTCATAATGATCAACTTTACGAAGATCCCATTGTATTCCGGAAGCCCGTAGCATTGGTCCTGACAAACCCCAATTTATTGCCTCTTCTCCGCCAATAATGCCCACCCCTTCAACTCGTTCTAAAAAAATAGGATTTCGTGTAATAAGCTTTTGATATTCAGCAATTGCTGTTAAAAAATACTCACAGAAATCCAAACATTTATCTATCCAACCGTAAGGTAGATCGGCAGCGACTCCTCCGATACGAAAATAATTATGCATCATTCTCATACCAGTAGCAGCTTCGAATAGATCATATATTAGTTCTCTTTCTCTGAAAATGTAGAAGAAGGGGGTCTGTGCGCCAATATCCGCCATAAAAGGACCAAGCCATAACAAATGAGAAGCTATACGACTCAACTCCAACATAATAACTCGAATATAGCTAGCCCTTTTAGGTACTTGAATATTTCCTAATTGTTCTGGTGCATTTATAGTTATTGCTTCTGTAAACATAGTAGCTAAATAATCCCAACGTGTTACATAAGGCAGATATTGTATAATTGTTCGGTTTTCCGCAATTTTTTCCATTCCTCTGTGCAAATAACCTAAAACGGGTTCACAGTCAATAACATCTTCGCCATCTAAAGTAACAATGAGTCGAAGAACGCCATGCATTGATGGGTGGTGAGGCCCCATATTGACTATCATTAGATCTTTTCTTGTAACTGGTTCAGTCATAAGTTTTTTCCGTATTTCTTCTTCCATGAATTGCTGAAAACGAAAAGAAGTTCATCAAAATTGAAGATCGAATAAATCAAAGAAAATAATTGTTCAAATTAACGTTTTTTTATCTCTCGAATATTCAATTGACTGATTAATTCTTTATAAAGTACTCTATTTTTTTTTGAAAAATAAGCCAGCAGTCGTTGACGTTTTCCCAAAATTTTCCGTAGACCTCTCTGAGATGAATAGTCTTTTTTGTGTAATTCCAAATGTGAGCTAAGTCTCCGTATCTTATTGGTGAAACAGAATACTTGAAATTCAACAGACCCCTTCTTTTCTTCTTTTTCTTCTTGCGAAATAACTGATATGAATGAATTTTTTGTCATAAGTATATAAATCCATAGAATATATATATATTCTATGCGTCAATTTTTTTATTAATTTACTATTCTTATTTTACGAATATGAATTTTACTGATCAGTAATAATAATGCGAGGTATTTTGATCTGGTATACACAATAATCAATCCGAATTTCCTTATTGATTCATTTTATGATCTAATTGATACGTCATTGAATTAGTATTCATGTATCAAACAAGGATGTAAGACAAGGCATGTGCGCAGCTATTTATCCACCCGATATGTATGGTAGGGGATATATAAGACAATTGTATCATCAATCAATTTTCAATCGTGGATACATATGTATCCTTAACATACTGAAACGACTACCATTATTAGTATCAAACCAATAGCGATTCATACAAGCTAAATCTTCTAATCGAGAATTGGGCCAAAGAAAGAATTTTAATTTAATTAATTTAATTTTATCTCTATCAAGATCTTTGCTTTCATCCAAAAATTGACCACAGGTTTTTACCTTGTTCCCATTGCAAAATACTGCATTTTTATCCACACAATTACTATTCCTTGAATTGAAACAACTTAGAATTCTCAATTCTCTACGCCGTCTAGACGATAAAATATTTTCAGGAACAAGCAAATCATAATGATTTTTGTTTCTATTTTTCGTCATCATTTGATGTCTTGCAATCGATTCCTCAAAATGATTCTTACCCATATTTTCTCTGTATCTTTTTTGATTATTTTTTTGTTTAATCTCATGAACCAAAGAAATCCTTATGGTTTGATACATAATAAATTCTACATTATGTTTTACAGGTATACGAACCGGTTCGATAATAAATATTCCCTCTTTTAGGATTTTTGAAAGATTCAAATCCCGGATTAGCATTGGATCCAGAGTTAACTCCTCCTTCTTAATAAAGCCGAAACCAAACTTTGTTGTCATTCTGCTTTCCTTTTCCCATTCAAGTACGGACAGCGCATAATCGAATAATTCCATAGTCAAAGGACTCAGCAGCCATTTCAATTGCAAAGCAAAAGATCCTTTCATGAACGCATCTAAGTCTATTTCCCAAGTCCAAATGCTTTTGGATTTATTTTTCGTTCTACCCATTTTCATATCTGATTTCGTATAAAGTTCTTCCATATATTTTTTTTTGTTTGAGAGAACAGATTCAGGGTTCCCTCGGTTTTGGGCATCTGATGCGAGATCTCTTTGACCTATGGGTTTTTTTTCTTCTTGATTCTCTAATTCAAAAGATTTTTTTTCTTTTTCATTTGATAGTATAAGATCCCCTTTTTTATTTTTAGTGATATTTTGATTTTGACTAACATCTTCATTAAAATGAAAAATAAGTGAATGAATTGGTATAAACCCGGGTTTCATTTTATATACATTCAAAAATAACTCAAATTGTGGGAATAACCAAGGTATCGGCTTCGATACAGGACGATTTAGTCTTTCTTCATTCATTCCCATCCAATCAAAAAAAGAAAAGAAACCCTTTTGATTGGGTGGGTTGATTTCTTTATCTTTATTAATTTTGAGATAAAAAAGACCTTTCGTATCAAAATATTTTCTATCTGGATTTTTTATATACATAAAATAATCTTTTCTTATAAAATTAGTAATAGGGATACTCCCCCCCATATCAACTAATTTCGGTTTATGTATGTTGTAATTATATGAGTCCTTCTTATCTTCATAATAAATCGATTGATATGCTAAAAGATCATATCTATACATTTTTTGAAAATGATCGTTTTTATTTAGCAATAACGAAACCTTTTCCTCTCTTTCAGATGAATCCCGTTTGATTAAATTTTTATTTTCAACCCTACAATGTTGATTGACTCTATTTCGCCATTTTTGCGGTACTAATTTAGACCATTTTAGCTCAGATAAATCGTATGGATAATGACTCTTTAACCAATTTTTCCATTGATTCATTCCAGAATTCTGAAGTTTCTTATGCTTTAATTCGGAAGAAATTATTCCTTGTCTTCGAAAATAATCTTTTATTTCATTCTTAAGAAATAAAGATGCTCCGTCATATTGAAGGACAGATCTTAACTTATACAAGTTAATAACCTGGGTTTGTGATAATTTGTAAAATACATAGGCCTGTGACACGAGGGATAATTTAAAAGAAACCTCCGACTTCGTCTGAATCTTATGACGAATACGAGAAGGTGAAAGTTTTTTTTGTATAGTTGAAATACGCTCAATTATCTTTTTCTCTTTTGTATCAAAAATATATTTTTTTTTTAATTTACGAAAAAGTTTTATAATGATCATGGGCCTATAAAGACTATTAGTAAGACGATTAATGATATATGGAAAGCTCTCTAGAAGGATATCCGTGTATATCCTTTCCACGATCCATTTTATAAAATAATGTGATTTACGGATTAATCGATCATTTCTTCTTTTTAATATCTGAAAAATCTTTTTTAGTGATGCTAATTTTTGAGCACCATAACTTGTTTTGTTAGGACTAATATTTATCTTTAGAGTTCGAAATCCATTTTTCTTGTCTTTTGTAATTCTTTCTATTTGATTTCTGATTGTGCTTGTTCTATCAGTCAGATCTTTCATTTTTATTTCTGTCAGTGAATAATTTGTCCAATCCATAGATCGGGTTTGAATGGATGATTCATGAATAATCTGATTATTGATTATAGAATCTTTTTTTATTTCACTCGAATCCTCTCTCAATTTTTTTGGTTCTTTTTGGACCTTTAGAAACAAAAATTTTGTTCTTTCTTTGAAACTTTTTAGAACTCGAAAACTCCATTTTATAATTGCTTTTTGGAGTTTTTTCAAAGGGGGTCCAAAATAATAACAAAACAAAATACCAAGTCCTACGAGAGGAGAACCAAAAGGACGGTCAGTTTCCAGTCCCCAAATCGTTAAAAAAGCAGCAGGACTTTCCTGCTTTGGATTTGGATCCCTACGAGAAGGTCGTATCTTAGATCGGTGCCAAGGTTTCAGACGGAAAGGAAATCGTATCATTATTTGTATACCCTCTGTGGCCCAGTTTTGAGGAAAAATTCCGTTTCTTCCTGGTTCTAGTACTGGCATACCATTATAGGTGCATATAACATACACTTCTCTATTCATCTCCCTTATATCCTGCTCCCACTCGGACTCTTGGCGTAATAAGAAACGGACAGTATTTTTAGCTAGTATCAATGAAGGTAATACAATAGATTGTCTAAGCCTCGACTGAATTAGTAAAATAAAAGCTCTTATTCCATGAGCATAAATAAGGATATCATAGAGGTCTGATATTCTTTCTCGTGTCCTTTCTATTCGTTCCATTTCCGTCTGCCCGTCCCGCCCCTTTTCCATTTCATTGACTTCTTTTTGAATTTCTTCGTAGCTTTTGTTTTCCTCCATGTACATTTTTTTTTGTTTTTTCAACCTCTTTATTCTTTTTGCTACGCTTCCTTTTATACCCTTTCTAAAAATGTCTTGTATTAGGTCGGAAATCTCAATTACTGATAATATGAATGGTTCGAAAAGAACATCCCAAGAAAATCCTACTCTGTCGAAAAAAAGTGGGGAATACGGATATAAGGGAAACATTTTCCCCGTAAGGGTTTTACGTCTTTGAACACGCATAGAACCTGTGATTATGTCTCGACGAAAATCCGCTTCATAGGGATAATCTATCATATCCACTTCTTCTATTTCATCAGGCTTCGTCATAGTTATACTAGGGTCGGCATTCTCTGCTTCCTCCGGACCCTGCGTAAAAAGAACTATACGTTTCGCTTTCCTCGAGCGAATTTGATGATCTACTATCCACTCTTCCCCCTCTTCCGTTGTTGCAGTTTTTCCGAGTTGTTCTACCTCGCTGAATAATTTGTATGACCATCGGGGGACTTTTTTATTTATTCCAATCGATTTTTTTCGAGTTGTTTTTTCCATGGGAGGAATTATGGCTGTATCGCATATTGTTTGAATGATGGGATCAATTATGACTCTTTCGATTAAAAATTTCAAAACTTTTTCTGGATCTTCTGAATCAATTCGTCTTTGTTCCGGAAATAAAGAAATTCCTTTCAAATTTGATGTTGATTCTTCAGCAAATTCATCGATTAAAAGACTCAATTCTCTTGCTAATGATTTTTTATCCAATGTATATATTTTCTGTCCCAATTTATCTTCCAATTTCTGGTCCAATTTCTGGACCAATTTCTCTTCCAATGTAGCTATTTTCCCTTCCAATTTCTGGTACAATTCTTCAAAATTATGAACATTAAGTTCCTTACCCTTAAAAAGAAGGATACTATGAACTTTATTGAGTTTATTTATAAAATTTGTCTCTATCGAATTTTTGACTGCAGTTTCATTTAGGATTGAAGGTAAATAAAATTTCTTAATTATTCCACGATAGGATCCGTTTAAGAGAGGATCATATATTTTAGGCAAGTATTCTTCTTTAGTTTTATTATTGCATAATCGAGTCTTTTTTTCGAGTACATCCAGATAAGGAGATCCTCTGTCTAGGGCTTCAATTCTATCTGTAAACTCGTTCCTTAGGCTCCTCCATTTTTTTTCATTGGTATAAATCCAACAATAATAATTATGCAGTTCATCATAGAAGAATTTATCCAGTTCATCACAGACGAATTTTTTTGTTGTAAAAAAATAAAAATACATTTTTTGTCGTAGCATTTCAAAAAAAGCTGATAAACTGGATGGATATGTAAAAGAAATTCTTCGTTTTCCATCACTTTGACATGTATAAAAAAAATATTGTGACATTTCATTTCTTACAGCATGTTCGAATCGATAATTTTTTATATATCGCAATGGACGATTCCATCGTTTATAGTCGAAAAGAAGACTCACAGGGGGTTTTTCAAACCAGAAGAGGTCTTTATCTTCTTTTAAGTGAAAGTGGAATTCATCCTTTGTTTTGTCCTTTCCATTCACTCGGATCCTTTCCGTTTCATCGATTTTGTCCGGATCCTCCCTTTCTTCCGAAAAAAGGGAAGGAGAAGGATCTTCTTCGGTGAATCCCTCTTGTTCCTGTTTAGTCCCCTTCGTTTCGAAAGTTGTTTCTATTTCTACATCTCTTTCTTCCTCACTTTCCCCCCTTTCTGTCGTTTTTGAGGTTTCTTGCAGTTTCCTACTAAAAATGGGTGACGGCATTCTGCCTAAAGAGTAGACACAGCTAATAAATAAGAGAATACTAAAGATTCGATCCATAGAATCTATCAAGTCTAACACAAAGTACTTAAATTCTGACACAAGGTACTTAAATTCTGACACAGAGTACTTGTACTTCTTATACCTCTTAGATCGAATAATTCCATTAAGGTACTTATTAGATCGAATAAGTATTACTTTAAGGTACTTATTAGATAGAATAAGTACATTAAGAAGGTACTTATTAGATCGAATAGGTACATTAATAGCTCGAATAAGTACATTAAGAAGGTACGTATTAGATCGAATAATCGATCGAATAGAATAATTTTGCCGTATCCAGACTAATACCAAGCCAACACATTTCATGAATAAAATGTGACCAATTAACCAACCAACAAAACTACTTGTTACAAATAACATCTTGTTGTTGCATCGAAACATATAAATGTTGACTAATCTGGCTAACATTGAACTTGGTAAAACGAAATGGTTGAATAATTGAAAAATGAGATTATTCAGGAATACACATTGAATGCTGAGATTACGCATTGAATTTCTGGTAGTAGATCCATAATCAAAATGATTGCTCCAGAAGAAATTAAACAAAAGATAGGGTAGAGCTAGGAAAGTTATTGTATGAGGTCTACCCAATGCTAGATGCAGAGGCGTATAATAGATCGATATGAACATCATGAGCTGTCCCATAATAAAACCGGTTGTTGCTGCTACCTTCTTCTCGGTTCCTTCTTCTCCTTCTTCCATAACCAGAGTTCGGAGAAGGACGAGATAGGAGGGCCCTATGGAGAATGTGGTCAGAAATCCATAATAGAGTCCGACCACAACGACCGAATTGACTATCTTCATGCATAAGTTACCTAGTAGAAAAAAAATCATGACAAACCCCCTTTGTGATTTTGCAATTTCTGAATTATGATTATTATATAATAAGTTTTTACCTTCTTATTATAAGATAATCTTTGACCTTATTATTTGACCTTATAAAATTATAAAATTCTATTATTTATATTATTCTATTTATATTATTATTATATTAATATTATTCTATTCTATATTCTATTCTATATGATTCTATTATATATGATAAGTTTTTACCTTCTTATCTTATATGATAATTATTATAAGATAATTGTTTAACTTATTTTATTCTAGATAGAAATAGAAAGAGATAGACTAGAAACGAGATCTCTTATGTCAATGACACCAAAGGGATATTAAATGAATGTAATTGGGATATGGATGGAATATAATGAAATAGAGCCACTTTGAGGTTCCCTATGAAATGAGGCATGGAAGGGAGCCACTACGAAGAAGTTTCGGGAGTTACGAAGGAAGCTTCGAGCTCATATGGGTCATGGGTTGAGAACGGGAATTTAACTCTATGAGATCAAATCCCCCGTTGTTCCTCAGTAGCTCAGTGGTAGAGCGGTCGGCTGTTAACTGACTGGTCGTAGGTTCGAATCCTACTTGGGGAGATTTGATTCATTCTGAATTCTTTAATTCAGAATGAGAATGAAAGGGCTCGCTTTGACCGTTAAGAGTAGGTAACCCGTTCCCTGTCTTTGTTTCTATTGTATTCTATCTCATCGTATGACATTCTGTTCTGCGATATTTGAGAATCTCCGTCAATACCTCGATGTAGGTCCGGGATAATCCTTTGTTCCATAGTCCTGGGGCTATTTACAACTAGCCAATTCAGAATTCTCGGATGTACTAGTACTAGCAGTGCATCAAAGATGGAGTCATCGATTCTCCCGAGAGGCTACAATTACCGCGAGCAAACATAAAACATATTAATGACGAGGAACGCTTTTTTGCTATGCTACTAATACTTGTACTTGCTCTGCTATTCTGCCCAAGCCTGGCTGAGGAAGAGTTACGGGACGTAAAACAAAAAAAAATATGCTGCCAGCCGGCGGCCGGGGTATGCTATAATTTTTAATTACCTCATTCACGATAACACGTAAAAAAAAAAGGTAAGGTGCATTCCATTTCGACAAAAGACCCACACCCAAGTCCCATAGCTTTGGGTCCGCTATCCCGATCATGATTTTCCTATCCCCGGAGGGATAGGTCCTTCCCAAAGGGGAAGGTTGTGGGCGAGGAGGGATTCGAACCCCCGACACCGTGGTTCGTAGCCACGTGCTCTAATCCTCTGAGCTACAGGCCCCACCCCGCCTCCACTGGATCTGTTCCCGGGAGTATCCTCAAAAAAAAGGAACCTTTCCTCTCCCCAGCCATTTCGGGTTAAGAAGATGTGAAAGCGCGTTTCTATCTATAAGAACGGTGCGTTCCGAGGTGTGAAGTGGGAGAGAGGGGATGTCATAATTGGGGTTTTGAATAAGACGACCCTTTCTTTTTTCCTTTTTTCATTTTTTTTCTTTCATATTGAAAAAGTAATAAGAATGAGAGGTGTTAAGCTTTTTATCATCCTGGCGTCGAGCTATTTTTCCGCAGGACCTCCCCTACAGTATCGTCACCGCAGTAGAGTTTAACCACCAAGTTCGGGATGGATTGGTGTGGTTCCTCTACGCCTAGGACACCAGAATATCGAACCACGAACTAAGAAAGGCATGAGAGAAAATTGGCTAGTGATTGTGAGGCCCCAATTCTTGACTGGAGGGGACACCAAAGGCCTCTGCCCTTCCATCCCTTGGTTAGATATAGAGGGAGGGCAGGGCTTTTGGTTTTTCATGTTGTCAAAGAGTTGAACAATGGTTTTTTCGTATTGTCAAAGAGTTGAACAATGAAAATAGATGGCGAGTGCCTGATCGAATTGATCGGGTCATGTAGGAACAAGGTTCAAGTCTATCGGTCTGTTAGGATGCCTCAGCTGCATACATCACTGCACTTCCACTTGACACCTATCGTAATGATAAACGGCTCGTCTCGCCGTGACCTTCTCTTGAATTCTCAAAACTTCTGTCGCTCCATCCCCGCAGGGGCAGAGAACCCGTCGCTGTCTCGGCTGTGCTACCGGAGGCTCTGGGGAAGTCGGAATAGGAGAGCACTCATCTTGAGGTGGGCTTACTACTTAGATGCTTTCAGCAGTTATCCGCTCCGCACTTGGCTACCCAGCGTTTACCGTGGGCACGATAACTGGTACACCAGAGGTGCGTCCTTCCCGGTCCTCTCGTACTAGGGAAAGGTCCTCTCAATGCTCTAACGCCCACACCGGATATGGACCGAACTGTCTCACGACGTTCTGAACCCAGCTCACGTACCGCTTTAATGGGCGAACAGCCCAACCCTTGGAACATACTACAGCTCCAGGTGGCGAAGAGCCGACATCGAGGTGCCAAACCTTCCCGTCGATGTGATCTCTTGGGGAAGATCAGCCTGTTATCCCTAGAGTAACTTTTATCCGTTGAGCGACGGCCCTTCCACTCGGCGCCGTCGGATCACTAAGGCCGACTTTCGTCCCTGCTCGACGGGTGGGTCTTGCAGTCAAGCTCCCTTCTGCCTTTGCACTCGAGGGCCAATCTCCGTCCGGCCCGAGGAAACCTTTGCACGCCTCCGTTACCTTTTGGGAGGCCTACGCCCCATAGAAACTGTCTACCTGAGACTGTCCCTTGGCCCGTGGGTCCTGACACAAGGTTAGAATTCTAGCTCTTCCAGAGTGGTATCTCACTGACGGCTCGGGCCCCCCCGGAAGGGGGCCTTCTTCGCCCTCCACCTAAGCTGCGCAGGAAAGGCCCAAAGCCAATCCCAGGGAACAGTGAAGCTTCATAGGGTCTTTCTGTCCAGGTGCAGGTAGTCCGCATCTTCACAGACATGTCTATTTCACCGAGCCTCTCTCCGAGACAGTGCCCAGATCGTTACGCCTTTCGTGCGGGTCGGAACTTACCCGACAAGGAATTTCGCTACCTTAGGACCGTTATAGTTACGGCCGCCGTTCACCGGGGCTTCGGTCGCCGGCTCCCCTGTCATCAGGTCACCGACTTCCTTGACCTTCCGGCACTGGGCAGGCGTCAGCCCCCATACATGGTCTTACGACTTTGCGGAGACCTGTGTTTTTGGTAAACAGTCGCCCGGGCCTGGTCACTTCGACCCCCTTTGTGAGGAGGCACCCCTTCTCCCGAAGTTACGGGGCTATTTTGCCGAGTTCCTTAGAGAGAGTTGTCTCGCGCCCCTAGGTATTCTCTACCTACCCACCTGTGTCGGTTTCGGGTACAGGTACCCCTTTGTTGAAGGTCGTTCGAGCTTTTCCTGGGAGTATGGCATGGGTTACTTCAGCGCCGTAGCGCCTGGTACTCGAATATTGGCTCGAGGCATTTTCTCTACCCCTTCTTACCCTGAAAAAGCAGGGTCACCTTGCATCCTTGAACCGATAACCATCTTTCGGCTAACCTAGCCTCCTCCGTCCCTCGGGACCAACAAGGGGTAGTACAGGAATATTCACCTGTTGTCCATCGACTACGCCTTTCGGCCTGATCTTAGGCCCTGACTCACCCTCCGTGGACGAACCTTGCGGAGGAACCCTTAGGTTTTCGGGGCATTGGATTCTCACCAATGTTTTCGTTACTCAAGCCGACATTCTCGCTTCCGCTTCGTCCACCGCGGCTCGCGCCGGTACTTCCTACTAAGGCGGAACGCTCCCCTACCGATGCATTTTTACATCCCACAGCTTCGGCAGATCGCTTAGCCCCGTTCATCTTCGGCGCAAGAGCGCTCGATCAGTGAGCTATTACGCACTCTTTCAAGGGTGGCTGCTTCTAGGCAAACCTCCTGGCTGTCTCTGCACCCCTACCTCCTTTATCACTGAGCGGTCATTTAGGGGCCTTAGCTGGTGATCCGGGCTGTTTCCCTCTCGACGATGAAGCTTATCCCCCATCGTCTCACTGGCCGACCTTGACCCCTGTTATTTGGAGGTCATATCTAGTATTCAGAGTTTGCCTCGATTTGGTACCGCTCTCGCGGCCCGCACCGAAACAGTGCTTTACCCCTAGATGTCCAGTCAACTGCTGCGCCTCAACGCATTTCGGGGAGAACCAGCTAGCTCTGGGTTCGAGTGGCATTTCACCCCTAACCACAACTCATCCGCTGATTCTTCAACATCAGTCGGTTCGGACCTCCACTTAGTTTCACCCAAGCTTCATCCTGGTCATGGATAGATCACCCAGGTTCGGGTCCATAAGCAGTGACAATTGCCCTATGAAGACTCGCTTTCGCTACGGCTCCGGTGGGTTCCCTTAACCAAGCCACTGCCTATGAGTCGCCGGCTCATTCTTCAACAGGCACGCGGTCAGAGCCCTGTGTGCTCCTCCCACTGCTTGGGAGCTTACGGTTTCATGTTCTATTTCACTCCCCGATGGGGGTTCTTTTCACCCTTCCCTCACGGTACTACTTCGCTATCGGTCACCCAGGAGTATTTAGCCTTGCAAGGTGGTCCTTGCTGATTCACACGGGATTCCACGTGCCCCATGCTACTCGGGTCAGAGCGTAAGCTAGTGATGCTTTCGGCTACTGGACTCTCGCCATCTAGGGTGCAGCACTCCACCGCTTCGCCTAGCAGCACGACGCTTGTATTGCTCTCCCACAACCCCGTTTTCACGGTTTAGGCTGCTCCCATTTCGCTCGCCGCTACTACGGGAATCGCTTTTGCTTTCTTTTCCTCTGGCTACTAAGATGTTTCAGTTCGCCAGGTTGTCTCTTGCCTGCCCATGGATTCAGCAGCAGTTCGAAAGGTTGACCTATTCGGGAATCTCCGGATCTATGCTTATTTTCAACTCCCCGAAGCATTTCGTCGCTTACTACGCCCTTCCTCGTCTCTGGGTGCCTAGGTATCCACCGTAAGCCTTTCCTCGTTTGAACCTCGCCCTTAACTTTAAGGCTATGCCATCCTAAGGTGCTGCTAAGCTAAATGGAAGGATCTTATCAACGTCCATGAATGATAAATCATAGATCGAACCGCCGAATCGGAAAATTTGGGTGCTATCATATAGCTTTGTATCGGCTAAGTTCACGAGTTGGAGATAAGCGGACTCGAACCGCTGACATCCGCCACAGGGTAAACCACCGTCTCTCAGGCCCCCCGACTGATTCTACCATAGAGGCTGACGATAGACAATAACTCCCCCCCGAACACAGCTTACAACTTTCATCGTACTGTGCTCTCCAAAGAGCAACTCTTCTCAAAATCTCAAAAGGTGCTGAGTTGGAATCCAATTCGAACTAAGGATTCTTGTGGTTCCGGAGGATCCAGCTACAGGAGAACCAGGAACGGAGAGCTTTCCCCCCTTTTCCGCCCAACTCTTTGGTCTTAAGAATGCTGGTTTTAAGAATGAGTGATTGCCCTTCTCCGACCCTTACTGCCCAACCGGAGAGCGGACAGCTAATGCGTTCCACTTATTGAACAGGGTTCTATGGTCGGCCCGTGACCCCTGGATGCCGAAGGCGTCCTTGGGGTGATCTCGTAGTTCCTACGGGGTGGAGACGATGGGGTCGGTCCATGGATTTTCCTTCCTTTTGCCGCATTTCGCTCAAAGGGTTGAAGGGAGATAGTGCATCAGGCTGTTCGCAAGGGCCAACTTGATCCTCTTCCCCAGGGATCCCAGATTAAGGAACCCTAGGAGAGCCGCTGACTCCAACTACGGTCCATGTACGATCCATACTAGATCTGACCAACTGCCCATCCTACCTCCTCTACGTTCTTGACAGCCCATCTTTGTCTCAGTAGAGTCTTTCAGTGGCATGTTTCGGTCCTCTTCCCCATTACTTAGAAAAAGTGAGCCACCGGTTCAGGTACAAGATACTATCATTACCGCCTGGACAATTAGACATCCAACCCGTAATCGCAACGACCCAATTGCAAGAGCGGAGCTCTACCAACTGAGCTATATCCCCCCGAGCCAGGTGGAGCATGCATGAAGGAGTCAGATGCTTCTTCTATTCTTTTCCCTGGCGCAGCTGGGCCATCCTGGACTTGAACCAGAGACCTCGCCCGTGAAGTAAATCATCGCACCTACGGTCCAACCAATTTGGAGAGAATCAATAGATTCCTTTTCGGGAGCGATTCATCCTTCCCGAACGCAGCATACAACTCTCCGTTGTACTGCGCTCTCCAAGTGTGCTTGTTCGCTCCTTCTTCCTTCTTACCATGGCAAGTCTTTGTGAAATAACCCCGATGGGAAGAAAAAAGAAGGCGTTAAGAGACCCTCCTGGCCCAACCCTAGACACTCTAAGATCCTTTTTCAAACCTGCTCCCATTTCGAGTCAAGAGATAGATAAATAGACACATCCCATTGCACTGATCGGGGGGCGTTCGTAGTGACTGAGGGGGTCGAAGACCAAGAAGTTAGTTATTTATACCAAGCATTCTTCTTACGGCTAGATCCAATCTCCTGGTCCCTGCGGAAAGGAAAAAGAATTTCACGTTCTTCCTTTCGGGAAGGGAGGATTAGGGAAATCCTATTGATTGCAGCTTTCTCCAGACCTCCGGGAAAGCATGAAAAAAAAAAGGCTCGAATGGTACGATCCTTCCGTCACCCCAGAATGAAGAGGGTGATCTCGTAGTTCTTGGTCTGTGAAGATGCGTTGTTAGGTGCTCCATTTTTTCCCATTGAGGCCGAACCTAAACCTGTGCTCGAGAGATAGCTGTCCATACACTGATAAGGGATGTATGGATTCTCGAGAAGAGAGGAGCCGTAGTGGTCCCCCCCGGACCGCCCGGATCCCACGAGTGAATCGAAAGTTGGATCTACATTGGATCTCACCTGAATCGCCCCATCTATCCTCCTGAGGAGAAGTTTGGTTTCAAACCCCGGTTCGAACAGGAGGAGTACGCCATGCTAATGTGCCTTGGATGATCCACATCTCAAGGTCAGGCGCGGATGGGCACATTGAACTATCCATGTGGTTGAGAGCCCTCACAGCCCAGGCACAACGACGCAATTATCAGGGGCGCGCTCTACCGCTGAGCTAATAGCCCGTCGTGCGGGCCCCCAGTGGGAGGCCCGCTATGCCAAAAGCGAGAGAAACCCCATCCCTCTCTTTCTTTTTTACGTCCCCATGTCCCCCGTGTGGCGACATGGGGGCGAAAAAGGGGGGATCCTATCAACTTGTTCCGACCTAGGATAATAAGCCCATGAGCTTGGTCTTACTTCACCGTCGAGAAACGAAAGAAGACTTCCATCTCCAAGTTTCACCCAGGCGTCGCTCGCTTCTTTTTGGGTGTGAAGCAGTGTCAAACCAAAATACCCAACAAGCATTAGCTCTCCCTGAAAAGGAGGTGATCCAGCCGCACCTTCCAGTACGGCTACCTTGTTACGACTTCACTCCAGTCACTAGCCCTGCCTTCGGCATCCCCCTCCTTGCGGTTAAGGTAACGACTTCGGGCATGGCCAGCTCCCATAGTGTGACGGGCGGTGTGTACAAGGCCCGGGAACGAATTCACCGCCGTATGGCTGACCGGCGATTACTAGCGATTCCGGCTTCATGCAGGCGAGTTGCAGCCTGCAATCCGAACTGAGGACGGGTTTTTGGGGTTAGCTCACCCTCGCGGGATCGCGACCCTTTGTCCCGGCCATTGTAGCACGTGTGTCGCCCAGGGCATAAAGGGCATGATGACTTGACGTCATCCTCACCTTCCTCCGGCTTATCACCGGCAGTCTGTTCAGGGTTCCAAACTCAACGTTGGCAACTAAACACGAGGGTTGCGCTCGTTGCGGGACTTAACCCAACACCTTACGGCACGAGCTGACGACAGCCATGCACCACCTGTGTCCGCGTTCCCGAAGGCACCCCTCCCTTTCAAGAGGATTCGCGGCATGTCAAGCCCTGGTAAGGTTCTTCGCTTTGCATCGAATTAAACCACATGCTCCACCGCTTGTGCGGGCCCCCGTCAATTCCTTTGAGTTTCATTCTTGCGAACGTACTCCCCAGGCGGGATACTTAACGCGTTAGCTACAGCACTGCACGGGTCGATACGCACAGCGCCTAGTATCCATCGTTTACGGCTAGGACTACTGGGGTATCTAATCCCATTCGCTCCCCTAGCTTTCGTCTCTCAGTGTCAGTGTCGGCCCAGCAGAGTGCTTTCGCCGTTGGTGTTCTTTCCGATCTCTACGCATTTCACCGCTCCACCGGAAATTCCCTCTGCCCCTACCGTACTCCAGCTTGGTAGTTTCCACCGCCTGTCCAGGGTTGAGCCCTGGGATTTGACGGCGGACTTAAAAAGCCACCTACAGACGCTTTACGCCCAATCATTCCGGATAACGCTTGCATCCTCTGTCTTACCGCGGCTGCTGGCACAGAGTTAGCCGATGCTTATTCCCCAGATACCGTCATTGCTTCTTCTCCGGGAAAAGAAGTTCACGACCCGTGGGCCTTCTACCTCCACGCGGCATTGCTCCGTCAGGCTTTCGCCCATTGCGGAAAATTCCCCACTGCTGCCTCCCGTAGGAGTCTGGGCCGTGTCTCAGTCCCAGTGTGGCTGATCATCCTCTCGGACCAGCTACTGATCATCGCCTTGGTAAGCTATTGCCTCACCAACTAGCTAATCAGACGCGAGCCCCTCCTCGGGCGGATTCCTCCTTTTGCTCCTCAGCCTACGGGGTATTAGCAGCCGTTTCCAGCTGTTGTTCCCCTCCCAAGGGCAGGTTCTTACGCGTTACTCACCCGTCCGCCACTGGAAACAACATTTCCCGTCCGACTTGCATGTGTTAAGCATGCCGCCAGCGTTCATCCTGAGCCAGGATCGAACTCTCCATGAGATTCATAGTTGCATTACTTATAGCTTCCTTGTTCGTAGACAAAGCAGATTCGGAATTGTCTTTCATTCCAAGGCATAACTTGTATCCATGCGCTTCCTATTCGCATGGAGTTCGCTCCCAGAAATATAGCCATCCCTAGCCCCTCGCGTCAATCCCACGAGCCTCTTATCCATTCTCTTTCAATCACGGCGGGGGAGCAAGTCAAAATAGAAAAACTCACATTGGGTTTAGGGATAATCAGGCTCGAACTGATGACTTCCACCACGTCAAGGTGACACTCTACCGCTGAGTTATATCCCTTCCCTGCCCCCATCGAGAAATAGAACTGACTAATCCTAAGGCAAGGGGTCGAGAAACTCAACGCCACTATTCTTGAACAACTTGGAGCCGGGCCTTCTTTTCGCACTATTACGGATACGAAAAGAATGGAAAAATTTGGATTCAATTGTCAACTGCTCCTATCGGAAATCGGATTGACTACGGATTCGAACCATAGCACATGGTTTCATAAAACCGTACGATTTTCCCGATCTAAATCAAGCGGGTTTTACATGAAGAAGATTTGGCTCAGCATGTTCTATTCGATATAGGTAGGAGAAGAACGCGACTCGGTATTAAAAAAAAAATAGAGGAAGCAGAACCAAGTCAAGATGATACGGATCAACCCCTTCTTCTTGCGCCAAAGATCTTACCATTTCCAAAGGAACTGGAGTTCCATCTCTTTTCCATTTCCATTCCAGAGTTCTTATGTGTTTACACGCCCCTTTGAGACCCCGAAAAATGGACAAATTCCTTTTCTTAGGAACACATACAGGATTCGTCACTCCAAAATGGATAATGGTAACCCCACCATTAACTACTTCATTTATGAATTTCATAGTAATAGAAATACATGTCCTACCGAGACAGAATTTGTAACTTGCTATCCTCTTGACTAGCAAGCAAAGATTTACCTACGTGGAAAGGATGATTCATTCGGATCGACATGAGAGTCCAACTACGTTGCATTGCCAGAATCCATGTTGTATATTTGAAAGGGGTTGACCTCCTTGCTTCTCTCATCGTACAATCCTCTTCCCGACGAGCCCCCTTTCCCCTCGGTCCACAGAGACAAAATGTAGGACTGGTGCCAACAGTTCATCACGGAAGAAAGGACTCACTGAGCCGGGATCACTAACCAATACTAATCTAATAGAAAATACTAATATAATAGAAAAGCACTGTCTTTTCTGTATACTTTCCCCGGTTCTGTTGCTACCGCGGGCTTTACGCAATCGATCGGATCATATAGATATCCCTTCAACACAACATAGGTCATCGAAAGGATCTCGGAGACCCACCAAAGCACGAAAGCCAGGATCTTTCAGAAAATGGATTCCTATTCGAAGAGTGCATAACCGCATGGATAAGCTCACACTAACCCGTCAATTTGGGATCCAATTCGGGATTTTCCTTGGGAGGTTTCGGGAAGGAATTGGATGGAATGTAATAATATCGATTCATACAGAAAGTTCTCTATTGATTTAAACGCTGTACCTATGGGATAGGAATAGAGGAAGAGGAAAAGCCGAAGATTTCACATAGTACTTTTGATCGAAAAATGAAAATCAATCTGATTTATTTCGTATCCCTCGCTCAATGAGAAAATGGGTCAGATTCTACAGGATCAAACCTATGAGACTTAAGGAATTATGGAAGGGAATAAAAAAAGAGAGGGAAAAAATAGAAAAAAAGTAAAAAGTAGAAGAACCCAGATTCCAAATGAAAACAAATTCAAACTTGAAAAGGATCTTTCTAATTCTCGAAGAATGAGGGGCAAGGGGATTGATCGAGAAAGATCTCTTGTTCTTGTTAGAAGATCGTGATTGGATCCGCATATGTTTGGTAAAAAGAATAATCTTCTCCTTTGAGAATAATCAAAAGTGGAACGTGTTCAATTGGAACATGAAAACATGACTGAATTGGTCCTAGTTACTCTTCGGGACGGAGTGGAAGAAGGGAGGAGATTCTCGAACGAGGGAAGGGATCCAATGACTTCGAAAGAATTGAACGAGGAGCCGTATGAGGTGAAAATCTCATGTACGGTTCTGTAGAGTGGCAGTAAGGGTGACTTATCTGTCAACTTTTCCACTATCACACCCAAAAAACCAAACTCTGCCTTACGTAAAGTTGCCAGAGTACGATTAACCTCTGGATTTGAAATCACTGCTTATATACCCGGTATTGGCCATAATTTACAAGAACATTCTGTAGTCTTAGTAAGAGGGGGAAGGGTTAAGGATTTACCCGGTGTGAGATATCACATTGTTCGAGGAACCCTAGATGCTGTCGGAGTAAAGGATCGTCAACAAGGGCGTTCTAGTGCGTTGTAGATTCTTATCCAAGACTTGTATCATTTGATGATGCCATGTGAATCGCTAGAAACATGTGAAGTGTATGGCTAACCCAATAACGAAAGTTTCGTAAGGGGACTGGAGCAGGCTACCATGAGACAAAAGATCTTCTTTCTAAAGAGATTCGATTCGGAACTATTATATGTCCAAGGTCCATAATTTAAGAGGTTTTCCCTGACTTTGTCCGTGTAAACAAACAATTCGAAATACCTCGACTTTTTTAGAACAGGTCTGAGTCAAATAGCAATGATTCGAAGCACTTCTTTTTACACTATTTCGGAAACCCAAGGACTCAATCGTATGGATATGGAAAATACAGGATTTCCAATCCTAGCAGGAAAAGGAGGGAAACGGATACTCAATTTAAAGTGAGTAAACAGAATTCCATACTCGATCTCATAGATACATATAGAATTCTGTGGAAAGCCGTATTCGATGAAAGTCGTATGTACGGTTTGGGGGGAGATCTTTCATATCTTTCGAGATCCACCCTACAATATGGGGTCAAAAAGCCAAAATAAGTGATTTTAGCCCTTATAAAAAGAAAACTGATTCTTGAACCCCTTTCACGCTCATGTCACGTCGAGGTACTGCAGAAGAAAAAACTGCAAAATCCGATCCAATTTATCGTAATCGATTAGTTAACATGTTGGTTAACCGTATTCTGAAACACGGAAAAAAATCATTGGCTTATCAAATTATCTATCGAGCCGTGAAAAAGATTCAACAAGAGACAGAAACAAATCCACTATCTGTTTTACGTCAAGCAATACGTGGAGTAACTCCCGATATAGCAGTAAAAGCAAGACGTGTAAGCGGATCGACTCATCAAGTTCCCATTGAAATAGGATCCACACAAGGAAAAGCACTTGCCATTCGTTGGTTATTAGGGGAATCCCGAAAACGTCCGGGTCGAAATATGGCTTTCAAATTAAGTTCCGAATTAGTGGATGCTGCCAAGGGGAGTGGCGGTGCCATACGCAAAAAGGAAGAGACTCATAGAATGGCAGAGGCAAATAGAGCTTTTGCACATTTTCGTTAATCCATGAACAGGATCTATATAGTAGACGCCTAGATCCATGGATCCATACATCTCGATCGGAAAAGAATCAATAGAAAAAGAAAGAATCGGAATTGATCGATATATTTATTTCGAAACAAACGAAAAGGAAACGAAAGATGAAAGATAAATCATGGATCAACTAAGCCCTCTCGGGGACTTGCTTAAGAATAAATAAGCAAGAGGAATCTCATGGAAATACCATGGAATAGGGTTGGATCCTATTCGTGGAGATTCCGTAAATATTCCATTTCAAAAATAGAAAGTTCTAAACAATTGGGATTTTTTTGGAGATTGGATGCAGTTACTAATTCATGATCTGGCATGTACAGAATGAAAACTTCATTCTCGATTCTACGAGAATTTTTATGAAAGCCTTTCATTTGCTTCTCTTCGATGGAAGTTTTATTTTCCCAGAATGTATCCTAATTTTTGGCCTAATTCTTCTTCTGATGATCGATTCAACCTCTGATCAAAAAGATATACCTTGGTTCTATTTCATCTCTTCAACAAGTTTAGTAATGAGCATAACGGCCCTGTTGTTCCGATGGAGAGAAGAACCTATGATTAGCTTTTCGGGAAATTTCCAAACGAACAATTTCAACGAAATCTTTCAATTTCTTATTTTACTATGTTCAACTCTATGTATTCCTCTATCCGTAGAGTACATTGAATGTACAGAAATGGCTATAACAGAGTTTCTGTTATTCGTATTAACAGCTACTCTAGGAGGAATGTTTTTATGCGGTGCTAACGATTTAATAACTATCTTTGTAGCTCCAGAATGTTTCAGTTTATGCTCCTACCTATTATCTGGATATACCAAGAAAGATGTACGGTCTAATGAGGCTACTACGAAATATTTACTCATGGGTGGGGCAAGCTCCTCTATTCTGGTTCATGGTTTCTCTTGGCTATATGGTTCATCCGGGGGAGAGATCGAGCTTCAAGAAATAGTGAATGGTCTTATCAATACACAAATGTATAACTCCCCAGGAATTTCAATTGCGCTTATATTCATCACTGTAGGAATTGGGTTCAAGCTTTCCCCAGCCCCTTCTCATCAATGGACTCCTGACGTATACGAAGGAGTGCGGTTCGTTCGAAAAATTCCTAGCTCTCTATCTATCTCCGAGATGTTTGGATTTTTCAAAACTCCATGGACATGCAGAAGAGAAATGCTATCCCCACTCGGACCAAGACATAACTTTTACTTGTTCAAATAACAATTAAGGTGAAGCAGGGTCAGGAACAACGAATCTCTTTATGATAAACAGATCCATTTTGCAAGTTCGTTATTACGGGTAGTTCCTACAAAGGATCGGACTAATGACGTATACAATACTTGAATTCTCGATGTAGATGCTACATAGTTGGTTCTCATCCTTCAGAGACTACGAGTGTAATAGGAGCATCCGTCGACAAAAGGATCACCCTAAGATGATCATCTCATGGCTATTGAGAACGAATCAAATCAGATGGTTCTATTTCTCAATCTTTCTGACTTGCTCCTACGGAACCAAGGTCGAAAAGATTGAAAAAATAAGTCATTCGCAACCACTGATGAAGGATTCCTCGAAAAGTTAAGGATTAGTAATCCTTTTTATAAATCGAACGGATTCGGTCTTATACATACGCGAGGAAGGTAATCAAAAAAGAAAGAAGATGAGTTCTTCTTTCTTTTATCACTTAGGAGCCGTGCGAGATGAAAGTCTCATGCACGGTTTTGAATGAGAGAAAGAAGTGAGGAATCCTCTTTTCGACTCTGACTCTCCCACTCCAGTCGTTGCTTTTCTTTCTGTTACTTCGAAAGTAGCTGCTTCAGCTTCAGCCACTCGAATTTTCGATATTCCTTTTTATTTCTCATCAAACGAATGGCATCTTCTTCTGGAAATCCTAGCTATTCTTAGCATGATAGTGGGGAATCTCATTGCTATTACTCAAACAAGCATGAAACGTATGCTTGCATATTCGTCCATAGGTCAAATCGGATATGTAATTATTGGAATAATTGTTGGAGACTCAAATGATGGATATGCAAGCATGATAACTTATATGCTGTTCTATATCTCCATGAATCTAGGAACTTTTGCTTGCATTGTATCATTTGGTCTACGTACCGGAACTGATAACATTCGAGATTATGCAGGATTATACACGAAAGATCCCCCTTTGGCTCTCTCTTTAGCACTATGTCTCTTATCCCTAGGAGGTCTTCCTCCACTAGCAGGTTTTTTCGGAAAACTCCATTTATTCTGGTGTGGATGGCAGGCAGGCCTATATTTCTTGGTTTCAATAGGACTCCTTACGAGCGTTGTTTCTATCTACTATTATCTAAAAATAATCAAGTTATTAATGACTGGACGAAACCAAGAAATAACCCCTCACGTGCGAAATTATAGAAGATCTCCTTTAAGATCAAAGAATTCCATCGAATTGAGTATGATTGTATGTGTGATAGCATCTACTATACCAGGAATATCAATGAACCCTATTATTGCAATTGCTCAGGATACCCTTTTTTAGCTTCTAGAGTCTATTTCATCCCTCTTACTAACTGGAATCAAAGAATTAGTAGATCTGTTCCGCCCAAAACGGGAATGGGCTAGGGTTATGAACTTATAATCTATAATCTGATGATCGAGTCGATTCCATGATTATAAGTTCATTCCATACCGGACTAGGCCGGAAGAGGGTTATATAAATTCTCATTATGAGAAGGGGTCATTCGAGCCTATCTAAATAGATACTATATTTCCATATGGATCCCTACGTCCTTACATTCCATTTCGGATTAGGAATAGGCGTAATCGGACCTTCTTTTTACATATCTCTCGTTATTTGATTTGGGACCCTATTCATCTCTTTGGGCTTCTATTGAATCGAGAAATAGGTTTGATTGTCCATCTTTTTGATATATATAAGTATAAGGCATCCTCCGGAAAATGAAAATCGAAGCAATTGGATGTCCGACTCGGGCCTATATGACATGACCGATCAATAGCAATACTCCAACACTCCACCTTTGTCATATATTCCATACATCACACTAGATAGATATCATATTCATGGAATACGAATCACTTTCAAGATGCCTTGGTGGTGAAATGGTAGACACGCGAGACTCAAAATCTCGTGCTAAAGAGCGTGGAGGTTCGAGTCCTCTTCAAGGCATAGAGAATGCTCATTGAATGAGCAATTCAATAAGAGATCTTGGAATTGGAATAAGTTCGGCAGCGGATCACGAAATCTTGGTGATCTTCTCTATCTAATGAGGGGGGAGTCCGCTTTGAAATCGTCCGCCCTGAACCCCCCCCCGAGTATATGCTTCAACAGGAATCACACAAGGGTAGATTGATACAATAGAAACCTCTGGTAAAATGCCCGCCCGTAACTCAGCAGAGAAATTACATAGTCCGTTTTAGGGATTGGCGACTTACCCATTAGTGACTTTGGCACTGGACGTTCCCCCCCAAAATGGGTACTATCGGGTCGGGTGAATTCAAGAATAGACGCCTGTTGGCATTCCAGCCTCCCTTCTCCTTTCGGGGCCTATCCGAAAGAGAATCCAGTACTTCTTGGTCGTGAATATCTGAATGAATAGGACGAAGGACCCCGTGGATACCTTTGCTTCGGAACAAAACAATTCGGTCAACTGGAATGTGTATTATCCCTATAGGGTATTTTATTTAGTTATTCAGTCAAACCAATGATTCGTTATTGGAACAGATAGCAACTTTCATCAGACATGCGTATTTTTTATTTTACAATGGATTTCCATCTTTCATTAATGGAAATTTTTTTATGTAGTGAGTAATAGGCTCTGGTTGTTCGCTGTTCAAGAATTCTTGTTTAGGCCGTTCATACCATCCATACATAGTGTTTTGTTTTGATCTAAGATTTCAATTCTTCCATCTTTCAGCAGTAGCATATTGTTCCATGGAGCTAAGGTCCGAAATATGGAAAAAACAAGTATTTCCACGACTCTACCGCCCAGTCAATTCTGTTCCACTTAATCCCTCTTTCATGGCCACATATCTTTACGGCTAAGGAATGGGAAATCTTTCTCCTGTTACATGAATCCAATTTTCAGTTCATCCGGGAAAATCCATCTTTTTCTAAACAATGTCTTTGTCATTTGATCCAATAGCCTTCCGTTAGATAGGAACAGATTTGATAAGTACTGATAACTCGCGGATTGAATATTAGAACGGAAAGATCTATTATATAATGAACTAATGGTTCTAAGCCGTCTCCGTCTCTGGCGATTAATAAAAAATTCGAAGTACTTTTCTTTCGGTTTCTTGCTAAACCCGCGTTTATATAGAGTCTCCCTTTGGGAAGTCAGAAGAAGCCCCTTTGACATCTCTTCATCTGCAAAGAATTCTCGATGTGAAAACAGAAAGACAGAGAGCTCATCGTTGAATATGTAAAAGAGTGGATCTCCAGGGTCCCAAATGAATTGGCCTTTTCGAAAAAAGACTTGTTCTTTGGAAGATCTATCTCGTCCCGGGTACTCCATGGTTTCACTCTGCAAGAACTCCCAATCATTCTCTTGAAGCTCATCCTCTTCATCATATCCATCATCCCCTTCACCATAAAATGCATAATCAAAATATTCATCATTAACTTCATCAGAAATGATCGGCTTGCCCCGAAATGACCTGGCCCAATAGGGAAATTCCAATTCATTGGGCCTTTCGATCCAATCAAATAGAAAGCCCCAAGGTTGCCATATTCTAGGAGCCCAAACTATGTGATCGACTACATCCTCCGCTAACTGTTGCGGGTCGGTGCCTTCTGCCCATTCTTTAAACTCCGATTCATAGAGAAATCTACGATCAAAGATAGAACGAGATCCATTTTCCATCATCTCTAAGGGATTCCTTGGTTCGGGCCGAAGAAGCGAGGATCCCACCAGAGCGCCTTCTACTACTTCTAATAGGCCATCAACTAGATCAGAATCAGTCTCAACGAGTCTATAAGAAGTGATCCAATTTTTTTCATCGGGTCCGGGTAGAGACCAAAGATCTTGAGCGACCGATCCGGCAGAACAACTCAAAAGATAAAGAAGTATCGTTAATTTCTTCATGCTCGTTCCAAGTTCGAAGAACCATTTGTACAAATAAGGATCCCCTTCGTAACATGATTGCTTCTTCATATAGATAGATATAGGATCTATAAGGCAGCAATTATTTATAAGTACATTTTGTGCAACAGCCCTTCCTATCTGATAGAAAAGGATCCCATGATCCGGAACCGGTCTTACATGGGCTCGCAACTCCCAAGTTTGTCTATGAAGAGCGAATCTAATTGTATTAGTGTCTATAATTGATTTCTTCTGTGTAATACTAATCGATAGGGCCTCATTGGTAATTGCTACAAGATCTCGTGCATTGTAACCCATGGCTATGGACCCGAATCCATTAGTATGGAACATTTTCTTTTCCAAGTGAAATCCCCTAGTATATGAAAGAGTGAAAACGTGCTTTCGTTGTTGTGGAATAAGAAGCCTTCGTATCTTAATGCATGTATTTAATTTATTCGGAGCTATTAGAGCGGGATCCACTTTTTGGGGAATATGAGTCGAAGCAATAACAAGAATATCTCTAGTGGACCGTCTTTCACAATCCCCGGAGAGATAGTTCAATAATAAACCGAGGGACTCCGACTCATCCACATACAGATCATGAATGTTTGGAATCCATACTATGCAAGGAGATATTGTTCTTGCCAATTCGAATTGCAAGTTGATAGAAGCTAGGTCTATTTCCAACTCGATGATATACCTAAGTATCTCATCCACCGTATACTCCTCCCTCAGCTCCGGGTCCGAGTCCAAGTTCGAATAAATATAGTCACGATCATCAATATCGTCCACATCTTTCAGCGCATCCATATATTCCTTAGCAGGATCGCTATCATCATCAATACCATCAATATCCACATCATCAAGCGCTTCCATATAGTCCTCAGGATCGAGATCATCAATAACTATTTTTAAATCCAGCTTGTTCAGAAATACCGTAATGAAAGGAAGATAGGAGTTTGTCGCTAGGGATTTGACCAAATAGGATCGTCCAGTTCCTATAGGACCTATCACTAAAATACCCCTAGAGGGGGATAGGGCTAGGCGGAACGAAAAGGGTTTTGGTTTTCCATGAGATGGGAAATGAAAACTATTAGCCCCACACGAGGTTTGTGAATAAGTGATTGTCTGATAATGAGCAAGGAATATCCGTCTTTCTGCTAAACAGGATGTATTGAACTCATAATTCATTAGATCCTTTTGATGAATGTCAACTAAGTATCGTAAGTAAATTGCTCCCGCTTGTTCAAACATTTGATAACCATAGTCACTCTTTAATAAATGATCAATATGAGTCAGACTCAATAGAATTTGATCAATCCCTTTTTCTGGCCTTAAGGTGGAGAAATGAAACGAGTAAACTCTCTCTTCATCCAAAAACCAATCACAGGTCTCGATCCAGGATTCTATTTCATCATGAGTCTGAAACCTTTGTCCTTTTCTTATCCATGAATAGATCTCTTTACTTGTATGACTTAGATGTCTCGTATTTCTCGAAAAAGTGATTCGATTGATGGGATTTGGTATGATACTTATGAGATCGATGAGATTGAAAAATATCTTCTGTATAAATTTGACCCCATAAGCGGGACCACCACCAAATAGCGCAAAACCCAGTATTTCTGCTAGAAAATCTTCTAATTGTTCCCGAGCAACTAGAAAGAGATTCTTTAACCAGAAAGAATTCGGTTCAGGTTTAGGATACCCATCCACAAGTTTGTACACCTCAATCGTGTATGATGGAATCGTCAAAGATTTTATCCTCTCGAACTCTGTCTGTAACTCACTAGAGTCTAGGGAAACAGAGAAAAGAAGTACCTGAACGAGACATCCAGCAAGAAGAAGAAGTAAAAGGCTTGAATAGAGGAACTCCCGAACATTTGGCGAGCTCAGATGTGTCGATATCAATGGTGACTCATTATTTCGATGAATCATTTCTTCGACCCGAAGAAGCCTACGGAAACACTTCCACGAAATATCACTTGAAATCTCACTTATTGGTGCCCACAATCCCCACAATTTTAGCTTAAGAGCTCGCCATTTTAGCTTAAGAATTCGCCATGCTGATCTGTGCATAATATAATGAAAAATGGATACAAATTTGTGACTGCTACTTAGCATCGGCAATAGGTCTGAAAAAGCATCTAAAAATAGCAAATTTAGATATTTGTACCCTGTCGAAGTAAAGAACCATGGCATATATGTTTGGAATAGATTCCATTTTGAGAGAGTTGAAAAAGCACTATCTCGTTGAAAGGTTCTATCCATCTGCCCTTTCTCAACGCCTTTCTTTAGCTTTAGCCAATTTCGCCAAAGACGCAATTTTTGACTCGTTTCGGATGGTAAATATTTCTCAGAACGTGGAGTGTGAATCAAACCCATGTTTGAATTGAAATTGAGATACTGATGCAAGTTCTTCCTTTCTGAATCAGATAGATTCATATCTGAAAGAGGTTGACAATAAGTTCTTTCCAAATTGACTATTTCTTCCTCTGTTAGAGGTGTTCCAGAAATGTCTGCGATCGAGTAAATAGTTCTACGAACGAATAGATCGGATCGAATTGGAAAATGGAAAGATTTGTACAAGTTATACCCTTCGTTACCCCTTTGTGGAAAATCGTTAGGTATGAATATGTTAGATACCTGTGACTCGATTGGTGAAATAGTATCTCTCTCCAAAAAAGCATGTTTTTTTTTACCGACGCACAAAGAAAATTTTTTGTTGCGAATGAACAAGATATTGAGGAATTGTCTATACGTAAAATCATAATTCTTGATACGGGCCTTTTCCATATAAAAAGGGAATCTTTTCTTACAATAGAAGAAGAAGTGATGTGGATTATTCAAGAATCGAAGTCGATTTGCTTTATAAAAAGAAGATATCAATGAACTTCTATGAAATGCTTTTACGGGATTCAGCCAATTGTCTTGATCGCAGGATATCATTGAGAAATAGGAATCCGTGTTATCAAAGGATTTCCTGGGATTCTTTCTAGTATGGGAGTCAATCATCCACTTCCACTTTGGTATCTTATTGAACAAAAAGGGTGATATTGTTCCTCCATTGATCAATAATTTCGATTTTTGGGAAGTATCATGATCATCCGCTTTCCATTTTTTCAAATGAACGATTGGAAGACCTAGTGATTTTAACAACGGATTGCAGAGTTGATCATTCGGACCTTTCAATTCATAAATGTGGATCTCGGACCTATGAATGGGCATATTCCCTAAACTCACAAAGAAAAAAGGAAGTGAGTTAGACAAAAAGAGAATCAACTTTGACAATGACTTAGAAAATTTTTTTTTGTTGATAACCTTAGACCAATCAATCCAATATTGATTAATACGTAATCGATCGAAGACTACTTGAACTTGAAAACGGCTTTTCTGCTCAGAAACGAAATGTTCCTGGAAATTCTTGCTCCCGTTGAACCATTTGTATCTATATGCATCAGGATCCCGATTCATGGATCTCTCGCTTCGAGAAATCAAAATAAGAGAATCGAACCATTTCTTCTGATTCTTTTTCAAATTCGATAAATGTTGGTTGATCGTATATTTCATTATAGTTCTATGATTCAGAGTATCGTTTCCTATTTGATCCCTTTGAATTCCATATTCGAAGTTGCGATCGGATCTATTTATTAAAAAGAATCGATTCAATACATTTCTTATGTACCCATAAGTGCTATATTGGATTTGAATCAGATTTCGGATCAATCTATATTGATTGACTGCCTCCATTATGTTGTTGCTAGCAAATACCACTATTTTTTCTTTTGTATCTTTCAAATAATTCCCGCAGGAGATCCGAAGCCATTTTTTTCTGATCCTTCGATAAAAAGATTCATTCTCTTCATAAAAAATAGGAGGTAGAGCCAATAAAGATTTCTTTTTCGATTCATCCCTGGAGTTGAATACCTCATTCAAGAATTGTTTTTGATCCAATCCGTAGGAATCAATAGAAAAGGCAAATCCCTTATGATACACCAGATCCGGCTCGGTTATTGATAGAGTGAATAGATCTGCCATTTCTTGAAATCTCTCTTCGGATTCAAAATCGTGGTGTAACGTGTATCCCCCCCTGTTCCGGTCATGGAATAGATGAAATAAATCAAAAAATGGATTTTTGTTCAAGAATGAAATCTTATTGGAACTGTCCATATTCGGTTCATCCTTCGGAACCATATCACATCCCCGATCTGATGAAATAGGATGAATTGAGACGGTCTTTTGTAAATACGTAATTATCTTGAATATATTAACCATTTCTTTATTTTCCGATCGCCTGCAGGGGACAAAAGAAACATCTTGTTCTTTCTTCAACAATTTCTGATCTCTAGTGGACCTCTCAGTAGGATTTGAACCCAGATGAAGTTCTGACCATCTGTCAGAGAAAAAAGAACGAATGGATCTTGTAGGATTCCCAAGAAGTTCTTCGATTTCTTCCGGAAGCAGATGATTATTCATCTGCTTCTCACCTTCCGTGAATAGCCGAGACATTGAGGAATATCCAGAAAGGCATTTCGGGAATCGGCCTGATCCTATCTCTGTTTCTTCCGTTTGAAGAAAGGAAGGATCCCAAGGAATCGATCTTTCTTTTCGCTGTTGAATCTCCCTTTGATTGAGAGATGTGTGATATTCCGAATCCGCATTCCTGATGGAATCCAAGCGATCTCTAGATTGATCAAAAGATCCTTTCAATTGGCTAGAATCCCTCTTTTTTACGATCCAGTTCCTCCACCACCGCGAACCCCAGTTAGATTTACACCTTTTAGTTATTGGAGGAACCCAAGTACTCTCTTTCGGATCCAGGAAACAGCTCTCAGAGATCTTTTTTCCTTTTGGAAGATAGAGGAGCGAAACAATCAGCCTATTGATATTGGAAGACCCAAAGGATTCTTCCAATGTATCATTTCTGGACCCAAGAGAATTCATAGGTATAGGAAGAAGCCCTATCAAATAGATATTTTTTTTTTCGACCATATTTCGATTGTTAATACGATATATAAGGCCCGCTACTACAAAGAATACTACACCCTTGATTACAAAGAATACTACACCCGTGATCGTGAAAGTGAAATATCGCTTAATTATTGAACCCCGCGAATTGGGTGAAAGTAGGATACTCCAAATTCGGGGATCAAAGAGTTTTATAAAACGTTCTTGGTGGAAAAAAATCTTAACCAAAGATCCCACTGAATTCTTGATCTCTCTCAATATCTCTTTCAACTCGAAAACCCAGAAATCTAAATTTGTGGAATGTTTTTTTGTCATTTGTTAAAAAAAAAATTGAATTTTAAAATACTATAAATTTACTTTAATATGCAATTGTTAAAGTTAAAAAAAAATTCAATTTGATACTTATACTATAGTATAATACAATACGAAAACGTAATTGTATATCTATAATATAATATGATTATGATACTCCAATTATGATATTTCACTGAAAATTTAATCACTGAAAATGAAAATATGTCTAACATTTCTATATTCTATTTCAATGAAAATGTGTCTAAAATTTCTATATTCTATTATATTCTATTTCAATGAAAATGTCAACAATGAAAATTTCAATGATATTTGTATAAAATTAAAATTGGATTTGTATTGATTTATCCTAAAGATTTCATTTCAATTGGAATTTGGTTATTCACCATGTACGAGGATCCCCGCTAAGCATCCATGGCTGAATGGTTAAAGCGCCCAACTCATAATTGGCGAATTCGTAGGTTCAATTCCTACTGGATGCACGCCAATGGGACCCTCCAATAAGTATAAGTCTATTGGAATTGGCTCTGTATCAATGGAATCTCATCCTCCATACATAACGAATTGGTATATTCATTTCATAACATAGGAACAGTAAGAACTAGCATTCTTATTGAGACTCGAACTCATAGGGAAGAAAATAGATAGAATCACATATGCAATATATGCCATTTTGAATATGAAGAAAATAGATAGAATCACATATGCAATATTTACAGACAAAAGTATTCGGTTATTGGGGAAAAATCAATATACTTCTAATGTCGAATCAGGATCAACTAGGACAGAAATAAAGCATTGGGTCGAACTCTTCTTTGGTGTCAAGGTAATAGCTATGAATAGTCATCGACTCCCGGGAAAGGGTAGAAGAATGGGACCTATTATGGGACATACAATGCATTACAGACGTATGATCATTACGCTTCAACCGGGTTATTCTATTCCACCTCTTAGAAAGAAAAGAACTTAAATAAAAATACTTAATAGCATGGCGATACATTTATACAAAACTTCTACCCCGAACACAGGCAATAGGGCCGTAGACAGTCAAGTGAAATCCAATCCACGAAATAATTTGATCTATGGACAGCGTCGTTGTGGTAAAGGTCGTAATGCCAGAGGAATCATTACCGCAAGGCATAGAGGGGGAGGTCATAAGCGTCTATACCGTAAAATCGATTTTCGACGGAATGAAAAAGACATATATGGTAGAATCGTAACCATAGAATACGACCCTAATCGAAATGCATACATTTGTCTCATACACTATGGGGATGGTGAGAAGAGATATATTTTACATCCCAGAGGGGCTATAATTGGAGATACCATTGTTTCTGGTACAGAAGTTCCTATAAAAATGGGAAATGCCCTACCTTTGAGTGCGGTTTGAACTATGGATTTACGTAATTGGAAGTAACCAATTAGGTTTACGACGAAACCTAGAAATCGATCACGGATCCAATTTGAGTACCTCTAGAGGATAGACCTCAACAGAAAACTGAAGAGTAACGGCAGCAAGTGATTGAGTTCAGTAGTTCCTCATATAAAATTATTGACTCTAGAGATATAGTAATATGGAGAAGACAAAATTGTTTCAAGCACCGACAGAACCGGAAGCGCCCCTTCTTTCAAAGAGAGGAGGACGGGTTATTCACATTTCATTTGATGGTCAGAGGCGAATTGAAAGCTAAGCAGTGTCTAAAGATTCCCCGGGGAAAAATAGAGATGTCTCCTACGTTACCCATAATATGTATATGTGGAAGTATCGACGTAATTTCATAGAGTCATTCGGTCTGAATGCTACATGAAGAACATAAGCCAGATGACGGAACGGGAAGACCTAGGATGTAGAAGATCATAACATGAGTGATTCGGCAGATTGGGATTCCTATATATCTGCTCATGTGGTACTTCATTGTATGATAAATAGAAGATCCATCTGTATAGATATCATCATCTACATCCAGAAAGCCGTATGCTTTGGAAGAAGCTTGTACAGTTTGGGAAGGGGTTTTGATTGATCAAAAAGAAGAATCTACTTCAACCGATATGCCTTTAGGCACGGCCATACATAACATAGAAATCACACTTGGAAAGGGTGGACAATTAGCTAGAGCAGCGGGTGCTGTAGCGAAACTGATTGCAAAAGAGGGGAAATCGGCCACATTAAAATTACCTTCTGGGGAGGTCCGTTTGATATCCAAAAACTGCTCAGCAACAGTCGGACAAGTGGGGAATGTTGGAGTGAACCAGAAAAGTTTGGGTAGAGCCGGATCTAAGCGTTGGCTAGGTAAACGTCCTGTAGTAAGAGGAGTCGTTATGAACCCTGTAGACCATCCCCATGGGGGTGGTGAAGGGAGGGCCCCAATTGGTAGAAAAAGCCCAACAAATAAATGGGGCTTTCCTGCACTTGGAAGAAGAAGTAGAAAAAGGAATAAATATAGTGATAATTTGATTATTCGTCGACGTAGTAAATAGGAGAGAAATTTGAATTAGTTTCTTCGTCTTTAAATAAAAAAAATAGGAGTAATTAACCGTGACACGTTCACTAAAAAAAAATCCTTTTGTAGCGAATCATTTATTAAGAAAAATTGATAAGCTTAACACAAAGGAGGAAAAAGAAATAATAGTAACTTGGTCCCGG
>GymnopodiumFloribundum
CGGGCGAACGACGGGAATTGAACCCGCGCATGGTGGATTCACAATCCACTGCCTTGATCCACTTGGCTACATCCGCCCCTATGCTAGGCTACTTATTTGAAAATGATTCAAATTGTACTCACTATTCATCATTCTTTTTTTTTTTGTAACTTTTCATTTTTTTATATTTCTTATTTTATATTCTATTTCTTTTTATTTAAATTTTATTTAAATATTTAAAATGGAAGATAAGGAAAACTTATGTAAGTAAAAAAGAAAAATAAAAGGAGCAATACCAACCCCCTCGATAGAACAAGAGATTGGTTATTGCTCCTTTACTTTCAAAAACTCGACTACACTAAGACCAAAATCTTATCCATTGATAGATGGAGCTTGGATAGCAGCTAGGTCTAGAGGGAAGTTATGAGCATTACGTTCATGCATAACTTCCATACCAAGGTTAGCGCGGTTAATAATATCAGCCCAAGTATTAATTACACGACCTTGACTATCAACTACAGATTGGTTGAAATTGAAGCCGTTTAGGTTAAATGCCATAGTACTAATACCTAAAGCAGTAAACCAGATACCTACTACAGGCCAAGCAGCTAAGAAGAAATGTAAAGAACGAGAATTGTTGAAACTAGCATATTGGAAGATTAATCGGCCAAAATAACCATGAGCAGCTACGATATTATAAGTTTCTTCCTCTTGACCAAATCTGTAACCTTCATTAGCAGATTCATTTTCTGTGGTTTCCCTGATCAAACTAGAGGTTACCAAAGAACCATGCATAGCACTGAATAGGGAGCCGCCGAATACACCAGCTACGCCTAACATGTGAAATGGGTGCATAAGGATGTTGTGCTCAGCCTGGAATACAATCATGAAGTTGAAAGTACCAGAGATTCCTAGAGGCATACCATCAGAAAAGCTTCCTTGACCAATTGGGTAGATCAAGAAAACAGCAGTAGCAGCCGCAACAGGAGCTGAATACGCAACAGCAATCCAAGGGCGCATACCCAGACGGAAACTAAGTTCCCACTCACGACCCATGTAACAAGCTACACCAAGTAAGAAGTGTAGAACAATTAGCTCATAAGGACCACCATTGTATAACCATTCATCAACAGATGCAGCTTCCCATATCGGATAAAAGTGCAAACCTATAGCTGCAGAAGTAGGAATAATGGCACCAGAGATAATATTGTTTCCGTAAAGAAGAGATCCAGAAACAGGTTCACGAATACCATCAATATCTACTGGAGGAGCAGCAATGAAGGCTATAATAAATACAGAAGTTGCGGTCAATAAGGTAGGGATCATCAAAACGCCAAACCATCCAATGTAAAGACGGTTTTCAGTACTGGTTATCCAGTTACAAAAACGACCCCATAGGCTTTCGCTTTCGCGTCTCTCTAAAATTGCAGTCATGGTAAAAAATCTTGGTCTATTTCATTTAATCATCAGGGACTCCCAAGCGCACAAATTCTCTATAATCTATAATAGAAATAGATAATTGAAGGCTTGTTATTCAACAGTATAACATGACTTATATGCCCGTGTCAACCAATATGAATCTAGGTCGATCAGAATTTATTGTGAATAACTGAAAAAATAGAAAAGTTTTATACACATATGACTTCGATATGAAAAGGAATATGGGTTGCCCGGGGCTCGAACCCGGAACTAGTCGGATGGAGTAGATATTTTCCTTAGTAAAAAGAATAAGAGAAAATCCCTCCCCAAGCCGTGCTTGCATTTTTCATTGCACACAGCTTTCCCTATGTACACATCTAAAACTCAGTTACTTTCCTAGATGGAAGTTGAATACTCAGTTGATTCAAACCCTACTGTATGAACATTTCAGAATTGAAATGGATGAATTTGATTATTTATCATATTTTTTTACTAGAATTTCCATTTCATTTAGTTAGATAGTTTCATAATCAATATCAATCATTATGACTGGCCAAATCATGAATACAAAAAATATCCAAATACCAAATGCGTCCTCTATATGACCTTCGTGAAATATAAGAATCTCTTGATAAGATCAAAGAAAGAACTTTTTCTTCCTTGAAAAAAAATTCTTCCAAAAATTCTGAACCTAATCTTTTCAAAAAAGCGCGTACGGTGCTTTTATGTTTACGAGCCAACGTTCTAGCACAAGAAAGTCGAAGTATATACTTTATCCGATACAAACTTCCTTTTCTTGAAGATCCGCTGTAATAATGAGAAAGATTTCTACACATACGCCCAAATCGGCGAATAATATCAGAATCTGATAAATCAGCCCAGGCTGGCTTACTAATGGGATGTCCTAATAGGTTACAAAATTTTGCTTTAGCCAATGATCCAATCAGAGGCATAATTGGAACTAGGGTCTCAAACTTCTTAATACCGTTATCTATTAAGAATGAATTTTCTAGCATTTGACTCCGTACCACGGAAGGATTTAATCGCACACTTAAAAGAAAACCCATAAAGTCAATGGGCTGATTTGATGATTGATTGATATAGATTCTTCTTGGTTGCAACCATAGGGAAAAATTACATTGACATAAATTGACAAGGTAATATTTCAATTTAGTCATCAGAAAAAACGTCCCTTTTGAAGCCAGAATCCATTTTCCTTGATACCTAACATAATGCAGAAAAGGATCCTTGAAGAACCATAGGATAACCTGAAAATGCTTAGTAAATACTTTGACAAGATGTTCTAGCTTTACGTAGAAATAGACTCGTGCAAGAAAGGTTCCGGAGGATGTTGATCGTAAATGAGAGGATTGGTTGCGGAGAAAAACAAAGATGGATTCGCATTCACACACATGGGAATTATATAGGAACAATAATAATCTTTGATTTTTTTTTTTTGAAAAAATTGAAACAGATTTCTTTAGAGTAATAACATTATTACAATACTCATAAAGAAAGAATCGTAATAAATGCAAACAGGAAGTATCTTTTACCCAGTAACGAATAGTTTGAACCAAGATTTCCAGATGGACAGGGTGAGGTATCAATATATCTAACACATAATTTAAACGTGAAAATTTGTCCTCTAAAAAAGGAAATATTGAATGAATTGATCGTAAATTTTGAGATTTTTTTAGTTCTTTTCCTTCTAGGGAAGATATTAATCGCAGAGAAAATGGAATTTCCGCAATAGCTGCAAATCCCTCTGAGATCCGTTGAGAATACAAATTTTTCTTGTGCCCAAGAAATTCATTTTTGTTAGAATCATTAACAGAAAGAATCAAATGATTCTGTTGATACATTCGAATAACTAAACGTTTTACAACTAGTAAACTGTATTTTGTGTCATAACCCTTATTTGTATTTTTTGTATTTGAAAAAAAAATGGACCTATTTAAACCTAAATCCTGATCATGTACAAGTGCATAAATATATTCCTGAAAGATAAGTGGATATAAAAAATCGTCTTGCCAAGATCTATCTAGTTCGAAATATCCTTGGAATTCCTCCATTTAAAATGAGACCAGAAACGGAAAGTAGAGGGTTTCTTGGGTTATAAAATGATACATAGTGCGATACAGTCAAAACAAGGTATTCTATTACAAAATAATAGATACCTCGGAGACAGGTAAACTCATCAACGGACTCTCTATCTTTTTTCCATCTAATTGCTTTCTTTCTTTATAGTTGATAATAAGATGGTTAGAAATCCTTTATTTTTTCAACCCAATCGCTCTTTTGATTTTGGAAAAAAAGTATCCTTATCAATATACTGTTTCTTCTACACATTCATCTCCATTTTTTTCTAATGGAAAATGGCTAATAGTTAGGATTCACTAAAAAATCGGTAATCCACTCCTGGGAAAAGCCTTTCCCACATCAGTCACTAATATATTTTTAACGTTTAATTAGGGCGGGTAATCGTTCCAATTAAGAACATAAGCTCGTTGCTTTTTCTTTCCCTACAATTAGAGCCATAGGGCTCGATCCATGTATTTAATCGACCCAACTTTGAATTCATTTTGTTTCGTTCTAAGAATTCAACCAAAGTTTTTGTACCGATCTAATAAGAACGAAATAGTTTCATAATTCTCCATTGATACGACATGCTCTTTTTTCCATTCATTCCTTTCAGGATCAGTCGTGGTCTTACAAACGCTACCGATGGTGTGGACGAATCCCTTGCTTCATCCAAATGTGTAAAAGAGCCTAGCCGCACTTAAAAGCCGAGTACTCTACCGTTGAGTTAGCAACCCGAAGAGGTTATGTAGATACAATCGAGATCAAAATAAAGAAATTAGACAAGACAATCAAAACATTGAATTAGCAAAAAAAGAAAGTTTTCGAATAAAAAAACAGATCAAATGACAATACAAGTGACAATACAAGAAATTTTCAAATAAAAAAGAATTCTAGACTACTTCTTAAATATTCTTATCTATTATATCTATATATAATAGATTTTTTTCGATTTCTCTATCTTTCTACCTCTATATTTTTATTCTTTTTCATTTTATTTAATTATCCATTTCCTTTTTTATAAAAAATTAGGTTTTGTATCACAGCAAATTCAACGAATCTTTGAATAAAGTAAAAAACAAAACCTATGTTTTGTATGTAGGACAAAAAAATAGAGAAATAAATGGATCCGTTTACACTTGAATTATATTTGTTCACTACACTCTTGTCAATATGTATGTTAACAAAAAGAATAAAATGAAAGAAAAAAAAGAATAAATAAAGAATTTGTGTTGGATTGGCACTATCTAAATATCTAAATAAGGTACATGATTAGAAAGGAATGTAGATAGAAATAAAAAAAAAGAAGTAGAAAAAATATCAATAACTATACATCAAATCAAATAAGGAAGCTCCATTCTTTTTCTTTTTTTAGTATAATTCCAAGGAAAACCATCCAATTGAAAGGAATGTCAGAATTGTCTATTGCTAGATCCAATTACTACCGTTAGTGACTTGATCAATATAACTTTCTTCGTTTGTTCACTTGACCTTTCTTCTAGACATCTTATATCAATAAAATCTATTTTGATCATTCATTAGAGGACACAGTCTCCTATGGGAACAATACCAAATAGGTCTGAATAGAGCAAAAGAAGGTGGGAATAATAAAGAAAGGATTATATCAAACTATATACGAATCCCTCAAGTCTCTTTCTTGTTGTATTTTATTAAGTATTAAGTGAATTTCGTTTCATTAGTGCGAAGTTCTTTAAAAACCTCTGCCTTCTTTAAAATATCATAAACAGTTCCAGTAGGTTGAGCGCCCCTTTCAAGAAAATATAGAATAGCGGGAACATTTAAATAAGTTTGATTCTTTATCGGATCATAAAAACCAACTTTCCGAAGATCTCTTCCTTCTCTTCGGGACCGAACATCAATTGCAACAATTCGATAGACGGCTCATTGGGATAGATTATATGAACAATACCCCCCCTAGAAACGTATAAGAAGTTTTCTCCTCGTACGGCTCAAGAAAAATGATTTTTTATTTATTTTTTTTTAGTTATATATAATTAATGGCAAATAGATCCATAAAATCATCAAATTAATTAGACTAAGAATTAAGTCCCCTTTTGCTCTTATTCTTCTTTCCGGAAAAAATCATTTGCACTCATAACTCAAGTTGAATAACTCTCAAATAACCAAAAAGAGAATCCTTTGGCATTTCTTTTATTGAGTGGTCTCTAACCCCCTTTTTTCTGGCTTATTTAACCTCTATTGGAATTCTTCATTCTAATCCAGTTGTTGATACAATTGAGAATGAAAAGGGTTTTTCCTTGTTTCGGAATCTCTTTGCTTTGAATCATTAGGTTTAGACATTACTTCGTTGATCTTTAATCCTTTCAAAATGGCAGCAACATACCCTTTGTTTGATTGTTTATCAAAGAAAAGAATCATACAAACGCTTGATTCTCTCACGATATACTTTACTTTTTATCGAAAAGGGTTTATCAATTCCAACCAATTTTCCTTGTTGATTGGAAACTTGGTGGGATTGGATCCTGTCGATTTCTATGTCAAAAATAGACTTACGAAGTTTTTCTAATTTATTGATTCACACTAACCCTAGATTCTTGCTCTTAAGAAATGAATCAATACTTTCTACTCGAGCTCCATCATGTACTAGTTTATTTTAAATTAACTACAACCCAATAAAAAGTGTGGGTTCTAGTCTAACAGAACAGGGGATGTCGAGCCAAGAGCACCTTTTTATATGGAAAATGATGGATATAAAAATCCACACCAGATCATGTCCTTCAAGTCGCACGTTGCTTTCTACCACATCGTTTCAAACGAAGTTTTACCATAACATTCCTCAAATTTGGAACCGGTATGCAATTGATTCAATTATGGAATCATGAATAGTCATTGGTTTAGTCGGTACATAGAAATCTATACCTATAGTTAGACTTTATTCTATATTAAATAAATATTTATTCTATATTAAATAAATATTAAATATACATATTATTTACATTAAATTAATACATTAAATGAATTAAATTATAATATTAGATTATATACATATATATAATATCCATATATTATTATTATATAATAATAATTTTTTATTTATATTAAAATTTTTATTTATATTAAAATAATATATGGATAGAATTATATTAATTAAATTCTATCTAATTGAATATTAATAGATTTTCTCTATATTTTACTATATTTTAGTATCTTATATTTCTGTTTTATAATCTTATATAATACTATATTTATAATAATATATATAATAATAATATATATATATATTTATAATAATCCAATCCATTTAGAATACTATATTCTATATTATAGTAATTTATATTAGTAGCATTCTATTATAGGATTATAAATCGATTATTTATAAATCGATTATAAATAGAATAAATATAAGTTATATAGAAATATAAGACATAATATAAGACATATAAGATTAAGTAAGTGAATAATTGAAAAAAAATTCCAATAATGATGTAATAATGATGTTGAATTAGGAATTTTTTTTACATTTACAATAAAAGCCAAAAAGAAATCCATTTTTTTCTCGAACTCAGCCATTAGCCATTAATGATTTAAATAAAATAAAACCGATAGGTATATCGAAATAATAACTTGGAAAAACAAATCTGATTTTTTTTCACAAAAATCGCAAACCCTTGTTACTGAGGTCAAAGGTTATATAGATAAGATAGAAATATTTCCTCATTTTATACGTTACGTATTTCTGGGTTCAAGAAATTTTCCATTAAGGTGAATAAAATGTCTGAATCACCTTTCCTTTCAACCATTACATAGATTTATACTTATTCATTCGTTATTAAATAAAGAACAAAATACGAAAGACCTAAAAATTCAGTTTCAAAATACATATGTTACATATGTAACTAACTTAATTTTTTTGGTAATTAGATTCGAAATGAGATTCGGGTAAATATGAAAATTGACACCTTTGATTGTTGATTAACTCCTATCTACTCCCCCAATTCCATAGGTATCGGGAGTCATAACCCTCCAAAGCACCCTTTTTATTTACTTTAAAAAATCATAAACTGTCTAGGTACAAAACGAAACAAAACAGACCTAGATTAAATACCTGTTCTTCCTTGTTATTTTACCTCAACATAGTTACATAGGAGCTTGAATCCTATTGATTGAATTCAATAAGTACTAAAATAGACTCTTGTTTCGAATACATACATATACAAAATACGGAGAATTAATAATTGAACTGCCTCATTTAAGGGATAGGGAAGAATTGCTTTCGCATTTTGATTATGAATAGGAATGCATCCTTGAGAATTCTATTAATATTAACTATTATTAATATTAAATTGAATATAGACACACGACATAAGTAACAAATTTCCTTCCCTATTATGTCCGTCTGACCCTTTTTTTTATTGTTTTTATTGAAATTATTAGAATCAATCTATTATCCTATCTTGCCTGTATTAGATCACAACTAGATTCAGTTATATCTGTGTGTGCTTTGAACTCAATTCTGTTTGTGAAAAAGATAGAATTCAGAAACGAATCTTTAAAAAAAGCGAAAAAAGAATAAAATTCTATCTAAATCAAATTCTATCTATATAATATTAGACTACACTTTGTTTTACAAACAGCCCCTTGGTAAAAAAAATATTTTTTTATGATAGAATCCAGATGCTCTGGGACGGAAGGATTCGAACCTCCGAATAGCGGGACCAAAACCCGTTGCCTTACCACTTGGCCACGCCCCACTTAGATTTCTAATCTACACTAATATGGTTATTAATTGTTCGTCAATTCCAGTCCAAATATCTATAGAATCCAGTCGATTGTTATGCGGATTTTCACACGTGTAGATATAGAATGAAACTGAATTTCTTGATCATTACATATAATTCAATTAAGATAATGTATGAAAGTATGATTTATTCTATTCTCTTTTGATTTGAGAATGGAAGGGTTTTTGATTGAGTAAGTTCAAAAAAAAAGAAAGGATTTTTGATCTACTTTGCTTTCTTCATTTTTCGCTTATCTTATATCAATAACTCAATCAAAATGCAATAATCTTCAAGAAAAAAATGTCTGCTATGCTTAATATCTTTAGTTTGATCTGTATTTATCTTAATTCTGCCCTTTCTTCGAGTAGTTTTTTATTCGCTAAATTGCCCGAGGCCTACGCATTTTTGAGTCCAATCGTCGATTTTATGCCAGTCATACCTCTACTCTTTTTTCTATTAGCCTTTGTTTGGCAAGCTGCTGTAAGTTTTCGATGAGATTTAAAATCTTGTCCTAGAAAAATGAATGATTTATTCGATAAAAAAAATTCTAATACGAATATACTAATAAATGAAAAGATCAGATACGGCTTATAGTATGAACTCTCGATTCAAATAGAAAAAGTCTTGGATAGCCTCGAAAAATTGAAATCACTTCCTTTCTCGTTCTAACAAAAATTTCCGTGAAAGACCCTATGAGGTCTTCCACAAAAATTTTGGGTAGGAAAGCGATTTTTGATAAAAGGGAGGCTCCTAACACTTAACAAATGAATTCATTTTATAAAAAGAAATTTCGATTTCCAGAAACTACTTAAATTCTCGGTGTCAAAATAGGATATATGGGAGAATCTATTCTCTTTTTTGCACAAAAAGATCTTGGAGATTGTGTAATGCTTACTCTCAAACTCTTCGTTTACACAGTAGTGATATTCTTTGTTTCTCTCTTCATTTTCGGATTTCTATCTAATGACCCAGGACGTAATCCTGGACGCGAAGAATAAAAGAGGAGTTTCCTTACTTTATTTTTAAGTATCTGATTTTTTTTTTTATGAATAAATCAAAAGAATTCAATAAATTCGAAAGAAAGAGAAATCAAATAAAAATAAAATAGTAAATCAGCAACAGAAACGGAAAGAGAGGGATTCGAACCCTCGGTACGAATGACTCGTACAACGGATTAGCAATCCGACGCTTTCGTCCACTCAGCCATCTCTCCCTATTGAAAAAAAGTAATTACAAAAAAAATTACTAATTACTATAGTTAGATTACACATAGCGTGCCAATTGAAAAATATTATTATTATTATTTAATATTATTTTCTATTTTTTCTAGGTTTTCAATTCGAAATTCTTTAAGATTCTAGAATCTTATAAATTTTAGAGAATAATTTGTAGAATAATTAAAACGAAATAGAAGTCAATTTTTTAAAATAATTTAATTTATTTAATAGAATTCGAAAGAATTAGAAAATAATAATTCTATTCTATAATAATTATATACTCTATACTATTATAACTATTAGATTCGATTTTTTATAATATTCGAATATTATTATATTTTTATATTATATTTCTTTAATTATAATTAAAATAATGAAAAATTATAATAATTAGAGATTAATTATAAATAATTAATATAAATAAAAATAATTAATATAAATAAATAATTAATATATAAATATTATAAATCAATACAAGATATACTACAAGGTTGATCCTAAATTTCAACTAAGGTAATAAAGATTCAATAAGTAAAGATTCAATAACAAGAAAAATTAAATCAATGTAAATAAAACCAGAAATACTTCTCCCGAAAGGCCTTTTATTCCCACGGCCTGGCCTGGTCAATACCTCGCCGGGCCTTTTTTTAGTTCAACGGATCATAGATATAAAATTTATTGCATATTATAAGTATATAATCTAAATAAGTAGAAAATAAGTAATAAGTATATAATATAAAAAAAAACGCTTGTTATTTATTGAAGCAAGAACAAAAAAGGAATGTTTCTAGTCTTTCGATATAGAATCAAAATGCCATTTCTTATCATCTTTTTTTTTTCAAAAAAAAATAAAACTAATAAAACTATAGGTTCTTGCACAATTCGTCTGTTATGACTTTAGCAATTTTGTTGAAATGTATCCGTCAAAACTCTCCATCAAAAAATAGAACTTCGTGCTTAGTTATTTAAATCTCTTTTCTGAATCTCCTTCTACGAAAAATCTCAATACTCTCATTTTTCATGATTTTTTTATGATCCTATCTTGATTACGTTAAATTTCGTTGTTCGACAAAAGTTCCATTTCGATACAATAATCGCATTGTAGCGGGTATAGTTTAGTGGTAAAAGTGTGATTCGTTCTTTAAGAGTTAAGGGATCCTTCGATTTGATTCCTAATCCGATAAAAAACTCTATTTCTTAAAAGGAATTAATCCTTTACCTCTCAATGACAAATTTGAGGAGAATTGAAAATTCTCGTAATTTGTATCCAAGGATCAATTATAAATTGAATAATTGAAAATTTGGATTATGAAATTACGAAACATAATTGTTTTTGAATTGGATCAATACTTCCAATTGAATGAGTATGAGTAAAGAATCCATGGATGAAGATAGAAAAATGAATTTCTAATCGTAACTAAATCTTCAATTTTTTATTTGTAGAGAGGACATTGAAGCAAAATAAATGGCTAAAAAACGATGACTTTAGTTTACTAGAGGCATCGATCGGCATATTCTTTCTTTTAGCTCGGTAGAAACAAAATGTTTTTCCTCAAGATTCTCTAAAATAGAAATAGAGAACGAAGTAACTAGAAAGATTTTTAGAATACCCATCTTCTAGAGGGATCATCTAGAAAGCGAGTACTTTTGAATGCCTTCAGGCAAAAGCTGACATAGATGTTATGGGTTAATTTTTTTTTGTTCCCATTCTAGATCTCGATCTGGCAATTTCTCCATCTTCCATAAAGGAGCCGAATGAAACCAAAGTTTCATGTTCGGTTTTGAATTAGAGACGTTAAAAATGAAAAATAGACGTCGACTATAACCCCTAGCCTTCCAAGCTAACGATGCGGGTTCGATTCCCGCTACCCGCTCTATATTCTAATTTTTAATGCATTAAAAAATACATTAATTCCTGAAATTCTTTCATCTCACATCACATACAATCTGATTCGAACAAGAAATAGGAAAGTCAAAAGGGAAAAATCGTAATGAGAAGCGTCCATTGTCTAATGGATAGGACAGAGGTCTTCTAAACCTTTGGTATAGGTTCAAATCCTATTGGACGCAATTTATTTCCATATGTTTTTTTCGATATCTATGGGATTTTTTGATATTCTTTTCTATTCTAAAGATAAAGAATTTGAATCAGAAATTTAGATTCTTTTTTTTTTTTATTATTATTAATTTATTATTATTATATTATTGAATTATTAATAAAAATGAAATCAATTAATTCTAAAATTGATTAATTATTTAATTTATTTCATATTAAAATAAATTCGAATTCTAATACTAATAAATAATAGATAATTAAAAAATATATAAAAATAGAATTCGAATATTATTTAATATTATTCTATAATATATAATAATAATATTATTCGAATTATTATTATATAATATTATTATATAAAATAGTATTATATAATATAAAATAGAATCTAAAATAATAGAAATATAAATAAGAAATATAAATAATAGAAATATATAAGTATTTCTTTGATTATTAATATTAAATATTCTATTTTTTTATTATTAAATTAAGATTAAGTTAAGCTAAGAAGGCTCAATTTCTTTATGCTTGTTCCTGAAGTAGAAAACGTTCCATCTGTTCTTGAATACCTTCTTTTAAAAGGGCTTCCGCTTCCTCAGTGAATGTCTTGGTAGAAGATATAATTTCTTGGAACTGTGGTTTATTCGTTTTGAAGTACTTACGTAAGTCATCAAGAAATGGACTTACCTGTCCAATTTCTAATGAATCAAGATACCCATTCGTTCCAGTATAAATAGTCATTATCTGTTCTTCCACCCCCAGGGGGGAAGCTTGGGATTGTTTGAGCAATTCGCGTAATCGTTGACCTCTTGCCAATTGATTCTGAGTAACTTTATCGAGATCAGAAGCAAATTGTGCAAAGGCTTCTAATTCTGTGAATTGCGCCAGTTCCAATTTTAATTTGCCGGCCACTTGTTTCATGGCTTTAATTTGAGCTGCAGATCCCACTCTGGAAACAGAAATACCCACATTAATCGCGGGTCTGATTCCAGCATTGAATAGATCGGCGGATAAGAATATTTGTCCATCTGTAATAGAAATTACATTAGTAGGAATATAAGCCGAAACATCTCCGGATTGGGTCTCAACTATCGGTAAAGCGGTCATACTTCCTTCGCCTAAACGCGAACTTGATTTAGCGGCTCTTTCCAAAAGGCGTGAATGCAAATAAAAAACATCTCCTGGATAAGCTTCACGACCCGGTGGTCTTCGTAATAGAAGAGACATTTGTCGATAAGCCTGTGCTTGCTTGGAAAGATCATCATAAATGATTAAAGTGTGTCGTTCACGGTACATAAAATATTCAGCCAGTGCCGCTCCTGTATAAGGGGCGAGATATTGTAATGCAGCGGGAGAATCCGCAGTTTCGGCTACCACAATAGTATATTCCATCGCTCCCCTTTCTTGGAAAGTAGTGACTACCTGCGCCACCGAAGATGCTTTTTGACCAATAGCTACATAAACACATATTACATTTTGTCCTTTTTGATTGAGAATAGTATCTGTGGCTACTGCTGTTTTACCAGTCTGTCTGTCCCCAATAATTAATTCTCGCTGACCGCGGCCTATAGGGATCATCGAATCAATAGCAATAAGCCCCGTTTGAAGAGGCTCATATACGGAACGTCTCGAAATAATACCAGGAGCGGGAGATTCAATTAAGCGAAATTCCGAAGCTGAAATTTCACCTCTACCATCAATAGGTTTAGCCAGGGCATTTATAACACGACCCAAATAAGCCTCACTGACTGGTATCTGAGCAATTTTTCCTGTTGCTTTTACAGAACTTCCTTCTTGTATCATCAAACCGTCACCCATTAATACAACACCAACATTATTTGATTCCAAATTCAGAGCAATGCCTATTGTACCCTCTTCAAATTTGACTAATTCGCCTGCCATTACTTCATCAAGACCGTGAATACGAGCAATGCCGTCGCCCACTTGAAGTACGGTACCTGTATTTACAATCTTGACTTCTCTATTATATTGTTCAATACGTTCGCGAATAATATTATAAATTTCATCAGCTCGAATGGTTGTCATGAGTCTTTCTTAAATTAAATGAATTCGTTTTTGGAAACAAAAAAAATAATACCTTACCCGCAGTAGAAGGACTAATCAGTTATTTCTTTCATTGCACCAAACATGCCAATATTGGCGTTGATGGTACGTAAATGTAACTCGTTGCTCAAACAACTATTCAGGGTTCCTAGAGCTCCTTGTAAGGCTTGTTGGAAAACCCGCTGGCGGACTTGATTAATCGCTCTTTGTTGTTCAAAATGAATGGTTTCGTTTTTGTAATTTTCTAATTGTTCTAAAGTTTTATAAGTTGAATTAATCAAATTCAATTTGTCTCGTTCTATTTCAGAATACCCATTCACTCGAAACTGATCTGCTTCCATTTCTACTTTCCGTAAGCGAGCCCGGGCTTTTTCTAGCTGTTCAACGGCCCTTCCGCGTAGTTCTTCTGAATTTCGAATAGTATTCACGATTCGCAGTTTTCGATTATCTAATAAATCACTTAATGAAAGTAGATTATCTTTCCATTCATTTCAAAACTTTCATGATCCCTTCCCGAACCAAACTTGAATCTTTCGATTCATTTGGCTCTCACGCTCAATTACTTCAAAATTTTTATGGTAAATTTCCATATCTTTTTTTGAATGTAATGAACCTATCCTCTACTCTTTGTTCATATTCGAACAAAATTGGAAATGAATCAATAATCCAAGGCCAGAATATTTGGAGGACTCTTCTGACCAAACAAAAAAATATGTAATTGTCAGCAAAGTTGTTTTTTTTTTCAAATCCAAAAAAGATTTCTTATTTTATATTTATACATAGGTCATCGACTCAGCATTTGGCATTTAAACAAAAATGTAAAAAAAAGGATGAACATTTTTGCAATACCAATAAAAGTTTCAAATCTTTTTATCGATATGAGTGTTATATATCGATAAATTTCTAACTATTCCTTGGAAATGGAAAACCATTTCAGTATTAACATAGTGGTAGAAAGAGTACCATGCTGTGGCTGAACTTCAAACGGTTTAGCTTTAACCATGTTAATGGTTCCACATTATTGGTTGCTAGAGAATCAAAGTATATTTACCAACGAATCACGAAATGCTATGGTTCTTACATATGATTATATTATTTCTTAATTTATTCAGAAGTAATTCGCGAGATCATGCACCTTTCTTTACTAGTTATACCGAAAAGGGGTGCAACTGGTTGAATCCAGTCTATTCTTGAAATAAACAACTCGCACACACTCCCTTTCCAAAAAAAATCAATACACCAATCACTACACTTAGATTTATTGGATTTGTTGCTAAAATATCGGTATTCAATCCGAAACTCCCGGCAGATGGCCAGTGACCCAGGGAAACGAAAGAATCGGTTACATTTTTCATATGATCTCCTCTTATAGATAGACTAAAAATTCTTATAGATAGACTAAAAATAGATAGACTAAAAAATAGAACATAATTTTTTGTTGTATTACTTGACCTATTTCCTATTTCGAAATAGAAAATAGATTCAAAATCTATTCCATTTCACAATGTATTTTCTTTTTCAATTGTCCAATAAGAATAAGACTTATTCGAATAGAATTAGGTACCAGGTTTTCGTGTAAATTGCGAAATACCTCCTTTGTTGCACCATTTCCTAAAGAGCTTTCGTTGGACTAAGAAGGGGGAAGGAAGAAAGCGAGTCGGTAACACTAATTCCTCATCCTCAAATCCGCCCTTCCCCCCGGGTTTTCTCAACGAATAAGTAATTGTAGGAGCGAAATCCTGGTATAATGCGAAAAGGCAAGCAGGCAAGCGTCAAGTCCAAAGAGATAAAAAAATACGTATTTTTTTCGGATTAGGATTAAACAAAAGGATTCGCAAATAAAAGCGCTAATGCTACAACCAATCCATAAATTGTTAAAGCTTCCATAAAAGCCAAACTAAGCAATAAAGTACCTCGTATTTTACCCTCTGCTTCGGGCTGTCTCGCAATACCTTCTACAGCTTGGCCTGCAGCAGTACCTTGACCAACTCCAGGTCCAATAGAAGCAAGCCCTACAGCCAATCCAGCAGCAATAACGGAAGCGGCAGAAATAAGTGGATTCATGATAAGTTCCTCGCGCAAAAAAAAGAAATGGTTAATGATACAATCAACGAATAAATTATGACTTAATTATTCCATCAACTAAGATTCAGCCAGTCGAAGTCAGTAAGAACTCCGAATTGAAATAATAATATTCCATCAGATCATCAGAAAGACTTTCTCTTTTTTAGTTCCTATTTGTTGAGTCTTTTCTGAATCTATACAACTAGAGTTTCTCATTTACTTCTTTCTAACCATTCTTTGAATTCTTCGACCCTTTCTTGATTTTATTTGTTTTTGTTTATTCATTCAATTCATAGTCATCAATAAATGAAAAAAACATAAAAAAAGACTTCTATTGATATCCCCCTCTAAAGAAAAGTAGGGCTTAATATTAGTTCATATATAACTAGTCAATATCTAATATCCAATATACATGTCTTTCTTCCATAACGTAAACCCAGTATTCTACCTTAAATTCAATTGGATTCTAGAATCATTCTTTGAATTGAAACGTCTACAAGAGTTGACTTATAGCCATTCGATTCCATATACCTAGTTCGGCCTTTCTATACTAACCAATCCCCCCTCTAGTATCCCCTTTTCTATTACTATAGAACATACTTGTATGTTCCCTAAGTAGATTATCTTGAAACACACATATATTGACTTGATCTAAGAAAAAAGACTCTTTCGAAAATGAATTAATGATGACCCTCCATGGACTCGCCTATATAAGCTGCGGCTAAAGTTGCAAAAATAAGAGCCTGAATACCACTTGTAAATAATCCAAGAAACATGACAGGTATAGGAACTACTAAAGGTACTAAAGAAACAAGAACAACAACGACTAATTCATCGGCTAATATATTTCCGAAAAGTCGAAAACTAAGGGATAGAGGTTTTGTGAAATCTTCTAAGATGTTAATGGGTAAAAGAATTGGAGTTGGTTGAATGTATTTACTAAAATAACCCAATCCTTTTTTTGTAAGACCCGCATAGAAATATGCCACTGACGTGAGTAAAGCTAAAGCTACAGTCGTATTTATATCATTCGTGGGTGCGGCTAACTCCCCATGAGGTAACTGTATGATTTTCCAAGGTAAAAGAGCCCCCGACCAATTAGAAACAAAAATAAATAGAAACATAGTCCCAATAAAGGGAACCCAAGGACGATATTCTTCTCCAATCTGAGTTTTGCTCACGTCTCGAATGAATTCAAGGACATATTCAAAGAAATTCTGACCGTCAGTCGGAATGGTTTGTGGATTCCGAACAGCTATGGTGGCTGAGCTTAATAAGATAGCAATTACAACCCAAGAAGTAATAAGTACTTGGCCGTGGACTTGGAAACCACCTATTTGCCAATAGAAATGTTGGCCGACTTCCACACCGGATATATCATATAATCCCCTTAGTGTATTGATTGAACATGATAGAACATTCATATTGTCCTCTGACAGAAATATACCCTTAAAAAAAATATTATTTTGATTCAACCATTTCTTTCTCGACTTGTCTACTTCAATCGTATATAATACCAACTAATCACATCATATCCCCAGTTATTTTTATAGATTTTTTGATATTCAGGAATCCTAACCGATTCTACTCTATTAATTGGAATTCAATTATAGAGTTCACTAAAGTAATTTTTTTATTATGAATCAAGGATTTCTTATATAGCTAGAACGACCTTCACAAATTGCGAATACTAATTTGGTGAGAATTAATCGGATTGAAGCTATAGCGTCATCGTTCGCCGGAATCGAAATATCTGCAAGATCGGGGTCACAATTTGTATCGATTAAACAAATCGTTGGAATTCCCAAAGTAATACATTCTCGAAGGGCTGTATATTCTTCTTGCTGATCAACGATGATTACAATATCCGGTAACCCTGTCATATATTTAATCCCACCCAGATATGTTTGCAAGTGAGATAATTGTCTCTTCAACATGGCTGCATCTCTCTTCGGAAGACAGGCCAGTCTTCCCGCCTTTTGTTCCATTCTCAAGTCTCTGAACTTATGAAGTCTCGTTTCTGTGGTGGACCAATTCGTTAACATACCCCCAAGCCATTTTTTATTAACATAATGACACCGAGCCCTTATTGCAGCCCATGCTACTGAATCAGCTGCTTTATTTTTTGTCCCAACAATTAAAAATTGTTTTCCTTTACTTGCTGCATCAAAAACTAAATCACAAGCTTCTGATAAAAAACGAGCAGTTCTTGTAAGATTTGTAATATGAATACCTTTACGCTTTGCAGAGATATAAGGTGACATTCTAGGATTCCATTTCCTAGTACCATGGCCAAAATGAACTCCCGCTTCCATCATCTCTTCCAAATTGATGTTCCAATATCTTCTTGTCATTTCTACTCACACTTTCTCTTTTTTTTAAGAGATGAGGTATCCCGAAATAAATAATTGTTCCGACGGAACCTTCTCTTCGACGGCGAATTGGCCATTGATACACAATCCAAACCATTAATTCTTTTCTATTCGTTATTATCTTTATTTAATAAATAAAAAAAATGCCCGTAAGAAATACAGAACATATAAATAGGAGGAATCCGTTCTTAAAAATGATTAAATACCCTAAACTAGGGTTTTGATGTATCATTGAAATTTTTTGAAACACAAGAATTAAATAATTCTCTGTGGTAAAACAAAATATCGTTCATTTCTCCCGCGAATAGATTCTTCTTTTTGTTTTTCAAAGGAATGCTCTTATGTTGCCTTGAACGGTGTACTAATCCTTTGAATCCGGTACCAACGGGTATCATTCCTCCCAGAACCACGTTTTCTTTTAGGCCTTTCAACCAATCGATACGGCCCCGGAGAGCAGCTTTTGCTAAAACTCGAGCAGTTTCTTGAAAACTCGCTTCGGATATAAAACTTTGAGTATTCAAAGAAGCTCTCGTTATTCCCAATAAGACGGCTCGGTAAGAGATCGCTTCTTCCAAAGCACGCCCTGTTCGTTCCGCTCGCAACAATCCAATTAGCTCTCCTGGTAAAAAAACATTAGACATTCCATCTTCTGAAACCAAGACTTTTGATGTTATTTGACGTACAATAATTTCTATATGCCTATTATGGATCTGTACCCCTTGGGATCGATAAACCTTTTGGATCTTATTAACCAAAGAGATACGACTTTGCACTATAGTTAGCTCAGCGCCAATCAAGAATCCCCAAGGAAGTCCAAGAATTCCTGTTATACGTTCGTTCCAAGCGTCAATCCTTCGTTCTAGATTCATCGATATTGACTCAAGCGAACGAACTTCTAAGACTTGTTCCACCTTTGGAAGGCCTTGCGTTATATCACCAGACCTCGATTTTTCATATATAAACGTGACTACTGTATCTCCTTCATAAATGATTTCCCCATAATGGCCATGAACAGTTGCTCCTGGGGTGGCCAAATAGGGCTTAGCTGCTCTTATTACTACAGAGTCAACTTGAACAATTAGAACTTGACCGGCCTTTAAGTGTGGCCCGTTTTTGGCTATACATACATTTTCACAAAGAAATTGTCCAAGACTTATTTTTGTGGAGGTCTCTTCACAATAATTGTGATGAAGACAATACCAATTCAATTTGAACGGATTCAAAATAATGTTACTTCCTGGATCGGGATTATAAATCTTCCTATTTTCATCGATTAAATAATATTTCATTACTCGAAAAGTTTGTTTTAAATTGTCAAGTTGAAAATAGTTAGTTACCAAGATCTGATTATGAGTTATTAAACGGTAAAATGAATAAAAATTCGCAATTTGAAAGGCTGTTCCAAAGGGGCCCGACGAATTCCTAATTGGAATTAGAGGATCGGATTCTTTGTAATATTTTAGACCCTTGAATAGACCCATTCGAAAACAATTGGTTGATGACAAAATGATAAAAGATTGGCATTCCTTCGTTCTATTCAACAACGTACGAACAGTTTCATGATTTTGGCTAAATGATTGTTGAAGTCTTGCTTTTGAATAAATGGAATAAAATGGATTCATACGATCTGATCCATTATCAGAAAGCAATCCCGAACCTGATGGATCATTCCTTTTTCCGGCATACGAAATAGTGGATTTCACTAAATCGATTCTTAGGAAATTTCGAATCATACCATTTGTCTTTACTTCAACAAAGGAGGCACGTGCCTCTTCGATAGAAGAACTTTTTTTGTGTTGGTCCCAATTCAATACTAAACAAGTCCGAACTAATTGAATACTTGTGTCAGAAATTCCCCGAATAGGCTTGCCATTTCCATAAAGGATATAATTGACAACTCGAAGTTGCACCTTATCCCTTTCCTGCAACAGATCCTGAGGGAAAAGTGTTGATAAACTTATACCGTCCGTTATTTCATATGTGACTACGGGTCGAACCAAAACAAAATACCTTTTCTTAGTAGGTGTGATTCGTTGGACATAGATCCAATTTTTATTTTTCAAATTTTTCGATTCCTTGGAATTTGTTTGTCCCCTTCCCGGCGGTATCAAAATGCCCCTGTGTCGGGATATCTTATCTGTTTCTCCAGGAAAATGGATATCTCCCGAAAAGATTTTAAGTTCAATCTTTTTTTTTTTCTTCTCTACTCGGACCAATCCGCCTACCCGGCTTCTTGTATTTAAAGTGATTTGTGTATCTACTCCAATGATACTATTGTTCCGTACCATTATGGAAGAAGATCGTGGTAAGATATGTACTTCCTCGGGAATGAAAAAAAATCGATCTACTTGCATTTGGTATTTTGCTCTAAATTCTTTGACTCCTCGATATTCAATCAAACCCTCTTTTTTTATGATTGAATGCGCCTCTATAGTCCCATATTTAGTAATTCCCGAACTATCTCTTCGGTATCGAGGATCATCAAAATAAGCAAGAATACTATTTCTACGGAAACTACCTTTTATGGGTATTTCAATCGAGATACCCGAAGGGGGCATTAATTCTTTCTCTCGTTCTTGAATCGATTGAAATGGAATGGTGAATCTATTTCTTCTCCTCTTTGCCAATAAATCGGAATTTTTGGCAGGATATATGAGATTATAATGTCCAGTTCTTACGCTTCGATTAAGTTCTGAATAATCAGGAATCCTATCCCCTTTCTTACTAGAAAGATCCGAACTCAAAAATTTGTGTCTCATGTGAGCATTGGTCACTGAAGAGTTATAAATATATCTTTGTTCGACAGAAAGAAAATGCGCGTTCGTTTGATCTTGATCCTTGTGGAGCGAACGCGGGGCCACAATGGATTTGTGCGGCCTTCCTGCTAATATCCATAAATGACTTGTTTTTGGTAAGAGATGAACATTACCATATGTAAATTCGGGTGCATGGTCCACGTCGGTACTCCAGTGCATTTCTCCCTCTGAGTCAGAATAAATATGTTTTCGAACCTTTTCTTTAAAATTCAAAGTGGATGTTCCCGCGCGAATTTCAGCAATCACTTGTTCTGATTCTACATATTGATCATTTTGAACTAAAAGAAAACTTTTTGGTGGAATATTCACATTATGTATAATATCTTCACTCTCAATAGTGACAGCCAAGTCTATATAACATAGAAAGGCAGGATGGCCATGACGTGTACGTGTGGGATGAACCAAATCTTCATTGAATTTAATTTTTCCATTAGAAGGGGCTCGTACATGTTCTGCAGTACCCCCTGTGAATACTCCGCCAGTATGAAAAGTTCTTAATGTTAGTTGAGTACCCGGTTCTCCAATGGATTGTCCCGCAATAATACCTACAGCTTCTCCCAATTCGACCAGGTCACCGTGAGTAGGACTCCGGCCATAGCATAATCGACAGATCCAAGATGTACTTCTACAGGTAAAGGGAGTTCGAATAGATATTGGTTGTACCCGAAAGGTTATCAATCGATTGACAAGTCCAACCCCAATATCCTGATTTCTAGCGGCAATGCACCGCGGGCCCATATATATATCGTCTGCTAATACACGACCAATTAGTGTTTGGATAAAAATTCTTTCCGGTATCGTCCCGTTTTGGGGACTCACAGAAATACCTCGGATGGTGCCACAATCTCTTCTACGTACAACAATATGTTGAACTACTTCAACAAGTCTTCGCGTGAGATATCCAGCATCTGATGTTCGTACAGCAGTATCCACAACTCCTTTACGGGCACCGTAGCAAGAAATTATATATTCTGTTAAAGAGAGTCCTTCGCGTAAATTGCTTTGAATAGGTAAATCAATCATTTGTCCTTGTGGATCTGACATTAATCCTCTCATGCCTACTAATTGGTGTACCTGAGAGGCATTTCCTCTAGCTCCTGAAAAAGACATCATATGAACTGGATTATAAGGGTCAGTCATCCTAAAATTAGGATTCATTTCTTGTCGCAAATATTCACTTGTAGCATACCATATCTCAATGGATTGACGTAATTTTTCTACCGCATGGACATTCCCATAATGATGGTGTTTTTCCAAAATCAAACTTTGTTGCTCAGCATCTTGGACTAGCCATCCCTTAGAAGGTATTGTTAAAAGATCATCAATTCCTAATGAAATAGATGTAGCAGTGGCTTGCTGGAAACCCAGAGTCTTTACTTGATCCAGGATGTGTGATGTATATGCCATTCCAAAATGATCTATTAATCTGCTAATAAGTCGTTTCATGGCAATTCCATCTATTACTTTATTGTGAAAGACCAGATTGGCCCCTTCTGCCATAAGTACCTCCATATTCCGCTGAGTGGGATTCGACAATGAATGGGTTTAAGTTAGTGATTGTAAAACTTCCTTTTCTCGATCTTAATTCGCGTAGAAATTCACGAACTATGATCCTAGTTGAACTCGGGCGAACCGAATTCCACCGGTATCATAGAGTTACTTAGCTAGGTACGATACGATTAAGTACCATATGAGCAGGCTCGATAAAATCCTTGTATAGCTTCTTCAATTTCTCGATAAAACGAAATATGACCAACGGTTGTTCGAATGTATATAAAAAGAATTTCTTTTTTTACACTTCTTACTATTAGATAGTGCCCATAAATCTCATGATAGGTACCCAAAGATTCATAGTGAACTTCGATGGGAGCTTCTCTTGAAGCAACAACACGTTGATCTAGTTGCCACCGGAGCCATAAAGGACTATCTAAATTGATTCTTTTTTGACGATAAGCCCCAATTGCATCATAGGAATTACAAAAAAAGGGTTCTTTCATATACTTATATATATGATCGTTCAATTTTTCATTTTGATAGTTTCTTCGACTCCATGGATTATATCTATTTGCACAAATACCTCGACGATTCCCACTCGTTAATACATAGAGCCCAATAAGCATATCTTGAGTTGGTACGGAAATGGGATCCCCAATAGCCGGAGACAAAAGATTCATATGAGAAAACATAAGTAAACGGGCCTCCGCTTGGGCCTCCAAAGATAAAGGTACATGAACAGCCATTTGATCCCCATCAAAGTCTGCATTGAATCCTTTACAAACCAATGGATGTAAACAAATAGCACGCCCTTCCACTAAAATGGGTTGGAATGCCTGTATGCCTAATCTATGCAGAGTAGGCGCTCTATTTAACAATACAGGATGCCCCCGCATAACTTCCTGAAGTATTTCCCATACAATGGGTTCTTTTTCCCGAATTTTACTCTTAGCAACTCCTATGTTCGAAGCAAGATCTTGTCTAATTAGACCGCGAATTACAAATGTCTGGAAAAGTTCTATTGCAATTTCACGAGGCAATCCACATCGATGTAATGAAAGTGAAGGACCTACAACAATGACGGAACGCCCTGAATAATCGACCCGTTTGCCAAGAAGAGTCTCACGAAACCTTCCCTCTTTACCTTCAATTACATCAGAAAATGATTTGTAAACCTTATTATGACCATCCCTCATTGGTTGTCCGCGTATTCCATTATCCAGAAGTGTATCTACGGCTTCTTGTACCAATTTCTCCTGACACATTACTAATTCTCCGGGCGTAGATCTACTTGTTGTTAATAGATCAGTAAGAGTATTGTTCCGATAGATAACTCTTCTATAGAGTTCATTAATATCCGAGCTCATTAATTTACCTCCATCGATCTGAATGATCGGTCTCAATTCGGGAGGAAGAACAGGTAATAGACACAAAACCATCCATTCTGGTTCTATATTTGTTCGAATGAAATGCTTAGCTAATTCCATGCGTCTAACCAAAAAATCCCTTCTTCTTCCAACTTTTCGATCTTCCCATTCATTTCCTGTAGGCCCTTCTTCCCCTAACTCTTTCCATTCTGCCAACGAAGAATCGAGAATAGTTCGCAAATCCAAATCGGCTAATTGTTCTCGGATAGCACTTGCTCCGGTAGATATTTCTCGATTTCGAAAGGTATCGAAGCCTTGGGTAGTAAAAAAAAGCGGGATGCTGTATTTCCAAGATTGGATTTCATATTCGAATAAACCTCGTAATCGTAAAAAAGTAGGTTTTTTAGCTATGGGCCTAGCAAAAGAAAAATTGGGATAGGATCCTATACTATAAGATCTCCCCCCTTCAAAGCCGGACGTGAAAGTTTCCTCTCATCCGGCTCAAGTAGTTACAGCAAAGAAAGAAAGGAGTTCTCGCTTTCAAATTCTAGAAATTCTGGAAAATCCTCAAAGCAAAAGATCTACTCCTTACTCAAGTTCCCGTGGAAGACCAAGCAACATTTCATTAATTCATTCTTCTTTTTATTTAGATTTTTTGAATTCTTTATTCAAGTCAAAATGAAATGTGAAATTCTTGAGTAGTCTACCTCCCTTCGAACGATGAATCCCCTTAAAGGAAAAAAGGCAGTGCCTTGGAATTCATAAGATATAAGATAAAGGATTTACTTGTCTATGTATCGTTCCATTCGATCTTTTAGGTCACGACTTCACCTCGACGGTTAGACTACGATGCCCTTAAAGCCTATATGCGATGGATAGACTCTTGTAACCATGACATATTTTCTATTTGCTTGCGTGAACATAATTTATTTCTAAACGACGGAGAGTGGTTAATTCCACAAACAAAAGAAGTCTTTTTTTACGAGGTACAACTAAAAATTCAAATTTACTTGTTACGAAATCGACCATGGATCAATTCCCTTTTTATTTGGGAGTATTGAATACACCCATAATTCTGAGCTTCATGTTACTTCTACCAAGAGACATGTCAGAGCCAGGGCATCCCAATTAGATGGAATGGGATAACAGTTTCTCATTTCGAATCTGTAAAATCAAAATTTCGATCAAATCACACATCGCAGTATACTAGGCCCTCTAATTCTTTAAGAGGTTTATCTAAAAAATTCGCAATATAACTAGGAAGACGTTTCAAATACCACACATGAGTTACTGGGCACGCCAATTTTATGTAGCCCATTTGATACCTTCGTATTCGAGAATCAACAAATTCGACTCCGCATTGTTCACAAAATTTCGGTTCTTCTTTTTTATCTCCGATTACTCGATAATTTCCACAAGCACAAATTCCGCTTTTTATAGGCCCAAAAATTCTTTCACAAAATAATCCATCTTTTTCCGGTTTATTGGTTTTGTAATGAAAAGTATAGGGTTTTGTGACCTCTCCAACTATCTCTCCGTTCGGTAGGATTTTCGTGGCCCACGCACTTATTTGTTGGGGAGAAACTAATCCAATTCGGAGTTGTTGATGTTTATACTGATCTATCATAGAAGAAAAATTTGAATTAATTCCGATTAAGCTTCCATCCTATTAATCTGCAAGTTCTTCTCAGATACAAGGAAATGATTCAGTTCCAGAGCCAAAGATCGTAGTTCTCGAACGAGCAATCGAAAAGATTCTGGAGCATCCTCGGGGTTAGGTATTGTTCCTCCAATGATCGTAGTACCAAGTACTTCCTGGCGAGCTCTAATATGATCAGATTTATAAGTAAGCATCTCTTGTAAAATATGAGCAACACCAAATCCTTCGAGAGCCCAAACCTCCATTTCTCCTACCCGCTGTCCCCCTTGCTTGGCCCTTCCTCTAAGGGGTTGTTGTGTAACAAGTGCATAATGTCCACTAGAACGTCCGTGGATTTTATCATCAACTTGATGTATTAATTTCAAGATATAAGGATTTCCGATTATAACAGGTTGTTCAAAAGGATCTCCTGTTCTTCCATCAAATATTCTGCTCTTTCCCGGATACTCGGGTTCAAATACCCATGGATTGGCTGTTTGCTTACTGGCCTCATATAATTCAGAAAACACTAGTTTTCGCGAAGCCTCTTGTTCATATCTCTCATCAAAAGGTGCTATTCGATAATGTCTGCCTAGCAAACCTCCCGCTAATCCGAGTGAACATTCAAAAATTTGCCCTACATTCATTCGTGAAGGTACTCCTAAGGGGTTAAAGACCATATCAACAGGTCTTCCATCTTGCAAATAAGGCATATCTTGTCTAGGTAAAATTTTTGAAACGATACCTTTATTTCCATGTCTTCCAGCTACTTTATCACCTACTTTTATTTCTCGTTTCTGTGAAATATATACACGAATCGTTTCCGGATTAGAACTCGAACCCCCCTTTTTCTGGATCCATCTCACATCAATAACTCGACCCCTACCACCTATAGGTAGTTTTAGACAAGTTTCCTTTGAAGTGGATACCTGAATGCCAAGTATGGCGCGTAATAATCTATCTTCCGGGGCATAGGATGATTCTTTTGCCATCTGAGGCGTTAATTTACCTACCAAAATATCGCCTGTCTCTACCCATGATCCCAGCATCACAATTCCATTTTTGTCTAAATTGCGGAGTAAATGGGCTTCTAAATGTGGTATTTCGTTAGTTAACCTTTCGGGACCTTGACTTGTCACATGAGTCTGAATTTCATATTTTCGTATGTGAAAAGAAGTATAAATATCTCCATATACAAGACGCTCACTAATGAGTACAGCATCTTCAAAATTGTAACCTTCCCATGGCATATAAGCTACTAATACGTTTTTTCCCAAAGCGAGTTCGCCCCCAACCGTGGCGGCACCGTCCGCTAAAATTTGTCCCTTTTTAATGCATTTACCCTGCTGAATCTGGGCTTTTTGATGCATACAAGTATTTTTGTTGGAACGTTGATACATAACTAATGGAATGCTTAAAGTATACCCATTACCTGATAAAATGATCTTGTCAGTATCGGTATAAATGATCTTTCCCTCATGTTCGGCTATAGCGAGAACCCCTGAATCTAGAGCCGCTTGGCGTTCCAATCCAGTTCCAACAATGCACTTCTCGGAATGAGAAAGCGGAACGGCTTGACGTTGCATATTCGAACTCATTAAAGCCCGATTCGCATCATTATGCTCGATAAAAGGAATGAGGGAAGCTCCAATAGAAAAATATTGGAAGGGAAAAATACTTCGAAAATGAACCTCTTCCCATGCACTAGTCAGGAATTCTTGACGGTATCGAGCTGGAACAACCCCCTCTTCCTGAATACCTCGATTCAAGGCCAAAGAATTTCCTGCCGCTACCATATAGTATTCATCTCTACTTGGTGATAAATAAAGCATCTGTACCCTTTTTGATTTCTCAGAAATTTCATAAAACGGGCTTTCTAGAGACCCCCAAGAACCAATCCTAGCATGAATTGCTAAGGATCCAATAAGTCCAACATTGATTCCTTCAGACGTGTCAATTGGGCAAATACGCCCATAGTGACTAGGATGAATATCTCGTATACGAAAACTAGCAGTTCGCCCCGTCAATCCTCCAGGGCCCAAATAACTCAATTTTCTCCCATGAACTATTTGCGTCAATGGATTAGTTCGATCCAAAACTTGAGATAATGGATGTAACCCGAAAAAAGATTCATAAGTGGTTGTTAATGGAGTTGAAGTTACCAAATTCTGAGGAGTCGGTATTAATTTATGCCTAATTGCTCCACATATAGTTCCTCGAACCACATTTTCTAAACGAACCAGAGCCAATCCAAATTGATCTTGTAAGAGATCCGCCACAGAACGAATACGTTTATTTTTCAAATGATTCATATCGTCAAGTGTACCCATTCCAAATTTCATTCCAATCAAATGGTCTGCAGCTGCCAATATATCTCGCGGTAACAAAAATGTATTGTTTTGAGGTATATCAAGGTTCAGTCTCTGATTCATATTTCGTCGACCAATCCTTCCTAATTCGCATCTTTGTTGAAAGAATTTTTTTTGTAATTCCTTACATAAAGATTCAGAAAATACCGGGTCCCCACCTACACAAGCAAATTGTTGATAAAACTCCAAAATGGCATTTTCTTTTGATCCAAAATTTCTTTTCTCCTTATCATTCAGGAAAGACAAGAAAATTTCAGGGTAGCAAACATTCTCTAGAATTTCTCTTAGATTCGAACCCATAGCTGATGATAGAACTAGAATAGATATTTTCTGTTTCCTACTCACACGAGCCCATATCCTTGCTTTTCTATCAATCTCTAATTCTGATCTTCCTCCCCAATCTGATATTATGGTGCCGGTATAGACCGAAATTCCGTTATGGTCCAATTCTGACCGATAATAAATACCGGGGCTTTGCAGTATTTGATTGATCACAATTCTGTATATTCCGTTTACTATAAAAGTTCCCAGGGAATTCATTAGAGGAATGTTTCCAATAAAAATTGTTTGCTCTTGCATATCCCTACTGGTTTTCCAAATTAACCCCGCGGATACATATAATTCAGAAGAATATGTAAGTGATTCATACACAGCATCTCTTTCTTTTAGCAATGGTTCCACCAATTGATATGTTTCCACAAATAATTGAAATTCAATTTCTTGATCTGTATCTTCAATCTTGGGAAACTTAGAAAGTTCTTCCGTCAAGCCCTGATCAATGAACCTACAAAATCCTTCAAATTGTATCTGATTAAACCCAGGTATTGTCGACATTCCCTCATTTCCATCCCGGAACATAATTTCCCATTTAATTTATAGAAAAATCCCATTATTAGCGCATTCTTCATCGATCATATAGATCGACCCAACGCCGATGGAATCTATATTCTGTTTACTGAATGACATAAAATTTTACCCCAATTCCATACCAAATCTGTCCATATATCAAATGTATGAAATACGTATGAACGGGGGAATAGAGAGAATTTTCTACTCAAGTAAAATTTTGGAATTGAATTTGTATTGTAAGAGAAGAGATGAAAGGAATTGATAAAACATTCTTGGAACCAGAATTCTGCTGCTTAGATTTATGTTATGGGCTTGACTAGAGAATATCAGAACAAAAGTGATTCAATTACTACTTTTATATATAATTAATATAATGATATTACATATTCCAATCCGATTGGATACCGGAAAAATAAACGGATTCGGGATTTGATCTGTTCGCCGAGATAAATACAGAATAATGAGAAACAGTACAACGTTGATTTTTTTTCTCACTTAACCCCTTGGGGGATTCCGTTGTTAAAAAAAAATTGCCAAGAAAAGAAAGAGAAACTTTGACCTATTCTCTTATTATTCTTAGATTATTACTTATTACTAGACTAGAATTCGTAAAATACGCTTGGGAAGCGGGTTGTATTTAGTTGTATTTATTAAACATGTGTAGCTCTCTTCTATATATCCCTATATATCCATCTCCCCTTTTATTGCAGTTCTATTCGGGGCAGCACGGGCGGTGTTCTATCCAAATTTATACTTTTTTTCGTCAATGAAAAATTGAAGTACGATATTTTCTGATAATTCCCTACGGGCATAATATATTCTATTTGAATACCAGATTATATATCTGTGTAATTTCTGTTCTGGTTCCGAGGTTTGCTTTACATATATTCCATAACATATATAATATAAAAACTTTACATATATAAATAGAATATAAAAACTTTACATATATAAAAATAGATTTATATAATCGATATAATCGAAATTGAGAAAAATGGAAGTTACGAAAGATTTTTGATTGAAAAGAATCAATAATAATTAGTTATTATTTGGACTTTCTTATGTCATTAGGGAAACATAATTTGAGATCCAAATCTAAGAATCATTCATGAATTCGCAGTCAAGTCACAAAATAGTTAATGGTTCAAATTTATCATGAATTTTTTTTGTGACTGAAAGTCCACATTTTCCTTTTCAATAGAAAGGATAAGGGAAGTTTTTAGGTATTGCGTATTTTGCGATACTATACAATCAATCGAAGGGGTGGGTCTAATCAAAAAGGGGAATGGTTTCTTTTGGTTAAGGCAAACGGGATTCAAGATGCAAGTACAAAAAAAAGAAGTTCAGTAATCCACCCCAACCCAAAAAGGGGTAATATTGTCATCTATTTTGTTTGTATTTTTGGCGACATGGCCGAGCGGTAAGGCGGAGGACTGCAAATCCTTTTTTCCCCGGTTCAAATCTGGGTGTCGCCTGATCAACAAAAGAACGGAAATCACTTTTTTATTGATTTATCGATATAACTCACCGAAATATTCCCCAGCAGAGGGGGAGGAGGGAGCTATTGATACGCGCTTGATTCGAAGCATATGGAGGTTCTCGAAAGATCTGTCAATTTTTGTGTCTAGGATTCAAAAAAAGATTTTCGTACTATGAAGAGAGTCGAGAAGTCTTGATAGCCCTTACACTACTAATGGAATTTTGACTGACTGGGCCTGGAATTAGATTGGCTAACCAAAGGGGAGTCTAACTAATTAGTAGAATTGTGGAAAAGGAAGAAGCTACTTTGGTCTACAAACTCACGAATGTCTTTGAGTACTCAGATATTCGATCAATATTTGATTGTATACTTCAGTTGTATAATTCAGTTTTATTTTATATAAAATAAAAACGTGCAAAAAGAACTTCTTTTCAGTAACCCCTAGTCAAGAATATAATCGACTGTCTCCCCATTTTTCACAGAGACGAAATAGATCAAATGGGAAAGGAAATAAATAAAATAGAGGTATGTGATAGATAATGATCTATCTTGGTTATATAGTTTTTATTCCGTTTTTTTATGAATGAATTATGAAGGTTAACAAAAGAATAGGTCTTTTTATTCCTACATGCTATATTAGTAAGACTCCCTTGTCCACGGGAGTCGTTGCATATTTTGCTTGTGCTTAATCTTTCCCAATTATACTAGAAATCATAATGATAAGAAAATTTGTATAAGAATTTATTAGAATTTATTATATTACAAGTGATAAGTGCATTTATTGGTTTATTGGATTGGTTCATTAAATAAAGAGTTTGGGGTAAGAATCCCCTTTTGACTATGCACCCTGGATTTCACTATTATTAGTGAACAAGAATGGAATAATTCCTTCATATTCATAGAGATAGGGGACATAATTCACATGGATATAGTAAGTCTCGCTTGGGCTGCTTTAATGGTAGTCTTTACATTTTCCCTTTCACTCGTAGTATGGGGAAGAAGTGGACTCTAGAAGTACAATTAATGTAATTGCGGTAAGGAATCAAACTTTATCAATTGTTTTATAGATCATTCTACTTCTACAAAGCGTTTTGTTTTAATTTTAACTAAAATAATGTCAATCAAACAGATATTTCAATGATTCCCATGTTTGTATTTCGTAAGGGGATATGGATGGTAAGAAATTTGAATTTTCCTAAATTCTCTATTTCGCCGAACGGGCTCTTATCAGACTTTCTTATCAGACTTATATAGAGACAATGAGGAACAACAGACCACTTCTTATTATTTGTTATTTGTTTCCCCAAGAAAAAGGCTTAATATTATTAAGCGGATGCGTACTTTCTAGGGTAAAGAACATATACATAGTGGTTGTTCAACAAGATACTACACATAATCAAATCTTGCCCCGGCCGAGTCACATATTGTGTACTCACCGCTTGCTTTATTGTTGTAGAAATTGGATTTATGCTTTATCGACATCGACTCATTTCATATCATGGTTCAAGTGTTCCAAATTGGTAGGGTTTACTACTCCTTTTCGAAATTCGAAGAAGTTCCCATACTCCTTTCTGTTAGCGGTTCAATCAAGTACTTCTTTGGAATGCTAAAAAAAGATTACTGGCTTTTATTAATAGGCGCCCTGCCCATAGACTTTTTACTTCTTTGATTTTCTTTTTTCGATGGATACTGGGATTTCGATTTGAAATAATCAGAAATCTCGGAATTCAAGCCGTAAAAGTAAGAGTTACCTTTTGATTATTTCGGATTGATCGGAATCAATACAAATATAAAATAAATGTAGCAAAAAAATAGAACTGGGGCCCTATGTATCTTCTATAGATAGAATTCTATCGATATGAATATAGATATTGTAGATTGATCTATATTAAGCCCGTATCTTTATACAGTACAACTTTATACACTACAAAACCGCTAATCTAATAGATAGTATGGTAGAAAGATTGATATATTCCTTTCTACCATATTATCAAATCTCATAGAATACTGTTGATTCTAGCCTGCGTATTTAATTGAAGATTTAAGAAAGGAAATTGGAATCCTTTGTTTCTTTCTTAAATCATTATCCTTGATAAAGACCTAAGAAGTCAAGTTTCATTCAGATTAATCATTTTGGCTGACCGTTTTTACATATATGATAAGTAAAAAAGCAGTAGGAACTAGAATGAAGAGTGCAGTAGCAATAAATGCGAGAATATTTACTTCCATAATCTCATTGGTTTTTACTTCGCAATAACTCGGGATTTAATCCCATAGAGATAATAAAAATTTCGCCTGTAAATTCAATGGGATGAATTCCATCTCGATGATATTGAATCGCATCAATATTATGAATAACAATATCTGGGCTATCAAATCACTTCCTCGTCGCGAATTGAATAGTATAACATAGGAAGATCTTTTATCCATACTCAATAGAAAATTGAATTTGTAATCGAATCAAGAAATCTTTTTATTTATTATTCTTTTACATTCTTTCCTTTCTACAACCTACCGTCTTCCTTGGACAATCATCGGATGAAGTATCATCTGACCGTTTTCTACTTACATTGCATTCATAACAAACCCCACAAAAAAATAACAATAGAAGTAAAATGAAAAAAGGGGGGAGAGGAGTTAAGCTCGAAACTCCTATTTTTTTTATGATATAATTTTTTTATGATATAATTTTCTTTTTCTTGTAAGAAAAAGAAAAGTGTGAAAAAGCCAAATTCCGGTATAAAAGATCTAATCTTTTATCAAATTCTTTCATTTTTCATTTTAGGCTTTGTTCTTTTTTCGATAGCACCTTTACTTTCACTTTCTAACTTGACTTTCACTTTCTAATAAAAAGGAAAAAGATTATTCATTACCTCACTAATAAAATAATAAGACTAAAAGGGGGTGGTTGTTTGATTTTTCTTTTATTAATATTCTCTTTTATTGGATTAGTACTAGCATAGATTAAAAGTTCGGTGTAAAATTTGAATGAGATAGATTTTAAAAAAGGAGTTAGTTTGAGTCATTGAGACTACACAAAATCGGAGCTAGAAAGGGAGAGAGTTTGAATCTGAAAAGTTTTTTCGGGGTAACTCAAATTCCATTTTTTTGTAGTGTACAAGAAATGAATTCTAGTTGTGTATGTGCTCCCGAGAAACATATGATACTCTATTCCATTCGATAGAGGCCATAATTTGAAAATTTCAAGACCAGACCCAGTACGCTATCTCTTGGAATCCTGAATATGATGTTCCCAATAATTGGACTAATCCAATTCTATCTCTCTCCCACCAATCGGTACTAGTTGAAGTAATCAAAATTTCTATATTTGTTTGTGTTTGATGAGAAATAAAGAAAAAAGAAATAAAAATCCCTATTGAAAATGACTGAAATTCTATTCATTGTGTCTCTTTTGCCAGAAAATGAAAATGGAGAGATGAGTTGATGTGTTTATTGGATCCGTCGGGACTGACGGGGCTCGAACCCGCAGCTTCCGCCTTGACAGGGCGGTGCTCTGACCAATTGAACTACAATCCCAGGGAAATAAGGTATACCGCATCAATATTTTTAGGATTTCATTCAAACCCATTTTTATTTATCATTTTCGTGTTGTAACAGAGACACGAGTGATATAGTGATATCTACATGGCTATGCACTTTCTTTTTAGTGATAAGTGATAGCGACACGAATTACATTACTAGTGATCCTTTCCTTATTTCCAAATCGATTGATAATCAATCTTTCAATAAAAAAATTTTTCTTTTCTCGTTTCCTTAGACTTTCTTTATACTTACAGATACTGATATGAATCTAGATCATTATATTATATAGATCCGAATTTCTTGGAACAAATAAATCGAGCAAACAAATAGAGGTAGGTATATAGAAAAAAATGGAATTCTTTGATTCCCACGTATCATGCGCTTCGGGAAGACAAATATTTGAATCTCGCGGTCTATGAGCGAATTCTTGGGCCGAGCTGGATTTGAACCAGCGTAGACATATTGCCAACGAATTTACAGTCCGTCCCCATTAACCGCTCGGGCATCGACCCAGGAAAAATCCATTCAATTTTCAATTTTAGGCTTATTGATAATGCACGATCAACTTCCTTTTGTAGTACCCTACCCCCAGGGGAAGTCGAATCCCCGCTGCCTCCTTGAAAGAGAGATGTCCTGAACCACTAGACGATGGGGGCATACGTGTCCGACCGCCATCATACTATGATCATAGTATGAACAGTTTTTCCAAATTGTCAATAGAGTCAATAGAATGTAAGAATATGATTCGATCCAAGGGATCCTTCCGCCCTTCACGATTCCATAGAGTTTTTTGATTCGTCATTCCTATTTATGAATCCTTAATTCTAACCGCCATTCCATTCGATTCGATATATAAAGCCGTTATCATTATTCATTATTCATATTTTATTTAATTTATTAGTTCTATTTATTAGTTTATTAGAATTTATTTATTATATTTTTTAAATTCTATTTAAAATTTTTTATTTATTTTCAATTTCTTTATATTTATATATTTATTTAATATATTTCTTATTTTCTTATATTAAATATTATATTAATTTTTTTATATTTATTATTATTATTATTTTATTCTATTTTCTTATTTTTTTATTATATTAATTCTATTCTAATTAGAATATTTCTAATAGAAATATTCTATTCTATATTCTATTCTAATCAATATTCTATTCTATTCTATATTCTATTCTAATCAATATTCTATTCTAATCAATATTCTATTCTAATAGAATAGAAAAACTGAAAAACGAATACTAAATCTAAAAAGAGAATATCTAATCAAATTCGATCGACAATTCTATCTAAATCGAAATTAAATAAAATATGAAATAAATAAAAATAGTAAAAATTCGATTAAATATCTATTTTTAAAATAAAAGCATTTCTTATAATTATAAATAGAACTAAATAAAATAAATAGAACTAAATACGAAGAAAGGGATCCCTTTGTAGTTATACACCCGAAAATCAAAATACAACTTTCAACTCCATTTCTTCCACTTTCATTGATTCATTCATTTTTTTTAAGACGAAATGTGCCTATCTCTATTTCTCTATTTCATAGTAAACCAGAAATTTAATAAAGGAGAAATCTGGGATGATAAAGGGGATCAATAAAATCTCGGAAATTTTTTTTTATTGATTAAGGGGACAAATCGAACTTCTCCACCACATGATTTAATGAAATATCTTGGATCTATGTCGAATTGACATGTATGAAGTGATTTTTGTTCTGATGGGGATCAATTTAATAAAAAAAGAAAAAAATTAGGGTCAGCTTGGTTCATTGAAGTGATAGTGTAAAAAGATCAATAAATCATTTGATCCTAGACTCGATAGTAAATCAACTTTATCATTCCGGAAAGAGCCACCACCGCTATGAATTTACTTTATTCTATAGTATAGTCTATAATAACTTATTATGTAATATATGGAGATAGATATATCTTATCCGCATAGTGACTCATTCAGGAATTCAGTAAAAGGGCCCCTTTAACTCAGTGGTAGAGTAACGCCATGGTAAGGCGTAAGTCATCGGTTCAAATCCGATAAGGGGCTTTTTACTTTTTTTTTCATAAAACCCGAGTCGTAGTATTTATATTTGAACATAGAATAGATTTGCTTCTTGCTATTTTTAAAGGAAAAAGTAAACAACTGTATAATAAGTTCTAGTAGTTATAAAGTTGAACATTTATTCATTATACTGAATAATGATAAATGATAAGAGAAGTCGTCTATTGAATCACCAAATATTCCTATTTTTTTTTTCAATTATTCCAACCTAATCCATTGGAAAGATTAGAAATCAACAAATGAAAAAGGAAAAGTAAGTGGACCTGACCTATTGAATCGGGACTCTATCCGCTATTCTGATAATCAAATTAGATAGAGATGAAATTGGAACGGTTGACCCCTTTTTTCTTTCATTTCTTTGGACTGCGCACGAATTTGTCGATATTTCCGAGTATATTTTTGTATTCCTAGATATTCCATAATTTTAAGAATAAATTGGCTATTCCCTTACTTCATAGAGAAGGAAGGGAAAAAGTTTCTTCTAAATCACAACATAAAAAAACCTTTTTTCGCTATCTTTCTTTGATTCCAGAGGAGGATTAATATCTATTTATAGAATAAGATTTAGATATATCTATTAAATCGTAACTTCATGTACCAACTATTTTTAGATCGATGCATCCCATATTTTTGTTTCGACAATGTGATGAAAAATACATGCGGGAAAAAACTTTCATTTCGGGTCTCCTAGTATTTTTTTTCTTATTGAATATTGTATGGAATTTCATTAAGTTAGGGAAAAAATAGTAATTCTCTATTTTTCTGACAGATAAAAGTAAAAAGGTAAATAGAAAATTTTTCATTCTTTCCTCGACCCATGAAAATGAAAAAAGAGACTCTGAAGTTTTCGATTCATCTAAAGGAAAGGGAAAGCAGGCAGAAAAGAAATATATATAAGACTGTAGTAGCATACATATAAATTCGAAAAATCTTTCAAGATATTTAATTTTTTTATTAATTATTAATCTCACGAACAAGAATAAGATAAGTTAGTTGATGGAAAGCAGGGGTCGGTCTCGGAATCAACTGGTAACGGGAACGAATATTGCTTGTTCCTTGAACAGTTCTTTCAAAAATTTTATATATTTGATTGATGAGACATAAGACAATTCATGGGTCAGATGCTTATTCAGACTAGGAAAGCATAATCGAATTGCGGTTAGGGATTTACTTTAACTTTAGGTAAAGTAATTTTATAAACCAATAAAGTTTATCTTCGAAACCCATTGTAAGAGTCAATGTACGAGAAATAAAATCATACATAAATGATCGAATCCTCGGACTCCGTACTATGAGGTGTTCGGAAATGGTTGAAGTAGTTGAATAGGAGGATCACTATGACTATAGCCCTTGGTAAATTTACCAAAGACGAAAATGATTTATTTGATATTATGGATGACTGGTTACGGAGGGACCGTTTCGTTTTTGTAGGTTGGTCCGGTCTATTGCTCTTTCCTTGTGCGTATTTCGCTTTAGGAGGTTGGTTTACAGGTACAACCTTTGTAACTTCATGGTATACTCATGGTTTGGCCAGTTCCTATTTGGAAGGCTGCAATTTCTTAACCGCAGCAGTTTCTACTCCGGCTAATAGTTTAGCACACTCTTTGTTGTTACTATGGGGCCCTGAAGCACAAGGAGATTTTACTCGTTGGTGTCAATTAGGTGGCCTGTGGACTTTTGTTGCTCTTCATGGCGCTTTCGGACTAATAGGTTTTATGTTACGTCAATTCGAACTTGCTCGATCTGTTCAATTGCGACCTTATAATGCAATCGCATTCTCTGGCCCAATTGCCGTTTTTGTTTCTGTGTTTCTAATTTATCCACTAGGTCAGTCTGGTTGGTTCTTTGCGCCTAGTTTTGGTGTAGCAGCTATATTTCGATTCATCCTCTTTTTTCAAGGGTTTCATAATTGGACGTTGAATCCATTTCATATGATGGGAGTTGCCGGTGTATTGGGTGCCGCTCTGCTATGCGCTATTCATGGTGCTACCGTAGAAAATACTTTATTTGAAGACGGTGATGGTGCAAATACATTCCGGGCTTTTAACCCAACCCAAGCTGAAGAAACTTATTCAATGGTCACTGCTAACCGCTTTTGGTCCCAAATCTTTGGGGTTGCTTTTTCCAATAAACGTTGGTTACATTTCTTTATGTTATTTGTACCAGTAACCGGTTTATGGATGAGTGCTCTTGGGGTAGTCGGTCTGGCTTTGAACCTACGCGCCTATGACTTCGTTTCCCAGGAAATCCGTGCAGCGGAAGATCCTGAATTTGAGACTTTCTACACAAAAAATATTCTCTTAAACGAGGGTATTCGCGCTTGGATGGCGGCTCAAGATCAGCCTCATGAAAACCTTATATTCCCTGAGGAGGTTCTACCACGTGGAAACGCTCTTTAATGGAACTTTAGCTTTAGCCAGTCGTGACCAAGAAACCACCGGTTTCGCTTGGTGGGCCGGGAATGCCCGACTTATCAATTTATCCGGTAAACTACTCGGAGCTCATGTAGCCCATGCCGGATTAATCGTATTCTGGGCCGGAGCAATGAACCTATTTGAAGTGGCTCATTTCGTACCAGAAAAGCCGATGTATGAACAAGGATTAATTTTACTTCCCCACCTCGCTACTCTAGGTTGGGGGGTAGGCCCTGGTGGGGAAGTTATAGACACTTTTCCATACTTTGTATCGGGAGTACTTCACTTAATTTCCTCGGCAGTATTGGGCTTTGGCGGGATTTATCATGCACTTCTGGGCCCTGAGACTCTTGAGGAATCTTTTCCATTCTTCGGTTATGTATGGAAAGATAGAAATAAAATGACCACAATTTTGGGCATTCACTTAATCTTGTTAGGTCTAGGTGCTTTTCTTCTAGTATTCAAGGCTGTTTTTTTTGGGGGCATATATGATACCTGGGCTCCAGGAGGGGGAGATGTAAGAAAAATTACCAACTTGACCCTTAGCCCAAGTATTATATTTGGTTATTTACTAAAATCGCCTTTTGGGGGAGAAGGATGGATTGTTAGTGTGGACGATTTGGAAGATATAATCGGAGGACATGTATGGTTAGGTTCCATTTGTATACTTGGCGGAATCTGGCATATATTAACCAAACCTTTTGCATGGGCTCGCCGCGCACTTGTATGGTCTGGAGAGGCTTATTTGTCTTATAGTTTAGGTGCTTTATCTGTCTTCGGTTTCATTGCTTGTTGCTTTGTCTGGTTCAATAATACCGCTTATCCTAGTGAGTTTTACGGTCCCACTGGACCAGAAGCTTCTCAAGCTCAAGCATTTACTTTTCTAGTTCGAGACCAACGTCTTGGGGCTAACGTGGGATCCGCTCAAGGACCTACTGGTTTAGGTAAGTATCTAATGCGTTCCCCAACTGGAGAAGTAATTTTTGGAGGAGAAACTATGCGTTTTTGGGATTTGCGTGCTCCTTGGTTAGAACCTCTAAGGGGTCCAAATGGTTTGGACTTGAGTCGGCTGAAAAAAGACATACAACCTTGGCAAGAACGGCGTTCCGCGGAATATATGACTCATGCTCCTTTAGGGTCCTTAAATTCTGTGGGTGGCGTAGCTACCGAGATCAATGCAGTCAATTATGTCTCTCCTAGAAGTTGGTTAGCTACTTCTCATTTTGTTCTAGGCTTCTTCCTATTCGTAGGTCATTTGTGGCACGCGGGAAGGGCTCGTGCAGCTGCAGCAGGATTTGAAAAAGGAATTGATCGTGATTTTGAACCTGTTCTTTCGATGACCCCTCTTAACTGAGACAAGAGATCCAATGCTTAAAGTAGGAATCATTTTGATTCCACCATACATATTGGGATCGGGTCATACTTAAAGATTAGTCCCTTTTTTCTTTATTTTATTTATTTTGTTTTTCAACTCATATAAAAATTCACTATCTATATATAAATAAAATTGTCGTTCTTTTCTGGCTCGGCTAGCTCACCCTAGCCGAGCCATTCCCTTTATTTTAGTTTACCGGGACGGGCAAAACCACTAAAATAAGGAAATCAATCTATTCAACGAGCAAAAGGAGAGAGAGGGATTCGAACCCTCGATAGTTTTTAGAACTATACCGGTTTTCAAGACCGGAGCTATCAACCACTCAGCCATCTCTCCAAAAGACAATTTCTATTTTATTTTGATTCCCCCGAATAGAACATGGTCATACGGGTTGATACTATTACTATCTATAGATAAATATCAGGTGTGAAATCGATAGGTCGATCTATTTATCTGTAGATATCTCTATATATAGAGGTATGATCTAGCCTGCCCATTTAGTGAAATTCCCCGCGACTCGAATAAAGTGGTAAAAGGTGGTAATAAGTCATAGTATATAGAATCAATATATTCATATAAAATGAAAAAAAATCCCTATATCTATATAATAATGCATTTTTTTACTGATAGAGGGATCAAATGGTCTAGTTCATTTGTTGGTAGCGTGGAGGATTATAAGTATGACTATAGCTTTCCAATTGGCTGTTTTTGCATTAATTGCTACTTCATCAATCTTACTGATTGGCGTACCCGTCGTATTTGCGTCTCCTGATGGTTGGTCAAGTAACAAAAATTTTGTATTTTCCGGTACATCATTATGGATTGGATTAGTCTTTCTGGTAGGTATTCTTAATTCTCTCATCTCTTGAACCTATTCGTCCCAGATCCAAAAATGACCCCTCCCCCGAATTCGAAATTTTTCGGGTTGCGAGACAGAGTAAAATTCAATCAATATAAGTCCCCAAAATGCAAACAAATACAACAAATACAAATAAAAATAAAGAAAACACAAAAAGGGGAGGGGTCAAACTTAAACTTCTTGAATGAATTCAATGAAATAAAAAAGATTGAAAATTTCATTGGACTTGATTGAAGAAAGTATCTGGCCCAGCTCTGCACAAATATGGTCAAGACATATATACTATATATGCGGACATATTCCTTCTCAAGAAGGACAAAATGCGGATATAGTCGAATGGTAAAATTTCTCTTTGCCAAGGAGAAGACGCGGGTTCGATTCCCGCTATCCGCTCAAGGTTCAAGATGAAGTTATTAATATGATTAAGGGATTGGGTATAGTTGACCGGGATAGTGTAGTGATTCTATCTTCCTCCTTCGTTTTTTTCTACCACCCACCCCAAAACAAACAAAAGAAAAATAAAGTAATAAATTAAGAATTTTTTTTTACAAAAAAATCGTGCGGAGACAGGATTTGAACCCGTGACCTCAAGGTTATGAGCCTTGCGAGCTACCAAGCTGCTCTACTCCGCGCTGAAGAGAAGAATTGGGAACTAATGTAATGGACAAACAAGAATGTACCCCCTACCATCTCTGTACAAATAGTATAACCCATTTATACAGAATGGTCAAGGGGCACTCTATGATTTATGATCATAGAAATGAAAGGATATTTGAATCCTTACCAACTTGATCTTGTTGCCCCTGGCAACAAGCATGTATGAACCATTTCACGAAGTATGTGTCCGGATAATCCAAAGTCTCGATAGTTAGCTCTCGGTCTTCCGGTCAAAAAACAACGTCGATGAAGACGTGTAGGTGCACTATTACGCGGTGGAGATTGTAACTTTCCATGAATTTCCCATTTCTCATTCAAGGACGTAACTTTGCTTATTTCTTTCTTTAAGGATCGACGAATCAAATGATATTTCTGTTCCAATTTTTGCCTCTTCTTCTCCCTCTGAATCAAACTTTTACTTGCCATAAAGTTTCAGTTCCTATTAGTATCAATGATACAAGTCGGATCCTAGATGTAAAAATAGAAAAAAGGGGACACCCTTCTACATCGAAAGAAATGAGATTATCGCGGATACAACACATTCCAAAAAATTAACCAAATTTTCCTGATGTAGAGGCAATCAAGAAAGCTGCATAAGTGAATATATAACCTACAGAGAAGTGGGCTAATCCAACCAATCTTGCTTGCACAATGGAAAGAGCTACAGGTTTATCTCTCCATCGAATCAAATTGGCCAAAGGTGTGCGTTCATGAGCCCATGCTAAAGTTTCGATCAATTCCTGCCAATACCCACGCCAGGAAATTAAGAACATAAATCCCGTAGCCCAAACAAGATGTCCAAATAAGAACATCCACGCCCAAACCGATAAACTATTCATACCAAAAGGGTTATATCCATTGATAAGTTGTGAAGAGTTTAACCATAGATAATCTCTTAACCATCCCATCAAATAGGTGGAAGATTCATTAAATTGTGAAATATTACCCTGCCATAATGTGATGTGTTTCCAATGCCAATAAAAGGTAACCCATCCAATGGTATTTAACATCCAGAAAACTGCCAAATAAAATGCGTCCCAAGCCGAAATATCACAAGTACCGCCTCGTCCCGGACCATCGCAAGGAAAACTATAACCGAAATCCTTTTTATCTGGCATTAACTTGGAACCACGTGCATCTAAAGCACCTTTTACTAAGATCAATGTAGTTGTATGTAAACCTAGAGCAATAGCATGATGAACCAAAAAGTCTCCAGGACCTATTGTTAAGAATAGTGAATTACTATTCTCATTAACAGCATTTAACCAGCCGGGCAACCAGATACTTCGACCCGCATTAAATGCCGGGCCGCTTGTTGAAGATAAAAGTACATCGAAGCCATATGAAGTTTTACCATGAGCAGATTGTATCCATTGAGCAAATATGGGTTCGATCAAGATTTGTTTTTCCGGAGTACCAAAAGCAAGCATGACATCATTATGAACATAAAGACCCAAAGTATGGAACCCTAAAAAGAGGCTGGCCCAGCTTAAATGGGATATGATAGCTTCCTTATGGTCTAACATTCTTGCCAATACATTATCCTCATTCTGCTCCGGATTGTAATCTCGAATGAAAAAGATAGCTCCATGCGCAAAAGCTCCTGTCATGATGAATCCTGCGATGTATTGGTGATGTGTATATAATGCAGCTTGAGTAGTAAAGTCTTGGGCTATGAACGCATAAGCAGGTAAAGAGTACATGTGTTGAGCGACCAAGGAGGTAATAACCCCTAAAGAAGCTAGAGCAAGCCCTAATTGAAAATGAATCGAATTATTGATTGTGTCATAAAGACCCTTATGTCCACGTCCCAATCGACCTCCCGGAGGAATATGTGCTTCTAAAAGATCTTTCATACTGTGCCCAATCCCGAAGTTAGTTCTATACATATGACCAGCAACGAGAAAAATAAATGCAATAGCTAAATGATGATGAGCAATATCGGTTAGCCATAAACTTTGCGTTTGCGGATGGAATCCCCCAAGAAGTGTTAGAATGGCAGTTCCCGCTCCTTGGGAGGTACCAAATAAATGACTACTTGAATCGGGGTTTTGAGCATAAAGATTCCACTGACCTGTAAAAAGTGGGCCTAACCCTTGAGGATGCGGTAATACGTCTAAGAAATTATTCCATCGAACGTACTCCCCCCTGGATCCGGGAATAGCGACATGAACTAAATGTCCTGTCCAAGCCAAGGAACTTACTCCGAAGAGCCCTGACAAATGATGATTGAGACGTGATTCTGCATTTTTGAACCACGAAACGCTCGGTTTCCATTTCGGTTGTAGGTGTAACCAACCCGCTATTAAAGATATGACAGAAAGAAATAATAGAAAAAGAGATCCAGTATAAAGATCCTCATTAGTGCGTAAACCGATTGTATACCACCACTGATAAACACCAGAATAAGCGATATTCACTGGGCCAGGAGCACCACCTCGAGTAAAAGCTTCCACAGCCGGTTGACCAAAATGAGGATCCCAAATTGCATGGGCAATAGGTCTTACATGTAAAGGGTCCTGTACCCATGACTCAAAATTTCCTTGCCAAGCTACATGAAACAGATTTCCGGAAGTCCACAGAAAAATTATTGCTAATTGCCCAAAATGAGAAGCAAAAATATTCTGATAAAGACGTTCTTCGGTAATATCATCATGACTCTCGAAATCATGTGCGGTAGCAATGCCAAACCAAATACGACGAGTAGTGGGGTCCTGAGCTAAGCCTTGGCTAAACCTTGGAAATCTTAATGCCATAATGCCTTTCAAATCCTCCTAGCCATTATCCTACTGCAATAATTCTTGCTAAGAAGAATGCCCATGTTGTGGCAATTCCACCCAGAAGGTAATGAGTTACTCCTACAGCACGTCCTTGTACAATGCTCAAGGCTCTAGGCTGAGTAGCAGGAGCAACTTTTAATTTATTATGAGCCCAAACGATGGATTCAATAAGTTCTTGCCAATAACCACGCCCGCTGAATAGAAACATTAAACTAAAAGCCCATACAAAATGAGCACCTAGGAAAAAAAGGCCATATGCAGATAATGAAGAACCATAAGACTGAATTACCTGAGATGCTTGTGCCCATAAGAAATCACGGAGCCAGCCATTAATAGTAATGGAACTCTGCGCAAAGTTTCCTCCCGTGATATGAGTTACCACCCCTTGATCACTTATACTACCCCAAACATCGGACTGCATTTTCCAACTGAAATGGAATATGACTACCGAAATTGCATTGTACATCCAAAATAGCCCCAAGAATACATGATCCCAAGCAGATACTTGGCATGTCCCCCCTCTTCCAGGCCCATCACAAGGAAAACGAAAACCGAGATTTGCCTTATCCGGTATCAAACGCGAGCTACGAGCAAATAGAACACCTTTCAGAAGTATCAGTACCGTCACATGAATTGTAAATGCATGAATATGGTGGACCAAAAAATCTGCGGTTCCTAATGGAATAGGTAACAAAGCGACCTTGCCACCCACTGCTACTAAATCACCACCCCCCCAAGTCAAGCTGGTGCTTGTTGTTGCACCAGGAGCCGTTGCACTAGGCGCTAAAGCATGGGTGTTTTGTATCCATTGAGCAAAGATGGGTTGTAATTGTATAGCGGTATCTGAAAACATATCTTGAGGACGTCCTAAAGCGCTCATGGTATCATTATGAATATACAAACCAAAACTGTGAAAGCCTAGAAATATACATGCCCAGTTGAGATGTGATATGATTGCATCGCGATGTCTAAGGACACGATCTAATAGATCATTGTATCGAGTAGTTGGATCATAGTCTCTTACCATAAAAATGGCTGCATGCGCAGCAGCACCAACGATGAGAAATCCACCAATCCACATGTGATGTGTAAACAATGACAGTTGAGTACCATAGTCAGTAGCTAGATATGGATAAGGGGGCATGGAATACATATGGTGAGCTACAACAATAGTTAAAGACCCTAACATAGCTAGGTTAAGAGCTAATTGAGCATGCCATGACGTTGTTAGGATCTCATATAGGCCCTTATGTCCCTGGCCTGTAAATGGACCTTTATGAGCCTCTAAAATATCTTTTAGGCCATGACCAATACCCCAGTTGGTCCTATACATGTGACCCGCTATCAGGAAAAGAATTGCAATAGCTAAATGATGGTGTGCAATATCGCTCAGCCATAGACCCCCAGTTACTGGGTCTAATCCTCCACGAAAAGTAAGAAATTCTGCATATTTTGACCAATTCAGGGTGAAAAACGGGGTTGCTCCCTCGGCAAAACTGGGATAAAGTTGAGCCAAAAGATCCCGATTCAAGATAAATTCATGAGGAAGTGGTATCTCTTTAGGATCGACTCCAGCATTTAGAAATTGGTTAATCGGCAAAGATACATGTACTTGATGCCCCGCCCAAGAAAGAGACCCAAGTCCTAGTAGCCCCGCTAAATGGTGATTCAACATAGATTCCACATCTTGGAACCAAGCCAGTTTTGGAGCTGCTTTGTGATAATGGAACCAACCAGCAAAAAGCATCAACGCCGCAAAGACCAATGCACCAATTGCGGTACAATAGAGTTGTAATTCACTAGTTATTCCAGAAGCTCGCCAAATATGAAAAAACCCAGAGGTTATTTGTATTCCTCGGAAACCTCCGCCTACATCACCATTCAATATTTCTTGGCCCACTATTGGCCAAACCACTTGGGCACTAGGTCCAATGTGAGTAGGATCGCTTAGCCATGCTTCATAATTGGAAAAACGAGCACCGTGGAAATACATGCCACTCAGCCAAAGAAAGATGATGGAGAGTTGACCGAAATGGGCACTAAATATTTTTCGAGAGATCTCCTCCAAATCACTGGTATGGCTATCGAAATCGTGAGCATCAGCATGTAGATTCCAGATCCAAGTGGTAGTTTCAGGGCCCTTAGCTATTGTCCTTGAAAAATGACCTGGTCTGGCCCATTCCTCAAATGAGGTTTTTATGGGGTCCCTATCTACCAAAATTTTGACTTCTGGTTCCGGCGAACGAATAATCATTGAGTCCTCCTCTTTCCGGACACGACATACAAAGAGACCTGCCAAGCGTCAAGTAATTAATGAACCTCTGAGAGATATTTCAATTTTGTTTCTTTATCTTCTATTTCCCATCTCTCTAGTTTCTTTAGTTATTCACTAGAACAAGTATGATCGGGAAGTCGATCTAAGGCAAGTGTTCGGATCTATTATGACATAGCCGTGAGGCGCTCAACGGACCTTTTTTCTATTATAAACCCTTTCTGGGCTTTGGATTGATGTAAAAAACGATTTTTTGTTATTCCTATCTAAACTAAATTATAAGGTCTTAGACGGAAGGACTTAATGTTTTACAGAGATTCTAGTAATTCGTATTACTCTATTCCAAATCACGCGAGCAGTCATTAGTCATTACTAATGAAACATCCCAGTATCTATATTTCATTTTTTTATTTTAAAATAACTAAAAATTCAAGTTAAAGTATTATTAAATTAGTATTAATAGCTAAAAAATTAATAGCTAAAACTAAAAAAGGGGTATTAATTGCTAATAAAAGAAATATATTCTGTACTCTATCTGTTTATTCTTTAACCATACGAAATACCCAACAAAATAGAACGATCTGAAAAGGGATATAATGAAATTCTTTGATTGGTTCTTCCCGTAGGAATGATCCTATTTTATTTGACTCATAGGTACAACATTAATTCTAACAAATAGAATATCAAAAATTCGAAATTTTTTATTCGAAACGCCTCGTGATCTTCAACCAATTATGTGCTTCAATATAATTACCAGGAGTAAGCGCTATAGCTTGTTTCCAATACTCAGCGGCTTGATCGAACCAAGCCTCCGCAATTTCAGAATCTCCCTGTCGAATGGCCTGCTCTCCCCGGTCGGAATAGAGTGTTCCTTCCCTTAGAACCGTACTTGAGAGTTTCCTAACTCATACGGCTCAGCAGTCAATTCTTTTGGTATCCGTTTTACCTATCGAAGGGAATGAGATTTCTCGTAGATCTATCTCGCTTTTCGGTTTCGGGCTAACCAAAAGAGGTTAATCGCATGAGTTTCAAACTTTCATTTTGATTTCTAATTAGTTTTTGTTTTATCTTTTATCCCACCTTCAGACGAATAAAGGATGGGCATTTCCTCCTTTCGTTAACATTTTCTGCAAGGTAACTATCTCGGTTTCATATCACAATTTATATAGAATCCTTGAAAAAGGCTTTCTTTCCTGCATAAGAAAGAAAAGCTTACTATCTTTGGGATCTGATCCTACACCGCTGCTCAATACCTTAGTGGATCGCCTCTATTACATAAGCAGATTACTAACTTTTATCTATCTTAGATTATGGCATAAGTAAGCAGTTCTTAATGTATTGGCCCAAACCTCGTTAATTGATCTTTACGGTGCTTCTTCTCTATCAATTCTATTTTTTTTATCCATAGAATAAAGTATCTAGGCATATCTTATTTCTTCATATTTTCGACTCATATGAAGTTTCGTTCCGTGCTACAGCTCATAAAAATCGTTGTTTTTGACGATGCATATGTAGAGAGCCTATTTTCTTTTTTTTCGTATTTACTAGAACATTTTTTGTCTTTCTTTCCTTTTATCTTTCTATAGTGGAGATAGTCGCACGTAATGACAGATCACGGCCATATTATTAAACGCTTGTGGTAAGAATGGGTTTCGTTCTAGTGCCCTAAAATAATATTCTAAAGCTTTCGTATGATCCCCATTACTTGTGTGAATAAGGCCTATGTTATAGAGTATATAACTTCGGTCGTAGGGATCAATTTCTAGTCGCATAGCTTCATAATAATTCTGTAAAGCTTCCGCATAATTTCCTTCGGATTGGGCTGACATCCGTTACGGTCGTCATTCGATTCAAAGAATCTCCGTTCCAGAACCGTACGTGAGATTTTCATCTCATACGGCTCCTCCCTTCTATGCATAATGAGAATATTTCAATCGTTTTTGATTCCATGTATCGATTATTCTCATTATGAATTGAGCGGGGCTAGTGTTTTTGCACGAAATGTCTAGCCAACCTTACTGCGCGAGAGCTTTTGTTAACATCAAACGTGTTGGTACTAGATAGAAATGGTAACTCCAACAATTTCTTTGTCCTCAACGCCCCCTAATTTCCAGGAATTAGTCACTTCAACAGTCTTTGATGGTTATATGGGTATCCAAGGTACGAACGAGATGGATGTTTGTTGTCCCAACCATTCTTTTAAGTCCCAATCCAGATAAGGAAGGGGGTAAGTAATTTTTAACAAAGTTTTCGTCTTGTTGATTTCTAGGTGTAGTGCTTTTCCCCTATGCTGCCTATTAGGACTAGTAGAGTGGGATTGACCTGTAATACAGAACCGATAGGTGTAACCTTTCGCTCAATACTAAAATGGATAATGGAAGCATATGAGGCTGCATTAATCGGGGATACACGACAGAAGGAATTGCTCTATTTCTAAACTTCACCTTCAACGAGCGTAGATTTCTTTCAATAATCTATCAAAATAATAATATTTTCTCTTTCTATCCCGAATTTTTTGTCTTTCTCATAAGACTGGGGTAAAAAAAAAAGAATCAAATCACACCATCTCTGTAATAGGTAAATGCCTCCCTTTCGCCTGAAGTTGTTGGAATTATTCGTAATAAAATATTGGCCACAACTGAAAAGGTCTTATCAATAAAATTTCCATTTATCCGTGATCTAGACATAGGTACCAATCCATTCTAAAATTCTTTTCATTCCCCCTCGTGGGAAAATGATTCTACAAACAAAGGAATTGTACAATACGAAATAGCATAAAAAAGATTCATAAAAAAAAAAGAAATTCAAATATCAATATTAAATAAAATGTAAAGATAGGAACCCTCTACTACTCATATTCAAAGACTCAATTTGCTCCTTTTTGCAGGAATCAATAATAAATATTTGAATACGATTCGAATTCATCCAATCCAAATGTAGTATACCAATGGGCGAACTAGTCCTATTTCATCGAAGCTGAAGAATCAAGGAATTTTGCTTATAGATTCGGGCGAAAGACTTTGATTGAATTATCATCCAAAGAGGGAGGGAAAAAGAATAGAATAATTATTCTATGATGTAAATAGAATAAACGTCTATTTTGTTTGTGTTATGTGCATAGTGAGTAGACACTATACAATCAAATAGCCCCAGGATGAGTCATGAATTTGTAAGAGATCTATGAAATAATCGATTTTTTTGGTGAAAACTTTAAAACTATAGAAAGGAATTTTCTAATTTTTATGGAATTGTCGTTTAAATGTAATCATGATCGAAAGGTATCCATTAATCATAGTCTAAAAAACCTAAACCCATCAATCCGTTGATTCCTTCCAATTCATTGATTTAATCCCGTATAAATATCATATCAGAAAGGGGCGGGAACATCATCTTCGCAAATATGAAAAATATATCTTTTTACTTTAACCTTTCACAAGCACAAGAAAAAACTTTTTTATTTTAATCTCCGAGCCTTGAATTGAAGTAAAGATCTTTATCAAAAATTTGATATTTTTTTAGTTTTAGTTAGAATTGGTTGGTTGTACCTTACCTAGCCAATCCAAACAAAAAGAAAATATGAAGAACTCGCTATTCATTCGGTTCCTGGGTCATAATCGTTGTGTAGGAGAGGTGGCCGAGTGGTTCAAGGCGTAGCATTGGAACTGCTATGTAGACTTTTGTTTACCGAGGGTTCGAATCCCTCTCTTTCCGTACCTTCACCCAACTCACCAACATCGCTGACCGTAACAAATCAACCAAGAGGTAGATCCTTCTTTCTATCTTTATATATATCTTATTTCTATTTATATATAGATATATAGATTTTCTATTTCTAATTAAATTGCTGCGATATGTAAAATAATGGAATAAATGGAATAAGGTCGACAAGGAATAAAAATATCTCTGTCGATCTATGATACATGAATGGGAAAAACCCGGATCAAACCCCTTACTTTTAATTTTAGTCAATTTACTTTGGCGAAAGGGGGCTTATTTTTCTGAAACCTTTTCTAAACCTTTTTCTTTAAGTGTAGGCTTAAGTCTGACGGGAATAATATTCTACGACTAGTAATTCATTTATTTTCAAACCGACCCACTTATTATCTATTATTTGATTGACTACTCCTTTATATGGGAATGGGTGAAGAGTCAAATGTTTTGGCAATTCCTCACTGTGGGATGAATCCAGATAATTTTGAACGAGAGCTTTGGATTTTTGCTTATCCCTCGCCATAATAATATCTTTGGGTTTGCAACGATAACTTGGTATATCTACTATACGACCATTAACTAAAATATGTCTATGGTTAACTAATTGGCGGGCTCCAGGAATAGTCGGAGCCATACCCAATCGAAAAAGGATGTTATCCAAACGCATTTCAAGTAATTGGAGTAAAACCTGACCTGTTGACCCTTTGGCTTTTCTAGCGATACGAAAGTATTTAAGTAATTGTCGTTCTGTAATACCATAATGAAAACGTAATTTTTGTTTTTCTTCTAGACGAATACGATATTGAGATCTTTTCCCGGAACGTGATTGGTTTCTAAGCTCTCTAGGCTTTTTATTAGTTAGTCCTGGTAAAACCCCCAGACGTCGTATTTTTTTGAAACGAGGCCCTCGGTAACGCGACATAAAGACTCCTTATTTATTGTTATTGATATTTCTTTTTATTTAAATAAATTAAAACTGAACTAAATGATAAATGAAGCGAAATCCCCTGAAGTATTCTATTCATTGTACTAGAACGAAGAATGGCACTAGAAGGAATAGTGAGATGAAAGATGTACGTATCCGAAGTTCCTCCTTTTTTTGTATTAATATGAATTATTCATTATTATTTTATTTGAAATGAAATTTCATTTATTAATTTTAATATTAAAAAAAAAATACTTTATGTATTTAATGTATTTAAATTTTTATAATTATTGGAATTGTATTTAGTATATATATATTTTAATTAATTGATTAATTGAATTAGAATTTTTGTATAAATATATTGTATATATTTGATTCTTAGTTTAGTTAATATTTCATTTATTTCATTTTCATTTTGAATTTTTGTTTTGTTGTATAGTATAATTAGATTTATATATAATCTAAATAAATAAAAATATAATGATATAAGTATAAAAAGAATCTAAAATAAAGAATATAAAATAGAATAATATACAAAATATACCAACATATAAAAAAACATAGAAAATAAAAATAAGAAAAAAGATCCTTTCCAGAGTTGAGTTGTTCTGCCGAGATTTGACTTTTTCATTGACCTTTTATTCGTAGTTGGAAGTTTCTATGACATAATAAATCGTCGACCTTTTTTTTGAAAAGGAAAGTTGTCTTTATTCTTGGATTTCAAAGAAGCATTCTCAATCATAAAAAAAAATAGAACAAATAGAGAAAAGCCGGCTATCGGAGTCGAACCGATGACCATCGCATTACAAATGCGATGCTCTAACCTCTGAGCTAAGCGGGCTCACATAAGATAAACTTTACATGCATAATAATTCCATAAACAATATCTTAGCTATTAACTATTCATAAATCATAAAAAATATAGAATATAAATTGATTTCAAATCCATATTGCATTATTTATAGTATATAAATAAGATAAAGATTACTATACCGATCTATAATTTAGATTTATTACATTTTACATTACAATTCTAATTATAACAATTATAATAATACATTTATCCTTTTTTATCAATCAAAAAAATTGTCATTTTTAATTAGATTATAATTTTAGTTTTCGAATTCGATTTAGATATTCTATTTTTAGATTTAGTATTCGTTTCTCAGTTTTTATAATCTTTTTAATATATATATTTCTTTATAAATCTTTATTAAAATTTGAATATATTCTTATAATTTATTCTATTCAATTCAAATTCAATTATGAGCTCTATTCAATTATGAGCTCTAACTATAACAATATATTAAATAAAAAAATGAAAATATCTTATTCTTTTTAATTTAATATTAAGTTCTTATTCTTATTATCTTATTCTTATTAATATTAAGTTAAGATTAAGGTAAGGAATTTTTATTTTATTTCTCTTTTTTCATATAATAGTTATTATATAGAGGTCTACCCTAAGAAAAGAATAAAAAAAAGAATAAAAAAAAATGGAATATGCCTATAAGATATGGAAAAAGAGAAATAAAATATATAAAAGATAAAGATGCAATTCAGATCAGAATAAGACATTCCCATGTTTTGATTTGGAGACTAAGACAAAAAAAAGAATCGACCCTTTGAGTATTCCAACTTTCATGGGAAAATGAAAAGAAAGGTTCATATAGATAGTGATAGATATCCGTCTATATTGAATTGAAGATAAAAAAACGATAGAATTATTTCTGATTGGCCCATATCCAATACGGGCTCCCACTAGAGATGAAAGAAAATAGGCAAAATCAAATAGGATGAAATGCCTTTCGATATATGACGATATATGAATTTATATATAATAATGAATTCAAAGGTTCCGGCGTAAATGAAAGGATAAAAAAGGGAAAGTACATCACACTGAGATCTTAAGCATAAAAAGGGGGATATGGCGAAATTGGTAGACGCTACGGACTTAATTGGTTTGAGCCTTAGTATGGAAACCTACTAAGTGAGAACTTTCAAATTCAGAGAAACCCTGGAATTAATAAAAATGGGCAATCCTGAGCCAACTCCTGCTTTACAAAAGGGAGAATAAATAAAGGATAGGTGCAGAGACTCAATGGAAGCTGTTCTAACAAATGGAGTTGACTGTCTTGCGTTGTTATAAGAATTCTTCCACCGAAACTCCAAAAAGGATGAAGAATAAACCTATATGCATACGTACTTTAATTTAAATACTATAAATATAAAATAAAAAGAAAAAAATAATAATTAATGACGACCCGAATCTGTATTTTATGAATATGAAAAAATGGAAGAATTGTCGTGAATCGATTCCAAGTTGAAGAAAGAATCGAATATTCGTTTATTAAATTAAAGCATTTACTCCACAGTCTGATAGATCTTTTGAAGAACCGATCAATCGGATGAGAATGAAGATAGAGTCCCATTCTACGTGTCAATACCGACAACAATGAAATTTATAGTAAGAGGAAAATCCGTCGACTTTATAAATCGTGAGGGTTCAAGTCCCTCTATCCCCAAAAAAGCTCATTTGATTCCCTAACTAGTTATCCTCTTTTTTTGTTAACGGTTCAAATCAAAATTGGGTCTTTTCCTCATTTACTCTTCTTTCACAAAGGTATCCGAGCGGAAAAATTTTTCTCTTATCAGAAGTGAAATGATACATGAACAGCTTTGACCAAGGAGTACTCTACTCATTTGAATGATTCCCAATACATATCATTACTCGTACTAAGACTTACATACAAAGTCTTCTTTTTAAGATCCACGAAATTCCGGGGCCTAGATAAGACTTTGTAATACCCTTTCGCCTTTTTAATTGACATAGACCCCAGTTTTCTAGTAAAATGAGTAGATGATGCATAGGGAATGGTCGGGATAGCTCAGTTGGTAGAGCAGAGGACTGAAAATCCTCGTGTCACCAGTTCAAATCTGGTTCCTGGCACATGATTCATTTGGATGAGTATCTATTTTACAAATTAATTGATATGGATCGATATTCATTAATCGTATAGATTATGCACATACGTAACTTCTCCAGGTGTCTAGAGATATACCCCACCTATAAAATAGATGGGTAAAGAGTATATAAAAAGATGTAAAAGAGTTATGTTTTATTTTCTTTCGTTTTTTATTTGTTGTTTATACTGTGTCTCCTCTCATTGAAAAAGAATATTACTCCTTCATACGAATTCGAAAAGGTTTAATTTAATTAGTTAAGTTAGTTGCAAGACGAAAAAGTCTAGGGGAGTTAAAGTAAAGGATGAGAATAGACAAGATGTATCTCAGATACAGTACAAATAGAATCCGACCTCCTCTCATTTCTTTTTTTCTATTTCTTCATTTCCCCCCTACATTACGTGACTTTCTACAGACCATCTAAGTGATGTTCGATGTTCGCGGTACAAAGTTCATGATACAAAATTTTTTTGGTTCATTCTATTGGCTCGGCTCATCCAAAATAAAAGTATCTTCCCAACTTTCGAATCTTAATGAATTCCTAATTCGATTTTCTTTCTTTCCAACCATAATCCGAATAAAATGTCAATTACTCCGATTGTTTGATCTAGAACAGAGTGTACAAATATTCCTTAATATATATATTTGTAACAAATATTCCTTAATATATATATTTGTATTTGATTTGAAATTATTGAAGTGAAGATGAGTTTCTTATCATTCAATAAGCATCTTGTATTTCATAAAAATTGGGGGCAATATAATCTTTACGTAAAGGCCATCCTATCCAACTTTCGGGCATTAAAATACGTTTCAGGCGTGGATGATTATCATAAAAGATTCCCAACATATCATAGGATTCCCGTTCTTGAAAATCCACACTTTTCCAAACCCAGAAAACAGACGGAATTCGAGGGTTCTTCCTTGGGGCAAATACTTTTATGCATACCTCTTCTGGTTGATCCACACCCGATTCTATTCTCGTAAGATGATACACACTAGCTAACAGCCCGCCGGGTTCGACATCATAGGCACATTGGGAACGTAGATAATTGTAACCATATACATATAAAATGACAGCAATGGAATGCCAATCTTCGGGCTTTATTTGTAAAGTCTCTATTCCTTGGTAATCGAAGCCCAAAGATCTATGAACTAGTCCATGTTTGACTAGCCAAGCAGACAAACGACCCTGCATCTTGTTTATTTCTCCCACATTTTGATTTGTATAAATATTGATTTGTATTAGTATTTCCTGAAATTTGTAAACGCGGGACCCCCATTATTACAAAAAAAAGAATCCTGCTTAATTCACAAATTCGTGGGAAGATACTGAACTTTTGTATTTGAAAAATGTTTCAGGAGGGATCTCGGAAATAGATGGAGATTTAGAAAGTAATCCTTGATTATAATTTCCAGTATGAATACGGCGTCCAACACGAAACTTGTGTTTGATAGTAAAACATCGATTCTTCCGTTGAGACTGAATTCTATCTTCATAGATTTCCCAAGATATTTTCTTACGAAGCTTTGTTATAGCATCTATAACTGCTTCCGGTTTAGGTGGACAGCCTGGCAAATAGACATCCACAGGAATTAGTTTATCGACCCCCCGAACAGTACTATAAGAATCGGTACTGAACATCCCCCCTGTAATTGTACAGGCTCCCATAGCAATAACATATTTTGGTTCAGGCATTTGCTCATATAATCTCACTAAAGAAGGGGCCATTTTCATTGTTACTGTACCAGCTGTTAAAATTAGGTCTGCTTGTCTAGGGCTCGATCTTGGTACCAGTCCATAACGATCAAAGTCGAATCGCGAGCCTATTAATGAAGCAAATTCAATGAAGCAACAACTGGTACCATAGAGAAGCGGCCATAAACTAGAGAGTCTTGACCAATTTGAAAGATCATTTGATGTAGTTGAAATAACTGAGTTAGGGGTTATTCGATCAAGTAGGGGAAACTCAACGGAATTCATAACTCTTTCAATCTTATTGTTTTTTCTTTTTTTATTTTGATTGTTAGAATATTCAGAAACTAAGACCATTCCAATGCTCCTTTTCGCCATGCATAAACTAAACCAACAATTAGGATAAGCACGAAAATAAAAGCTTCTATAAATACAGATACACCCAATACATCGAAACTCATTGCCCATGGATAAAGAAAAACCGTTTCAACATCAAAAACAACAAAAACTAAAGCAAACATATAATAACGGATTCGAAATTGTAACCAAGCATCACCAATGGGTTCTATACCTGATTCATAACTAGAAAGTTTCTCTGGTTCTTTTCTAATCGGAGCTAAAATTCCGGAAAGTAGAAATGCCAAAATCGGAATAACACTTGATATTATTAGAAATGCCCAGAAAATATCATATTCATAAAGCAGAAACATAGACGCACTCCTATGAATGTGGAAAATATAACGAATTAGGCGATTCGACTTGGAATTGTCAAGTCATCCATAACTGTTTAGTCAAAACAACAATTCATTTTGATCGAACCACCTAGTTTCGTTTGTTTACCGCGGGACATGTATCCTTTCAAGATTCATTGACTGGAATCTTATTTCCGTTTTTCATTACACCTAACTTACTTATCTTATTAACTTACTTATCTTATATATATTTCATATCATATATATATTTCAAATTATATATATTTCAAATAAAATAAAAATATAAGTAAAATAATAATAGATATTGCTCTTATCTTATATTATACATATACAAAATACATATATACAAAATACATATATACAAAATACATATACAAAAAAAACGTTCTCGCTTTCACCTCATTTCCTATTTTCTCTAAAGAAAAGGAATGGAAAATCGAATTCTCATTTCACATTTCTTTCTAATTTCTCATCTTTTTTTAATAAAAAAAATGAGAAATTGAAAATCTTAATTCTTAAAATTCTTAAAACTTAAAATAAGAAAGATAATAATATCTTCAAAAAATATTAATAATAAAATAAATTCTATTTCTTTATTATATTTTTTATTTAGATTTTTTTATGAATAGATTATCTTATGAATAAATAGAATCAAGAAAACTGTCTTATTTTTTCCTCTTTTTTCGTAGTGATTTAGAATAAAGTAGTCAGACCTAAAAAAATTTCTATTTCAGGATTTCATTTGAAATTCAAAAATGAATAATTATTAATTTAATAATTTTCATTATTATTAATAATGAAAAGGTCTTGGTATATTTTATTAGTTATTAGAATCCGGGCGAAGTAATTTATATTGATTGGATATGGAAAGAAAAAACGACTTGTTTTGTTCTTTGCTAGGTAAGGTATACGACGAGAAAAAGTCTATTTCACAACGTTGACAACGTTTCCAATGAAACTTACTAAAGATCTTTTTCAAGCCCTCGCTTGTCCACAAATCAAATAAAACAAATCAAATTCAGTAGTATGTTCCGAGATCAATATGATTTACTATTCGATTGGGGTGAAGTTGAATTAGGTTGCTCGCTCAGATCATGATTTTGACTCCAAAAATTCACTAGGATTACATATAAAATAAAACTAGGATTATCATAATAGATTGGGGAGAGTAAAGAGAAAAATTCACATGACATTCGCCTACGAAGATTAATGGAGAAAATGGGTTTGTTTAGCCGAAATTCTAACAATGCTGATTGGATCCGTCGAAATGCACTTTTGTTTTCCGTTTTATGCTTCGCTCTGAATGATCCTGGGGAGCAAGCTTCTGGGAAGAATTTTTTTTTCGATGAACCGTCCGGCCATTTAAAAATAAAATAACAAACACTACTACTGAGGAAATGCAAATTTTCATATAGAAAATTATAGGGCTATACGGACTCGAACCGTAGACCTTCTCGGTAAAACAGCTCAAACTTGATTATTATTATCAAACTGATTCGAACTGTTTCAAAGACCCAACATGCATTTTTTTTGCATTGGGCTCTTTCATTAACTGATATAAATATAAGTTAGTCCACCATTTTTTTTCTTGACAGAAAGCAGATGGTTCCAGGTGCTCGGATTCATTATTTGGACTCTGATCCAGGAGCACTACCAAAGTGTTTCAAAGAAGGGTTATCTTGACGTAGGTCTGCCTTTGGCCTAGATCTATTTTAAAATGAAATGGAGTCTCTATCGCTCTGCTTAAAGAATCAAATATGAAACTTCATACACCTTAAAGTTCATAGGACGAAAAGAGATTTTTCGAGGTCCTTATACTTCATTATGCCTAGCATTGAATAGGCTGGTTATTCACCCTATCACTATCTCAAATCAATAATGCGTTCTATTTGGCGACTAAATGGACACCCGAATTGGACTGAACTAATTGTCAGGCTATTGTTCTCTTGTTTCCGCAAAGTCATAGAGTAAGACATCGATTTATCAATAAGATCAATTCTTTTGATTGCATGATGAACTCCCCTGAAAAACATTGGCGCACGTGTAAACGAGGTGCTCTACCAACTGAGCTATAGCCCTTGTGTTTGTGCTACGCTACATATTTTAGCACGTAGAAAATTTCTTGTCAAGATGCGGATTCCATGATCCAACATCGTAGCTCTTTGATCTGTTTGATTCATATTGCTTAGAAGTAATATTAGATTTATAATCTATGTGATGGGGTTTCTATTTGTTTCCCTTTGTGATGATAAATGACCTACTTAACTCAGCGGTTAGAGTATTGCTTTCATACGGCAGGAGTCATTGGTTCAAATCCAATAGTAGGTAGAGCTTATTAGATACCAGAGTCGATGGTATCTAATAAGTTTTTCTACTCACCCCTAATCTTATTAAAATTAGAAAAAAATCTTATTCTTATATTTCTTATATTAAAATTCTTATTTATATTCTATTTATATATTTATATTCTATTTATAATAGAAAATAGAAAGAAATATTCTATTTACTATTTCTATTTACTATTTATATATTATATATTTATTTTATATATTTATTTAGAAATAGAATATTTATAATAAATAAATATTTATAATAAATAAATAAAATAGGATTCTATTTCTAAACAGAACTTCCTTTTTATTATTATTAGGCTTATGCGGACATACCAACTCGTTACGAAATCATATTGATAGCCTCTACTCGTGTCCTAGCTCGTCTGAGAGCTAGATTTGCCTCAATTATTTGTCTCTTTCCTTCCGCTTTCCTCAAATTAGCTTCTGCTATTTCAAGAGTTTGCTGAGCTTCTTGTGGATCAATGTCACTACCCTTCTCAGCATCATTTACTAAAACTGTGATCTCATTATTGCCTATTCGAGCAAAACCGCCCATCAGAGCCATCGTTAACCATTGGTCGTTAAGTCGTATTCTCAATATACCTATATCTACAGCAGTGGCAATAGGAGCGTGGTTTGGTAATACGCCGATTTGTCCACTATTAGTAGATAAAACGATTTCTTTTACTTCGGAATCCCAAACAATTCGATTAGGAGTCAGTACACAAAGATTTAAGGTCATTTCTTCAATTTGTTCTCCGTTTCTAAGTTCATAGCTTTCGCGGTAGCTTCATCGATGTTACCTACCAAATAAAAGGCCTGCTCGGGAAGCCCGTCTAATTCTCCGGAAAGGATCAATTGAAACCCTCTAATTGTTTCTGCTAGACCAACATATTTTCCTGGCGAACCTGTAAATACTTCGGCTACGAAAAAGGGTTGTGATAAGAAACGCTCAATTTTTCGTGCTCTTGCTACGGTTAAACGATCTTCTTCGGATAATTCGTCTAACCCAAGGATAGCTATAATGTCCTGAAGTTCTTTGTAACGTTGTAAAGTTTGCTTAACTCTTTGCGCAGTTTTATAATGTTCCTCGCCAACGATTCGAGGTTGGAGCATAGTTGACGTTGAATCTAAAGGATCTACTGCCGGATAGATACCTTTGGCGGCTAATGCTCTTGATAGTACAGTAGTTGCATCTAAGTGTGCAAATGTCGTGGCAGGAGCGGGGTCAGTCAAATCGTCTGCAGGTACATAAACTGCTTGAATCGAAGTTATGGACCCTTGTTTGGTAGAAGTAATTCTTTCTTGTAAAGAGCCCATTTCGGTACTAAGAGTAGGTTGATAACCCACAGCGGAAGGCATTCTACCCAATAAGGCGGATACTTCAGATCCTGCTTGGACGAAACGGAAGATATTGTCAATAAATAGAAGTACGTCTTGCTTATTAACATCTCGGAAATATTCCGCCATAGTTAGGGCAGTCAAACCAACTCTCATACGAGCTCCGGGCGGTTCATTCATTTGCCCGTAGACTAGAGCTACTTTTGATTCTGCAATATTTTGTTCATTAATAACTCCGGATTCTTTCATTTCCATGTAAAGATCATTTCCTTCACGAGTACGTTCACCTACGCCACCAAATACAGATACACCTCCATGAGCTTTTGCAATATTGTTGATCAATTCCATAATGAGTACGGTTTTACCCACTCCAGCTCCCCCAAATAGTCCTATTTTTCCTCCACGGCGATAGGGGGCTAAAAGGTCTACCACTTTAATTCCTGTTTCAAAAATAGATAATTTTGTATCTAACTGTATAAAGGCAGGTGCGGATCTATGAATAGGAGATGTTGTGCGAGTATCTACAGGACCTAAATTATCAACGGGCTCCCCAAGTACATTAAAAATTCGTCCGAGAGTTGCTCCGCCGACTGGAACACTTAAAGGAGCCCCTGTATCAATCACTTCCATTCCTCGCGTTAGGCCATCTGTAGCACTCATAGCTACAGCCCTAACTCGATTATTTCCTAATAATTGTTGTACCTCACAGGTTACATTAATTGGTTGACCGGCAGTATCTCGGCCCTTAACGACCAGAGCGTTGTAAATATTAGGCATCTTGCCTGGTGGAAAAGCTACATCCAGTACTGGACCAATGATTTGAGCGACACGCCCCTGGTTATTTTTGTCAAGTGTGGAAACCCCAGGACTAGAAGTAGTAGGATTGATTCTCATAATAATAAAGTAATAAAGTCAAATAAAAATATATAAAAAAAATGAAATAGAAATATAATATATATATTTCTATTTCATTTTCATTTCGAATTTTTTGCGAAAATGAAAATAAAATGTCCGATAACAAGTTGATCGGTTAATTCACTAAGAAATGGGAGTTAGCACTCGATTTTGTTGGGACCATCCAACCGAATCCAATTCAATTGTTGACTTATTCCTTCCTTTATTTTCGATTTCTTTGATTCAATTTCAATGAGTGAGTTCAACCAATCTATTTTCAAAATATCAAGTAAATGAACAAAAATTTGTAGAAAGTCTTTTATTTGTCTATCATTATAGACAATCCCATATATATTATCGACGGAATTCGAACCTGAACTCTAAACTCGATTTAATTTATGGTTCATTATTTCATTTCTATCGCATTGGCTATCGCATCGGCCCTTTTTTTTATTTAGCATATTGGTTTAGCCTATTCTTTTTTTTCTTATTTATACCCTTTCGTCTATGAATTCCGCATATTTTCACATCTAGGATTTACATATACAACATATATTGCTGTCAAGAGTGAATTTTGATTATTATTTAGTCAATTCAAAAAAGGTTAAGGCATTAGAAACTTGAAAAAGAAGGGTTGGGTTGCGCCACATATAGGAAAGAGTATACAATAATGATGTATTTGGCGAATCAAATACTATGGTCTAATAACGAACCATTTTAATTAGTTGATAATTTTATGAAAGATTCCTGTAAAAGGGTTAATTAACGCCTAATTCATGTCGAGTAGACCTTGTTGTTGTGAGAATTAGTAATTGATGAGTTGTAGGGAGGGACTTATGTCACCACAAACAGAGACTAAAGCAAGTGTTGGATTCAAAGCTGGTGTTAAAGAATACAAATTGACTTATTATACTCCTGAATATGAACCCCATCCGCATGATATCTTGGCAGCATTTCGAGTAACTCCTCAACCTGGAGTTCCACCCGAAGAAGCAGGGGCCGCGGTAGCTGCCGAATCTTCTACTGGTACATGGACAACTGTGTGGACCGATGGACTTACCAGCCTTGATCGTTACAAAGGACGATGCTACCACATCGAGCCTGTTCCTGGAGAAGAAAGTCAATTTATTGCTTATGTAGCTTACCCATTAGACCTTTTTGAAGAAGGTTCTGTTACTAACATGTTTACTTCCATTGTGGGTAATGTATTTGGGTTCAAAGCCCTGCGTGCTCTACGTTTGGAGGATTTGCGAATCCCTCCTGCTTATTCGAAAACTTTCCAAGGCCCGCCTCATGGTATCCAAGTTGAGAGAGATAAATTGAACAAATATGGACGTCCCCTATTGGGATGTACTATTAAACCGAAATTGGGGTTGTCCGCTAAGAACTACGGTCGAGCAGTTTATGAATGTCTTCGCGGCGGACTTGATTTTACCAAAGATGATGAAAACGTGAACTCCCAACCATTTATGCGTTGGAGAGACCGTTTCTTATTCTGTGCCGAAGCTATTTTTAAATCACAGTCTGAAACAGGTGAAATCAAAGGACATTACTTGAATGCTACTGCAGGTACATCCGAAGAAATGATCAAAAGGGCTGTATTTGCCAGAGAATTGGGAGTTCCTATCGTAATGCATGACTACTTAACGGGGGGATTCACTTCAAATACTAGTTTAGCTCATTATTGCAGAGATAATGGCCTACTTCTTCACATCCACCGTGCAATGCATGCAGTTATTGATAGACAGAAAAATCACGGTATGCACTTCCGTGTACTAGCTAAAGCTTTACGTCTGTCTGGTGGAGATCATATTCATGCTGGTACTGTAGTAGGTAAACTTGAAGGGGAAAGGGACATCACTTTAGGTTTTGTTGATTTACTACGTGATGACTTTGTTGAAAAAGACCGAAGTCGCGGTATTTATTTCACCCAAGATTGGGTTTCCCTACCTGGTGTTATTCCTGTGGCTTCCGGGGGTATTCACGTTTGGCATATGCCCGCTCTAACCGAGATCTTTGGGGATGATTCTGTACTACAGTTCGGTGGAGGAACTTTAGGCCACCCGTGGGGCAATGCGCCGGGTGCTGTAGCAAATCGAGTAGCTCTAGAAGCCTGTGTACAAGCTCGTAATGAGGGGCGTGATCTTGCTCGCGAAGGTAACGAGATTATTCGTAAAGCTGCCAAATGGAGTCCTGAACTGGCTGCCGCTTGTGAAGTATGGAAGGAAATCAAATTTGAATTCGCAGCAGTGGATACTTTGTAATCCAGTAATTACTGGTCGGTCCCTTAAATTGAATTGCAATTAAACTCGGCCCAATCTTTTTACTTTTAGTAAAAGGATTGAGCCGAATACAACTATTGTTTTGCATAGATCTTAGATCTACAAGCAAAATCCTAAATAAAAAATCGAAGACTAAAAAACTCAAAAGTTTCTTTGGTTGTGCTGGATCCACAATTAATCCTATGGATCTCTAGGATTGGTGTATTCTTATATATATCCCGTAGCTTAGGACCGCGGATAGCGAGTCAAGTATAAGAACCCCTTCGACCCATCCTGTATATTGTCCTTTTCTTCCGTATTGGAATAGAAACTTTAGACGAGATTTTACGAAAAAAATTATTCATGGACATGGAAAATAATAATTATTTTTTTTTCTGTTGATGCGAATTTCACACGACATGGAAAAACCCCTACTTTTTTTATTTCTATTTTTATATTCCAATAAAAAAAAAGTTCTATCATATAGAGTGAAGTGATACCCGGATTCTTACAAAGGATAATCTTTTATTTCTCACTCGTTTTTAGTTAATAATCCGAGTTAGGATTTGAAAATTTCAAATGACTTTTCATCGAATGACTATTCATCTATTTTTTTATTTCATGCAAATAGGGGGCAAGAAAGCTCTATGGAAAAACGGTCGTTTAATTCGATGGTGTCTAAGGAGGAGTTAGAACATAGGTGTGGGCTAAGTAAATCAATGGGCAGTCTTGATCCTATTGACAATACCAGTAGCAGTGAAAATACGAGTCTAAATTATACAGAAAAAAACATTCATAGTTGGAGTAATGGTTCTAGTTACAGTAATTTTGATCTTTTATTCGGTATCAGGGACATTCGGAATTTCATCTCTGATGATACTTTTTTAGTTAGGGATAGTAAGGGGGATACTTATTCCATCTATTTTGATATTGAAAATCAGATTGTTGAGATTGAAAATGATCATTCTTTTCGGAGTGAACTACAAAATTATTTTTCTAATTATTGGAATTCTAGTTATGGGAATGGATCTAAAAGTGACGATACCCATTATGATCTTTACATGTATGATACTAAATCTAGTTTGAATAATCACATTAATAGTTGTATTGACAGTTATCTTCATTCTCAAATGCGTATTGATAATTCTGTTTTAAGTGATAGTGACAATTACAGTGATAGTTACATTTTTGATGAAAGTCAGACTACCACTAACGCAAAAGGTAGGGATAAGAATCTTGATGTAACTAAAAAATACAGGCATTTATGGATTCAATGCGAAAATTGTTATGAATTAAATTATAAGAAAATTTTGAAGTCAAAAATGAACATTTGTGCGGACTGCGGATATCATTTGAAAATGAGTAGTTCAGAGAGAATCGAACTCTCGATTGATCCAGGTACTTGGGATCCTATGGATGAAGACATGGTCTCTCTAGATCCCATTGAATTTCATTCGGCGGAGGAACCTTATAAAGATCGTATTAATTCTTATCAAAAAGAGACAGGGTTAACCGAAGCTGTTCAAACAGGTATAGGTCAACTAAATGGTATTCCTGTAGCAATAGGGGTTATGGATTTTAAGTTTATGGGAGGTAGTATGGGATCCGTAGTAGGAGAGAAAATCACTCGTTTGATTGAGTATGCTACTAATCAAAGTTTACCCCTTATTATAGTGTGTGCTTCCGGCGGGGCGCGCATGCAAGAAGGAAGTTTGAGCTTGATGCAAATGGCTAAAATTTCGTCTGCTTTATTTGATTATCAATCAAATAAAAAGTTATTCTATGTATCAATTCTTACATCTCCTACTACTGGAGGGGTGACAGCTAGTTTTGGTATGTTGGGAGATATCATTATTTCCGAACCCAAAGCCTACATTGCATTTGCGGGTAAAAGAGTAATTGAAGAAACATTGAATACGACAGTACCAGAAGGTTCACAAGAAGCTGAATATTTATTCGATAAGGGTTTATTCGATCCAATTGTACCACGTAATCCTTTAAAAAGCGTTCTAAGTGAGTTATTTCAGTTGCACGCTTTCTTTCCTTTGAATCAAAATTCGATCGAGCAGTAAGGTCAATTATTATTTTTTTTTTATTTGTTTATTTTTGGCAAAAAAAGTAGTTAGTTATCGTAATCAATCAAAGTCAAAATGATAAAGAATCAATCATAATAGAATAATGGGTATGGGGTTTTCGTGGTTGCCATACTAATTCTATTCTATACTAATTCTATAACTATAATATAAAGAATCAAAAGTTGCGGATAAATTTTTTTACTTTTTTTATTTTACTTCATATTCCATTCCTGATTACTAATCAGAGAACCTCTATTCTATCAACAAACAAGATATTCTTACTTCTGTAACTAAATGGAAAATTTGGCAAATAAAACGAATTTCATCTTCCTTTCTTACATCTGGATATAAAAATTCGAATAAAAAAAAATAGCCTTTTGCATCTTAATAGATTTCCTTGTCAGAGACTCTCCATTTTTACTAACAAGAGAATATCTCTTGGATCAGATTCGTTAGAATCTTTCGGGACTTTGTAAGCAACTCTTTCTTTATTGATATTGAATTAAAAGACAAGGGCAAAAGAAGAAGAAAAGATGAAGAATCAAAAAGTTGATTATCAAACATATATTTTTTTGTGTCGACGGCTAATTAAGTTCATTTAGTTAGTTCTACATTTCTTGCACTTAGTATATACTCACTTAGATATAATTAGTATAATTATATAGAATTAGAATTCTAATTAAGTTAAGATAATTTTAGAACAATATAACAAACAGGTACAAATAGTAAATCGAGGTACCCATTCTATGATAGATCTAAACCTTCCCTCTATTTTTGTGCCTTTGGTAGGCCTAGTATTTCCGGCAATTGCAATGGCTTCTTTATTTCTTCATGTTCAAAAAAACAAGATTGTTTAGGCCGGATGGTGAGGCCAAATCACATTTTTTCAAGACTTAGGCTTGATTGAATCATAACACAGATATCGATTTATTGGAAAGTGGAATATGGTATAATGCGTGATTTCTTTCGAACATAAATGCAAGAACTCTTATGCATGCGAAACCTGATATGATATAGGGGTAATTTAATTTTAACTATTTTCAAATCAATAGATCAGGACCGGTCGGTCCATGTTTGAAATAGAAAGTCAATGTTTGTAGATATCTAGGATGGGGTCATATGAAGGGGACGTTCTTATTTTCGATCGAACGAATTATATGAATTACCCCTACAGGTTCACATTAGAATAGTGCTAGTTGATGAGAGTTACTTCAGAAACAAAATAGCGTTAAGTTAAGTATAAGTAAGGTGAAATTCATTTTGGTTATTCTATCAATTCAATCAATTAAAATTAAATGCAACTAGATTAGTATGAATTGGCGATCAGAACGTATATGGATAGAACTTATAAGGGGGTCTCGAAAAACAAGTAATTTCTGCTGGGCCTTTATCCTTTTTTTAGGTTCATTAGGATTTTTATTAGTTGGAACTTCCAGCTATCTTGGTAGGAATTTGATATCTTTATTTCCCTCTCAACAAATAATTTTTTTTCCACAGGGGATCGTGATGTCTTTCTATGGGATCGCAGGCCTCTTTATTAGCTCCTATTTGTGGTGCACAATTTCGTGGAATGTAGGTAGTGGTTATGATCTATTCGATAAAAAAGAAGGAATAGTGTGTATTTTTCGTTGGGGATTTCCGGGAAAAAATCGTCGCATCTTCCTCCGATTCCTTATAAATGACATTCAGTCTATCAGAATAGAACTTAAAGAGGGTATTTATCCTCGTCGTGTCCTTTATCTAGAAATCAGAGGCCAGGGGGCCGTTCCTTTGACTCGTACTGATGAGAATTTGACTCCACGAGAAATGGAACAAAAAGCTGCTGAATTGGCCTATTTCTTGCGTGTACCGATTGAAGTATTTTGAAATGAACTGAAGAATGAATGCTTTCTCATTCTCGGCTGGGGGTAAAAAAAACTCTACAATCCCCTTTTTGTATAACTTTAATTTTTATATGGTATAACTTAACGAAAATTTCGTCAGAACGTCCATTCGAGTCAAAGCAAACGTATATTATATGGAACATCCAAAAAAGGGGTGGGATTTTTTTTGTCTGCAAAAAAGATATTTTATGCGTATGGAAATCCACTCGACGCAATTCATTAGCGAAAAAAGTAACAAATCGAAATAAGGATAGATTCATCCCAAAACATTTCGAAATAAAGAAATTTTTTATTTTAGTTTCAATATTTTGAATTGATAGGTTAGAGACAAATAACTCATGTAAATTAATTATCTCTCTTTTATCGATGTTTCGTTTTCTCCCTTCTTTCGCTCTCTTCTCTTTAATGAATGACCCAACATTTGGATTTCTTATAACGATAATTATCCAATTTCTGTCTTGTTTTGCTACCCCTTTTTGATCATCACATTCAGAAAATTCTCTCAATTATTTTTGTACTATGGTAGTCGGCGAATTTTGAAATATTTGGAGAGTTTTTCGTCTCGAAATCCGAATTCATTAACTAAAGCGGGTTTTCGGGGATTCATCGAAAGGAAGGAATTGCTTCGAATTTGACCAATTGAATGAAATAACTGGAAACTTTATTTTTTCTTATTTTCGCATTCGAATTCGAAGTGGACTCTTATTCGATTTCTGTATTCTTGCAAGCTTCTTCAAAATTCTCAAGGACTAATTCTAATTACCGAATCACAAATAAAAAACAGAGAATGATTCGATACCTTGGAATAGAACTCATTTTGATGAAAAATCAAATATTAGATCACATAGAGTCGACGAAAGAGGCCGCTTTATTAACAATTTCTAAATGAAAAAAATGGCAAAAAAGAAAGTATTTATTCCCCTTCTATTTCTTGTATCTATAGTCTTTTTACCCTGGTGGAGCTTTCTAGCATTTAATAAAAGTCTGGAATCTTGGGTTACTAATTGGTGGAATACCAAGCAATCCGAAACTTTTTTGAATGATATTCACGAAAAGAGTATTCTAGAAAAATTCATAGAATTCGAGGAGCTCTTACTGTTGGACGAGATGATAAAGGAATATCCGGAGACACATCTAAAAAAGCTTCGTATAGGAATCCATAAAGAAACGATTCAATTGATCAAGCTGCACAATGAAGATTGTATCCATACAATTTTGTCCTTCTCGACCAATATAATCTGTTTCGTTATTCTAAGTGGTTATTCTATTATAGGTAATGAAGAACTTATTATTCTTAACTCTTGGGTTCAGGAATTCCTATATAACCTAAGCGACACAATAAAAGCATTTTCTATTCTTTTATTAACCGATTTATGTATCGGATTCCATTCACCCCATGGTTGGGAACTAATGATTGGCTCTATCTACAAAGATTTTGGATTTTCTCATAACGATCAAATTATATCTGGCCTTGTTTCCACTTTTCCAGTCATTCTCGATACAATTTTGAAATATTGGATCTTCCGTTATTTAAATCGTCTATCCCCATCACTTGTAGTGATTTATCATTCAATGAATGACTGAAAAAAAGGGTCTACTGATATTAATCCAATTAGAATGTTTGGTACTTTGGGCATAAGCATTCCAAATCGTACTGACTCTTTCTACCCATCCAAGGCAGGAAGGTCCTCCTATATTCCAGTAAGATTATTCCAGTCAAGTAAATAGCAGAATCGTGGATAGGGAACTATACTAGCGACCTACCCAATTTATTGTAGAAATTTTCGGGATCAATAATTGGACCATGCAAACTAGAAATACCCTTTCTTGGATAAAAGAACAGATTACTCGATCCATTTCCGTATCACTCATTATATATATAATAACTCAGTCATCCATTTCAAATGCATATCCCATTTTTGCACAGCAGGGTTATGAAAATCCCCGAGAAGCGACCGGTCGTATTGTATGTGCCAATTGTCATTTAGCTAATAAGCCCGTGGATATTGAGGTTCCACAAGCGGTGCTTCCTGATACTGTATTTGAAGCAGTTGTTCGAATTCCTTATGATATGCAACTAAAACAAGTTCTTGCTAATGGCAAGAAGGGAGGTTTGAATGTAGGAGCTGTTCTTATTTTACCCGAGGGGTTTGAGTTGGCTCCAACCGATCGTATTTCTCCCGAGATGAAAGAAAAGATAGGCAATCTTTCTTTTCAGAGCTATCGACCAAATAAAAAAAATATTCTTGTGATAGGCCCTGTTCCGGGGCAAAAATATAGTGAAATCACCTTTCCTATTCTTTCACCGGACCCTGCTACTAAGAAAGATGTTCACTTTTTAAAATATCCCATATATGTAGGCGGTAACAGGGGAAGGGGGCAGATTTATCCCGACGGAAGCAAGAGTAACAATACTGTTTATAATGCTACAGCAGCTGGTATAGTAAAGAAAATAATACGAAAAGAAAAGGGCGGATATGAAATAACCATAGGGGATGCCTCGGATGGGCGTCAAGTCGTTGATATTATTCCTCCAGGGCCAGAACTTCTTGTTTCAGAGGGTGAATCTATCAAACTCGATCAACCATTAACGAGTAATCCTAATGTGGGTGGATTTGGTCAGGGAGATACAGAAATAGTACTTCAAGACCCATTACGCGTCCAAGGCCTTTTGTTCTTCTTGGCATCGGTTATTTTAGCACAAATTTTTTTGGTTCTTAAAAAGAAACAGTTCGAGAAAGTTCAATTGTCCGAAATGAATTTCTAGATCAAGTTCATAACAAGAACCAAATTCTTCTTTGTTTATAGTAATGTACGATTATGTATGATCACGAAATAAAAAAAAGTACAAAAGCCCTCTTCTTTGCTTATACTTTTTTAACGAGATGTCGGGAATTAGTTGTACTACATTCTTAGTCATACTATGTATATTGTGCAGAAGACTATTTGACTTTTGAATTTTACTTTTTTTTCAATACAAATTGTAATGATGTGACTATGTAACTCTTATCAGATTTAAATGTTATCGAATGCATCAATAGTTTTTTCTATTCGAGAATCAAATTCGATTAGATACTAGACTAAACATAGAATAGAACACACAGATAAATGAGGGGAATAAATGAGTCTAGGAGGGATTCTTTGCCTTCCTAATCTTCGACACAAGAAAGGGAATTTTACACATCCTTTTCTTGTGTCGAAATAGTAATTATTCGTGATGGTCTTCGTCAAAGACGCGTAATTTTCGATTTTTTTATAGATTTTTTGGGGGGGGTTCAGGAGATTTAACCGAACTTTTTTTGTCTTACTATTACGCATATAATAGATAGAACAAAGTAGTGGACAAACAAAAAAAGAGAGAGAATTTTATTGAGCAACAAATAGAACTTCTTCAATGAACTTATAAAAAAATTTCTTTGATTTGATGAATACAAAGAGGATATACTACTAGAAAAAATTCTAGTAGTATAGAAGGGTTTTCATGCTATTTGATCTACCTATTTTTTTACTTTACTTTATTTTTATGCGATAAAATATTCTAATTATTACGAATTTAACGGGTACCTCCCCCTTCTTTGTTTCTCTAATTCGAGGGGGAAGGAGGGTCCCGTTGAGTTCTTACGCTTTCATATCTATACCGCAGTTCATCCGATTACTACAGGGATGAACTACTACAGGGATGAACCCAATCCGGAATATGAACCATAAAAGAAAATGCCTATTAAACCGATCACAAGAATGCCGGTTACAGTACCTATTATCCAAAGCGGAATCCTTCCAGTAGTATCGGCCATTTACCCCACTTCCCTCCACATTTCATCAAGTGGTCATGCTAGAGACATAAACAGTCATGGGTAATTATGAGATGATCCTTCCAAATGGGATAAGAAAATTCCTACTCTATATCTTATTCTCCCTCGTTCTTTTTAATTGAAAAAATAATTGGAAAATAAAACAGCAAGTACAAAAATGAGTAATAACCCCCAGTAGAGACTGGTACGATTCAATTCAACATTTTGTTCGTTCGGGTTTGATTGTGTCGTAGCTCTATAATTCGGATTAGGTTTTTTTTATCGTTGGATGAATTGCATTGCGGATATTGAACCCAAAAAAGAAACGGTAGGTACAGCTAGTCCGTGAACAGCCAACCATCGCACTGTAAAAATGGGATAGGTTCGATCTATGGTCATTGGGCCTCCTAAAAGGATCTACTAAATTCATCGAGTTGTTCCAAAGAATCAAAACGGCCAGTTATTAATGGAATTCCTTGTCGGCTCTCTGTAAAATACTCGTTTGGCCGAGGGCTTCCAAACACATCGTAAGCTAAACCTGTGCTGACGAATAACCAACCCGCAATGAATAGGGAAGGTATAGTAATGCTATGAATGACCCAGTATCGAATACTGGTAATAATATCCGCAAAAGAACGTTCTCCTGTGCTTCCAGACATGCTCAGCTCCACATATTCTTGTACAGGCAAATGTAAATCGATTCCGTAAAAGATGAGATCCGTAAATTTAAATTTACGAAAATTCTTTGTGGGATCGTCAATATTGTACCAAGGGTGTCTTTAGAGTATACCAAATCAGTATAGCCGTCCTTCTTCTGACACAGCAAGGCAATTTCAATTAATATTGAAACTAAGTATACGTATTAGACAATTTATTTTTTTTTTGCTTGTCAATATAGAACTATGCGCCATTTAATAGAAAAATCCTCCAATTAAAATAGTATAGAGGTTATCTATTGGCTTCGGACTAGAAACGGAAATCGGGTAGAAATCGGGTAAGGGATCAATCTGACAAGTTGGTTTCTAATAATTCATCAAGGAGAGTATTATATTCCCACAATTGAATTAGACGCGAAATCTAGAACTCTCTTTTTTGTGTTTATAGAAAGAAAAGCTTTTTTGGAATCCTTTTTTATTTTAAGGAATTGATTAGTCGTCTAGTACCAAATAAGAGTAGTAGGTAGGAGATAGTATTCGATGAAAGAAAACAAAGAATACAAGAATTGAATTGTAAGAAATTAATCAACATTCGTGATGCATGCATTCCTGTATTAGATCCAAGGGTTCTTTCTTGCTCTAACTACAGGGATGAGACTTGATAAAAAAGACGAAAAGAAAAAATATAGAATCTATATAAAAAATAAAGATAATCAGAATGAATCATCTTAGAATCGATTTGGACTAAAATCAAAATTTTCTTTTTTTTGCGTGATCGTGTTGATAGCCTTTTTCTTATCATTCAAAAAAAAAAAGATTCTGTGAATAAACTCGATTGCTGAATCTAATGAGTGAAAATAAACATAAGATCGCTTTTCGTCCTAAACTAAAAAAAAAGAAATAAAATAAATGCTCAAACTAGAAATGCTTTTCCTGTTTTCTTCGATAGTGAGTGGGAGTTTTTTTTTGAACAAAGTGACATGACATAGTTCTTATTTTTTTTTATTAGTTTACTCCAAGAGTTGCTCAAAAAATATGTTGATTAGAAATCACGGAATCTGTAGATGTCACAGACAATGAGTCGATTTAGTTTTCTACCTCTCTTCCTTTATCTTATCTTTCTTAGTTATATCTATAACGTAATAAATCAATGTAATGATTGGGGAATCAATTGAACTTATTCTTTCAATTGGTATTTTTGCTTATTTTCGCTCCTATCTTTCACAAAAAAAAGTAAACTTAGGTAAGTGCTTTATAAATATATGTATAAAAAAAAACATATTTGATTTAGCTCCTTCATGCCTACTCTAACTAGTTATTTTGGTTTTCTACTAGCAGCTTTAACTATAACCTCAGCTCTATTTATTGGTCTGAGCAAGATACGACTTATTTGAAATAAATTGAATCAACAATTCATAATCATAAAAAGAAATCTTTCTGTAGGATTTCATGTATTTTTTAAGCTCTTTATCGCGACAATTGAAAATTGTCTGTCGGTTATTGAGATTCATGGACAATTAGGATGAATATTTAGGGATAGATATTACCTCCCTTTTTTCCTTTCAAACAAATTGAAATGATTGAAGTACTTCTATTTGGAATCGTCTTAGGTTTGATTCCTATTACTTTGGCTGGATTATTCGTAACTGCGTATTTACAATACAGACGTGGTGATCAGTTGGACCTTTGATTAGTTAACAAATCTTTTTTGATTGACCTCCTTTCTTTACGCCACAGGAGGTCAATTTTAGATTCCTCTTCAAGGTAGTGAAGTTATTTCAGTCTAATTAGAACTAACAAGAATGGAATCACGCTCTGTAGGATTTGAACCTACGACATCGGGTTTTGGAGACCCACGTTCTACCGAACTGAACTAAGAGCGCTTTCTTATCACAGACAGTAAAGAGATTCCTTTTGTAACCCAATACTACATCCTGCATGCATATACTATCACATATATAAGTATCGCATATATAGTTAGAATTGTATATGTGTTATATGTATAGATATATTTATCTAATAGTATAGTTTATATAATACTATAGTAGTTATAAAAAGTCTTATAAAAATCATAAATAAAAAATCATAACATAATCATAATAATAAATAAATATAAATAATAATAAATAATAGAAATAAAAATCAATTATATATTCATATTACATTAATATTATATATTAATATAATAGAATAGTATTAATAGTCTTAGAATACTATTCTAATAATGACAGATTATGTATGTCCAATTTGAATCGATTTCATCGATCTCAATTAATTCCTCTTTACTTCTCAGAGGAAAAGTAATAGGTAGGGATGACAGGATTTGAACCCGTGACATTTTGTACCCAAAACAAACGCGCTACCAAGCTGCGCTACATCCCTTTTAATAGGTTTACAGTGTTATTGTAAAGAATCCTTTTCTTTTTTTCCACATCATTATTTCTCATATTTAGATACACAATAGATCTTGCCATTTTTTCTTTTTTTTTTTCATATCATATAGATATAATATAAAAGTCTTTGGCGCTGTAAAGTAAAAAAAGGCATGCTCAGTAGGAAAGATGCATCTTTTTACTTTTTTTTCAATTTAACTTAAAATAAAGGTGGAAAATCTTATCTATCGGATTGTTGTACATTTTAATTTGCCTTAGGAATTTCATGTACAAATACAAGCGGTTTTTCTTTTTAAATACATCTACATATGCATCTGATCATCTATCATATATGTATTATTCTTATGTGTTACAATATATAAATAAATAAAAAAAAGAAGGAGGATTTTCAATGCGAGATCTAAAAACGTATCTCTCCGTGGCACCGGTACTAAGTACTTTATGGTTCGGATCTTTAGCAGGTTTATTGATAGAAATCAATCGTTTTTTCCCGGATGCGTTAACATTCCCCTTTTTTTCATTCTAGTTCTAGTTATTGACATGGTAAGGGGTAACGAAGATTAGAGATAGAATCCACTATCTGTGACTAATCCCCCGCCCTTTCTCCCTTTGACCTTCTATTCGAAAAGGGAGAAAGAAAAATGGATTCAACCTCAGCAAAGCTTGGGCTCAAGCTCGAATTTCAAATTCAATTTATAAATAAATAAAATAAGAGGGAGAACGGAAATTCAAATGTGGGTCTAGGGCAAAAGTCTCATAGACGAGACTTAAATGAAATACTGTACTGTGATTAGAAATATAGTTAGAAATCGTTGGATTACGTATTCAGTATTATACTGAATTCTATTTTCTATTAATATTGAATATTATTCTTATTGTATTGATATTCCTCTATTTACTGCAACAAAATCTTTTTAAGTGTATTTCGAGTTAGCAATTTCTATTATTATTTATTCTTTTTTTCTTTCTCTCCGCTTCGGTGAAAGAGTTGCTTGAATAAAAAATTCAAACACAAAAAAAGGGGGTTCATGGCCAAGGGTAAAGATGTCCGAGTAAGCGTTATTTTGGAATGTACTAGTTGTGTCCGAAAGAATGTTAATAAGGAATCAAGGGGTATTTCCCGATATATTACTCAAAAAAATCGACACAACACATCCAGTCGATTGGAATTGAGAAAATTCTGTCCCTATTGTTACAAACATACAATTCATGGAGAGATAAAGAAATAGATCGAACCGAACGTCTGTGTATCACCTTTTGAAGGAAAAGTACAAAAGGACATATATTATACATATATTTCATTATATATTATACATATATTTCATTATATGCATTTACCTTATTTCATTATATGCATTTACCTTTTTAAAAAATTATAAGAAAATGTAATAAACATACATATTATTATATGCAATATACATATTATTATATGCAATAGATAATAATTATTAATATTATATTAATATTAAAATTATATATTAAAATTATATAGAATTCTATTTAAATTTTATTTAAAATACTAAATTTTTTTTAAATACTAAATAATCTAAATAATAAAAAATGAAATATTAAATAAAAAAAAGAAAAAAATAGAATTATATAATATTATAAATAGAATATTCTATTTATATTATAAAATTGTATATATAAGAATATATCAAAATAATATATAAGAATAATATATAAAAATATATAAAAACCGAACTAGAAACTATATCGATCTTCTATATAATAGAAGCGTTCTATATAATAGAAGCGTATAAATATAACATATATAATATAAAATAAACAAATTCAAATTAAAGAAAAGAAAATAATAAGAAAACCAAATCCTATTTTTCATTTTTTTTAGATCCGACCGAAATAGGATTTTCGGTAGAATATTATTTATATTATAAGGAATAAACTAAACAAACCATGGATAAATCCAAGCGATCTTTCCTTAAATCTAAGCGGCCTTTTCGTAGGCGCTTACCCCCGCTCCAATCGGGGGACCGAATTGATTATAGAAACATGAGTTTAATTAGTCGATTTATTAGTGAACAGGGAAAAATATTATCTAGGCGCGTGAATAGATTGACTCTGAAACAACAACGATTAATTACTATTGCTATAAAACAAGCTCGTATTCTATCTTTGTTACCCTTTCTTAATAATGAGAAACAATTTGAAAGAGCCAAGTCGGCCGTTAGAACTACGGGTTTTCGAGGTTTTCGAACAAAAAAACAATAGCTCTTTAGTCAATTCAATTGATGTTTTGTTCGAAAAAAATCCGATAATCCGGATTTGATTGTTGTCTCGGAAGAAAAATCGAGGAAGAAGAAATCTTTTTTTATTGAATGGCTTTGTTCATTGTTCATTTTGACTACTTTATTGTTATTGTATTTTATATTTTATTTTATCGGACTAATTTCATTTCTATTCTATCTTCCCTTCCCGGAGTTCCTTCTCCGGGGAACTTTGTTTAAATCATTTCGGGTGCTTCTTTCCAATCTTCTTTTTTTATGATCTCATTGGAAATACTATAAAGACAATTCCTATTTAATATAGCTATTTGTGCAAGTATTTTACGATTAAGAATCAACTGTCTCTTGTACAGATCGTGTATAAATTTACTATAATTATAGTATACGCCTATTTCTGTTTCGCGAATTGCTGCGTTTATACGAGTAATCCACAAACGACGAAAATCTCTCTTTTTCCTATCTCTATCTCGATGAGCCGAAAACAAAGCTCTTATTTTCTGTTGAGCAATAGTACGAATAGTTTTTGAATGAGCCCCTCGAAAGCTTGATACAAATAAACGCATTTTTTTTCTACGTCTCCGAGCTATATATCCTCGTCTAACTCTGGTCATTGAATAAATGAAACTTTGCTAAATAATTAATTGATTTTCTTTCTTTGAGTTATTCTTTTTTCCCCGCTGGGTATATTAATAACAAAACGGATTTTTCCAATGTATAAAAAGAATTCCAATGGCTTTTGCTACTATAACCTTCCCGACCACTATTTTTTTCTTTTTTTTCTAGGCATTTCGCCTCAACTCAAAATGAAATAAATAAGAAATTGGATTGATACTAGGTATCAAAAAGAAAAACGTAGTAAATCGAGATGAATAAAGAAATAGTGGGTTCCTTCGTTTCTATGGTTACTTCTTAAACGGTGAGGTCCTCTCTATACACCGGAGCCCTTTACTTCGTTTAGTCAACGTTATTGGTAACTTGTACAATTCAAAATCTTTGGCTCTACCCATGAATTATCCAGTAATAGGTCTTTCACAACGAGATCCACCTATACAGTAACGGTATTTAATTTGGAAGGTTAGCTGGATAGCTGACCCTGTTAGTCCGTTTTGCAAAAATGGGAGCATAATCTTTTTTCTTTTAAAATAGTACTTTCCCGCTTAATGTATAGCATTTGCTACCAATGGGAACTTGCTTCTCATCTTAAATTGAGCTGATTGGGGTTACACCAAGGGAAACCATAAATTTCGTACACAATAGGCGGATATGATAGATCTTTTTTTTCGATAGTGACCAAAGTTCTTCCATTTTATCCTATTCACTGGTAATGATCATTGATACTGGAAAATTGATTTCTTTTGTTGGCCCAGCTCATAATCTAAACGAGTCGCACATACACCCTAGTACATGTTCCTCGGCGCTGAGGACATCCCCGAAGAGCGGGGGATTTCGTGACGTTTCTGATTGGCTGTCTTGTGTTTCTAATAAGCTGTTTAATAGTTGGCATGCTGAATCATTTACATAAGGGACTGGTTTAGATCAATCCTAACCTGATGATTATGAGTTATTTCTAGTTAATATTCTATTAACTAGAAAGTAAAAAGATAAAATATCAATTTGCGAATTTGACTACTTCTACTCCTTCCATTTTTCCTTCTTTACTATTTCTACTCCTTCATTCGCTATAAGATCAATAATTCCGTGAGCTTGGGCTTCTGTTGCTGACATAAAAACATCCCTTTCCAGGTCTTCGGTTAGAACCCACAAGGGTTGGCCTGTTCTTTGTGCATAAACCTTTGTGAGGGTTTCTCGCAAAGTCAATAGTTCTTCCGCTTCCATCATACATTCTCCCGTTGATCCCTCATGAAAAGAACTAGCAGGTTGATGAATCATTACCCTGATGATATAAAAAAAAGAGGGTTCCTCTATCTCGCATGATGAGGCGAAGACAAAAGATAGAGAATAACAATAAACTTGAACAACCGTACGTGCATCTTTTGCGCATTGCATACGGCTCGACAATGGAATTTACTTTTCTCTTCCATCGAAAAAAAATAGAATCGATCAGATCCAGATCAGTAAATCATCCAATTACCACCCTTCTTTTCGTGAGTTCAAAATACTATGATGGCTCCGTTGCTTTCTATATTCATTTCGTTCATTTCGTCTGTAATTCAGCAATCCCAAAGTTTCTTTTTGATCCGAAATAAGTAATTTTTTTTTATTTTCGTACTCTTTCAAACATAAATATTGTTAGGATTAAGAGTCTTTTAATATAAAAATAAAAAAGTTTGTGACGCTGAAATGGACTCCGGATAAATTCAAAATCGGGAATACCCTTTATCTCATACTCCTCTCTCGATACATAATCTAATGTTTTGAAAAAAAAACTAACAAAATTTTGCATATCGAATTCAAAGTGCCATGCTATTTTTACTATTTTTACTTAACACTACTTAATATATATTGATATTTCTTATGGTGAAGGCATAGTCTTTTTTTTCTTTCAAATAAAAAACTCATTGGCGCCAACCAAAGCCAAGCGTGAGGGAATGCAAAACGTTTGGTAATTTCTCCTCCGACCAGGATAAAAGATCCCATTGAAGCGGCTATTCCCATGCATATTGTATATACATCTGGTAGCACAAGTTGCATAGCATCAAAAATAGCTATTCCGGGTAATACCCATCCGCCGGGACAATTTATAAACAAATACAAATCCTGGGTCTGATCCTCTATACTGAGATATACGATAAGACCAACAAGGTAATTCGAGATCTCGGTCGTAATCTCTTGGGTTAAAAAAAGTAATCTTGCTCGATAAAGTCGGTTGATTAGGGTCAAATTGTATCCCTTAGGAACCGTACATGCACCTTTTGATGCATACGGTTCAAAAAAATGTGAAAAATAAAAAAATCAATGTGTAGATTACTGCCCTCTTCTTTTTGGATAGCAGTTCGTTCTTTGTAACTTCTACTGAGGGGGATTTGTCTTCTATTTTTCAATAAATATGAGTTTTTCATCCTTTCCTTATTTTTACTATCTATATAAACTTTTTTTACTATAACTATATATATAAATAATATATAAATATCTAACTATATATAAATATATAACTATTATTTATAGAAATATAGAAATTAGAATTCTATATAATATAGAAATAAAAAAAAAGATAATATAATATAGTAAATTAAAAAGATAAGATAGAAGACTCCATATCTAGATACAAAATAGAACAAAGGAATCATAAACATAATGTAAAATTACATATCATATAAAGGAAATATTCTATTTAATATGCAAATAAAAAAATATTTCAAATAAAAAAAATCATAAAAAAAATAGTAAAACAATAAATAAATAGTATTAGTATAGTTATAGTATAGTAAGAGGTAAACTTTTCGAACTAACTGCTCGTTGATTTATTGTTTCATCGAGATCGAATGTAAACCACGATGTCATTTTCTTGTTCTTGAAGGGGCCTCTTTAATTCTTTTAGGTTTATGCTCTACTCCGGGTAAAAATCTGCTCGATTTTGATTTGCACATATAGGTCAAATGCGTCTAATACCGCTTCTTTTTGTTTTGCTAAGATTTCCCTTTTTTCATTCAGTTCATGCCTTTGCCAAGTTGGATATTCGACATATTGCATTCATCTATTCTATGCGAGTGATTAAGGTTCAGATGATTCCTTTATCTATTCCATTTAGAATTTGAAAATAAAAATAGGAATAATTTTTGATACAAGCAGTAATTATCGATATATTACCAATTGGGATTTGTTTAAACGGAGCCTGGATACTTCATGTCATTTTATTGGTTCAACCAAGCCAACCATAAATTATTCTCATTGATACTATTAGTCTGAATCCCCCTCCAAATGGATCTAGTTGTACTTCACGCTCCAAATTTTTGATGATTAAATCAATCTTTCTTGGGCGAAGGGAAGGATATCTCGATCGGGGAAGAGAACGGGGAAAGCCCATATGACCCAATATATCTGACAAGTCGCACTATACGTCAACCCAAGTTGCATCTTCGTCTCCCGGAATTCGAAAGGGTACTTTTGGAACACCAATAGGCATTAACTAACATAAAAAAGAATTAAGTACTATATTTCACTTTGATATGGAAACGTAATAATCGGGCTATTCTCTTCATAATATCAACCTTTATTATATATTATATCTAGAATACACATTAGAAAAGGTCATAAAAGACGATAGAATGACTAAAGGAAAATTCTTACGACTAGAGCCTTCCAAGGATGAACAAGTATGGCGGCGTTTGCTCATAGAAAAGGGTATCAACCCCCATTGCGTATTGGTACTTATCGGGTATAGAATAGATCTGCTTCTCTTTGTTCCTACAATCATAATTGTTCCATTATTACCAATAGAATAGAACAAATATTAACCCTTGCTCCTAGATAATCCACTGAACAGAACAGGGGTTCATTTATAGTCATAGTCTTTTCCAATGCAATAAAGTTACATAGTGTCTATTTTTATTTGATAAAGGGGTATTTCCATGGGTTTGCCTTGGTATCGTGTTCATACCGTCGTATTGAATGATCCTGGTCGGTTGATTTCTGTCCATATAATGCATACGGCTCTGGTTGCTGGTTGGGCCGGTTCGATGGCTCTATATGAATTAGCAGTTTTTGATCCCTCTGATCCCGTTCTTGATCCAATGTGGAGACAGGGTATGTTCGTTATACCCTTCATGACTCGTTTAGGAATAACTAATTCCTGGGGTGGTTGGAGTATTACAGGGGGGACGGTAACGGATCCCGGTATTTGGAGTTATGAAGGTGTGGCCGGGGCACATATTGTGTTTTCTGGCTTGTGCTTTTTGGCAGCTATTTGGCATTGGGTATATTGGGATCTAGAAATTTTTTGTGATGAACGTACAGGAAAACCTTCATTAGATTTGCCTAAGATTTTTGGAATTCATTTATTTCTTTCCGGGGTGGCTTGTTTTGGTTTTGGCGCATTTCATGTAACAGGCTTGTATGGTCCTGGAATATGGGTGTCTGATCCTTATGGACTAACTGGAAAAGTCCAGGCAGTAAATCCCGCATGGGGCGTGGAAGGTTTTGATCCTTTTGTTCCGGGGGGAATAGCCTCGCATCATATTGCAGCAGGGACATTGGGCATATTAGCGGGCTTATTCCAT

CTTAGTGTTCGCCCGCCCCAACGTCTATACAAAGGATTACGTATGGGTAATATTGAAACCGTCCTTTCCAGCAGTATCGCTGCTGTCTTTTTTGCAGCTTTTGTAGTTGCCGGAACTATGTGGTATGGTTCAGCAACTACTCCGATCGAATTATTTGGTCCCACTCGTTATCAATGGGATCAGGGATACTTTCAGCAAGAAATATATCGAAGAGTTAGTGCTGGGCTGGCCGAAAATCAAAGCTTATCAGAAGCTTGGTCGAAAATTCCTGAGAAATTAGCCTTTTATGATTACATTGGCAATAATCCGGCAAAGGGAGGATTATTCAGGGCAGGTTCAATGGACAATGGGGATGGAATAGCTGTTGGATGGTTAGGACACCCAATATTTAGAGATAAAGAAGGACGTGAGCTCTTTGTACGTCGTATGCCTACTTTTTTTGAAACATTTCCAGTCGTTTTGATAGACGGAGATGGAATTGTTAGAGCCGATGTTCCTTTTAGAAGAGCAGAATCGAAGTATAGCGTCGAACAAGTAGGTGTAACTGTTGAGTTCTATGGTGGCGAACTCAATGGAGTGAGTTATAGTGATCCTGCTACTGTGAAAAAATATGCTAGACGTGCTCAATTGGGTGAAATTTTTGAATTAGATCGTGCTACTTTGAAATCGGATGGTGTTTTTCGTAGTAGTCCAAGGGGTTGGTTTACTTTTGGACATGCTTCCTTTGCCCTGCTCTTCTTCTTCGGACATATTTGGCATGGGGCTAGAACCTTGTTCAGAGATGTTTTTGCTGGTATTGACCCAGATTTAGATGCTCAAGTAGAATTTGGAGCATTCCAAAAACTAGGAGATCCAACTACAAGAAGACAAGCAGTCTGATACAAAATTGCTTTTGTATTTTTCGTCTTTCTTTTTGTGATTTGATTTGACATTGGGGATCAGAGAAATCTTGATTTTCTCATTACCCTTTCGTTGACTCTTTCTTTATCAGGGAAATAATCCCCAATAAACAGGTATGGAAGCTATAATTGTAAACCACAATCGAATCTATGGAAGCATTGGTTTATACATTTCTATTAGTCTCGACTCTAGGGATAATTTTTTTCGCTATCTTTTTTCGAGAACCGCCTAAAGTTCCAACTAAGAAATGATTTTTCATTATCTCCGTTGAAGTAAAGAGCCTCCCAATATTGAATGAATATTGGGAGGCTCTTTACTTCAACTAGTCCCCGTGTTCCTCGAACGGATCTCTTAGTTGTTGAGAGGGTTGCCCAAAAGCGGTATATAAGGCGTACCCGGTAAAACTTACAAGTAAACCAGATATAAAGATGGCGACTAGGGTTGCTGTTTCCATTCTTATATAAATTCAAGACCGCAATGGATCTCTGATAAGATCCTTTATTTACAAGGGAATGGTATACAAAGTCAACAGATCTCAATGAATACAATCGGATTTATGGCTACACAAACCGTTGAGAGTAGTTCTAGGTCTCGTCCAAGACAAACTACGGTAGGGGCTTTATTGAAACCATTGAATTCGGAATATGGTAAGGTAGCTCCTGGATGGGGAACTACTCCTTTGATGGGTGTCGCAATGGCTTTATTTGCAGTATTCCTATCTATTATTTTGGAGATTTATAATTCTTCCGTTTTACTGGATGGAATTTCAATGAATTAAATCCACAAGAACTACTAAGTTCGAGCTTTTCAATACAAAGTAAAGTTAAATTTCAGGTTTCCGATTTATAACCCCGTTGGTAGTTCGATCGCGGAATTTATTTCTTTCTGTATTTCCGGAATATGAGTGTGTGACTTGTTATAATTGATCCTATTGATAATACAGAGAATGAGTCTGTCATCTTATCTTGATAGAGATGGTTCTACCTCGTCGGATACTCATCCTAGTATCTGGAACACGAGATATTATGAAATAGATCAATAAATATTTGAACTATGATTCATACTTAATATTCAGACCTCGTGACCGGATTCTAAAAAATTTTCAACGAATTAGAAATAGTTATAAATTGAAAGATTTTTCTTTCTGTTTATGCTTATTTTGTTTTGACCAAAGACCAAAAGGTCAATTCTTTCGTATTTTGAGTCATTATACTATTGCTTGAATAAGTGATGATCCGATAGTTCTTACTCAGGGAATCTTTGGGCTTGAGGTTTTTATTGAATCATCGTGGTTCTAGTATGAATCTGGGGTTTCAATTTCTTTTTAATTTATAGGGTCTTAACAAGAGAAATTCCTATCAATGAGAAAAAACAATAGTCAAGATTACACACAACTAACAAATCAAAGAAAAAATAGGGAAAGAGAAGATTCAAGAGGCCTGTAGTAACAATAAGAAGAGCCGACTTGATATTTTGGCATTATCACCACAAAGAAGAACTTTCGTATTTTTAATGCTTCGTATCTTCACTTCCGAGAAGATTAAATCGAAGGAGTAAGATATTTCTATTCCATATGCGTTGGGAGCAGTATTTGTGTGTTTCTGCTTGAGCTGTACGAGATAAAATTCTCATATACGGTTCTCAGAGGGGGAGTCCCCCCGGTTTACCTATCTCAATAAAGTCTATGATTGGTTCGAAGAACGTCTCGAGATTCAGGCGATTGCAGATGATATAACTAGTAAATATGTTCCTCCTCATGTCAACATATTTTATTGTCTGGGCGGAATTACCCTTACTTGTTTTTTAGTACAAGTAGCTACGGGGTTTGCTATGACTTTTTACTATCGTCCAACTGTTACTGAGGCCTTTGCTTCTGTTCAATACATAATGACTGAAGCTAATTTTGGTTGGTTAATCCGATCAGTTCATCGGTGGTCGGCAAGCATGATGGTTCTAATGATGATACTGCACGTATTTCGTGTGTATCTCACCGGTGGATTTAAAAAACCTCGAGAATTGACTTGGGTTACAGGCGTAGTTCTGGCTGTATTGACCGCGTCTTTTGGTGTAACTGGTTATTCCTTACCTTGGGACCAAATAGGCTATTGGGCGGTCAAAATTGTAACAGGCGTACCTGAAGCGATTCCGATAATAGGATCGCCTTTGGTAGAGTTATTACGCGGAAGTGCTAGTGTGGGACAATCCACTTTGACTCGTTTTTATAGTTTACACACCTTTGTATTGCCTCTTCTTACTGCTGTATTTATGTTAATGCACTTCCTAATGATACGTAAACAGGGTATTTCTGGTCCCTTATAGAGAAGCTTATAGAGAAGATAGATCATAGATCTTTGTAATCAATCATTTATCACTTGGGGAAGGAACAATAGTATTTCATTGCTACAAATATGTCTTATTCATTTTAATAAGACATGTTTTTGGACATTCTCTTTCCTTCAACCCCACAATATTGGAATATTGTATTATGTTATTTAACATAACACGACTAGTTGAAGGGAATTCTCCGAAACGAAAATGGATTATGGGAGTGTGTGACTTGAACTATTGATTAGGCCGTGCAGATATATGCCCCTTTCTGCCACATTGAAATTCACAAACCAATGTGTCTTTGTTCCAACCACCGTATAAGCTCTCTACAGACGATAGGCTGGTTCGCTTGAATAGAATTCTTTCTATGATCAGCCCCGAATCATGTCATGCATGAACAGGCTCCGTAAGATCCAGTGGAATCAATGATTTGGCAGAATCCAGATTCCATTTAATCTATTTCATTTATTTAATTTAATGTTTTGTTTTAATAGTATGGAAATGCATTCATTTCCTCTGCATCGACCCGATCTATGATACTATCGGAGTGAAACAGGGCATCTAAAGAAGACTAGAGGCTATAATATGTTAGTTAGTAACAAGTAAACCCTTTGCTTTGTATGTAAAAAAAAGTCTCAAAATTTTTGGAGATAAACACCAATCGCAAGGTCTAAGACGACCCAGAAAGCATTTGAGCATGATTAACTTTGTAAGCCTACTTGGGGATTGAGCATTTATCTGGAAGAACGGAATTCCTTGTAATGGGTAGTTGCAACCTTGGAAAGGGGAATCTAGTCAAACTTTTCATATTTCATACAGAGAACCATTCATATATGTATGGATATAGACAACATAACATATATCTTAATATATGTTGGTCTTTTTTATGTTGGTTCTTTTAATTCTTGCTCGAGCCGGATGATGAAAAATTCTCATGTCCGGTTCCTTCGGGGGATGAGTCTATAAGAATTCACCTATCCTAATAACAAAAAAACCTGACTTGAATGATCCTGTATTAAGAGCTAAATTGGCTAAAGGGATGGGTCATAATTATTACGGGGAGCCTGCATGGCCAAATGACCTTTTATATATTTTTCCAGTCGTCATTCTAGGGACTATTGCGTGTAACGTAGGCTTAGCCGTTCTCGAACCGTCAATGATTGGCGAGCCGGCAGATCCATTTGCAACTCCTTTGGAAATATTACCTGAATGGTATTTTTTTCCCGTATTTCAAATACTTCGTACAGTACCCAATAAATTATTGGGTGTTCTTTTAATGGTTTCAGTACCTGCGGGATTATTAACAGTACCCTTTCTGGAGAATGTTAATAAATTCCAAAATCCATTTCGTCGTCCAGTAGCCACAACCGTCTTTTTGGTTGGCACCGTAGTGGCCCTTTGGTTAGGTATTGGAGCAACATTACCCATTGATAAATCTCTAACTTTAGGTCTTTTTTAAATTGATTCAATTGTAAAATAATACGATGTGTGTATCTAGGGAATAGTTGCTTCAAAGTGACTTTTCCCTAGATACATCTATTCAATTGAATTCAGGAACTATTCCGAATAATACGGGTTGCGCTAAAAAAAATTGATTATTGGTTTTGAATTCTAAAGAAAAAAAGGAAAGAAATCCAATGAATTTAAACCTTCTTTTTAGGTAAATCAATTGCGAAATGCTTTTCTAGAGTGCCCAATATTTGTTTTACATCTTCTATGCGAAAATGCTCAATTTTCATAAGATCTTCTTGACTGTTATTCAATAGGTCCAATAATGTATGTATATTGGAATTTTTGAGGCAATTGTAGATCCTGGGAGGCAATTCTAATTGATCAATAAAAATCGATTTCAATGCTATTTTTTTTTTGTTTTTTCTTAGTTCAGTCAATCTATCATGAAAGAAAAAAAGGGGTAAAGTAACCCTGTCTTGATTATCCTCGAAATGTAAGTTTTCTTCTTCCGCATATAGAAAGGGAATAAATAAATCAATTAAATTCCGGGAGGCTTCATAAAGTGCTTCTTTAGGAGTTAAACTGCCATTTGTCCATATTTCGAGAAAAAGTATCTCTTGTTTTTCATTACCATTACTATACGAATGAATACTATGATTCACATTTCGAACAGGCATGAATACAGCATCTATAGGATAACTTCCGTCTTGAAGGTTATTTGGCGTTTTTATACGAAATCCGCGATTCCTCTCGAGTTGTAATCCAATACGCAAATCAATTGGTTCCGTCAAGTTAGCTATATGCTGTGTAGTATCAACGATTTCCACATAAGGTGGTGAGATGATATCTTGAGCAGTTACACATCCAGGTCCCCTAACACAAATAGACGCGTCACAGGTTCCATATAGATTACTTCTCAATACAATTTCTTTCAAATTCATTAAAATTTCATGTACTGATTCTTGAATACCTACTATAGTAGAATATTCGTGTGGTATTTTCTCAGATTTTGCACGTGTAATACATGTTCCTTCTATTTCTCCAAGCAGAGCTCTGCGCATCGCAATGCCTATTGTGTCGGCTTGACCTTTCATAAGTGGAGATAGAATAAAGCGTCCATAATAAAGACGTTTACTATCTGTTCTTGATTCAACACACTTCCACTGCAGTGTCCGAGTAGATACTCTTATTTTCTCTCGAACCATAGTAATATTATAGATAATAGATCAGATCATTGAGTCATTTATTTCTCTTGAAATCCCTTCAATGCTTATTTTTACACACGTCTTTTTTTAGGGGGCCGACAGCCATTATGCGGCATGGGGGTTACATCTCGTACGAAACTTAATAGTATGCCACTTCTACGAATAGCTCGTAATGCTGCATCCCTTCCGAGACCAGGACCCTTTATCATGACTTCTGCTCGTTGCATGCCTTGATCTACTACTTGACGAATAGCGTTTCCTGCTGCGGTTTGGGCAGCAAATGGTGTCCCTCTTTTTGCCCCTTTGAACCCGCAAGTACCGGCGGAGGCCCAAGAAACCACTCGACCTCGTACATCTGTAACAGTCACAATGGTATTGTTGAAACCGGCTTGAACATAAATAACTCCTCTTGGTATTTTACGTGCACTCTTACGTAAATTAATACGCCTATTCCTACGTGAACCAATTCTTGGTATGGGTTTTGCCATATTTTTTCGTCTCATAAATACGAGTCAGAGATATATGGAGATATCCATTTCATGTCAAAACAAATCCTTTCATTTTTTTTTTCTACATCATTTGTACATTGAGTCCGTTAGAAAGTCCCTTTTTTTATTAGTAGACTGATTATCCTTGTCGTTGTTTATGTTTCGGGTTGGAACAAATTACTATAATTCGTCCCCGCCTACGAATTAGTCGACATTTTTCACAAATTTTACGAACGGAGGCCCTTATTTTCATATTTGTCATTCCTTACTTTAATTCCGAATCTATTTCTTGGAGGAAAATAAGTTTCTTGAAATTTTGAATCTCAAATTGTATTCCGGAATGTAGAAGTTGAAAAACCACTTAATCGGTTGAATCCTTGTTACGGAGTCTATAAATTATACGTCCTCTGGTTGAATCATAACGGCTTACTTCAATTTTAACTCTATCCCCCGGTAGGATTCGTATAAAACTACGGCGTATCCTTCCTGAAACATAACCTAGAATCAGATCCTCATTATCTAAACGAACCCGGAACATACCGTTAGGAAGTGATTCAATAATTAAACCTTCATGAATCGATTTTTCTTCTTTCATTCCAGGCAAAACCCCCTTAAAGTATCAACTAATGGAGGAGGAGTGATATTAGACAACCCGTCCTTTCTCTTTTTTTCCAAAATAGGAAATTTCGGATCCAATTCGTATATCAGAGGGATTACCATATATAACATAAAATTTCTCCGCCAATTCTTTCTAGTCGAGCCTCTCGGTCTGTCATTATACCTCGAGAAGTAGAAAGAATTAGAATCCCCATTCCACCTAAAATTCTAGGAAGTCGTTGATAGTTAGAATAGATTCGTAGACCAGGGCGACTGACCTGCTTTAAATTTAAAAATTTTGTATACGGCCCTTTCCTATTCCTTCTATGTCGTAGAGTTGAAACCAAAAAATATTTGTTTTTTTCCTGATGTTTCCTCACGTTTTCGATAAAACCTTCTCGTAAAAGTATTTTAACAAGGGTTTCCGTGATTTTAGTAGATGTTATTCGAACCGTTCCTTTTTTATTCATGTCAGCATTTCGTATCGAGGTTAGTATATCAGCAATGGTGTCCCTATTCATGATGACCTCAAATTAGTGGTGCTCCGAATTTTGATATAATCAACGTGCTTTTATTAGTTTCTTTTTCTTATTCTTTTTTTTTTATTATTATTTATTTTTATATTTCTGTAATGTAAAAAGGAAAGGTATATACGTGATACACAATCTACTACTAAATCGATTTCGTTCAAATAGCCTACTATTCTCGTGGTTTATAATACCTCAGGGATTCTCGTGATTTATAATACCTCGGGAGCTAATGAAACGATTTTAGTAAAGTTTAACTGTCTCAATTCTCGGGCGATTGCACCAAAAACTCGAGTTCCTTTTGGATTTCCTTTTTGATCAATAATAACTGCAGCATTGTCATCATATCGTATTATCATACCGTTGTCGCGTTTGAGTTCTTTGCGAGTACGTACAATTACAGCTCGGATCACTTCTGATCTTTCTAAGGGCATATTTGGTATTGCTTCTTTTATCACAGCAACAATAATGTCACCAATATGAGCATATCGACGATTGCTAGCTCCTATGATTCGAATACACATCAATTCTCGAGCCCCGCTGTTGTCTGCTACATTCAAATGGGTCTGAGGTTGAATCATATCATTTTTGAATCTTTCAATGCAAAGGCGAAAAAAGAAAAAGAAATATTATTTGTCCAAAACTTTGTCCAAAACAAAAAACTGGCGGTTGTTTTTTTATCCCAACGTTTGTTTCTACATTCCTACATTCCTATCCTGAAATAAGAAATTGAGTTCTTATAGGCATTTTGGATGCCGCTAGTGATATAGCCTTTCTGGCTATTTTTTCTGTTACTCCGCTTATTTCATAAAGTATTCGACCTGGTTTGACAACAGCCACCCAATATTCGGGGGATCCTTTCCCCGAACCCATACGTGTTTCCGCAGGTCTTACTGTAACGGGTTTGTCTGGAAATATACGTACCCATAGTTTTCCACCACGACGCACATTTCGTGTCATTGCCCGCCGACCTGCTTCTATTTGTCTAGATGTGATCCAAGCGGGTTCAAGTGCCTGAAGAGCATATCTGCCGAAACAAATACGATTACCTCGATAAGATATTCCCTTCATTCTTCCTCTATGTTGTTTACGGAATCGAGTTCTTTTGGGGTTATAGTGGATGGTTCTTTTTCAATTCCATCTCTATTACAGAACCGGACATGAGAATTTCTTCTCATCCGGCTCCTCGCGAATGAAATGATCCAAAAGTATATATTTTTGAATAAAAAAAATGAAATAGATTTATATTTATTATTTATAAATATATTTAATAATAAGAATATTAGTATATAAATAATAAGAATATCAGTATATAAATAATAAGAATATTAATATATAAATATATAAATAATAAGAATATAAATATATAAATAATAAGAATATAAATATATAAATAATAAGAATATTAATATATAAATAATAAGAATATTAATATAATTAATATAATATAAATATAATTAATATAATATAAAGAAATATAATATAAATAGAAATAAATAAAATATAAATATAAAAAAAAGAAAAATATTTAATATTTCTAATATTTCTTTAATTTTTATTTAAAATTTAATGAAAATTATTTGATTTATTTTTTTAATTTGAATAAATAAATCAAATTAATATATTGATTTAGATTGTTATCAGTATAAAATATAAAAAGAAGTGAAATCATTTTTGTTTTGAATTTAAAAACTTTATATATATTTAAATATTTACTATTTCATTTTATTAAATTCCATTTTTTATTTAATATTTAATAATAGTTATAACAAATCTTTTTTTTGTTTTCGCTTTTATCGTATCAGATCACCTACATTTTAGCAATTCAATAAAAAAAATGTCGTGGGCGAATATTTACTCTTTCAATATCTATTTCTGTTGTAGGGTTAGTTCATGACTTCTCAGAATAGATGAATTGGTCTCTGGTTTATTCCGCCATCCCGCCCGCTGAATCATGTGTATTCATTTTCAATTGAATCTTCTGTATTCACAGGTTCCATCGTTCCCACCGCTTCTTGATTAATGGTTAGGCCTGAATTTGACAACGGAGCTTTTACTTAAATTTGTTCTTGAGTCAACGTTCTTAGTCTTTATTGGCTCGAGGCTCTTAATTTTGGTGCTATGAAAAGATTCATATAATGATAGATGAATCCGTATTGATGCTTTATTACACTGCCTTTTATGAGATGATTCATAGACCTTACATATTGGAATTCTATATCATTGATAGATTTTTATATCTTTCTCTCACCTTCCATTTATCCACATCCTTTCGCTTACAACTCATAATCGGATTGCTTTTTCTTTTGTTTATGCCAAAAAGAGTTCAGTTGCTGCAATGATACGACCAATATATCATATCTTGACTGCTTCCTTGGATCCAGATAATTTGAAGTGATGAGTTAGTTATTAGTTCTATAGTTATTAGTTCATATTATGGGTTGTTAATTTTTTATCTTAATCCTAACAAAAACCAACGAGTCACACACTAAGCATAGCAATTCGTTCAATTCAAAAGGGGTTCAATCGAATTTTTATTCAACCTTATAGAATTAGAATTGATCATTTTTCTTTTTTGTTCTGTCATTGAATAGAAGGGAAAGACAAGTAAAGGCTTATTATTTTATTCCTCGTCTATAAATATCCAAATTTTGATACCTAAAACCCCATATATAGTTCGAACTGTATAGGCGCAATAATCAATTTTGGCGTGAATGGTTTGTAGGGGAACTCTACCTTCTCTGATCCATTCGATACGTGCAATTTCTTTTCCGTCGATACGTCCTGCAATTTGTATTTGAATTCCTTTTGTATCAGCCTGTTCGGTTAGTTCAATAGCTTTTTTCATCGCTTTTCGAAAAGAGACCCTATTTTTTAATTGTCCGGCTATAAATTCTGCAAGAATATTAGGGTGTCTATAAGGTTTTGCAATTCTTGTAATAGCAATGTTGAGTTTTCGGTTCACAGAATTTAATTCTTTTTGTACATTCATCTGTAGTTCTTCGATGCCTCGTGGTCTATTTTCTATTAATAACTTGGGGAATCCCATATAGATTATGACCTGGATCAGATCAATTCTTTTTTGAATTTCTATACGTGCAATTCCCTCGACACCGGAGGATGTTCTCATATTTTGTTGAGCATAATTCTTGATACAATCCCGTATTTTTTGATCTTCTTGTAAACCCTCAGAATAATTTTTTGGTTGTGCAAACCAAATGGAATAATGACTTTGGCTTGTACCAAGTCTGAAACCAAGTGGATTTATTTTTTGTCCCATATTGCCCCGCTATATTTTAAACGGAATTTGCTAGGTAAATACTTTTCTCGATTCTCTAATATTTCCTCTAATCTCGATTCTCTAATATTTCCCCAAATGTTTGAGGTATTTCATAGTAGGATATATCTTTCAATACAATAGTTATATGACAAGTGGGTCTTTTTATCAGATAACTACGCCCTCGAGCTCGGGGTTTTAATTTTTTCCTGGTAGTTCCTTTGTTGACTTCCGCTTTCCAAATGATTAAATCTTCTTTTTCGAAACCCTTATTGTGTTTAGCGTTTGCTGCTGCGGAATAAATCAATTTAAAAATGGGATAACATGCTCGATAAGGCATGAGTTCGAGTATCATCAGTGTTTGTTTATAGGAACGCCCGCGGATCTGATCAATTACTCTTCGTGCTTTGTGAACAGACATAGGTATATATTGGCCTAGAGCAGATACTTCAGTCAGCGACTTTCTTTTTTTTCTCATAAGTTTTACCTCTGCATTAATGAAGGATAAGCATCTCTATTTATTAATTCTTAACGACGAGATCTATTATCGTTTTTTGCATGTCCCCGGAAATTTAGAGTAGGTGCAAATTCTCCCAATTTGTGGCCTACCATACGATCTGTTATATAAACAGGCAAATGTTCTCTTCCATTATGGATAGCAATAGTATGGCCAATCATTGTGGGGATAATGGTAGATGCCCGGGACCAAGTTACTATTATTTCTTTTTCCTCCTTTGTGTTAAGCTTATCAATTTTTCTTAATAAATGATTCGCTACAAAAGGATTTTTTTTTAGTGAACGTGTCACGGTTAATTACTCCTATTTTTTTTATTTAAAGACGAAGAAACTAATTCAAATTTCTCTCCTATTTACTACGTCGACGAATAATCAAATTATCACTATATTTATTCCTTTTTCTACTTCTTCTTCCAAGTGCAGGAAAGCCCCATTTATTTGTTGGGCTTTTTCTACCAATTGGGGCCCTCCCTTCACCACCCCCATGGGGATGGTCTACAGGGTTCATAACGACTCCTCTTACTACAGGACGTTTACCTAGCCAACGCTTAGATCCGGCTCTACCCAAACTTTTCTGGTTCACTCCAACATTCCCCACTTGTCCGACTGTTGCTGAGCAGTTTTTGGATATCAAACGGACCTCCCCAGAAGGTAATTTTAATGTGGCCGATTTCCCCTCTTTTGCAATCAGTTTCGCTACAGCACCCGCTGCTCTAGCTAATTGTCCACCCTTTCCAAGTGTGATTTCTATGTTATGTATGGCCGTGCCTAAAGGCATATCGGTTGAAGTAGATTCTTCTTTTTGATCAATCAAAACCCCTTCCCAAACTGTACAAGCTTCTTCCAAAGCATACGGCTTTCTGGATGTAGATGATGATATCTATACAGATGGATCTTCTATTTATCATACAATGAAGTACCACATGAGCAGATATATAGGAATCCCAATCTGCCGAATCACTCATGTTATGATCTTCTACATCCTAGGTCTTCCCGTTCCGTCATCTGGCTTATGTTCTTCATGTAGCATTCAGACCGAATGACTCTATGAAATTACGTCGATACTTCCACATATACATATTATGGGTAACGTAGGAGACATCTCTATTTTTCCCCGGGGAATCTTTAGACACTGCTTAGCTTTCAATTCGCCTCTGACCATCAAATGAAATGTGAATAACCCGTCCTCCTCTCTTTGAAAGAAGGGGCGCTTCCGGTTCTGTCGGTGCTTGAAACAATTTTGTCTTCTCCATATTACTATATCTCTAGAGTCAATAATTTTATATGAGGAACTACTGAACTCAATCACTTGCTGCCGTTACTCTTCAGTTTTCTGTTGAGGTCTATCCTCTAGAGGTACTCAAATTGGATCCGTGATCGATTTCTAGGTTTCGTCGTAAACCTAATTGGTTACTTCCAATTACGTAAATCCATAGTTCAAACCGCACTCAAAGGTAGGGCATTTCCCATTTTTATAGGAACTTCTGTACCAGAAACAATGGTATCTCCAATTATAGCCCCTCTGGGATGTAAAATATATCTCTTCTCACCATCCCCATAGTGTATGAGACAAATGTATGCATTTCGATTAGGGTCGTATTCTATGGTTACGATTCTACCATATATGTCTTTTTCATTCCGTCGAAAATCGATTTTACGGTATAGACGCTTATGACCTCCCCCTCTATGCCTTGCGGTAATGATTCCTCTGGCATTACGACCTTTACCACAACGACGCTGTCCATAGATCAAATTATTTCGTGGATTGGATTTCACTTGACTGTCTACGGCCCTATTGCCTGTGTTCGGGGTAGAAGTTTTGTATAAATGTATCGCCATGCTATTAAGTATTTTTATTTAAGTTCTTTTCTTTCTAAGAGGTGGAATAGAATAACCCGGTTGAAGCGTAATGATCATACGTCTGTAATGCATTGTATGTCCCATAATAGGTCCCATTCTTCTACCCTTTCCCGGGAGTCGATGACTATTCATAGCTATTACCTTGACACCAAAGAAGAGTTCGACCCAATGCTTTATTTCTGTCCTAGTTGATCCTGATTCGACATTAGAAGTATATTGATTTTTCCCCAATAACCGAATACTTTTGTCGGTAAATATTGCATATGTGATTCTATCTATTTTCTTCATATTCAAAATGGCATATATTGCATATGTGATTCTATCTATTTTCTTCCCTATGAGTTCGAGTCTCAATAAGAATGCTAGTTCTTACTGTTCCTATGTTATGAAATGAATATACCAATTCGTTATGTATGGAGGATGAGATTCCATTGATACAGAGCCAATTCCAATAGACTTATACTTATTGGAGGGTCCCATTGGCGTGCATCCAGTAGGAATTGAACCTACGAATTCGCCAATTATGAGTTGGGCGCTTTAACCATTCAGCCATGGATGCTTAGCGGGGATCCTCGTACATGGTGAATAACCAAATTCCAATTGAAATGAAATCTTTAGGATAAATCAATACAAATCCAATTTTCATTTTATACAAATATCATTGAAATTTTCATTGTTGACATTTTCATTGAAATAGAATATAGAAATGTTAGACATATTTTCATTTTCAGTGATTCAATTTTCAGTGAAATATCATAATTGGAGTATCATAATCATATTATATTATAGATATACAATTACGTTTTCGTATTGTATTATACTATAGTATAAGTATCAAATTGAATTTTTTTTTTTAACTTTAACAATTGCATATTAAAGTAAATTTATAGTATTTTAAAATTCAATTTTTTTTTTAACAAATGACAAAAAAACATTCCACAAATTTAGATTTCTGGGTTTTCGAGTTGAAAGAGATATTGAGAGAGATCAAGAATTCAGTGGGATCTTTGGTTAAGATTTTTTTCCACCAAGAACGTTTTATAAAACTCTTTGATCCCCGAATTTGGAGTATCCTACTTTCACCCAATTCGCGGGGTTCAATAATTAAGCGATATTTCACTTTCACGATCACGGGTGTAGTATTCTTTGTAATCAAGGGTGTAGTATTCTTTGTAGTAGCGGGCCTTATATATCGTATTAACAATCGAAATATGGTCGAAAAAAAAAATATCTATTTGATAGGGCTTCTTCCTATACCTATGAATTCTCTTGGGTCCAGAAATGATAGATTGGAAGAATCCTTTGGGTCTTCCAATATCAATAGGCTGATTGTTTCGCTCCTCTATCTTCCAAAAGGAAAAAAGATCTCTGAGAGCTGTTTCCTGGATCCGAAAGAGAGTACTTGGGTTCCTCCAATAACTAAAAGGTGTAAATCTAACTGGGGTTCGCGGTGGTGGAGGAACTGGATCGTAAAAAAGAGGGATTCTAGCCAATTGAAAGGATCTTTTGATCAATCTAGAGATCGCTTGGATTCCATCAGGAATGCGGATTCGGAATATCACACATCTCTCAATCAAAGGGAGATTCAACAGCGAAAAGAAAGATCGATTCCTTGGGATCCTTCCTTTCGTCAAACGGAAGAAACAGAGATAGGATCAGGCCGATTCCCGAAATGCCTTTCTGGATATTCCTCAATGTCTCGGCTATTCACGGAAGGTGAGAAGCAGATGAATAATCATCTGCTTCCGGAAGAAATCGAAGAACTTCTTGGGAATCCTACAAGATCCATTCGTTCTTTTTTCTCTGACAGATGGTCAGAACTTCATCTGGGTTCAAATCCTACTGAGAGGTCCACTAGAGATCAGAAATTGTTGAAGAAAGAACAAGATGTTTCTTTTGTCCCCTGCAGGCGATCGGAAAATAAAGAAATGGTTAATATATTCAAGATAATTACGTATTTACAAAAGACCGTCTCAATTCATCCTATTTCATCAGATCGGGGATGTGATATGGTTCCGAAGGATGAACCGAATATGGACAGTTCCAATAAGATTTCATTCTTGAACAAAAATCCATTTTTTGATTTATTTCATCTATTCCATGACCGGAACAGGGGGGGATACACGTTACACCACGATTTTGAATCCGAAGAGAGATTTCAAGAAATGGCAGATCTATTCACTCTATCAATAACCGAGCCGGATCTGGTGTATCATAAGGGATTTGCCTTTTCTATTGATTCCTACGGATTGGATCAAAAACAATTCTTGAATGAGGTATTCAACTCCAGGGATGAATCGAAAAAGAAATCTTTATTGGTTCTACCTCCTATTTTTTATGAAGAGAATGAATCTTTTTATCGAAGGATCAGAAAAAAATGGCTTCGGATCTCCTGCGGGAATTATTTGAAAGATACAAAAGAAAAAATAGTGGTATTTGCTAGCAACAACATAATGGAGGCAGTCAATCAATATAGATTGATCCGAAATCTGATTCAAATCCAATATAGCACTTATGGGTACATAAGAAATGTATTGAATCGATTCTTTTTAATAAATAGATCCGATCGCAACTTCGAATATGGAATTCAAAGGGATCAAATAGGAAACGATACTCTGAATCATAGAACTATAATGAAATATACGATCAACCAACATTTATCGAATTTGAAAAAGAATCAGAAGAAATGGTTCGATTCTCTTATTTTGATTTCTCGAAGCGAGAGATCCATGAATCGGGATCCTGATGCATATAGATACAAATGGTTCAACGGGAGCAAGAATTTCCAGGAACATTTCGTTTCTGAGCAGAAAAGCCGTTTTCAAGTTCAAGTAGTCTTCGATCGATTACGTATTAATCAATATTGGATTGATTGGTCTAAGGTTATCAACAAAAAAAAATTTTCTAAGTCATTGTCAAAGTTGATTCTCTTTTTGTCTAACTCACTTCCTTTTTTCTTTGTGAGTTTAGGGAATATGCCCATTCATAGGTCCGAGATCCACATTTATGAATTGAAAGGTCCGAATGATCAACTCTGCAATCCGTTGTTAAAATCACCAGGTCTTCCAATCGTTCATTTGAAAAAATGGAAAGCGGATGATCATGATACTTCCCAAAAATCTAAATTATTGATCAATGGAGGAACAATATCACCCTTTTTGTTCAATAAGATACCAAAGTGGAAGTGGATGATTGACTCCCATACTAGAAAGAATCGCAGGAAATCCTTTGATAACACGGATTCCTATTTCTCAATGATATCCTGCGATCAAGACAATTGGCTGAATCCCGTAAAAGCATTTCATAGAAGTTCATTGATATCTTCTTTTTATAAAGCAAATCGACTTCGATTCTTGAATAATCCACATCACTTCTTCTTCTATTGTAAGAAAAGATTCCCTTTTTATATGGAAAAGGCCCGTATCAAGAATTATGATTTTACGTATAGACAATTCCTCAATATCTTGTTCATTCGCAACAAAAAATTTTCTTTGTGCGTCGGTAAAAAAAAACATGCTTTTTTGGAGAGAGATACTATTTCACCAATCGAGTCACAGGTATCTAACATATTCATACCTAACGATTTTCCACAAAGGGGTAACGAAGGGTATAACTTGTACAAATCTTTCCATTTTCCAATTCGATCCGATCTATTCGTTCGTAGAACTATTTACTCGATCGCAGACATTTCTGGAACACCTCTAACAGAGGAAGAAATAGTCAATTTGGAAAGAACTTATTGTCAACCTCTTTCAGATATGAATCTATCTGATTCAGAAAGGAAGAACTTGCATCAGTATCTCAATTTCAATTCAAACATGGGTTTGATTCACACTCCACGTTCTGAGAAATATTTACCATCCGAAACGAGTAAAAAATTGCGTCTTTGGCGAAATTGGCTAAAGAAAGGCGTTGAGAAAGGGCAGATGGATAGAACCTTTCAACGAGATAGTGCTTTTTCAACTCTCTCAAAATGGAATCTATTCCAAACATATATGCCATGGTTCTTTACTTCGACAGGGTACAAATATCTAAATTTGCTATTTTTAGATGCTTTTTCAGACCTATTGCCGATGCTAAGTAGCAGTCACAAATTTGTATCCATTTTTCATTATATTATGCACAGATCAGCATGGCGAATTCTTAAGCTAAAATGGCGAGCTCTTAAGCTAAAATTGTGGGGATTGTGGGCACCGATAAGTGAGATTTCAAGTGATATTTCGTGGAAGTGTTTCCGTAGGCTTCTTCGGGTCGAAGAAATGATTCATCGAAATAATGAGTCACCATTGATATCGACACATCTGAGCTCGCCAAATGTTCGGGAGTTCCTCTATTCAAGCCTTTTACTTCTTCTTCTTGCTGGATGTCTCGTTCAGGTACTTCTTTTCTCTGTTTCCCTAGACTCTAGTGAGTTACAGACAGAGTTCGAGAGGATAAAATCTTTGACGATTCCATCATACACGATTGAGGTGTACAAACTTGTGGATGGGTATCCTAAACCTGAACCGAATTCTTTCTGGTTAAAGAATCTCTTTCTAGTTGCTCGGGAACAATTAGAAGATTTTCTAGCAGAAATACTGGGTTTTGCGCTATTTGGTGGTGGTCCCGCTTATGGGGTCAAATTTATACAGAAGATATTTTTCAATCTCATCGATCTCATAAGTATCATACCAAATCCCATCAATCGAATCACTTTTTCGAGAAATACGAGACATCTAAGTCATACAAGTAAAGAGATCTATTCATGGATAAGAAAAGGACAAAGGTTTCAGACTCATGATGAAATAGAATCCTGGATCGAGACCTGTGATTGGTTTTTGGATGAAGAGAGAGTTTACTCGTTTCATTTCTCCACCTTAAGGCCAGAAAAAGGGATTGATCAAATTCTATTGAGTCTGACTCATATTGATCATTTATTAAAGAGTGACTATGGTTATCAAATGTTTGAACAAGCGGGAGCAATTTACTTACGATACTTAGTTGACATTCATCAAAAGGATCTAATGAATTATGAGTTCAATACATCCTGTTTAGCAGAAAGACGGATATTCCTTGCTCATTATCAGACAATCACTTATTCACAAACCTCGTGTGGGGCTAATAGTTTTCATTTCCCATCTCATGGAAAACCAAAACCCTTTTCGTTCCGCCTAGCACTATCCCCCTCTAGGGGTATTTTAGTGATAGGTCCTATAGGAACTGGACGATCCTATTTGGTCAAATCCCTAGCGACAAACTCCTATCTTCCTTTCATTACGGTATTTCTGAACAAGCTGGATTTGAAAATAGTTATTGATGATCTCGATCCTGAGGACTATATGGAAGCGCTTGATGATGTGGATATTGATGGTATTGATGATAGCGATCCTGCTAAGGAATATATGGATGCGCTGAAAGATGTGGACGATATTGATGATCGTGACTATATTTATTCGAACTTGGACTCGGACCCGGAGCTGAGGGAGGAGTATACGGTGGATGAGATACTTAGGTATATCATCGAGTTGGAAATAGACCTAGCTTCTATCAACTTGCAATTCGAATTGGCAAGAACAATGTCTCCTTGCATAGTATGGATTCCAAACATTCATGATCTGTATGTGGATGAGTCGGAGTCCCTCGGTTTATTATTGAACTATCTCTCCGGGGATTGTGAAAGATGGTCCACTAGAGATATTCTTGTTATTGCTTCGACTCATATTCCCCAAAAAGTGGATCCCGCTCTAATAGCTCCGAATAAATTAAATACATGCATTAAGATACGAAGGCTTCTTATTCCACAACAACGAAAGCACGTTTTCACTCTTTCATATACTAGGGGATTTCACTTGGAAAAGAAAATGTTCCATACTAATGGATTCGGGTCCATAGCCATGGGTTACAATGCACGAGATCTTGTAGCAATTACCAATGAGGCCCTATCGATTAGTATTACACAGAAGAAATCAATTATAGACACTAATACAATTAGATTCGCTCTTCATAGACAAACTTGGGAGTTGCGAGCCCATGTAAGACCGGTTCCGGATCATGGGATCCTTTTCTATCAGATAGGAAGGGCTGTTGCACAAAATGTACTTAGAAATAATTGCTGCCTTATAGATCCTATATCTATCTATATGAAGAAGCAATCATGTTACGAAGGGGATCCTTATTTGTACAAATGGTTCTTCGAACTTGGAACGAGCATGAAGAAATTAACGATACTTCTTTATCTTTTGAGTTGTTCTGCCGGATCGGTCGCTCAAGATCTTTGGTCTCTACCCGGACCCGATGAAAAAAATTGGATCACTTCTTATAGACTCGTTGAGACTGATTCTGATCTAGTTGATGGCCTATTAGAAGTAGTAGAAGGCGCTCTGGTGGGATCCTCGCTTCTTCGGCCCGAACCAAGGAATCCCTTAGAGATGATGGAAAATGGATCTCGTTCTATCTTTGATCGTAGATTTCTCTATGAATCGGAGTTTAAAGAATGGGCAGAAGGCACCGACCCGCAACAGTTAGCGGAGGATGTAGTCGATCACATAGTTTGGGCTCCTAGAATATGGCAACCTTGGGGCTTTCTATTTGATTGGATCGAAAGGCCCAATGAATTGGAATTTCCCTATTGGGCCAGGTCATTTCGGGGCAAGCCGATCATTTCTGATGAAGTTAATGATGAATATTTTGATTATGCATTTTATGGTGAAGGGGATGATGGATATGATGAAGAGGATGAGCTTCAAGAGAATGATTGGGAGTTCTTGCAGAGTGAAACCATGGAGTACCCGGGACGAGATAGATCTTCCAAAGAACAAGTCTTTTTTCGAAAAGGCCAATTCATTTGGGACCCTGGAGATCCACTCTTTTACATATTCAACGATGAGCTCTCTGTCTTTCTGTTTTCACATCGAGAATTCTTTGCAGATGAAGAGATGTCAAAGGGGCTTCTTCTGACTTCCCAAAGGGAGACTCTATATAAACGCGGGTTTAGCAAGAAACCGAAAGAAAAGTACTTCGAATTTTTTATTAATCGCCAGAGACGGAGACGGCTTAGAACCATTAGTTCATTATATAATAGATCTTTCCGTTCTAATATTCAATCCGCGAGTTATCAGTACTTATCAAATCTGTTCCTATCTAACGGAAGGCTATTGGATCAAATGACAAAGACATTGTTTAGAAAAAGATGGATTTTCCCGGATGAACTGAAAATTGGATTCATGTAACAGGAGAAAGATTTCCCATTCCTTAGCCGTAAAGATATGTGGCCATGAAAGAGGGATTAAGTGGAACAGAATTGACTGGGCGGTAGAGTCGTGGAAATACTTGTTTTTTCCATATTTCGGACCTTAGCTCCATGGAACAATATGCTACTGCTGAAAGATGGAAGAATTGAAATCTTAGATCAAAACACTATGTATGGATGGTATGAACGGCCTAAACAAGAATTCTTGAACAGCGAACAACCAGAGCCTATTACTCACTACATAAAAAAATTTCCATTAATGAAAGATGGAAATCCATTGTAAAATCAAAAATACGCATGTCTGATGAAAGTTGCTATCTGCTCCAATAACGAATCATTGGTTTGACTGAATAACTAAATAAAATACCCTATAGGGATAATACACATTCCAGTTGACCGAATTGTTTTGTTCCGAAGCAAAGGTATCCACGGGGTCGTTCGTCCTATTCATTCAGATATTCACGACCAAGAAGTACTGGATTCTCTTTCGGATAGGCCCCGAAAGGAGAAGGGAGGCTGGAATGCCAACAGGCGTCTATTCTTGAATTCACCCGACCCGATAGTACCCATTTTGGGGGGGAACGTCCAGTGCCAAAGTCACTAATGGGTAAGTCGCCAATCCCTAAAACGGACTATGTAATTTCTCTGCTGAGTTACGTTACGGGCGGGCATTTTACCAGAGGTTTCTATTGTATCAATCTACCCTTGTGTGATTCCTGTTGAAGCATATACTCGGGGGGGTTCAGGGCGGACGATTTCAAAGCGGACTCCCCCCTCATTAGATAGAGAAGATCACCAAGATTTCGTGATCCGCTGCCGAACTTATTCCAATTCCAAGATCTCTTATTGAATTGCTCATTCAATGAGCATTCTCTATGCCTTGAAGAGGACTCGAACCTCCACGCTCTTTAGCACGAGATTTTGAGTCTCGCGTGTCTACCATTTCACCACCAAGGCATCTTGAAAGTGATTCGTATTCCATGAATATGATATCTATCTAGTGTGATGTATGGAATATATGACAAAGGTGGAGTGTTGGAGTATTGCTATTGATCGGTCATGTCATATAGGCCCGAGTCGGACATCCAATTGCTTCGATTTTCATTTTCCGGAGGATGCCTTATACTTATATATATCAAAAAGATGGACAATCAAACCTATTTCTCGATTCAATAGAAGCCCAAAGAGATGAATAGGGTCCCAAATCAAATAACGAGAGATATGTAAAAAGAAGGTCCGATTACGCCTATTCCTAATCCGAAATGGAATGTAAAGACGTAGGGATCCATATGGAAATAGAGTATCTATTTAGATAGGCTCGAATGACCCCTTCTCATAATGAGAATTTATATAACCCTCTTCCGGCCTAGTCCGGTATGGAATGAACTTATAATCATGGAATCGACTCGATCATCAGATTATAGATTATAAGTTCATAACCCTAGCCCATTCCCATTTTGGGCGGAACAGATCTACTAATTCTTTGATTCCAGTTAGTAAGAGGGATGAAATAGACTCTAGAAGCTAAAAAAGGGTATCCTGAGCAATTGCAATAATAGGGTTCATTGATATTCCTGGTATAGTAGATGCTATCACACATACAATCATACTCAATTCGATGGAATTCTTTGATCTTAAAGGAGATCTTCTATAATTTCGCACGTGAGGGGTTATTTCTTGGTTTCGTCCAGTCATTAATAACTTGATTATTTTTAGATAATAGTAGATAGAAACAACGCTCGTAAGGAGTCCTATTGAAACCAAGAAATATAGGCCTGCCTGCCATCCACACCAGAATAAATGGAGTTTTCCGAAAAAACCTGCTAGTGGAGGAAGACCTCCTAGGGATAAGAGACATAGTGCTAAAGAGAGAGCCAAAGGGGGATCTTTCGTGTATAATCCTGCATAATCTCGAATGTTATCAGTTCCGGTACGTAGACCAAATAATACAATGCAAGCAAAAGTTCCTAGATTCATGGAGATATAGAACAGCATATAAGTTATCATGCTTGCATATCCATCATTTGAGTCTCCAACAATTATTCCAATAATTACATATCCGATTTGACCTATGGACGAATATGCAAGCATACGTTTCATGCTTGTTTGAGTAATAGCAATGAGATTCCCCACTATCATGCTAAGAATAGCTAGGATTTCCAGAAGAAGATGCCATTCGTTTGATGAGAAATAAAAAGGAATATCGAAAATTCGAGTGGCTGAAGCTGAAGCAGCTACTTTCGAAGTAACAGAAAGAAAAGCAACGACTGGAGTGGGAGAGTCAGAGTCGAAAAGAGGATTCCTCACTTCTTTCTCTCATTCAAAACCGTGCATGAGACTTTCATCTCGCACGGCTCCTAAGTGATAAAAGAAAGAAGAACTCATCTTCTTTCTTTTTTGATTACCTTCCTCGCGTATGTATAAGACCGAATCCGTTCGATTTCTAAAAAGGATTACTAATCCTTAACTTTTCGAGGAATCCTTCATCAGTGGTTGCGAATGACTTATTTTTTCAATCTTTTCGACCTTGGTTCCGTAGGAGCAAGTCAGAAAGATTGAGAAATAGAACCATCTGATTTGATTCGTTCTCAATAGCCATGAGATGATCATCTTAGGGTGATCCTTTTGTCGACGGATGCTCCTATTACACTCGTAGTCTCTGAAGGATGAGAACCAACTATGTAGCATCTACATCGAGAATTCAAGTATTGTATACGTCATTAGTCCGATCCTTTGTAGGAACTACCCGTAATAACGAACTTGCAAAATGGATCTGTTTATCATAAAGAGATTCGTTGTTCCTGACCCTGCTTCACCTTAATTGTTATTTGAACAAGTAAAAGTTATGTCTTGGTCCGAGTGGGGATAGCATTTCTCTTCTGCATGTCCATGGAGTTTTGAAAAATCCAAACATCTCGGAGATAGATAGAGAGCTAGGAATTTTTCGAACGAACCGCACTCCTTCGTATACGTCAGGAGTCCATTGATGAGAAGGGGCTGGGGAAAGCTTGAACCCAATTCCTACAGTGATGAATATAAGCGCAATTGAAATTCCTGGGGAGTTATACATTTGTGTATTGATAAGACCATTCACTATTTCTTGAAGCTCGATCTCTCCCCCGGATGAACCATATAGCCAAGAGAAACCATGAACCAGAATAGAGGAGCTTGCCCCACCCATGAGTAAATATTTCGTAGTAGCCTCATTAGACCGTACATCTTTCTTGGTATATCCAGATAATAGGTAGGAGCATAAACTGAAACATTCTGGAGCTACAAAGATAGTTATTAAATCGTTAGCACCGCATAAAAACATTCCTCCTAGAGTAGCTGTTAATACGAATAACAGAAACTCTGTTATAGCCATTTCTGTACATTCAATGTACTCTACGGATAGAGGAATACATAGAGTTGAACATAGTAAAATAAGAAATTGAAAGATTTCGTTGAAATTGTTCGTTTGGAAATTTCCCGAAAAGCTAATCATAGGTTCTTCTCTCCATCGGAACAACAGGGCCGTTATGCTCATTACTAAACTTGTTGAAGAGATGAAATAGAACCAAGGTATATCTTTTTGATCAGAGGTTGAATCGATCATCAGAAGAAGAATTAGGCCAAAAATTAGGATACATTCTGGGAAAATAAAACTTCCATCGAAGAGAAGCAAATGAAAGGCTTTCATAAAAATTCTCGTAGAATCGAGAATGAAGTTTTCATTCTGTACATGCCAGATCATGAATTAGTAACTGCATCCAATCTCCAAAAAAATCCCAATTGTTTAGAACTTTCTATTTTTGAAATGGAATATTTACGGAATCTCCACGAATAGGATCCAACCCTATTCCATGGTATTTCCATGAGATTCCTCTTGCTTATTTATTCTTAAGCAAGTCCCCGAGAGGGCTTAGTTGATCCATGATTTCTCTTTCATCTTTCGTTTCCTTTTCGTTTGTTTCGAAATAATCGATCAATTCCGATTCTTTCTTTTTCTATTGATTCTTTTCCGATCGAGATGTATGGATCCATGGATCTAGGCGTCTACTATATAGATCCTGTTCATGGATTAACGAAAATGTGCAAAAGCTCTATTTGCCTCTGCCATTCTATGAGTCTCTTCCTTTTTGCGTATGGCACCGCCACTCCCTTTGGCAGCATCCACTAATTCGGAACTTAATTTGAAAGCCATATTTCGACCCGGACGTTTTCGGGATTCCCCTAATAACCAACGAATGGCAAGTGCTTTTCCTTGTGTGGATCCTATTTCAATGGGAACTTGATGAGTCGATCCGCTTACACGTCTTGCTTTTACTGCTATATCGGGAGTTACTCCACGTATTGCTTGACGTAAAACAGATAGTGGATTTGTTTCTGTCTCTTGTTGAATCTTTTTCACGGCTCGATAGATAATTTGATAAGCCAATGATTTTTTTCCGTGTTTCAGAATACGGTTAACCAACATGTTAACTAATCGATTACGATAAATTGGATCGGATTTTGCAGTTTTTTCTTCTGCAGTACCTCGACGTGACATGAGCGTGAAAGGGGTTCAAGAATCAGTTTTCTTTTTATAAGGGCTAAAATCACTTATTTTGGCTTTTTGACCCCATATTGTAGGGTGGATCTCGAAAGATATGAAAGATCTCCCCCCAAACCGTACATACGACTTTCATCGAATACGGCTTTCCACAGAATTCTATATGTATCTATGAGATCGAGTATGGAATTCTGTTTACTCACTTTAAATTGAGTATCCGTTTCCCTCCTTTTCCTGCTAGGATTGGAAATCCTGTATTTTCCATATCCATACGATTGAGTCCTTGGGTTTCCGAAATAGTGTAAAAAGAAGTGCTTCGAATCATTGCTATTTGACTCAGACCTGTTCTAAAAAAGTCGAGGTATTTCGAATTGTTTGTTTACACGGACAAAGTCAGGGAAAACCTCTTAAATTATGGACCTTGGACATATAATAGTTCCGAATCGAATCTCTTTAGAAAGAAGATCTTTTGTCTCATGGTAGCCTGCTCCAGTCCCCTTACGAAACTTTCGTTATTGGGTTAGCCATACACTTCACATGTTTCTAGCGATTCACATGGCATCATCAAATGATACAAGTCTTGGATAAGAATCTACAACGCACTAGAACGCCCTTGTTGACGATCCTTTACTCCGACAGCATCTAGGGTTCCTCGAACAATGTGATATCTCACACCGGGTAAATCCTTAACCCTTCCCCCTCTTACTAAGACTACAGAATGTTCTTGTAAATTATGGCCAATACCGGGTATATAAGCAGTGATTTCAAATCCAGAGGTTAATCGTACTCTGGCAACTTTACGTAAGGCAGAGTTTGGTTTTTTGGGTGTGATAGTGGAAAAGTTGACAGATAAGTCACCCTTACTGCCACTCTACAGAACCGTACATGAGATTTTCACCTCATACGGCTCCTCGTTCAATTCTTTCGAAGTCATTGGATCCCTTCCCTCGTTCGAGAATCTCCTCCCTTCTTCCACTCCGTCCCGAAGAGTAACTAGGACCAATTCAGTCATGTTTTCATGTTCCAATTGAACACGTTCCACTTTTGATTATTCTCAAAGGAGAAGATTATTCTTTTTACCAAACATATGCGGATCCAATCACGATCTTCTAATAAGAACAAGAGATCTTTCTCGATCAATCCCCTTGCCCCTCATTCTTCGAGAATTAGAAAGATCCTTTTCAAGTTTGAATTTGTTTTCATTTGGAATCTGGGTTCTTCTACTTTTTACTTTTTTTCTATTTTTTCCCTCTCTTTTTTTATTCCCTTCCATAATTCCTTAAGTCTCATAGGTTTGATCCTGTAGAATCTGACCCATTTTCTCATTGAGCGAGGGATACGAAATAAATCAGATTGATTTTCATTTTTCGATCAAAAGTACTATGTGAAATCTTCGGCTTTTCCTCTTCCTCTATTCCTATCCCATAGGTACAGCGTTTAAATCAATAGAGAACTTTCTGTATGAATCGATATTATTACATTCCATCCAATTCCTTCCCGAAACCTCCCAAGGAAAATCCCGAATTGGATCCCAAATTGACGGGTTAGTGTGAGCTTATCCATGCGGTTATGCACTCTTCGAATAGGAATCCATTTTCTGAAAGATCCTGGCTTTCGTGCTTTGGTGGGTCTCCGAGATCCTTTCGATGACCTATGTTGTGTTGAAGGGATATCTATATGATCCGATCGATTGCGTAAAGCCCGCGGTAGCAACAGAACCGGGGAAAGTCTACAGAAAAGACAGTGCTTTTCTATTATATTAGTATTTTCTATTAGATTAGTATTGGTTAGTGATCCCGGCTCAGTGAGTCCTTTCTTCCGTGATGAACTGTTGGCACCAGTCCTACATTTTGTCTCTGTGGACCGAGGGGAAAGGGGGCTCGTCGGGAAGAGGATTGTACGATGAGAGAAGCAAGGAGGTCAACCCCTTTCAAATATACAACATGGATTCTGGCAATGCAACGTAGTTGGACTCTCATGTCGATCCGAATGAATCATCCTTTCCACGTAGGTAAATCTTTGCCTGCTAGTCAAGAGGATAGCAAGTTACAAATTCTGTCTCGGTAGGACATGTATTTCTATTACTATGAAATTCATAAATGAAGTAGTTAATGGTGGGGTTACCATTATCCATTTTGGAGTGACGAATCCTGTATGTGTTCCTAAGAAAAGGAATTTGTCCATTTTTCGGGGTCTCAAAGGGGCGTGTAAACACATAAGAACTCTGGAATGGAAATGGAAAAGAGATGGAACTCCAGTTCCTTTGGAAATGGTAAGATCTTTGGCGCAAGAAGAAGGGGTTGATCCGTATCATCTTGACTTGGTTCTGCTTCCTCTATTTTTTTTTAATACCGAGTCGCGTTCTTCTCCTACCTATATCGAATAGAACATGCTGAGCCAAATCTTCTTCATGTAAAACCCGCTTGATTTAGATCGGGAAAATCGTACGGTTTTATGAAACCATGTGCTATGGTTCGAATCCGTAGTCAATCCGATTTCCGATAGGAGCAGTTGACAATTGAATCCAAATTTTTCCATTCTTTTCGTATCCGTAATAGTGCGAAAAGAAGGCCCGGCTCCAAGTTGTTCAAGAATAGTAGAATAGTGGCGTTGAGTTTCTCGACCCCTTGCCTTAGGATTAGTCAGTTCTATTTCTCGATGGGGGCAGGGAAGGGATATAACTCAGCGGTAGAGTGTCACCTTGACGTGGTGGAAGTCATCAGTTCGAGCCTGATTATCCCTAAACCCAATGTGAGTTTTTCTATTTTGACTTGCTCCCCCGCCGTGATTGAAAGAGAATGGATAAGAGGCTCGTGGGATTGACGCGAGGGGCTAGGGATGGCTATATTTCTGGGAGCGAACTCCATGCGAATAGGAAGCGCATGGATACAAGTTATGCCTTGGAATGAAAGACAATTCCGAATCTGCTTTGTCTACGAACAAGGAAGCTATAAGTAATGCAACTATGAATCTCATGGAGAGTTCGATCCTGGCTCAGGATGAACGCTGGCGGCATGCTTAACACATGCAAGTCGGACGGGAAATGTTGTTTCCAGTGGCGGACGGGTGAGTAACGCGTAAGAACCTGCCCTTGGGAGGGGAACAACAGCTGGAAACGGCTGCTAATACCCCGTAGGCTGAGGAGCAAAAGGAGGAATCCGCCCGAGGAGGGGCTCGCGTCTGATTAGCTAGTTGGTGAGGCAATAGCTTACCAAGGCGATGATCAGTAGCTGGTCCGAGAGGATGATCAGCCACACTGGGACTGAGACACGGCCCAGACTCCTACGGGAGGCAGCAGTGGGGAATTTTCCGCAATGGGCGAAAGCCTGACGGAGCAATGCCGCGTGGAGGTAGAAGGCCCACGGGTCGTGAACTTCTTTTCCCGGAGAAGAAGCAATGACGGTATCTGGGGAATAAGCATCGGCTAACTCTGTGCCAGCAGCCGCGGTAAGACAGAGGATGCAAGCGTTATCCGGAATGATTGGGCGTAAAGCGTCTGTAGGTGGCTTTTTAAGTCCGCCGTCAAATCCCAGGGCTCAACCCTGGACAGGCGGTGGAAACTACCAAGCTGGAGTACGGTAGGGGCAGAGGGAATTTCCGGTGGAGCGGTGAAATGTGTAGAGATCGGAAAGAACACCAACGGCGAAAGCACTCTGCTGGGCCGACACTGACACTGAGAGACGAAAGCTAGGGGAGCGAATGGGATTAGATACCCCAGTAGTCCTAGCCGTAAACGATGGATACTAGGCGCTGTGCGTATCGACCCGTGCAGTGCTGTAGCTAACGCGTTAAGTATCCCGCCTGGGGAGTACGTTCGCAAGAATGAAACTCAAAGGAATTGACGGGGGCCCGCACAAGCGGTGGAGCATGTGGTTTAATTCGATGCAAAGCGAAGAACCTTACCAGGGCTTGACATGCCGCGAATCCTCTTGAAAGGGAGGGGTGCCTTCGGGAACGCGGACACAGGTGGTGCATGGCTGTCGTCAGCTCGTGCCGTAAGGTGTTGGGTTAAGTCCCGCAACGAGCGCAACCCTCGTGTTTAGTTGCCAACGTTGAGTTTGGAACCCTGAACAGACTGCCGGTGATAAGCCGGAGGAAGGTGAGGATGACGTCAAGTCATCATGCCCTTTATGCCCTGGGCGACACACGTGCTACAATGGCCGGGACAAAGGGTCGCGATCCCGCGAGGGTGAGCTAACCCCAAAAACCCGTCCTCAGTTCGGATTGCAGGCTGCAACTCGCCTGCATGAAGCCGGAATCGCTAGTAATCGCCGGTCAGCCATACGGCGGTGAATTCGTTCCCGGGCCTTGTACACACCGCCCGTCACACTATGGGAGCTGGCCATGCCCGAAGTCGTTACCTTAACCGCAAGGAGGGGGATGCCGAAGGCAGGGCTAGTGACTGGAGTGAAGTCGTAACAAGGTAGCCGTACTGGAAGGTGCGGCTGGATCACCTCCTTTTCAGGGAGAGCTAATGCTTGTTGGGTATTTTGGTTTGACACTGCTTCACACCCAAAAAGAAGCGAGCGACGCCTGGGTGAAACTTGGAGATGGAAGTCTTCTTTCGTTTCTCGACGGTGAAGTAAGACCAAGCTCATGGGCTTATTATCCTAGGTCGGAACAAGTTGATAGGATCCCCCCTTTTTCGCCCCCATGTCGCCACACGGGGGACATGGGGACGTAAAAAAGAAAGAGAGGGATGGGGTTTCTCTCGCTTTTGGCATAGCGGGCCTCCCACTGGGGGCCCGCACGACGGGCTATTAGCTCAGCGGTAGAGCGCGCCCCTGATAATTGCGTCGTTGTGCCTGGGCTGTGAGGGCTCTCAACCACATGGATAGTTCAATGTGCCCATCCGCGCCTGACCTTGAGATGTGGATCATCCAAGGCACATTAGCATGGCGTACTCCTCCTGTTCGAACCGGGGTTTGAAACCAAACTTCTCCTCAGGAGGATAGATGGGGCGATTCAGGTGAGATCCAATGTAGATCCAACTTTCGATTCACTCGTGGGATCCGGGCGGTCCGGGGGGGACCACTACGGCTCCTCTCTTCTCGAGAATCCATACATCCCTTATCAGTGTATGGACAGCTATCTCTCGAGCACAGGTTTAGGTTCGGCCTCAATGGGAAAAAATGGAGCACCTAACAACGCATCTTCACAGACCAAGAACTACGAGATCACCCTCTTCATTCTGGGGTGACGGAAGGATCGTACCATTCGAGCCTTTTTTTTTTCATGCTTTCCCGGAGGTCTGGAGAAAGCTGCAATCAATAGGATTTCCCTAATCCTCCCTTCCCGAAAGGAAGAACGTGAAATTCTTTTTCCTTTCCGCAGGGACCAGGAGATTGGATCTAGCCGTAAGAAGAATGCTTGGTATAAATAACTAACTTCTTGGTCTTCGACCCCCTCAGTCACTACGAACGCCCCCCGATCAGTGCAATGGGATGTGTCTATTTATCTATCTCTTGACTCGAAATGGGAGCAGGTTTGAAAAAGGATCTTAGAGTGTCTAGGGTTGGGCCAGGAGGGTCTCTTAACGCCTTCTTTTTTCTTCCCATCGGGGTTATTTCACGTTATTTCACAAAGACTTGCCATGGTAAGAAGGAAGAAGGAGCGAACAAGCACACTTGGAGAGCGCAGTACAACGGAGAGTTGTATGCTGCGTTCGGGAAGGATGAATCGCTCCCGAAAAGGAATCTATTGATTCTCTCCAAATTGGTTGGACCGTAGGTGCGATGATTTACTTCACGGGCGAGGTCTCTGGTTCAAGTCCAGGATGGCCCAGCTGCGCCAGGGAAAAGAATAGAAGAAGCATCTGACTCCTTCATGCATGCTCCACCTGGCTCGGGGGGATATAGCTCAGTTGGTAGAGCTCCGCTCTTGCAATTGGGTCGTTGCGATTACGGGTTGGATGTCTAATTGTCCAGGCGGTAATGATAGTATCTTGTACCTGAACCGGTGGCTCACTTTTTCTAAGTAATGGGGAAGAGGACCGAAACATGCCACTGAAAGACTCTACTGAGACAAAGATGGGCTGTCAAGAACGTAGAGGAGGTAGGATGGGCAGTTGGTCAGATCTAGTATGGATCGTACATGGACCGTAGTTGGAGTCAGCGGCTCTCCTAGGGTTCCTTAATCTGGGATCCCTGGGGAAGAGGATCAAGTTGGCCCTTGCGAACAGCCTGATGCACTATCTCCCTTCAACCCTTTGAGCGAAATGCGGCAAAAGGAAGGAAAATCCATGGACCGACCCCATCGTCTCCACCCCGTAGGAACTACGAGATCACCCCAAGGACGCCTTCGGCATCCAGGGGTCACGGGCCGACCATAGAACCCTGTTCAATAAGTGGAACGCATTAGCTGTCCGCTCTCCGGTTGGGCAGTAAGGGTCGGAGAAGGGCAATCACTCATTCTTAAAACCAGCATTCTTAAGACCAAAGAGTTGGGCGGAAAAGGGGGGAAAGCTCTCCGTTCCTGGTTCTCCTGTAGCTGGATCCTCCGGAACCACAAGAATCCTTAGTTCGAATTGGATTCCAACTCAGCACCTTTTGAGATTTTGAGAAGAGTTGCTCTTTGGAGAGCACAGTACGATGAAAGTTGTAAGCTGTGTTCGGGGGGGAGTTATTGTCTATCGTCAGCCTCTATGGTAGAATCAGTCGGGGGGCCTGAGAGACGGTGGTTTACCCTGTGGCGGATGTCAGCGGTTCGAGTCCGCTTATCTCCAACTCGTGAACTTAGCCGATACAAAGCTATATGATAGCACCCAAATTTTCCGATTCGGCGGTTCGATCTATGATTTCTCATTCATGGACGTTGATAAGATCCTTCCATTTAGCTTAGCAGCACCTTAGGATGGCATAGCCTTAAAGTTAAGGGCGAGGTTCAAACGAGGAAAGGCTTACGGTGGATACCTAGGCACCCAGAGACGAGGAAGGGCGTAGTAAGCGACGAAATGCTTCGGGGAGTTGCAAATAAGCATAGATCCGGAGATTCCCGAATAGGTCAACCTTTCGAACTGCTGCTGAATCCATGGGCAGGCAAGAGACAACCTGGCGAACTGAAACATCTTAGTAGCCAGAGGAAAAGAAAGCAAAAGCGATTCCCGTAGTAGCGGCGAGCGAAATGGGAGCAGCCTAAACCGTGAAAACGGGGTTGTGGGAGAGCAATACAAGCGTCGTGCTGCTAGGCGAAGCGGTGGAGTGCCGCACCCTAGATGGCTAGAGTCCAGTAGCCGAAAGCATCACTAGCTTACGCTCTGACCCGAGTAGCATGGGGCACGTGGAATCCCGTGTGAATCAGCAAGGACCACCTTGCAAGGCTAAATACTCCTGGGTGACCGATAGCGAAGTAGTACCGTGAGGGAAGGGTGAAAAGAACCCCCATCGGGGAGTGAAATAGAACATGAAACCGTAAGCTCCCAAGCAGTGGGAGGAGCACATAGGGCTCTGACCGCGTGCCTGTTGAAGAATGAGCCGGCGACTCATAGGCAGTGGCTTGGTTAAGGGAACCCACCGGAGCCGTAGCGAAAGCGAGTCTTCATAGGGCAATTGTCACTGCTTATGGACCCGAACCTGGGTGATCTATCCATGACCAGGATGAAGCTTGGGTGAAACTAAGTGGAGGTCCGAACCGACTGATGTTGAAGAATCAGCGGATGAGTTGTGGTTAGGGGTGAAATGCCACTCGAACCCAGAGCTAGCTGGTTCTCCCCGAAATGCGTTGAGGCGCAGCAGTTGACTGGACATCTAGGGGTAAAGCACTGTTTCGGTGCGGGCCGCGAGAGCGGTACCAAATCGAGGCAAACTCTGAATACTAGATATGACCTCCAAATAACAGGGGTCAAGGTCGGCCAGTGAGACGATGGGGGATAAGCTTCATCGTCGAGAGGGAAACAGCCCGGATCACCAGCTAAGGCCCCTAAATGACCGCTCAGTGATAAAGGAGGTAGGGGTGCAGAGACAGCCAGGAGGTTTGCCTAGAAGCAGCCACCCTTGAAAGAGTGCGTAATAGCTCACTGATCGAGCGCTCTTGCGCCGAAGATGAACGGGGCTAAGCGATCTGCCGAAGCTGTGGGATGTAAAAATGCATCGGTAGGGGAGCGTTCCGCCTTAGGAGGAAGCAACGGCGCGAGCCGCGGTGGACGAAGCGGAAGCGAGAATGTCGGCTTGAGTAACGAAAACATTGGTGAGAATCCAATGCCCCGAAAACCTAAGGGTTCCTCCGCAAGGTTCGTCCACGGAGGGTGAGTCAGGGCCTAAGATCAGGCCGAAAGGCGTAGTCGATGGACAACAGGTGAATATTCCTGTACTACCCCTTGTTGGTCCCGAGGGACGGAGGAGGCTAGGTTAGCCGAAAGATGGTTATCGGTTCAAGGATGCAAGGTGACCCTGCTTTTTCAGGGTAAGAAGGGGTAGAGAAAATGCCTCGAGCCAATATTCGAGTACCAGGCGCTACGGCGCTGAAGTAACCCATGCCATACTCCCAGGAAAAGCTCGAACGACCTTCAACAAAGGGGTACCTGTACCCGAAACCGACACAGGTGGGTAGGTAGAGAATACCTAGGGGCGCGAGACAACTCTCTCTAAGGAACTCGGCAAAATAGCCCCGTAACTTCGGGAGAAGGGGTGCCTCCTCACAAAGGGGGTCGAAGTGACCAGGCCCGGGCGACTGTTTACCAAAAACACAGGTCTCCGCAAAGTCGTAAGACCATGTATGGGGGCTGACGCCTGCCCAGTGCCGGAAGGTCAAGGAAGTCGGTGACCTGATGACAGGGGAGCCGGCGACCGAAGCCCCGGTGAACGGCGGCCGTAACTATAACGGTCCTAAGGTAGCGAAATTCCTTGTCGGGTAAGTTCCGACCCGCACGAAAGGCGTAACGATCTGGGCACTGTCTCGGAGAGAGGCTCGGTGAAATAGACATGTCTGTGAAGATGCGGACTACCTGCACCTGGACAGAAAGACCCTATGAAGCTTCACTGTTCCCTGGGATTGGCTTTGGGCCTTTCCTGCGCAGCTTAGGTGGAGGGCGAAGAAGGCCCCCTTCCGGGGGGGCCCGAGCCGTCAGTGAGATACCACTCTGGAAGAGCTAGAATTCTAACCTTGTGTCAGGACCCACGGGCCAAGGGACAGTCTCAGGTAGACAGTTTCTATGGGGCGTAGGCCTCCCAAAAGGTAACGGAGGCGTGCAAAGGTTTCCTCGGGCCGGACGGAGATTGGCCCTCGAGTGCAAAGGCAGAAGGGAGCTTGACTGCAAGACCCACCCGTCGAGCAGGGACGAAAGTCGGCCTTAGTGATCCGACGGCGCCGAGTGGAAGGGCCGTCGCTCAACGGATAAAAGTTACTCTAGGGATAACAGGCTGATCTTCCCCAAGAGATCACATCGACGGGAAGGTTTGGCACCTCGATGTCGGCTCTTCGCCACCTGGAGCTGTAGTATGTTCCAAGGGTTGGGCTGTTCGCCCATTAAAGCGGTACGTGAGCTGGGTTCAGAACGTCGTGAGACAGTTCGGTCCATATCCGGTGTGGGCGTTAGAGCATTGAGAGGACCTTTCCCTAGTACGAGAGGACCGGGAAGGACGCACCTCTGGTGTACCAGTTATCGTGCCCACGGTAAACGCTGGGTAGCCAAGTGCGGAGCGGATAACTGCTGAAAGCATCTAAGTAGTAAGCCCACCTCAAGATGAGTGCTCTCCTATTCCGACTTCCCCAGAGCCTCCGGTAGCACAGCCGAGACAGCGACGGGTTCTCTGCCCCTGCGGGGATGGAGCGACAGAAGTTTTGAGAATTCAAGAGAAGGTCACGGCGAGACGAGCCGTTTATCATTACGATAGGTGTCAAGTGGAAGTGCAGTGATGTATGCAGCTGAGGCATCCTAACAGACCGATAGACTTGAACCTTGTTCCTACATGACCCGATCAATTCGATCAGGCACTCGCCATCTATTTTCATTGTTCAACTCTTTGACAATACGAAAAAACCATTGTTCAACTCTTTGACAACATGAAAAAACCAAAAGCCCTGCCCTCCCTCTCTATCTAACCAAGGGATGGAAGGGCAGAGGCCTTTGGTGTCCCCTCCAGTCAAGAATTGGGGCCTCACAATCACTAGCCAATTTTCTCTCATGCCTTTCTTAGTTCGTGGTTCGATATTCTGGTGTCCTAGGCGTAGAGGAACCACACCAATCCATCCCGAACTTGGTGGTTAAACTCTACTGCGGTGACGATACTGTAGGGGAGGTCCTGCGGAAAAATAGCTCGACGCCAGGATGATAAAAAGCTTAACACCTCTCATTCTTATTACTTTTTCAATATGAAAGAAAAAAAAATGAAAAAAGGAAAAAAGAAAGGGTCGTCTTATTCAAAACCCCAATTATGACATCCCCTCTCTCCCACTTCACACCTCGGAACGCACCGTTCTTATAGATAGAAACGCGCTTTCACATCTTCTTAACCCGAAATGGCTGGGGAGAGGAAAGGTTCCTTTTTTTTTTGAGGATACTCCCGGGAACAGATCCAGTGGAGGCGGGGTGGGGCCTGTAGCTCAGAGGATTAGAGCACGTGGCTACGAACCACGGTGTCGGGGGTTCGAATCCCTCCTCGCCCACAACCTTCCCCTTTGGGAAGGACCTATCCCTCCGGGGATAGGAAAATCATGATCGGGATAGCGGACCCAAAGCTATGGGACTTGGGTGTGGGTCTTTTGCCGAAATGGAATGGGCCTTACCTTCCTTTTTTTTTTACGTGTTATCGTGAATGAGGTAATTTAAAATTATAGCATACCCCGGCCGTCGGCTGGCAGCATATTTTTTTGTTTTATGTCCCGTAACCCTTACTCAGCCAGGCTTGGGCAGAATAGCAGAGCAAGTACAAGTATTAGTAGCATAGCAAAAAAGCGTTCCTCGTCATTAATATGTTTTATGTTTGCTCGCGGTAATTGTAGCCTCTCGGGAGAACCGATGACTCCATCTTTGATGCACTGCTAGTACTAGCAGTGCATCAAAGAATTCTTAATTGGCTAGTTGTAAATAGCCCCAGGACTATGGAACAAAGGATTATCCCGGACCTACATCGAGGTATTGACGGAGATTCTCAAATATCGCAGAACAGAATGTCATACGATGAGATATAATACAATAGAAACAAAGACAGGGAACGGGTTACCTACTCTTAACGGTCAAAGCGAGCCCTTTCATTCTCATTCTGAATTCTTTAATTCAGAATGAATCAAATCTCCCCAAGTAGGATTCGAACCTACGACCAGTCAGTTAACAGCCGACCGCTCTACCACTGAGCTACTGAGGAACAACGGGGGATTTGATCTCATAGAGTGAAATTCCCGTTCTCAACCCATGACCCATATGAGCTCGAAGCTTCCTTCGTAACTCCCGAAACTTCTTCGTAGTGGCTCCCTTCCATGCCTCATTTCATAGGGAACCTCAAAGTGGCTCTATTTCATTATATTCCATCCATATCCCAATTCCATTCATTTAATATCCCTTTGGTGTCATTGACATAAGAGATCTCGTTTCTAGTCTATCTCTTTCTATTTCTATCTAGAATAAAATAAGTTAAACAATTATCTTATAATAATTATCATATAAGATAAGAAGGTAAAAACTTATCATATATAATAGAATCATATAGAATAGAATATAGAATAATATTAATATAATAATAATATAAATAGAATAATATAAATAGAATAATATAAATAATAGAATTTTCTAATTTTATAAGGTCAAATAATAAGGTCAAAGATTATCTTATAATAAGAAGGTAAAAACTTATTATATAATAATCATAATTCAGAAATTGCAAAATCACAAAGGGGATTTGTCATGATTTTTTTTCTACTAGGTAACTTATGCATGAAGATAGTCAATTCGGTCGTTGTGGTCGGACTCTATTATGGATTTCTGACCACATTCTCCATAGGGCCCTCCTATCTCGTCCTTCTCCGAACTCTGGTTATGGAAGAAGGAGAAGAAGGAGAAGAAGGAACCGAGAAGAAGGTAGCAGCAACAACCGGTTTTATTATGGGACAACTTATGATGTTCATATCGATCTATTATACGCCTCTGCATCTAGCATTGGGTAGACCTCATACAATAACTTTCCTAGCTCTACCCTATCTTTTGTTTAATTTCTTCTGGAGCAATCATTTTGATTATGGATCTACTACCAGAAATTCAATGCGTAATCTCAGCATTCAATGTGTATTCCTGAATAATCTCATTTTTCAATTATTCAACCATTTCGTTTTACCAAGTTCAATGTTAGCCAGATTAGTCAACATTTATATGTTTCGATGCAACAACAAGATGTTATTTGTAACAAGTAGTTTTGTTGGTTGGTTAATTGGTCACATTTTATTCATGAAATGTGTTGGCTTGGTATTAGTCTGGATACGGCAAAATTATTCTATTCGATCGATTATTCGATCTAATACGTACCTTCTTAATGTACTTATTCGAGCTATTAATGTACCTATTCGATCTAATAAGTACCTTCTTAATGTACTTATTCTATCTAATAAGTACCTTAAAGTAATACTTATTCGATCTAATAAGTACCTTAATGGAATTATTCGATCTAAGAGGTATAAGAAGTACAAGTACTCTGTGTCAGAATTTAAGTACCTTGTGTCAGAATTTAAGTACTTTGTGTTAGACTTGATAGATTCTATGGATCGAATCTTTAGTATTCTCTTATTTATTAGCTGTGTCTACTCTTTAGGCAGAATGCCGTCACCCATTTTTAGTAGGAAACTGCAAGAAACCTCAAAAACGACAGAAAGGGGGGAAAGTGAGGAAGAAAGAGATGTAGAAATAGAAACAACTTTCGAAACGAAGGGGACTAAACAGGAACAAGAGGGATTCACCGAAGAAGATCCTTCTCCTTCCCTTTTTTCGGAAGAAAGGGAGGATCCGGACAAAATCGATGAAACGGAAAGGATCCGAGTGAATGGAAAGGACAAAACAAAGGATGAATTCCACTTTCACTTAAAAGAAGAAGATAAAGACCTCTTCTGGTTTGAAAAACCCCCTGTGAGTCTTCTTTTCGACTATAAACGATGGAATCGTCCATTGCGATATATAAAAAATTATCGATTCGAACATGCTGTAAGAAATGAAATGTCACAATATTTTTTTTATACATGTCAAAGTGATGGAAAACGAAGAATTTCTTTTACATATCCATCCAGTTTATCAGCTTTTTTTGAAATGCTACGACAAAAAATGTATTTTTATTTTTTTACAACAAAAAAATTCGTCTGTGATGAACTGGATAAATTCTTCTATGATGAACTGCATAATTATTATTGTTGGATTTATACCAATGAAAAAAAATGGAGGAGCCTAAGGAACGAGTTTACAGATAGAATTGAAGCCCTAGACAGAGGATCTCCTTATCTGGATGTACTCGAAAAAAAGACTCGATTATGCAATAATAAAACTAAAGAAGAATACTTGCCTAAAATATATGATCCTCTCTTAAACGGATCCTATCGTGGAATAATTAAGAAATTTTATTTACCTGCAATCCTAAATGAAACTGCAGTCAAAAATTCGATAGAGACAAATTTTATAAATAAACTCAATAAAGTTCATAGTATCCTTCTTTTTAAGGGTAAGGAACTTAATGTTCATAATTTTGAAGAATTGTACCAGAAATTGGAAGGGAAAATAGCTACATTGGAAGAGAAATTGGTCCAGAAATTGGACCAGAAATTGGAAGATAAATTGGGACAGAAAATATATACATTGGATAAAAAATCATTAGCAAGAGAATTGAGTCTTTTAATCGATGAATTTGCTGAAGAATCAACATCAAATTTGAAAGGAATTTCTTTATTTCCGGAACAAAGACGAATTGATTCAGAAGATCCAGAAAAAGTTTTGAAATTTTTAATCGAAAGAGTCATAATTGATCCCATCATTCAAACAATATGCGATACAGCCATAATTCCTCCCATGGAAAAAACAACTCGAAAAAAATCGATTGGAATAAATAAAAAAGTCCCCCGATGGTCATACAAATTATTCAGCGAGGTAGAACAACTCGGAAAAACTGCAACAACGGAAGAGGGGGAAGAGTGGATAGTAGATCATCAAATTCGCTCGAGGAAAGCGAAACGTATAGTTCTTTTTACGCAGGGTCCGGAGGAAGCAGAGAATGCCGACCCTAGTATAACTATGACGAAGCCTGATGAAATAGAAGAAGTGGATATGATAGATTATCCCTATGAAGCGGATTTTCGTCGAGACATAATCACAGGTTCTATGCGTGTTCAAAGACGTAAAACCCTTACGGGGAAAATGTTTCCCTTATATCCGTATTCCCCACTTTTTTTCGACAGAGTAGGATTTTCTTGGGATGTTCTTTTCGAACCATTCATATTATCAGTAATTGAGATTTCCGACCTAATACAAGACATTTTTAGAAAGGGTATAAAAGGAAGCGTAGCAAAAAGAATAAAGAGGTTGAAAAAACAAAAAAAAATGTACATGGAGGAAAACAAAAGCTACGAAGAAATTCAAAAAGAAGTCAATGAAATGGAAAAGGGGCGGGACGGGCAGACGGAAATGGAACGAATAGAAAGGACACGAGAAAAAATATCAGACCTCTATGATATCCTTATTTATGCTCATGGAATAAGAGCTTTTATTTTACTAATTCAGTCGAGGCTTAGACAATCTATTGTATTACCTTCATTGATACTAGCTAAAAATACTGTCCGTTTCTTATTACGCCAAGAGTCCGAGTGGGAGCAGGATATAAGGGAGATGAATAGAGAAGTGTATGTTATATGCACCTATAATGGTATGCCAGTACTAGAACCAGGAAGAAACGGAATTTTTCCTAAAAACTGGGCCACAGAGGGTATACAAATAATGATACGATTTCCTTTCCGTCTGAAACCTTGGCACCGATCTAAGATACGACCTTCTCGTAGGGATCCAAATCCAAAGCAGGAAAGTCCTGCTGCTTTTTTAACGATTTGGGGACTGGAAACTGACCGTCCTTTTGGTTCTCCTCTCGTAGGACTTGGTATTTTGTTTTGTTATTATTTTGGACCCCCTTTGAAAAAACTCCAAAAAGCAATTATAAAATGGAGTTTTCGAGTTCTAAAAAGTTTCAAAGAAAGAACAAAATTTTTGTTTCTAAAGGTCCAAAAAGAACCAAAAAAATTGAGAGAGGATTCGAGTGAAATAAAAAAAGATTCTATAATCAATAATCAGATTATTCATGAATCATCCATTCAAACCCGATCTATGGATTGGACAAATTATTCACTGACAGAAATCAAAATGAAAGATCTGACTGATAGAACAAGCACAATCAGAAATCAAATAGAAAGAATTACAAAAGACAAGAAAAATGGATTTCGAACTCTAAAGATAAATATTAGTCCTAACAAAACAAGTTATGGTGCTCAAAAATTAGCATCACTAAAAAATATTTTTCAGATATTAAAAAGAAGAAATGATCGATTAATCCGTAAATCACATTATTTTATAAAATGGATCGTGGAAAGGATATACACGGATATCCTTCTAGAGAGCTTTCCATATATCATTAATCGTCTTACTAATAGTCTTTATAGGCCCATGATCATTATAAAACTTTTTCGTAAATTAAAAAAAAAATATATTTTTGATACAAAAGAGAAAAAGATAATTGAGCGTATTTCAACTATACAAAAAAAACTTTCACCTTCTCGTATTCGTCATAAGATTCAGACGAAGTCGGAGGTTTCTTTTAAATTATCCCTCGTGTCACAGGCCTATGTATTTTACAAATTATCACAAAGCCAGGTTATTAACTTGTATAAGTTAAGATCTGTCCTTCAATATGACGGAGCATCTTTATTTCTTAAGAATGAAATAAAAGATTATTTTCGAAGACAAGGAATAATTTCTTCCGAATTAAAGCATAAGAAACTTCAGAATTCTGGAATGAATCAATGGAAAAATTGGTTAAAGAGTCATTATCCATACGATTTATCTGAGCTAAAATGGTCTAAATTAGTACCGCAAAAATGGCGAAATAGAGTCAATCAACATTGTAGGGTTGAAAATCAAAATTTAATCAAACGGGATTCATCTGAAAGAGAGGAAAAGGTTTCGTTATTGCTAAATAAAAACGATCATTTTCAAAAAATGTATAGATATGATCTTTTAGCATATCAATCGATTTATTATGAAGATAAGAAGGACTCATATAATTACAACATACATAAACCGAAATTAGTTGATATGGGGGGGAGTATCCCTATTACTAATTTTATAAGAAAAGATTATTTTATGTATATAAAAAATCCAGATAGAAAATATTTTGATACGAAAGGTCTTTTTTATCTCAAAATTTATAAAGATAAAGAAATCAACCCACCCAATCAAAAGGGTTTCTTTTCTTTTTTTGATTGGATGGGAATGAATGAAGAAAGACTAAATCGTCCTGTATCGAAGCCGATACCTTGGTTATTCCCACAATTTGAGTTATTTTATAATGTATATAAAATGAAACCCCGGTTTATACCAATTCATTCACTTATTTTTCATTTTAATGAAGATGTTAGTCAAAATCAAAATATCACTAAAAATCAAAAAGGGGATCTTATACTATCAAATGAAAAAGAAAAAAAATCTTTTGAATTAGAGAATCAAGAAGAAAAAAAACCCATAGGTCAAAGAGATCTCGCATCAGATGCCCAAAACCGAGGGAACCCTGAATCTGTTCTCTCAAACCAACAAAAATATATGGAAGAACTTTATACGAAATCAGATATGAAAATGGGTAGAACGAAAAATCAATCCAAAAGCATTTGGACTTGGGAAATAGACTTAGATGCGTTCATGAAAGGATCTTTTGCTTTGCAATTGAAATGGCTGCTGAGTCCTTTGACTATGGAATTATTCGATTATGCGCTGTCCGTACTTGAATGGGAAAAGGAAAGCAGAATGACAACCAAGTTTGGTTTCGGCTTTATTAAGAAGGAGGAGTTAACTCTGGATCCAATGCTAATCCGGGATTTGAATCTTTCAAAAATCCTAAAAGAGGGAATATTTATTATCGAACCGGTTCGTATACCTGTAAAACATAATGTAGAATTTATTATGTATCAAACCATAAGGATTTCTTTGGTTCATGAGATTAAACAAAAAAATAATCAAAAAAGATACAGAGAAAATATGGATAAGAATCATTTTGAGGAATCGATTGCAAGACATCAAATGATGACGAAAAATAGAAACAAAAATCATTATGATTTGCTTGTTCCTGAAAATATTTTATCGTCTAGACGGCGTAGAGAATTGAGAATTCTAAGTTGTTTCAATTCAAGGAATAGTAATTGTGTGGATAAAAATGCAGTATTTTGCAATGGGAACAAGGTAAAAACCTGTGGTCAATTTTTGGATGAAAGCAAAGATCTTGATAGAGATAAAATGAAATTAATTCAATTTAAATTCTTTCTTTGGCCCAATTCTCGATTAGAAGATTTAGCTTGTATGAATCGCTATTGGTTTGATACTAATAATGGTAGTCGTTTCAGTATGTTAAGGATACATATGTATCCACGATTGAAAATTGATTGATGATACAATTGTCTTATATATCCCCTACCATATATATCGGGTGGATAAATAGCTGCGCACATGCCTTGTCTTACATCCTTTTTTGATACATGAATACTAATTCAATGACGTATCAATTAGATCATAAAATGAATCAATAAGGAAATTCGGATTGATTATTGTGTATACCAGATCAAAATACCTCGCATTATTATTACTGATCAGTAAAATTCATATTCGTAAAATAAAAAAAGTAAATTAATAAAAAAATTGACGCATAGAATATATACAAAAAAAGATAAGAAGAAATGCGCCCCCCACCTACATACTTGATACCTTCTCCTACAAAAAAACTTGTAAGACCAAATCCATTTGGAATTCCATCAATTACTCGTCTGTCAAAAAAATGAACTAGTTCGGACAATCCTCTTACACTCCGAATTACGTATGTTGTATAAAAAGCATCTATGTAACCACGATGATGGGCCCAATCATATATTACATTTTGAATTTTGTCCGAAAAAGGATGCTTTTTGGCAAAAAAATTAATTAAATAAAAATTTTGTAACGATGAATAAATGGGTTTATATAAAAAAGACGCTATAACTATTCCAGAATAAGCTATACTAACCGACAATGTTGCATTTGTCACAAATTCAGACCAATCAAAAGAATTTTGATTATTTAATTTTTGATGTAAAAGGTTTATAGATGGAGTTAACCATTTGGACAATATATCCAAGTCTATGCCTTCTTGATTGAAAGGAATTCCTATGAATCCAATGAACAAAGTAAATAAAATCAATACAAGTAGCGGGACTAACATAGTATTCCCCGATTCGTGAGGATATGCCGAAATTTTTTTATTGTCACAATTATTAATAATAATAAAGGATCGCATCATTTTTTTTATATTTCCGTGTGAATTCTTAGAAACCCTTTCATTATTTTTTATTGGTAACAAAGTTAATAAACGAAAATTTTTGTTAATAGGTTTCAGTCCTTCTTGACCCCATAAGGATATTGAATAGAAGGAGCTACTTTTTTGTCCATTGTAATTTTGAAAATGAACGTTTAAATGACCTTCAAACGTAAGTAAATAGATGCGAAACATATAAAATGCAGTTAATCCTGCTGTAGCCCAGGATAGAATTGCGAAAATTGGTGAATACAACCAAGTATCATTAAGAATTTCATCTTTGGACCAAAAACAAGCAAGAGGTGGAATACCAGAAAGAGAAAGTGTACCTAATAAAAAAGCTGTTTTTGTAATTGGCACGTGTTTTTTTAACCCCCCCATAAAAACCATATTCTGACTTTTATCTGGAGAATATCCAACAATAGCTTCCATAGAATGAATAATGGATCCCGATCCTAAAAACAATAATGCTTTTGAGTAAGCATGAGTAATCAAATGAAATAAAGCAGCTCGATAAGACCCCATACCTAGAGCTAACATCATATACCCCAATTGAGACATTGTAGAATAAGCTAAACCTCTCTTAATGTCTTTTTGAGCAAGAGCTAAAGTAGCTCCTAATAGTACTGTTATTATACCTATTAAAGCGATTAGATTTAGTATGGAGGGGATTACTATCAAAAGAGGAAAAAGTCGAGCGACAAGAAAAATCCCCGCTGCTACCATAGTAGCAGCATGTATAAGAGCCGAAATAGGAGTAGGCCCCTCCATAGCATCAGGTAACCATACATGAAGGGGAAATTGGGCGGATTTGGCAACTGCACCAGCAAATAATAAGAAAGTACATACAGTTCCAACTAAAAGATGGACTTCATTAGTATAAGTCAGGGTATTGAATATTTTGAACAAATCTCGAAATTCGAAACTGCCCGTTAGCCAATAAAGACCTAAAATTCCTAATAATAAACCAAAATCCCCTACACGATTAGTCACAAACGCTTTTTGACAAGCATTTGCTGCAATAGGTCGTGTAAACCAAAAACCTATTAATAGATACGAACACATTCCAACTAATTCCCAAAAAATATAAATTTGTATTAAATTCGAACTAGTAACTAAGCCAAGCATAGAAGTATTGAAAAAACTCAGATAAGCAAAAAATCTCAAATATCCTTGATCATGAGACATATAACTGTCACTATAAATAAGAACTAGAATCCCAACAGTAGTTATTAACATTGACATAATAGAAGTAAGTGGATCAACCAAGTAACCGAACTCTAAAGAAAAATCATTATTGATGGTCCAAGACCATACAGATTGATAGATAGAACTGCTATTTATTTGCTGAATAGACAATTTCATTGAAAAAATCATAACTATACTTAACAACGAAATACTAGGAAAAGCCCACATACGCCGAAGATTTTTTGTTGTCATCGGAAAAAGAAGAAGTCCCGCTCCGATTAACAAAGGAACTGTAAGTGGAATAAAAGGTATGATCCATGCATATTGGTATATATGTTCCATAAAAAATAAAATTTGATTTTCGATTCGCCGGCTCTTACCTCTTTCGAAAGAGGTCAATAAAAAAATTAAGATATGCAATAATAGAATTTTCAAATTTTCTATTCGAAATTCTTAGAATAAGCATTTTATTTATATTCAACTCAAGAAGTTCTAATTGGTCAAATGACCGAGTAGTTATTAGTGAAAGTAAATACTTAGTTATTAAGTAAACTATTAAAACCTATGAATATAGAGAATATCGAAAATTTATATTTTCCATTTTTGATAAATCCATAGATAGAAAAATGGTAAAAAATTAAACCAAAAAGTACTTAAGTCTGATACTGATATGAATCACAAGATCTACTAAAATTCATAACATAATTTGAGCCAAAAACTAGGAACTATTTCAATTAGTTTATTCGGAATCAGTTATTAGTTATCTGCGAAACTCTTTGATTGATTGTCTTCTATTCCAATCCAATAAAGCATATTTCTCGTTTTCTATGCTAGCCAAGACTTTACTTTCGACTTTACATAACATAAAATTAAAAAAAATGATGAAAACATATCAAATCATGTCTACTTTACTAAATAACAAGTCTCATTTCAATTTTTAAATAATAAAAAAAATGCCATGTATTTGTTAAAAGGAGTCAAGTTTTTCTATTAAGTAATTAGGTAAGAGTAGCTTGACTCTTCTAAATTTAAACCCTTCGACGTGTTTATTACATGACATGTAAATACAATATAAAATACAATAAAAGTCACATAGCAAAAATAAACATAAATAGAAGTCTAAAGTTTGCTTCTTGATTTTTTTTACGGCCTACCGTATAAATAGAAATAGAAATTTGAATAAGAAAAATGGGAGTCTCTCATAATATGTAATTTTCATATATTTCGAGTTTTTGAGATTTTTTATAAAAAAACTTAGAATAGAAAAACGTCTATAACTAAAAAAAAGGACAGAAAGATTCCTTTGAACAATAGATGTCTTTCACATCCAACTATAACAATGAATAACCTATTTTTTTTTGAATGGCAGTTCCAAAAAAGCGTACTTCAAGTTCCAAAAAGCGTATTCGTAAAAATATTTGGAAAAGAAAAGGGTATTCGGCAGCATTAAAAGCTTTTTCATTAGCGAAATCTCTTTCTACCGGCAATTCAAAAAGTTTTTTATACGAAAAATAAGTAATCAAATGTTAGAATAATCTGAATCGATCTGACTCAAAAAAACTTCTACAAAATTGCATTTAGCATTTATAAGCATTTATATTCATAAAATAAATAAAAAAAAATTAAAATATAAAAAAAATCAATCATTTTCATATAGATATAGAATTCATTATTTGTACTCATTAATATTTTATTTGAACTGATCAACATAAAATAGAACTTGCTTCTCTCTTATGATATGTAGACTAAAAATACGTCTGTATACTCTAAAATGGTACTTTTTTGTTTGAAAACTAGAAGATTTCACTAACCTTCTTTTAGTATATTTTCTCATTTCTGGGATGGGGATTCTTACTTTCCCCATCAACCGACTGGTCACAATATTAATATTAAATAAAAGGTTTTTATATCTAAAGAAAAAAAAATGAAAAATGGAAGAGCAAACTAAAAATCTTTAGTAAAAAAAGGCATCCTTAAAAAATGGATTCAATTAAAAATTTTCCCTCTCTGATTCATTATGTCTCTGAATTGTTATATATCTCCTGATTTTATCAAAAAAAAAAGAATCTTTATTTATGATTTTGAGAATTTATATTTATTATATTCTATTTATTTTATTATATTCTATTTATTATTTTTATTTATTATATTTATTTTATTATATTCTATTTATTATTTATATTTCTATTATTTGATTTTTATTTCATTTTATATTTCTTTTTTTGTTTTTTAATGTTTTCTATTTTATAAAAATATCGCCATTGAATTGACTCTTTCAATCTCGACGATTAAAGATAAATAGGCTATTATGATTTCAAACAAGCCGCTATGGTGAAATTGGTAGACACGCTGCTCTTAGGAAGCAGTGCTAGAGCATCTCGGTTCGAGTCCGAGTAGCGGCACAGCATCTTATAAATTCGAAAAAAGAATTTAATAGTCCTAGAATGAATAAGAATCCCAATGAGATGAATTTCATTCTTGATTTCTATTTCGTAATTTGTAATTGAGGGATCGGATCTACTTTCTTTTTTTTATATATATATTCTTATGATATTTTTTACTTTAGAGCATCTTTTCAATCATATTTCCTTTTCGACCATTTCTATTGTAATTACAATTCATTTACTAACTTTAGTAGTCAACGAAATAGTAGAACTAGATGATTCATTAGAAAAGGGCATGATACTTACTTTTTTCTGTATAACAGGATTATTAGGCATTCGTTGGATTTATTCGGGGCATTTCCCGTTAAGTGATTTATATGAATCATTAATCTTCCTTTCGTGGAGTTTTTATATTATTCATATGATTCCTTATTTAAAAAAAAAAAAAAATCATTTAAGTGCAATAACGGCGCCCGGTGCTATTTTTACCCAAGGCTTTGCTACTTCGGGCTTTTTAACTCAAATGCAGCAATCCACAATATTAGTACCCGCTCTCCAATCCCAATGGTTAATGATGCATGTAAGTATGATGATATTGGCCTATGCAGCCCTTTTATGTGGATCATTATTATCAGTAGCCCTTCTAGTCATTACATTTCAAAACAATATAAGTATTTTTGGTAAAAAAAAACTTATATTAAACGAGTCTTTGTTCTTCGAGAAGATCCAATACATGAATGAAGAAAACAATATTTTACAAAGCACTTATCTCCTTTCTCTTAGGAATTATTATAGGTCCCAGTTAATTGAACAATTAGATCATTGGAGTTCCCGTGTTATTAGTCTAGGATTTATCTTTTTAACCGTAGGTATCCTTTCAGGAGCAGTATGGGCTAATGAAGCATGGGGATCATATTGGAATTGGGACCCAAAGGAAACGTGGGCATTTATTACTTGGACCATATTCGCGATTTATTTACATATTAAAACAAATATAAATTTGCAAAGTGAAAATTCTGCAATTGTGGCTTCTATAGGATTTCTTATAATTTGGATATGCTATTTTGGGGTCAATCTATTAGGAATAGGACTACATAGTTATGGTTCATTTATATTAAAAAGCACCTAAATTGAATTCAAGAAAGGACCTAACCTGACGAATACAACCACAGGACAGGGTATATCCCATATATAGAAATAAGCAAGCCTCGCCGAGAACCATTTCAATCAAGTAGTATAGTGATTCAAATGGTTCTCACAAACGTCAAACTATCCGATTATAATTCATATTCGTTTTTGCCTTACGTAAAAAAGACTTTTTTGAAATGAAAACTATCTATAAAAAAAATTCGATAGAATAGCTTCTACCTTCTCAACTGATAGTGAGAGAACGAAATCTGGGTAAATGCCAATACCTATTACTGGTAGAAAGATAGCGAGTGAAACAAATAACTCTCGTGGACCAGAATCAAAAAAATAAGAGTTTGCACTATTAAATAACCTGTATCCATAGAACATTTGGCGTAACATAGATAATAAATAAATAGGAGTTAATATCATTCCAATTGCCATGCCAAAAGTAATTAATACTTTTGACATGAAAAAATATTTTTGGCTGGTAATTATTCCAAAAAATACTATTAATTCGGCAAAAAAACCACTCATGCCCGGTAACGCAAGGGAAGCCATCGAAAAAGTACTGAAGAGTGTGAATATTTTTGGCATTGAAATGGCTATTCCGCCAATTTCGTCGAGATAAACAAGACGTATTCTATCATAACTCGTTCCTGCTAGGAAAAAAAGCGCAGCACCAATAAATCCATGAGAGATTATTTGTAAAATGGCCCCGTTGAATCCCGTATCAGTTATAGAACTAATTCCTATAATTATGAAACCCATATGAGACACAGAGGAATATGCTATTCTTTTTTTTAAATTACGTTGCCCCGGAGATGCTGAAGCTGCATAGATTATTTGTATTGTGCCTACTATCATCAACCAAGGAGAAAATATAGAATGAGCGTGAGGTAATAATTCCATATTGATCCGAACCAATCCATATGCTCCCATTTTTAATAGGATTCCAGCTAGAAGCATACAAGTACTGTAATGTGCTTCTCCATGGGTATCCGGTAACCATGTATGTAGGGGTATAATCGGCGATTTGACAGCAAAAGCAATAAGAAATCCAATATAGAATATTATTTCTAATCCCACAGGATACGATTGATTAGCTAACGTTTCCAAATTTAATGTTGGTTCATTGGAACCATATAACCCGATACCCAAAACTCCCAGTAACAGAAAAACGGAACCCCCTGCTGTGTACAAAATAAACTTTGTAGCTGAGTATAGACGTTTCTTTCCTCCCCATATGAATAAAAGCAGATAAACGGGAATTAATTCTAATTCCCACATTATAAAAAAAAGTAAAAGGTCCCGCGAAGAAAATAATCCTATTTGACCACTATACATTGCTAACATCAAGAAATGGAATAATCGGGAATCTCGAGTAACAGGCCAAGCCGCTAAAGTAGCTAAAGTTGTGATGAATCCCGTCAGTAAAATGGGTCCTATAGAAAGCCCGTCTATTCCCAATCTCCAGTGGAAATCAAAAAAGTGAATCCATTTATAATCTTCTGCCAGTTGTATTAATGGATCGTCTGGTTGAAAATGATAACAGAATACATAGGTTGTTAGGAGGAATTCTAATATACATATACACATAGTATACCATCGAATTACCTTATTACCCCTATGAGGGAGAAAGAAAATTAATGAACCCGCAAATATAGGCAAAACTACAATTAATGTTAACCAAGGAAAATAATTCGTGGTAAAGACAAAATACACTTGGACTAAAAAACCCGTACTCGAATAAGAACAAAATAAGATATATATATATTTCATTTCGAGCGCGGGTTTTTGTCGGTAAACAAAAATCAAATAGATTCAAGTGGAGTTTTCTGGAACGTATCAATAAGCTAGACCCATACTGCGAGTTGTTTCATGCCATAAATAAACTCGAACACTCAAAAAATCGGTTGGACAGGCGGATTCACATCTCTTACAACCAACACAGTCCTCTGTTCTTGGGGCGGAAGCTATTTGCTTAGCTTTACATCCGTCCCAAGGTATCATTTCTAATACATCTGTGGGGCAGGCTCGGACACATTGAGTACATCCTATACATGTATCATAAATCTTTACTGAATGGGACATTGGATCTATACCTTTTTTTGAATCTCATTAATTTTCGATCTAGTATAACCCTGTATTATGTATTATATGTAAATGAATTACATATTCAAAGACCAGACGAATCGATGATTCACCAGAATTTGTCGAATCAACTTATTTCTGGGTCGGTTTAGAAAAGAGGTCAAAAATACTTTGATTTCTTACATTTTTGAAGATTCTACATACCTAGTAATCTAATTTGAATTGATAACTATTCAAATTTCTATAATAATAATATTAATACTACTTATTCAAATATTAATACTACTTATTCAACAAATTCGATTGATTAATACGAGTTGATTTTCTGTTACGATAAATTGCCGAAACAATAGCCGGTCCAATAGCTGCTTCAGCGGCTGCAATAGCTATAACAAAAATGGAAAAAATGTTTCCTTTTAATTGACGACTATCAAAAAAGTCAGAAAATGTTACTAAATTAAGATTAACCGCATTCAATAGAAGTTCAAGACACATAAGAGCTCGAACTAAATTTCGGCTTGTGATTAATCCATAGATACCGATAGAAAATAAATAGGCACTCAAAACAAGTACATGTTCGAGCATCATTGAACAACTCCTTATCAATCTTGATTCATTTCATTTCAATATGAACAATAATTAAAGCGATTTCGTTGACTCGACTATAACAAATACAGAGCAAAGGAGTATGTTAGTAATAGATTGACATTTATATTTTATATTATACATCAATTCAAATGGAATTGAATGGAAATCGATACGATAAAACAGAATAAAGTTTGATTTGGATTGATGCTTTTAAAGATTTATTATTTTATTGACGGGCCACGGCAATTGCACCTATCAAAGCAACTAAAAGAATTATCGAAATGAGTTCAAATGGGAGAAAAAAATCTGTTGATAAATGAATTCCAATTTGTTGACTATTATTTATCAAATCTTGTTCTATAATCTGGTTTAATCTTGTAGTCCAAATAATTCCGTACCATGACGTATTTAGAATAGTAGTAATTAATAAAACAAAAATACTGGCACAAACTAACAAAGTAATTCCATCCCCAAGAGTCCAAAGACGAAAATCTTTGTCATATTCTAACCCGCTGATAAACATTACAGCAAATATTATTAAAACATTTATAGCTCCCACGTAAATAAGGAGTTGGGCAGAAGCTACAAACTGAGAGTTTGCTAGAATATAGAATAAAGATATACAAACAAGAACCAATCCCAACGAAAAGGCAGAAAAAATGGGATTGGTAAATAATATCACTCCTAGGCCTCCTAATATAAGACCTGATCCCAGAAAGACTAAAAGAAAATCATGTATTGGTCCAGGTAAATCCATTCGATGAAAAAAAGATATAATAAATCGGACTCTTTCATGATCTTATTGAACTGACCAGGAAAAAATAAGTTAAGTTGATCTATTTAGGACACGTTCCTAGTTGAATGCAATTCTAATGGATGTGAATTGGTGTAGGTACAGTTAGTGGACTAATCACATTCTTTATCGGAAAGGCTAATTCGAATCTAGTTGAAATCATTACATTAAAAAAATTTCCAAGTAAATCTACAATTTTCATGAACCAATCAGATCAATAGTTGTTATTTTTAGTTTAGTTATTCACAAAAAAAAAAGAAACCTGTGAAATTTATATAACAACCTTAGTAATAAAATAAATACCGTTCTTGAATCAAGGTATTTCTTTTTTATTGAATTGAGGCCAATTCAAGATAGTTCGAATTGTATAATCGTCAATTATTGATATTGGTAAACGGCCTAAAGCAATTTGATTATAATTTAATTCATGGCGATCATACGTAGAAAGCTCATATTCTTCAGTCATTGATAAACAATTTGTTGGGCAATACTCAACGCAATTACCACAAAATATACAGATTCCGAAATCAATACTGTAATTAAGCAATCGTTTCTTTCGAATATCAGTTTCCAATTTCCAATCTACAACAGGTAGATCTATAGGACATACACGAACACATACTTCACAAGCAATGCATTTATCGAATTCAAAGTGGATTCGACCACGAAAACGTTCTGATGTGATCAATTTTTCATAAGGGTATTGAATAGTTACAGGTAAACGATTCGCATGGGATAAGGTAATCAGAAAACCTTGACCAATGTACCTTGCCGCTCGTACTGTTTGTTGACCATAATTCAGGAACCCAGTTACCATAGGGAACATATCCTAAATATCGATAAAAAAAATATTTTGTTTGTTTCTTTCTCTTGTTTGGGACAAGTTATCACTCTACTTACTAGTGAATAGAAAATATTCGATTTTGCTTATAGTGAAAGGAGTTGGGAAGAAGTTGTTAATAATAAATTACCTAAAGAAATAGGTAAAAGAAATTTCCATCCAAGATTTAATAATTGGTCCATTCTCAGTCTAGGTAACGTCCATCTTGTTGTGATAGAAATGAACAAGAACAAAAAAGTTTTCGCTAGTGTAATGAAAAGACCAATTGTTGTTCCAAAGACTCCATCCCTTGCATTTATTTCAAAAAGGTCAGGAACGAATATGTACGGAATAGAGAAATTCCAGCCTCCCAAGTAAAGAACTGTTACAAATAATGAAGAAACTAGTAGATTTAGATAGGAAGCAACATAAAATAAACCAAATTTAATACCTGAATATTCTGTTTGATAACCTGCTACTAATTCTTCCTCTGCTTCTGGTAAATCAAAAGGCAATCTTTCACATTCGGCTAGAGAAGAAATTAGAAAAACGAGAAATCCTATAGGCTGCCGCCACAAATTCCACCCCCAAAAACCGTATTTGGCCTGTGCCTCAACTATATCAACTGTACTCGAACTGTTAGATAATTCTAGTCGGTGATAAGATCACTGTTATCATCGCTATTACAGAACCGTACATGAGATTTTCACCTCATACGGCTCCTCGAGGGCCCCATATAAATCTAAGGACTGCTTCGATATTCTTTATATCTTGATATTTTTGTAGGATAGATAGAGTCAAAATCAATCGAAAGGTCCCGAATTAGACCAATGGAATTCTGTCTGCTATACTAGATATAAGGGCTTCTGAATTGATCTCATCCTTTACTTTTTTAAATTAAAAATTTTTTTATTTAATTTTATTAATTATAAATTTTCAGTTTTCTTTATTGAGATTTAATTTAAACACTCTTTTTAGAAATATTAATAGATATCTCACCCTATCCTTTTTTAGTACGAAAAGAAATTAACATGAAGCATGACATGAAGTGTAGCTCTCTTAATACAGCTATAGGAAAGCAAGAATACTAAAAAATTTTGCAATTACATTCTTTCTATTTTTTTATTCCTTTTTTTATAAAAAAAAAAGAAGTAAAGGATTACTTCGTTCCTGATAGTCATTCACTTAATCGGTGGATAGGAGCATACTCTGGATCGGAATTCTGGGGAGTACTACTTGATCATTTCTACAAATTTTAAGCCCCAATTAGTATTTCGTTTATGTGGAATTTTTTTCCGATAATTTAGAAAATCTCTATTACTAATCCTTTGTGTAACTTGGTGTTCCTAACCATCCACTCAGTTTTGCTCAATCTCTGCGGTAATTCGTGTCATGTATAGTAATACATACAAACGATAGCACGAACTCCAAAGAGTGGATCTGTTTAACCCGCTTCAAGCCATGATAACTAATCAACCAGTCTTGGGGTAAATTGTTTTTCTTTATAGTTTTTCTTTACTGCTTCTATTTACTTATATTTACTTTGGCATAATTCTTGTACATAGGAAATGAGACTCAATCTTTTTACTGCGAATTTCGAAGCTGTTTTCTTTCACTCATCTAACTATCTGGTTTAGTTCATCAACTCGAAGGTTGAATAAAAAAAAAGTTATCTATTCAATGTATTTAAGTTCATTCTTAGAAAACTCTAGAAAGAAAAAAATTGTGGAAAATTTATGCCTCAACGAATCACACGTAGAGATATTGATAACACACATAGAGTTAATGGTATTTCATAACTAATAGATTGGGCAGCAGCCCGTAGACCGCCTAAAAAGGAATATTTATTATTTGATCCATATCCTGACATAAGAAGTCCAATGGGAGCAATACTTGAAATGGCGATCCATAAAAAAACACCATTACTGAGATCGACTAGAATAAGTCGATAGCTAAAAGGAATTACTGAATAACTTAGTAGAATTGATATGACTGCTATGGAGGGTCCAACACTAAATAAACCCCTATCTCCTCTTGATGGAAGAAGGTTCTCTTTGAAAAGTAGTTTTGTTCCATCTGCTAGCGCTTGAAGAATTCCCAAGGGGCCAGCATATTCAGGTCCAATACGTTGTTGTATCCCTGCGGATATTTCTCTTTCTAACCACACAATTACTAGTACACCTATTGTGATTCCCAAAACAAGAATCACAATAGGTACAAGCATCCATATGGTCCCATAAACTTCTTTTAAGGATTCAAATATAGAAAAAGAATTGATAGCCTGTACTCCTGTTGTATCAATTATCATTTCAACGATCAATTTCCCCCATAATGATATCTATGCTACCTAGTATTGTCATAATATCAGCCAATTTCATTCTTTTAACTAACTGAGGAAGAATTTGCAAATTGATAAAACCCGGCGGGCGAATTTTCCATCTCCATGGAAAAGCACTCTGATCTCCTATCAAATAAATTCCCAATTCGCCCTTTGGCGCTTCGACTCTTACATAAAGTTCTTGTTTCGATAACTCATAGGCGGGAGACGGCTTTTTACTAATGAATCGATATTCAAAATTATTCCACTCAGGATCTCTTACTCTATTAAAGCGTCGGATTTCTAAATTCTCATAGGGGCCCCCCGGAATTCCTTCCAGAGCTTGCTGAATAATTTTTACAGATTCCACCATTTCGCCAATTCGGATTAAATAACGAGCTAATGAATCGCCTTCCTTCTGCCATTGGACTTCCCAATCAAATTCTTCATAACATTCATAATGATCAACTTTACGAAGGTCCCATTGTATTCCGGAAGCCCGTAGCATTGGTCCTGACAAACCCCAATTTATTGCCTCTTCTCCGCCAATAATGCCCACCCCTTCAACTCGTTCTAAAAAAATAGGATTTCGTGTAATAAGCTTTTGATATTCAGCAATTGCTGTTAAAAAATACTCACAGAAATCCAAACATTTATCTATCCAACCGTAAGGTAGATCGGCAGCGACTCCTCCGATACGAAAATAATTATGCATCATTCTCATACCAGTGGCAGCTTCGAATAGATCATATATCAGTTCTCTTTCTCTGAAAATGTAGAAGAAGGGGGTCTGTGCGCCAATATCCGCCATAAAAGGGCCAAGCCATAACAAATGAGAAGCTATACGACTCAATTCCAACATAATAACTCGGATATAGCTAGCCCTTTTAGGTACTTGAATATTTCCTAATTGTTCTGGTGCATTTATAGTTATTGCTTCTGTAAACATAGTAGCTAAATAATCCCAACGTGTTACATAAGGCAGATATTGTATAATTGTTCGGTTTTCCGCAATTTTTTCCATTCCTCTGTGCAAATAACCTAAAATTGGTTCACAGTCAATAACATCTTCGCCATCTAAAGTAACAATGAGTCGAAGAACGCCATGCATTGATGGGTGGTGAGGCCCCATATTGACTATCATTAAATCTTTTCTTGTAACTGGTCCAGTCATAAGTTTTTTCCGTATTTCTTCTTCCATGAATTGCTGAAAACGAAAAGAAGTTCATCAAAATTGAAGATCGAATAAATCAAAGAAAATAATTGTTCAAATTACCGTTTTTTTATCTCTCGAATATTCAATTGACTGATTAATTCTTTATAAAGTACTCTATTTTTTTTTGAAAAATAAGCCAGCAGTCGTTGACGTTTTCCCAAAATTTTCCGTAGACCTCTCTGAGATGAATAGTCTTTTTTGTGTAATTCCAAATGTGAGCTAAGTCTCCGTATCTTATTGGTGAAACAGAATACTTGAAATTCAACAGACCCCTTCTTTTCTTCTTTTTCTTCTTGCGAAATAACTGATATGAATGAATTTTTCGTCATAAGTATATAAATCTAAATATATATATATATTCTATGCGTCAATTTTTTTATTAATTTACTTTTTTTATTTTACGAATATGAATTTTACTGATCAGTAATAATAATGCGAGGTATTTTGATCTGGTATACACAATAATCAATCCGAATTTCCTTATTGATTCATTTTATGATCTAATTGATACGTCATTGAATTAGTATTCATGTATCAAAAAAGGATGTAAGACAAGGCATGTGCGCAGCTATTTATCCACCCGATATATATGGTAGGGGATATATAAGACAATTGTATCATCAATCAATTTTCAATCGTGGATACATATGTATCCTTAACATACTGAAACGACTACCATTATTAGTATCAAACCAATAGCGATTCATACAAGCTAAATCTTCTAATCGAGAATTGGGCCAAAGAAAGAATTTAAATTGAATTAATTTCATTTTATCTCTATCAAGATCTTTGCTTTCATCCAAAAATTGACCACAGGTTTTTACCTTGTTCCCATTGCAAAATACTGCATTTTTATCCACACAATTACTATTCCTTGAATTGAAACAACTTAGAATTCTCAATTCTCTACGCCGTCTAGACGATAAAATATTTTCAGGAACAAGCAAATCATAATGATTTTTGTTTCTATTTTTCGTCATCATTTGATGTCTTGCAATCGATTCCTCAAAATGATTCTTATCCATATTTTCTCTGTATCTTTTTTGATTATTTTTTTGTTTAATCTCATGAACCAAAGAAATCCTTATGGTTTGATACATAATAAATTCTACATTATGTTTTACAGGTATACGAACCGGTTCGATAATAAATATTCCCTCTTTTAGGATTTTTGAAAGATTCAAATCCCGGATTAGCATTGGATCCAGAGTTAACTCCTCCTTCTTAATAAAGCCGAAACCAAACTTGGTTGTCATTCTGCTTTCCTTTTCCCATTCAAGTACGGACAGCGCATAATCGAATAATTCCATAGTCAAAGGACTCAGCAGCCATTTCAATTGCAAAGCAAAAGATCCTTTCATGAACGCATCTAAGTCTATTTCCCAAGTCCAAATGCTTTTGGATTGATTTTTCGTTCTACCCATTTTCATATCTGATTTCGTATAAAGTTCTTCCATATATTTTTGTTGGTTTGAGAGAACAGATTCAGGGTTCCCTCGGTTTTGGGCATCTGATGCGAGATCTCTTTGACCTATGGGTTTTTTTTCTTCTTGATTCTCTAATTCAAAAGATTTTTTTTCTTTTTCATTTGATAGTATAAGATCCCCTTTTTGATTTTTAGTGATATTTTGATTTTGACTAACATCTTCATTAAAATGAAAAATAAGTGAATGAATTGGTATAAACCGGGGTTTCATTTTATATACATTATAAAATAACTCAAATTGTGGGAATAACCAAGGTATCGGCTTCGATACAGGACGATTTAGTCTTTCTTCATTCATTCCCATCCAATCAAAAAAAGAAAAGAAACCCTTTTGATTGGGTGGGTTGATTTCTTTATCTTTATAAATTTTGAGATAAAAAAGACCTTTCGTATCAAAATATTTTCTATCTGGATTTTTTATATACATAAAATAATCTTTTCTTATAAAATTAGTAATAGGGATACTCCCCCCCATATCAACTAATTTCGGTTTATGTATGTTGTAATTATATGAGTCCTTCTTATCTTCATAATAAATCGATTGATATGCTAAAAGATCATATCTATACATTTTTTGAAAATGATCGTTTTTATTTAGCAATAACGAAACCTTTTCCTCTCTTTCAGATGAATCCCGTTTGATTAAATTTTGATTTTCAACCCTACAATGTTGATTGACTCTATTTCGCCATTTTTGCGGTACTAATTTAGACCATTTTAGCTCAGATAAATCGTATGGATAATGACTCTTTAACCAATTTTTCCATTGATTCATTCCAGAATTCTGAAGTTTCTTATGCTTTAATTCGGAAGAAATTATTCCTTGTCTTCGAAAATAATCTTTTATTTCATTCTTAAGAAATAAAGATGCTCCGTCATATTGAAGGACAGATCTTAACTTATACAAGTTAATAACCTGGCTTTGTGATAATTTGTAAAATACATAGGCCTGTGACACGAGGGATAATTTAAAAGAAACCTCCGACTTCGTCTGAATCTTATGACGAATACGAGAAGGTGAAAGTTTTTTTTGTATAGTTGAAATACGCTCAATTATCTTTTTCTCTTTTGTATCAAAAATATATTTTTTTTTTAATTTACGAAAAAGTTTTATAATGATCATGGGCCTATAAAGACTATTAGTAAGACGATTAATGATATATGGAAAGCTCTCTAGAAGGATATCCGTGTATATCCTTTCCACGATCCATTTTATAAAATAATGTGATTTACGGATTAATCGATCATTTCTTCTTTTTAATATCTGAAAAATATTTTTTAGTGATGCTAATTTTTGAGCACCATAACTTGTTTTGTTAGGACTAATATTTATCTTTAGAGTTCGAAATCCATTTTTCTTGTCTTTTGTAATTCTTTCTATTTGATTTCTGATTGTGCTTGTTCTATCAGTCAGATCTTTCATTTTGATTTCTGTCAGTGAATAATTTGTCCAATCCATAGATCGGGTTTGAATGGATGATTCATGAATAATCTGATTATTGATTATAGAATCTTTTTTTATTTCACTCGAATCCTCTCTCAATTTTTTTGGTTCTTTTTGGACCTTTAGAAACAAAAATTTTGTTCTTTCTTTGAAACTTTTTAGAACTCGAAAACTCCATTTTATAATTGCTTTTTGGAGTTTTTTCAAAGGGGGTCCAAAATAATAACAAAACAAAATACCAAGTCCTACGAGAGGAGAACCAAAAGGACGGTCAGTTTCCAGTCCCCAAATCGTTAAAAAAGCAGCAGGACTTTCCTGCTTTGGATTTGGATCCCTACGAGAAGGTCGTATCTTAGATCGGTGCCAAGGTTTCAGACGGAAAGGAAATCGTATCATTATTTGTATACCCTCTGTGGCCCAGTTTTTAGGAAAAATTCCGTTTCTTCCTGGTTCTAGTACTGGCATACCATTATAGGTGCATATAACATACACTTCTCTATTCATCTCCCTTATATCCTGCTCCCACTCGGACTCTTGGCGTAATAAGAAACGGACAGTATTTTTAGCTAGTATCAATGAAGGTAATACAATAGATTGTCTAAGCCTCGACTGAATTAGTAAAATAAAAGCTCTTATTCCATGAGCATAAATAAGGATATCATAGAGGTCTGATATTTTTTCTCGTGTCCTTTCTATTCGTTCCATTTCCGTCTGCCCGTCCCGCCCCTTTTCCATTTCATTGACTTCTTTTTGAATTTCTTCGTAGCTTTTGTTTTCCTCCATGTACATTTTTTTTTGTTTTTTCAACCTCTTTATTCTTTTTGCTACGCTTCCTTTTATACCCTTTCTAAAAATGTCTTGTATTAGGTCGGAAATCTCAATTACTGATAATATGAATGGTTCGAAAAGAACATCCCAAGAAAATCCTACTCTGTCGAAAAAAAGTGGGGAATACGGATATAAGGGAAACATTTTCCCCGTAAGGGTTTTACGTCTTTGAACACGCATAGAACCTGTGATTATGTCTCGACGAAAATCCGCTTCATAGGGATAATCTATCATATCCACTTCTTCTATTTCATCAGGCTTCGTCATAGTTATACTAGGGTCGGCATTCTCTGCTTCCTCCGGACCCTGCGTAAAAAGAACTATACGTTTCGCTTTCCTCGAGCGAATTTGATGATCTACTATCCACTCTTCCCCCTCTTCCGTTGTTGCAGTTTTTCCGAGTTGTTCTACCTCGCTGAATAATTTGTATGACCATCGGGGGACTTTTTTATTTATTCCAATCGATTTTTTTCGAGTTGTTTTTTCCATGGGAGGAATTATGGCTGTATCGCATATTGTTTGAATGATGGGATCAATTATGACTCTTTCGATTAAAAATTTCAAAACTTTTTCTGGATCTTCTGAATCAATTCGTCTTTGTTCCGGAAATAAAGAAATTCCTTTCAAATTTGATGTTGATTCTTCAGCAAATTCATCGATTAAAAGACTCAATTCTCTTGCTAATGATTTTTTATCCAATGTATATATTTTCTGTCCCAATTTATCTTCCAATTTCTGGTCCAATTTCTGGACCAATTTCTCTTCCAATGTAGCTATTTTCCCTTCCAATTTCTGGTACAATTCTTCAAAATTATGAACATTAAGTTCCTTACCCTTAAAAAGAAGGATACTATGAACTTTATTGAGTTTATTTATAAAATTTGTCTCTATCGAATTTTTGACTGCAGTTTCATTTAGGATTGCAGGTAAATAAAATTTCTTAATTATTCCACGATAGGATCCGTTTAAGAGAGGATCATATATTTTAGGCAAGTATTCTTCTTTAGTTTTATTATTGCATAATCGAGTCTTTTTTTCGAGTACATCCAGATAAGGAGATCCTCTGTCTAGGGCTTCAATTCTATCTGTAAACTCGTTCCTTAGGCTCCTCCATTTTTTTTCATTGGTATAAATCCAACAATAATAATTATGCAGTTCATCATAGAAGAATTTATCCAGTTCATCACAGACGAATTTTTTTGTTGTAAAAAAATAAAAATACATTTTTTGTCGTAGCATTTCAAAAAAAGCTGATAAACTGGATGGATATGTAAAAGAAATTCTTCGTTTTCCATCACTTTGACATGTATAAAAAAAATATTGTGACATTTCATTTCTTACAGCATGTTCGAATCGATAATTTTTTATATATCGCAATGGACGATTCCATCGTTTATAGTCGAAAAGAAGACTCACAGGGGGTTTTTCAAACCAGAAGAGGTCTTTATCTTCTTCTTTTAAGTGAAAGTGGAATTCATCCTTTGTTTTGTCCTTTCCATTCACTCGGATCCTTTCCGTTTCATCGATTTTGTCCGGATCCTCCCTTTCTTCCGAAAAAAGGGAAGGAGAAGGATCTTCTTCGGTGAATCCCTCTTGTTCCTGTTTAGTCCCCTTCGTTTCGAAAGTTGTTTCTATTTCTACATCTCTTTCTTCCTCACTTTCCCCCCTTTCTGTCGTTTTTGAGGTTTCTTGCAGTTTCCTACTAAAAATGGGTGACGGCATTCTGCCTAAAGAGTAGACACAGCTAATAAATAAGAGAATACTAAAGATTCGATCCATAGAATCTATCAAGTCTAACACAAAGTACTTAAATTCTGACACAAGGTACTTAAATTCTGACACAGAGTACTTGTACTTCTTATACCTCTTAGATCGAATAATTCCATTAAGGTACTTATTAGATCGAATAAGTATTACTTTAAGGTACTTATTAGATAGAATAAGTACATTAAGAAGGTACTTATTAGATCGAATAGGTACATTAATAGCTCGAATAAGTACATTAAGAAGGTACGTATTAGATCGAATAATCGATCGAATAGAATAATTTTGCCGTATCCAGACTAATACCAAGCCAACACATTTCATGAATAAAATGTGACCAATTAACCAACCAACAAAACTACTTGTTACAAATAACATCTTGTTGTTGCATCGAAACATATAAATGTTGACTAATCTGGCTAACATTGAACTTGGTAAAACGAAATGGTTGAATAATTGAAAAATGAGATTATTCAGGAATACACATTGAATGCTGAGATTACGCATTGAATTTCTGGTAGTAGATCCATAATCAAAATGATTGCTCCAGAAGAAATTAAACAAAAGATAGGGTAGAGCTAGGAAAGTTATTGTATGAGGTCTACCCAATGCTAGATGCAGAGGCGTATAATAGATCGATATGAACATCATAAGTTGTCCCATAATAAAACCGGTTGTTGCTGCTACCTTCTTCTCGGTTCCTTCTTCTCCTTCTTCTCCTTCTTCCATAACCAGAGTTCGGAGAAGGACGAGATAGGAGGGCCCTATGGAGAATGTGGTCAGAAATCCATAATAGAGTCCGACCACAACGACCGAATTGACTATCTTCATGCATAAGTTACCTAGTAGAAAAAAAATCATGACAAATCCCCTTTGTGATTTTGCAATTTCTGAATTATGATTATTATATAATAAGTTTTTACCTTCTTATTATAAGATAATCTTTGACCTTATTATTTGACCTTATAAAATTAGAAAATTCTATTATTTATATTATTCTATTTATATTATTCTATTTATATTATTATTATATTAATATTATTCTATATTCTATTCTATATGATTCTATTATATATGATAAGTTTTTACCTTCTTATCTTATATGATAATTATTATAAGATAATTGTTTAACTTATTTTATTCTAGATAGAAATAGAAAGAGATAGACTAGAAACGAGATCTCTTATGTCAATGACACCAAAGGGATATTAAATGAATGGAATTGGGATATGGATGGAATATAATGAAATAGAGCCACTTTGAGGTTCCCTATGAAATGAGGCATGGAAGGGAGCCACTACGAAGAAGTTTCGGGAGTTACGAAGGAAGCTTCGAGCTCATATGGGTCATGGGTTGAGAACGGGAATTTCACTCTATGAGATCAAATCCCCCGTTGTTCCTCAGTAGCTCAGTGGTAGAGCGGTCGGCTGTTAACTGACTGGTCGTAGGTTCGAATCCTACTTGGGGAGATTTGATTCATTCTGAATTAAAGAATTCAGAATGAGAATGAAAGGGCTCGCTTTGACCGTTAAGAGTAGGTAACCCGTTCCCTGTCTTTGTTTCTATTGTATTATATCTCATCGTATGACATTCTGTTCTGCGATATTTGAGAATCTCCGTCAATACCTCGATGTAGGTCCGGGATAATCCTTTGTTCCATAGTCCTGGGGCTATTTACAACTAGCCAATTAAGAATTCTTTGATGCACTGCTAGTACTAGCAGTGCATCAAAGATGGAGTCATCGGTTCTCCCGAGAGGCTACAATTACCGCGAGCAAACATAAAACATATTAATGACGAGGAACGCTTTTTTGCTATGCTACTAATACTTGTACTTGCTCTGCTATTCTGCCCAAGCCTGGCTGAGTAAGGGTTACGGGACATAAAACAAAAAAATATGCTGCCAGCCGACGGCCGGGGTATGCTATAATTTTAAATTACCTCATTCACGATAACACGTAAAAAAAAAAGGAAGGTAAGGCCCATTCCATTTCGGCAAAAGACCCACACCCAAGTCCCATAGCTTTGGGTCCGCTATCCCGATCATGATTTTCCTATCCCCGGAGGGATAGGTCCTTCCCAAAGGGGAAGGTTGTGGGCGAGGAGGGATTCGAACCCCCGACACCGTGGTTCGTAGCCACGTGCTCTAATCCTCTGAGCTACAGGCCCCACCCCGCCTCCACTGGATCTGTTCCCGGGAGTATCCTCAAAAAAAAAAGGAACCTTTCCTCTCCCCAGCCATTTCGGGTTAAGAAGATGTGAAAGCGCGTTTCTATCTATAAGAACGGTGCGTTCCGAGGTGTGAAGTGGGAGAGAGGGGATGTCATAATTGGGGTTTTGAATAAGACGACCCTTTCTTTTTTCCTTTTTTCATTTTTTTTTCTTTCATATTGAAAAAGTAATAAGAATGAGAGGTGTTAAGCTTTTTATCATCCTGGCGTCGAGCTATTTTTCCGCAGGACCTCCCCTACAGTATCGTCACCGCAGTAGAGTTTAACCACCAAGTTCGGGATGGATTGGTGTGGTTCCTCTACGCCTAGGACACCAGAATATCGAACCACGAACTAAGAAAGGCATGAGAGAAAATTGGCTAGTGATTGTGAGGCCCCAATTCTTGACTGGAGGGGACACCAAAGGCCTCTGCCCTTCCATCCCTTGGTTAGATAGAGAGGGAGGGCAGGGCTTTTGGTTTTTTCATGTTGTCAAAGAGTTGAACAATGGTTTTTTCGTATTGTCAAAGAGTTGAACAATGAAAATAGATGGCGAGTGCCTGATCGAATTGATCGGGTCATGTAGGAACAAGGTTCAAGTCTATCGGTCTGTTAGGATGCCTCAGCTGCATACATCACTGCACTTCCACTTGACACCTATCGTAATGATAAACGGCTCGTCTCGCCGTGACCTTCTCTTGAATTCTCAAAACTTCTGTCGCTCCATCCCCGCAGGGGCAGAGAACCCGTCGCTGTCTCGGCTGTGCTACCGGAGGCTCTGGGGAAGTCGGAATAGGAGAGCACTCATCTTGAGGTGGGCTTACTACTTAGATGCTTTCAGCAGTTATCCGCTCCGCACTTGGCTACCCAGCGTTTACCGTGGGCACGATAACTGGTACACCAGAGGTGCGTCCTTCCCGGTCCTCTCGTACTAGGGAAAGGTCCTCTCAATGCTCTAACGCCCACACCGGATATGGACCGAACTGTCTCACGACGTTCTGAACCCAGCTCACGTACCGCTTTAATGGGCGAACAGCCCAACCCTTGGAACATACTACAGCTCCAGGTGGCGAAGAGCCGACATCGAGGTGCCAAACCTTCCCGTCGATGTGATCTCTTGGGGAAGATCAGCCTGTTATCCCTAGAGTAACTTTTATCCGTTGAGCGACGGCCCTTCCACTCGGCGCCGTCGGATCACTAAGGCCGACTTTCGTCCCTGCTCGACGGGTGGGTCTTGCAGTCAAGCTCCCTTCTGCCTTTGCACTCGAGGGCCAATCTCCGTCCGGCCCGAGGAAACCTTTGCACGCCTCCGTTACCTTTTGGGAGGCCTACGCCCCATAGAAACTGTCTACCTGAGACTGTCCCTTGGCCCGTGGGTCCTGACACAAGGTTAGAATTCTAGCTCTTCCAGAGTGGTATCTCACTGACGGCTCGGGCCCCCCCGGAAGGGGGCCTTCTTCGCCCTCCACCTAAGCTGCGCAGGAAAGGCCCAAAGCCAATCCCAGGGAACAGTGAAGCTTCATAGGGTCTTTCTGTCCAGGTGCAGGTAGTCCGCATCTTCACAGACATGTCTATTTCACCGAGCCTCTCTCCGAGACAGTGCCCAGATCGTTACGCCTTTCGTGCGGGTCGGAACTTACCCGACAAGGAATTTCGCTACCTTAGGACCGTTATAGTTACGGCCGCCGTTCACCGGGGCTTCGGTCGCCGGCTCCCCTGTCATCAGGTCACCGACTTCCTTGACCTTCCGGCACTGGGCAGGCGTCAGCCCCCATACATGGTCTTACGACTTTGCGGAGACCTGTGTTTTTGGTAAACAGTCGCCCGGGCCTGGTCACTTCGACCCCCTTTGTGAGGAGGCACCCCTTCTCCCGAAGTTACGGGGCTATTTTGCCGAGTTCCTTAGAGAGAGTTGTCTCGCGCCCCTAGGTATTCTCTACCTACCCACCTGTGTCGGTTTCGGGTACAGGTACCCCTTTGTTGAAGGTCGTTCGAGCTTTTCCTGGGAGTATGGCATGGGTTACTTCAGCGCCGTAGCGCCTGGTACTCGAATATTGGCTCGAGGCATTTTCTCTACCCCTTCTTACCCTGAAAAAGCAGGGTCACCTTGCATCCTTGAACCGATAACCATCTTTCGGCTAACCTAGCCTCCTCCGTCCCTCGGGACCAACAAGGGGTAGTACAGGAATATTCACCTGTTGTCCATCGACTACGCCTTTCGGCCTGATCTTAGGCCCTGACTCACCCTCCGTGGACGAACCTTGCGGAGGAACCCTTAGGTTTTCGGGGCATTGGATTCTCACCAATGTTTTCGTTACTCAAGCCGACATTCTCGCTTCCGCTTCGTCCACCGCGGCTCGCGCCGTTGCTTCCTCCTAAGGCGGAACGCTCCCCTACCGATGCATTTTTACATCCCACAGCTTCGGCAGATCGCTTAGCCCCGTTCATCTTCGGCGCAAGAGCGCTCGATCAGTGAGCTATTACGCACTCTTTCAAGGGTGGCTGCTTCTAGGCAAACCTCCTGGCTGTCTCTGCACCCCTACCTCCTTTATCACTGAGCGGTCATTTAGGGGCCTTAGCTGGTGATCCGGGCTGTTTCCCTCTCGACGATGAAGCTTATCCCCCATCGTCTCACTGGCCGACCTTGACCCCTGTTATTTGGAGGTCATATCTAGTATTCAGAGTTTGCCTCGATTTGGTACCGCTCTCGCGGCCCGCACCGAAACAGTGCTTTACCCCTAGATGTCCAGTCAACTGCTGCGCCTCAACGCATTTCGGGGAGAACCAGCTAGCTCTGGGTTCGAGTGGCATTTCACCCCTAACCACAACTCATCCGCTGATTCTTCAACATCAGTCGGTTCGGACCTCCACTTAGTTTCACCCAAGCTTCATCCTGGTCATGGATAGATCACCCAGGTTCGGGTCCATAAGCAGTGACAATTGCCCTATGAAGACTCGCTTTCGCTACGGCTCCGGTGGGTTCCCTTAACCAAGCCACTGCCTATGAGTCGCCGGCTCATTCTTCAACAGGCACGCGGTCAGAGCCCTATGTGCTCCTCCCACTGCTTGGGAGCTTACGGTTTCATGTTCTATTTCACTCCCCGATGGGGGTTCTTTTCACCCTTCCCTCACGGTACTACTTCGCTATCGGTCACCCAGGAGTATTTAGCCTTGCAAGGTGGTCCTTGCTGATTCACACGGGATTCCACGTGCCCCATGCTACTCGGGTCAGAGCGTAAGCTAGTGATGCTTTCGGCTACTGGACTCTAGCCATCTAGGGTGCGGCACTCCACCGCTTCGCCTAGCAGCACGACGCTTGTATTGCTCTCCCACAACCCCGTTTTCACGGTTTAGGCTGCTCCCATTTCGCTCGCCGCTACTACGGGAATCGCTTTTGCTTTCTTTTCCTCTGGCTACTAAGATGTTTCAGTTCGCCAGGTTGTCTCTTGCCTGCCCATGGATTCAGCAGCAGTTCGAAAGGTTGACCTATTCGGGAATCTCCGGATCTATGCTTATTTGCAACTCCCCGAAGCATTTCGTCGCTTACTACGCCCTTCCTCGTCTCTGGGTGCCTAGGTATCCACCGTAAGCCTTTCCTCGTTTGAACCTCGCCCTTAACTTTAAGGCTATGCCATCCTAAGGTGCTGCTAAGCTAAATGGAAGGATCTTATCAACGTCCATGAATGAGAAATCATAGATCGAACCGCCGAATCGGAAAATTTGGGTGCTATCATATAGCTTTGTATCGGCTAAGTTCACGAGTTGGAGATAAGCGGACTCGAACCGCTGACATCCGCCACAGGGTAAACCACCGTCTCTCAGGCCCCCCGACTGATTCTACCATAGAGGCTGACGATAGACAATAACTCCCCCCCGAACACAGCTTACAACTTTCATCGTACTGTGCTCTCCAAAGAGCAACTCTTCTCAAAATCTCAAAAGGTGCTGAGTTGGAATCCAATTCGAACTAAGGATTCTTGTGGTTCCGGAGGATCCAGCTACAGGAGAACCAGGAACGGAGAGCTTTCCCCCCTTTTCCGCCCAACTCTTTGGTCTTAAGAATGCTGGTTTTAAGAATGAGTGATTGCCCTTCTCCGACCCTTACTGCCCAACCGGAGAGCGGACAGCTAATGCGTTCCACTTATTGAACAGGGTTCTATGGTCGGCCCGTGACCCCTGGATGCCGAAGGCGTCCTTGGGGTGATCTCGTAGTTCCTACGGGGTGGAGACGATGGGGTCGGTCCATGGATTTTCCTTCCTTTTGCCGCATTTCGCTCAAAGGGTTGAAGGGAGATAGTGCATCAGGCTGTTCGCAAGGGCCAACTTGATCCTCTTCCCCAGGGATCCCAGATTAAGGAACCCTAGGAGAGCCGCTGACTCCAACTACGGTCCATGTACGATCCATACTAGATCTGACCAACTGCCCATCCTACCTCCTCTACGTTCTTGACAGCCCATCTTTGTCTCAGTAGAGTCTTTCAGTGGCATGTTTCGGTCCTCTTCCCCATTACTTAGAAAAAGTGAGCCACCGGTTCAGGTACAAGATACTATCATTACCGCCTGGACAATTAGACATCCAACCCGTAATCGCAACGACCCAATTGCAAGAGCGGAGCTCTACCAACTGAGCTATATCCCCCCGAGCCAGGTGGAGCATGCATGAAGGAGTCAGATGCTTCTTCTATTCTTTTCCCTGGCGCAGCTGGGCCATCCTGGACTTGAACCAGAGACCTCGCCCGTGAAGTAAATCATCGCACCTACGGTCCAACCAATTTGGAGAGAATCAATAGATTCCTTTTCGGGAGCGATTCATCCTTCCCGAACGCAGCATACAACTCTCCGTTGTACTGCGCTCTCCAAGTGTGCTTGTTCGCTCCTTCTTCCTTCTTACCATGGCAAGTCTTTGTGAAATAACGTGAAATAACCCCGATGGGAAGAAAAAAGAAGGCGTTAAGAGACCCTCCTGGCCCAACCCTAGACACTCTAAGATCCTTTTTCAAACCTGCTCCCATTTCGAGTCAAGAGATAGATAAATAGACACATCCCATTGCACTGATCGGGGGGCGTTCGTAGTGACTGAGGGGGTCGAAGACCAAGAAGTTAGTTATTTATACCAAGCATTCTTCTTACGGCTAGATCCAATCTCCTGGTCCCTGCGGAAAGGAAAAAGAATTTCACGTTCTTCCTTTCGGGAAGGGAGGATTAGGGAAATCCTATTGATTGCAGCTTTCTCCAGACCTCCGGGAAAGCATGAAAAAAAAAAGGCTCGAATGGTACGATCCTTCCGTCACCCCAGAATGAAGAGGGTGATCTCGTAGTTCTTGGTCTGTGAAGATGCGTTGTTAGGTGCTCCATTTTTTCCCATTGAGGCCGAACCTAAACCTGTGCTCGAGAGATAGCTGTCCATACACTGATAAGGGATGTATGGATTCTCGAGAAGAGAGGAGCCGTAGTGGTCCCCCCCGGACCGCCCGGATCCCACGAGTGAATCGAAAGTTGGATCTACATTGGATCTCACCTGAATCGCCCCATCTATCCTCCTGAGGAGAAGTTTGGTTTCAAACCCCGGTTCGAACAGGAGGAGTACGCCATGCTAATGTGCCTTGGATGATCCACATCTCAAGGTCAGGCGCGGATGGGCACATTGAACTATCCATGTGGTTGAGAGCCCTCACAGCCCAGGCACAACGACGCAATTATCAGGGGCGCGCTCTACCGCTGAGCTAATAGCCCGTCGTGCGGGCCCCCAGTGGGAGGCCCGCTATGCCAAAAGCGAGAGAAACCCCATCCCTCTCTTTCTTTTTTACGTCCCCATGTCCCCCGTGTGGCGACATGGGGGCGAAAAAGGGGGGATCCTATCAACTTGTTCCGACCTAGGATAATAAGCCCATGAGCTTGGTCTTACTTCACCGTCGAGAAACGAAAGAAGACTTCCATCTCCAAGTTTCACCCAGGCGTCGCTCGCTTCTTTTTGGGTGTGAAGCAGTGTCAAACCAAAATACCCAACAAGCATTAGCTCTCCCTGAAAAGGAGGTGATCCAGCCGCACCTTCCAGTACGGCTACCTTGTTACGACTTCACTCCAGTCACTAGCCCTGCCTTCGGCATCCCCCTCCTTGCGGTTAAGGTAACGACTTCGGGCATGGCCAGCTCCCATAGTGTGACGGGCGGTGTGTACAAGGCCCGGGAACGAATTCACCGCCGTATGGCTGACCGGCGATTACTAGCGATTCCGGCTTCATGCAGGCGAGTTGCAGCCTGCAATCCGAACTGAGGACGGGTTTTTGGGGTTAGCTCACCCTCGCGGGATCGCGACCCTTTGTCCCGGCCATTGTAGCACGTGTGTCGCCCAGGGCATAAAGGGCATGATGACTTGACGTCATCCTCACCTTCCTCCGGCTTATCACCGGCAGTCTGTTCAGGGTTCCAAACTCAACGTTGGCAACTAAACACGAGGGTTGCGCTCGTTGCGGGACTTAACCCAACACCTTACGGCACGAGCTGACGACAGCCATGCACCACCTGTGTCCGCGTTCCCGAAGGCACCCCTCCCTTTCAAGAGGATTCGCGGCATGTCAAGCCCTGGTAAGGTTCTTCGCTTTGCATCGAATTAAACCACATGCTCCACCGCTTGTGCGGGCCCCCGTCAATTCCTTTGAGTTTCATTCTTGCGAACGTACTCCCCAGGCGGGATACTTAACGCGTTAGCTACAGCACTGCACGGGTCGATACGCACAGCGCCTAGTATCCATCGTTTACGGCTAGGACTACTGGGGTATCTAATCCCATTCGCTCCCCTAGCTTTCGTCTCTCAGTGTCAGTGTCGGCCCAGCAGAGTGCTTTCGCCGTTGGTGTTCTTTCCGATCTCTACACATTTCACCGCTCCACCGGAAATTCCCTCTGCCCCTACCGTACTCCAGCTTGGTAGTTTCCACCGCCTGTCCAGGGTTGAGCCCTGGGATTTGACGGCGGACTTAAAAAGCCACCTACAGACGCTTTACGCCCAATCATTCCGGATAACGCTTGCATCCTCTGTCTTACCGCGGCTGCTGGCACAGAGTTAGCCGATGCTTATTCCCCAGATACCGTCATTGCTTCTTCTCCGGGAAAAGAAGTTCACGACCCGTGGGCCTTCTACCTCCACGCGGCATTGCTCCGTCAGGCTTTCGCCCATTGCGGAAAATTCCCCACTGCTGCCTCCCGTAGGAGTCTGGGCCGTGTCTCAGTCCCAGTGTGGCTGATCATCCTCTCGGACCAGCTACTGATCATCGCCTTGGTAAGCTATTGCCTCACCAACTAGCTAATCAGACGCGAGCCCCTCCTCGGGCGGATTCCTCCTTTTGCTCCTCAGCCTACGGGGTATTAGCAGCCGTTTCCAGCTGTTGTTCCCCTCCCAAGGGCAGGTTCTTACGCGTTACTCACCCGTCCGCCACTGGAAACAACATTTCCCGTCCGACTTGCATGTGTTAAGCATGCCGCCAGCGTTCATCCTGAGCCAGGATCGAACTCTCCATGAGATTCATAGTTGCATTACTTATAGCTTCCTTGTTCGTAGACAAAGCAGATTCGGAATTGTCTTTCATTCCAAGGCATAACTTGTATCCATGCGCTTCCTATTCGCATGGAGTTCGCTCCCAGAAATATAGCCATCCCTAGCCCCTCGCGTCAATCCCACGAGCCTCTTATCCATTCTCTTTCAATCACGGCGGGGGAGCAAGTCAAAATAGAAAAACTCACATTGGGTTTAGGGATAATCAGGCTCGAACTGATGACTTCCACCACGTCAAGGTGACACTCTACCGCTGAGTTATATCCCTTCCCTGCCCCCATCGAGAAATAGAACTGACTAATCCTAAGGCAAGGGGTCGAGAAACTCAACGCCACTATTCTACTATTCTTGAACAACTTGGAGCCGGGCCTTCTTTTCGCACTATTACGGATACGAAAAGAATGGAAAAATTTGGATTCAATTGTCAACTGCTCCTATCGGAAATCGGATTGACTACGGATTCGAACCATAGCACATGGTTTCATAAAACCGTACGATTTTCCCGATCTAAATCAAGCGGGTTTTACATGAAGAAGATTTGGCTCAGCATGTTCTATTCGATATAGGTAGGAGAAGAACGCGACTCGGTATTAAAAAAAAATAGAGGAAGCAGAACCAAGTCAAGATGATACGGATCAACCCCTTCTTCTTGCGCCAAAGATCTTACCATTTCCAAAGGAACTGGAGTTCCATCTCTTTTCCATTTCCATTCCAGAGTTCTTATGTGTTTACACGCCCCTTTGAGACCCCGAAAAATGGACAAATTCCTTTTCTTAGGAACACATACAGGATTCGTCACTCCAAAATGGATAATGGTAACCCCACCATTAACTACTTCATTTATGAATTTCATAGTAATAGAAATACATGTCCTACCGAGACAGAATTTGTAACTTGCTATCCTCTTGACTAGCAGGCAAAGATTTACCTACGTGGAAAGGATGATTCATTCGGATCGACATGAGAGTCCAACTACGTTGCATTGCCAGAATCCATGTTGTATATTTGAAAGGGGTTGACCTCCTTGCTTCTCTCATCGTACAATCCTCTTCCCGACGAGCCCCCTTTCCCCTCGGTCCACAGAGACAAAATGTAGGACTGGTGCCAACAGTTCATCACGGAAGAAAGGACTCACTGAGCCGGGATCACTAACCAATACTAATCTAATAGAAAATACTAATATAATAGAAAAGCACTGTCTTTTCTGTAGACTTTCCCCGGTTCTGTTGCTACCGCGGGCTTTACGCAATCGATCGGATCATATAGATATCCCTTCAACACAACATAGGTCATCGAAAGGATCTCGGAGACCCACCAAAGCACGAAAGCCAGGATCTTTCAGAAAATGGATTCCTATTCGAAGAGTGCATAACCGCATGGATAAGCTCACACTAACCCGTCAATTTGGGATCCAATTCGGGATTTTCCTTGGGAGGTTTCGGGAAGGAATTGGATGGAATGTAATAATATCGATTCATACAGAAAGTTCTCTATTGATTTAAACGCTGTACCTATGGGATAGGAATAGAGGAAGAGGAAAAGCCGAAGATTTCACATAGTACTTTTGATCGAAAAATGAAAATCAATCTGATTTATTTCGTATCCCTCGCTCAATGAGAAAATGGGTCAGATTCTACAGGATCAAACCTATGAGACTTAAGGAATTATGGAAGGGAATAAAAAAAGAGAGGGAAAAAATAGAAAAAAAGTAAAAAGTAGAAGAACCCAGATTCCAAATGAAAACAAATTCAAACTTGAAAAGGATCTTTCTAATTCTCGAAGAATGAGGGGCAAGGGGATTGATCGAGAAAGATCTCTTGTTCTTATTAGAAGATCGTGATTGGATCCGCATATGTTTGGTAAAAAGAATAATCTTCTCCTTTGAGAATAATCAAAAGTGGAACGTGTTCAATTGGAACATGAAAACATGACTGAATTGGTCCTAGTTACTCTTCGGGACGGAGTGGAAGAAGGGAGGAGATTCTCGAACGAGGGAAGGGATCCAATGACTTCGAAAGAATTGAACGAGGAGCCGTATGAGGTGAAAATCTCATGTACGGTTCTGTAGAGTGGCAGTAAGGGTGACTTATCTGTCAACTTTTCCACTATCACACCCAAAAAACCAAACTCTGCCTTACGTAAAGTTGCCAGAGTACGATTAACCTCTGGATTTGAAATCACTGCTTATATACCCGGTATTGGCCATAATTTACAAGAACATTCTGTAGTCTTAGTAAGAGGGGGAAGGGTTAAGGATTTACCCGGTGTGAGATATCACATTGTTCGAGGAACCCTAGATGCTGTCGGAGTAAAGGATCGTCAACAAGGGCGTTCTAGTGCGTTGTAGATTCTTATCCAAGACTTGTATCATTTGATGATGCCATGTGAATCGCTAGAAACATGTGAAGTGTATGGCTAACCCAATAACGAAAGTTTCGTAAGGGGACTGGAGCAGGCTACCATGAGACAAAAGATCTTCTTTCTAAAGAGATTCGATTCGGAACTATTATATGTCCAAGGTCCATAATTTAAGAGGTTTTCCCTGACTTTGTCCGTGTAAACAAACAATTCGAAATACCTCGACTTTTTTAGAACAGGTCTGAGTCAAATAGCAATGATTCGAAGCACTTCTTTTTACACTATTTCGGAAACCCAAGGACTCAATCGTATGGATATGGAAAATACAGGATTTCCAATCCTAGCAGGAAAAGGAGGGAAACGGATACTCAATTTAAAGTGAGTAAACAGAATTCCATACTCGATCTCATAGATACATATAGAATTCTGTGGAAAGCCGTATTCGATGAAAGTCGTATGTACGGTTTGGGGGGAGATCTTTCATATCTTTCGAGATCCACCCTACAATATGGGGTCAAAAAGCCAAAATAAGTGATTTTAGCCCTTATAAAAAGAAAACTGATTCTTGAACCCCTTTCACGCTCATGTCACGTCGAGGTACTGCAGAAGAAAAAACTGCAAAATCCGATCCAATTTATCGTAATCGATTAGTTAACATGTTGGTTAACCGTATTCTGAAACACGGAAAAAAATCATTGGCTTATCAAATTATCTATCGAGCCGTGAAAAAGATTCAACAAGAGACAGAAACAAATCCACTATCTGTTTTACGTCAAGCAATACGTGGAGTAACTCCCGATATAGCAGTAAAAGCAAGACGTGTAAGCGGATCGACTCATCAAGTTCCCATTGAAATAGGATCCACACAAGGAAAAGCACTTGCCATTCGTTGGTTATTAGGGGAATCCCGAAAACGTCCGGGTCGAAATATGGCTTTCAAATTAAGTTCCGAATTAGTGGATGCTGCCAAAGGGAGTGGCGGTGCCATACGCAAAAAGGAAGAGACTCATAGAATGGCAGAGGCAAATAGAGCTTTTGCACATTTTCGTTAATCCATGAACAGGATCTATATAGTAGACGCCTAGATCCATGGATCCATACATCTCGATCGGAAAAGAATCAATAGAAAAAGAAAGAATCGGAATTGATCGATTATTTCGAAACAAACGAAAAGGAAACGAAAGATGAAAGAGAAATCATGGATCAACTAAGCCCTCTCGGGGACTTGCTTAAGAATAAATAAGCAAGAGGAATCTCATGGAAATACCATGGAATAGGGTTGGATCCTATTCGTGGAGATTCCGTAAATATTCCATTTCAAAAATAGAAAGTTCTAAACAATTGGGATTTTTTTGGAGATTGGATGCAGTTACTAATTCATGATCTGGCATGTACAGAATGAAAACTTCATTCTCGATTCTACGAGAATTTTTATGAAAGCCTTTCATTTGCTTCTCTTCGATGGAAGTTTTATTTTCCCAGAATGTATCCTAATTTTTGGCCTAATTCTTCTTCTGATGATCGATTCAACCTCTGATCAAAAAGATATACCTTGGTTCTATTTCATCTCTTCAACAAGTTTAGTAATGAGCATAACGGCCCTGTTGTTCCGATGGAGAGAAGAACCTATGATTAGCTTTTCGGGAAATTTCCAAACGAACAATTTCAACGAAATCTTTCAATTTCTTATTTTACTATGTTCAACTCTATGTATTCCTCTATCCGTAGAGTACATTGAATGTACAGAAATGGCTATAACAGAGTTTCTGTTATTCGTATTAACAGCTACTCTAGGAGGAATGTTTTTATGCGGTGCTAACGATTTAATAACTATCTTTGTAGCTCCAGAATGTTTCAGTTTATGCTCCTACCTATTATCTGGATATACCAAGAAAGATGTACGGTCTAATGAGGCTACTACGAAATATTTACTCATGGGTGGGGCAAGCTCCTCTATTCTGGTTCATGGTTTCTCTTGGCTATATGGTTCATCCGGGGGAGAGATCGAGCTTCAAGAAATAGTGAATGGTCTTATCAATACACAAATGTATAACTCCCCAGGAATTTCAATTGCGCTTATATTCATCACTGTAGGAATTGGGTTCAAGCTTTCCCCAGCCCCTTCTCATCAATGGACTCCTGACGTATACGAAGGAGTGCGGTTCGTTCGAAAAATTCCTAGCTCTCTATCTATCTCCGAGATGTTTGGATTTTTCAAAACTCCATGGACATGCAGAAGAGAAATGCTATCCCCACTCGGACCAAGACATAACTTTTACTTGTTCAAATAACAATTAAGGTGAAGCAGGGTCAGGAACAACGAATCTCTTTATGATAAACAGATCCATTTTGCAAGTTCGTTATTACGGGTAGTTCCTACAAAGGATCGGACTAATGACGTATACAATACTTGAATTCTCGATGTAGATGCTACATAGTTGGTTCTCATCCTTCAGAGACTACGAGTGTAATAGGAGCATCCGTCGACAAAAGGATCACCCTAAGATGATCATCTCATGGCTATTGAGAACGAATCAAATCAGATGGTTCTATTTCTCAATCTTTCTGACTTGCTCCTACGGAACCAAGGTCGAAAAGATTGAAAAAATAAGTCATTCGCAACCACTGATGAAGGATTCCTCGAAAAGTTAAGGATTAGTAATCCTTTTTAGAAATCGAACGGATTCGGTCTTATACATACGCGAGGAAGGTAATCAAAAAAGAAAGAAGATGAGTTCTTCTTTCTTTTATCACTTAGGAGCCGTGCGAGATGAAAGTCTCATGCACGGTTTTGAATGAGAGAAAGAAGTGAGGAATCCTCTTTTCGACTCTGACTCTCCCACTCCAGTCGTTGCTTTTCTTTCTGTTACTTCGAAAGTAGCTGCTTCAGCTTCAGCCACTCGAATTTTCGATATTCCTTTTTATTTCTCATCAAACGAATGGCATCTTCTTCTGGAAATCCTAGCTATTCTTAGCATGATAGTGGGGAATCTCATTGCTATTACTCAAACAAGCATGAAACGTATGCTTGCATATTCGTCCATAGGTCAAATCGGATATGTAATTATTGGAATAATTGTTGGAGACTCAAATGATGGATATGCAAGCATGATAACTTATATGCTGTTCTATATCTCCATGAATCTAGGAACTTTTGCTTGCATTGTATTATTTGGTCTACGTACCGGAACTGATAACATTCGAGATTATGCAGGATTATACACGAAAGATCCCCCTTTGGCTCTCTCTTTAGCACTATGTCTCTTATCCCTAGGAGGTCTTCCTCCACTAGCAGGTTTTTTCGGAAAACTCCATTTATTCTGGTGTGGATGGCAGGCAGGCCTATATTTCTTGGTTTCAATAGGACTCCTTACGAGCGTTGTTTCTATCTACTATTATCTAAAAATAATCAAGTTATTAATGACTGGACGAAACCAAGAAATAACCCCTCACGTGCGAAATTATAGAAGATCTCCTTTAAGATCAAAGAATTCCATCGAATTGAGTATGATTGTATGTGTGATAGCATCTACTATACCAGGAATATCAATGAACCCTATTATTGCAATTGCTCAGGATACCCTTTTTTAGCTTCTAGAGTCTATTTCATCCCTCTTACTAACTGGAATCAAAGAATTAGTAGATCTGTTCCGCCCAAAATGGGAATGGGCTAGGGTTATGAACTTATAATCTATAATCTGATGATCGAGTCGATTCCATGATTATAAGTTCATTCCATACCGGACTAGGCCGGAAGAGGGTTATATAAATTCTCATTATGAGAAGGGGTCATTCGAGCCTATCTAAATAGATACTCTATTTCCATATGGATCCCTACGTCTTTACATTCCATTTCGGATTAGGAATAGGCGTAATCGGACCTTCTTTTTACATATCTCTCGTTATTTGATTTGGGACCCTATTCATCTCTTTGGGCTTCTATTGAATCGAGAAATAGGTTTGATTGTCCATCTTTTTGATATATATAAGTATAAGGCATCCTCCGGAAAATGAAAATCGAAGCAATTGGATGTCCGACTCGGGCCTATATGACATGACCGATCAATAGCAATACTCCAACACTCCACCTTTGTCATATATTCCATACATCACACTAGATAGATATCATATTCATGGAATACGAATCACTTTCAAGATGCCTTGGTGGTGAAATGGTAGACACGCGAGACTCAAAATCTCGTGCTAAAGAGCGTGGAGGTTCGAGTCCTCTTCAAGGCATAGAGAATGCTCATTGAATGAGCAATTCAATAAGAGATCTTGGAATTGGAATAAGTTCGGCAGCGGATCACGAAATCTTGGTGATCTTCTCTATCTAATGAGGGGGGAGTCCGCTTTGAAATCGTCCGCCCTGAACCCCCCCGAGTATATGCTTCAACAGGAATCACACAAGGGTAGATTGATACAATAGAAACCTCTGGTAAAATGCCCGCCCGTAACGTAACTCAGCAGAGAAATTACATAGTCCGTTTTAGGGATTGGCGACTTACCCATTAGTGACTTTGGCACTGGACGTTCCCCCCCAAAATGGGTACTATCGGGTCGGGTGAATTCAAGAATAGACGCCTGTTGGCATTCCAGCCTCCCTTCTCCTTTCGGGGCCTATCCGAAAGAGAATCCAGTACTTCTTGGTCGTGAATATCTGAATGAATAGGACGAACGACCCCGTGGATACCTTTGCTTCGGAACAAAACAATTCGGTCAACTGGAATGTGTATTATCCCTATAGGGTATTTTATTTAGTTATTCAGTCAAACCAATGATTCGTTATTGGAGCAGATAGCAACTTTCATCAGACATGCGTATTTTTGATTTTACAATGGATTTCCATCTTTCATTAATGGAAATTTTTTTATGTAGTGAGTAATAGGCTCTGGTTGTTCGCTGTTCAAGAATTCTTGTTTAGGCCGTTCATACCATCCATACATAGTGTTTTGATCTAAGATTTCAATTCTTCCATCTTTCAGCAGTAGCATATTGTTCCATGGAGCTAAGGTCCGAAATATGGAAAAAACAAGTATTTCCACGACTCTACCGCCCAGTCAATTCTGTTCCACTTAATCCCTCTTTCATGGCCACATATCTTTACGGCTAAGGAATGGGAAATCTTTCTCCTGTTACATGAATCCAATTTTCAGTTCATCCGGGAAAATCCATCTTTTTCTAAACAATGTCTTTGTCATTTGATCCAATAGCCTTCCGTTAGATAGGAACAGATTTGATAAGTACTGATAACTCGCGGATTGAATATTAGAACGGAAAGATCTATTATATAATGAACTAATGGTTCTAAGCCGTCTCCGTCTCTGGCGATTAATAAAAAATTCGAAGTACTTTTCTTTCGGTTTCTTGCTAAACCCGCGTTTATATAGAGTCTCCCTTTGGGAAGTCAGAAGAAGCCCCTTTGACATCTCTTCATCTGCAAAGAATTCTCGATGTGAAAACAGAAAGACAGAGAGCTCATCGTTGAATATGTAAAAGAGTGGATCTCCAGGGTCCCAAATGAATTGGCCTTTTCGAAAAAAGACTTGTTCTTTGGAAGATCTATCTCGTCCCGGGTACTCCATGGTTTCACTCTGCAAGAACTCCCAATCATTCTCTTGAAGCTCATCCTCTTCATCATATCCATCATCCCCTTCACCATAAAATGCATAATCAAAATATTCATCATTAACTTCATCAGAAATGATCGGCTTGCCCCGAAATGACCTGGCCCAATAGGGAAATTCCAATTCATTGGGCCTTTCGATCCAATCAAATAGAAAGCCCCAAGGTTGCCATATTCTAGGAGCCCAAACTATGTGATCGACTACATCCTCCGCTAACTGTTGCGGGTCGGTGCCTTCTGCCCATTCTTTAAACTCCGATTCATAGAGAAATCTACGATCAAAGATAGAACGAGATCCATTTTCCATCATCTCTAAGGGATTCCTTGGTTCGGGCCGAAGAAGCGAGGATCCCACCAGAGCGCCTTCTACTACTTCTAATAGGCCATCAACTAGATCAGAATCAGTCTCAACGAGTCTATAAGAAGTGATCCAATTTTTTTCATCGGGTCCGGGTAGAGACCAAAGATCTTGAGCGACCGATCCGGCAGAACAACTCAAAAGATAAAGAAGTATCGTTAATTTCTTCATGCTCGTTCCAAGTTCGAAGAACCATTTGTACAAATAAGGATCCCCTTCGTAACATGATTGCTTCTTCATATAGATAGATATAGGATCTATAAGGCAGCAATTATTTCTAAGTACATTTTGTGCAACAGCCCTTCCTATCTGATAGAAAAGGATCCCATGATCCGGAACCGGTCTTACATGGGCTCGCAACTCCCAAGTTTGTCTATGAAGAGCGAATCTAATTGTATTAGTGTCTATAATTGATTTCTTCTGTGTAATACTAATCGATAGGGCCTCATTGGTAATTGCTACAAGATCTCGTGCATTGTAACCCATGGCTATGGACCCGAATCCATTAGTATGGAACATTTTCTTTTCCAAGTGAAATCCCCTAGTATATGAAAGAGTGAAAACGTGCTTTCGTTGTTGTGGAATAAGAAGCCTTCGTATCTTAATGCATGTATTTAATTTATTCGGAGCTATTAGAGCGGGATCCACTTTTTGGGGAATATGAGTCGAAGCAATAACAAGAATATCTCTAGTGGACCATCTTTCACAATCCCCGGAGAGATAGTTCAATAATAAACCGAGGGACTCCGACTCATCCACATACAGATCATGAATGTTTGGAATCCATACTATGCAAGGAGACATTGTTCTTGCCAATTCGAATTGCAAGTTGATAGAAGCTAGGTCTATTTCCAACTCGATGATATACCTAAGTATCTCATCCACCGTATACTCCTCCCTCAGCTCCGGGTCCGAGTCCAAGTTCGAATAAATATAGTCACGATCATCAATATCGTCCACATCTTTCAGCGCATCCATATATTCCTTAGCAGGATCGCTATCATCAATACCATCAATATCCACATCATCAAGCGCTTCCATATAGTCCTCAGGATCGAGATCATCAATAACTATTTTCAAATCCAGCTTGTTCAGAAATACCGTAATGAAAGGAAGATAGGAGTTTGTCGCTAGGGATTTGACCAAATAGGATCGTCCAGTTCCTATAGGACCTATCACTAAAATACCCCTAGAGGGGGATAGTGCTAGGCGGAACGAAAAGGGTTTTGGTTTTCCATGAGATGGGAAATGAAAACTATTAGCCCCACACGAGGTTTGTGAATAAGTGATTGTCTGATAATGAGCAAGGAATATCCGTCTTTCTGCTAAACAGGATGTATTGAACTCATAATTCATTAGATCCTTTTGATGAATGTCAACTAAGTATCGTAAGTAAATTGCTCCCGCTTGTTCAAACATTTGATAACCATAGTCACTCTTTAATAAATGATCAATATGAGTCAGACTCAATAGAATTTGATCAATCCCTTTTTCTGGCCTTAAGGTGGAGAAATGAAACGAGTAAACTCTCTCTTCATCCAAAAACCAATCACAGGTCTCGATCCAGGATTCTATTTCATCATGAGTCTGAAACCTTTGTCCTTTTCTTATCCATGAATAGATCTCTTTACTTGTATGACTTAGATGTCTCGTATTTCTCGAAAAAGTGATTCGATTGATGGGATTTGGTATGATACTTATGAGATCGATGAGATTGAAAAATATCTTCTGTATAAATTTGACCCCATAAGCGGGACCACCACCAAATAGCGCAAAACCCAGTATTTCTGCTAGAAAATCTTCTAATTGTTCCCGAGCAACTAGAAAGAGATTCTTTAACCAGAAAGAATTCGGTTCAGGTTTAGGATACCCATCCACAAGTTTGTACACCTCAATCGTGTATGATGGAATCGTCAAAGATTTTATCCTCTCGAACTCTGTCTGTAACTCACTAGAGTCTAGGGAAACAGAGAAAAGAAGTACCTGAACGAGACATCCAGCAAGAAGAAGAAGTAAAAGGCTTGAATAGAGGAACTCCCGAACATTTGGCGAGCTCAGATGTGTCGATATCAATGGTGACTCATTATTTCGATGAATCATTTCTTCGACCCGAAGAAGCCTACGGAAACACTTCCACGAAATATCACTTGAAATCTCACTTATCGGTGCCCACAATCCCCACAATTTTAGCTTAAGAGCTCGCCATTTTAGCTTAAGAATTCGCCATGCTGATCTGTGCATAATATAATGAAAAATGGATACAAATTTGTGACTGCTACTTAGCATCGGCAATAGGTCTGAAAAAGCATCTAAAAATAGCAAATTTAGATATTTGTACCCTGTCGAAGTAAAGAACCATGGCATATATGTTTGGAATAGATTCCATTTTGAGAGAGTTGAAAAAGCACTATCTCGTTGAAAGGTTCTATCCATCTGCCCTTTCTCAACGCCTTTCTTTAGCCAATTTCGCCAAAGACGCAATTTTTTACTCGTTTCGGATGGTAAATATTTCTCAGAACGTGGAGTGTGAATCAAACCCATGTTTGAATTGAAATTGAGATACTGATGCAAGTTCTTCCTTTCTGAATCAGATAGATTCATATCTGAAAGAGGTTGACAATAAGTTCTTTCCAAATTGACTATTTCTTCCTCTGTTAGAGGTGTTCCAGAAATGTCTGCGATCGAGTAAATAGTTCTACGAACGAATAGATCGGATCGAATTGGAAAATGGAAAGATTTGTACAAGTTATACCCTTCGTTACCCCTTTGTGGAAAATCGTTAGGTATGAATATGTTAGATACCTGTGACTCGATTGGTGAAATAGTATCTCTCTCCAAAAAAGCATGTTTTTTTTTACCGACGCACAAAGAAAATTTTTTGTTGCGAATGAACAAGATATTGAGGAATTGTCTATACGTAAAATCATAATTCTTGATACGGGCCTTTTCCATATAAAAAGGGAATCTTTTCTTACAATAGAAGAAGAAGTGATGTGGATTATTCAAGAATCGAAGTCGATTTGCTTTATAAAAAGAAGATATCAATGAACTTCTATGAAATGCTTTTACGGGATTCAGCCAATTGTCTTGATCGCAGGATATCATTGAGAAATAGGAATCCGTGTTATCAAAGGATTTCCTGCGATTCTTTCTAGTATGGGAGTCAATCATCCACTTCCACTTTGGTATCTTATTGAACAAAAAGGGTGATATTGTTCCTCCATTGATCAATAATTTAGATTTTTGGGAAGTATCATGATCATCCGCTTTCCATTTTTTCAAATGAACGATTGGAAGACCTGGTGATTTTAACAACGGATTGCAGAGTTGATCATTCGGACCTTTCAATTCATAAATGTGGATCTCGGACCTATGAATGGGCATATTCCCTAAACTCACAAAGAAAAAAGGAAGTGAGTTAGACAAAAAGAGAATCAACTTTGACAATGACTTAGAAAATTTTTTTTTGTTGATAACCTTAGACCAATCAATCCAATATTGATTAATACGTAATCGATCGAAGACTACTTGAACTTGAAAACGGCTTTTCTGCTCAGAAACGAAATGTTCCTGGAAATTCTTGCTCCCGTTGAACCATTTGTATCTATATGCATCAGGATCCCGATTCATGGATCTCTCGCTTCGAGAAATCAAAATAAGAGAATCGAACCATTTCTTCTGATTCTTTTTCAAATTCGATAAATGTTGGTTGATCGTATATTTCATTATAGTTCTATGATTCAGAGTATCGTTTCCTATTTGATCCCTTTGAATTCCATATTCGAAGTTGCGATCGGATCTATTTATTAAAAAGAATCGATTCAATACATTTCTTATGTACCCATAAGTGCTATATTGGATTTGAATCAGATTTCGGATCAATCTATATTGATTGACTGCCTCCATTATGTTGTTGCTAGCAAATACCACTATTTTTTCTTTTGTATCTTTCAAATAATTCCCGCAGGAGATCCGAAGCCATTTTTTTCTGATCCTTCGATAAAAAGATTCATTCTCTTCATAAAAAATAGGAGGTAGAACCAATAAAGATTTCTTTTTCGATTCATCCCTGGAGTTGAATACCTCATTCAAGAATTGTTTTTGATCCAATCCGTAGGAATCAATAGAAAAGGCAAATCCCTTATGATACACCAGATCCGGCTCGGTTATTGATAGAGTGAATAGATCTGCCATTTCTTGAAATCTCTCTTCGGATTCAAAATCGTGGTGTAACGTGTATCCCCCCCTGTTCCGGTCATGGAATAGATGAAATAAATCAAAAAATGGATTTTTGTTCAAGAATGAAATCTTATTGGAACTGTCCATATTCGGTTCATCCTTCGGAACCATATCACATCCCCGATCTGATGAAATAGGATGAATTGAGACGGTCTTTTGTAAATACGTAATTATCTTGAATATATTAACCATTTCTTTATTTTCCGATCGCCTGCAGGGGACAAAAGAAACATCTTGTTCTTTCTTCAACAATTTCTGATCTCTAGTGGACCTCTCAGTAGGATTTGAACCCAGATGAAGTTCTGACCATCTGTCAGAGAAAAAAGAACGAATGGATCTTGTAGGATTCCCAAGAAGTTCTTCGATTTCTTCCGGAAGCAGATGATTATTCATCTGCTTCTCACCTTCCGTGAATAGCCGAGACATTGAGGAATATCCAGAAAGGCATTTCGGGAATCGGCCTGATCCTATCTCTGTTTCTTCCGTTTGACGAAAGGAAGGATCCCAAGGAATCGATCTTTCTTTTCGCTGTTGAATCTCCCTTTGATTGAGAGATGTGTGATATTCCGAATCCGCATTCCTGATGGAATCCAAGCGATCTCTAGATTGATCAAAAGATCCTTTCAATTGGCTAGAATCCCTCTTTTTTACGATCCAGTTCCTCCACCACCGCGAACCCCAGTTAGATTTACACCTTTTAGTTATTGGAGGAACCCAAGTACTCTCTTTCGGATCCAGGAAACAGCTCTCAGAGATCTTTTTTCCTTTTGGAAGATAGAGGAGCGAAACAATCAGCCTATTGATATTGGAAGACCCAAAGGATTCTTCCAATCTATCATTTCTGGACCCAAGAGAATTCATAGGTATAGGAAGAAGCCCTATCAAATAGATATTTTTTTTTTCGACCATATTTCGATTGTTAATACGATATATAAGGCCCGCTACTACAAAGAATACTACACCCTTGATTACAAAGAATACTACACCCGTGATCGTGAAAGTGAAATATCGCTTAATTATTGAACCCCGCGAATTGGGTGAAAGTAGGATACTCCAAATTCGGGGATCAAAGAGTTTTATAAAACGTTCTTGGTGGAAAAAAATCTTAACCAAAGATCCCACTGAATTCTTGATCTCTCTCAATATCTCTTTCAACTCGAAAACCCAGAAATCTAAATTTGTGGAATGTTTTTTTGTCATTTGTTAAAAAAAAAATTGAATTTTAAAATACTATAAATTTACTTTAATATGCAATTGTTAAAGTTAAAAAAAAAAATTCAATTTGATACTTATACTATAGTATAATACAATACGAAAACGTAATTGTATATCTATAATATAATATGATTATGATACTCCAATTATGATATTTCACTGAAAATTGAATCACTGAAAATGAAAATATGTCTAACATTTCTATATTCTATTTCAATGAAAATGTCAACAATGAAAATTTCAATGATATTTGTATAAAATGAAAATTGGATTTGTATTGATTTATCCTAAAGATTTCATTTCAATTGGAATTTGGTTATTCACCATGTACGAGGATCCCCGCTAAGCATCCATGGCTGAATGGTTAAAGCGCCCAACTCATAATTGGCGAATTCGTAGGTTCAATTCCTACTGGATGCACGCCAATGGGACCCTCCAATAAGTATAAGTCTATTGGAATTGGCTCTGTATCAATGGAATCTCATCCTCCATACATAACGAATTGGTATATTCATTTCATAACATAGGAACAGTAAGAACTAGCATTCTTATTGAGACTCGAACTCATAGGGAAGAAAATAGATAGAATCACATATGCAATATATGCCATTTTGAATATGAAGAAAATAGATAGAATCACATATGCAATATTTACCGACAAAAGTATTCGGTTATTGGGGAAAAATCAATATACTTCTAATGTCGAATCAGGATCAACTAGGACAGAAATAAAGCATTGGGTCGAACTCTTCTTTGGTGTCAAGGTAATAGCTATGAATAGTCATCGACTCCCGGGAAAGGGTAGAAGAATGGGACCTATTATGGGACATACAATGCATTACAGACGTATGATCATTACGCTTCAACCGGGTTATTCTATTCCACCTCTTAGAAAGAAAAGAACTTAAATAAAAATACTTAATAGCATGGCGATACATTTATACAAAACTTCTACCCCGAACACAGGCAATAGGGCCGTAGACAGTCAAGTGAAATCCAATCCACGAAATAATTTGATCTATGGACAGCGTCGTTGTGGTAAAGGTCGTAATGCCAGAGGAATCATTACCGCAAGGCATAGAGGGGGAGGTCATAAGCGTCTATACCGTAAAATCGATTTTCGACGGAATGAAAAAGACATATATGGTAGAATCGTAACCATAGAATACGACCCTAATCGAAATGCATACATTTGTCTCATACACTATGGGGATGGTGAGAAGAGATATATTTTACATCCCAGAGGGGCTATAATTGGAGATACCATTGTTTCTGGTACAGAAGTTCCTATAAAAATGGGAAATGCCCTACCTTTGAGTGCGGTTTGAACTATGGATTTACGTAATTGGAAGTAACCAATTAGGTTTACGACGAAACCTAGAAATCGATCACGGATCCAATTTGAGTACCTCTAGAGGATAGACCTCAACAGAAAACTGAAGAGTAACGGCAGCAAGTGATTGAGTTCAGTAGTTCCTCATATAAAATTATTGACTCTAGAGATATAGTAATATGGAGAAGACAAAATTGTTTCAAGCACCGACAGAACCGGAAGCGCCCCTTCTTTCAAAGAGAGGAGGACGGGTTATTCACATTTCATTTGATGGTCAGAGGCGAATTGAAAGCTAAGCAGTGTCTAAAGATTCCCCGGGGAAAAATAGAGATGTCTCCTACGTTACCCATAATATGTATATGTGGAAGTATCGACGTAATTTCATAGAGTCATTCGGTCTGAATGCTACATGAAGAACATAAGCCAGATGACGGAACGGGAAGACCTAGGATGTAGAAGATCATAACATGAGTGATTCGGCAGATTGGGATTCCTATATATCTGCTCATGTGGTACTTCATTGTATGATAAATAGAAGATCCATCTGTATAGATATCATCATCTACATCCAGAAAGCCGTATGCTTTGGAAGAAGCTTGTACAGTTTGGGAAGGGGTTTTGATTGATCAAAAAGAAGAATCTACTTCAACCGATATGCCTTTAGGCACGGCCATACATAACATAGAAATCACACTTGGAAAGGGTGGACAATTAGCTAGAGCAGCGGGTGCTGTAGCGAAACTGATTGCAAAAGAGGGGAAATCGGCCACATTAAAATTACCTTCTGGGGAGGTCCGTTTGATATCCAAAAACTGCTCAGCAACAGTCGGACAAGTGGGGAATGTTGGAGTGAACCAGAAAAGTTTGGGTAGAGCCGGATCTAAGCGTTGGCTAGGTAAACGTCCTGTAGTAAGAGGAGTCGTTATGAACCCTGTAGACCATCCCCATGGGGGTGGTGAAGGGAGGGCCCCAATTGGTAGAAAAAGCCCAACAAATAAATGGGGCTTTCCTGCACTTGGAAGAAGAAGTAGAAAAAGGAATAAATATAGTGATAATTTGATTATTCGTCGACGTAGTAAATAGGAGAGAAATTTGAATTAGTTTCTTCGTCTTTAAATAAAAAAAATAGGAGTAATTAACCGTGACACGTTCACTAAAAAAAAATCCTTTTGTAGCGAATCATTTATTAAGAAAAATTGATAAGCTTAACACAAAGGAGGAAAAAGAAATAATAGTAACTTGGTCCCGG
>NeomillspaughiaEmarginata
[truncated: 682,247 more chars]
