# Supplementary material for: The complete annotated plastome sequences of six genera in the tropical woody Polygonaceae
Source: BMC Plant Biol. 2024 May 17;24:417. doi: 10.1186/s12870-024-05144-y (PMC11100190; doi:10.1186/s12870-024-05144-y)
Supplement: Supplementary file 6 — Supplementary Material 6. [file 12870_2024_5144_MOESM6_ESM.docx]

Supplement6: This is the annotation for all of the chloroplast genomes assembled in this study. The information is in BED format.

CoccolobaRugosa 3 76 1_trnH-GUG_tRNA_CoccolobaRugosa
CoccolobaRugosa 603 1664 2_psbA_Exon_CoccolobaRugosa
CoccolobaRugosa 2194 3713 3_matK_Exon_CoccolobaRugosa
CoccolobaRugosa 5659 5889 4_rps16_Exon_CoccolobaRugosa
CoccolobaRugosa 5890 6772 5_rps16_Intron_CoccolobaRugosa
CoccolobaRugosa 6773 6808 6_rps16_Exon_CoccolobaRugosa
CoccolobaRugosa 8560 8631 7_trnQ-UUG_tRNA_CoccolobaRugosa
CoccolobaRugosa 8972 9157 8_psbK_Exon_CoccolobaRugosa
CoccolobaRugosa 9551 9661 9_psbI_Exon_CoccolobaRugosa
CoccolobaRugosa 9765 9852 10_trnS-GCU_tRNA_CoccolobaRugosa
CoccolobaRugosa 11986 12057 11_trnR-UCU_tRNA_CoccolobaRugosa
CoccolobaRugosa 12481 14004 12_atpA_Exon_CoccolobaRugosa
CoccolobaRugosa 14077 14487 13_atpF_Exon_CoccolobaRugosa
CoccolobaRugosa 14488 15235 14_atpF_Intron_CoccolobaRugosa
CoccolobaRugosa 15236 15379 15_atpF_Exon_CoccolobaRugosa
CoccolobaRugosa 15838 16083 16_atpH_Exon_CoccolobaRugosa
CoccolobaRugosa 16772 17515 17_atpI_Exon_CoccolobaRugosa
CoccolobaRugosa 17739 18449 18_rps2_Exon_CoccolobaRugosa
CoccolobaRugosa 18699 22773 19_rpoC2_Exon_CoccolobaRugosa
CoccolobaRugosa 22963 24565 20_rpoC1_Exon_CoccolobaRugosa
CoccolobaRugosa 24566 25336 21_rpoC1_Intron_CoccolobaRugosa
CoccolobaRugosa 25337 25766 22_rpoC1_Exon_CoccolobaRugosa
CoccolobaRugosa 25793 29005 23_rpoB_Exon_CoccolobaRugosa
CoccolobaRugosa 30328 30399 24_trnC-GCA_tRNA_CoccolobaRugosa
CoccolobaRugosa 31347 31436 25_petN_Exon_CoccolobaRugosa
CoccolobaRugosa 32550 32654 26_psbM_Exon_CoccolobaRugosa
CoccolobaRugosa 33849 33922 27_trnD-GUC_tRNA_CoccolobaRugosa
CoccolobaRugosa 34349 34432 28_trnY-GUA_tRNA_CoccolobaRugosa
CoccolobaRugosa 34504 34576 29_trnE-UUC_tRNA_CoccolobaRugosa
CoccolobaRugosa 35700 35771 30_trnT-GGU_tRNA_CoccolobaRugosa
CoccolobaRugosa 37146 38207 31_psbD_Exon_CoccolobaRugosa
CoccolobaRugosa 38191 39576 32_psbC_Exon_CoccolobaRugosa
CoccolobaRugosa 39859 39946 33_trnS-UGA_tRNA_CoccolobaRugosa
CoccolobaRugosa 40299 40487 34_psbZ_Exon_CoccolobaRugosa
CoccolobaRugosa 40796 40866 35_trnG-GCC_tRNA_CoccolobaRugosa
CoccolobaRugosa 41042 41115 36_trnM-CAU_tRNA_CoccolobaRugosa
CoccolobaRugosa 41271 41573 37_rps14_Exon_CoccolobaRugosa
CoccolobaRugosa 41698 43902 38_psaB_Exon_CoccolobaRugosa
CoccolobaRugosa 43928 46180 39_psaA_Exon_CoccolobaRugosa
CoccolobaRugosa 46926 47080 40_pafI_Exon_CoccolobaRugosa
CoccolobaRugosa 47081 47830 41_pafI_Intron_CoccolobaRugosa
CoccolobaRugosa 47831 48056 42_pafI_Exon_CoccolobaRugosa
CoccolobaRugosa 48057 48782 43_pafI_Intron_CoccolobaRugosa
CoccolobaRugosa 48783 48908 44_pafI_Exon_CoccolobaRugosa
CoccolobaRugosa 49853 49939 45_trnS-GGA_tRNA_CoccolobaRugosa
CoccolobaRugosa 50277 50873 46_rps4_Exon_CoccolobaRugosa
CoccolobaRugosa 51570 51642 47_trnT-UGU_tRNA_CoccolobaRugosa
CoccolobaRugosa 53656 53728 48_trnF-GAA_tRNA_CoccolobaRugosa
CoccolobaRugosa 54449 54925 49_ndhJ_Exon_CoccolobaRugosa
CoccolobaRugosa 55039 55716 50_ndhK_Exon_CoccolobaRugosa
CoccolobaRugosa 55776 56138 51_ndhC_Exon_CoccolobaRugosa
CoccolobaRugosa 58351 58423 52_trnM-CAU_tRNA_CoccolobaRugosa
CoccolobaRugosa 58883 59284 53_atpE_Exon_CoccolobaRugosa
CoccolobaRugosa 59281 60777 54_atpB_Exon_CoccolobaRugosa
CoccolobaRugosa 61652 63079 55_rbcL_Exon_CoccolobaRugosa
CoccolobaRugosa 63735 65183 56_accD_Exon_CoccolobaRugosa
CoccolobaRugosa 65940 66040 57_psaI_Exon_CoccolobaRugosa
CoccolobaRugosa 66495 67049 58_pafII_Exon_CoccolobaRugosa
CoccolobaRugosa 67955 68644 59_cemA_Exon_CoccolobaRugosa
CoccolobaRugosa 68865 69827 60_petA_Exon_CoccolobaRugosa
CoccolobaRugosa 70722 70844 61_psbJ_Exon_CoccolobaRugosa
CoccolobaRugosa 70977 71093 62_psbL_Exon_CoccolobaRugosa
CoccolobaRugosa 71121 71240 63_psbF_Exon_CoccolobaRugosa
CoccolobaRugosa 71249 71500 64_psbE_Exon_CoccolobaRugosa
CoccolobaRugosa 72779 72874 65_petL_Exon_CoccolobaRugosa
CoccolobaRugosa 73063 73176 66_petG_Exon_CoccolobaRugosa
CoccolobaRugosa 73296 73369 67_trnW-CCA_tRNA_CoccolobaRugosa
CoccolobaRugosa 73793 73866 68_trnP-UGG_tRNA_CoccolobaRugosa
CoccolobaRugosa 74280 74414 69_psaJ_Exon_CoccolobaRugosa
CoccolobaRugosa 74937 75137 70_rpl33_Exon_CoccolobaRugosa
CoccolobaRugosa 75691 75981 71_rps18_Exon_CoccolobaRugosa
CoccolobaRugosa 76264 76656 72_rpl20_Exon_CoccolobaRugosa
CoccolobaRugosa 77442 77555 73_rps12_Exon_CoccolobaRugosa
CoccolobaRugosa 77442 77555 74_rps12_Exon_CoccolobaRugosa
CoccolobaRugosa 77738 77962 75_clpP1_Exon_CoccolobaRugosa
CoccolobaRugosa 77963 78613 76_clpP1_Intron_CoccolobaRugosa
CoccolobaRugosa 78614 78905 77_clpP1_Exon_CoccolobaRugosa
CoccolobaRugosa 78906 80059 78_clpP1_Intron_CoccolobaRugosa
CoccolobaRugosa 80060 80130 79_clpP1_Exon_CoccolobaRugosa
CoccolobaRugosa 80571 82097 80_psbB_Exon_CoccolobaRugosa
CoccolobaRugosa 82268 82369 81_psbT_Exon_CoccolobaRugosa
CoccolobaRugosa 82441 82572 82_pbf1_Exon_CoccolobaRugosa
CoccolobaRugosa 82675 82896 83_psbH_Exon_CoccolobaRugosa
CoccolobaRugosa 83795 84440 84_petB_Exon_CoccolobaRugosa
CoccolobaRugosa 85427 85905 85_petD_Exon_CoccolobaRugosa
CoccolobaRugosa 86110 87103 86_rpoA_Exon_CoccolobaRugosa
CoccolobaRugosa 87175 87591 87_rps11_Exon_CoccolobaRugosa
CoccolobaRugosa 87735 87848 88_rpl36_Exon_CoccolobaRugosa
CoccolobaRugosa 87957 88190 89_infA_Exon_CoccolobaRugosa
CoccolobaRugosa 88309 88713 90_rps8_Exon_CoccolobaRugosa
CoccolobaRugosa 88921 89289 91_rpl14_Exon_CoccolobaRugosa
CoccolobaRugosa 89426 89828 92_rpl16_Exon_CoccolobaRugosa
CoccolobaRugosa 90888 91544 93_rps3_Exon_CoccolobaRugosa
CoccolobaRugosa 91607 91973 94_rpl22_Exon_CoccolobaRugosa
CoccolobaRugosa 92040 92318 95_rps19_Exon_CoccolobaRugosa
CoccolobaRugosa 92377 92810 96_rpl2_Exon_CoccolobaRugosa
CoccolobaRugosa 92811 93475 97_rpl2_Intron_CoccolobaRugosa
CoccolobaRugosa 93476 93866 98_rpl2_Exon_CoccolobaRugosa
CoccolobaRugosa 93885 94163 99_rpl23_Exon_CoccolobaRugosa
CoccolobaRugosa 94373 94446 100_trnM-CAU_tRNA_CoccolobaRugosa
CoccolobaRugosa 94884 101196 101_ycf2_Exon_CoccolobaRugosa
CoccolobaRugosa 102047 102127 102_trnL-CAA_tRNA_CoccolobaRugosa
CoccolobaRugosa 102695 103450 103_ndhB_Exon_CoccolobaRugosa
CoccolobaRugosa 103451 104129 104_ndhB_Intron_CoccolobaRugosa
CoccolobaRugosa 104130 104906 105_ndhB_Exon_CoccolobaRugosa
CoccolobaRugosa 105239 105706 106_rps7_Exon_CoccolobaRugosa
CoccolobaRugosa 105760 105786 107_rps12_Exon_CoccolobaRugosa
CoccolobaRugosa 105787 106313 108_rps12_Intron_CoccolobaRugosa
CoccolobaRugosa 106314 106544 109_rps12_Exon_CoccolobaRugosa
CoccolobaRugosa 108374 108445 110_trnV-GAC_tRNA_CoccolobaRugosa
CoccolobaRugosa 108673 110163 111_rrn16_rRNA_CoccolobaRugosa
CoccolobaRugosa 110457 110544 112_trnI-GAU_tRNA_CoccolobaRugosa
CoccolobaRugosa 110493 110508 113_trnI-GAU_Intron_CoccolobaRugosa
CoccolobaRugosa 111541 111645 114_trnA-UGC_tRNA_CoccolobaRugosa
CoccolobaRugosa 111578 111617 115_trnA-UGC_Intron_CoccolobaRugosa
CoccolobaRugosa 112574 112885 116_rrn23-fragment_rRNA_CoccolobaRugosa
CoccolobaRugosa 112893 115385 117_rrn23_rRNA_CoccolobaRugosa
CoccolobaRugosa 115484 115586 118_rrn4.5_rRNA_CoccolobaRugosa
CoccolobaRugosa 115837 115957 119_rrn5_rRNA_CoccolobaRugosa
CoccolobaRugosa 116217 116290 120_trnR-ACG_tRNA_CoccolobaRugosa
CoccolobaRugosa 116912 116983 121_trnN-GUU_tRNA_CoccolobaRugosa
CoccolobaRugosa 117534 123261 122_ycf1_Exon_CoccolobaRugosa
CoccolobaRugosa 123497 125695 123_ndhF_Exon_CoccolobaRugosa
CoccolobaRugosa 126772 126945 124_rpl32_Exon_CoccolobaRugosa
CoccolobaRugosa 128233 128312 125_trnL-UAG_tRNA_CoccolobaRugosa
CoccolobaRugosa 128456 129408 126_ccsA_Exon_CoccolobaRugosa
CoccolobaRugosa 129626 131128 127_ndhD_Exon_CoccolobaRugosa
CoccolobaRugosa 131268 131513 128_psaC_Exon_CoccolobaRugosa
CoccolobaRugosa 131768 132073 129_ndhE_Exon_CoccolobaRugosa
CoccolobaRugosa 132300 132830 130_ndhG_Exon_CoccolobaRugosa
CoccolobaRugosa 133207 133695 131_ndhI_Exon_CoccolobaRugosa
CoccolobaRugosa 133795 134335 132_ndhA_Exon_CoccolobaRugosa
CoccolobaRugosa 134336 135453 133_ndhA_Intron_CoccolobaRugosa
CoccolobaRugosa 135454 136004 134_ndhA_Exon_CoccolobaRugosa
CoccolobaRugosa 136006 137186 135_ndhH_Exon_CoccolobaRugosa
CoccolobaRugosa 137286 137558 136_rps15_Exon_CoccolobaRugosa
CoccolobaRugosa 137851 143578 137_ycf1_Exon_CoccolobaRugosa
CoccolobaRugosa 144129 144200 138_trnN-GUU_tRNA_CoccolobaRugosa
CoccolobaRugosa 144822 144895 139_trnR-ACG_tRNA_CoccolobaRugosa
CoccolobaRugosa 145155 145275 140_rrn5_rRNA_CoccolobaRugosa
CoccolobaRugosa 145526 145628 141_rrn4.5_rRNA_CoccolobaRugosa
CoccolobaRugosa 145727 148219 142_rrn23_rRNA_CoccolobaRugosa
CoccolobaRugosa 148227 148538 143_rrn23-fragment_rRNA_CoccolobaRugosa
CoccolobaRugosa 149467 149571 144_trnA-UGC_tRNA_CoccolobaRugosa
CoccolobaRugosa 149495 149534 145_trnA-UGC_Intron_CoccolobaRugosa
CoccolobaRugosa 150568 150655 146_trnI-GAU_tRNA_CoccolobaRugosa
CoccolobaRugosa 150604 150619 147_trnI-GAU_Intron_CoccolobaRugosa
CoccolobaRugosa 150949 152439 148_rrn16_rRNA_CoccolobaRugosa
CoccolobaRugosa 152667 152738 149_trnV-GAC_tRNA_CoccolobaRugosa
CoccolobaRugosa 154568 154798 150_rps12_Exon_CoccolobaRugosa
CoccolobaRugosa 154799 155325 151_rps12_Intron_CoccolobaRugosa
CoccolobaRugosa 155326 155352 152_rps12_Exon_CoccolobaRugosa
CoccolobaRugosa 155406 155873 153_rps7_Exon_CoccolobaRugosa
CoccolobaRugosa 156206 156982 154_ndhB_Exon_CoccolobaRugosa
CoccolobaRugosa 156983 157661 155_ndhB_Intron_CoccolobaRugosa
CoccolobaRugosa 157662 158417 156_ndhB_Exon_CoccolobaRugosa
CoccolobaRugosa 158985 159065 157_trnL-CAA_tRNA_CoccolobaRugosa
CoccolobaRugosa 159916 166228 158_ycf2_Exon_CoccolobaRugosa
CoccolobaRugosa 166666 166739 159_trnM-CAU_tRNA_CoccolobaRugosa
CoccolobaRugosa 166949 167227 160_rpl23_Exon_CoccolobaRugosa
CoccolobaRugosa 167246 167636 161_rpl2_Exon_CoccolobaRugosa
CoccolobaRugosa 167637 168301 162_rpl2_Intron_CoccolobaRugosa
CoccolobaRugosa 168302 168735 163_rpl2_Exon_CoccolobaRugosa
CoccolobaRugosa 168794 168901 164_rps19-fragment_Exon_CoccolobaRugosa
GymnopodiumFloribundum 3 76 1_trnH-GUG_tRNA_GymnopodiumFloribundum
GymnopodiumFloribundum 352 1413 2_psbA_Exon_GymnopodiumFloribundum
GymnopodiumFloribundum 1942 3470 3_matK_Exon_GymnopodiumFloribundum
GymnopodiumFloribundum 5155 5385 4_rps16_Exon_GymnopodiumFloribundum
GymnopodiumFloribundum 5386 6251 5_rps16_Intron_GymnopodiumFloribundum
GymnopodiumFloribundum 6252 6287 6_rps16_Exon_GymnopodiumFloribundum
GymnopodiumFloribundum 8139 8210 7_trnQ-UUG_tRNA_GymnopodiumFloribundum
GymnopodiumFloribundum 8547 8732 8_psbK_Exon_GymnopodiumFloribundum
GymnopodiumFloribundum 9125 9235 9_psbI_Exon_GymnopodiumFloribundum
GymnopodiumFloribundum 9363 9450 10_trnS-GCU_tRNA_GymnopodiumFloribundum
GymnopodiumFloribundum 11453 11524 11_trnR-UCU_tRNA_GymnopodiumFloribundum
GymnopodiumFloribundum 12033 13556 12_atpA_Exon_GymnopodiumFloribundum
GymnopodiumFloribundum 13628 14038 13_atpF_Exon_GymnopodiumFloribundum
GymnopodiumFloribundum 14039 14785 14_atpF_Intron_GymnopodiumFloribundum
GymnopodiumFloribundum 14786 14929 15_atpF_Exon_GymnopodiumFloribundum
GymnopodiumFloribundum 15393 15638 16_atpH_Exon_GymnopodiumFloribundum
GymnopodiumFloribundum 16319 17062 17_atpI_Exon_GymnopodiumFloribundum
GymnopodiumFloribundum 17282 17992 18_rps2_Exon_GymnopodiumFloribundum
GymnopodiumFloribundum 18246 22326 19_rpoC2_Exon_GymnopodiumFloribundum
GymnopodiumFloribundum 22516 24118 20_rpoC1_Exon_GymnopodiumFloribundum
GymnopodiumFloribundum 24119 24884 21_rpoC1_Intron_GymnopodiumFloribundum
GymnopodiumFloribundum 24885 25314 22_rpoC1_Exon_GymnopodiumFloribundum
GymnopodiumFloribundum 25341 28553 23_rpoB_Exon_GymnopodiumFloribundum
GymnopodiumFloribundum 29948 30019 24_trnC-GCA_tRNA_GymnopodiumFloribundum
GymnopodiumFloribundum 30947 31036 25_petN_Exon_GymnopodiumFloribundum
GymnopodiumFloribundum 32149 32253 26_psbM_Exon_GymnopodiumFloribundum
GymnopodiumFloribundum 33488 33561 27_trnD-GUC_tRNA_GymnopodiumFloribundum
GymnopodiumFloribundum 33996 34079 28_trnY-GUA_tRNA_GymnopodiumFloribundum
GymnopodiumFloribundum 34151 34223 29_trnE-UUC_tRNA_GymnopodiumFloribundum
GymnopodiumFloribundum 35554 35625 30_trnT-GGU_tRNA_GymnopodiumFloribundum
GymnopodiumFloribundum 36978 38039 31_psbD_Exon_GymnopodiumFloribundum
GymnopodiumFloribundum 38023 39408 32_psbC_Exon_GymnopodiumFloribundum
GymnopodiumFloribundum 39678 39765 33_trnS-UGA_tRNA_GymnopodiumFloribundum
GymnopodiumFloribundum 40110 40298 34_psbZ_Exon_GymnopodiumFloribundum
GymnopodiumFloribundum 40611 40681 35_trnG-GCC_tRNA_GymnopodiumFloribundum
GymnopodiumFloribundum 40847 40920 36_trnM-CAU_tRNA_GymnopodiumFloribundum
GymnopodiumFloribundum 41069 41371 37_rps14_Exon_GymnopodiumFloribundum
GymnopodiumFloribundum 41497 43701 38_psaB_Exon_GymnopodiumFloribundum
GymnopodiumFloribundum 43727 45979 39_psaA_Exon_GymnopodiumFloribundum
GymnopodiumFloribundum 46699 46853 40_pafI_Exon_GymnopodiumFloribundum
GymnopodiumFloribundum 46854 47600 41_pafI_Intron_GymnopodiumFloribundum
GymnopodiumFloribundum 47601 47826 42_pafI_Exon_GymnopodiumFloribundum
GymnopodiumFloribundum 47827 48558 43_pafI_Intron_GymnopodiumFloribundum
GymnopodiumFloribundum 48559 48684 44_pafI_Exon_GymnopodiumFloribundum
GymnopodiumFloribundum 49622 49708 45_trnS-GGA_tRNA_GymnopodiumFloribundum
GymnopodiumFloribundum 50034 50630 46_rps4_Exon_GymnopodiumFloribundum
GymnopodiumFloribundum 51402 51474 47_trnT-UGU_tRNA_GymnopodiumFloribundum
GymnopodiumFloribundum 53511 53583 48_trnF-GAA_tRNA_GymnopodiumFloribundum
GymnopodiumFloribundum 54319 54795 49_ndhJ_Exon_GymnopodiumFloribundum
GymnopodiumFloribundum 54904 55581 50_ndhK_Exon_GymnopodiumFloribundum
GymnopodiumFloribundum 55641 56003 51_ndhC_Exon_GymnopodiumFloribundum
GymnopodiumFloribundum 58262 58334 52_trnM-CAU_tRNA_GymnopodiumFloribundum
GymnopodiumFloribundum 58680 59081 53_atpE_Exon_GymnopodiumFloribundum
GymnopodiumFloribundum 59078 60574 54_atpB_Exon_GymnopodiumFloribundum
GymnopodiumFloribundum 61411 62838 55_rbcL_Exon_GymnopodiumFloribundum
GymnopodiumFloribundum 63483 64907 56_accD_Exon_GymnopodiumFloribundum
GymnopodiumFloribundum 65667 65767 57_psaI_Exon_GymnopodiumFloribundum
GymnopodiumFloribundum 66222 66776 58_pafII_Exon_GymnopodiumFloribundum
GymnopodiumFloribundum 67688 68377 59_cemA_Exon_GymnopodiumFloribundum
GymnopodiumFloribundum 68598 69560 60_petA_Exon_GymnopodiumFloribundum
GymnopodiumFloribundum 70465 70587 61_psbJ_Exon_GymnopodiumFloribundum
GymnopodiumFloribundum 70720 70836 62_psbL_Exon_GymnopodiumFloribundum
GymnopodiumFloribundum 70864 70983 63_psbF_Exon_GymnopodiumFloribundum
GymnopodiumFloribundum 70992 71243 64_psbE_Exon_GymnopodiumFloribundum
GymnopodiumFloribundum 72539 72634 65_petL_Exon_GymnopodiumFloribundum
GymnopodiumFloribundum 72817 72930 66_petG_Exon_GymnopodiumFloribundum
GymnopodiumFloribundum 73050 73123 67_trnW-CCA_tRNA_GymnopodiumFloribundum
GymnopodiumFloribundum 73549 73622 68_trnP-UGG_tRNA_GymnopodiumFloribundum
GymnopodiumFloribundum 74025 74159 69_psaJ_Exon_GymnopodiumFloribundum
GymnopodiumFloribundum 74662 74862 70_rpl33_Exon_GymnopodiumFloribundum
GymnopodiumFloribundum 75551 75841 71_rps18_Exon_GymnopodiumFloribundum
GymnopodiumFloribundum 76098 76490 72_rpl20_Exon_GymnopodiumFloribundum
GymnopodiumFloribundum 77270 77383 73_rps12_Exon_GymnopodiumFloribundum
GymnopodiumFloribundum 77270 77383 74_rps12_Exon_GymnopodiumFloribundum
GymnopodiumFloribundum 77551 77775 75_clpP1_Exon_GymnopodiumFloribundum
GymnopodiumFloribundum 77776 78417 76_clpP1_Intron_GymnopodiumFloribundum
GymnopodiumFloribundum 78418 78709 77_clpP1_Exon_GymnopodiumFloribundum
GymnopodiumFloribundum 78710 79892 78_clpP1_Intron_GymnopodiumFloribundum
GymnopodiumFloribundum 79893 79963 79_clpP1_Exon_GymnopodiumFloribundum
GymnopodiumFloribundum 80409 81935 80_psbB_Exon_GymnopodiumFloribundum
GymnopodiumFloribundum 82106 82207 81_psbT_Exon_GymnopodiumFloribundum
GymnopodiumFloribundum 82279 82410 82_pbf1_Exon_GymnopodiumFloribundum
GymnopodiumFloribundum 82513 82734 83_psbH_Exon_GymnopodiumFloribundum
GymnopodiumFloribundum 83630 84275 84_petB_Exon_GymnopodiumFloribundum
GymnopodiumFloribundum 85265 85743 85_petD_Exon_GymnopodiumFloribundum
GymnopodiumFloribundum 85948 86941 86_rpoA_Exon_GymnopodiumFloribundum
GymnopodiumFloribundum 87013 87429 87_rps11_Exon_GymnopodiumFloribundum
GymnopodiumFloribundum 87569 87682 88_rpl36_Exon_GymnopodiumFloribundum
GymnopodiumFloribundum 87791 88024 89_infA_Exon_GymnopodiumFloribundum
GymnopodiumFloribundum 88143 88547 90_rps8_Exon_GymnopodiumFloribundum
GymnopodiumFloribundum 88766 89134 91_rpl14_Exon_GymnopodiumFloribundum
GymnopodiumFloribundum 89265 89667 92_rpl16_Exon_GymnopodiumFloribundum
GymnopodiumFloribundum 91177 91833 93_rps3_Exon_GymnopodiumFloribundum
GymnopodiumFloribundum 91943 92300 94_rpl22_Exon_GymnopodiumFloribundum
GymnopodiumFloribundum 92359 92637 95_rps19_Exon_GymnopodiumFloribundum
GymnopodiumFloribundum 92696 93129 96_rpl2_Exon_GymnopodiumFloribundum
GymnopodiumFloribundum 93130 93794 97_rpl2_Intron_GymnopodiumFloribundum
GymnopodiumFloribundum 93795 94185 98_rpl2_Exon_GymnopodiumFloribundum
GymnopodiumFloribundum 94204 94482 99_rpl23_Exon_GymnopodiumFloribundum
GymnopodiumFloribundum 94692 94765 100_trnM-CAU_tRNA_GymnopodiumFloribundum
GymnopodiumFloribundum 95159 101462 101_ycf2_Exon_GymnopodiumFloribundum
GymnopodiumFloribundum 102311 102391 102_trnL-CAA_tRNA_GymnopodiumFloribundum
GymnopodiumFloribundum 102959 103714 103_ndhB_Exon_GymnopodiumFloribundum
GymnopodiumFloribundum 103715 104393 104_ndhB_Intron_GymnopodiumFloribundum
GymnopodiumFloribundum 104394 105170 105_ndhB_Exon_GymnopodiumFloribundum
GymnopodiumFloribundum 105498 105965 106_rps7_Exon_GymnopodiumFloribundum
GymnopodiumFloribundum 106019 106045 107_rps12_Exon_GymnopodiumFloribundum
GymnopodiumFloribundum 106046 106572 108_rps12_Intron_GymnopodiumFloribundum
GymnopodiumFloribundum 106573 106803 109_rps12_Exon_GymnopodiumFloribundum
GymnopodiumFloribundum 108640 108711 110_trnV-GAC_tRNA_GymnopodiumFloribundum
GymnopodiumFloribundum 108939 110429 111_rrn16_rRNA_GymnopodiumFloribundum
GymnopodiumFloribundum 110723 110810 112_trnI-GAU_tRNA_GymnopodiumFloribundum
GymnopodiumFloribundum 110759 110774 113_trnI-GAU_Intron_GymnopodiumFloribundum
GymnopodiumFloribundum 111817 111921 114_trnA-UGC_tRNA_GymnopodiumFloribundum
GymnopodiumFloribundum 111854 111893 115_trnA-UGC_Intron_GymnopodiumFloribundum
GymnopodiumFloribundum 112850 113403 116_rrn23-fragment_rRNA_GymnopodiumFloribundum
GymnopodiumFloribundum 113409 115661 117_rrn23_rRNA_GymnopodiumFloribundum
GymnopodiumFloribundum 115760 115862 118_rrn4.5_rRNA_GymnopodiumFloribundum
GymnopodiumFloribundum 116114 116234 119_rrn5_rRNA_GymnopodiumFloribundum
GymnopodiumFloribundum 116497 116570 120_trnR-ACG_tRNA_GymnopodiumFloribundum
GymnopodiumFloribundum 117197 117268 121_trnN-GUU_tRNA_GymnopodiumFloribundum
GymnopodiumFloribundum 117828 123567 122_ycf1-fragment_Exon_GymnopodiumFloribundum
GymnopodiumFloribundum 123798 125995 123_ndhF_Exon_GymnopodiumFloribundum
GymnopodiumFloribundum 127016 127166 124_rpl32_Exon_GymnopodiumFloribundum
GymnopodiumFloribundum 127976 128055 125_trnL-UAG_tRNA_GymnopodiumFloribundum
GymnopodiumFloribundum 128201 129153 126_ccsA_Exon_GymnopodiumFloribundum
GymnopodiumFloribundum 129370 130872 127_ndhD_Exon_GymnopodiumFloribundum
GymnopodiumFloribundum 131014 131259 128_psaC_Exon_GymnopodiumFloribundum
GymnopodiumFloribundum 131542 131847 129_ndhE_Exon_GymnopodiumFloribundum
GymnopodiumFloribundum 132074 132604 130_ndhG_Exon_GymnopodiumFloribundum
GymnopodiumFloribundum 132978 133466 131_ndhI_Exon_GymnopodiumFloribundum
GymnopodiumFloribundum 133568 134108 132_ndhA_Exon_GymnopodiumFloribundum
GymnopodiumFloribundum 134109 135217 133_ndhA_Intron_GymnopodiumFloribundum
GymnopodiumFloribundum 135218 135768 134_ndhA_Exon_GymnopodiumFloribundum
GymnopodiumFloribundum 135770 136950 135_ndhH_Exon_GymnopodiumFloribundum
GymnopodiumFloribundum 137050 137322 136_rps15_Exon_GymnopodiumFloribundum
GymnopodiumFloribundum 137614 143353 137_ycf1_Exon_GymnopodiumFloribundum
GymnopodiumFloribundum 143913 143984 138_trnN-GUU_tRNA_GymnopodiumFloribundum
GymnopodiumFloribundum 144611 144684 139_trnR-ACG_tRNA_GymnopodiumFloribundum
GymnopodiumFloribundum 144947 145067 140_rrn5_rRNA_GymnopodiumFloribundum
GymnopodiumFloribundum 145319 145421 141_rrn4.5_rRNA_GymnopodiumFloribundum
GymnopodiumFloribundum 145520 147772 142_rrn23_rRNA_GymnopodiumFloribundum
GymnopodiumFloribundum 147778 148331 143_rrn23-fragment_rRNA_GymnopodiumFloribundum
GymnopodiumFloribundum 149260 149364 144_trnA-UGC_tRNA_GymnopodiumFloribundum
GymnopodiumFloribundum 149288 149327 145_trnA-UGC_Intron_GymnopodiumFloribundum
GymnopodiumFloribundum 150371 150458 146_trnI-GAU_tRNA_GymnopodiumFloribundum
GymnopodiumFloribundum 150407 150422 147_trnI-GAU_Intron_GymnopodiumFloribundum
GymnopodiumFloribundum 150752 152242 148_rrn16_rRNA_GymnopodiumFloribundum
GymnopodiumFloribundum 152470 152541 149_trnV-GAC_tRNA_GymnopodiumFloribundum
GymnopodiumFloribundum 154378 154608 150_rps12_Exon_GymnopodiumFloribundum
GymnopodiumFloribundum 154609 155135 151_rps12_Intron_GymnopodiumFloribundum
GymnopodiumFloribundum 155136 155162 152_rps12_Exon_GymnopodiumFloribundum
GymnopodiumFloribundum 155216 155683 153_rps7_Exon_GymnopodiumFloribundum
GymnopodiumFloribundum 156011 156787 154_ndhB_Exon_GymnopodiumFloribundum
GymnopodiumFloribundum 156788 157466 155_ndhB_Intron_GymnopodiumFloribundum
GymnopodiumFloribundum 157467 158222 156_ndhB_Exon_GymnopodiumFloribundum
GymnopodiumFloribundum 158790 158870 157_trnL-CAA_tRNA_GymnopodiumFloribundum
GymnopodiumFloribundum 159719 166022 158_ycf2_Exon_GymnopodiumFloribundum
GymnopodiumFloribundum 166416 166489 159_trnM-CAU_tRNA_GymnopodiumFloribundum
GymnopodiumFloribundum 166699 166977 160_rpl23_Exon_GymnopodiumFloribundum
GymnopodiumFloribundum 166996 167386 161_rpl2_Exon_GymnopodiumFloribundum
GymnopodiumFloribundum 167387 168051 162_rpl2_Intron_GymnopodiumFloribundum
GymnopodiumFloribundum 168052 168485 163_rpl2_Exon_GymnopodiumFloribundum
GymnopodiumFloribundum 168544 168651 164_rps19-fragment_Exon_GymnopodiumFloribundum
NeomillspaughiaEmarginata 3 76 1_trnH-GUG_tRNA_NeomillspaughiaEmarginata
NeomillspaughiaEmarginata 571 1632 2_psbA_Exon_NeomillspaughiaEmarginata
NeomillspaughiaEmarginata 2162 3681 3_matK_Exon_NeomillspaughiaEmarginata
NeomillspaughiaEmarginata 5633 5863 4_rps16_Exon_NeomillspaughiaEmarginata
NeomillspaughiaEmarginata 5864 6727 5_rps16_Intron_NeomillspaughiaEmarginata
NeomillspaughiaEmarginata 6728 6763 6_rps16_Exon_NeomillspaughiaEmarginata
NeomillspaughiaEmarginata 8589 8660 7_trnQ-UUG_tRNA_NeomillspaughiaEmarginata
NeomillspaughiaEmarginata 9002 9187 8_psbK_Exon_NeomillspaughiaEmarginata
NeomillspaughiaEmarginata 9580 9690 9_psbI_Exon_NeomillspaughiaEmarginata
NeomillspaughiaEmarginata 9821 9908 10_trnS-GCU_tRNA_NeomillspaughiaEmarginata
NeomillspaughiaEmarginata 12099 12170 11_trnR-UCU_tRNA_NeomillspaughiaEmarginata
NeomillspaughiaEmarginata 12683 14206 12_atpA_Exon_NeomillspaughiaEmarginata
NeomillspaughiaEmarginata 14279 14689 13_atpF_Exon_NeomillspaughiaEmarginata
NeomillspaughiaEmarginata 14690 15440 14_atpF_Intron_NeomillspaughiaEmarginata
NeomillspaughiaEmarginata 15441 15584 15_atpF_Exon_NeomillspaughiaEmarginata
NeomillspaughiaEmarginata 16037 16282 16_atpH_Exon_NeomillspaughiaEmarginata
NeomillspaughiaEmarginata 16956 17699 17_atpI_Exon_NeomillspaughiaEmarginata
NeomillspaughiaEmarginata 17921 18631 18_rps2_Exon_NeomillspaughiaEmarginata
NeomillspaughiaEmarginata 18883 22957 19_rpoC2_Exon_NeomillspaughiaEmarginata
NeomillspaughiaEmarginata 23147 24749 20_rpoC1_Exon_NeomillspaughiaEmarginata
NeomillspaughiaEmarginata 24750 25527 21_rpoC1_Intron_NeomillspaughiaEmarginata
NeomillspaughiaEmarginata 25528 25957 22_rpoC1_Exon_NeomillspaughiaEmarginata
NeomillspaughiaEmarginata 25984 29196 23_rpoB_Exon_NeomillspaughiaEmarginata
NeomillspaughiaEmarginata 30520 30591 24_trnC-GCA_tRNA_NeomillspaughiaEmarginata
NeomillspaughiaEmarginata 31545 31634 25_petN_Exon_NeomillspaughiaEmarginata
NeomillspaughiaEmarginata 32766 32870 26_psbM_Exon_NeomillspaughiaEmarginata
NeomillspaughiaEmarginata 34067 34140 27_trnD-GUC_tRNA_NeomillspaughiaEmarginata
NeomillspaughiaEmarginata 34566 34649 28_trnY-GUA_tRNA_NeomillspaughiaEmarginata
NeomillspaughiaEmarginata 34721 34793 29_trnE-UUC_tRNA_NeomillspaughiaEmarginata
NeomillspaughiaEmarginata 35909 35980 30_trnT-GGU_tRNA_NeomillspaughiaEmarginata
NeomillspaughiaEmarginata 37362 38423 31_psbD_Exon_NeomillspaughiaEmarginata
NeomillspaughiaEmarginata 38407 39792 32_psbC_Exon_NeomillspaughiaEmarginata
NeomillspaughiaEmarginata 40083 40170 33_trnS-UGA_tRNA_NeomillspaughiaEmarginata
NeomillspaughiaEmarginata 40523 40711 34_psbZ_Exon_NeomillspaughiaEmarginata
NeomillspaughiaEmarginata 41020 41090 35_trnG-GCC_tRNA_NeomillspaughiaEmarginata
NeomillspaughiaEmarginata 41266 41339 36_trnM-CAU_tRNA_NeomillspaughiaEmarginata
NeomillspaughiaEmarginata 41495 41797 37_rps14_Exon_NeomillspaughiaEmarginata
NeomillspaughiaEmarginata 41922 44126 38_psaB_Exon_NeomillspaughiaEmarginata
NeomillspaughiaEmarginata 44152 46404 39_psaA_Exon_NeomillspaughiaEmarginata
NeomillspaughiaEmarginata 47155 47309 40_pafI_Exon_NeomillspaughiaEmarginata
NeomillspaughiaEmarginata 47310 48059 41_pafI_Intron_NeomillspaughiaEmarginata
NeomillspaughiaEmarginata 48060 48285 42_pafI_Exon_NeomillspaughiaEmarginata
NeomillspaughiaEmarginata 48286 49011 43_pafI_Intron_NeomillspaughiaEmarginata
NeomillspaughiaEmarginata 49012 49137 44_pafI_Exon_NeomillspaughiaEmarginata
NeomillspaughiaEmarginata 50079 50165 45_trnS-GGA_tRNA_NeomillspaughiaEmarginata
NeomillspaughiaEmarginata 50521 51117 46_rps4_Exon_NeomillspaughiaEmarginata
NeomillspaughiaEmarginata 52161 52233 47_trnT-UGU_tRNA_NeomillspaughiaEmarginata
NeomillspaughiaEmarginata 54230 54302 48_trnF-GAA_tRNA_NeomillspaughiaEmarginata
NeomillspaughiaEmarginata 55021 55497 49_ndhJ_Exon_NeomillspaughiaEmarginata
NeomillspaughiaEmarginata 55611 56288 50_ndhK_Exon_NeomillspaughiaEmarginata
NeomillspaughiaEmarginata 56348 56710 51_ndhC_Exon_NeomillspaughiaEmarginata
NeomillspaughiaEmarginata 59010 59082 52_trnM-CAU_tRNA_NeomillspaughiaEmarginata
NeomillspaughiaEmarginata 59532 59933 53_atpE_Exon_NeomillspaughiaEmarginata
NeomillspaughiaEmarginata 59930 61426 54_atpB_Exon_NeomillspaughiaEmarginata
NeomillspaughiaEmarginata 62279 63706 55_rbcL_Exon_NeomillspaughiaEmarginata
NeomillspaughiaEmarginata 64398 65846 56_accD_Exon_NeomillspaughiaEmarginata
NeomillspaughiaEmarginata 66597 66697 57_psaI_Exon_NeomillspaughiaEmarginata
NeomillspaughiaEmarginata 67152 67706 58_pafII_Exon_NeomillspaughiaEmarginata
NeomillspaughiaEmarginata 68612 69301 59_cemA_Exon_NeomillspaughiaEmarginata
NeomillspaughiaEmarginata 69522 70484 60_petA_Exon_NeomillspaughiaEmarginata
NeomillspaughiaEmarginata 71377 71499 61_psbJ_Exon_NeomillspaughiaEmarginata
NeomillspaughiaEmarginata 71632 71748 62_psbL_Exon_NeomillspaughiaEmarginata
NeomillspaughiaEmarginata 71776 71895 63_psbF_Exon_NeomillspaughiaEmarginata
NeomillspaughiaEmarginata 71904 72155 64_psbE_Exon_NeomillspaughiaEmarginata
NeomillspaughiaEmarginata 73430 73525 65_petL_Exon_NeomillspaughiaEmarginata
NeomillspaughiaEmarginata 73714 73827 66_petG_Exon_NeomillspaughiaEmarginata
NeomillspaughiaEmarginata 73947 74020 67_trnW-CCA_tRNA_NeomillspaughiaEmarginata
NeomillspaughiaEmarginata 74493 74566 68_trnP-UGG_tRNA_NeomillspaughiaEmarginata
NeomillspaughiaEmarginata 74980 75114 69_psaJ_Exon_NeomillspaughiaEmarginata
NeomillspaughiaEmarginata 75637 75837 70_rpl33_Exon_NeomillspaughiaEmarginata
NeomillspaughiaEmarginata 76410 76700 71_rps18_Exon_NeomillspaughiaEmarginata
NeomillspaughiaEmarginata 76956 77348 72_rpl20_Exon_NeomillspaughiaEmarginata
NeomillspaughiaEmarginata 78133 78246 73_rps12_Exon_NeomillspaughiaEmarginata
NeomillspaughiaEmarginata 78133 78246 74_rps12_Exon_NeomillspaughiaEmarginata
NeomillspaughiaEmarginata 78424 78648 75_clpP1_Exon_NeomillspaughiaEmarginata
NeomillspaughiaEmarginata 78649 79293 76_clpP1_Intron_NeomillspaughiaEmarginata
NeomillspaughiaEmarginata 79294 79585 77_clpP1_Exon_NeomillspaughiaEmarginata
NeomillspaughiaEmarginata 79586 80739 78_clpP1_Intron_NeomillspaughiaEmarginata
NeomillspaughiaEmarginata 80740 80810 79_clpP1_Exon_NeomillspaughiaEmarginata
NeomillspaughiaEmarginata 81256 82782 80_psbB_Exon_NeomillspaughiaEmarginata
NeomillspaughiaEmarginata 82953 83054 81_psbT_Exon_NeomillspaughiaEmarginata
NeomillspaughiaEmarginata 83126 83257 82_pbf1_Exon_NeomillspaughiaEmarginata
NeomillspaughiaEmarginata 83360 83581 83_psbH_Exon_NeomillspaughiaEmarginata
NeomillspaughiaEmarginata 84485 85130 84_petB_Exon_NeomillspaughiaEmarginata
NeomillspaughiaEmarginata 86132 86610 85_petD_Exon_NeomillspaughiaEmarginata
NeomillspaughiaEmarginata 86815 87808 86_rpoA_Exon_NeomillspaughiaEmarginata
NeomillspaughiaEmarginata 87880 88296 87_rps11_Exon_NeomillspaughiaEmarginata
NeomillspaughiaEmarginata 88441 88554 88_rpl36_Exon_NeomillspaughiaEmarginata
NeomillspaughiaEmarginata 88663 88896 89_infA_Exon_NeomillspaughiaEmarginata
NeomillspaughiaEmarginata 89015 89419 90_rps8_Exon_NeomillspaughiaEmarginata
NeomillspaughiaEmarginata 89620 89988 91_rpl14_Exon_NeomillspaughiaEmarginata
NeomillspaughiaEmarginata 90125 90527 92_rpl16_Exon_NeomillspaughiaEmarginata
NeomillspaughiaEmarginata 91818 92474 93_rps3_Exon_NeomillspaughiaEmarginata
NeomillspaughiaEmarginata 92537 92903 94_rpl22_Exon_NeomillspaughiaEmarginata
NeomillspaughiaEmarginata 92970 93248 95_rps19_Exon_NeomillspaughiaEmarginata
NeomillspaughiaEmarginata 93307 93740 96_rpl2_Exon_NeomillspaughiaEmarginata
NeomillspaughiaEmarginata 93741 94405 97_rpl2_Intron_NeomillspaughiaEmarginata
NeomillspaughiaEmarginata 94406 94796 98_rpl2_Exon_NeomillspaughiaEmarginata
NeomillspaughiaEmarginata 94815 95093 99_rpl23_Exon_NeomillspaughiaEmarginata
NeomillspaughiaEmarginata 95303 95376 100_trnM-CAU_tRNA_NeomillspaughiaEmarginata
NeomillspaughiaEmarginata 95813 102119 101_ycf2_Exon_NeomillspaughiaEmarginata
NeomillspaughiaEmarginata 102975 103055 102_trnL-CAA_tRNA_NeomillspaughiaEmarginata
NeomillspaughiaEmarginata 103623 104378 103_ndhB_Exon_NeomillspaughiaEmarginata
NeomillspaughiaEmarginata 104379 105057 104_ndhB_Intron_NeomillspaughiaEmarginata
NeomillspaughiaEmarginata 105058 105834 105_ndhB_Exon_NeomillspaughiaEmarginata
NeomillspaughiaEmarginata 106167 106634 106_rps7_Exon_NeomillspaughiaEmarginata
NeomillspaughiaEmarginata 106688 106714 107_rps12_Exon_NeomillspaughiaEmarginata
NeomillspaughiaEmarginata 106715 107241 108_rps12_Intron_NeomillspaughiaEmarginata
NeomillspaughiaEmarginata 107242 107472 109_rps12_Exon_NeomillspaughiaEmarginata
NeomillspaughiaEmarginata 109315 109386 110_trnV-GAC_tRNA_NeomillspaughiaEmarginata
NeomillspaughiaEmarginata 109614 111104 111_rrn16_rRNA_NeomillspaughiaEmarginata
NeomillspaughiaEmarginata 111398 111485 112_trnI-GAU_tRNA_NeomillspaughiaEmarginata
NeomillspaughiaEmarginata 111434 111449 113_trnI-GAU_Intron_NeomillspaughiaEmarginata
NeomillspaughiaEmarginata 112492 112596 114_trnA-UGC_tRNA_NeomillspaughiaEmarginata
NeomillspaughiaEmarginata 112529 112568 115_trnA-UGC_Intron_NeomillspaughiaEmarginata
NeomillspaughiaEmarginata 113525 113836 116_rrn23-fragment_rRNA_NeomillspaughiaEmarginata
NeomillspaughiaEmarginata 113844 116336 117_rrn23_rRNA_NeomillspaughiaEmarginata
NeomillspaughiaEmarginata 116435 116537 118_rrn4.5_rRNA_NeomillspaughiaEmarginata
NeomillspaughiaEmarginata 116788 116908 119_rrn5_rRNA_NeomillspaughiaEmarginata
NeomillspaughiaEmarginata 117168 117241 120_trnR-ACG_tRNA_NeomillspaughiaEmarginata
NeomillspaughiaEmarginata 117863 117934 121_trnN-GUU_tRNA_NeomillspaughiaEmarginata
NeomillspaughiaEmarginata 118499 124226 122_ycf1_Exon_NeomillspaughiaEmarginata
NeomillspaughiaEmarginata 124523 124795 123_rps15_Exon_NeomillspaughiaEmarginata
NeomillspaughiaEmarginata 124895 126075 124_ndhH_Exon_NeomillspaughiaEmarginata
NeomillspaughiaEmarginata 126077 126627 125_ndhA_Exon_NeomillspaughiaEmarginata
NeomillspaughiaEmarginata 126628 127761 126_ndhA_Intron_NeomillspaughiaEmarginata
NeomillspaughiaEmarginata 127762 128302 127_ndhA_Exon_NeomillspaughiaEmarginata
NeomillspaughiaEmarginata 128402 128890 128_ndhI_Exon_NeomillspaughiaEmarginata
NeomillspaughiaEmarginata 129267 129797 129_ndhG_Exon_NeomillspaughiaEmarginata
NeomillspaughiaEmarginata 130024 130329 130_ndhE_Exon_NeomillspaughiaEmarginata
NeomillspaughiaEmarginata 130584 130829 131_psaC_Exon_NeomillspaughiaEmarginata
NeomillspaughiaEmarginata 130969 132471 132_ndhD_Exon_NeomillspaughiaEmarginata
NeomillspaughiaEmarginata 132689 133641 133_ccsA_Exon_NeomillspaughiaEmarginata
NeomillspaughiaEmarginata 133783 133862 134_trnL-UAG_tRNA_NeomillspaughiaEmarginata
NeomillspaughiaEmarginata 135161 135334 135_rpl32_Exon_NeomillspaughiaEmarginata
NeomillspaughiaEmarginata 136405 138594 136_ndhF_Exon_NeomillspaughiaEmarginata
NeomillspaughiaEmarginata 138830 144557 137_ycf1_Exon_NeomillspaughiaEmarginata
NeomillspaughiaEmarginata 145122 145193 138_trnN-GUU_tRNA_NeomillspaughiaEmarginata
NeomillspaughiaEmarginata 145815 145888 139_trnR-ACG_tRNA_NeomillspaughiaEmarginata
NeomillspaughiaEmarginata 146148 146268 140_rrn5_rRNA_NeomillspaughiaEmarginata
NeomillspaughiaEmarginata 146519 146621 141_rrn4.5_rRNA_NeomillspaughiaEmarginata
NeomillspaughiaEmarginata 146720 149212 142_rrn23_rRNA_NeomillspaughiaEmarginata
NeomillspaughiaEmarginata 149220 149531 143_rrn23-fragment_rRNA_NeomillspaughiaEmarginata
NeomillspaughiaEmarginata 150460 150564 144_trnA-UGC_tRNA_NeomillspaughiaEmarginata
NeomillspaughiaEmarginata 150488 150527 145_trnA-UGC_Intron_NeomillspaughiaEmarginata
NeomillspaughiaEmarginata 151571 151658 146_trnI-GAU_tRNA_NeomillspaughiaEmarginata
NeomillspaughiaEmarginata 151607 151622 147_trnI-GAU_Intron_NeomillspaughiaEmarginata
NeomillspaughiaEmarginata 151952 153442 148_rrn16_rRNA_NeomillspaughiaEmarginata
NeomillspaughiaEmarginata 153670 153741 149_trnV-GAC_tRNA_NeomillspaughiaEmarginata
NeomillspaughiaEmarginata 155584 155814 150_rps12_Exon_NeomillspaughiaEmarginata
NeomillspaughiaEmarginata 155815 156341 151_rps12_Intron_NeomillspaughiaEmarginata
NeomillspaughiaEmarginata 156342 156368 152_rps12_Exon_NeomillspaughiaEmarginata
NeomillspaughiaEmarginata 156422 156889 153_rps7_Exon_NeomillspaughiaEmarginata
NeomillspaughiaEmarginata 157222 157998 154_ndhB_Exon_NeomillspaughiaEmarginata
NeomillspaughiaEmarginata 157999 158677 155_ndhB_Intron_NeomillspaughiaEmarginata
NeomillspaughiaEmarginata 158678 159433 156_ndhB_Exon_NeomillspaughiaEmarginata
NeomillspaughiaEmarginata 160001 160081 157_trnL-CAA_tRNA_NeomillspaughiaEmarginata
NeomillspaughiaEmarginata 160937 167243 158_ycf2_Exon_NeomillspaughiaEmarginata
NeomillspaughiaEmarginata 167680 167753 159_trnM-CAU_tRNA_NeomillspaughiaEmarginata
NeomillspaughiaEmarginata 167963 168241 160_rpl23_Exon_NeomillspaughiaEmarginata
NeomillspaughiaEmarginata 168260 168650 161_rpl2_Exon_NeomillspaughiaEmarginata
NeomillspaughiaEmarginata 168651 169315 162_rpl2_Intron_NeomillspaughiaEmarginata
NeomillspaughiaEmarginata 169316 169749 163_rpl2_Exon_NeomillspaughiaEmarginata
NeomillspaughiaEmarginata 169808 169915 164_rps19-fragment_Exon_NeomillspaughiaEmarginata
PodopterusMexicanus 3 76 1_trnH-GUG_tRNA_PodopterusMexicanus
PodopterusMexicanus 611 1672 2_psbA_Exon_PodopterusMexicanus
PodopterusMexicanus 2214 3730 3_matK_Exon_PodopterusMexicanus
PodopterusMexicanus 5698 5928 4_rps16_Exon_PodopterusMexicanus
PodopterusMexicanus 5929 6819 5_rps16_Intron_PodopterusMexicanus
PodopterusMexicanus 6820 6855 6_rps16_Exon_PodopterusMexicanus
PodopterusMexicanus 8646 8717 7_trnQ-UUG_tRNA_PodopterusMexicanus
PodopterusMexicanus 9045 9230 8_psbK_Exon_PodopterusMexicanus
PodopterusMexicanus 9652 9762 9_psbI_Exon_PodopterusMexicanus
PodopterusMexicanus 9888 9975 10_trnS-GCU_tRNA_PodopterusMexicanus
PodopterusMexicanus 12411 12482 11_trnR-UCU_tRNA_PodopterusMexicanus
PodopterusMexicanus 13192 14715 12_atpA_Exon_PodopterusMexicanus
PodopterusMexicanus 14788 15198 13_atpF_Exon_PodopterusMexicanus
PodopterusMexicanus 15199 15952 14_atpF_Intron_PodopterusMexicanus
PodopterusMexicanus 15953 16096 15_atpF_Exon_PodopterusMexicanus
PodopterusMexicanus 16545 16790 16_atpH_Exon_PodopterusMexicanus
PodopterusMexicanus 17471 18214 17_atpI_Exon_PodopterusMexicanus
PodopterusMexicanus 18438 19148 18_rps2_Exon_PodopterusMexicanus
PodopterusMexicanus 19401 23475 19_rpoC2_Exon_PodopterusMexicanus
PodopterusMexicanus 23665 25267 20_rpoC1_Exon_PodopterusMexicanus
PodopterusMexicanus 25268 26033 21_rpoC1_Intron_PodopterusMexicanus
PodopterusMexicanus 26034 26463 22_rpoC1_Exon_PodopterusMexicanus
PodopterusMexicanus 26490 29702 23_rpoB_Exon_PodopterusMexicanus
PodopterusMexicanus 31094 31165 24_trnC-GCA_tRNA_PodopterusMexicanus
PodopterusMexicanus 32077 32166 25_petN_Exon_PodopterusMexicanus
PodopterusMexicanus 33299 33403 26_psbM_Exon_PodopterusMexicanus
PodopterusMexicanus 34353 34426 27_trnD-GUC_tRNA_PodopterusMexicanus
PodopterusMexicanus 34850 34933 28_trnY-GUA_tRNA_PodopterusMexicanus
PodopterusMexicanus 35005 35077 29_trnE-UUC_tRNA_PodopterusMexicanus
PodopterusMexicanus 36228 36299 30_trnT-GGU_tRNA_PodopterusMexicanus
PodopterusMexicanus 37674 38735 31_psbD_Exon_PodopterusMexicanus
PodopterusMexicanus 38719 40104 32_psbC_Exon_PodopterusMexicanus
PodopterusMexicanus 40393 40480 33_trnS-UGA_tRNA_PodopterusMexicanus
PodopterusMexicanus 40839 41027 34_psbZ_Exon_PodopterusMexicanus
PodopterusMexicanus 41336 41406 35_trnG-GCC_tRNA_PodopterusMexicanus
PodopterusMexicanus 41574 41647 36_trnM-CAU_tRNA_PodopterusMexicanus
PodopterusMexicanus 41803 42105 37_rps14_Exon_PodopterusMexicanus
PodopterusMexicanus 42231 44435 38_psaB_Exon_PodopterusMexicanus
PodopterusMexicanus 44461 46713 39_psaA_Exon_PodopterusMexicanus
PodopterusMexicanus 47434 47588 40_pafI_Exon_PodopterusMexicanus
PodopterusMexicanus 47589 48348 41_pafI_Intron_PodopterusMexicanus
PodopterusMexicanus 48349 48574 42_pafI_Exon_PodopterusMexicanus
PodopterusMexicanus 48575 49300 43_pafI_Intron_PodopterusMexicanus
PodopterusMexicanus 49301 49426 44_pafI_Exon_PodopterusMexicanus
PodopterusMexicanus 50361 50447 45_trnS-GGA_tRNA_PodopterusMexicanus
PodopterusMexicanus 50788 51384 46_rps4_Exon_PodopterusMexicanus
PodopterusMexicanus 52678 52750 47_trnT-UGU_tRNA_PodopterusMexicanus
PodopterusMexicanus 54720 54792 48_trnF-GAA_tRNA_PodopterusMexicanus
PodopterusMexicanus 55493 55969 49_ndhJ_Exon_PodopterusMexicanus
PodopterusMexicanus 56082 56759 50_ndhK_Exon_PodopterusMexicanus
PodopterusMexicanus 56819 57181 51_ndhC_Exon_PodopterusMexicanus
PodopterusMexicanus 59385 59457 52_trnM-CAU_tRNA_PodopterusMexicanus
PodopterusMexicanus 59982 60383 53_atpE_Exon_PodopterusMexicanus
PodopterusMexicanus 60380 61876 54_atpB_Exon_PodopterusMexicanus
PodopterusMexicanus 62721 64148 55_rbcL_Exon_PodopterusMexicanus
PodopterusMexicanus 64806 66230 56_accD_Exon_PodopterusMexicanus
PodopterusMexicanus 66976 67076 57_psaI_Exon_PodopterusMexicanus
PodopterusMexicanus 67517 68071 58_pafII_Exon_PodopterusMexicanus
PodopterusMexicanus 69000 69689 59_cemA_Exon_PodopterusMexicanus
PodopterusMexicanus 69910 70872 60_petA_Exon_PodopterusMexicanus
PodopterusMexicanus 71772 71894 61_psbJ_Exon_PodopterusMexicanus
PodopterusMexicanus 72027 72143 62_psbL_Exon_PodopterusMexicanus
PodopterusMexicanus 72171 72290 63_psbF_Exon_PodopterusMexicanus
PodopterusMexicanus 72299 72550 64_psbE_Exon_PodopterusMexicanus
PodopterusMexicanus 73842 73937 65_petL_Exon_PodopterusMexicanus
PodopterusMexicanus 74126 74239 66_petG_Exon_PodopterusMexicanus
PodopterusMexicanus 74364 74437 67_trnW-CCA_tRNA_PodopterusMexicanus
PodopterusMexicanus 74925 74998 68_trnP-UGG_tRNA_PodopterusMexicanus
PodopterusMexicanus 75433 75559 69_psaJ_Exon_PodopterusMexicanus
PodopterusMexicanus 76064 76264 70_rpl33_Exon_PodopterusMexicanus
PodopterusMexicanus 76774 77064 71_rps18_Exon_PodopterusMexicanus
PodopterusMexicanus 77314 77706 72_rpl20_Exon_PodopterusMexicanus
PodopterusMexicanus 78481 78594 73_rps12_Exon_PodopterusMexicanus
PodopterusMexicanus 78481 78594 74_rps12_Exon_PodopterusMexicanus
PodopterusMexicanus 78777 79001 75_clpP1_Exon_PodopterusMexicanus
PodopterusMexicanus 79002 79639 76_clpP1_Intron_PodopterusMexicanus
PodopterusMexicanus 79640 79931 77_clpP1_Exon_PodopterusMexicanus
PodopterusMexicanus 79932 81074 78_clpP1_Intron_PodopterusMexicanus
PodopterusMexicanus 81075 81145 79_clpP1_Exon_PodopterusMexicanus
PodopterusMexicanus 81587 83113 80_psbB_Exon_PodopterusMexicanus
PodopterusMexicanus 83284 83385 81_psbT_Exon_PodopterusMexicanus
PodopterusMexicanus 83457 83588 82_pbf1_Exon_PodopterusMexicanus
PodopterusMexicanus 83691 83912 83_psbH_Exon_PodopterusMexicanus
PodopterusMexicanus 84813 85458 84_petB_Exon_PodopterusMexicanus
PodopterusMexicanus 86441 86919 85_petD_Exon_PodopterusMexicanus
PodopterusMexicanus 87126 88119 86_rpoA_Exon_PodopterusMexicanus
PodopterusMexicanus 88191 88607 87_rps11_Exon_PodopterusMexicanus
PodopterusMexicanus 88757 88870 88_rpl36_Exon_PodopterusMexicanus
PodopterusMexicanus 88979 89212 89_infA_Exon_PodopterusMexicanus
PodopterusMexicanus 89331 89735 90_rps8_Exon_PodopterusMexicanus
PodopterusMexicanus 89917 90285 91_rpl14_Exon_PodopterusMexicanus
PodopterusMexicanus 90408 90810 92_rpl16_Exon_PodopterusMexicanus
PodopterusMexicanus 92570 93226 93_rps3_Exon_PodopterusMexicanus
PodopterusMexicanus 93289 93655 94_rpl22_Exon_PodopterusMexicanus
PodopterusMexicanus 93714 93992 95_rps19_Exon_PodopterusMexicanus
PodopterusMexicanus 94052 94485 96_rpl2_Exon_PodopterusMexicanus
PodopterusMexicanus 94486 95150 97_rpl2_Intron_PodopterusMexicanus
PodopterusMexicanus 95151 95541 98_rpl2_Exon_PodopterusMexicanus
PodopterusMexicanus 95560 95838 99_rpl23_Exon_PodopterusMexicanus
PodopterusMexicanus 96048 96121 100_trnM-CAU_tRNA_PodopterusMexicanus
PodopterusMexicanus 96559 102865 101_ycf2_Exon_PodopterusMexicanus
PodopterusMexicanus 103720 103800 102_trnL-CAA_tRNA_PodopterusMexicanus
PodopterusMexicanus 104368 105123 103_ndhB_Exon_PodopterusMexicanus
PodopterusMexicanus 105124 105802 104_ndhB_Intron_PodopterusMexicanus
PodopterusMexicanus 105803 106579 105_ndhB_Exon_PodopterusMexicanus
PodopterusMexicanus 106912 107379 106_rps7_Exon_PodopterusMexicanus
PodopterusMexicanus 107433 107459 107_rps12_Exon_PodopterusMexicanus
PodopterusMexicanus 107460 107986 108_rps12_Intron_PodopterusMexicanus
PodopterusMexicanus 107987 108217 109_rps12_Exon_PodopterusMexicanus
PodopterusMexicanus 110055 110126 110_trnV-GAC_tRNA_PodopterusMexicanus
PodopterusMexicanus 110354 111844 111_rrn16_rRNA_PodopterusMexicanus
PodopterusMexicanus 112138 112225 112_trnI-GAU_tRNA_PodopterusMexicanus
PodopterusMexicanus 112174 112189 113_trnI-GAU_Intron_PodopterusMexicanus
PodopterusMexicanus 113223 113327 114_trnA-UGC_tRNA_PodopterusMexicanus
PodopterusMexicanus 113260 113299 115_trnA-UGC_Intron_PodopterusMexicanus
PodopterusMexicanus 114256 114567 116_rrn23-fragment_rRNA_PodopterusMexicanus
PodopterusMexicanus 114575 117067 117_rrn23_rRNA_PodopterusMexicanus
PodopterusMexicanus 117166 117268 118_rrn4.5_rRNA_PodopterusMexicanus
PodopterusMexicanus 117520 117640 119_rrn5_rRNA_PodopterusMexicanus
PodopterusMexicanus 117900 117973 120_trnR-ACG_tRNA_PodopterusMexicanus
PodopterusMexicanus 118593 118664 121_trnN-GUU_tRNA_PodopterusMexicanus
PodopterusMexicanus 119235 124956 122_ycf1_Exon_PodopterusMexicanus
PodopterusMexicanus 125184 127394 123_ndhF_Exon_PodopterusMexicanus
PodopterusMexicanus 128459 128632 124_rpl32_Exon_PodopterusMexicanus
PodopterusMexicanus 129678 129757 125_trnL-UAG_tRNA_PodopterusMexicanus
PodopterusMexicanus 129906 130858 126_ccsA_Exon_PodopterusMexicanus
PodopterusMexicanus 131074 132576 127_ndhD_Exon_PodopterusMexicanus
PodopterusMexicanus 132716 132961 128_psaC_Exon_PodopterusMexicanus
PodopterusMexicanus 133215 133520 129_ndhE_Exon_PodopterusMexicanus
PodopterusMexicanus 133747 134277 130_ndhG_Exon_PodopterusMexicanus
PodopterusMexicanus 134647 135135 131_ndhI_Exon_PodopterusMexicanus
PodopterusMexicanus 135235 135775 132_ndhA_Exon_PodopterusMexicanus
PodopterusMexicanus 135776 136902 133_ndhA_Intron_PodopterusMexicanus
PodopterusMexicanus 136903 137453 134_ndhA_Exon_PodopterusMexicanus
PodopterusMexicanus 137455 138635 135_ndhH_Exon_PodopterusMexicanus
PodopterusMexicanus 138735 139007 136_rps15_Exon_PodopterusMexicanus
PodopterusMexicanus 139328 145049 137_ycf1-fragment_Exon_PodopterusMexicanus
PodopterusMexicanus 145620 145691 138_trnN-GUU_tRNA_PodopterusMexicanus
PodopterusMexicanus 146311 146384 139_trnR-ACG_tRNA_PodopterusMexicanus
PodopterusMexicanus 146644 146764 140_rrn5_rRNA_PodopterusMexicanus
PodopterusMexicanus 147016 147118 141_rrn4.5_rRNA_PodopterusMexicanus
PodopterusMexicanus 147217 149709 142_rrn23_rRNA_PodopterusMexicanus
PodopterusMexicanus 149717 150028 143_rrn23-fragment_rRNA_PodopterusMexicanus
PodopterusMexicanus 150957 151061 144_trnA-UGC_tRNA_PodopterusMexicanus
PodopterusMexicanus 150985 151024 145_trnA-UGC_Intron_PodopterusMexicanus
PodopterusMexicanus 152059 152146 146_trnI-GAU_tRNA_PodopterusMexicanus
PodopterusMexicanus 152095 152110 147_trnI-GAU_Intron_PodopterusMexicanus
PodopterusMexicanus 152440 153930 148_rrn16_rRNA_PodopterusMexicanus
PodopterusMexicanus 154158 154229 149_trnV-GAC_tRNA_PodopterusMexicanus
PodopterusMexicanus 156067 156297 150_rps12_Exon_PodopterusMexicanus
PodopterusMexicanus 156298 156824 151_rps12_Intron_PodopterusMexicanus
PodopterusMexicanus 156825 156851 152_rps12_Exon_PodopterusMexicanus
PodopterusMexicanus 156905 157372 153_rps7_Exon_PodopterusMexicanus
PodopterusMexicanus 157705 158481 154_ndhB_Exon_PodopterusMexicanus
PodopterusMexicanus 158482 159160 155_ndhB_Intron_PodopterusMexicanus
PodopterusMexicanus 159161 159916 156_ndhB_Exon_PodopterusMexicanus
PodopterusMexicanus 160484 160564 157_trnL-CAA_tRNA_PodopterusMexicanus
PodopterusMexicanus 161419 167725 158_ycf2_Exon_PodopterusMexicanus
PodopterusMexicanus 168163 168236 159_trnM-CAU_tRNA_PodopterusMexicanus
PodopterusMexicanus 168446 168724 160_rpl23_Exon_PodopterusMexicanus
PodopterusMexicanus 168743 169133 161_rpl2_Exon_PodopterusMexicanus
PodopterusMexicanus 169134 169798 162_rpl2_Intron_PodopterusMexicanus
PodopterusMexicanus 169799 170232 163_rpl2_Exon_PodopterusMexicanus
PodopterusMexicanus 170292 170399 164_rps19-fragment_Exon_PodopterusMexicanus
RuprechtiaCoriacea 4 77 1_trnH-GUG_tRNA_RuprechtiaCoriacea
RuprechtiaCoriacea 494 1555 2_psbA_Exon_RuprechtiaCoriacea
RuprechtiaCoriacea 2085 3604 3_matK_Exon_RuprechtiaCoriacea
RuprechtiaCoriacea 5523 5735 4_rps16_Exon_RuprechtiaCoriacea
RuprechtiaCoriacea 5736 6608 5_rps16_Intron_RuprechtiaCoriacea
RuprechtiaCoriacea 6609 6644 6_rps16_Exon_RuprechtiaCoriacea
RuprechtiaCoriacea 8409 8480 7_trnQ-UUG_tRNA_RuprechtiaCoriacea
RuprechtiaCoriacea 8817 9002 8_psbK_Exon_RuprechtiaCoriacea
RuprechtiaCoriacea 9395 9505 9_psbI_Exon_RuprechtiaCoriacea
RuprechtiaCoriacea 9629 9716 10_trnS-GCU_tRNA_RuprechtiaCoriacea
RuprechtiaCoriacea 12220 12291 11_trnR-UCU_tRNA_RuprechtiaCoriacea
RuprechtiaCoriacea 12915 14438 12_atpA_Exon_RuprechtiaCoriacea
RuprechtiaCoriacea 14510 14920 13_atpF_Exon_RuprechtiaCoriacea
RuprechtiaCoriacea 14921 15665 14_atpF_Intron_RuprechtiaCoriacea
RuprechtiaCoriacea 15666 15809 15_atpF_Exon_RuprechtiaCoriacea
RuprechtiaCoriacea 16252 16497 16_atpH_Exon_RuprechtiaCoriacea
RuprechtiaCoriacea 17185 17928 17_atpI_Exon_RuprechtiaCoriacea
RuprechtiaCoriacea 18140 18850 18_rps2_Exon_RuprechtiaCoriacea
RuprechtiaCoriacea 19142 23216 19_rpoC2_Exon_RuprechtiaCoriacea
RuprechtiaCoriacea 23406 25006 20_rpoC1_Exon_RuprechtiaCoriacea
RuprechtiaCoriacea 25007 25782 21_rpoC1_Intron_RuprechtiaCoriacea
RuprechtiaCoriacea 25783 26214 22_rpoC1_Exon_RuprechtiaCoriacea
RuprechtiaCoriacea 26241 29453 23_rpoB_Exon_RuprechtiaCoriacea
RuprechtiaCoriacea 30858 30929 24_trnC-GCA_tRNA_RuprechtiaCoriacea
RuprechtiaCoriacea 31853 31942 25_petN_Exon_RuprechtiaCoriacea
RuprechtiaCoriacea 33078 33182 26_psbM_Exon_RuprechtiaCoriacea
RuprechtiaCoriacea 34362 34435 27_trnD-GUC_tRNA_RuprechtiaCoriacea
RuprechtiaCoriacea 34870 34953 28_trnY-GUA_tRNA_RuprechtiaCoriacea
RuprechtiaCoriacea 35018 35090 29_trnE-UUC_tRNA_RuprechtiaCoriacea
RuprechtiaCoriacea 36245 36316 30_trnT-GGU_tRNA_RuprechtiaCoriacea
RuprechtiaCoriacea 37664 38725 31_psbD_Exon_RuprechtiaCoriacea
RuprechtiaCoriacea 38709 40094 32_psbC_Exon_RuprechtiaCoriacea
RuprechtiaCoriacea 40363 40450 33_trnS-UGA_tRNA_RuprechtiaCoriacea
RuprechtiaCoriacea 40805 40993 34_psbZ_Exon_RuprechtiaCoriacea
RuprechtiaCoriacea 41302 41372 35_trnG-GCC_tRNA_RuprechtiaCoriacea
RuprechtiaCoriacea 41539 41612 36_trnM-CAU_tRNA_RuprechtiaCoriacea
RuprechtiaCoriacea 41761 42063 37_rps14_Exon_RuprechtiaCoriacea
RuprechtiaCoriacea 42189 44393 38_psaB_Exon_RuprechtiaCoriacea
RuprechtiaCoriacea 44419 46671 39_psaA_Exon_RuprechtiaCoriacea
RuprechtiaCoriacea 47386 47540 40_pafI_Exon_RuprechtiaCoriacea
RuprechtiaCoriacea 47541 48288 41_pafI_Intron_RuprechtiaCoriacea
RuprechtiaCoriacea 48289 48514 42_pafI_Exon_RuprechtiaCoriacea
RuprechtiaCoriacea 48515 49244 43_pafI_Intron_RuprechtiaCoriacea
RuprechtiaCoriacea 49245 49370 44_pafI_Exon_RuprechtiaCoriacea
RuprechtiaCoriacea 50307 50393 45_trnS-GGA_tRNA_RuprechtiaCoriacea
RuprechtiaCoriacea 50708 51304 46_rps4_Exon_RuprechtiaCoriacea
RuprechtiaCoriacea 52389 52461 47_trnT-UGU_tRNA_RuprechtiaCoriacea
RuprechtiaCoriacea 54429 54501 48_trnF-GAA_tRNA_RuprechtiaCoriacea
RuprechtiaCoriacea 55236 55712 49_ndhJ_Exon_RuprechtiaCoriacea
RuprechtiaCoriacea 55821 56498 50_ndhK_Exon_RuprechtiaCoriacea
RuprechtiaCoriacea 56558 56920 51_ndhC_Exon_RuprechtiaCoriacea
RuprechtiaCoriacea 59112 59184 52_trnM-CAU_tRNA_RuprechtiaCoriacea
RuprechtiaCoriacea 59677 60078 53_atpE_Exon_RuprechtiaCoriacea
RuprechtiaCoriacea 60075 61571 54_atpB_Exon_RuprechtiaCoriacea
RuprechtiaCoriacea 62570 63997 55_rbcL_Exon_RuprechtiaCoriacea
RuprechtiaCoriacea 64658 66082 56_accD_Exon_RuprechtiaCoriacea
RuprechtiaCoriacea 66847 66947 57_psaI_Exon_RuprechtiaCoriacea
RuprechtiaCoriacea 67402 67956 58_pafII_Exon_RuprechtiaCoriacea
RuprechtiaCoriacea 68893 69582 59_cemA_Exon_RuprechtiaCoriacea
RuprechtiaCoriacea 69803 70765 60_petA_Exon_RuprechtiaCoriacea
RuprechtiaCoriacea 71653 71775 61_psbJ_Exon_RuprechtiaCoriacea
RuprechtiaCoriacea 71908 72024 62_psbL_Exon_RuprechtiaCoriacea
RuprechtiaCoriacea 72052 72171 63_psbF_Exon_RuprechtiaCoriacea
RuprechtiaCoriacea 72180 72431 64_psbE_Exon_RuprechtiaCoriacea
RuprechtiaCoriacea 73717 73812 65_petL_Exon_RuprechtiaCoriacea
RuprechtiaCoriacea 73991 74104 66_petG_Exon_RuprechtiaCoriacea
RuprechtiaCoriacea 74224 74297 67_trnW-CCA_tRNA_RuprechtiaCoriacea
RuprechtiaCoriacea 74794 74867 68_trnP-UGG_tRNA_RuprechtiaCoriacea
RuprechtiaCoriacea 75296 75430 69_psaJ_Exon_RuprechtiaCoriacea
RuprechtiaCoriacea 75927 76127 70_rpl33_Exon_RuprechtiaCoriacea
RuprechtiaCoriacea 76848 77138 71_rps18_Exon_RuprechtiaCoriacea
RuprechtiaCoriacea 77401 77793 72_rpl20_Exon_RuprechtiaCoriacea
RuprechtiaCoriacea 78570 78683 73_rps12_Exon_RuprechtiaCoriacea
RuprechtiaCoriacea 78570 78683 74_rps12_Exon_RuprechtiaCoriacea
RuprechtiaCoriacea 78867 79091 75_clpP1_Exon_RuprechtiaCoriacea
RuprechtiaCoriacea 79092 79748 76_clpP1_Intron_RuprechtiaCoriacea
RuprechtiaCoriacea 79749 80040 77_clpP1_Exon_RuprechtiaCoriacea
RuprechtiaCoriacea 80041 81262 78_clpP1_Intron_RuprechtiaCoriacea
RuprechtiaCoriacea 81263 81333 79_clpP1_Exon_RuprechtiaCoriacea
RuprechtiaCoriacea 81779 83305 80_psbB_Exon_RuprechtiaCoriacea
RuprechtiaCoriacea 83476 83577 81_psbT_Exon_RuprechtiaCoriacea
RuprechtiaCoriacea 83649 83780 82_pbf1_Exon_RuprechtiaCoriacea
RuprechtiaCoriacea 83883 84104 83_psbH_Exon_RuprechtiaCoriacea
RuprechtiaCoriacea 84994 85639 84_petB_Exon_RuprechtiaCoriacea
RuprechtiaCoriacea 86618 87096 85_petD_Exon_RuprechtiaCoriacea
RuprechtiaCoriacea 87302 88292 86_rpoA_Exon_RuprechtiaCoriacea
RuprechtiaCoriacea 88359 88775 87_rps11_Exon_RuprechtiaCoriacea
RuprechtiaCoriacea 88919 89032 88_rpl36_Exon_RuprechtiaCoriacea
RuprechtiaCoriacea 89141 89374 89_infA_Exon_RuprechtiaCoriacea
RuprechtiaCoriacea 89493 89897 90_rps8_Exon_RuprechtiaCoriacea
RuprechtiaCoriacea 90109 90477 91_rpl14_Exon_RuprechtiaCoriacea
RuprechtiaCoriacea 90600 91002 92_rpl16_Exon_RuprechtiaCoriacea
RuprechtiaCoriacea 92670 93326 93_rps3_Exon_RuprechtiaCoriacea
RuprechtiaCoriacea 93389 93755 94_rpl22_Exon_RuprechtiaCoriacea
RuprechtiaCoriacea 93814 94092 95_rps19_Exon_RuprechtiaCoriacea
RuprechtiaCoriacea 94151 94584 96_rpl2_Exon_RuprechtiaCoriacea
RuprechtiaCoriacea 94585 95249 97_rpl2_Intron_RuprechtiaCoriacea
RuprechtiaCoriacea 95250 95640 98_rpl2_Exon_RuprechtiaCoriacea
RuprechtiaCoriacea 95659 95937 99_rpl23_Exon_RuprechtiaCoriacea
RuprechtiaCoriacea 96147 96220 100_trnM-CAU_tRNA_RuprechtiaCoriacea
RuprechtiaCoriacea 96617 102926 101_ycf2_Exon_RuprechtiaCoriacea
RuprechtiaCoriacea 103780 103860 102_trnL-CAA_tRNA_RuprechtiaCoriacea
RuprechtiaCoriacea 104426 105181 103_ndhB_Exon_RuprechtiaCoriacea
RuprechtiaCoriacea 105182 105860 104_ndhB_Intron_RuprechtiaCoriacea
RuprechtiaCoriacea 105861 106637 105_ndhB_Exon_RuprechtiaCoriacea
RuprechtiaCoriacea 106970 107437 106_rps7_Exon_RuprechtiaCoriacea
RuprechtiaCoriacea 107491 107517 107_rps12_Exon_RuprechtiaCoriacea
RuprechtiaCoriacea 107518 108044 108_rps12_Intron_RuprechtiaCoriacea
RuprechtiaCoriacea 108045 108275 109_rps12_Exon_RuprechtiaCoriacea
RuprechtiaCoriacea 110106 110177 110_trnV-GAC_tRNA_RuprechtiaCoriacea
RuprechtiaCoriacea 110405 111895 111_rrn16_rRNA_RuprechtiaCoriacea
RuprechtiaCoriacea 112188 112275 112_trnI-GAU_tRNA_RuprechtiaCoriacea
RuprechtiaCoriacea 112224 112239 113_trnI-GAU_Intron_RuprechtiaCoriacea
RuprechtiaCoriacea 113283 113387 114_trnA-UGC_tRNA_RuprechtiaCoriacea
RuprechtiaCoriacea 113320 113359 115_trnA-UGC_Intron_RuprechtiaCoriacea
RuprechtiaCoriacea 114316 114869 116_rrn23-fragment_rRNA_RuprechtiaCoriacea
RuprechtiaCoriacea 114875 117127 117_rrn23_rRNA_RuprechtiaCoriacea
RuprechtiaCoriacea 117226 117328 118_rrn4.5_rRNA_RuprechtiaCoriacea
RuprechtiaCoriacea 117580 117700 119_rrn5_rRNA_RuprechtiaCoriacea
RuprechtiaCoriacea 117962 118035 120_trnR-ACG_tRNA_RuprechtiaCoriacea
RuprechtiaCoriacea 118654 118725 121_trnN-GUU_tRNA_RuprechtiaCoriacea
RuprechtiaCoriacea 119290 125020 122_ycf1_Exon_RuprechtiaCoriacea
RuprechtiaCoriacea 125256 127457 123_ndhF_Exon_RuprechtiaCoriacea
RuprechtiaCoriacea 128484 128657 124_rpl32_Exon_RuprechtiaCoriacea
RuprechtiaCoriacea 129944 130023 125_trnL-UAG_tRNA_RuprechtiaCoriacea
RuprechtiaCoriacea 130147 131099 126_ccsA_Exon_RuprechtiaCoriacea
RuprechtiaCoriacea 131317 132819 127_ndhD_Exon_RuprechtiaCoriacea
RuprechtiaCoriacea 132959 133204 128_psaC_Exon_RuprechtiaCoriacea
RuprechtiaCoriacea 133456 133761 129_ndhE_Exon_RuprechtiaCoriacea
RuprechtiaCoriacea 133997 134527 130_ndhG_Exon_RuprechtiaCoriacea
RuprechtiaCoriacea 134891 135379 131_ndhI_Exon_RuprechtiaCoriacea
RuprechtiaCoriacea 135492 136032 132_ndhA_Exon_RuprechtiaCoriacea
RuprechtiaCoriacea 136033 137171 133_ndhA_Intron_RuprechtiaCoriacea
RuprechtiaCoriacea 137172 137722 134_ndhA_Exon_RuprechtiaCoriacea
RuprechtiaCoriacea 137724 138904 135_ndhH_Exon_RuprechtiaCoriacea
RuprechtiaCoriacea 139004 139276 136_rps15_Exon_RuprechtiaCoriacea
RuprechtiaCoriacea 139606 145336 137_ycf1_Exon_RuprechtiaCoriacea
RuprechtiaCoriacea 145901 145972 138_trnN-GUU_tRNA_RuprechtiaCoriacea
RuprechtiaCoriacea 146591 146664 139_trnR-ACG_tRNA_RuprechtiaCoriacea
RuprechtiaCoriacea 146926 147046 140_rrn5_rRNA_RuprechtiaCoriacea
RuprechtiaCoriacea 147298 147400 141_rrn4.5_rRNA_RuprechtiaCoriacea
RuprechtiaCoriacea 147499 149751 142_rrn23_rRNA_RuprechtiaCoriacea
RuprechtiaCoriacea 149757 150310 143_rrn23-fragment_rRNA_RuprechtiaCoriacea
RuprechtiaCoriacea 151239 151343 144_trnA-UGC_tRNA_RuprechtiaCoriacea
RuprechtiaCoriacea 151267 151306 145_trnA-UGC_Intron_RuprechtiaCoriacea
RuprechtiaCoriacea 152351 152438 146_trnI-GAU_tRNA_RuprechtiaCoriacea
RuprechtiaCoriacea 152387 152402 147_trnI-GAU_Intron_RuprechtiaCoriacea
RuprechtiaCoriacea 152731 154221 148_rrn16_rRNA_RuprechtiaCoriacea
RuprechtiaCoriacea 154449 154520 149_trnV-GAC_tRNA_RuprechtiaCoriacea
RuprechtiaCoriacea 156351 156581 150_rps12_Exon_RuprechtiaCoriacea
RuprechtiaCoriacea 156582 157108 151_rps12_Intron_RuprechtiaCoriacea
RuprechtiaCoriacea 157109 157135 152_rps12_Exon_RuprechtiaCoriacea
RuprechtiaCoriacea 157189 157656 153_rps7_Exon_RuprechtiaCoriacea
RuprechtiaCoriacea 157989 158765 154_ndhB_Exon_RuprechtiaCoriacea
RuprechtiaCoriacea 158766 159444 155_ndhB_Intron_RuprechtiaCoriacea
RuprechtiaCoriacea 159445 160200 156_ndhB_Exon_RuprechtiaCoriacea
RuprechtiaCoriacea 160766 160846 157_trnL-CAA_tRNA_RuprechtiaCoriacea
RuprechtiaCoriacea 161700 168009 158_ycf2_Exon_RuprechtiaCoriacea
RuprechtiaCoriacea 168406 168479 159_trnM-CAU_tRNA_RuprechtiaCoriacea
RuprechtiaCoriacea 168689 168967 160_rpl23_Exon_RuprechtiaCoriacea
RuprechtiaCoriacea 168986 169376 161_rpl2_Exon_RuprechtiaCoriacea
RuprechtiaCoriacea 169377 170041 162_rpl2_Intron_RuprechtiaCoriacea
RuprechtiaCoriacea 170042 170475 163_rpl2_Exon_RuprechtiaCoriacea
RuprechtiaCoriacea 170534 170640 164_rps19-fragment_Exon_RuprechtiaCoriacea
TriplarisCumingiana 3 76 1_trnH-GUG_tRNA_TriplarisCumingiana
TriplarisCumingiana 784 1845 2_psbA_Exon_TriplarisCumingiana
TriplarisCumingiana 2376 3895 3_matK_Exon_TriplarisCumingiana
TriplarisCumingiana 5834 6046 4_rps16_Exon_TriplarisCumingiana
TriplarisCumingiana 6047 6923 5_rps16_Intron_TriplarisCumingiana
TriplarisCumingiana 6924 6959 6_rps16_Exon_TriplarisCumingiana
TriplarisCumingiana 8810 8881 7_trnQ-UUG_tRNA_TriplarisCumingiana
TriplarisCumingiana 9225 9410 8_psbK_Exon_TriplarisCumingiana
TriplarisCumingiana 9803 9913 9_psbI_Exon_TriplarisCumingiana
TriplarisCumingiana 10050 10137 10_trnS-GCU_tRNA_TriplarisCumingiana
TriplarisCumingiana 12611 12682 11_trnR-UCU_tRNA_TriplarisCumingiana
TriplarisCumingiana 13462 14985 12_atpA_Exon_TriplarisCumingiana
TriplarisCumingiana 15057 15467 13_atpF_Exon_TriplarisCumingiana
TriplarisCumingiana 15468 16222 14_atpF_Intron_TriplarisCumingiana
TriplarisCumingiana 16223 16366 15_atpF_Exon_TriplarisCumingiana
TriplarisCumingiana 16809 17054 16_atpH_Exon_TriplarisCumingiana
TriplarisCumingiana 17744 18487 17_atpI_Exon_TriplarisCumingiana
TriplarisCumingiana 18708 19418 18_rps2_Exon_TriplarisCumingiana
TriplarisCumingiana 19712 23786 19_rpoC2_Exon_TriplarisCumingiana
TriplarisCumingiana 23976 25578 20_rpoC1_Exon_TriplarisCumingiana
TriplarisCumingiana 25579 26354 21_rpoC1_Intron_TriplarisCumingiana
TriplarisCumingiana 26355 26784 22_rpoC1_Exon_TriplarisCumingiana
TriplarisCumingiana 26811 30023 23_rpoB_Exon_TriplarisCumingiana
TriplarisCumingiana 31437 31508 24_trnC-GCA_tRNA_TriplarisCumingiana
TriplarisCumingiana 32440 32529 25_petN_Exon_TriplarisCumingiana
TriplarisCumingiana 33668 33772 26_psbM_Exon_TriplarisCumingiana
TriplarisCumingiana 34954 35027 27_trnD-GUC_tRNA_TriplarisCumingiana
TriplarisCumingiana 35462 35545 28_trnY-GUA_tRNA_TriplarisCumingiana
TriplarisCumingiana 35610 35682 29_trnE-UUC_tRNA_TriplarisCumingiana
TriplarisCumingiana 36813 36884 30_trnT-GGU_tRNA_TriplarisCumingiana
TriplarisCumingiana 38228 39289 31_psbD_Exon_TriplarisCumingiana
TriplarisCumingiana 39273 40658 32_psbC_Exon_TriplarisCumingiana
TriplarisCumingiana 40931 41018 33_trnS-UGA_tRNA_TriplarisCumingiana
TriplarisCumingiana 41374 41562 34_psbZ_Exon_TriplarisCumingiana
TriplarisCumingiana 41871 41941 35_trnG-GCC_tRNA_TriplarisCumingiana
TriplarisCumingiana 42108 42181 36_trnM-CAU_tRNA_TriplarisCumingiana
TriplarisCumingiana 42330 42632 37_rps14_Exon_TriplarisCumingiana
TriplarisCumingiana 42758 44962 38_psaB_Exon_TriplarisCumingiana
TriplarisCumingiana 44988 47240 39_psaA_Exon_TriplarisCumingiana
TriplarisCumingiana 47966 48120 40_pafI_Exon_TriplarisCumingiana
TriplarisCumingiana 48121 48870 41_pafI_Intron_TriplarisCumingiana
TriplarisCumingiana 48871 49096 42_pafI_Exon_TriplarisCumingiana
TriplarisCumingiana 49097 49836 43_pafI_Intron_TriplarisCumingiana
TriplarisCumingiana 49837 49962 44_pafI_Exon_TriplarisCumingiana
TriplarisCumingiana 50898 50984 45_trnS-GGA_tRNA_TriplarisCumingiana
TriplarisCumingiana 51336 51932 46_rps4_Exon_TriplarisCumingiana
TriplarisCumingiana 53089 53161 47_trnT-UGU_tRNA_TriplarisCumingiana
TriplarisCumingiana 55166 55238 48_trnF-GAA_tRNA_TriplarisCumingiana
TriplarisCumingiana 55986 56462 49_ndhJ_Exon_TriplarisCumingiana
TriplarisCumingiana 56571 57248 50_ndhK_Exon_TriplarisCumingiana
TriplarisCumingiana 57308 57670 51_ndhC_Exon_TriplarisCumingiana
TriplarisCumingiana 59567 59639 52_trnM-CAU_tRNA_TriplarisCumingiana
TriplarisCumingiana 60348 60749 53_atpE_Exon_TriplarisCumingiana
TriplarisCumingiana 60746 62242 54_atpB_Exon_TriplarisCumingiana
TriplarisCumingiana 63106 64533 55_rbcL_Exon_TriplarisCumingiana
TriplarisCumingiana 65194 66618 56_accD_Exon_TriplarisCumingiana
TriplarisCumingiana 67383 67483 57_psaI_Exon_TriplarisCumingiana
TriplarisCumingiana 67938 68492 58_pafII_Exon_TriplarisCumingiana
TriplarisCumingiana 69442 70131 59_cemA_Exon_TriplarisCumingiana
TriplarisCumingiana 70352 71314 60_petA_Exon_TriplarisCumingiana
TriplarisCumingiana 72199 72321 61_psbJ_Exon_TriplarisCumingiana
TriplarisCumingiana 72454 72570 62_psbL_Exon_TriplarisCumingiana
TriplarisCumingiana 72598 72717 63_psbF_Exon_TriplarisCumingiana
TriplarisCumingiana 72726 72977 64_psbE_Exon_TriplarisCumingiana
TriplarisCumingiana 74265 74360 65_petL_Exon_TriplarisCumingiana
TriplarisCumingiana 74539 74652 66_petG_Exon_TriplarisCumingiana
TriplarisCumingiana 74772 74845 67_trnW-CCA_tRNA_TriplarisCumingiana
TriplarisCumingiana 75416 75489 68_trnP-UGG_tRNA_TriplarisCumingiana
TriplarisCumingiana 75909 76043 69_psaJ_Exon_TriplarisCumingiana
TriplarisCumingiana 76545 76745 70_rpl33_Exon_TriplarisCumingiana
TriplarisCumingiana 77376 77666 71_rps18_Exon_TriplarisCumingiana
TriplarisCumingiana 77929 78321 72_rpl20_Exon_TriplarisCumingiana
TriplarisCumingiana 79103 79216 73_rps12_Exon_TriplarisCumingiana
TriplarisCumingiana 79103 79216 74_rps12_Exon_TriplarisCumingiana
TriplarisCumingiana 79395 79619 75_clpP1_Exon_TriplarisCumingiana
TriplarisCumingiana 79620 80273 76_clpP1_Intron_TriplarisCumingiana
TriplarisCumingiana 80274 80565 77_clpP1_Exon_TriplarisCumingiana
TriplarisCumingiana 80566 81774 78_clpP1_Intron_TriplarisCumingiana
TriplarisCumingiana 81775 81845 79_clpP1_Exon_TriplarisCumingiana
TriplarisCumingiana 82291 83817 80_psbB_Exon_TriplarisCumingiana
TriplarisCumingiana 83988 84089 81_psbT_Exon_TriplarisCumingiana
TriplarisCumingiana 84161 84292 82_pbf1_Exon_TriplarisCumingiana
TriplarisCumingiana 84395 84616 83_psbH_Exon_TriplarisCumingiana
TriplarisCumingiana 85500 86145 84_petB_Exon_TriplarisCumingiana
TriplarisCumingiana 87130 87608 85_petD_Exon_TriplarisCumingiana
TriplarisCumingiana 87813 88806 86_rpoA_Exon_TriplarisCumingiana
TriplarisCumingiana 88873 89289 87_rps11_Exon_TriplarisCumingiana
TriplarisCumingiana 89435 89548 88_rpl36_Exon_TriplarisCumingiana
TriplarisCumingiana 89657 89890 89_infA_Exon_TriplarisCumingiana
TriplarisCumingiana 90009 90413 90_rps8_Exon_TriplarisCumingiana
TriplarisCumingiana 90619 90987 91_rpl14_Exon_TriplarisCumingiana
TriplarisCumingiana 91110 91512 92_rpl16_Exon_TriplarisCumingiana
TriplarisCumingiana 93226 93882 93_rps3_Exon_TriplarisCumingiana
TriplarisCumingiana 93945 94311 94_rpl22_Exon_TriplarisCumingiana
TriplarisCumingiana 94370 94648 95_rps19_Exon_TriplarisCumingiana
TriplarisCumingiana 94707 95140 96_rpl2_Exon_TriplarisCumingiana
TriplarisCumingiana 95141 95805 97_rpl2_Intron_TriplarisCumingiana
TriplarisCumingiana 95806 96196 98_rpl2_Exon_TriplarisCumingiana
TriplarisCumingiana 96215 96493 99_rpl23_Exon_TriplarisCumingiana
TriplarisCumingiana 96703 96776 100_trnM-CAU_tRNA_TriplarisCumingiana
TriplarisCumingiana 97173 103482 101_ycf2_Exon_TriplarisCumingiana
TriplarisCumingiana 104336 104416 102_trnL-CAA_tRNA_TriplarisCumingiana
TriplarisCumingiana 104982 105737 103_ndhB_Exon_TriplarisCumingiana
TriplarisCumingiana 105738 106416 104_ndhB_Intron_TriplarisCumingiana
TriplarisCumingiana 106417 107193 105_ndhB_Exon_TriplarisCumingiana
TriplarisCumingiana 107526 107993 106_rps7_Exon_TriplarisCumingiana
TriplarisCumingiana 108047 108073 107_rps12_Exon_TriplarisCumingiana
TriplarisCumingiana 108074 108600 108_rps12_Intron_TriplarisCumingiana
TriplarisCumingiana 108601 108831 109_rps12_Exon_TriplarisCumingiana
TriplarisCumingiana 110673 110744 110_trnV-GAC_tRNA_TriplarisCumingiana
TriplarisCumingiana 110972 112462 111_rrn16_rRNA_TriplarisCumingiana
TriplarisCumingiana 112755 112842 112_trnI-GAU_tRNA_TriplarisCumingiana
TriplarisCumingiana 112791 112806 113_trnI-GAU_Intron_TriplarisCumingiana
TriplarisCumingiana 113849 113953 114_trnA-UGC_tRNA_TriplarisCumingiana
TriplarisCumingiana 113886 113925 115_trnA-UGC_Intron_TriplarisCumingiana
TriplarisCumingiana 114882 115435 116_rrn23-fragment_rRNA_TriplarisCumingiana
TriplarisCumingiana 115441 117693 117_rrn23_rRNA_TriplarisCumingiana
TriplarisCumingiana 117792 117894 118_rrn4.5_rRNA_TriplarisCumingiana
TriplarisCumingiana 118146 118266 119_rrn5_rRNA_TriplarisCumingiana
TriplarisCumingiana 118528 118601 120_trnR-ACG_tRNA_TriplarisCumingiana
TriplarisCumingiana 119220 119291 121_trnN-GUU_tRNA_TriplarisCumingiana
TriplarisCumingiana 119856 125586 122_ycf1_Exon_TriplarisCumingiana
TriplarisCumingiana 125813 128023 123_ndhF_Exon_TriplarisCumingiana
TriplarisCumingiana 129061 129234 124_rpl32_Exon_TriplarisCumingiana
TriplarisCumingiana 130500 130579 125_trnL-UAG_tRNA_TriplarisCumingiana
TriplarisCumingiana 130782 131658 126_ccsA_Exon_TriplarisCumingiana
TriplarisCumingiana 131876 133378 127_ndhD_Exon_TriplarisCumingiana
TriplarisCumingiana 133518 133763 128_psaC_Exon_TriplarisCumingiana
TriplarisCumingiana 134015 134320 129_ndhE_Exon_TriplarisCumingiana
TriplarisCumingiana 134551 135081 130_ndhG_Exon_TriplarisCumingiana
TriplarisCumingiana 135451 135939 131_ndhI_Exon_TriplarisCumingiana
TriplarisCumingiana 136052 136592 132_ndhA_Exon_TriplarisCumingiana
TriplarisCumingiana 136593 137737 133_ndhA_Intron_TriplarisCumingiana
TriplarisCumingiana 137738 138288 134_ndhA_Exon_TriplarisCumingiana
TriplarisCumingiana 138290 139462 135_ndhH_Exon_TriplarisCumingiana
TriplarisCumingiana 139570 139842 136_rps15_Exon_TriplarisCumingiana
TriplarisCumingiana 140176 145906 137_ycf1_Exon_TriplarisCumingiana
TriplarisCumingiana 146471 146542 138_trnN-GUU_tRNA_TriplarisCumingiana
TriplarisCumingiana 147161 147234 139_trnR-ACG_tRNA_TriplarisCumingiana
TriplarisCumingiana 147496 147616 140_rrn5_rRNA_TriplarisCumingiana
TriplarisCumingiana 147868 147970 141_rrn4.5_rRNA_TriplarisCumingiana
TriplarisCumingiana 148069 150321 142_rrn23_rRNA_TriplarisCumingiana
TriplarisCumingiana 150327 150880 143_rrn23-fragment_rRNA_TriplarisCumingiana
TriplarisCumingiana 151809 151913 144_trnA-UGC_tRNA_TriplarisCumingiana
TriplarisCumingiana 151837 151876 145_trnA-UGC_Intron_TriplarisCumingiana
TriplarisCumingiana 152920 153007 146_trnI-GAU_tRNA_TriplarisCumingiana
TriplarisCumingiana 152956 152971 147_trnI-GAU_Intron_TriplarisCumingiana
TriplarisCumingiana 153300 154790 148_rrn16_rRNA_TriplarisCumingiana
TriplarisCumingiana 155018 155089 149_trnV-GAC_tRNA_TriplarisCumingiana
TriplarisCumingiana 156931 157161 150_rps12_Exon_TriplarisCumingiana
TriplarisCumingiana 157162 157688 151_rps12_Intron_TriplarisCumingiana
TriplarisCumingiana 157689 157715 152_rps12_Exon_TriplarisCumingiana
TriplarisCumingiana 157769 158236 153_rps7_Exon_TriplarisCumingiana
TriplarisCumingiana 158569 159345 154_ndhB_Exon_TriplarisCumingiana
TriplarisCumingiana 159346 160024 155_ndhB_Intron_TriplarisCumingiana
TriplarisCumingiana 160025 160780 156_ndhB_Exon_TriplarisCumingiana
TriplarisCumingiana 161346 161426 157_trnL-CAA_tRNA_TriplarisCumingiana
TriplarisCumingiana 162280 168589 158_ycf2_Exon_TriplarisCumingiana
TriplarisCumingiana 168986 169059 159_trnM-CAU_tRNA_TriplarisCumingiana
TriplarisCumingiana 169269 169547 160_rpl23_Exon_TriplarisCumingiana
TriplarisCumingiana 169566 169956 161_rpl2_Exon_TriplarisCumingiana
TriplarisCumingiana 169957 170621 162_rpl2_Intron_TriplarisCumingiana
TriplarisCumingiana 170622 171055 163_rpl2_Exon_TriplarisCumingiana
TriplarisCumingiana 171114 171221 164_rps19-fragment_Exon_TriplarisCumingiana
